# MicroRNA in diagnosis and therapy monitoring of early-stage triple-negative breast cancer

**DOI:** 10.1038/s41598-018-29917-2

**Published:** 2018-08-02

**Authors:** Mustafa Kahraman, Anne Röske, Thomas Laufer, Tobias Fehlmann, Christina Backes, Fabian Kern, Jochen Kohlhaas, Hannah Schrörs, Anna Saiz, Cassandra Zabler, Nicole Ludwig, Peter A. Fasching, Reiner Strick, Matthias Rübner, Matthias W. Beckmann, Eckart Meese, Andreas Keller, Michael G. Schrauder

**Affiliations:** 1 Clinical Bioinformatics, Saarland University, Homburg, Germany; 2 Hummingbird Diagnostics GmbH, Heidelberg, Germany; 3 Friedrich-Alexander University Erlangen-Nürnberg (FAU), Department of Obstetrics and Gynecology, Erlangen University Hospital, Comprehensive Cancer Center Erlangen-EMN, Erlangen, Germany; 4 Department of Human Genetics, Saarland University, Homburg, Germany

## Abstract

Breast cancer is a heterogeneous disease with distinct molecular subtypes including the aggressive subtype triple-negative breast cancer (TNBC). We compared blood-borne miRNA signatures of early-stage basal-like (cytokeratin-CK5-positive) TNBC patients to age-matched controls. The miRNAs of TNBC patients were assessed prior to and following platinum-based neoadjuvant chemotherapy (NCT). After an exploratory genome-wide study on 21 cases and 21 controls using microarrays, the identified signatures were verified independently in two laboratories on the same and a new cohort by RT-qPCR. We differentiated the blood of TNBC patients before NCT from controls with 84% sensitivity. The most significant miRNA for this diagnostic classification was miR-126-5p (two tailed t-test p-value of 1.4 × 10^−5^). Validation confirmed the microarray results for all tested miRNAs. Comparing cancer patients prior to and post NCT highlighted 321 significant miRNAs (among them miR-34a, p-value of 1.2 × 10^−23^). Our results also suggest that changes in miRNA expression during NCT may have predictive potential to predict pathological complete response (pCR). In conclusion we report that miRNA expression measured from blood facilitates early and minimally-invasive diagnosis of basal-like TNBC. We also demonstrate that NCT has a significant influence on miRNA expression. Finally, we show that blood-borne miRNA profiles monitored over time have potential to predict pCR.

## Introduction

Breast cancer (BC) is a major health problem and is the most common cancer in women worldwide, affecting 12% of all women and leading to 450,000 deaths each year^1^. Early detection is crucial, as timely treatment leads to a higher survival rate. Various approaches, ranging from self-examination of the breast to mammography screening, are aimed at detecting BC as early as possible. However, the precision of all the methods currently used is limited, and particularly in younger women, mammography is often difficult to interpret due to high-density breast tissue. Reliable and early-stage blood-based biomarkers to support imaging methods of BC detection have been explored extensively in recent years. Potential molecular marker candidates include gene expressions, proteins, as well as miRNA expression signatures. An effective blood-based diagnostic test might not only increase the specificity and sensitivity of BC screening and act as a rule-in factor for further diagnostic imaging procedures such as magnetic resonance imaging (MRI), but might also increase patient compliance and acceptance of preventive medical check-ups.

Early diagnosis and the implementation of (neo-)adjuvant chemotherapy and endocrine therapy have improved the treatment of BC, but a significant proportion of BC patients develop recurrent or metastatic disease^2^. As BC is a very heterogeneous disease with distinct morphologies, molecular traits, prognoses, and treatment options, clinical decisions are mainly made on the basis of the tumour stage, lymph-node involvement, and molecular subtype, represented clinically by the expression of estrogen receptor (ER), progesterone receptor (PR), HER2 receptor (HER), and the proliferation marker Ki-67 (Mib-1)^3^. Endocrine therapy is indicated in the presence of ER+ and/or PR+ tumours, while the most suitable treatment for HER2+ cancer patients includes targeted therapy^4^. Neoadjuvant chemotherapy (NCT) administered before surgery for breast cancer is often used in patients with locally advanced BC and in patients with aggressive BC subtypes such as triple-negative breast cancer (TNBC), characterized by absence of ER, PR, and HER2. However, the outcome for patients with TNBC is rather poor, especially in the subgroup who do not achieve a pathological complete response (pCR) with NCT^5,6^. Predicting the response to NCT is therefore a critical issue that needs to be addressed, particularly in patients with TNBC.

MiRNAs are small, non-coding RNAs, 17–27 nucleotides in length. They exert regulatory functions on the expression of multiple genes by initiating translational silencing or degradation of their cognate mRNA targets. Accumulating evidence indicates that miRNA expression patterns are tissue-specific and reflect pathophysiological processes such as tumorigenesis, metastasis, and drug responsiveness in their cells of origin. Moreover, miRNAs can be detected not only in tissue samples but also in blood, serum, urine, and other sources that are accessible with minimal invasiveness. Extensive research has demonstrated that miRNAs are dysregulated at all stages of BC and could have a potential role as prognostic and predictive biomarkers^7,8^. Especially serum profiles have been explored^9–11^ and are promising candidates for detecting BC, also having a substantial potential as prognostic markers.

It has previously been reported by our group that also the expression of miRNAs originating from blood cells is generally linked to diseases^12,13^, and miRNA signatures represent potential novel biomarkers for early and minimally-invasive diagnosis. In a proof-of-concept study using ethylenediamine tetraacetic acid (EDTA) as anticoagulant in the blood collections systems we have demonstrated that blood-borne miRNAs may serve as diagnostic blood-based biomarkers of early-stage BC^14^. A short-coming of EDTA is the detected variability in miRNA expression depending on the exposure time of the blood to the anticoagulant^15^. Other collection systems such as PAXgene Blood RNA tubes showed a better stabilization of cellular ribonucleic acids in blood^16^.

The present study analysed miRNA expression profiles in patients with basal-like triple-negative breast cancer before and after NCT, in order to compare these profiles with each other and with those of age-matched healthy women. The aim was to identify miRNAs, miRNA profiles, and miRNA profile changes that are capable of predicting a pathological complete response after NCT. To minimize errors associated with sampling and sample preparation, we collected the patients’ blood samples for this study in PAXgene RNA blood tubes.

## Methods

### Study set-up

All of the patients included in this study were participating in a prospective case–control study on the *mo*lecular *de*tection of *b*reast cancer (the MODE-B Study) and the follow-up study, iMODE-B. Patients with suspicious diagnostic findings or suspected BC who had been referred to the specialized breast unit at the University Breast Center for Franconia in Erlangen University Hospital underwent further diagnosis using ultrasound-guided high-speed core-needle biopsies. Venipuncture was done before the biopsies and again after NCT was completed. A total of 100 blood samples (2.5 mL per patient) were collected in PAXgene tubes for the different microarray and validation cohorts. After arrival at Hummingbird Diagnostics GmbH (Heidelberg, Germany), the blood samples were stored at −80 °C until RNA extraction.

### Patient characteristics

All patients included in the study had been diagnosed with early-stage basal-like (cytokeratin CK5-positive) triple-negative breast cancer (TNBC). The clinical data for the patients can be found in Table 1. All patients had a tumour size of less than 5 cm (≤pT2), were free of distant metastases, and were treated with NCT, including six cycles of carboplatin (AUC 5) every 3 weeks with parallel weekly treatment with paclitaxel (80 mg/m^2^ body surface) for 18 weeks. The second venipuncture was performed after NCT and before surgery.

**Table 1:** Characteristics of the patients.

Mean age at TNBC diagnosis	58.43 years
Mean age of healthy controls	58.33 years
Proportion of patients with pCR	48% (11 out of 21)
Proportion of patients with positive lymph nodes after NCT	14% (3 out of 21)
Histological tumour type in all patients	Non-special type (NST)
Molecular tumour subtype in all patients	Basal-like TNBC
Mean proliferation index (Ki-67)	63%
Mean positivity for CK5	65%
Neoadjuvant chemotherapy regimen in all patients	Carboplatin and paclitaxel

CK, cytokeratin; NCT, neoadjuvant chemotherapy; pCR, pathological complete response; TNBC, triple-negative breast cancer.

### Ethics statement

The MODE-B and iMODE-B studies were approved by the Ethics Committee of the Medical Faculty of Friedrich Alexander University of Erlangen–Nuremberg (FAU; reference numbers 3937 and 4514). Written informed consent was obtained from every patient and healthy control individual before the blood was taken.

### miRNA extraction

Prior to RNA extraction, PAXgene tubes were thawed overnight at room temperature to ensure complete lysis of blood cells. Total RNA, including miRNA, was extracted and purified using the PAXgene Blood miRNA Kit in accordance with the manufacturer’s instructions (Qiagen GmbH, Hilden, Germany). Quantification of purified RNA was performed with NanoDrop 1000 (Thermo Fisher Scientific, Waltham, Massachusetts, USA). The quality and integrity of the RNA (RIN value) was evaluated using Agilent Bioanalyzer and the Nano RNA Kit in accordance with the manufacturer’s protocols (Agilent Technologies, Santa Clara, California, USA).

### miRNA measurement on microarrays

For microRNA expression, profiling samples were analysed on Agilent Sureprint G3 Human miRNA (8 × 60k) microarray slides with the latest miRBase v21 content. Each array targets 2,549 microRNAs with 20 replicates per probe. Extracted microRNA was labeled and hybridized using the miRNA Complete Labeling and Hybridization Kit from Agilent, in accordance with the manufacturer’s protocol (Agilent Technologies, Santa Clara, California, USA). After rotating hybridization for 20 hours at 55 °C, the slides were washed twice and scanned on Agilent’s SureScan Microarray Scanner. Image files from the scanner were transformed into text raw data using Feature Extraction Software (Agilent Technologies) for bioinformatics analysis.

### Two staged validation using RT-qPCR

Validation of the miRNAs was carried out using reverse transcription quantitative polymerase chain reaction (RT-qPCR) on eight selected miRNAs (hsa-miR-101-3p, hsa-miR-126-3p, hsa-miR-126-5p, hsa-miR-144-3p, hsa-miR-144-5p, hsa-miR-301a-3p, hsa-miR-664b-5p, hsa-miR-93-5p). RNU48 was measured as an endogenous control using the SYBR Green approach. For comparison to other assays we carried out a second validation. In more detail, total RNA was isolated using the Maxwell (Promega) system. Two ng of each RNA was reverse transcribed using TaqMan MicroRNA reverse transcription kit (Applied Biosystems) according to manufactures protocol. The miRNA qPCR reaction was performed for the following housekeeping miRNAs RNU6B and RNU48 and the target miRNA hsa-miR34a-5p with TaqMan Universal Master mix II (without Uracil N-glycosylase) (Applied Biosystems) for 40 cycles using a StepOnePlus (Applied Biosystems) according to manufactures protocol.

### Bioinformatics evaluation

For data processing, on the one hand, the profiled samples were subjected to variance stabilizing normalization (VSN). On the other hand, only miRNAs with present calls in at least three patients were considered. A total of 946 of the 2,549 human miRNAs available on the Agilent miRBase v21 arrays thus remained for the bioinformatics analysis. For subsequent data analysis three methods were applied: unsupervised clustering, statistical analysis by using hypothesis tests, and supervised classification. To identify potential relationships or similarities in the miRNA expression data, a hierarchical clustering method was performed using the standard Euclidian distance metric. For the statistical analysis to identify biomarker candidates among the three groups (controls, pre-chemotherapy, and post-chemotherapy), pairwise and multiple comparisons were applied. For pairwise comparisons, the *t*-test was used for comparisons between the control group and each one of the cancer-related groups, and a paired *t*-test for comparing pre-NCT and post-NCT samples taken from the same cancer patients. In addition, multiple comparison was also carried out using the analysis of variance (ANOVA) test. All *p-values* underwent Benjamini–Hochberg adjustment to control the false discovery rate. In addition to the significance values, the area under the receiver operating characteristics curve (AUC) value was computed. For further analysis, radial basis function support vector machines were applied to classify samples as cases or controls respectively. These were evaluated using 20 independent repetitions of fivefold cross-validation, and a filter as a subset selection method was applied on the basis of the significance of miRNAs. Finally, a pathway analysis of the statistically significant deregulated miRNAs was performed. Using the online tool miRPathDB^17^, pathways that are enriched with target genes of the significant miRNA markers were searched. Both, experimentally validated as well as predicted targets were considered and compared in this analysis step.

For RT-qPCR analysis of the miRNAs, a technical and biological replication was performed. To make the two data sets comparable with each other, each cohort was scaled to a mean value of zero and unit variance.

### Ethical approval

Both studies MODE-B and iMODE-B were approved by the Ethics Committee of the Medical Faculty of the Friedrich-Alexander University Erlangen-Nürnberg (FAU), Germany (reference numbers 3937 and 4514). All procedures performed in these studies involving human participants were in accordance with the ethical standards of the institutional research committee, and with the 1964 Helsinki declaration and its later amendments.

### Informed consent

Informed consent was obtained from all individual participants (patients and controls) included in this study.

### Availability of data and material

All data generated or analyzed during this study are included in this published article or submitted to a public repository (Gene Expression Omnibus, GEO).

## Results

To study the impact of platinum-based NCT on microRNA expression, 63 blood samples were investigated from 21 patients with early-stage basal-like TNBC, taken before and after NCT, and from 21 healthy age-matched women. This was accomplished by comparing the controls, pre-NCT, and post-NCT profiles using clustering and statistical analysis. Subsequent, RT-qPCR validation steps were performed in the same cohort and in a new study group of patients with basal-like TNBC and controls.

### General analysis of the three cohorts

**Figure 1:**
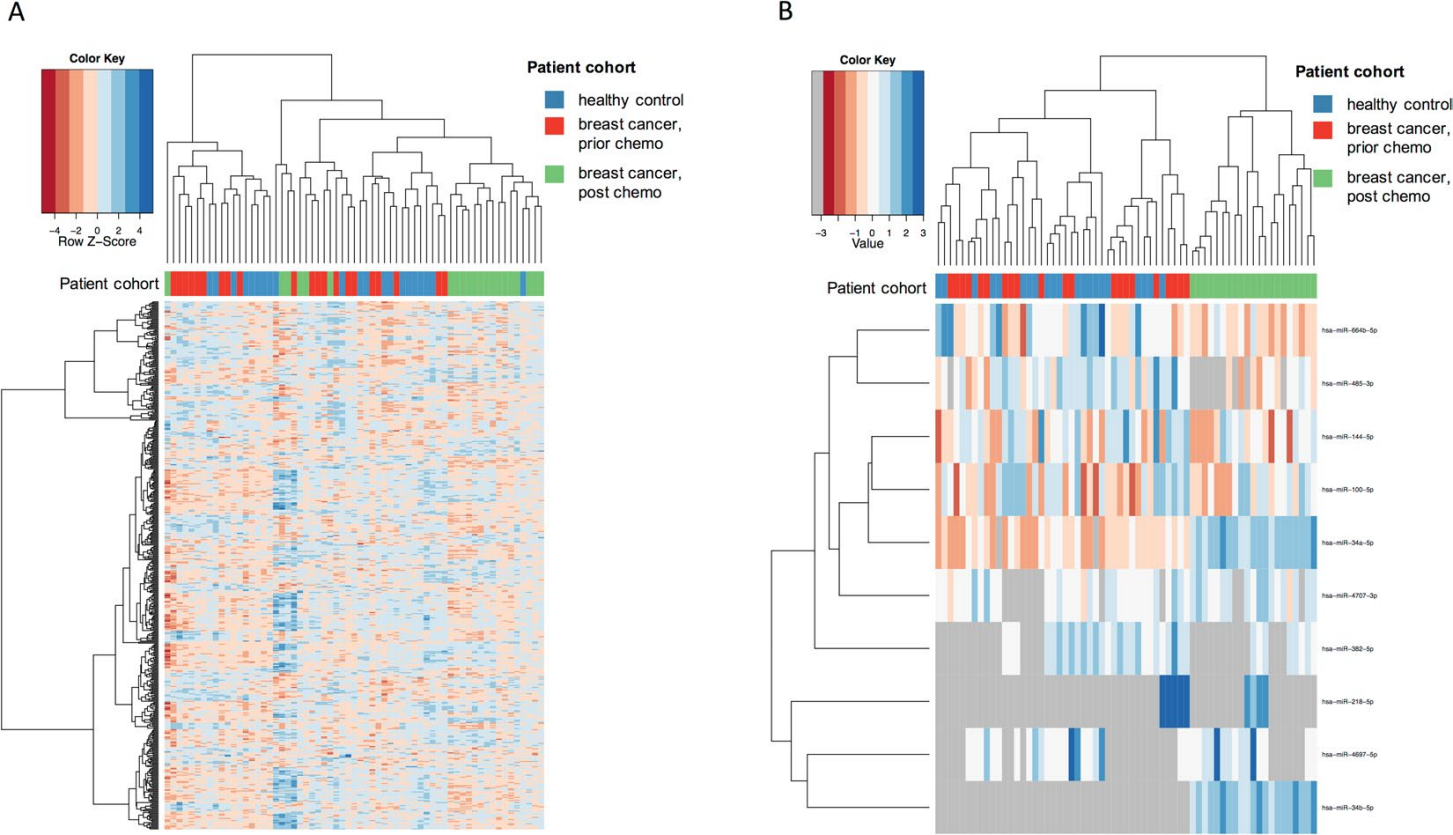
Heat map of the hierarchical clustering. The dendrogram on top shows the clustering of patients, and the dendrogram on the side shows the clustering of miRNAs. The colours on top of the heat map represent the cohorts: controls and patients before and after neoadjuvant chemotherapy. The colours in the heat map represent miRNA expression intensities, scaled to mean of zero and unit variance for each miRNA. Panel A presents the heat map for all miRNAs while panel B focuses on the 10 miRNAs with the overall highest data variance.

In a first analysis we evaluated the three cohorts (controls, matched patients prior to NCT and the same patients after NCT) together. Hierarchical clustering based on all measured miRNAs, as depicted in Fig. 1A, showed that the samples from TNBC patients following NCT (green) had a tendency to cluster together, while controls (blue) and pre-NCT (red) samples showed generally divergent patterns. Since many low-expression miRNAs without significant variability contain limited diagnostic information, but may add noise to the cluster patterns, a cluster analysis was also performed with the 10 most variable miRNAs across all samples (Fig. 1B). Here, miRNAs such as hsa-miR-34a-5p and hsa-miR-34b-5p were only present or significantly highly expressed in the post-NCT samples. These miRNAs consequently led to clustering in which post-NCT samples gather in one cluster, while healthy controls and pre-NCT samples build a separate second cluster. The expression of the miRNAs with the greatest overall variance appears to be more affected by NCT than by the development of cancer.

**Figure 2:**
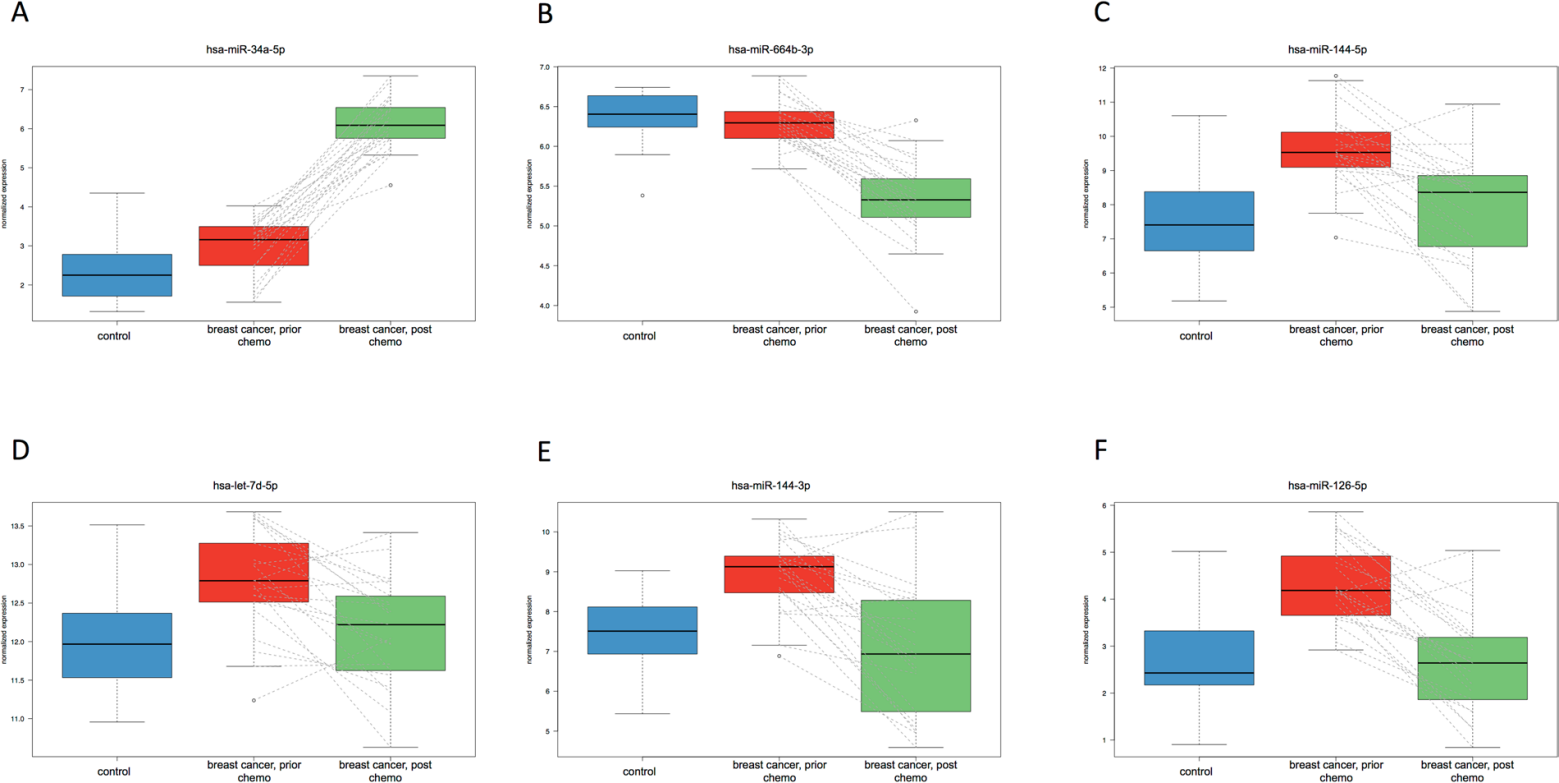
For selected examples, miRNA expression in the three cohorts is shown as box plots. The colours of the cohorts are matched to the colours in Fig. 1. Dashed lines between the patients before and after therapy indicate the individual (paired) changes in miRNA during therapy. The box plots for all significant miRNAs in the ANOVA are available in the supplemental material.

One way to detect whether a miRNA is significantly deregulated in at least one of the three cohorts is an analysis of variance (ANOVA). The ANOVA test between the three groups yielded 389 significant miRNAs prior to adjustment for multiple testing and 274 statistically significant deregulated miRNAs following the adjustment for multiple testing, which are presented with the respective *p-values* in Table S1. Figure 2A–F presents selected examples of miRNA expression in the three cohorts as box-plots. The Box-plots of all significant miRNAs in the ANOVA are shown in the supplemental material (Supplemental Fig. 1). For the respective miRNAs the ANOVA yielded significant *p-values* (*p-values* adjusted for multiple testing below the alpha level of 0.05; miR-34a-5p: 1.2 × 10^−23^; miR-664b-3p: 1.1 × 10^−9^; hsa-miR-144-3p: 8.8 × 10^−22^; hsa-miR-144-5p: 0.0004; hsa-miR-126-5p: 2.7 × 10^−5^; hsa-let-7d-5p 0.01). For miR-34a-5p (Fig. 2A), expression was highest post-NCT, while pre-NCT samples and controls had similar expression rates. On the other hand, miR-664b-3p was down-regulated following NCT (Fig. 2B). For the remaining four miRNAs, hsa-miR-144-3p, hsa-miR-144-5p, hsa-miR-126-5p, and hsa-let-7d-5p (Fig. 2C–F), the expression ratio in TNBC patients prior to NCT was significantly increased, while post-NCT signatures matched to the controls. These miRNAs are of particular interest, as they show a pre-NCT “cancer pattern” that returns to a “healthy control pattern” after NCT.

### Pairwise comparisons of the three cohorts

**Figure 3:**
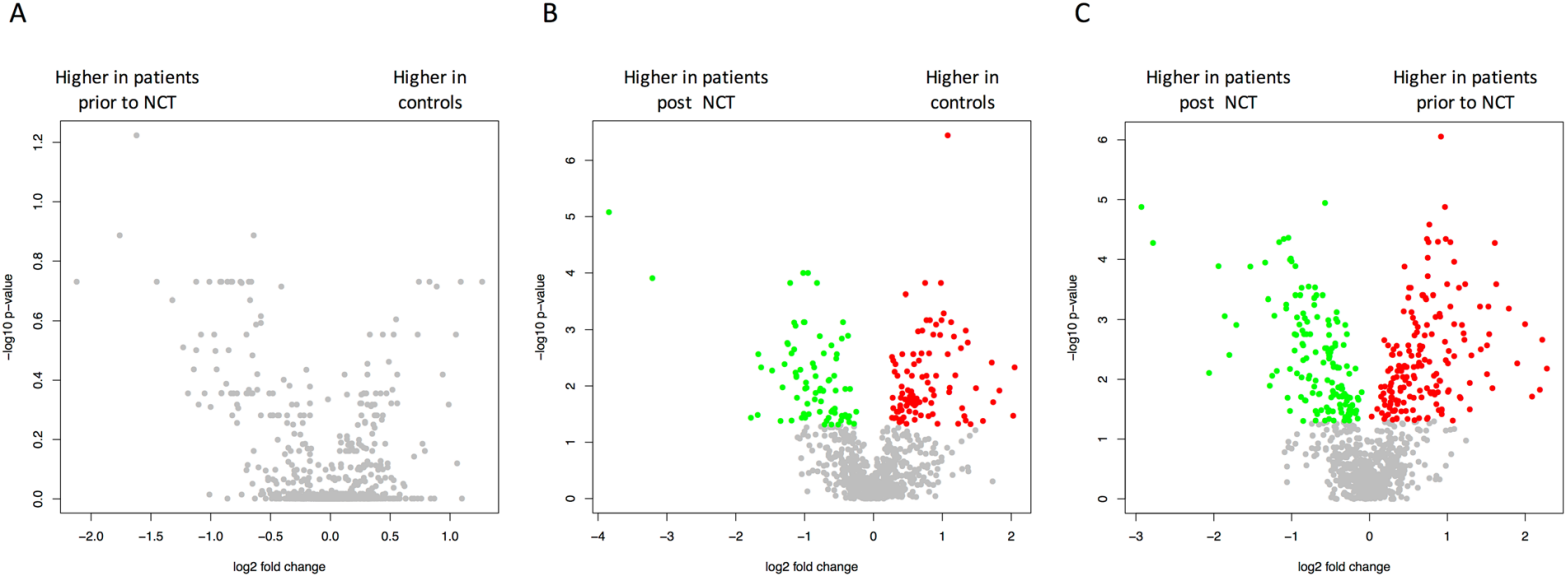
Volcano plots (x-axis represents the log2 of the fold change, y-axis represents the negative decade logarithm of the significance value) for the three different comparisons. Each dot represents a single miRNA. Significantly up-regulated miRNAs are highlighted in red and significantly down-regulated miRNAs in green. Panel A presents the comparison of breast cancer prior to NCT to control, panel B the comparison of breast cancer following NCT to controls and panel C the comparison of breast cancer post versus prior NCT.

To identify differences between the three cohorts, all three possible pairwise comparisons were carried out in addition to the ANOVA. Again, miRNAs with adjusted significance values below the alpha level of 0.05 were considered significant. As the volcano plots (Fig. 3A–C) indicate, 180 statistically significantly deregulated miRNAs were discovered between controls and the post-NCT group, but no significantly deregulated miRNAs between controls and the pre-NCT group. To assess if there exist any miRNAs with a diagnostic potential towards the latter case, the according raw *p-values* were taken into consideration. Here, 95 miRNAs were significant, but given the large number of features in comparison with the limited cohort size, the respective miRNAs lost significance during the adjustment (the *p* ≫ n problem). The largest number of significantly different miRNAs was observed for the comparison between pre-NCT and post-NCT samples. Since these samples were taken from the same patients before and after NCT, a paired *t*-test was applied. Here, 321 significantly deregulated miRNAs were detected following adjustment for multiple testing. The raw and adjusted *p-values* together with the area under the receiver operator characteristics curve (AUC) values for all miRNAs are listed in Table S2.

These statistical comparisons basically confirmed the findings of the hierarchical clustering, and the expressions are presented in box plots in Fig. 2. The large numbers of significantly deregulated miRNAs reflect the substantial difference in hierarchical clustering and expression between the post-NCT group, on the one hand, and the healthy control and pre-NCT groups on the other. For detection of BC, the four miRNAs previously mentioned (hsa-miR-144-3p, hsa-miR-144-5p, hsa-miR-126-5p, and hsa-let-7d-5p) that returned to normal expression after NCT were significantly deregulated both in the comparisons of “controls vs. pre-NCT” (raw *p* value) and “pre-NCT vs. post-NCT” (adjusted *p* value). These miRNAs appear to be reasonable candidates for minimally invasive detection of basal-like TNBC and therapy monitoring.

### Differentiation between basal-like TNBC patients and healthy women using miRNA profiles

**Figure 4:**
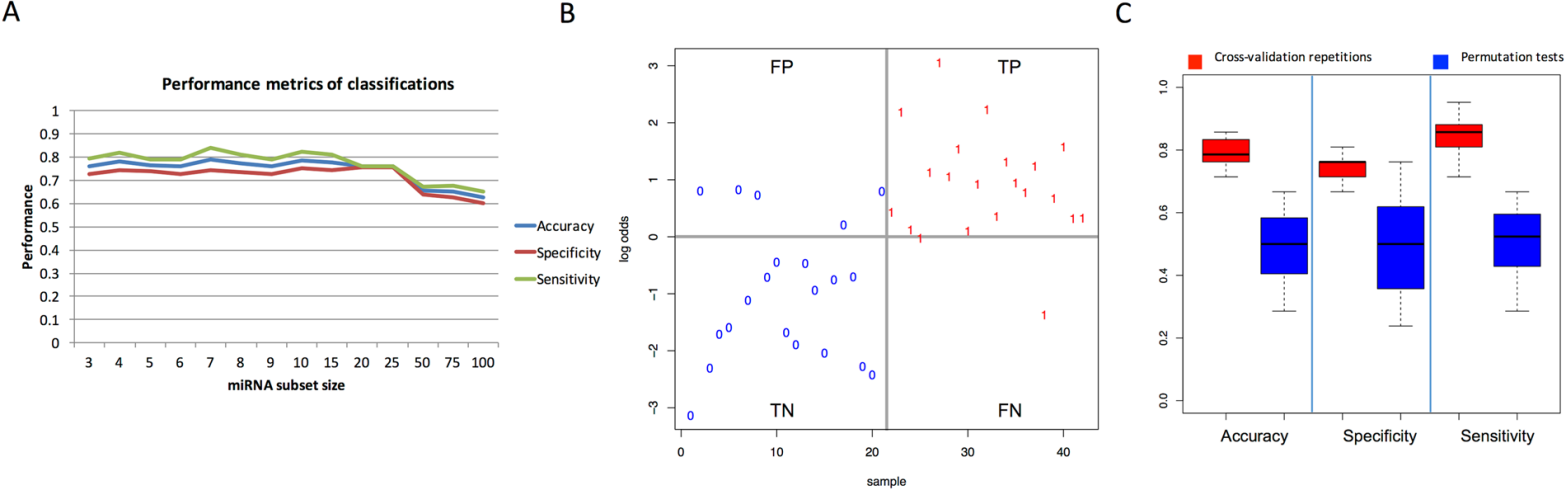
Classification results. Panel A shows the performance (y-axis) of the classification for different subset sizes from 3 to 100 miRNAs (x-axis). For smaller subset sizes, the performance remains constantly high; for larger sets of more than 25 miRNAs, the performance continuously decreases. Panel B presents the classification results for the best repetition of the cross-validation. The y-axis shows the quotient of the logarithm of probabilities for being diseased and for being healthy. Samples on the horizontal line are equally likely to be healthy and diseased, samples above are more likely to be diseased, and samples below are more likely to be healthy. Individuals marked “0” are controls; individuals marked “1” represent breast cancer (TNBC) samples. Panel C shows for the seven miRNA signature all repeated cross-validation runs as box plots (red). The performance of permutation tests is presented in blue, along with the true classification performance.

Since single miRNAs appear to have a limited potential for BC detection, several combinations of miRNAs were evaluated. The selection of the most reasonable candidate miRNAs for blood-based differentiation of patients with basal-like TNBC and healthy women was one of the reasons for performing microarray analyses of the entire set of human miRNAs currently known (miRBase version 21). From all the marker candidates, a suitable miRNA signature for distinguishing between healthy women and TNBC patients was computed using support vector machines. For classification, different subsets of the most deregulated miRNAs were tested in 20 repeated runs of fivefold cross-validations to discriminate TNBC cases from controls (Fig. 4A). During testing of different subset sizes (smallest: 3, largest: 100), a set of seven miRNAs achieved the highest average performance values across the repeated cross-validation runs: the statistical model reached an accuracy of 79%, a specificity of 74.2%, a sensitivity of 83.8%, and an AUC value of 0.814. Interestingly, a subset size of up to 25 markers showed stable performance, whereas larger sets led to a reduced performance. The seven selected markers for the best performance according to accuracy were hsa-miR-126-5p, hsa-miR-144-5p, hsa-miR-144-3p, hsa-miR-301a-3p, hsa-miR-126-3p, hsa-miR-101-3p, and hsa-miR-664b-5p. Among all cross-validation runs, the maximal performance was 85.7% accuracy, 76.2% specificity, and 95.2% sensitivity (Fig. 4B). Only five false-positive and two false-negative cases were observed here.

To recognize potential overtraining, the performance values for the cross-validations of the seven markers were compared with results from permutation tests with random class labelling, as shown in Fig. 4C. Importantly, the subset of seven miRNAs yields average performance values of 50% for the permutation tests in accuracy, specificity, and sensitivity, as expected.

### Validation of blood-borne breast cancer miRNAs by RT-qPCR

To facilitate measurement of miRNA signatures in larger cohorts of patients RT-qPCR represents a reasonable experimental approach. To ensure that the miRNAs that have been discovered as biomarkers can be measured using RT-qPCR, we carried out a two-staged validation using two different RT-qPCR approaches (SYBR-Green and TaqMan) independently in two different laboratories.

For the first iteration of the validation we have chosen the following eight miRNAs for this validation step: hsa-miR-101-3p, hsa-miR-126-3p, hsa-miR-126-5p, hsa-miR-144-3p, hsa-miR-144-5p, hsa-miR-301a-3p, hsa-miR-664b-5p, and hsa-miR-93-5p. These include the seven miRNAs from the breast cancer detection signature mentioned in the previous paragraph. As additional marker we have selected miR-93-5p as a miRNA that was not significantly different prior to and following chemotherapy but significant between cancer cases and controls. RNU48 was measured as an endogenous control. The miRNAs were analyzed in 34 healthy women (including 15 controls as technical replicates from the microarray analysis and 19 new control samples) and 31 patients with basal-like TNBC (including 13 TNBC cases as technical replicates from the microarray analysis and 18 new TNBC patients). With regard to the cycle threshold (Ct) values for each miRNA, as well as the delta Ct values in comparison with the endogenous control, concordant dysregulation was observed for each of the eight miRNAs tested in the biological and the technical validation cohort.

**Figure 5:**
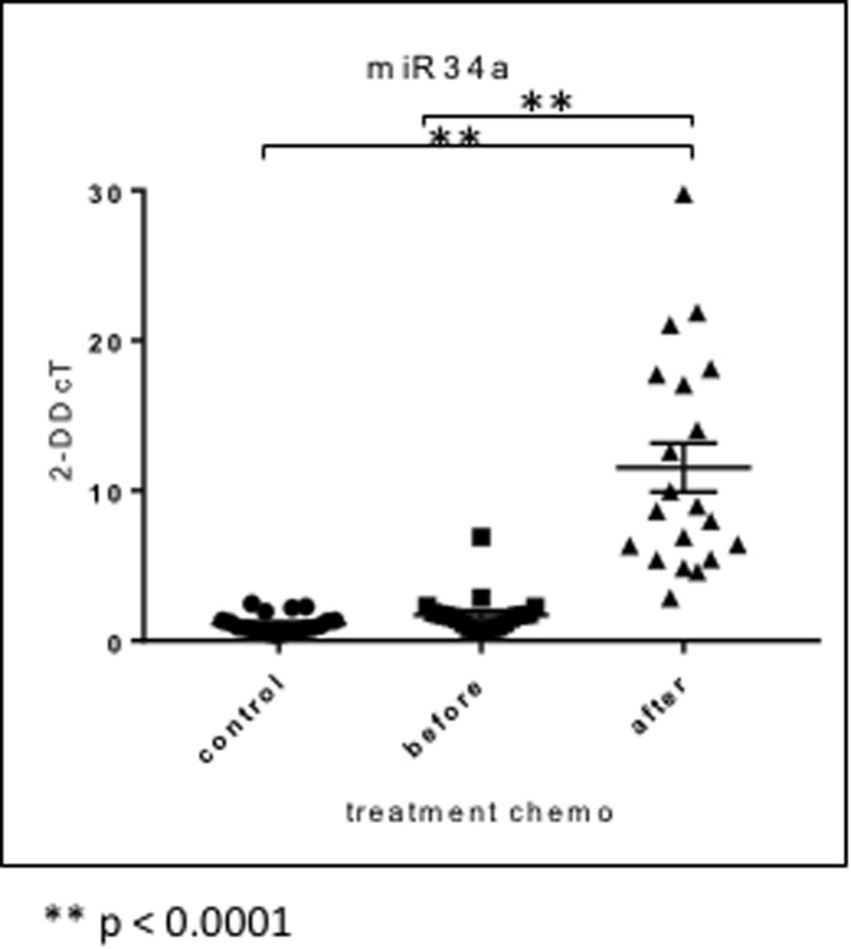
TaqMan RT-qPCR validation results for miR34a show a highly significant (p < 0.0001) difference of blood miR34a expression levels before and after, platinum-based neoadjuvant chemotherapy in patients with TNBC (consistent with the previous microarray results).

As a further validation we performed measurements using the TaqMan system as described in the methods section. One of the core miRNA families in oncology is the miR-34 family. Since we already validated many targets of the miR-34a family and proved the role in gene regulation using systems biology approaches^18,19^ we also performed the validation experiments with this miRNA. As for the initial microarray analysis we observed a highly significant dys-regulation for patients after chemotherapy (adjusted p-value < 0.001, 10-fold increased expression; Fig. 5).

### Pathway and tissue-of-origin analysis reveals miRNAs targeting important genes in basal-like TNBC

On the basis of the statistically significantly deregulated miRNAs, the “WikiPathways” database was searched for pathways that are targeted by miRNAs. Specifically, the focus was on experimentally validated targets and the “Integrated Breast Cancer Pathway” in WikiPathways (http://www.wikipathways.org/index.php/Pathway:WP1984). To this end proper information from the miRNA pathway dictionary database miRPathDB^17,20^ was considered. A miRNA was considered as “pathway-specific” when the database returned at most ten pathways out of all tested 252 pathways to be significantly regulated by this miRNA. By applying this criterion, 1–3% pathway-specific miRNAs were obtained on the basis of the significantly deregulated miRNA sets with the sizes 95 (controls vs. pre-NCT), 180 (controls vs. post-NCT), 321 (pre-NCT vs. post-NCT), and 274 (ANOVA). In detail, the miRNAs shown in Supplemental Fig. 2A–D were considered as pathway-specific. Notably, some of these miRNAs were observed in several of the statistical comparisons presented above. Controls vs. pre-NCT (hsa-miR-26b-5p, hsa-miR-18a-5p, hsa-miR-93-5p), for controls vs. post-NCT (hsa-miR-361-5p, hsa-miR-18a-5p), for pre-NCT vs, post-NCT (hsa-miR-361-5p, hsa-miR-2110, hsa-miR-224-5p, hsa-miR-100-5p, hsa-miR-365a-3p, hsa-miR-92a-3p), and for the ANOVA (hsa-miR-93-5p, hsa-miR-361-5p, hsa-miR-18a-5p, hsa-miR-185-5p) were reported as central for the “Integrated Breast Cancer Pathway”. Importantly, hsa-18a-5p is the only miRNA that shows significantly different expression in the two groups of basal-like TNBC samples (pre- and post-NCT) in comparison with controls, but without significant alteration by NCT.

Also the question on whether miRNAs are specific for one or few tissues or expressed in all tissues plays an important role for the selection of biomarkers^21^. For our breast cancer miRNAs, a literature search highlighted that those miRNAs are usually no only expressed in breast tissue. Our miRNA tissue atlas^22^ demonstrated that the miRNAs were detectable in almost all of 31 tested tissues. Only hsa-miR-664b-5p expression was not present in the following tissues gallbladder, lymph nodes, and adrenal gland.

### MiRNAs as a predictor of NCT response

**Figure 6:**
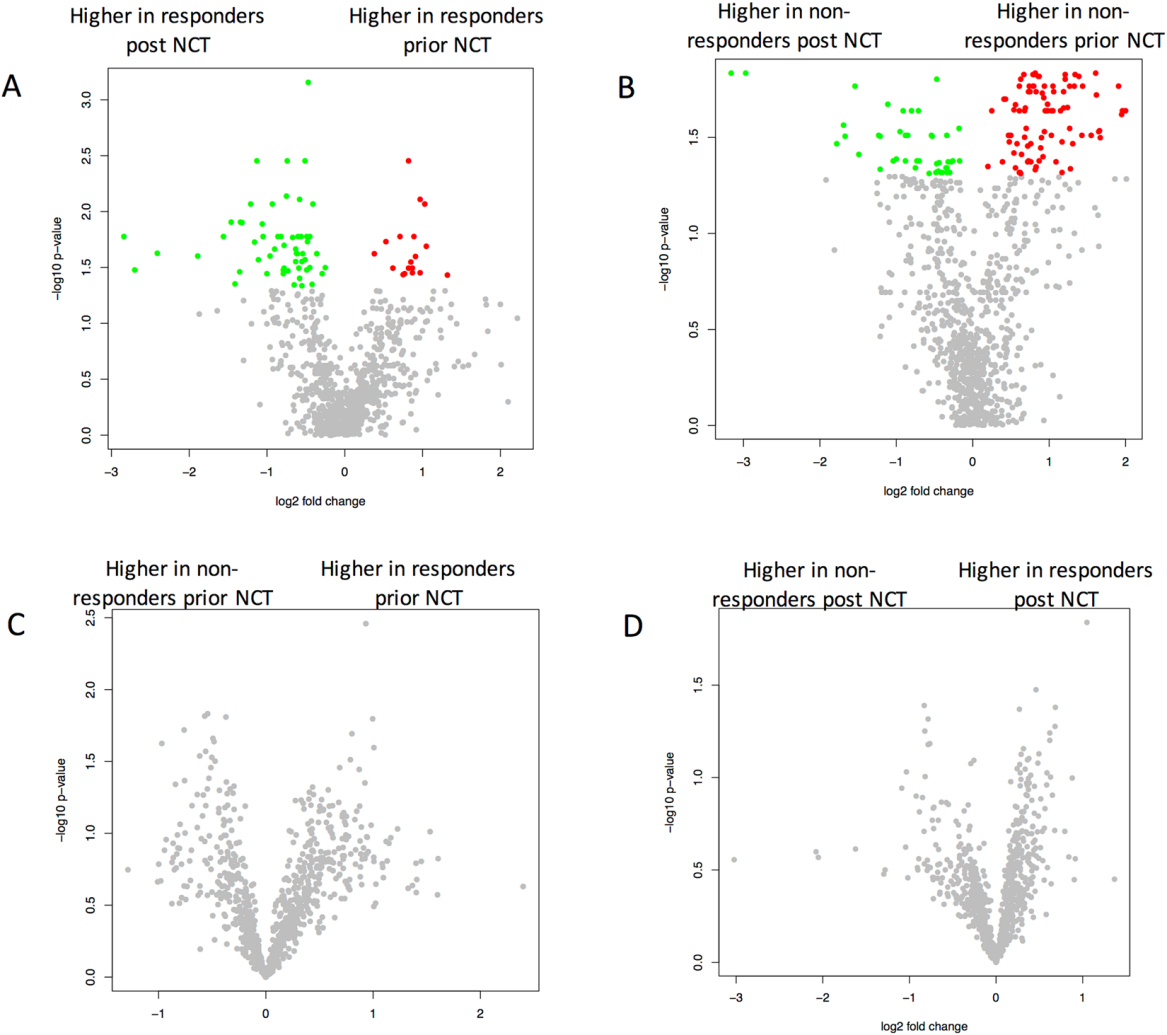
Similar to Fig. 3, volcano plots for comparisons between complete responders (pCR) and patients without pCR before and after therapy are shown here. For complete responders, a tendency toward a decrease in miRNA expression is observed, while for patients without pCR, there is a tendency toward up-regulation.

In addition to the pairwise comparisons our data also allow to search for markers that are differentially expressed in the group of responders versus non-responders. As indicator for the treatment response we considered pCR, defined as an absence of both *in situ* and invasive cancer in the breast and in the axillary lymph nodes. For this purpose, miRNA expression pre- and post-NCT was analysed and correlated with the clinical data for therapy response: the cancer samples were divided depending on whether or not a pCR was achieved after NCT. In detail, ten out of 21 cancer patients had a pCR (complete responders) and 11 did not have a pCR (Table 1). Four statistical comparisons taking into account adjusted *p-values* < 0.05 were performed to define significantly deregulated miRNAs. With regard to complete responders, pre- versus post-NCT states were compared (Fig. 6A), revealing 74 significantly deregulated miRNAs. In samples from patients who did not achieve a complete response, 131 miRNAs were significantly deregulated (Fig. 6B). In contrast, no significant deregulation was observed between the pCR and non-pCR groups when all pre-NCT samples (Fig. 6C) and post-NCT samples (Fig. 6D) were analysed. Complete responders showed a tendency to have higher miRNA levels after NCT (Fig. 6A; green dots), while patients in the non-pCR group showed a tendency toward lower expression (Fig. 6B; red dots).

These results suggest that comparing samples before chemotherapy does not allow any prediction of the success of treatment. However, monitoring the changes in blood miRNA expression during NCT might be predictive for the success of treatment.

## Discussion

The present study investigated whether blood-based miRNA patterns have sufficient statistical power in patients with basal-like TNBC to be used as a liquid biopsy method for diagnosis and for predicting the pathological complete response (pCR) after platinum-based neoadjuvant chemotherapy. The aim was to elucidate whether miRNAs can be used to differentiate between patients with basal-like TNBC and healthy women. As secondary and tertiary goals, it was investigated what effects platinum-based NCT has on miRNA expression and whether it is possible to distinguish complete responders (those who achieve a pCR) from the non-pCR group on the basis of their miRNA profile from PAXgene Blood RNA tubes. In this context pCR was used as substitute for so far outstanding long-term follow-up data of our study cohort.

Different approaches for the minimally-invasive diagnosis and prognosis prediction of breast cancer by using miRNAs have been explored. Among the most relevant ones are those relying on serum or plasma^9^ and those relying on whole blood^14^. In other diseases, blood-borne markers have shown an increased potential for the diagnosis of diseases^23^, especially for autoimmune diseases. Plasma profiles seem for example to be affected in cancer patients following tumor resection^24^. To predict the outcome or long-term prognosis tissue profiles seem however to have a very high performance^25^. The main focus of this study lies in the area of breast cancer diagnosis and therefore we selected PAXgene Blood RNA tubes as most promising specimen type. Building on experience from a previous study^14^, miRNA measurements using microarrays were performed to identify expressed and deregulated miRNAs.

In this study it was possible to identify healthy women and patients with basal-like TNBC using a seven-miRNA signature, with performance values of 74.2% specificity, 83.8% sensitivity, 79% accuracy, and an AUC value of 0.814. The performance of the different classifiers was quite stable for subset sizes of between 3 and 25 miRNAs, with a mean accuracy of 77%, a mean specificity of 74.1%, and a mean sensitivity of 79.9%. These smaller sets performed better than larger sets with subset sizes of between 25 and 100 miRNAs. Their performances showed means of 64.5% accuracy, 62.2% specificity, and 66.8% sensitivity. Importantly, the best repetition of classification showed an accuracy of 85.7%, a specificity of 76.2%, and a sensitivity of 95.2%; however, more samples are required in order to validate this seven-miRNA signature and yield these improved sensitivities repeatedly. A literature search for these seven markers (hsa-miR-126-5p, hsa-miR-144-5p, hsa-miR-144-3p, hsa-miR-301a-3p, hsa-miR-126-3p, hsa-miR-101-3p, and hsa-miR-664b-5p) showed that some of the miRNAs also occur in more general cancer-related topics — e.g., in relation to different cancer types such as breast cancer and lung, prostate, and colon cancer. In 2014, our group showed that several markers, including hsa-miR-144-5p, were related to at least 13 of 19 cancer diseases^12^. A database search with hsa-miR-126-3p and hsa-miR-101-3p as search terms in the OncomiRDB — a database for experimentally verified oncogenic and tumour-suppressive miRNAs^26^ — showed that these two miRNAs are mentioned in numerous publications as being oncogenic or tumour-suppressive regulators in various types of tissues and diseases. Most miRNAs in our signature are from very early miRBase versions, which are known to contain less false positive miRNAs as compared to later versions^27–29^.

Dysregulated expression for eight selected dysregulated miRNAs in TNBC patients was confirmed in a cohort of 65 individuals, consisting of 31 patients with basal-like TNBC and 34 healthy women. According to the results of the clustering analysis, the miRNA expression profiles of the pre-NCT samples were closer to the profiles of healthy women than the profiles of post-NCT samples. While the post-NCT samples clustered to each other, the samples for the other two groups lay in the same clusters. However, statistically significant differences between controls and pre-NCT samples were only found in relation to raw *p-values*, whereas when these two groups were compared with the post-NCT group, a substantial number of significantly deregulated miRNAs was found according to adjusted *p-values* below the alpha level of 0.05. Concentrating on the 10 most significantly deregulated miRNAs in the ANOVA test, it was shown that the pre-NCT samples had expression values similar to those of the controls. After NCT, these and other miRNAs revealed a substantial difference in their expression — indicating that NCT affects a large number of miRNAs, including non–disease-related miRNAs.

Validation using different approaches in different, independent laboratories is essential, thus we performed validation experiments not only using SYBRGreen, but also in a second laboratory using TaqMan. In all cases the results of the microarray measurements were validated.

The question arises why miRNA expression levels are aberrant in cancer patients. Potentially, miRNAs originating from the tumor as freely circulating markers can be measured as well as miRNAs from different blood cell types^30,31^. In previous case control studies of different blood fractions we found not one single blood cell type that was able to explain the cancer profiles^32^. Especially in the context of broad studies describing profiles in multiple diseases^13^ it becomes clear that all the different factors have to be taken into account to explain the disease specific alterations of miRNA signatures.

Like breast cancer, TNBC is also a heterogeneous disease that encompasses several distinct entities with remarkably different molecular characteristics and clinical behaviour. In order to build a homogeneous study cohort of patients with TNBC, only patients with basal-like (CK5-positive) TNBC were included in the study. To determine whether deregulated miRNAs are associated with basal-like TNBC, a search was carried out in the “Integrated Breast Cancer Pathway” (miRPathDB), identifying experimental evidence of these miRNAs in other studies. Interestingly, van Schooneveld *et al*.^7^ described a large number of BC-related miRNAs that are involved in oncogenesis, metastasis, and resistance to various therapies. On the basis of these findings, the published miRNAs were grouped into lists that describe their potential role as diagnostic, prognostic, and predictive biomarkers^7^. When their lists were compared with the BC pathway–related miRNAs in the present study, it was found that 12 of 41 markers identified here matched their observations. Importantly, nine belonged to the list of “major diagnostic microRNAs for the early diagnosis of breast cancer” (hsa-miR-133b, hsa-miR-145-5p, hsa-miR-15a-5p, hsa-miR-16-5p, hsa-miR-181a-5p, hsa-miR-18a-5p, hsa-miR-21-5p, hsa-miR-365a-3p, and hsa-miR-92a-3p); one belonged to the list of “major oncogenic microRNAs in breast cancer” (hsa-miR-21-5p); one belonged to the list of “major tumour-suppressive microRNAs in breast cancer” (hsa-miR-126-5p); and one belonged to the list of “negative prognostic microRNA signatures in breast cancer” (hsa-miR-21-5p). Serum miR-21 has also previously been reported as a presumed independent poorly prognostic factor in patients with BC^33^.

In terms of predicting the response we were not successful in differentiating the pCR positive from the pCR negative group of patients based on the samples taken prior to the NCT. However, we found that changes over time have a potential to predict pCR. Besides pCR, other measures, especially for long-term metastatic free survival are important. We excluded this consideration in the present work for two reasons: 1) for the present cohort long-time follow-up is available only for a fraction of patients. This makes statistically reliable conclusions impossible. 2) Further, we found already comparably weak signals in predicting pCR. This makes it unlikely that our diagnostic signatures are suited to predict long-term outcome.

However, the findings need to be interpreted in the light of several limitations. Firstly, the study was conducted at a single site, so that hospital-level variations and potential selection bias are not taken into account. Secondly, only patients with a special subtype of TNBC were included, in order to achieve a homogeneous study cohort who were treated identically with platinum-based NCT. For reliable conclusions to be drawn regarding the diagnostic and prognostic value of blood-based miRNAs in all patients with breast cancer, large prospective studies including various subtypes of breast cancer are indispensable.

In conclusion, this study shows that neoadjuvant chemotherapy has a very substantial influence on miRNA patterns in patients with basal-like TNBC. Using miRNA profiles to predict the response to neoadjuvant chemotherapy is challenging, but it might possibly be achieved by analysing changes in the miRNA profile during neoadjuvant chemotherapy.

## Electronic supplementary material

Supplemental Figures

Supplemental Table 1

Supplemental Table 2

## References

[1] Torre LA (2015). Global cancer statistics, 2012. CA Cancer J Clin.

[2] Serpico D, Molino L, Di Cosimo S (2014). microRNAs in breast cancer development and treatment. Cancer Treat Rev.

[3] Fitzgibbons PL (2014). Template for reporting results of biomarker testing of specimens from patients with carcinoma of the breast. Arch Pathol Lab Med.

[4] Hammond ME (2010). American Society of Clinical Oncology/College of American Pathologists guideline recommendations for immunohistochemical testing of estrogen and progesterone receptors in breast cancer (unabridged version). Arch Pathol Lab Med.

[5] Shao Z, Chaudhri S, Guo M, Zhang L, Rea D (2016). Neoadjuvant Chemotherapy in Triple Negative Breast Cancer: An Observational Study. Oncol Res.

[6] von Minckwitz G (2011). Impact of treatment characteristics on response of different breast cancer phenotypes: pooled analysis of the German neo-adjuvant chemotherapy trials. Breast Cancer Res Treat.

[7] van Schooneveld E (2015). Dysregulation of microRNAs in breast cancer and their potential role as prognostic and predictive biomarkers in patient management. Breast Cancer Res.

[8] Inns J, James V (2015). Circulating microRNAs for the prediction of metastasis in breast cancer patients diagnosed with early stage disease. Breast.

[9] Kleivi Sahlberg K (2015). A serum microRNA signature predicts tumor relapse and survival in triple-negative breast cancer patients. Clin Cancer Res.

[10] Han JG (2017). A novel panel of serum miR-21/miR-155/miR-365 as a potential diagnostic biomarker for breast cancer. Ann Surg Treat Res.

[11] Zhu W, Qin W, Atasoy U, Sauter ER (2009). Circulating microRNAs in breast cancer and healthy subjects. BMC Res Notes.

[12] Keller A (2014). miRNAs can be generally associated with human pathologies as exemplified for miR-144. BMC Med.

[13] Keller A (2011). Toward the blood-borne miRNome of human diseases. Nat Methods.

[14] Schrauder MG (2012). Circulating micro-RNAs as potential blood-based markers for early stage breast cancer detection. PLoS One.

[15] Leidinger P, Backes C, Rheinheimer S, Keller A, Meese E (2015). Towards Clinical Applications of Blood-Borne miRNA Signatures: The Influence of the Anticoagulant EDTA on miRNA Abundance. PLoS One.

[16] Zhang H (2014). Biomarkers for monitoring pre-analytical quality variation of mRNA in blood samples. PLoS One.

[17] Backes C (2017). miRPathDB: a new dictionary on microRNAs and target pathways. Nucleic Acids Res.

[18] Hart M (2016). Identification of miR-34a-target interactions by a combined network based and experimental approach. Oncotarget.

[19] Werner TV (2017). MiR-34a-3p alters proliferation and apoptosis of meningioma cells *in vitro* and is directly targeting SMAD4, FRAT1 and BCL2. Aging (Albany NY).

[20] Backes C, Meese E, Lenhof HP, Keller A (2010). A dictionary on microRNAs and their putative target pathways. Nucleic Acids Res.

[21] Fehlmann T, Ludwig N, Backes C, Meese E, Keller A (2016). Distribution of microRNA biomarker candidates in solid tissues and body fluids. RNA Biol.

[22] Ludwig N (2016). Distribution of miRNA expression across human tissues. Nucleic Acids Res.

[23] Keller A (2015). Next-generation sequencing identifies altered whole blood microRNAs in neuromyelitis optica spectrum disorder which may permit discrimination from multiple sclerosis. J Neuroinflammation.

[24] Leidinger P, Keller A, Backes C, Huwer H, Meese E (2012). MicroRNA expression changes after lung cancer resection: a follow-up study. RNA Biol.

[25] Liu Y (2015). Tumor tissue microRNA expression in association with triple-negative breast cancer outcomes. Breast Cancer Res Treat.

[26] Wang D, Gu J, Wang T, Ding Z (2014). OncomiRDB: a database for the experimentally verified oncogenic and tumor-suppressive microRNAs. Bioinformatics.

[27] Backes C (2016). Bias in High-Throughput Analysis of miRNAs and Implications for Biomarker Studies. Anal Chem.

[28] Backes C (2016). Prioritizing and selecting likely novel miRNAs from NGS data. Nucleic Acids Res.

[29] Fehlmann T (2016). cPAS-based sequencing on the BGISEQ-500 to explore small non-coding RNAs. Clin Epigenetics.

[30] Leidinger P, Backes C, Meder B, Meese E, Keller A (2014). The human miRNA repertoire of different blood compounds. BMC Genomics.

[31] Schwarz EC (2016). Deep characterization of blood cell miRNomes by NGS. Cell Mol Life Sci.

[32] Leidinger P (2014). What makes a blood cell based miRNA expression pattern disease specific?–a miRNome analysis of blood cell subsets in lung cancer patients and healthy controls. Oncotarget.

[33] Wang G, Wang L, Sun S, Wu J, Wang Q (2015). Quantitative measurement of serum microRNA-21 expression in relation to breast cancer metastasis in Chinese females. Ann Lab Med.

