# Supplementary material for: MicroRNA in diagnosis and therapy monitoring of early-stage triple-negative breast cancer
Source: Sci Rep. 2018 Aug 2;8:11584. doi: 10.1038/s41598-018-29917-2 (PMC6072710; doi:10.1038/s41598-018-29917-2)
Supplement: Supplementary file 1 — Supplemental Figures [file 41598_2018_29917_MOESM1_ESM.pdf]

Supplemental Figures

MicroRNA in diagnosis and therapy monitoring of early-stage triple-negative breast cancer

Mustafa Kahraman[1,2,], Anne Röske[2], Thomas Laufer[2,], Tobias Fehlmann[1], Christina Backes[1], Fabian Kern[1], Jochen Kohlhaas[2], Hannah Schrörs[2], Anna Saiz[2], Cassandra Zabler[2], Nicole Ludwig[4], Peter A. Fasching[3], Reiner Strick[3], Matthias Rübner[3], Matthias W. Beckmann[3], Eckart Meese[4], Andreas Keller[1,&,], Michael G. Schrauder[3,]

[1] Clinical Bioinformatics, Saarland University, Homburg, Germany

[2] Hummingbird Diagnostics GmbH, Heidelberg, Germany

[3] Friedrich-Alexander University Erlangen-Nürnberg (FAU), Department of Obstetrics and Gynecology, Erlangen University Hospital, Comprehensive Cancer Center Erlangen-EMN, Erlangen, Germany

[4] Department of Human Genetics, Saarland University, Homburg, Germany

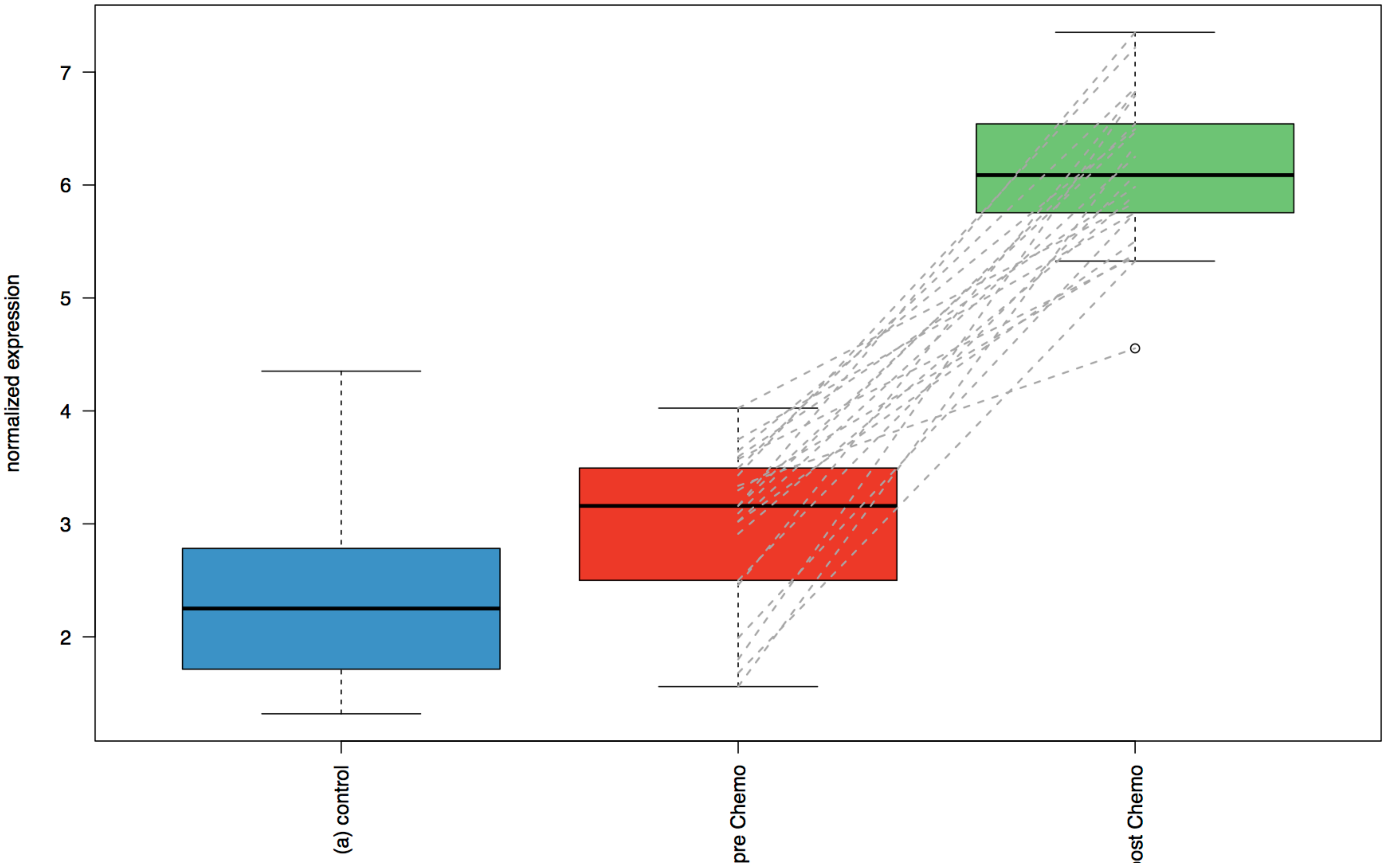
normalized expression
7
6
5
4
3
2
(a) control
pre Chemo
ost Chemo

Fig. S2A

Controls vs Pre-chemo

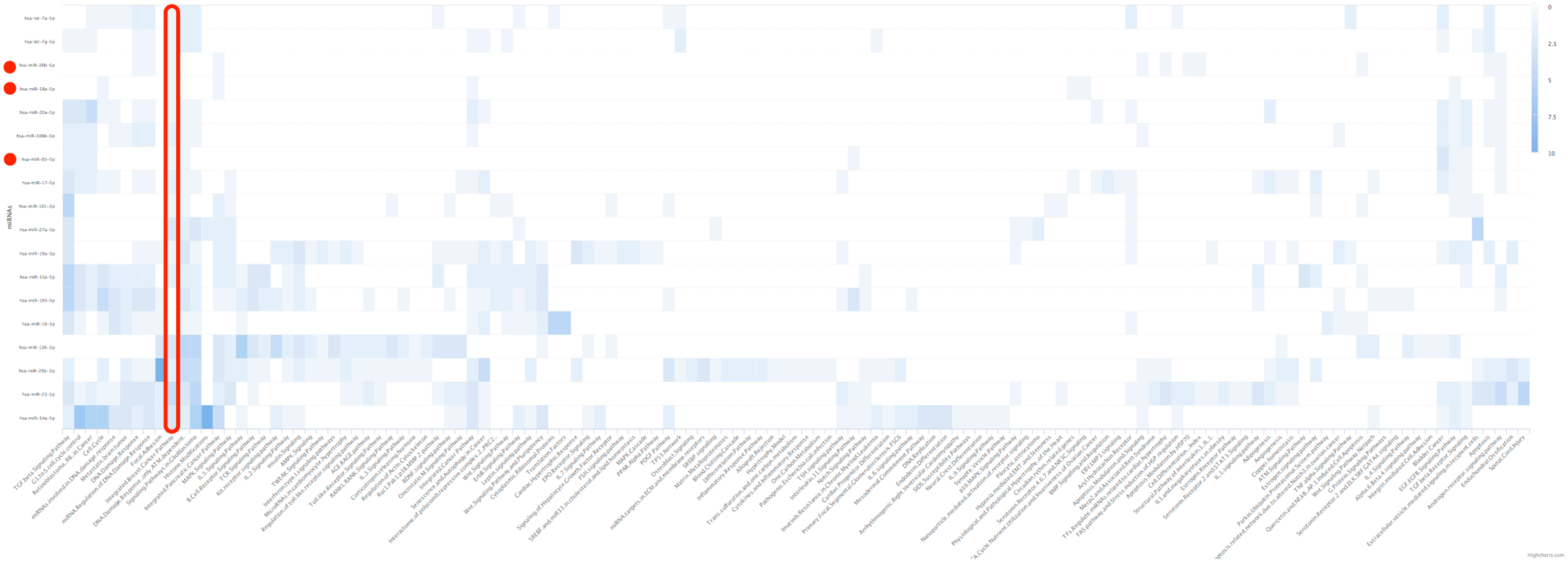

Fig. S2B

Controls vs Post-chemo

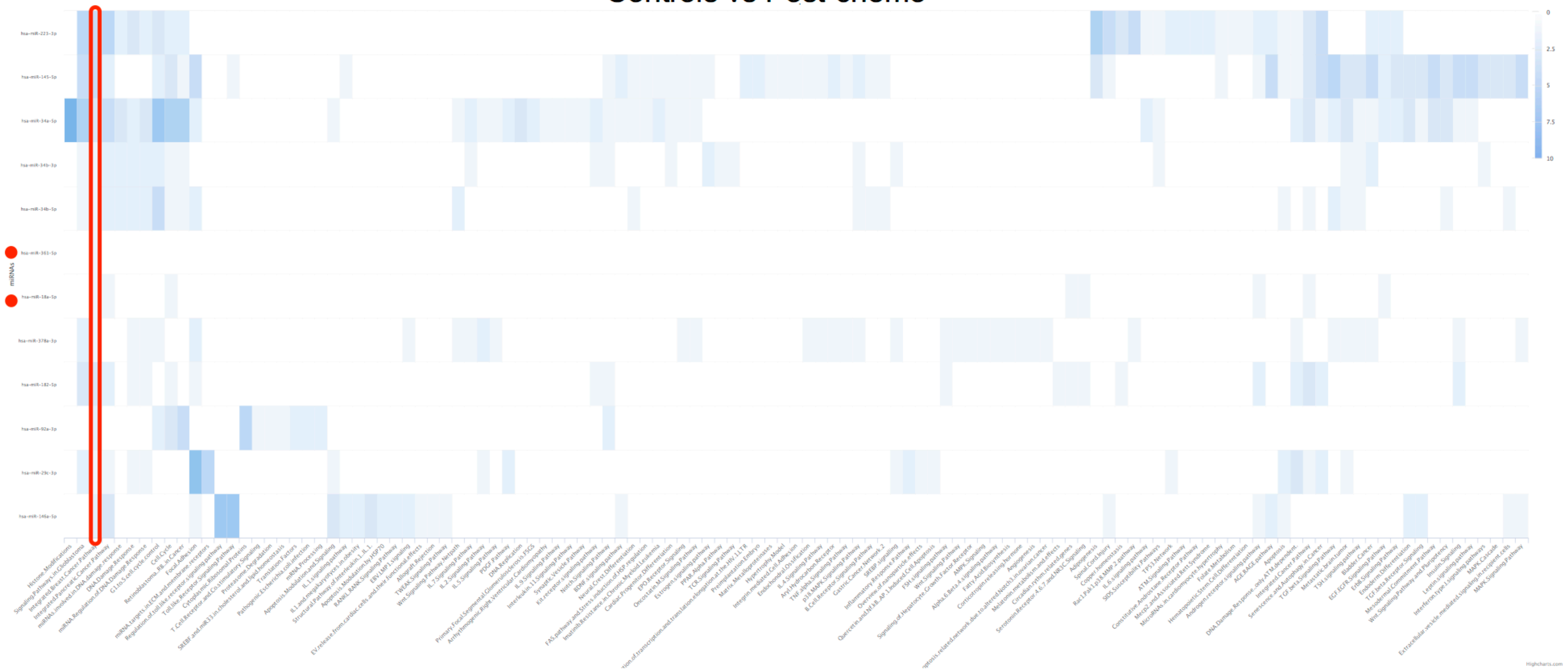

Fig. S2C

Pre-chemo vs Post-chemo

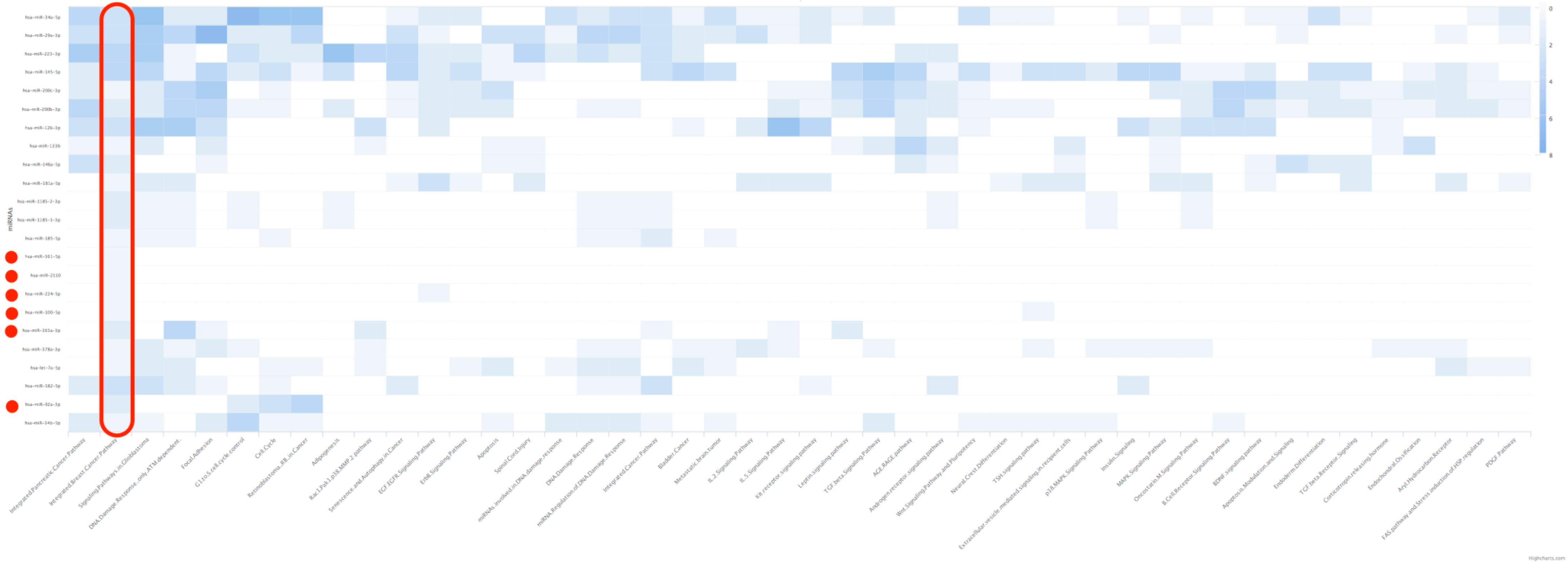

Fig. S2D

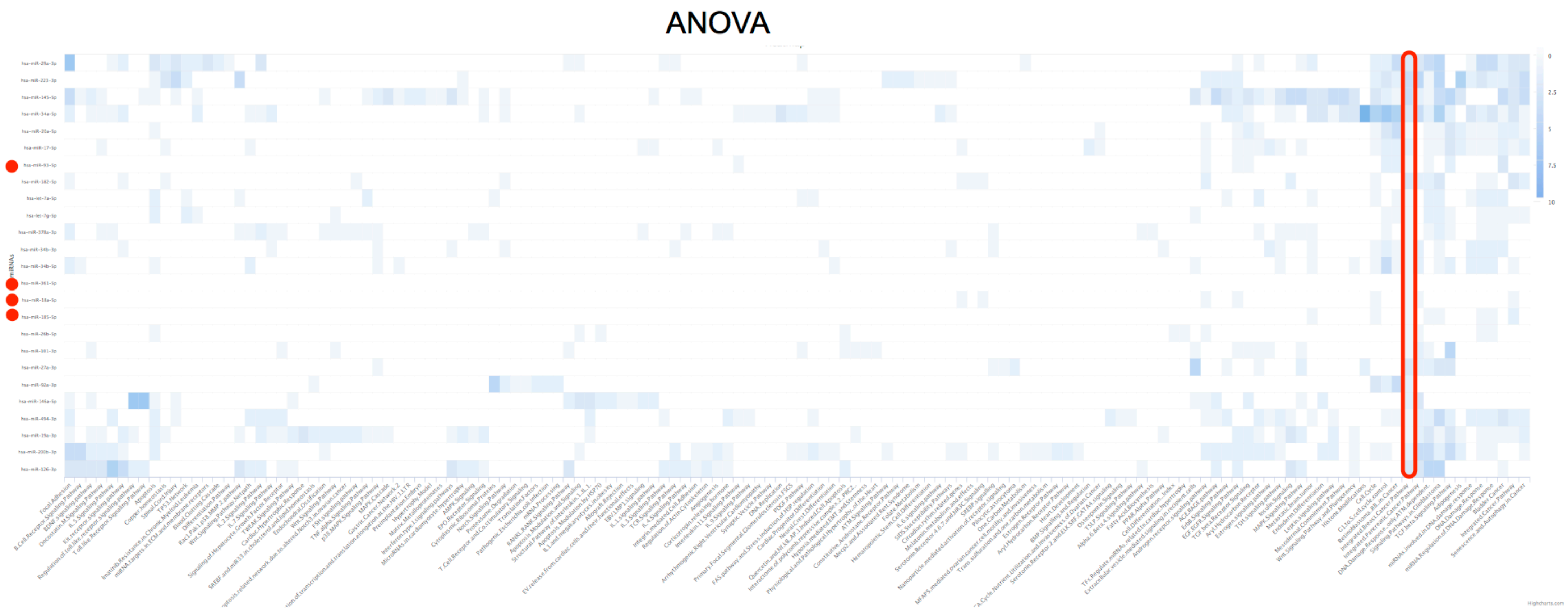

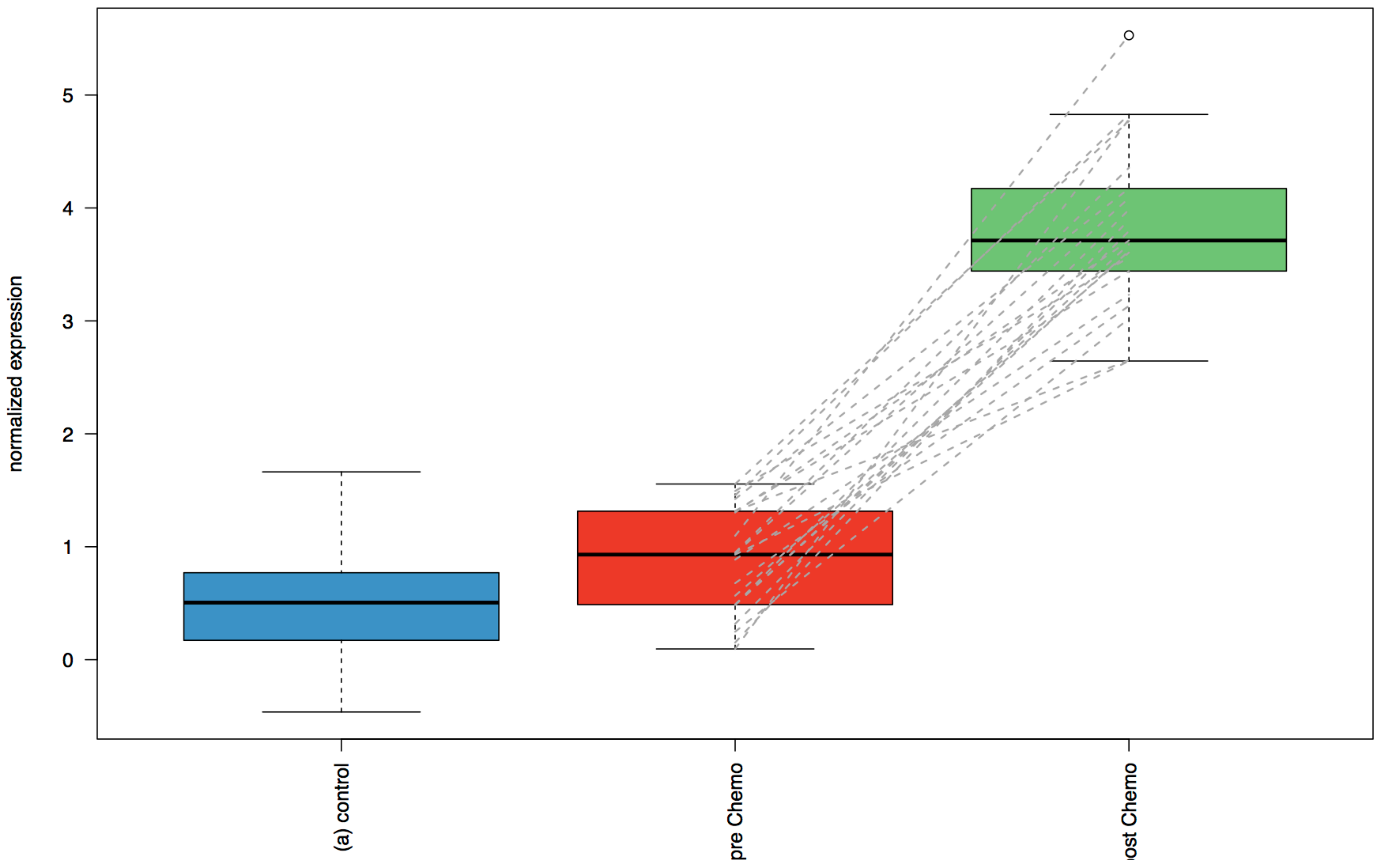
normalized expression
0
1
2
3
4
5
(a) control
pre Chemo
ost Chemo

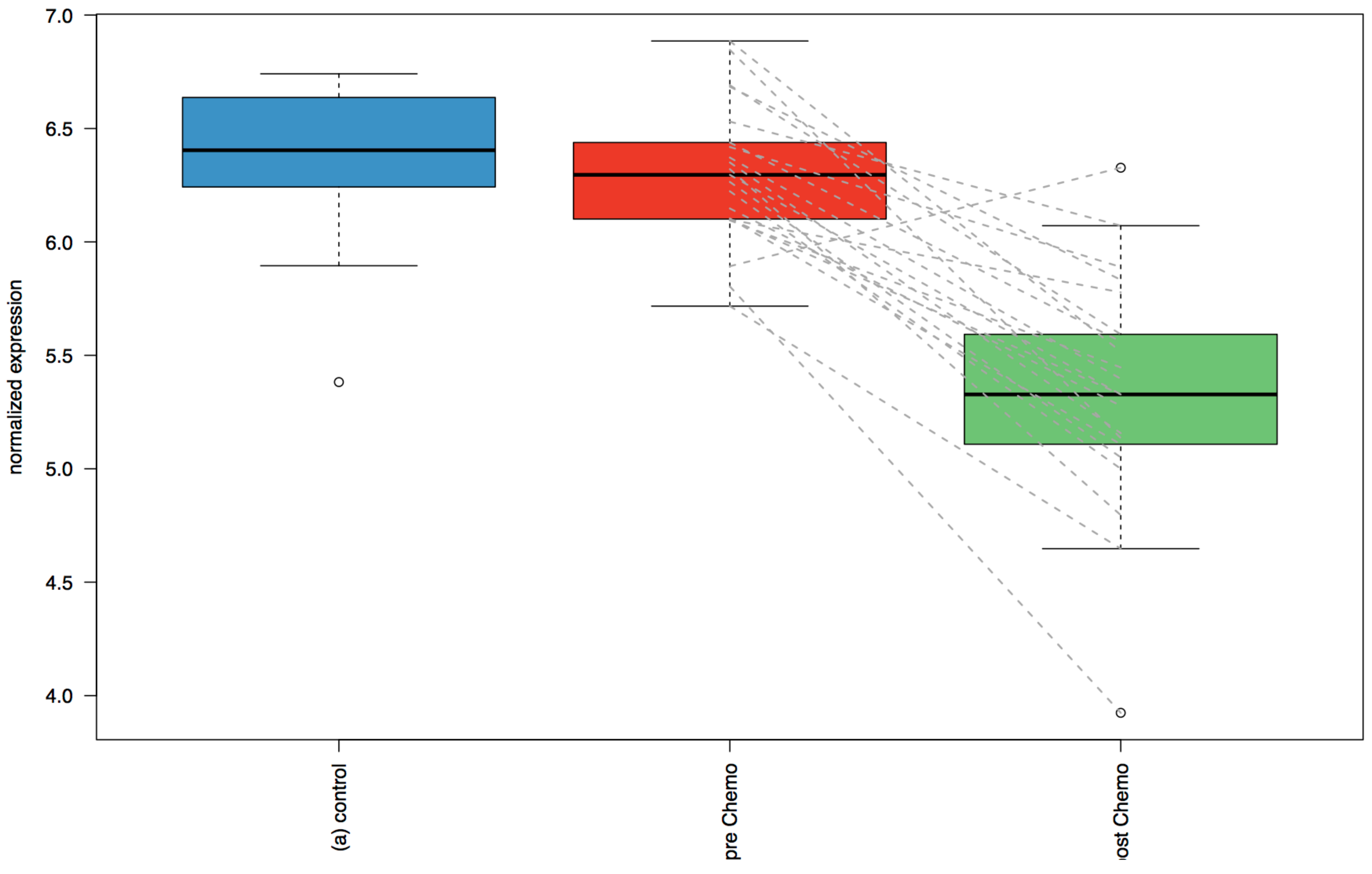
normalized expression
7.0
6.5
6.0
5.5
5.0
4.5
4.0
(a) control
pre Chemo
ost Chemo

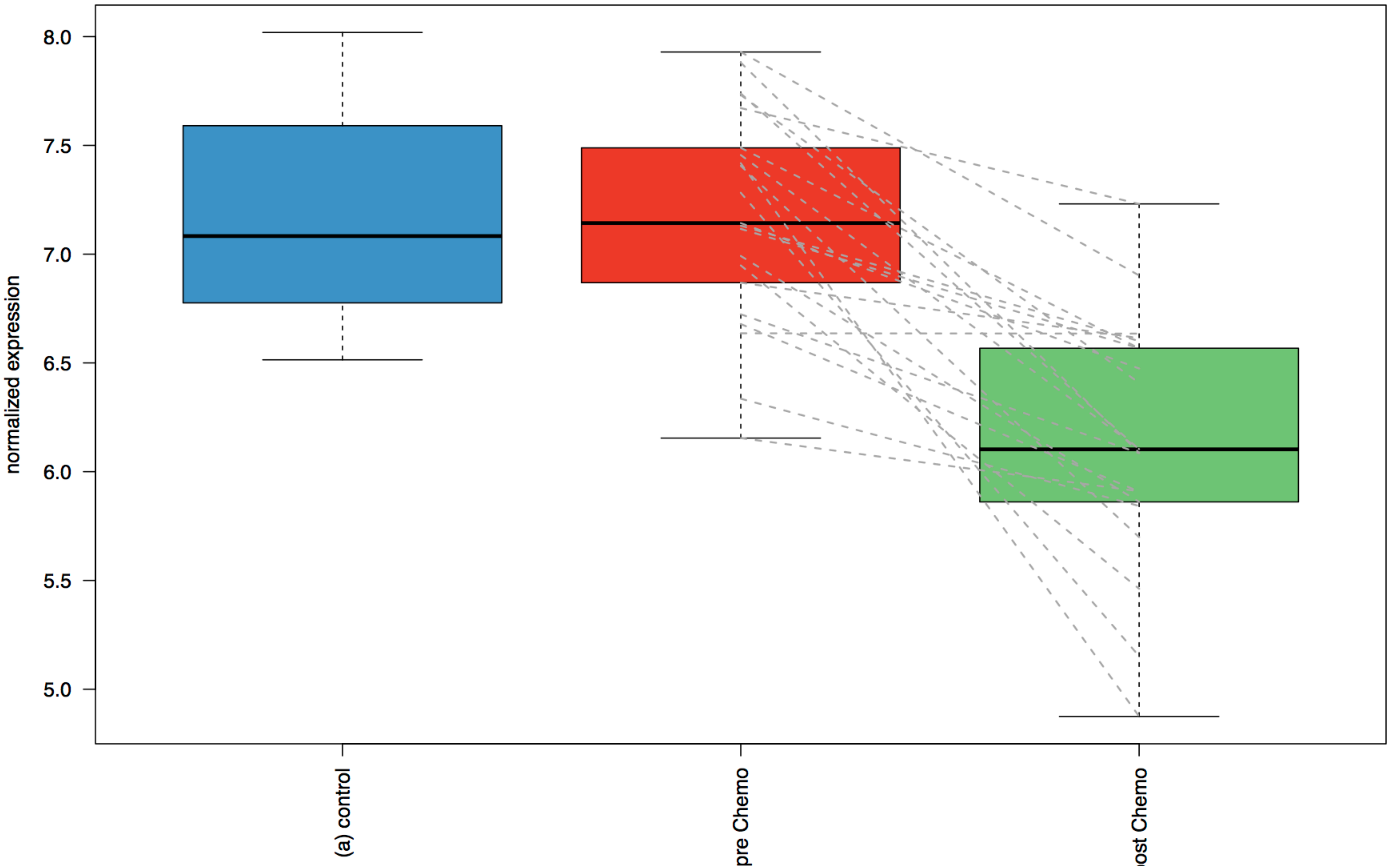
normalized expression
8.0
7.5
7.0
6.5
6.0
5.5
5.0
(a) control
pre Chemo
ost Chemo

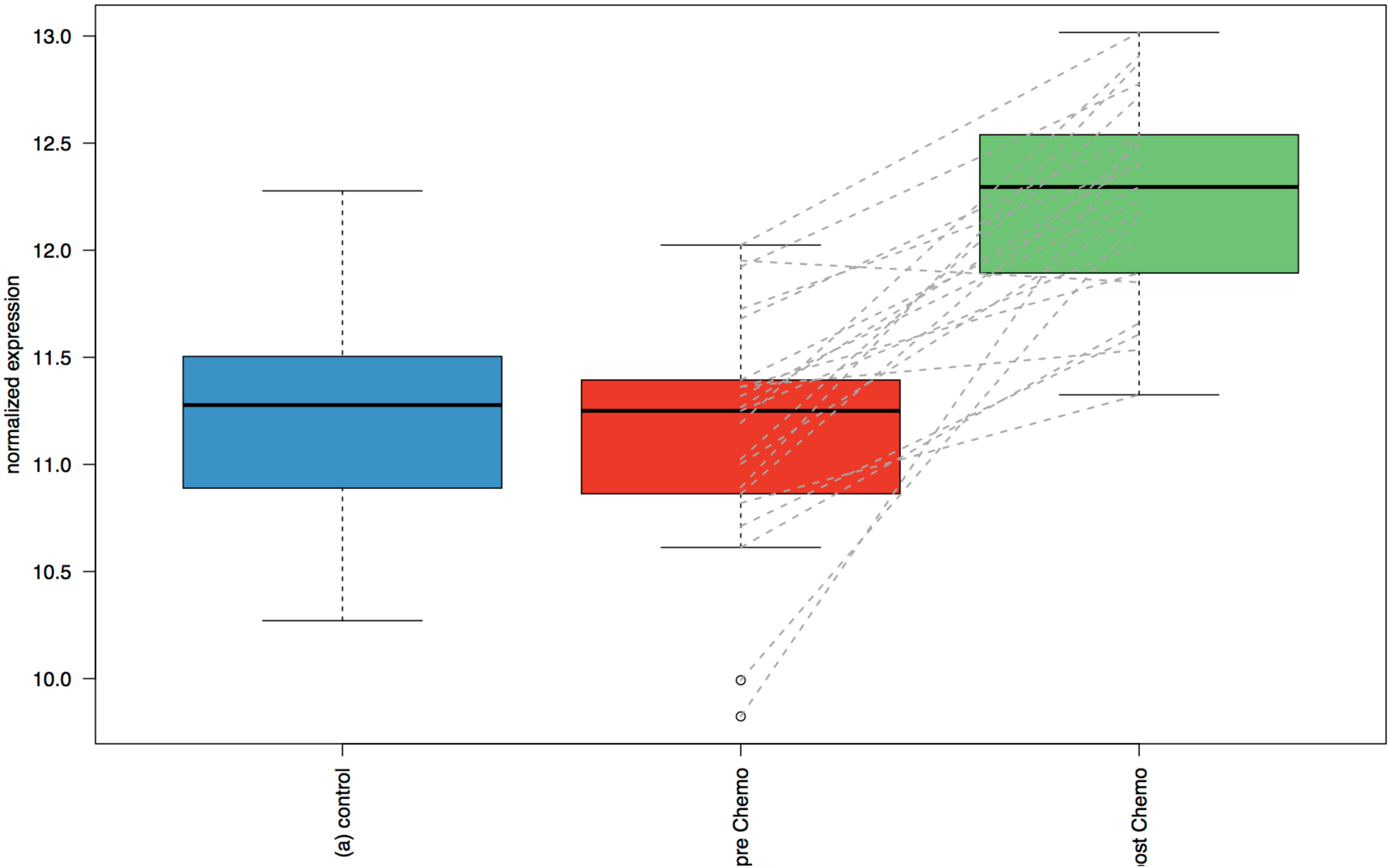
normalized expression
13.0
12.5
12.0
11.5
11.0
10.5
10.0
(a) control
pre Chemo
ost Chemo

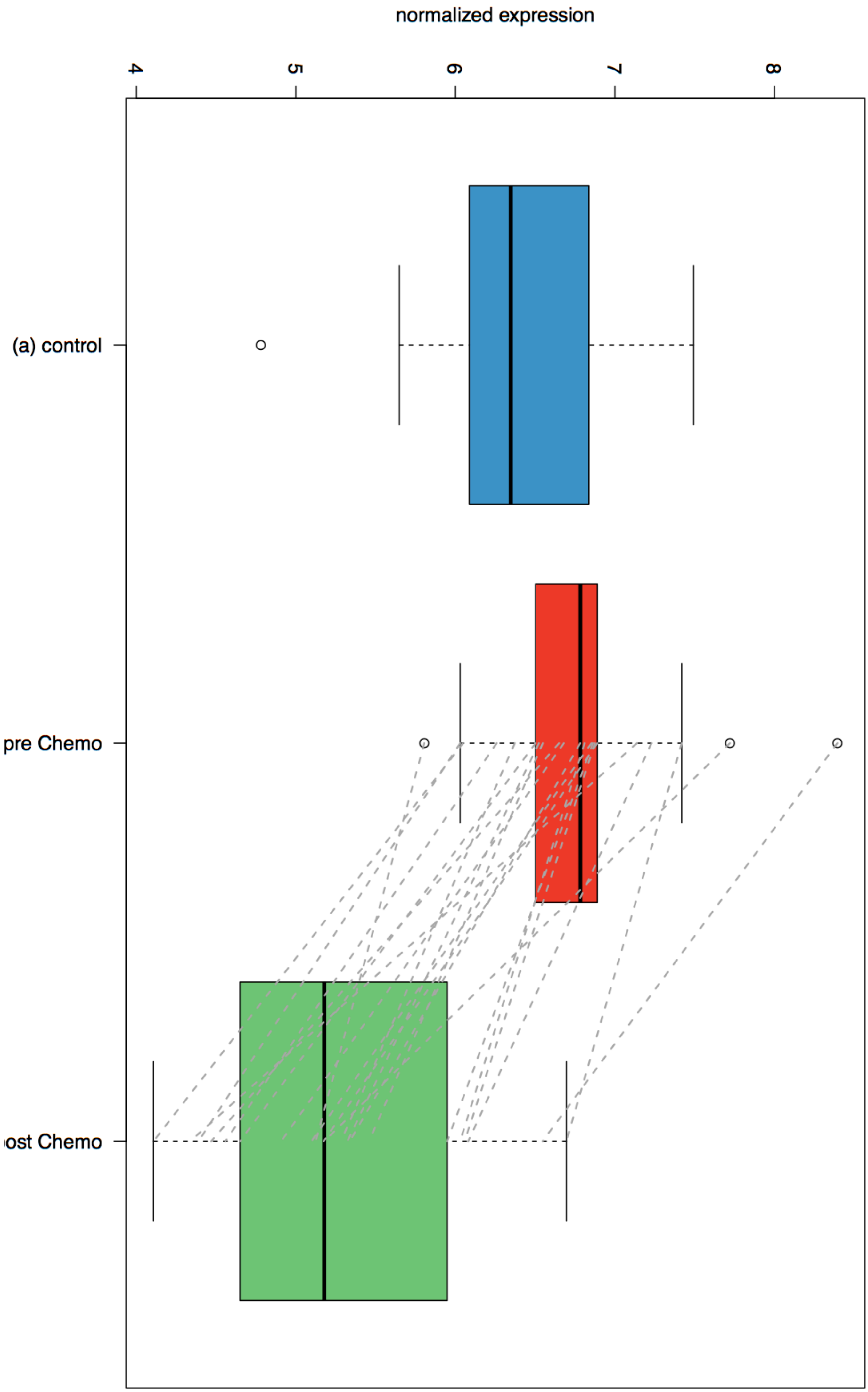
normalized expression
4
5
6
7
8
(a) control
pre Chemo
ost Chemo

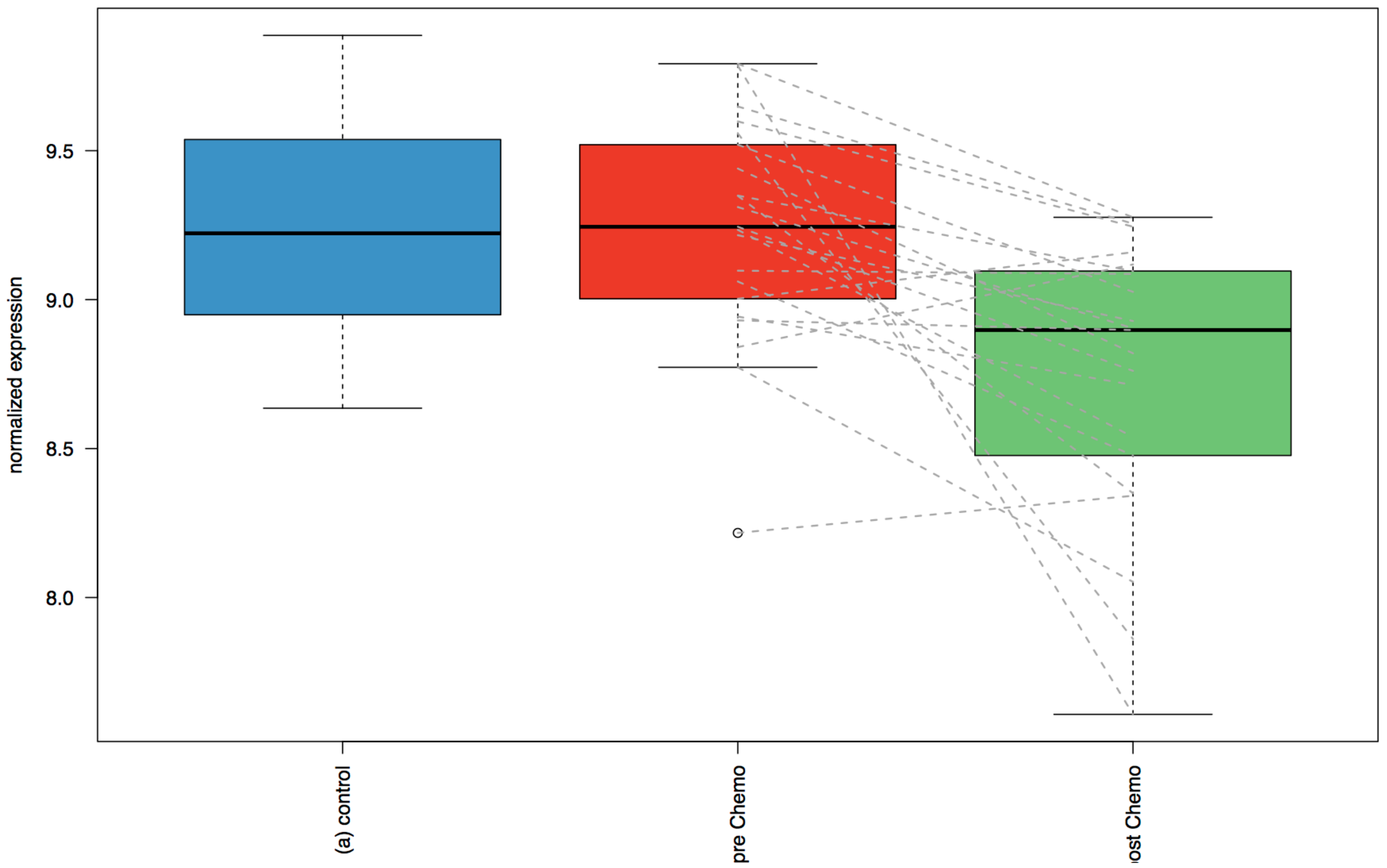
normalized expression
9.5
9.0
8.5
8.0
(a) control
pre Chemo
ost Chemo

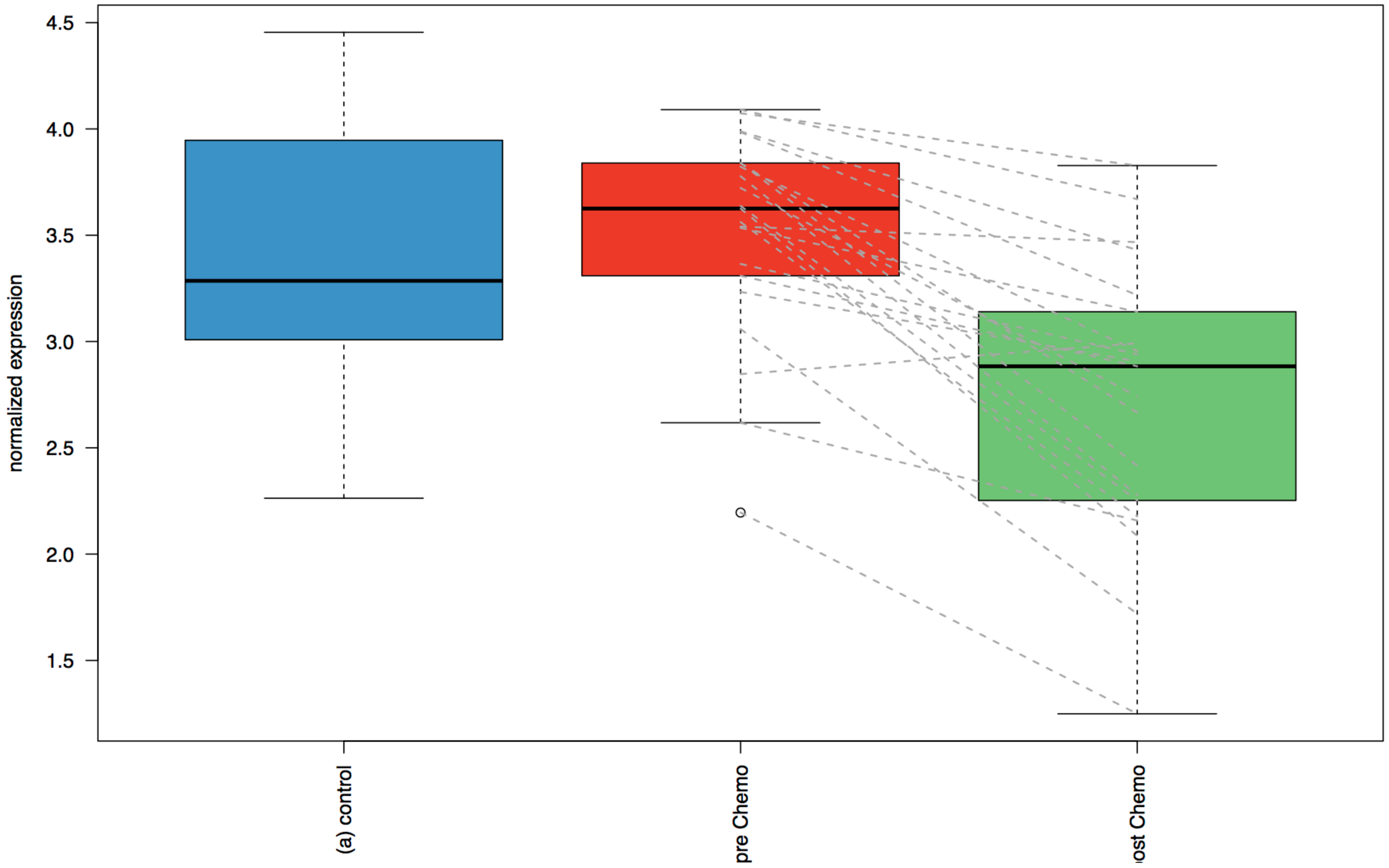
normalized expression
4.5
4.0
3.5
3.0
2.5
2.0
1.5
(a) control
pre Chemo
ost Chemo

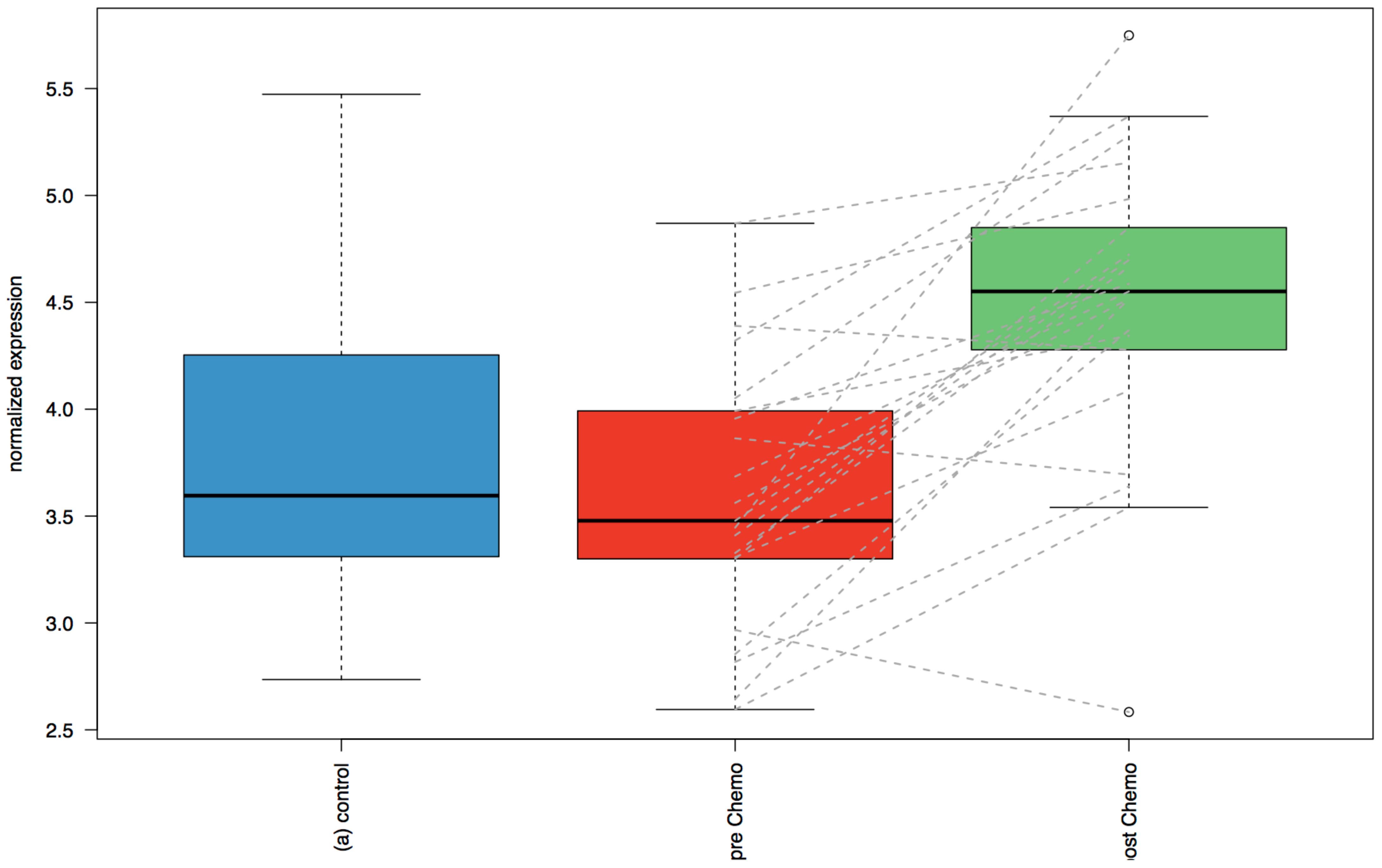
normalized expression
5.5
5.0
4.5
4.0
3.5
3.0
2.5
(a) control
pre Chemo
ost Chemo

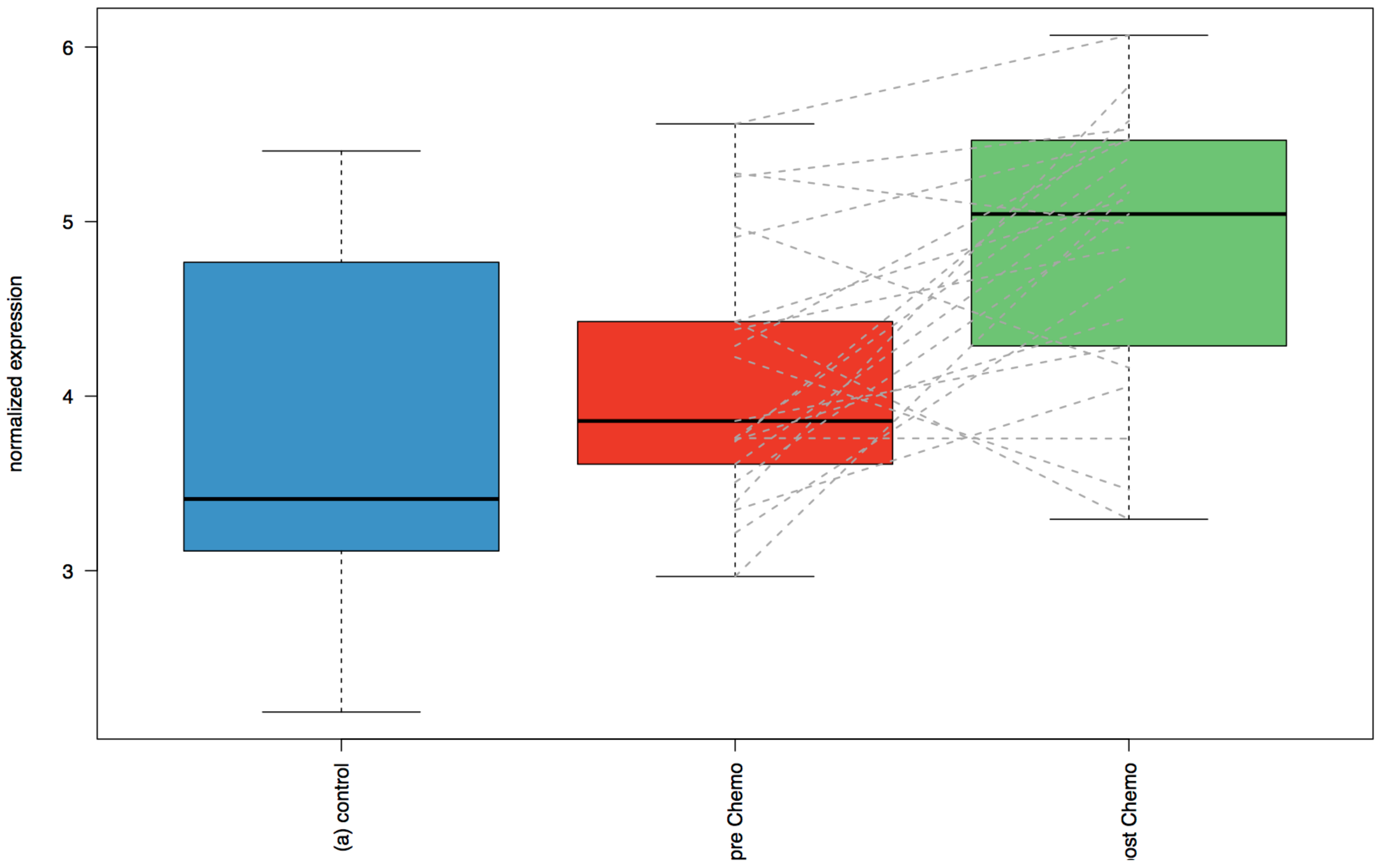
normalized expression
6
5
4
3
(a) control
pre Chemo
ost Chemo

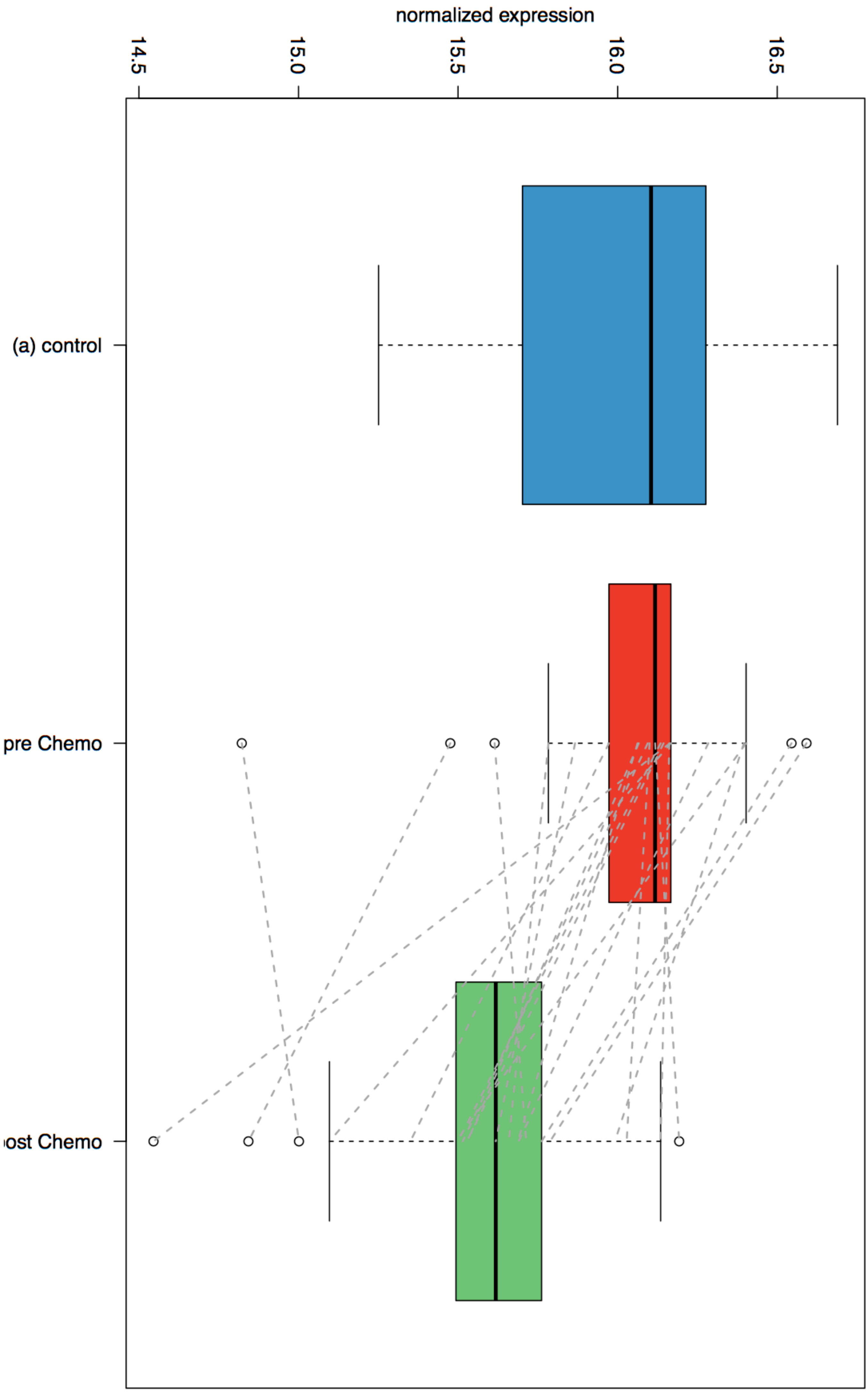
normalized expression
14.5
15.0
15.5
16.0
16.5
(a) control
pre Chemo
ost Chemo

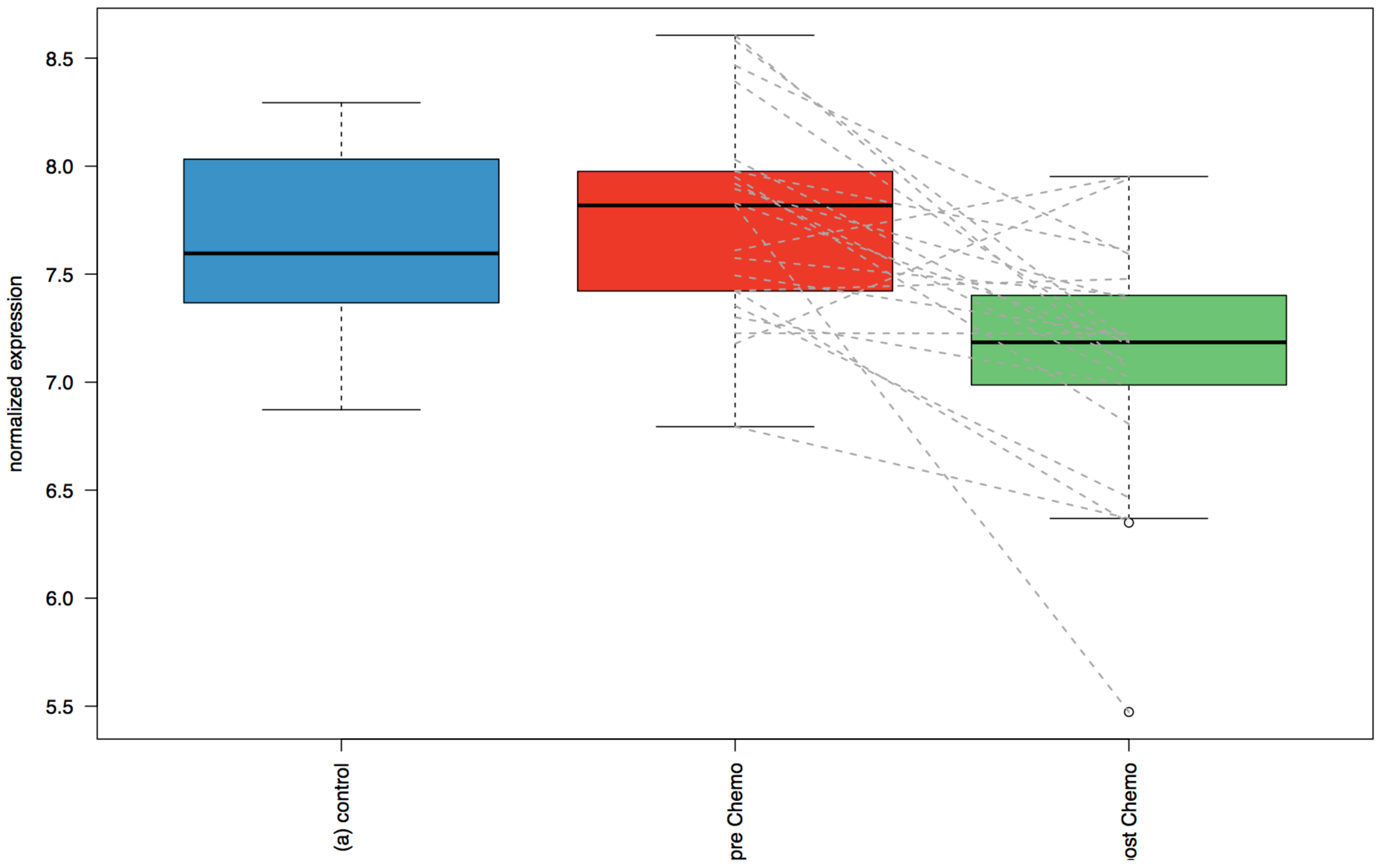
normalized expression
8.5
8.0
7.5
7.0
6.5
6.0
5.5
(a) control
pre Chemo
ost Chemo

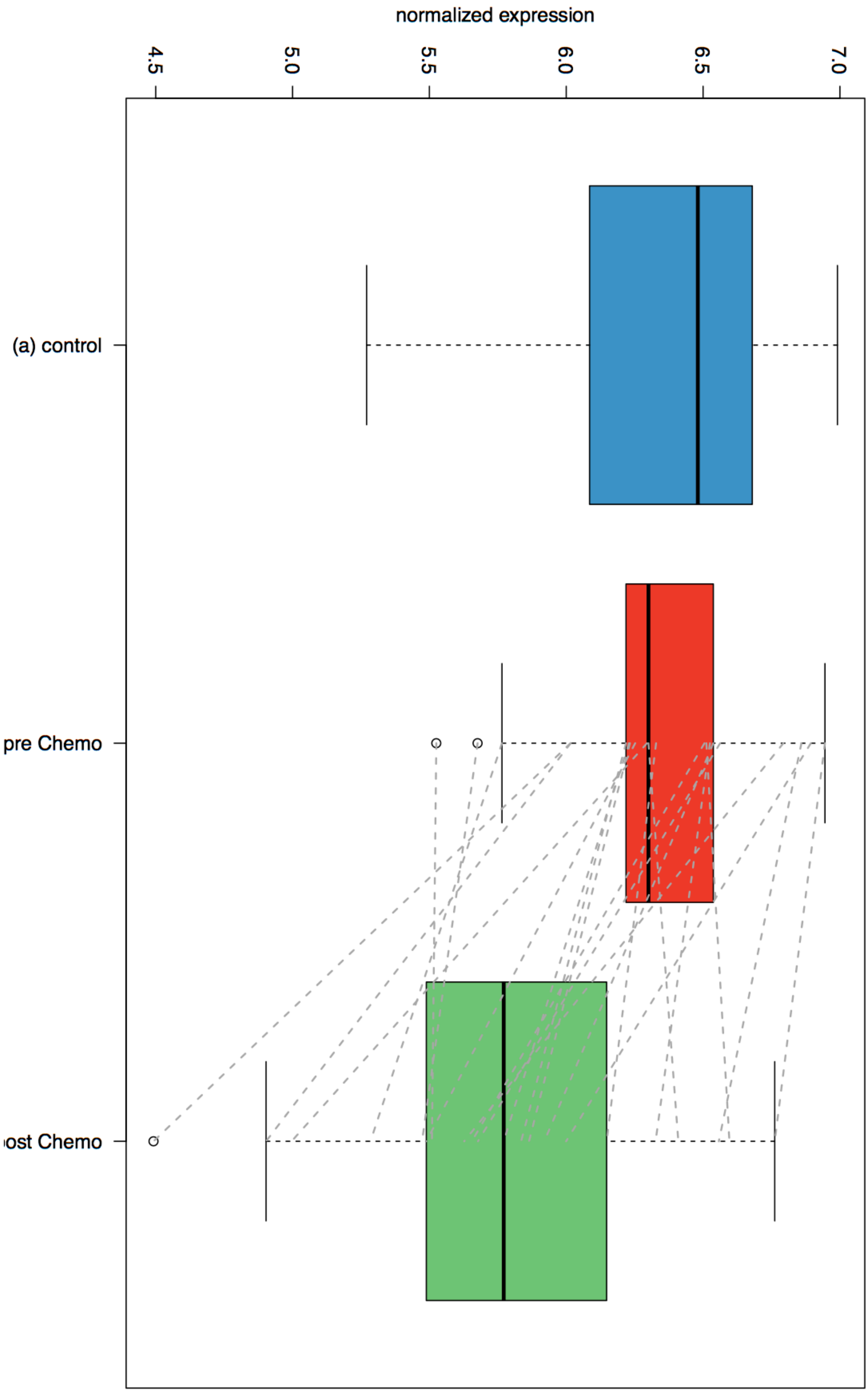
normalized expression
4.5
5.0
5.5
6.0
6.5
7.0
(a) control
pre Chemo
ost Chemo

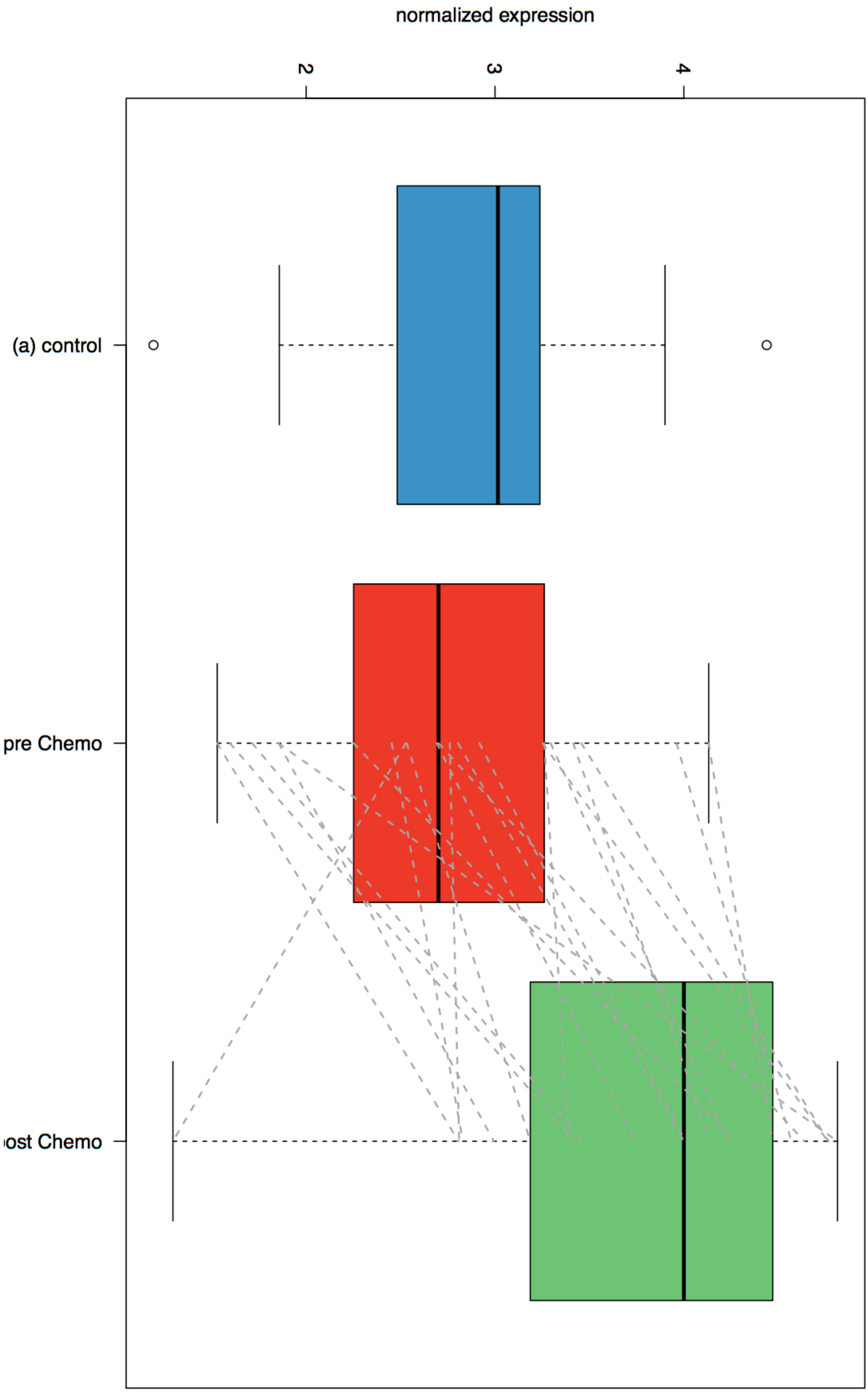
normalized expression
2
3
4
(a) control
pre Chemo
ost Chemo

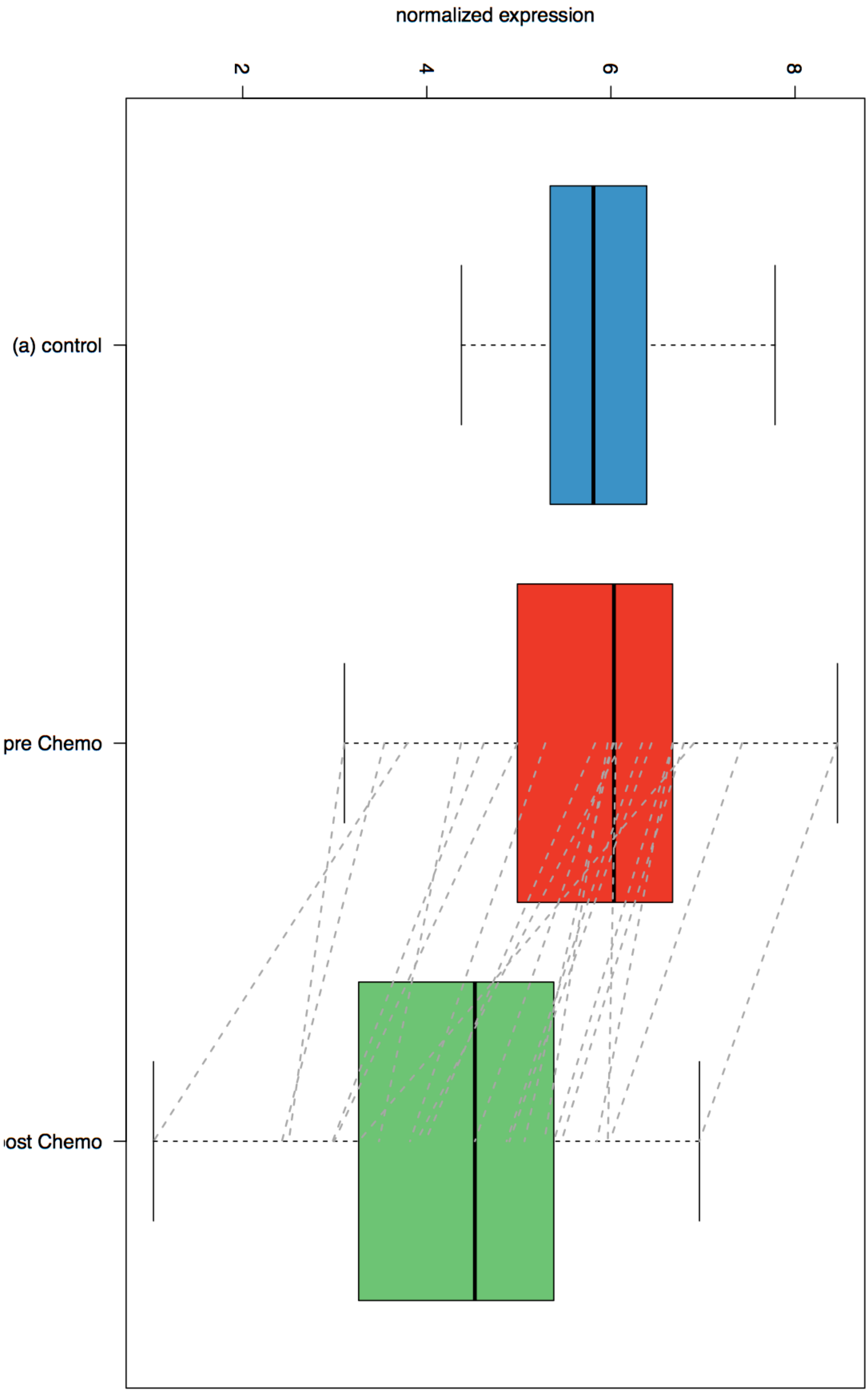
normalized expression
2
4
6
8
(a) control
pre Chemo
ost Chemo

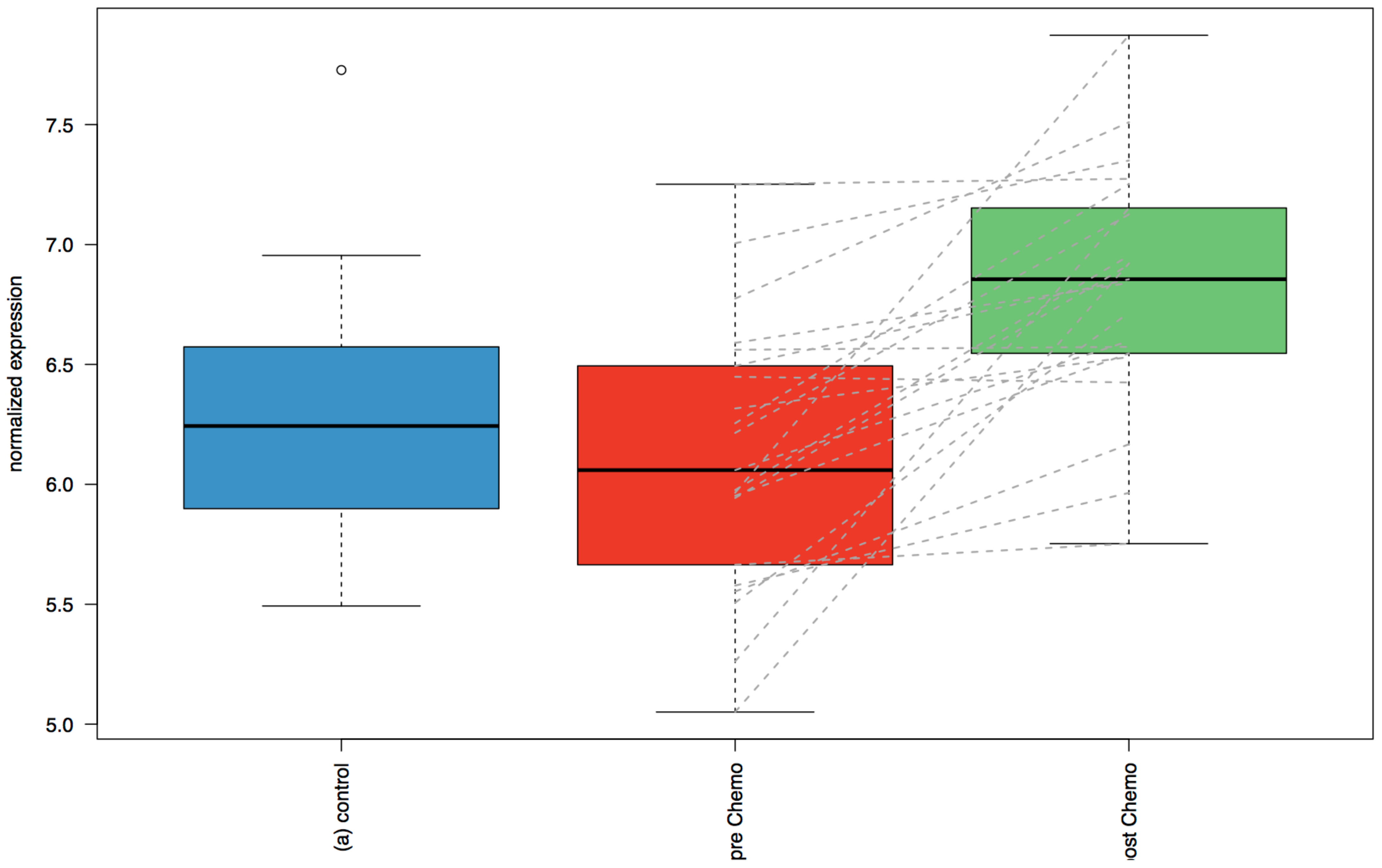
normalized expression
7.5
7.0
6.5
6.0
5.5
5.0
(a) control
pre Chemo
ost Chemo

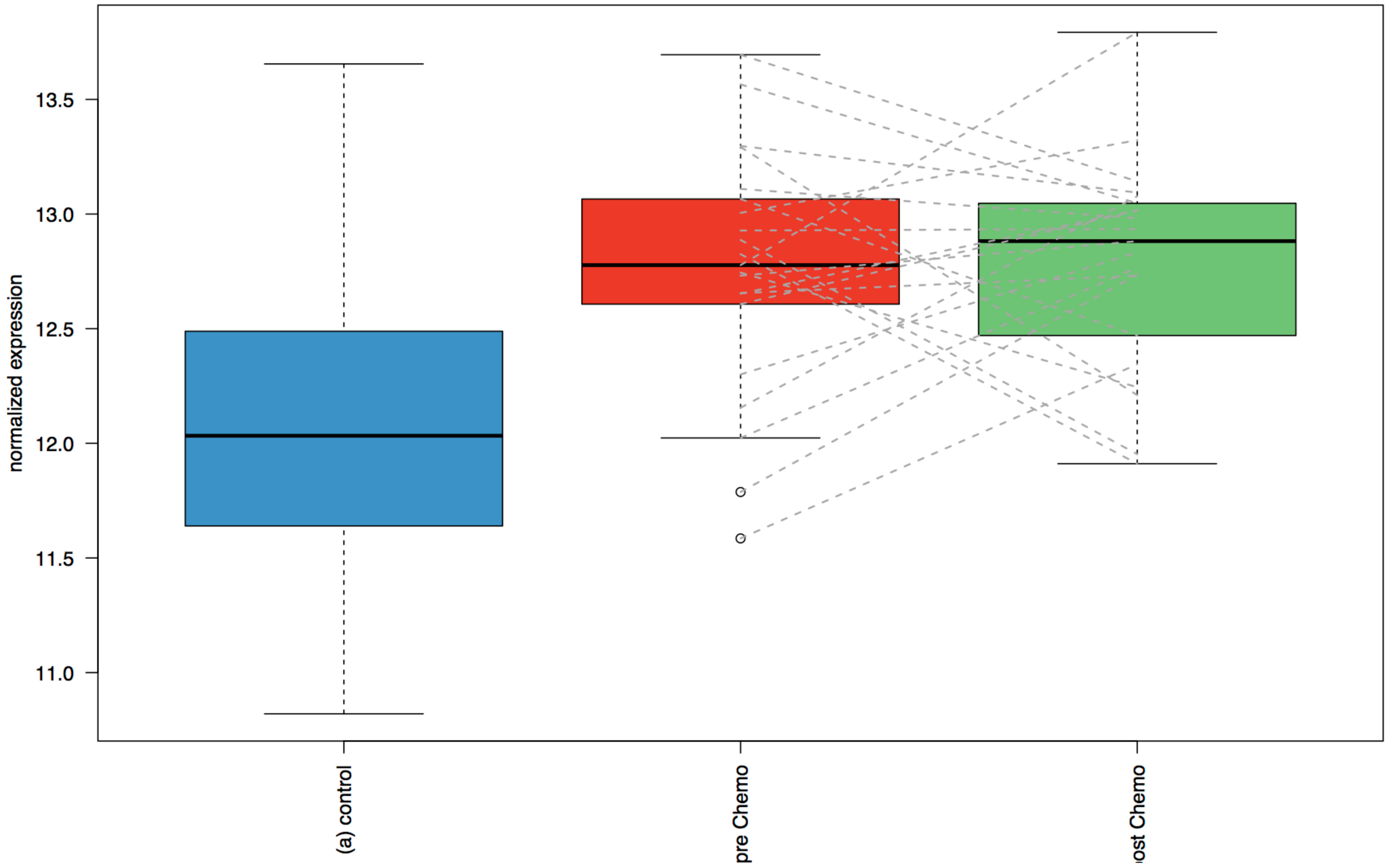
normalized expression
13.5
13.0
12.5
12.0
11.5
11.0
(a) control
pre Chemo
ost Chemo

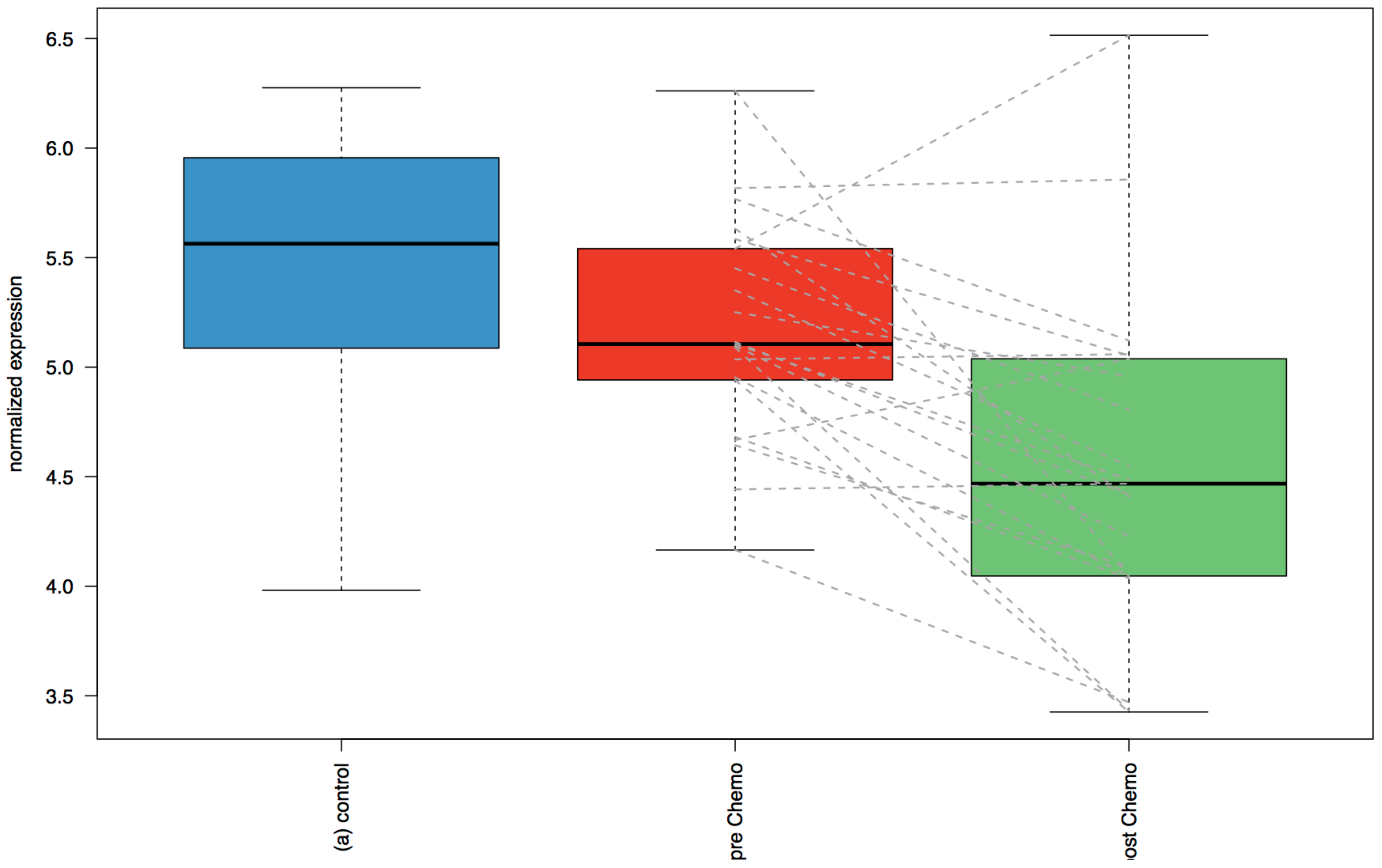
normalized expression
6.5
6.0
5.5
5.0
4.5
4.0
3.5
(a) control
pre Chemo
ost Chemo

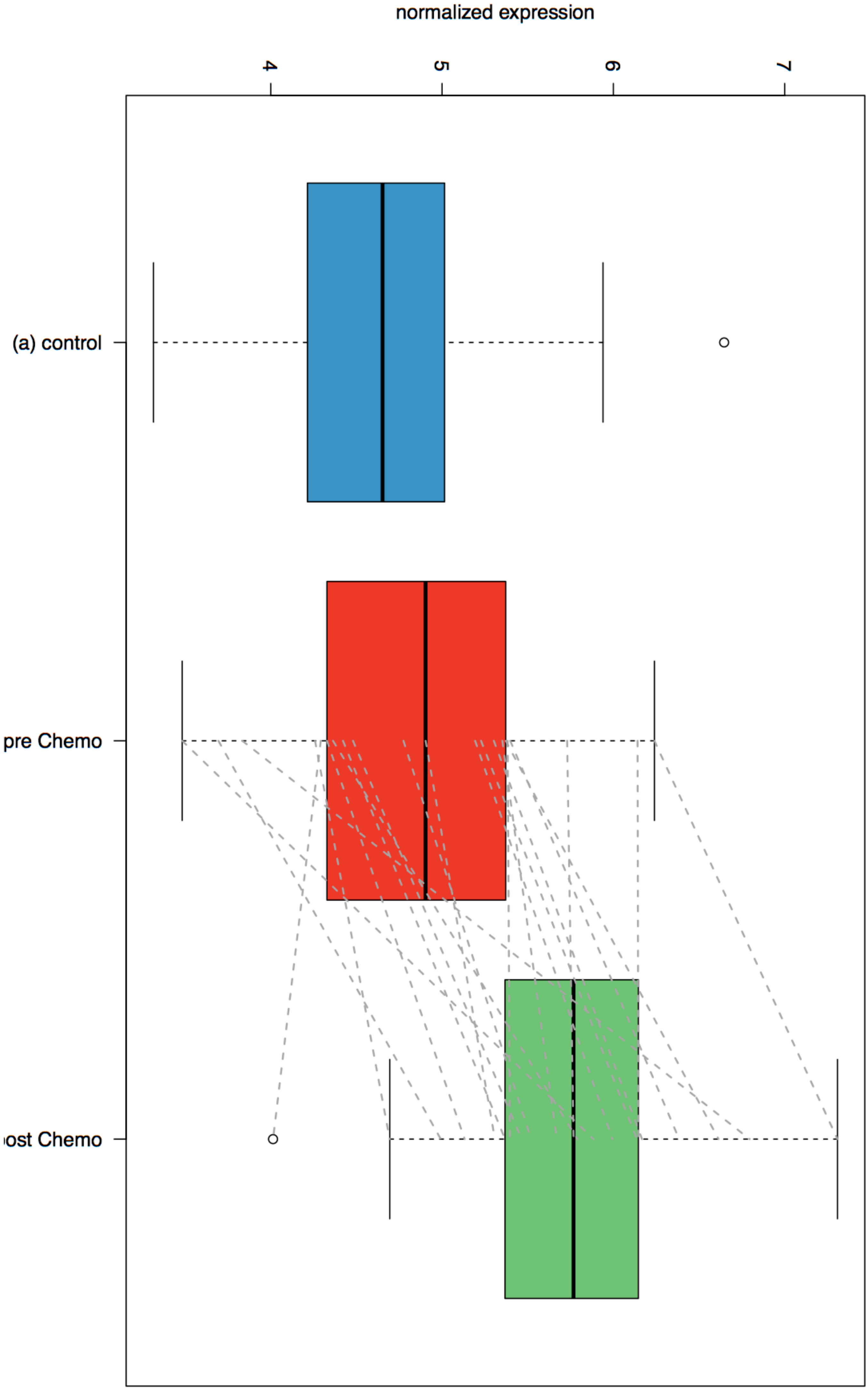

normalized expression
4
5
6
7
(a) control
pre Chemo
ost Chemo

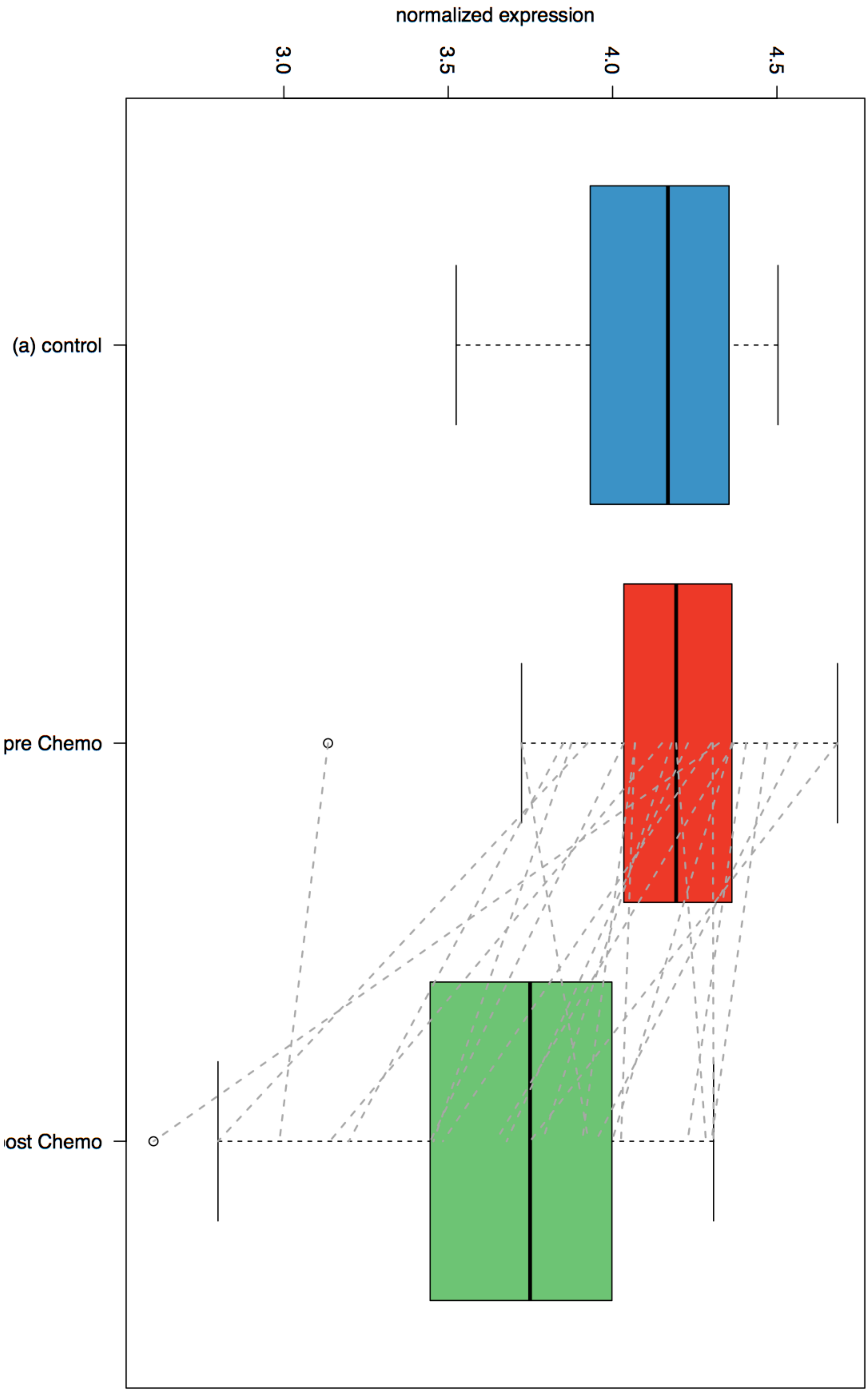

normalized expression
3.0
3.5
4.0
4.5
(a) control
pre Chemo
ost Chemo

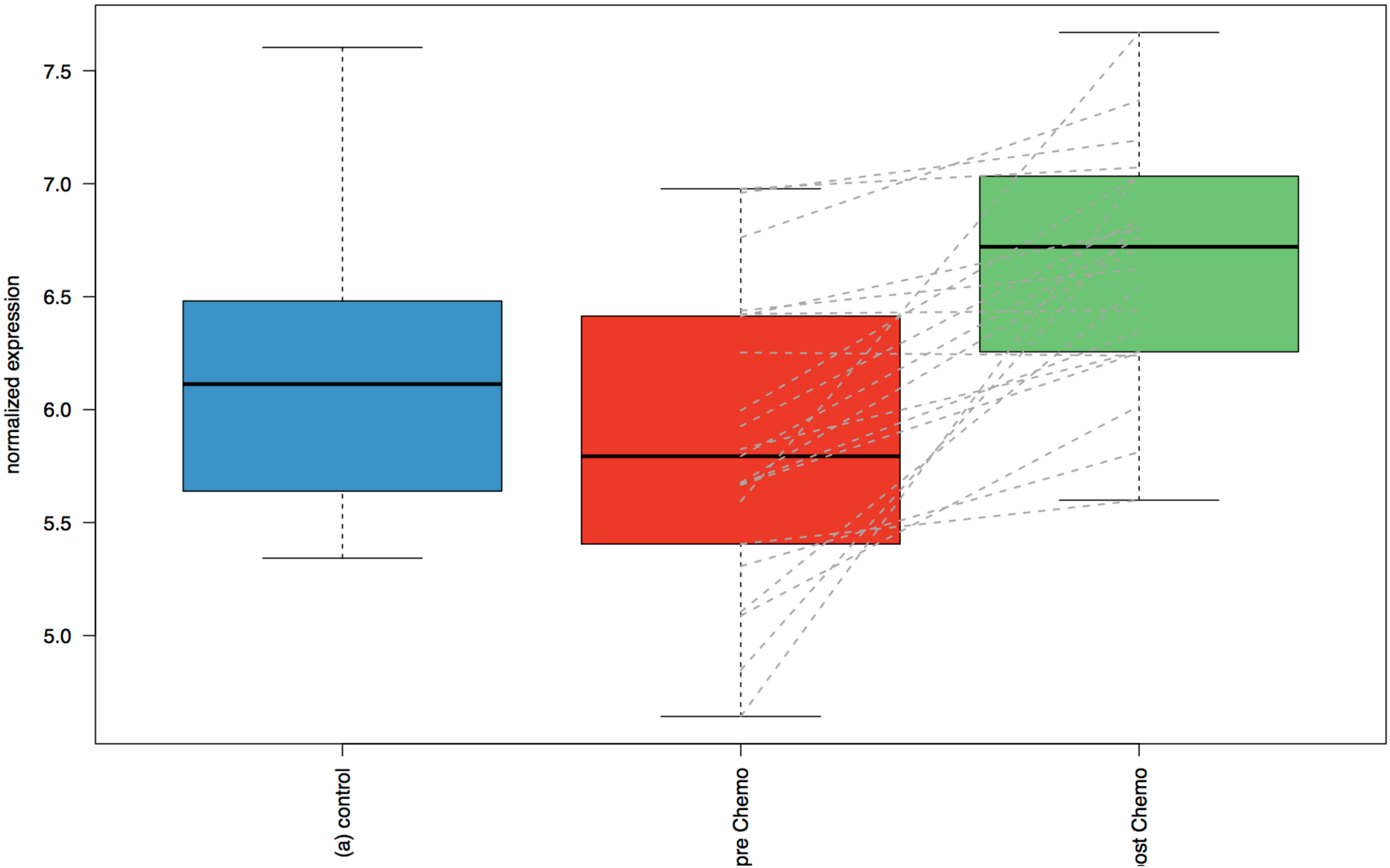
normalized expression
7.5
7.0
6.5
6.0
5.5
5.0
(a) control
pre Chemo
ost Chemo

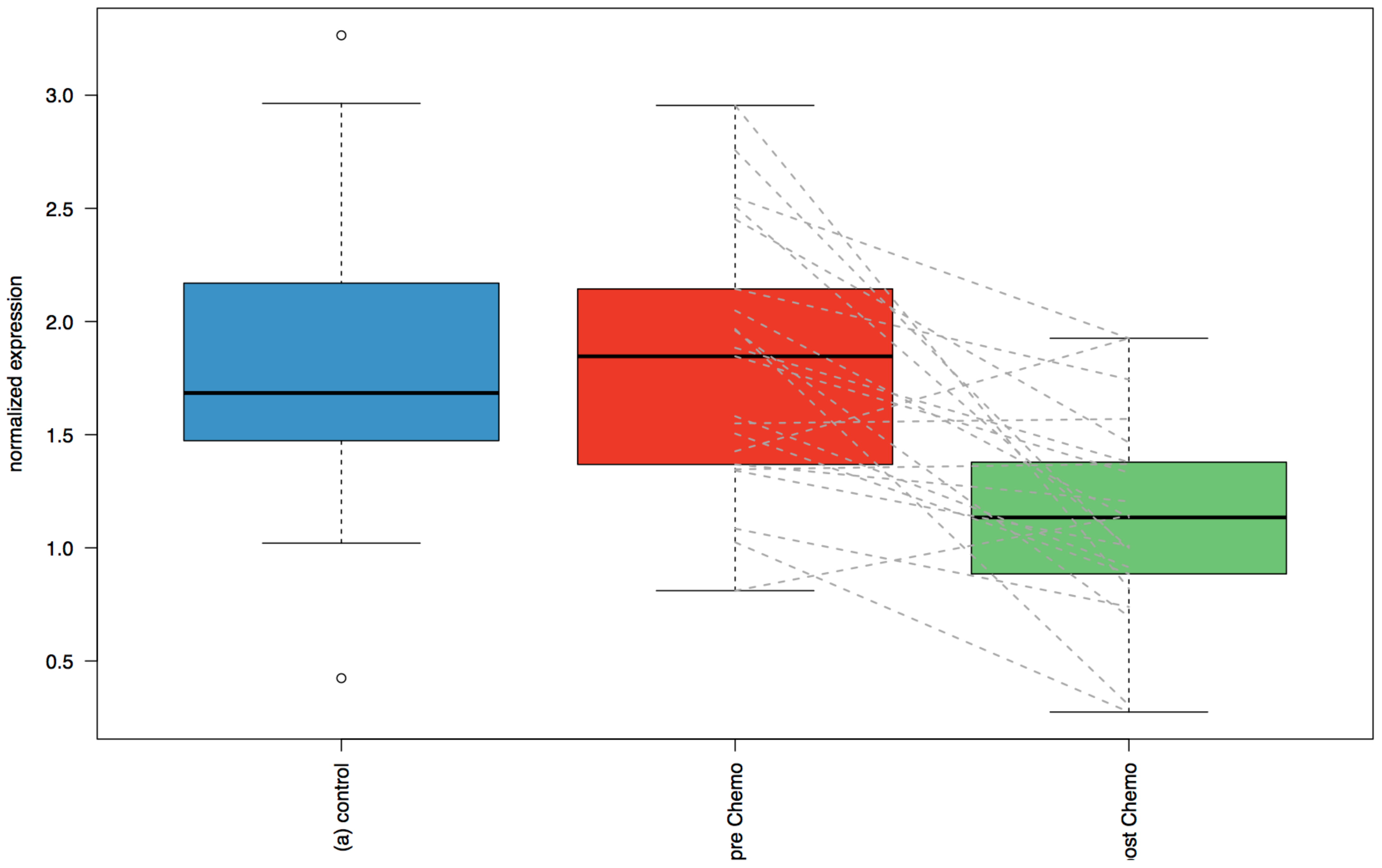
normalized expression
3.0
2.5
2.0
1.5
1.0
0.5
(a) control
pre Chemo
ost Chemo

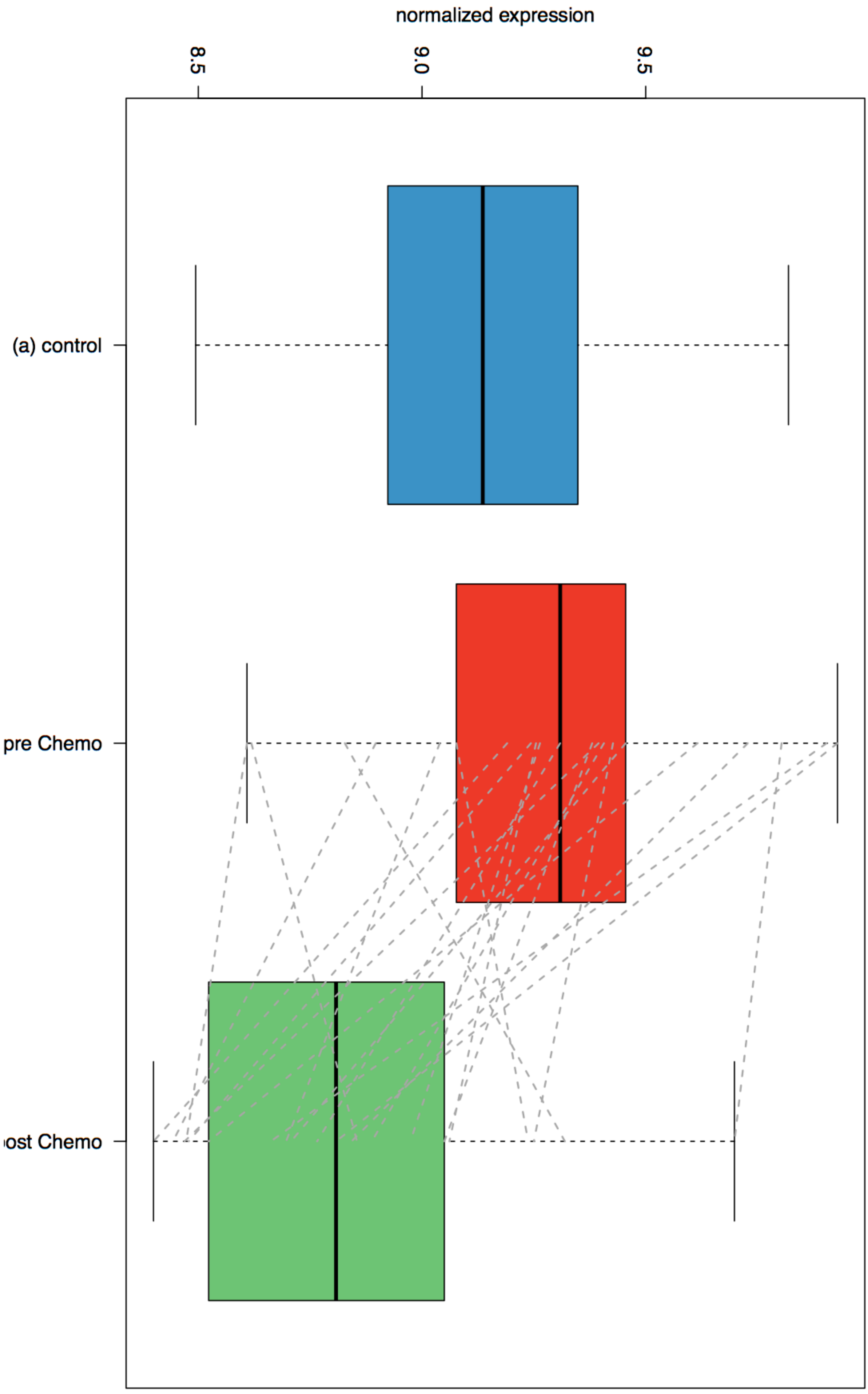
normalized expression
8.5
9.0
9.5
(a) control
pre Chemo
ost Chemo

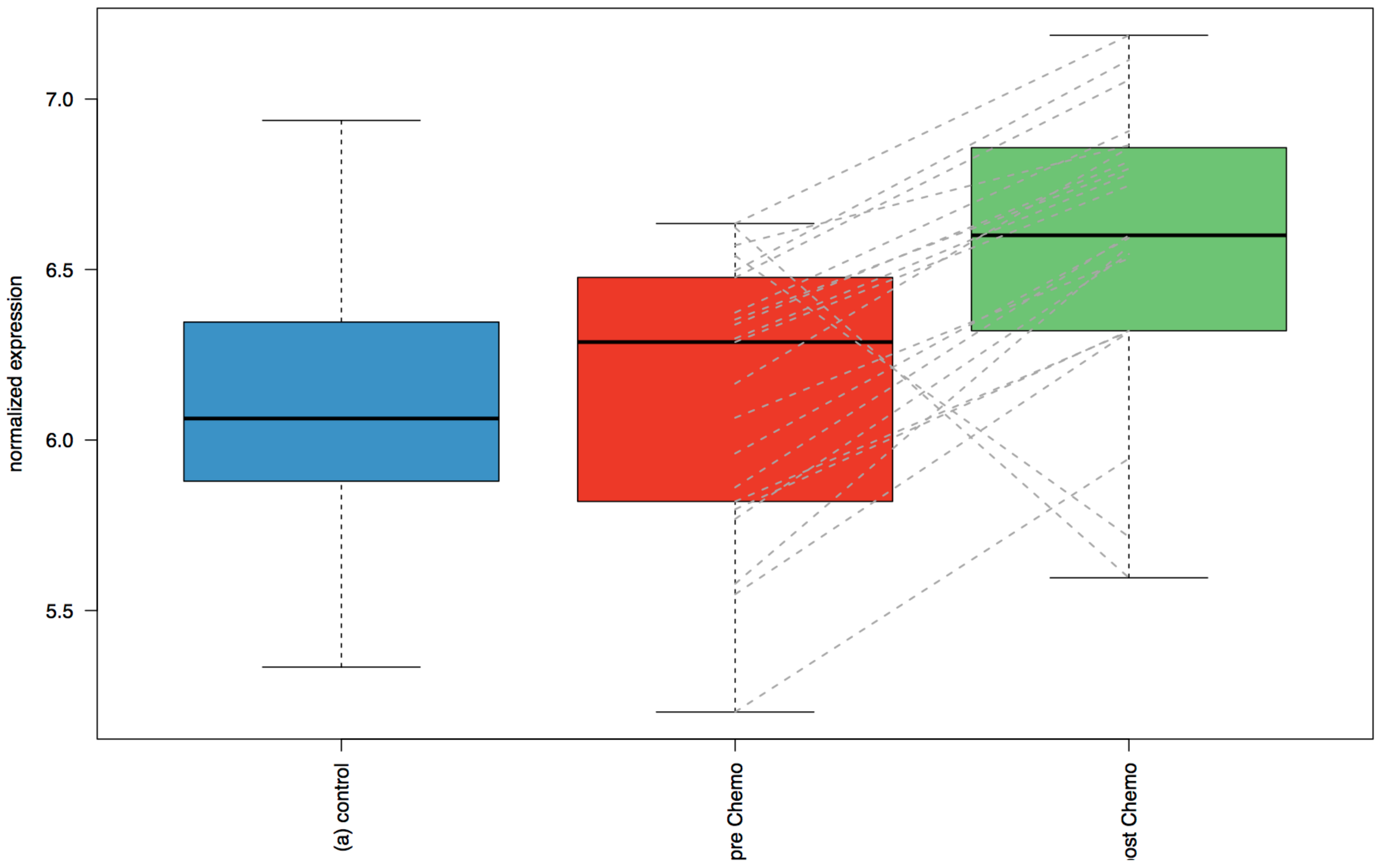
normalized expression
7.0
6.5
6.0
5.5
(a) control
pre Chemo
ost Chemo

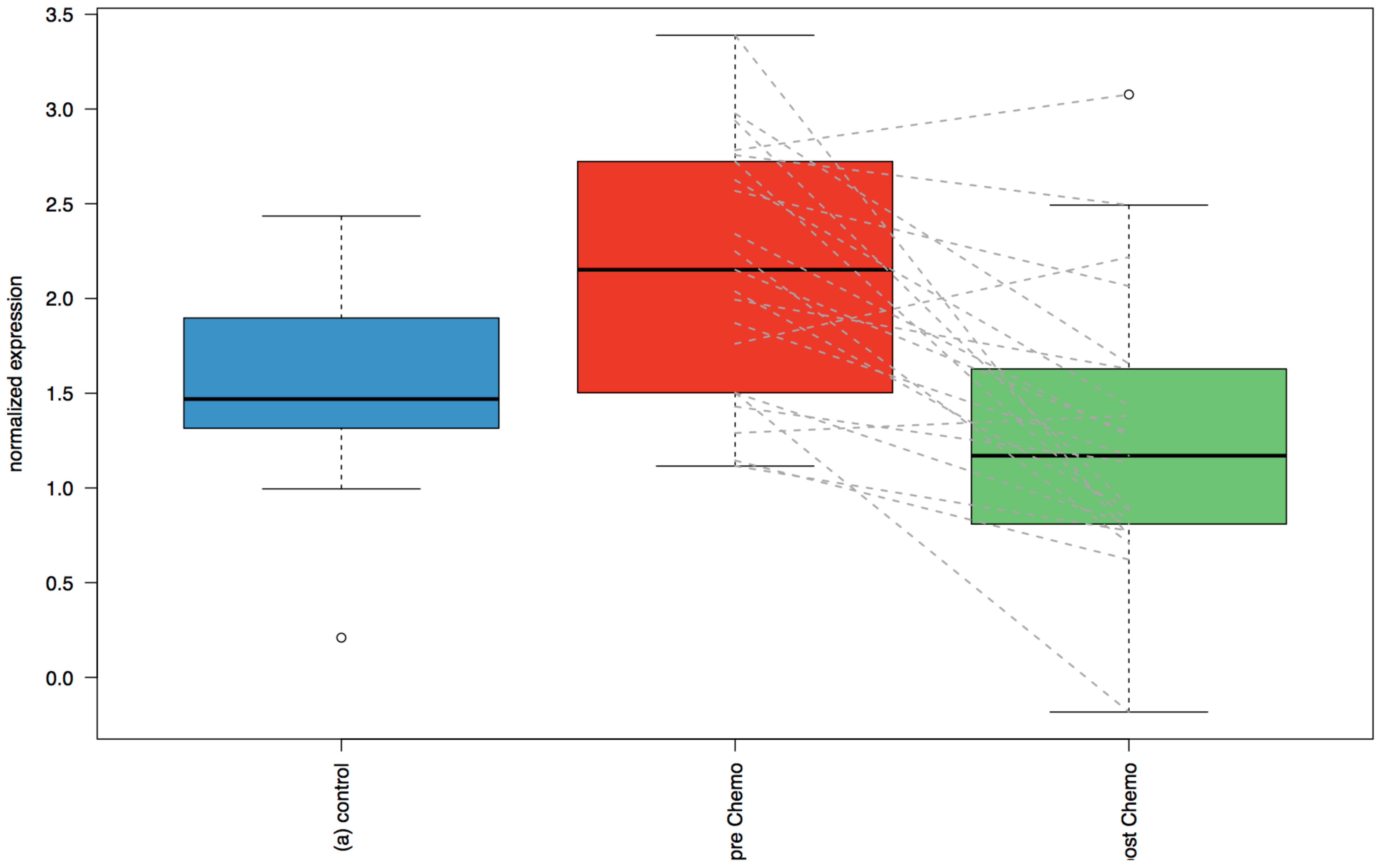
normalized expression
3.5
3.0
2.5
2.0
1.5
1.0
0.5
0.0
(a) control
pre Chemo
ost Chemo

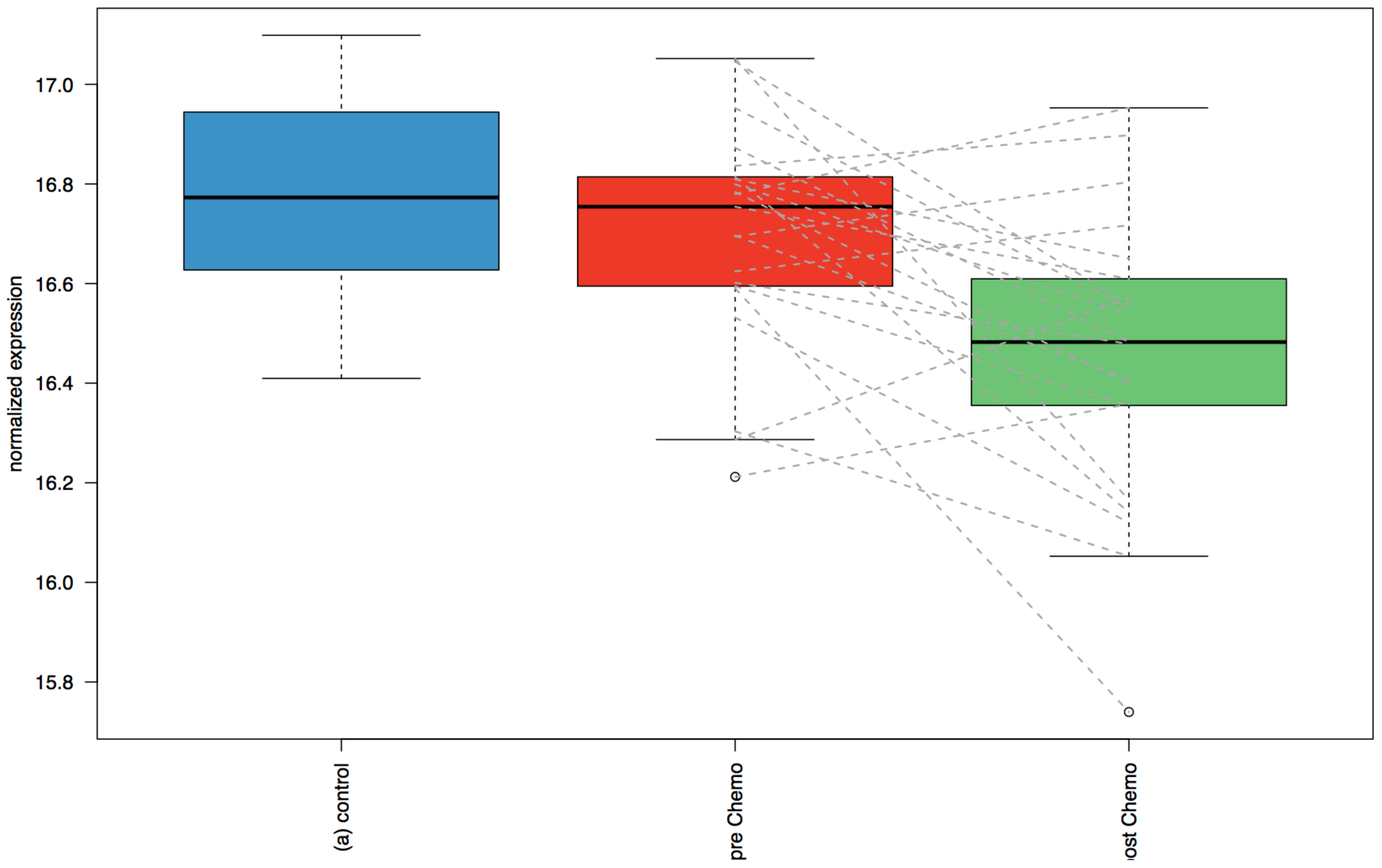
normalized expression
17.0
16.8
16.6
16.4
16.2
16.0
15.8
(a) control
pre Chemo
ost Chemo

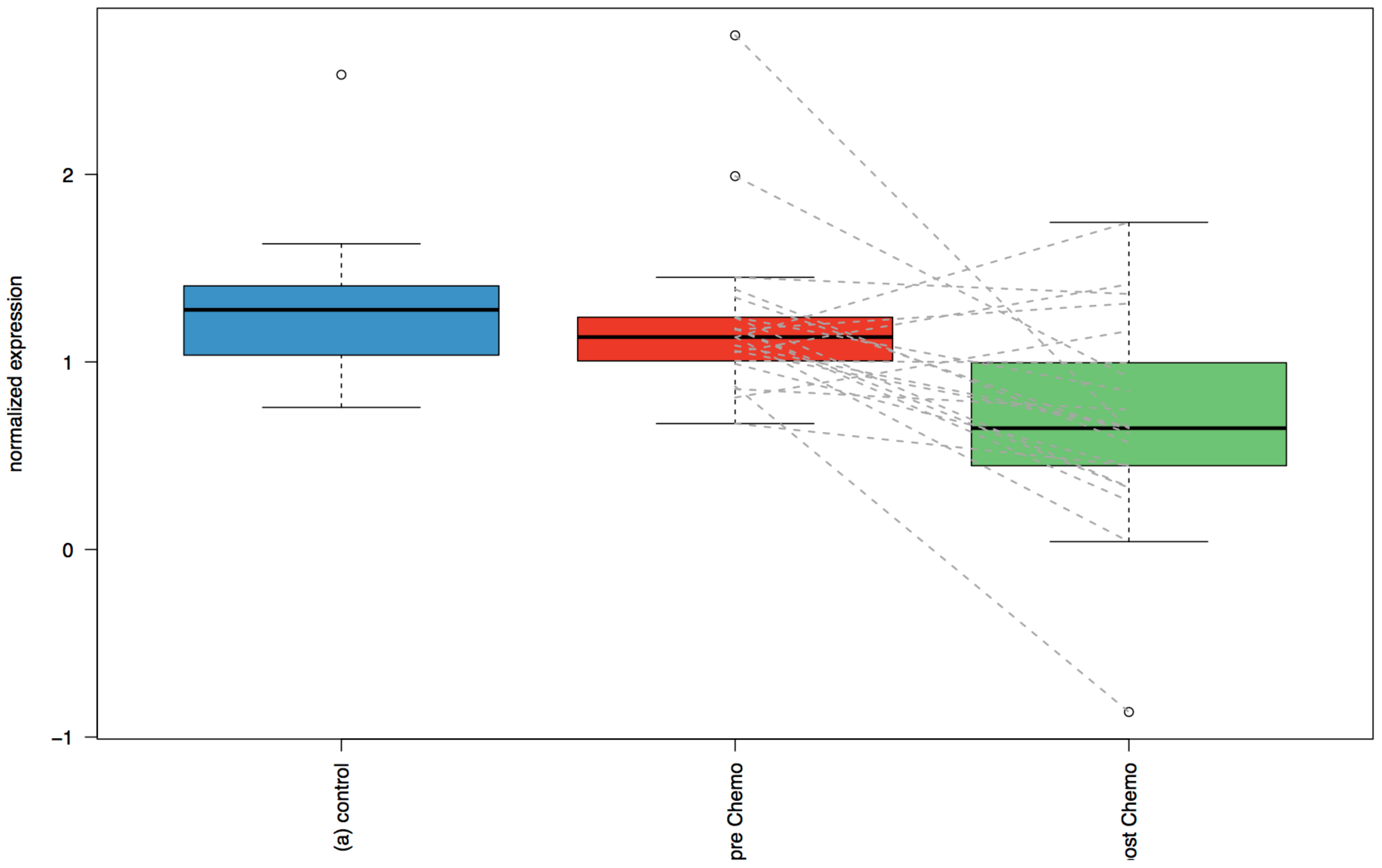

normalized expression
2
1
0
−1
(a) control
pre Chemo
ost Chemo

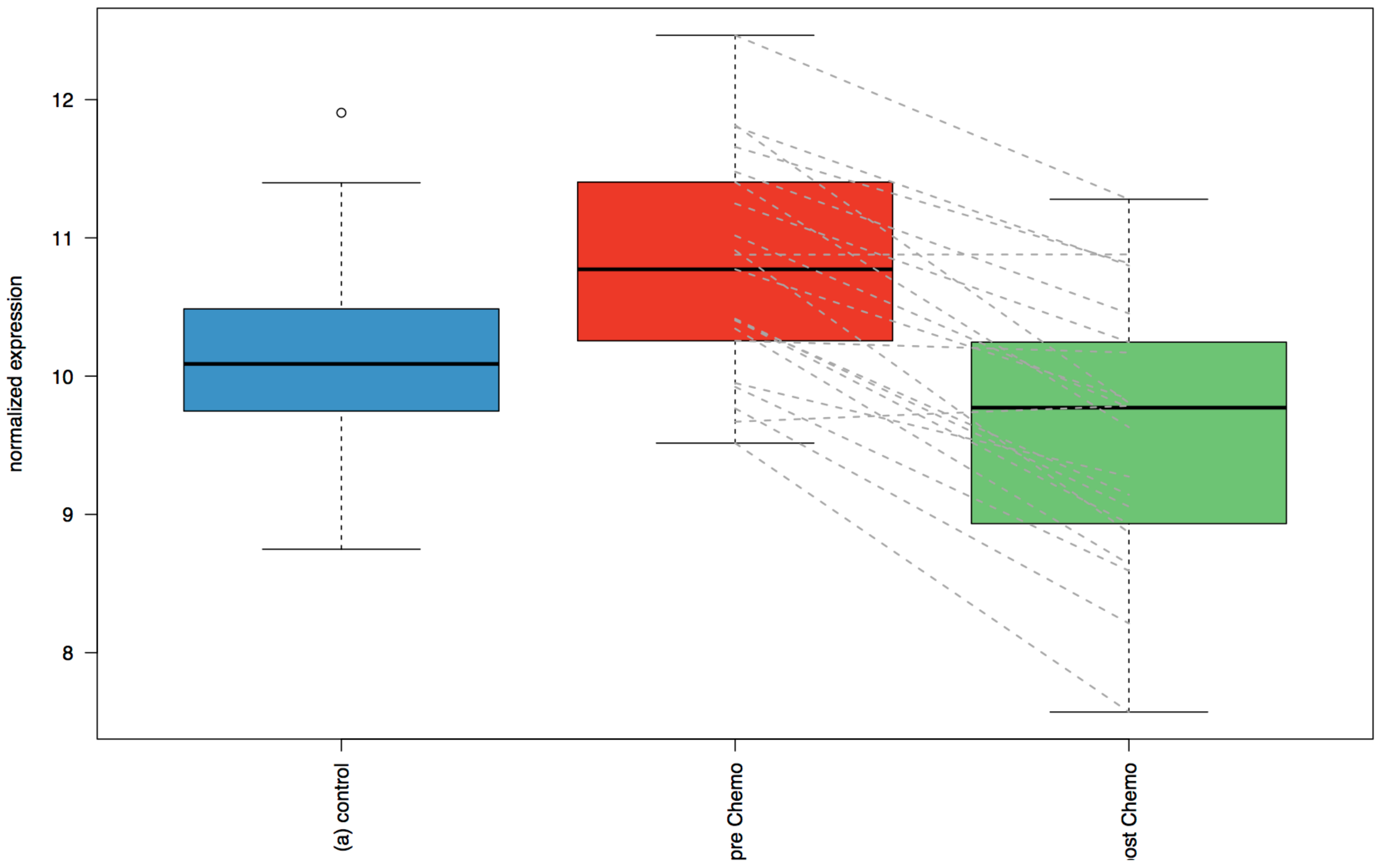

normalized expression
12
11
10
9
8
(a) control
pre Chemo
ost Chemo

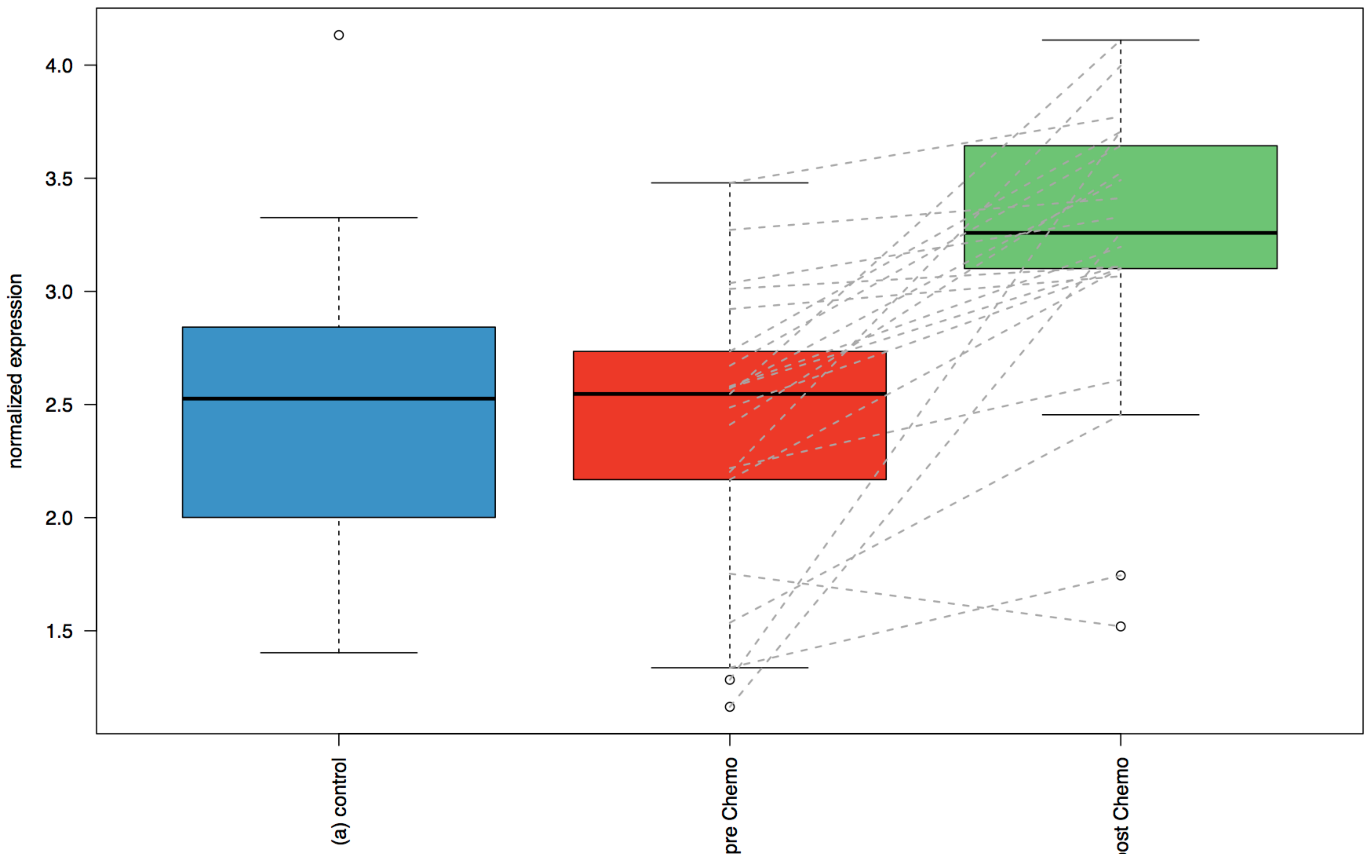
normalized expression
4.0
3.5
3.0
2.5
2.0
1.5
(a) control
pre Chemo
ost Chemo

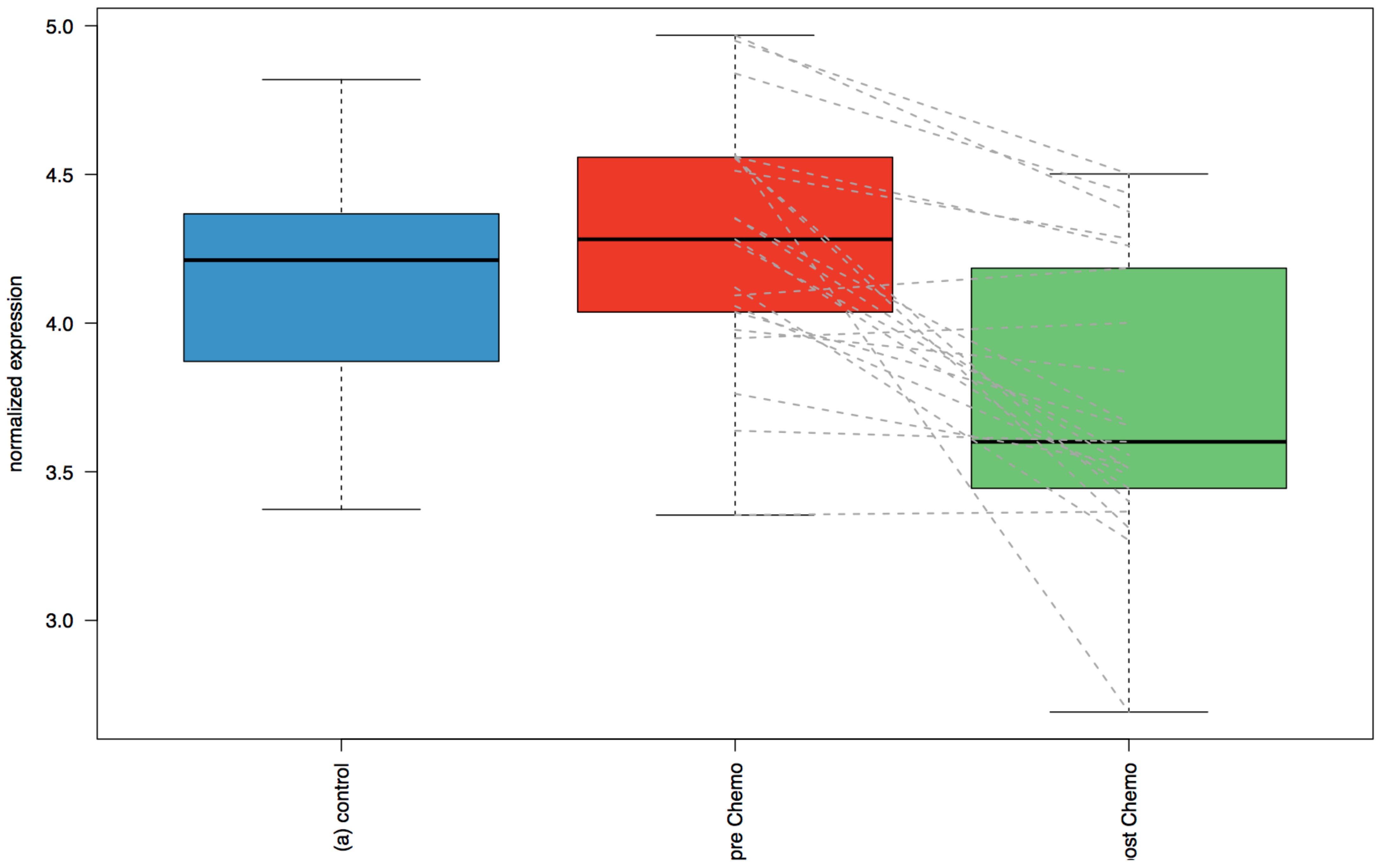

normalized expression
5.0
4.5
4.0
3.5
3.0
(a) control
pre Chemo
ost Chemo

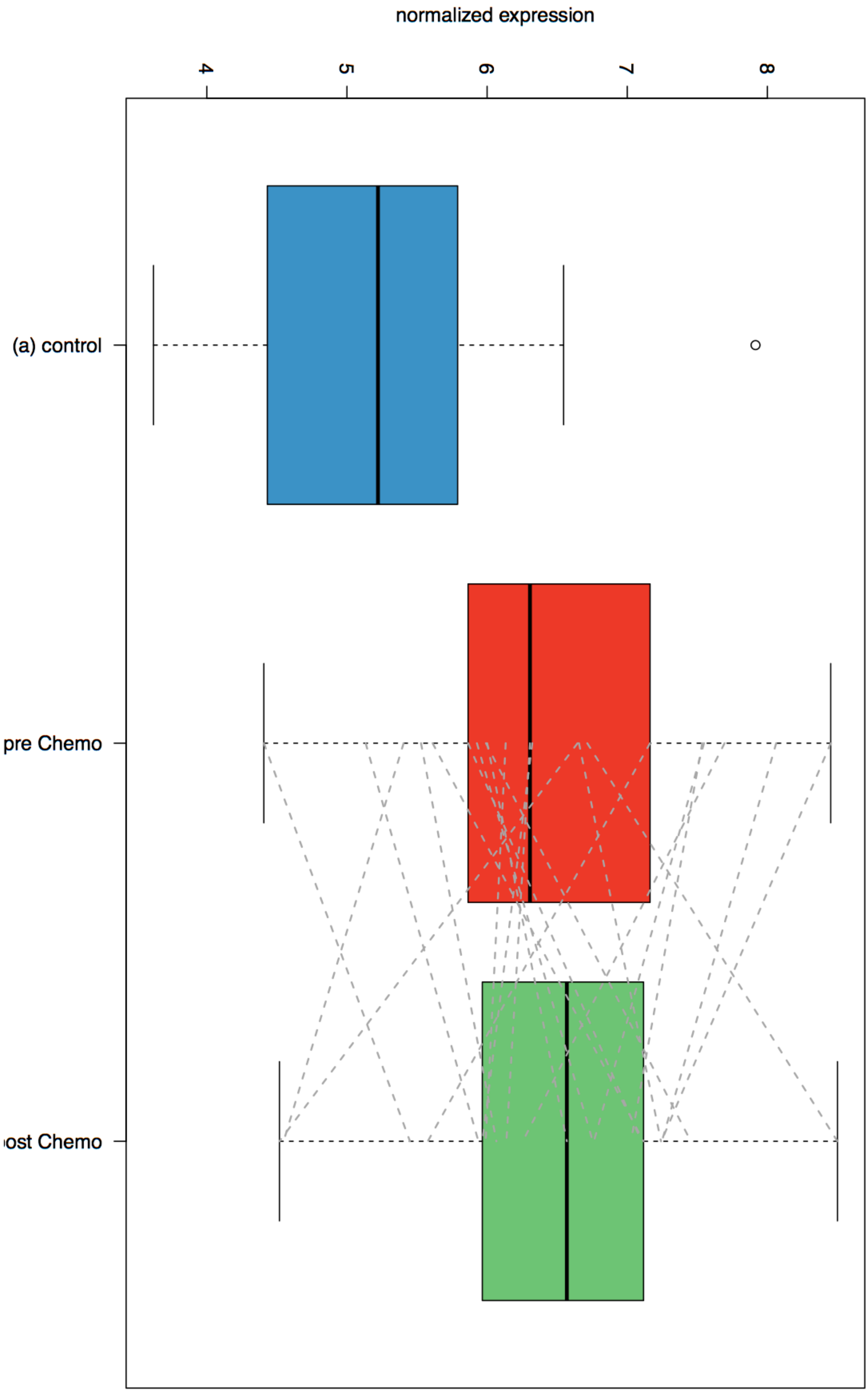
normalized expression
4
5
6
7
8
(a) control
pre Chemo
ost Chemo

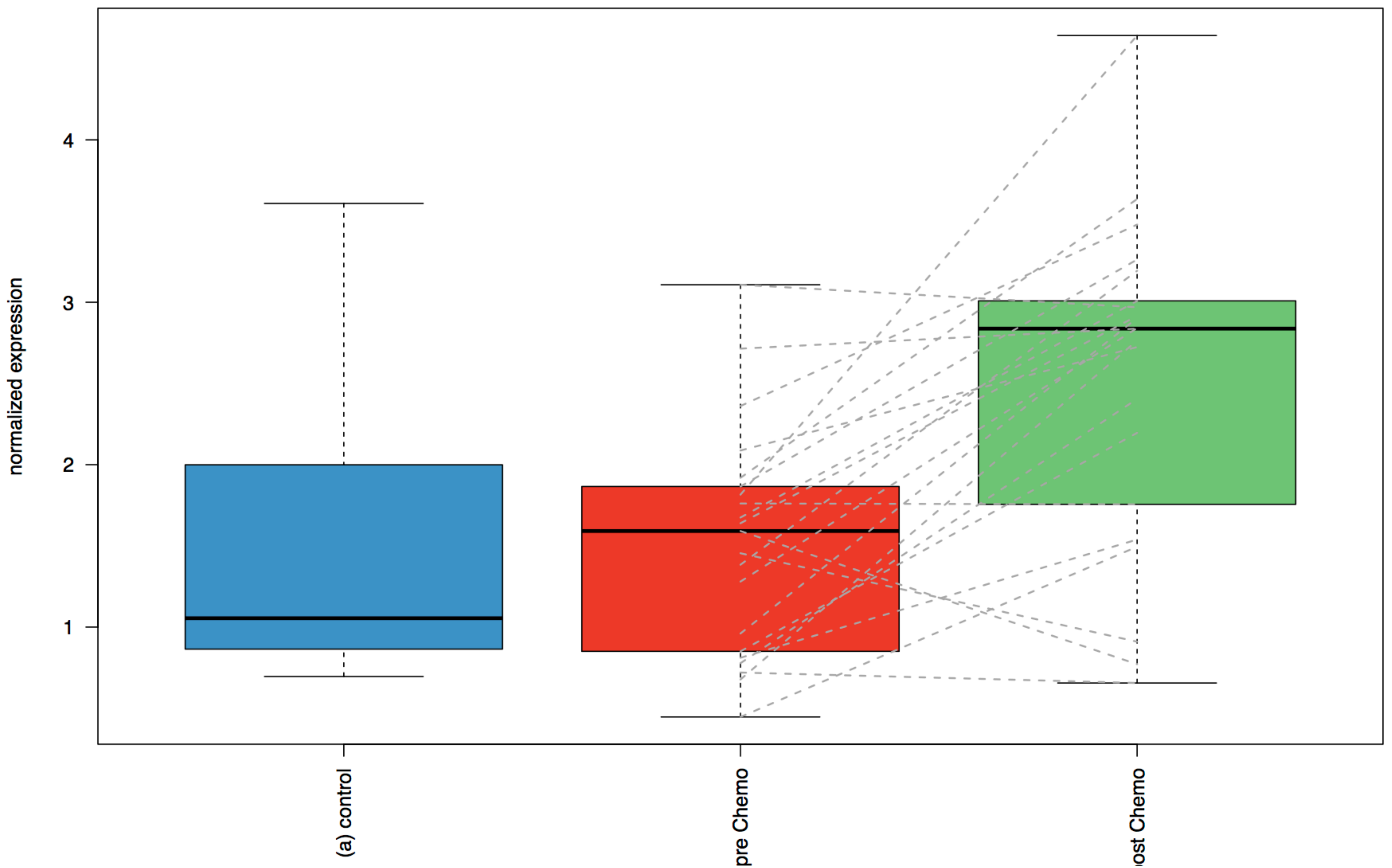
normalized expression
4
3
2
1
(a) control
pre Chemo
ost Chemo

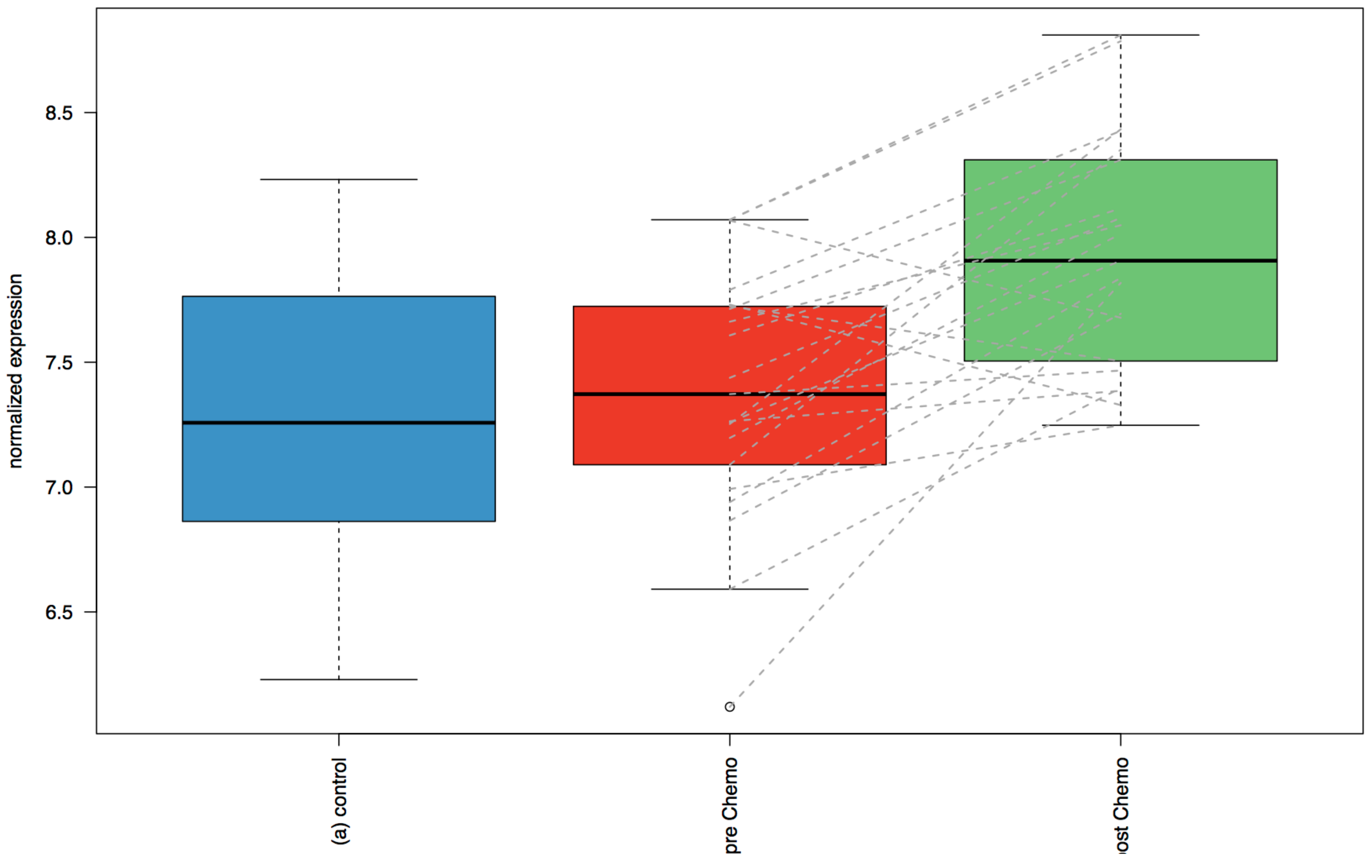
normalized expression
8.5
8.0
7.5
7.0
6.5
(a) control
pre Chemo
ost Chemo

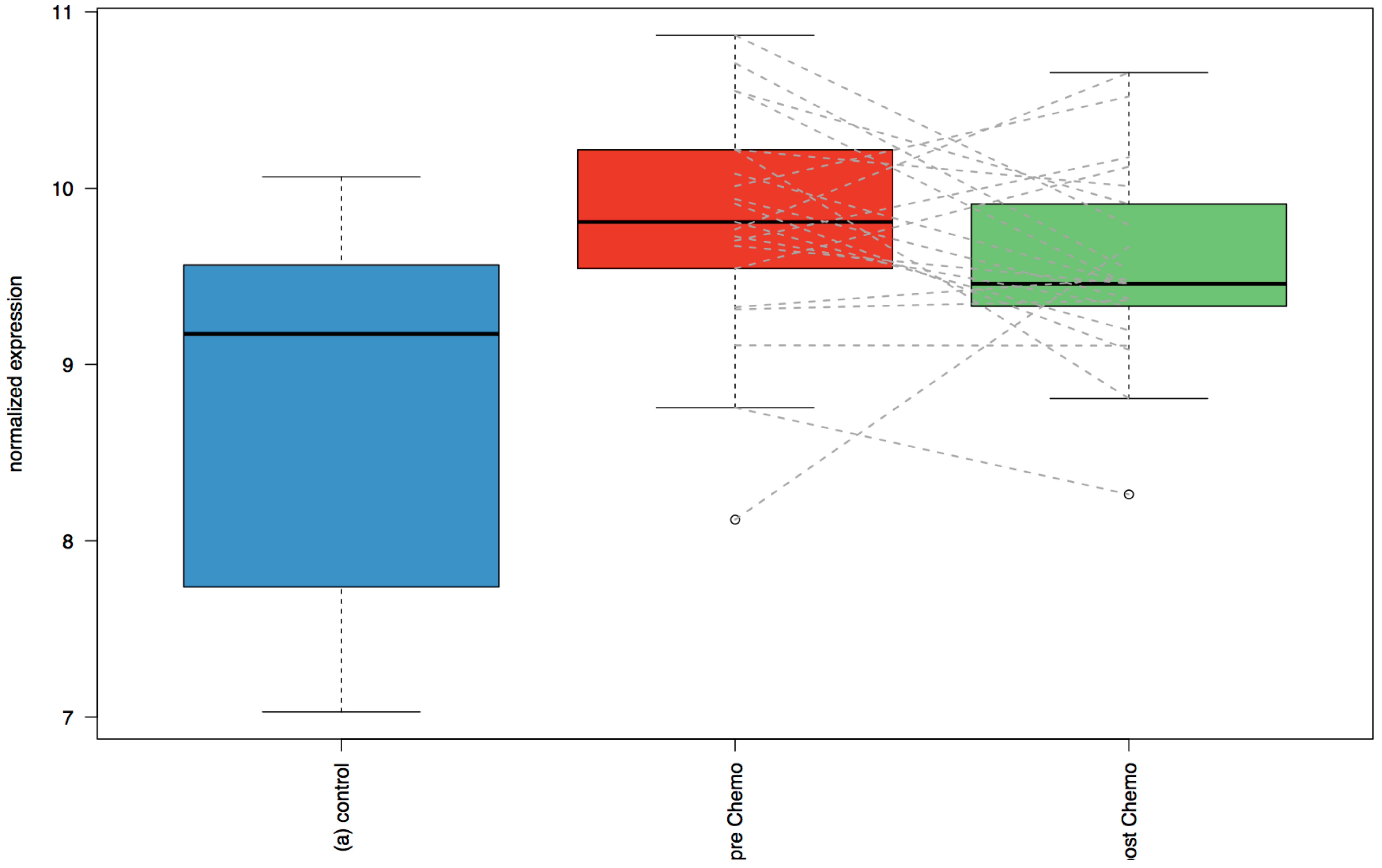
normalized expression
11
10
9
8
7
(a) control
pre Chemo
ost Chemo

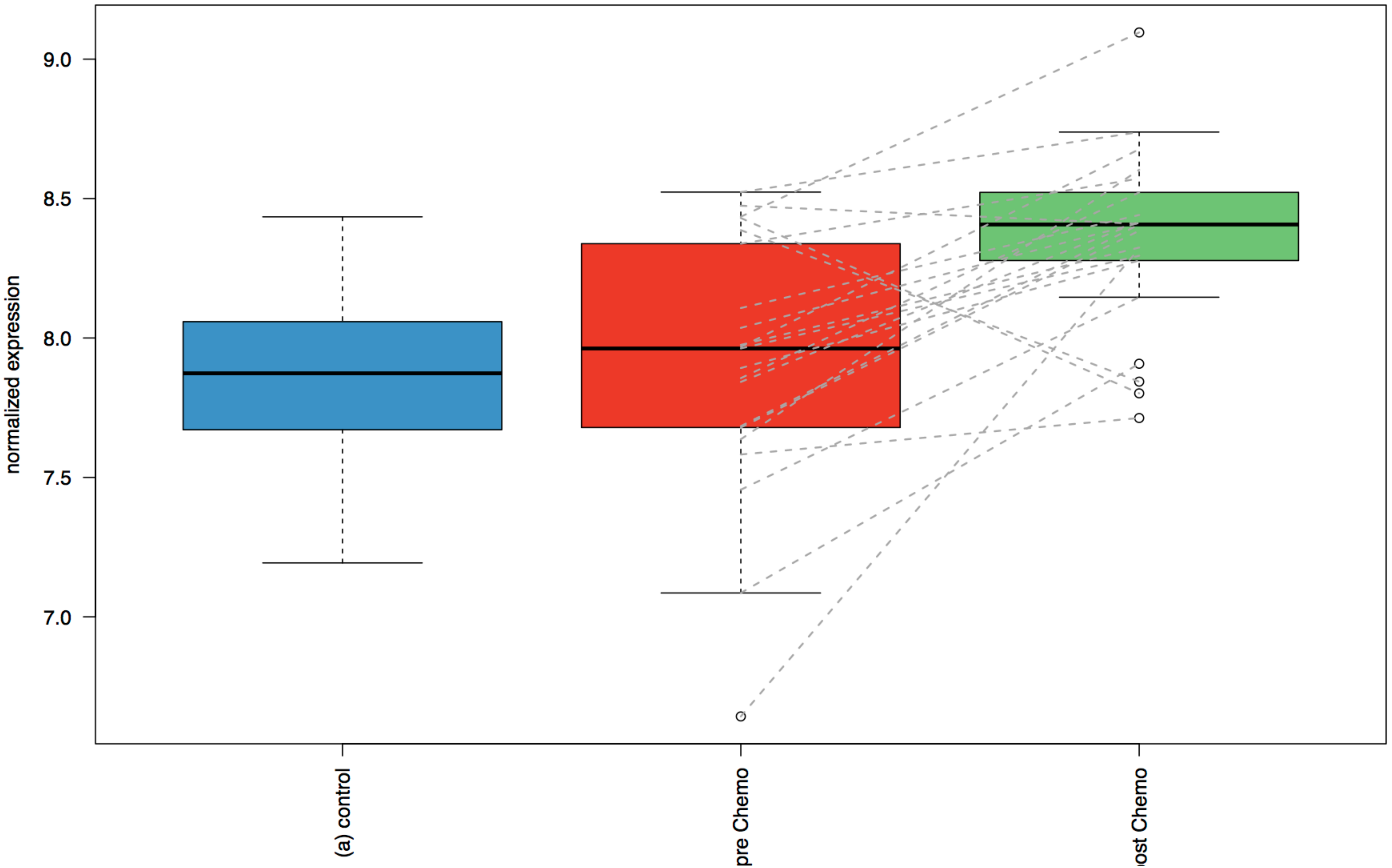

normalized expression
9.0
8.5
8.0
7.5
7.0
(a) control
pre Chemo
ost Chemo

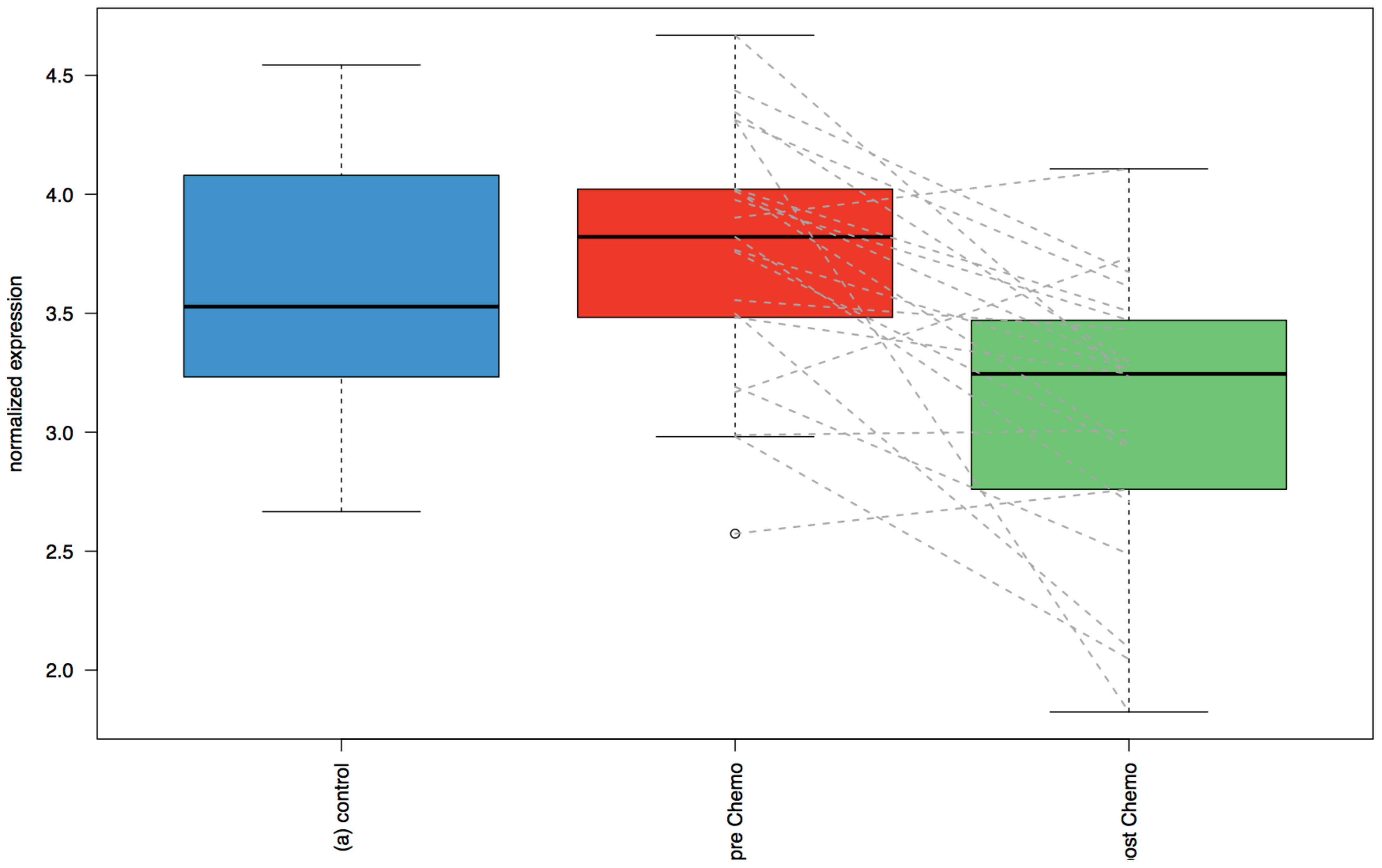

normalized expression
4.5
4.0
3.5
3.0
2.5
2.0
(a) control
pre Chemo
ost Chemo

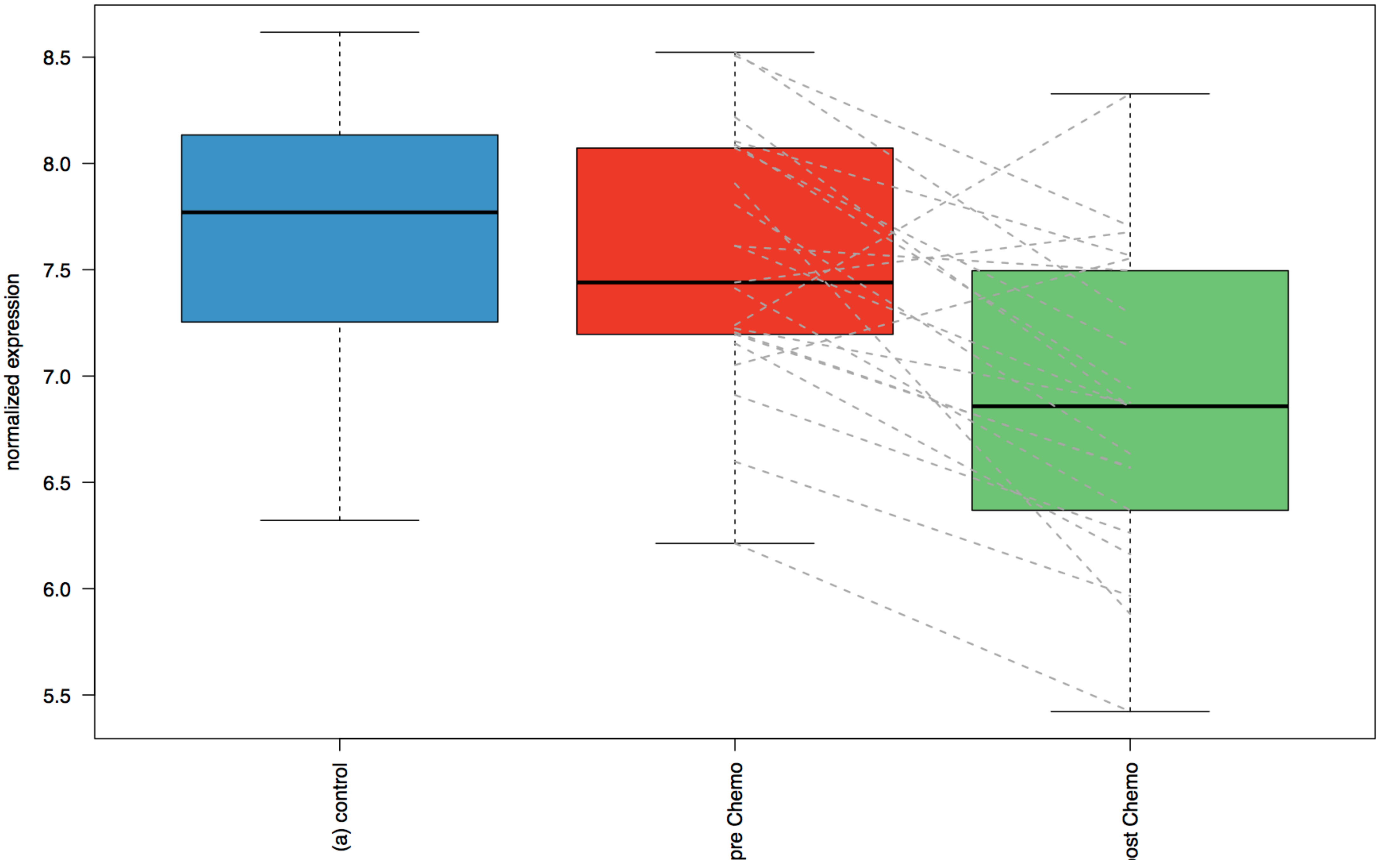

normalized expression
8.5
8.0
7.5
7.0
6.5
6.0
5.5
(a) control
pre Chemo
ost Chemo

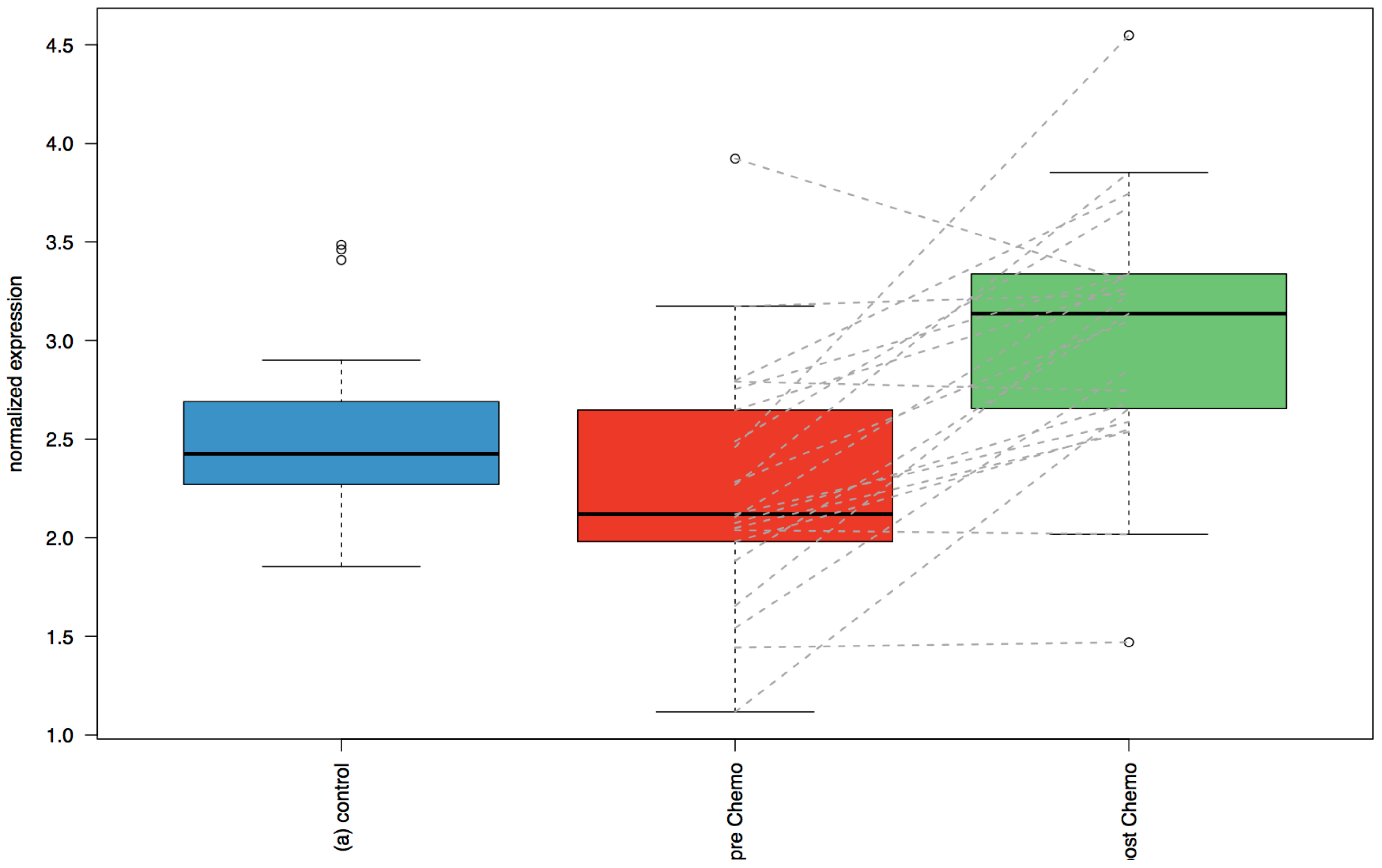
normalized expression
4.5
4.0
3.5
3.0
2.5
2.0
1.5
1.0
(a) control
pre Chemo
ost Chemo

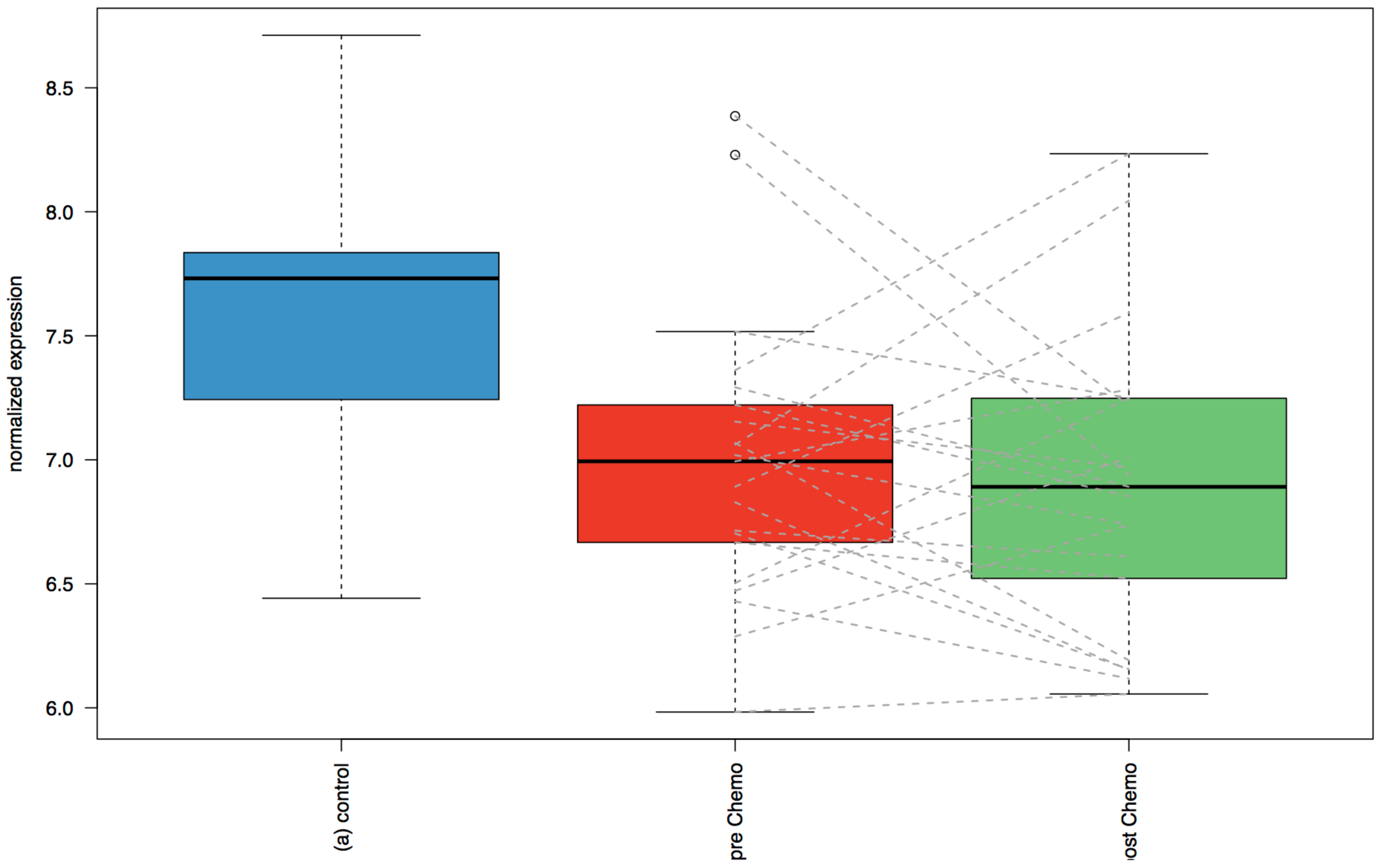
normalized expression
8.5
8.0
7.5
7.0
6.5
6.0
(a) control
pre Chemo
ost Chemo

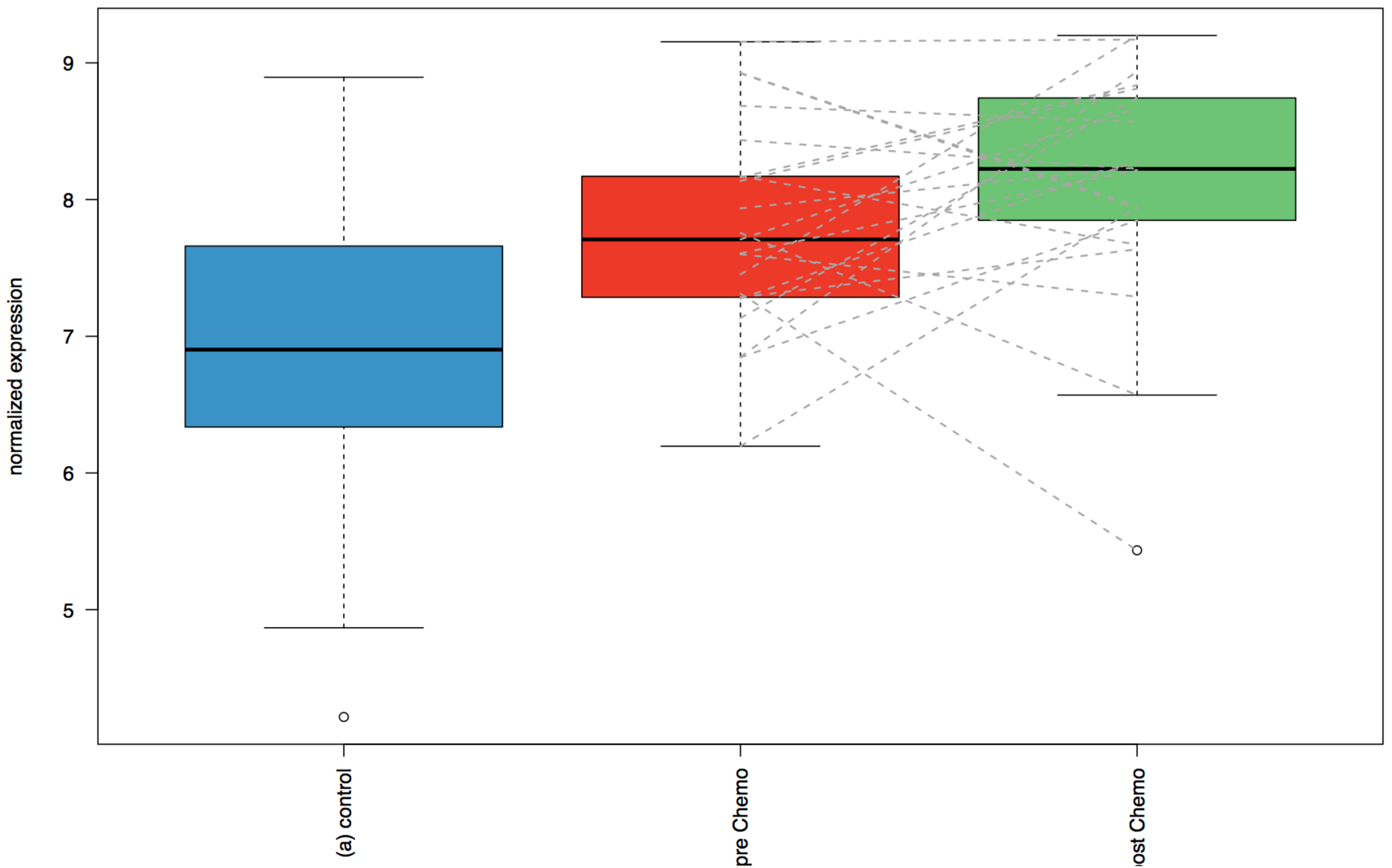

normalized expression
9
8
7
6
5
(a) control
pre Chemo
ost Chemo

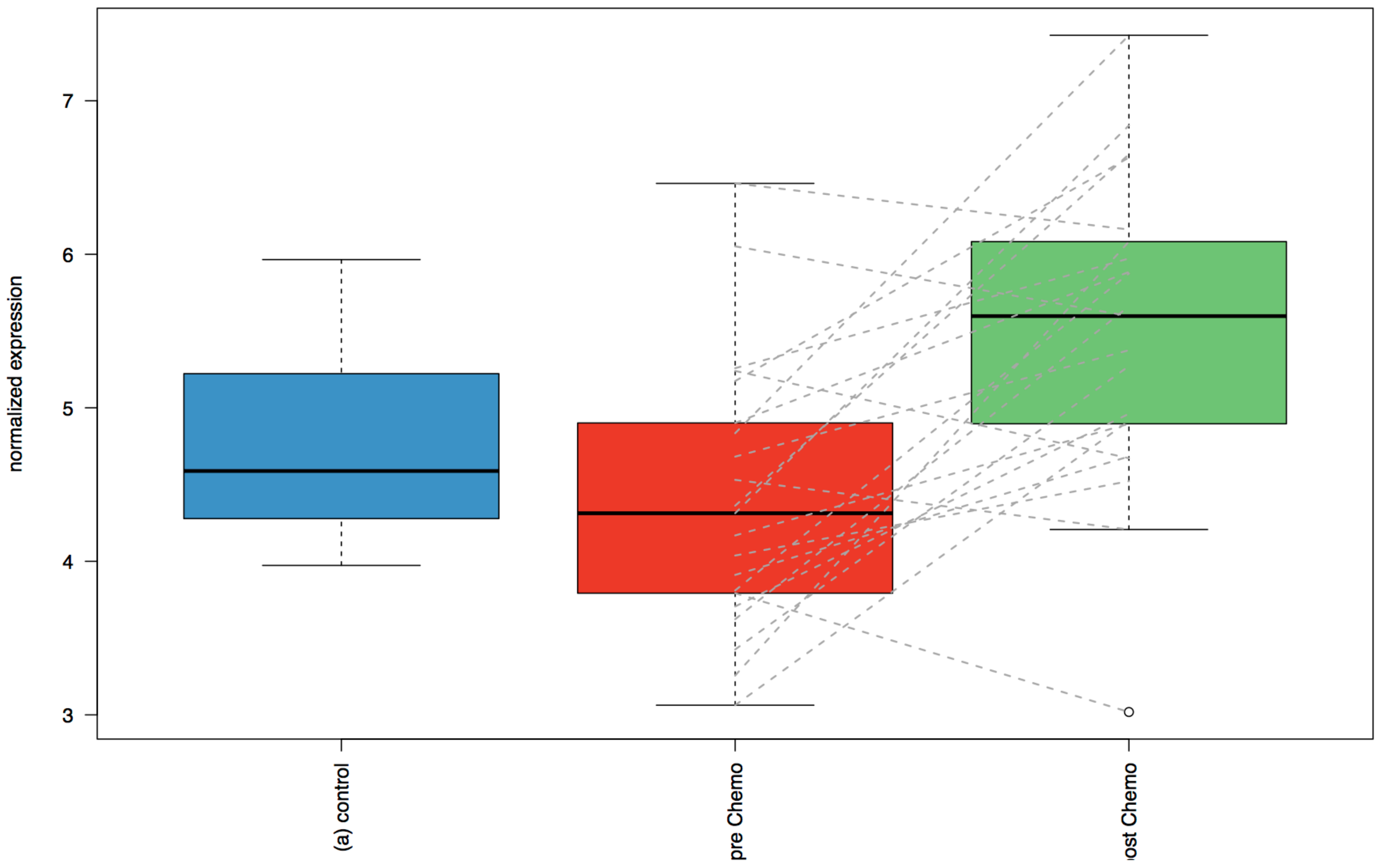
normalized expression
3
4
5
6
7
(a) control
pre Chemo
ost Chemo

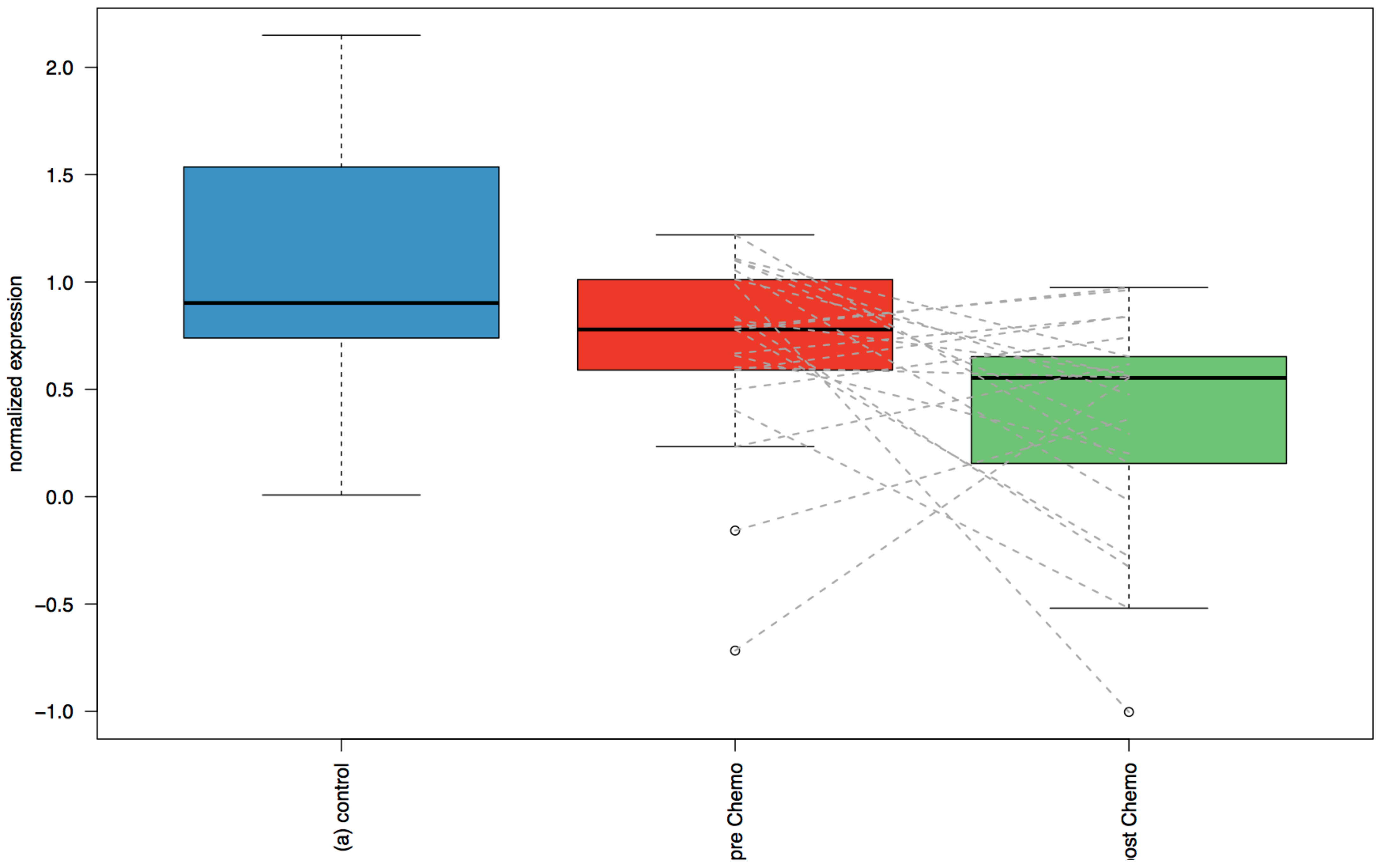
normalized expression
2.0
1.5
1.0
0.5
0.0
−0.5
−1.0
(a) control
pre Chemo
ost Chemo

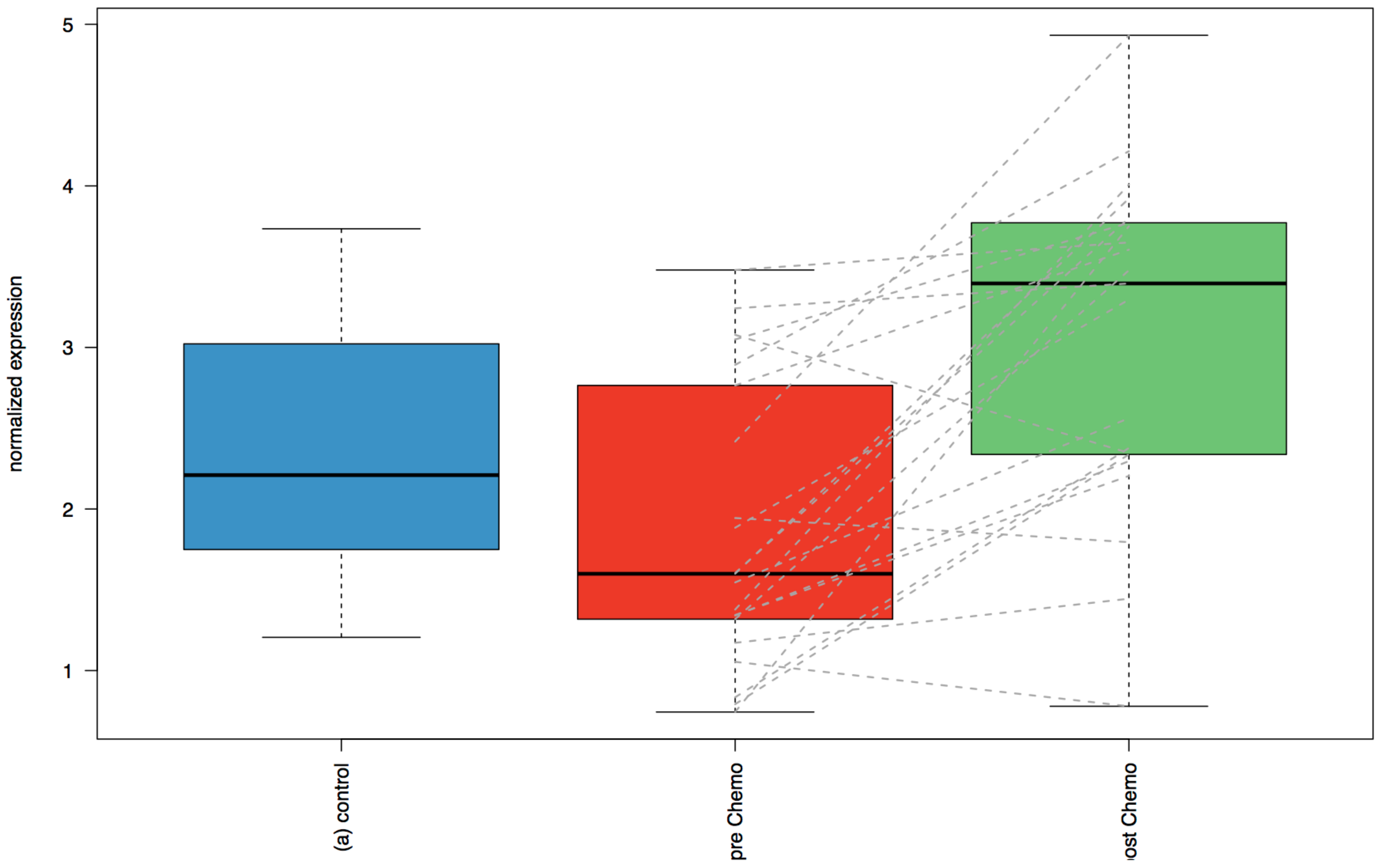

normalized expression
5
4
3
2
1
(a) control
pre Chemo
ost Chemo

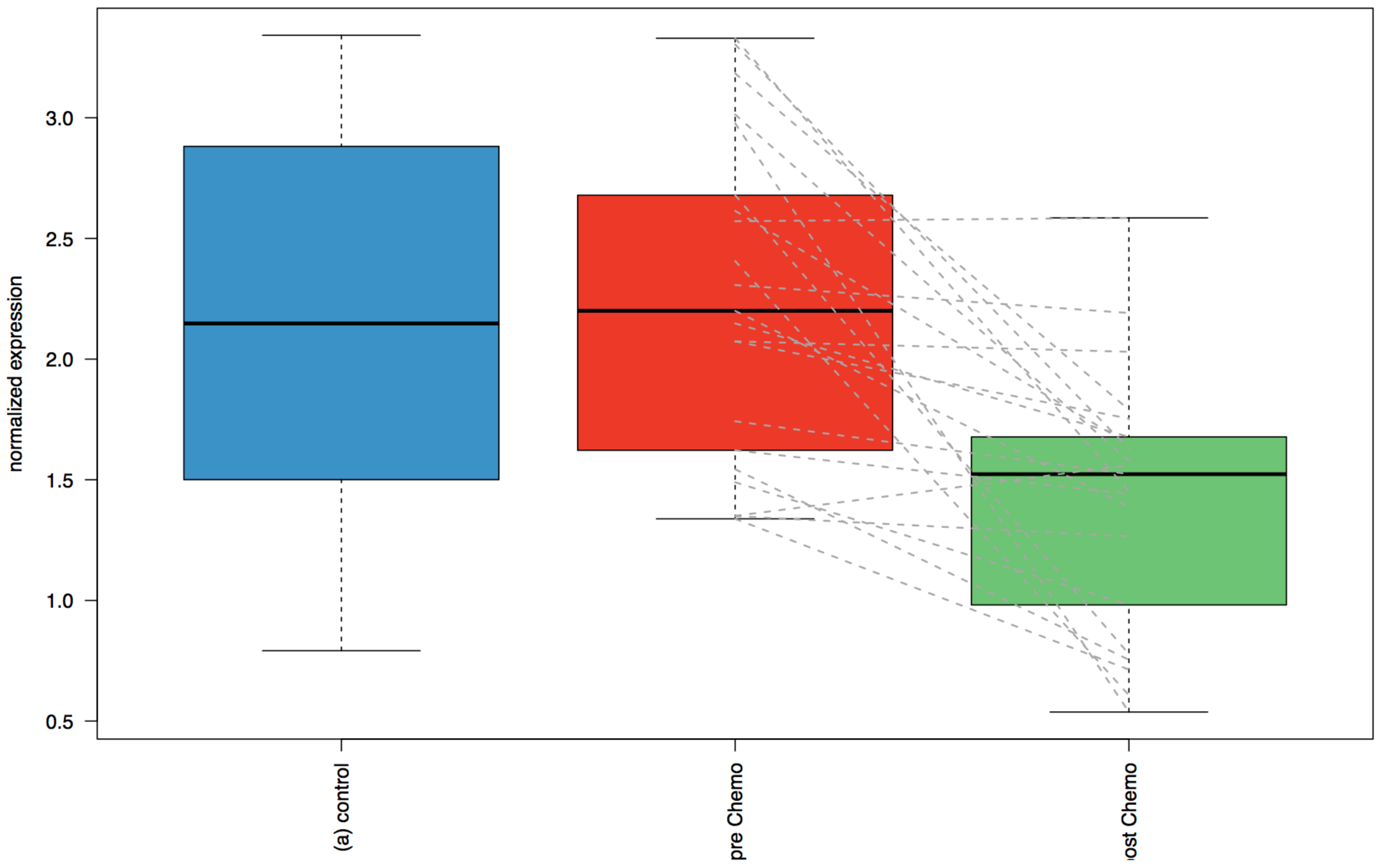

normalized expression
3.0
2.5
2.0
1.5
1.0
0.5
(a) control
pre Chemo
ost Chemo

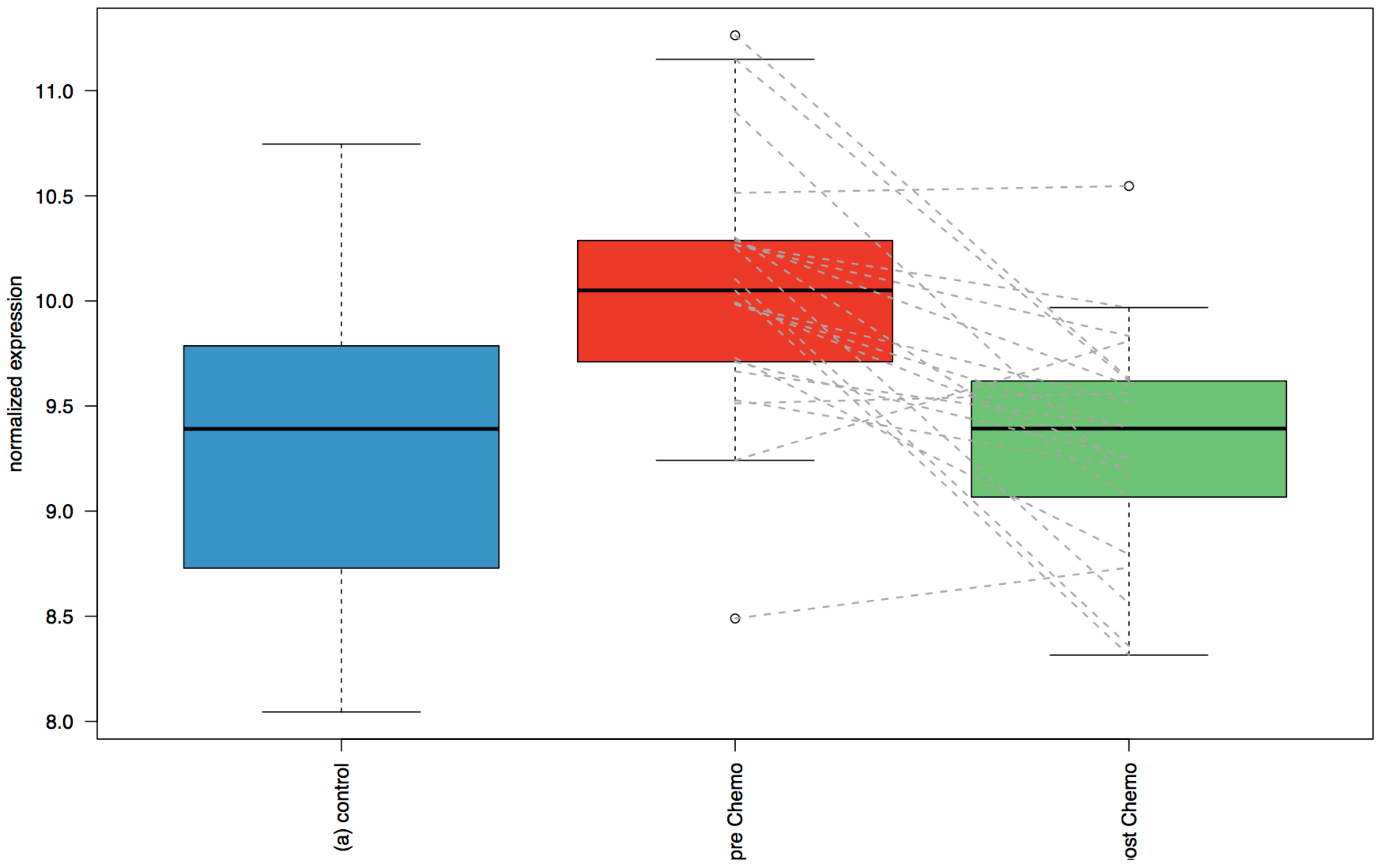
normalized expression
11.0
10.5
10.0
9.5
9.0
8.5
8.0
(a) control
pre Chemo
ost Chemo

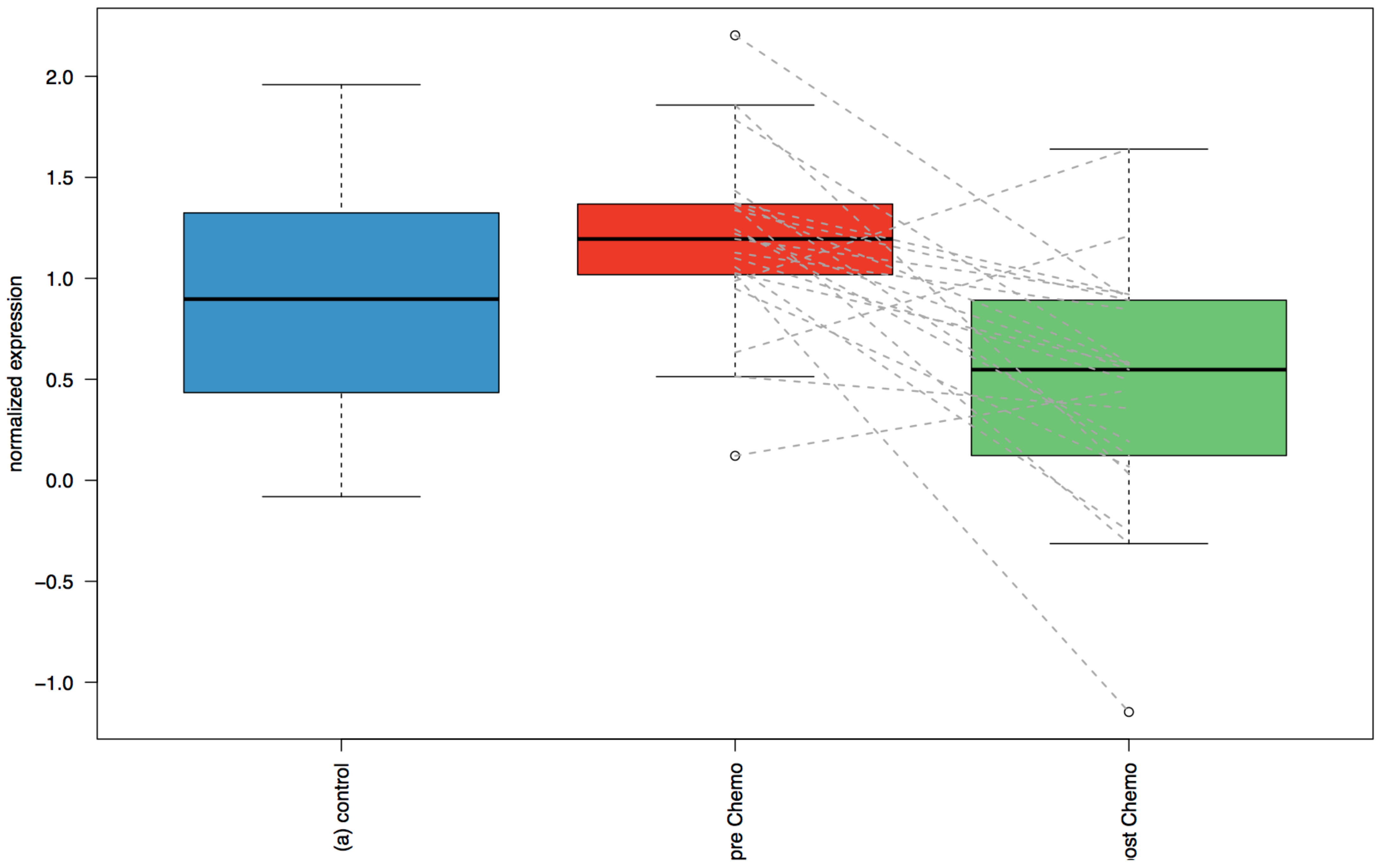

2.0
1.5
1.0
0.5
0.0
−0.5
−1.0
normalized expression
(a) control
pre Chemo
ost Chemo

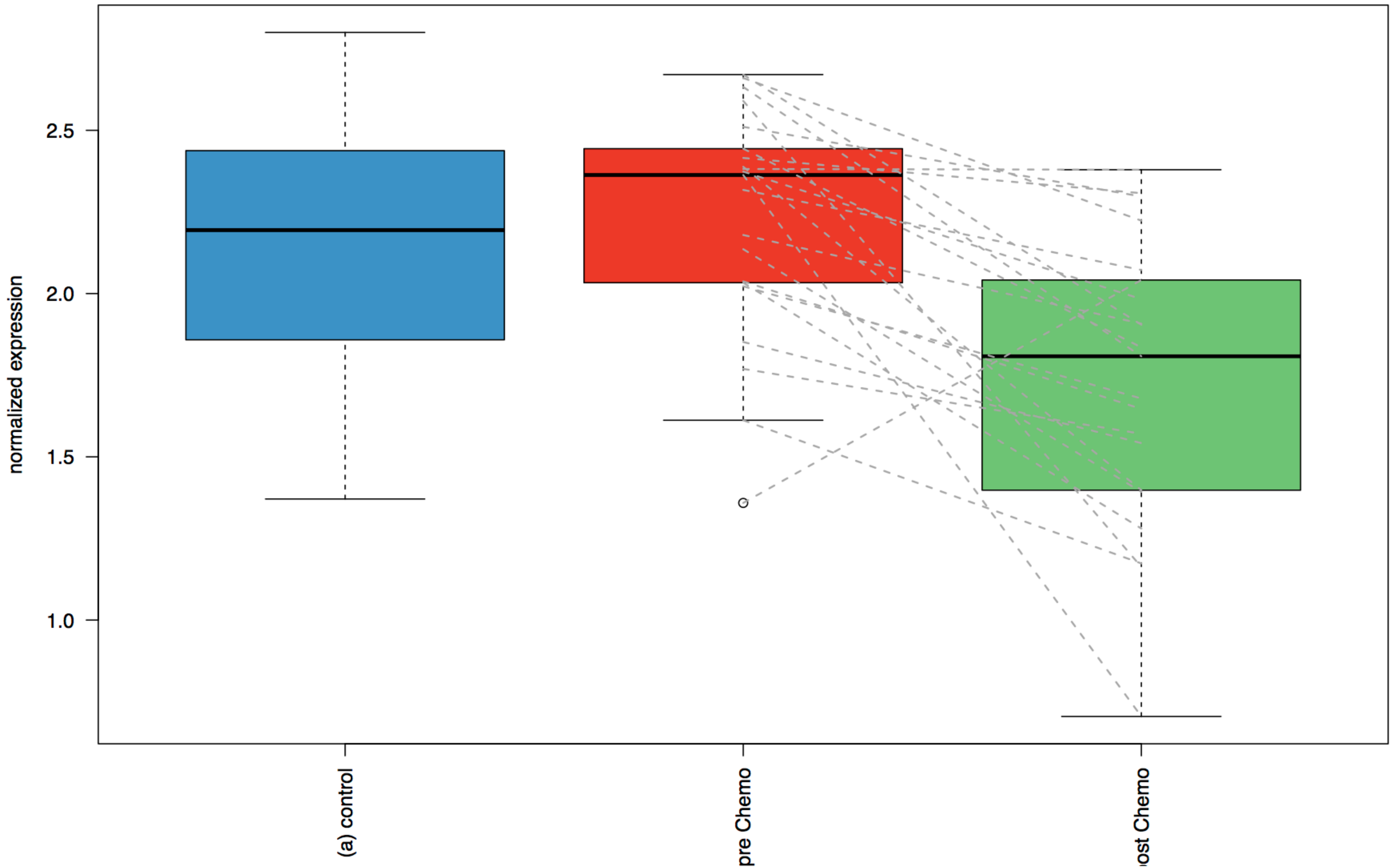
normalized expression
2.5
2.0
1.5
1.0
(a) control
pre Chemo
ost Chemo

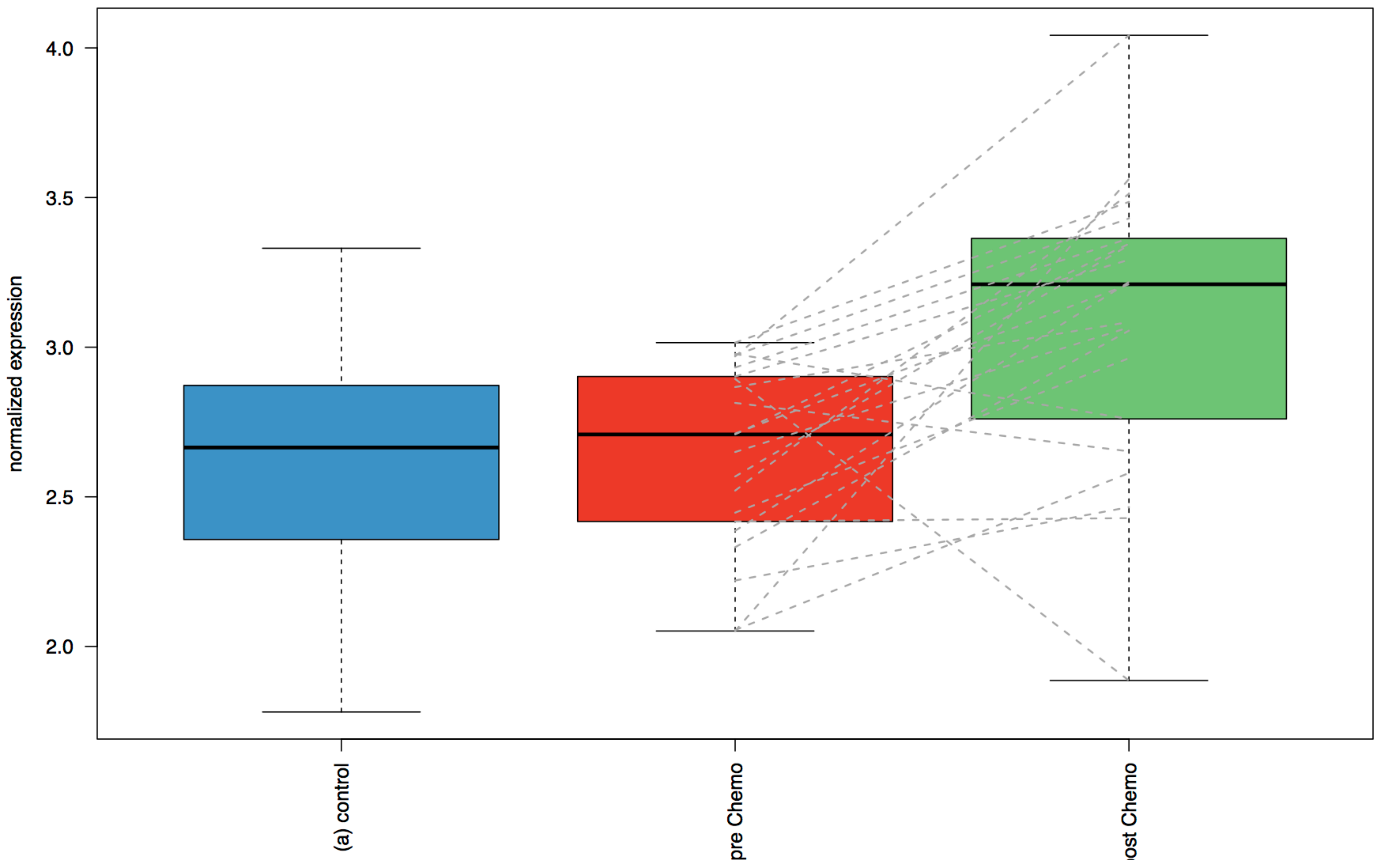
normalized expression
4.0
3.5
3.0
2.5
2.0
(a) control
pre Chemo
ost Chemo

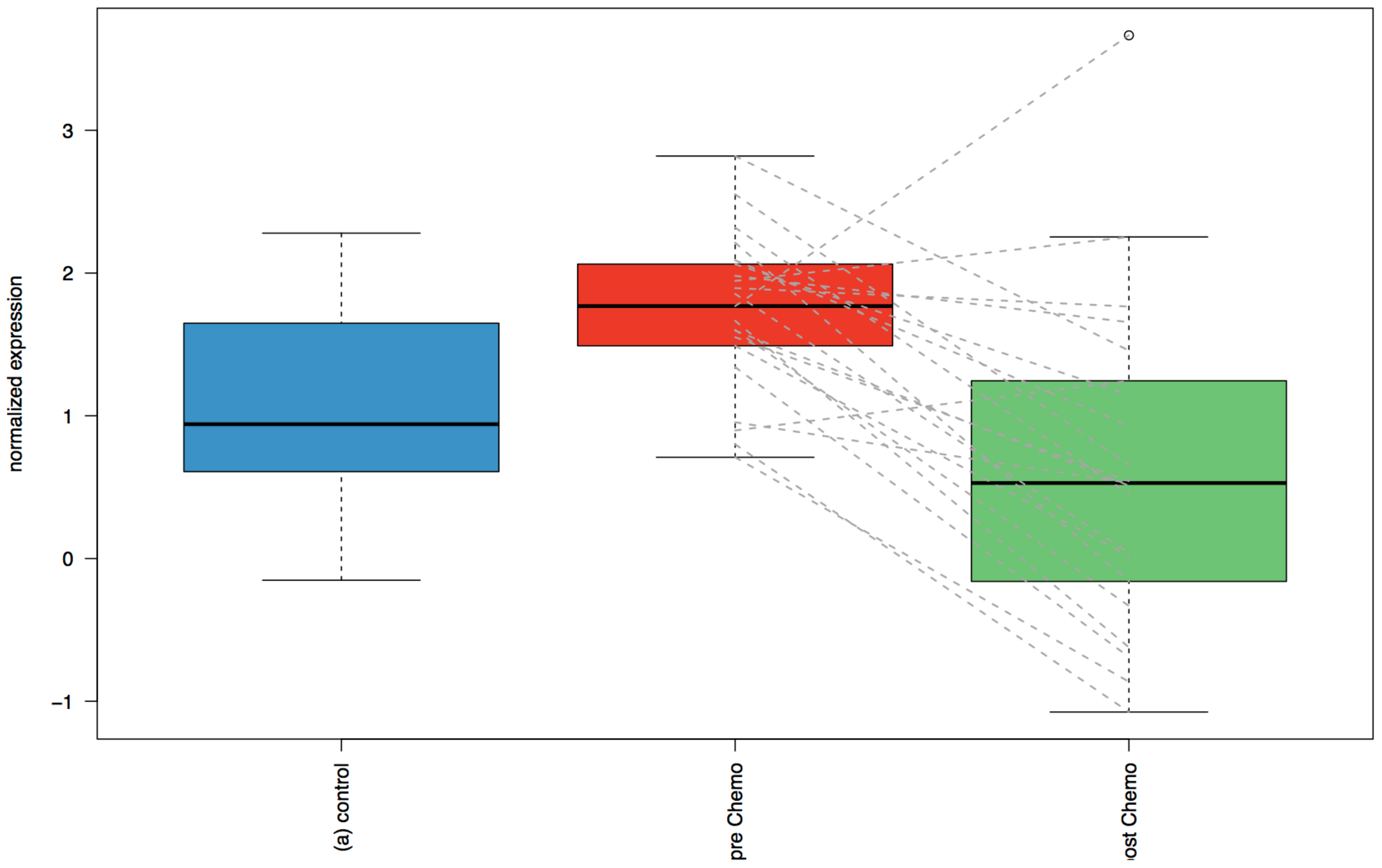
normalized expression
3
2
1
0
−1
(a) control
pre Chemo
ost Chemo

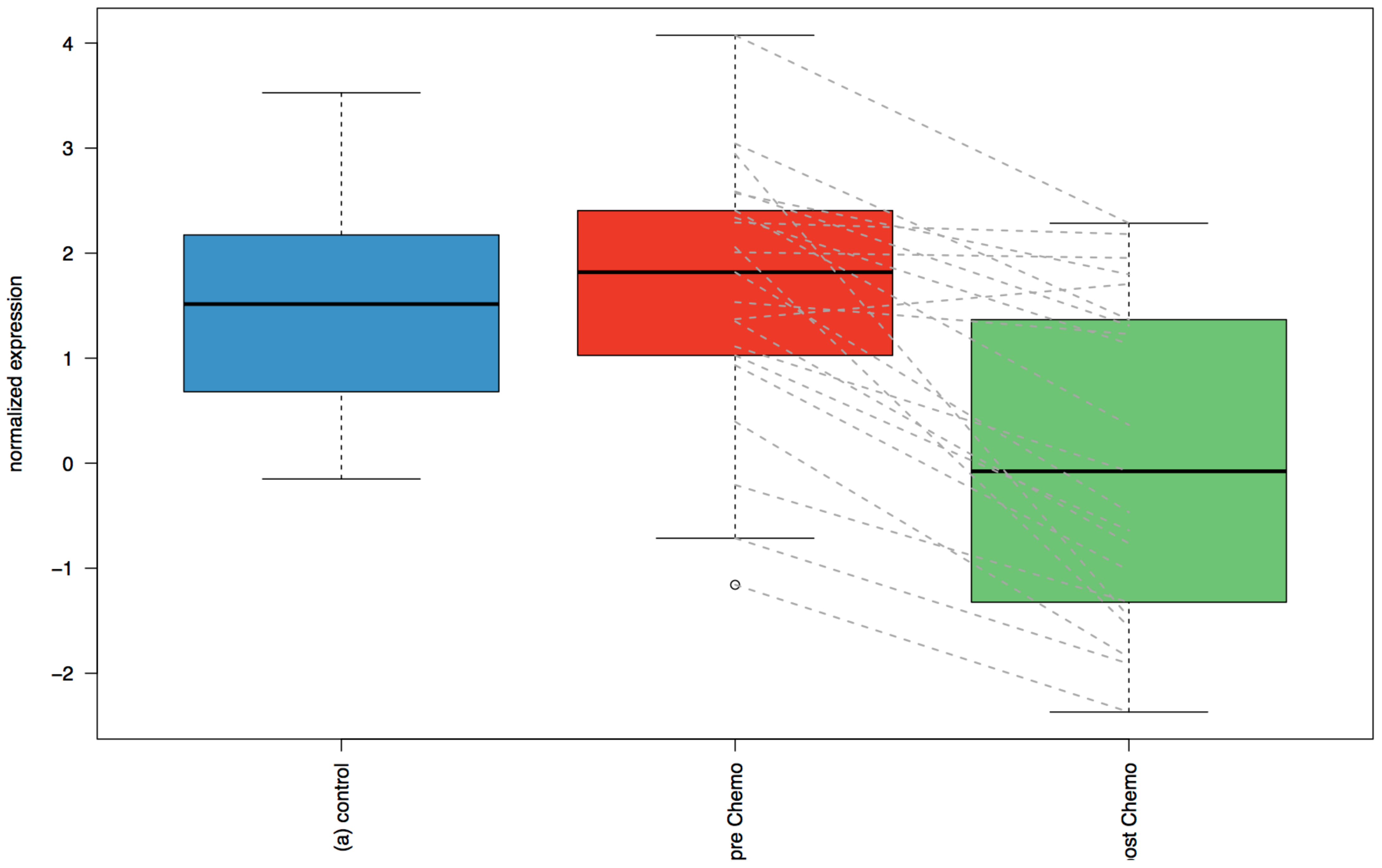
normalized expression
4
3
2
1
0
−1
−2
(a) control
pre Chemo
ost Chemo

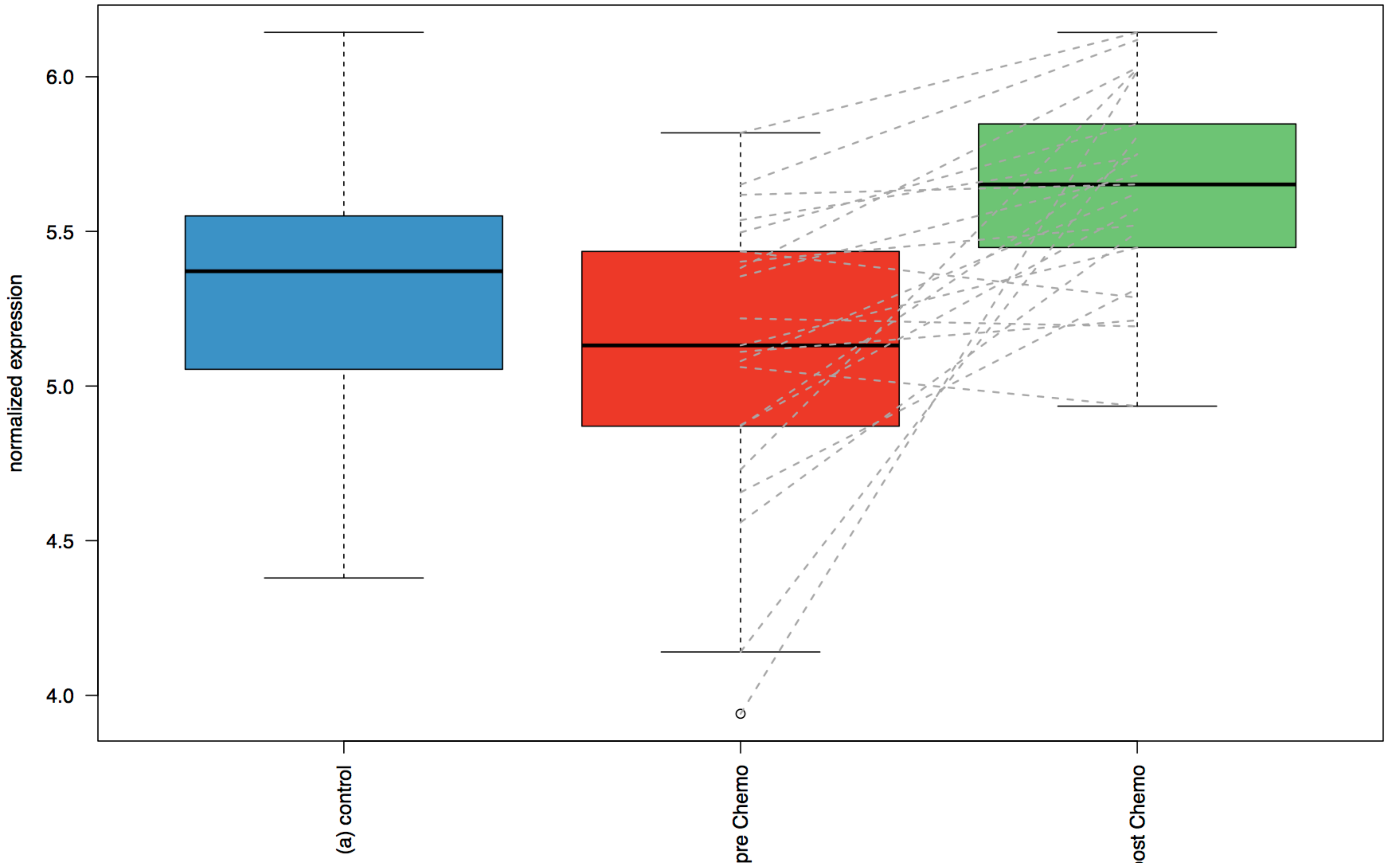

normalized expression
6.0
5.5
5.0
4.5
4.0
(a) control
pre Chemo
ost Chemo

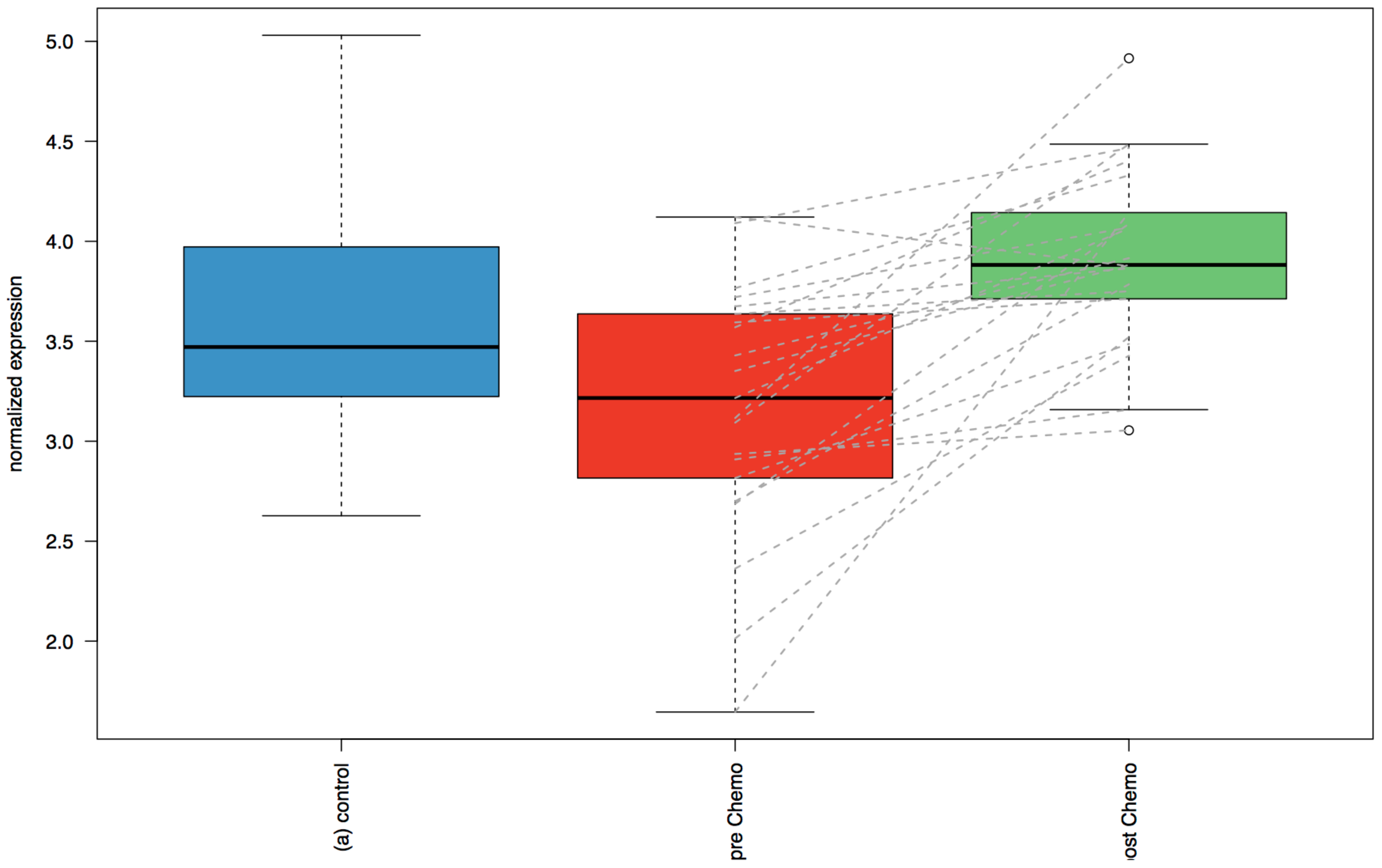
normalized expression
5.0
4.5
4.0
3.5
3.0
2.5
2.0
(a) control
pre Chemo
ost Chemo

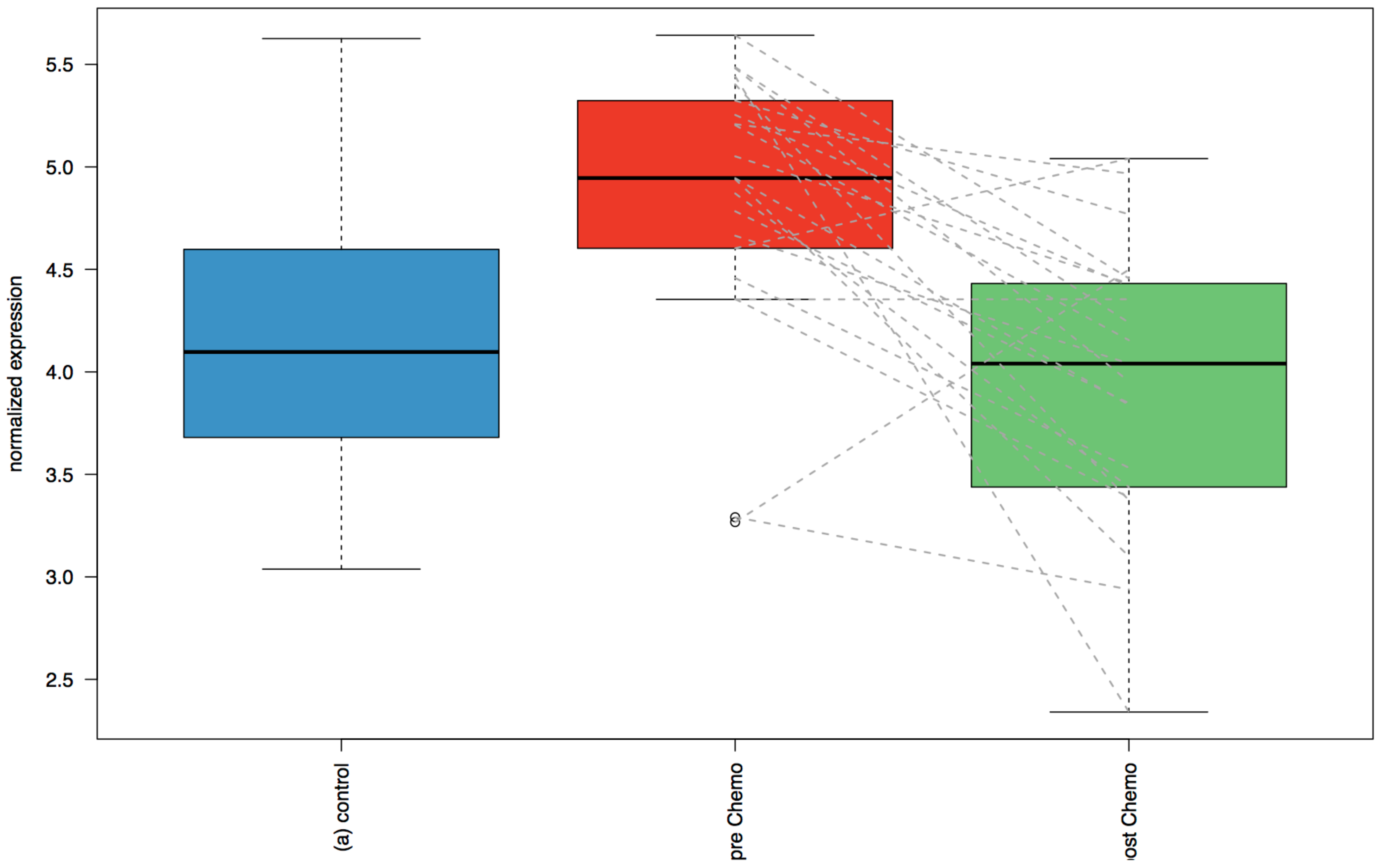
normalized expression
5.5
5.0
4.5
4.0
3.5
3.0
2.5
(a) control
pre Chemo
ost Chemo

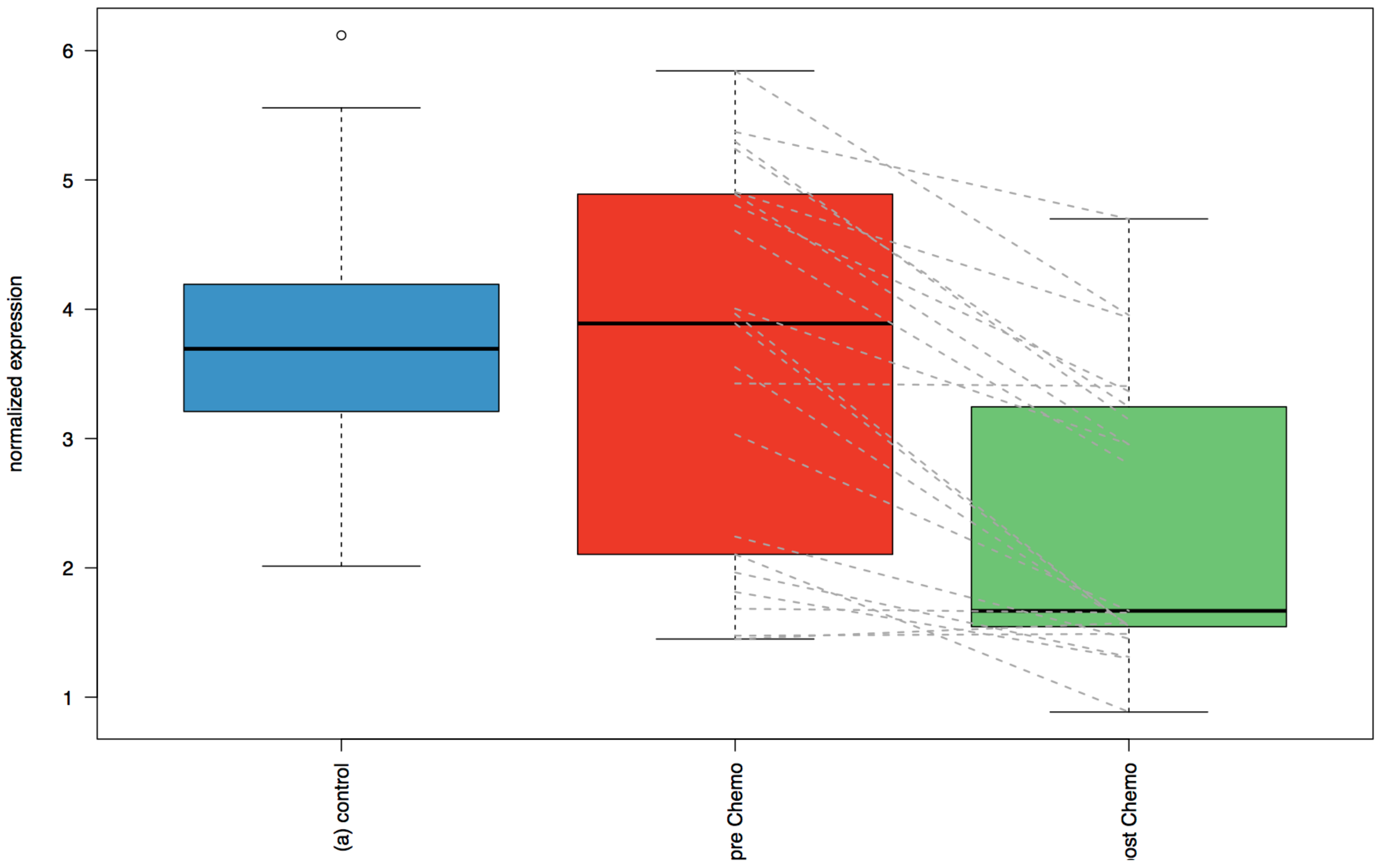
normalized expression
6
5
4
3
2
1
(a) control
pre Chemo
ost Chemo

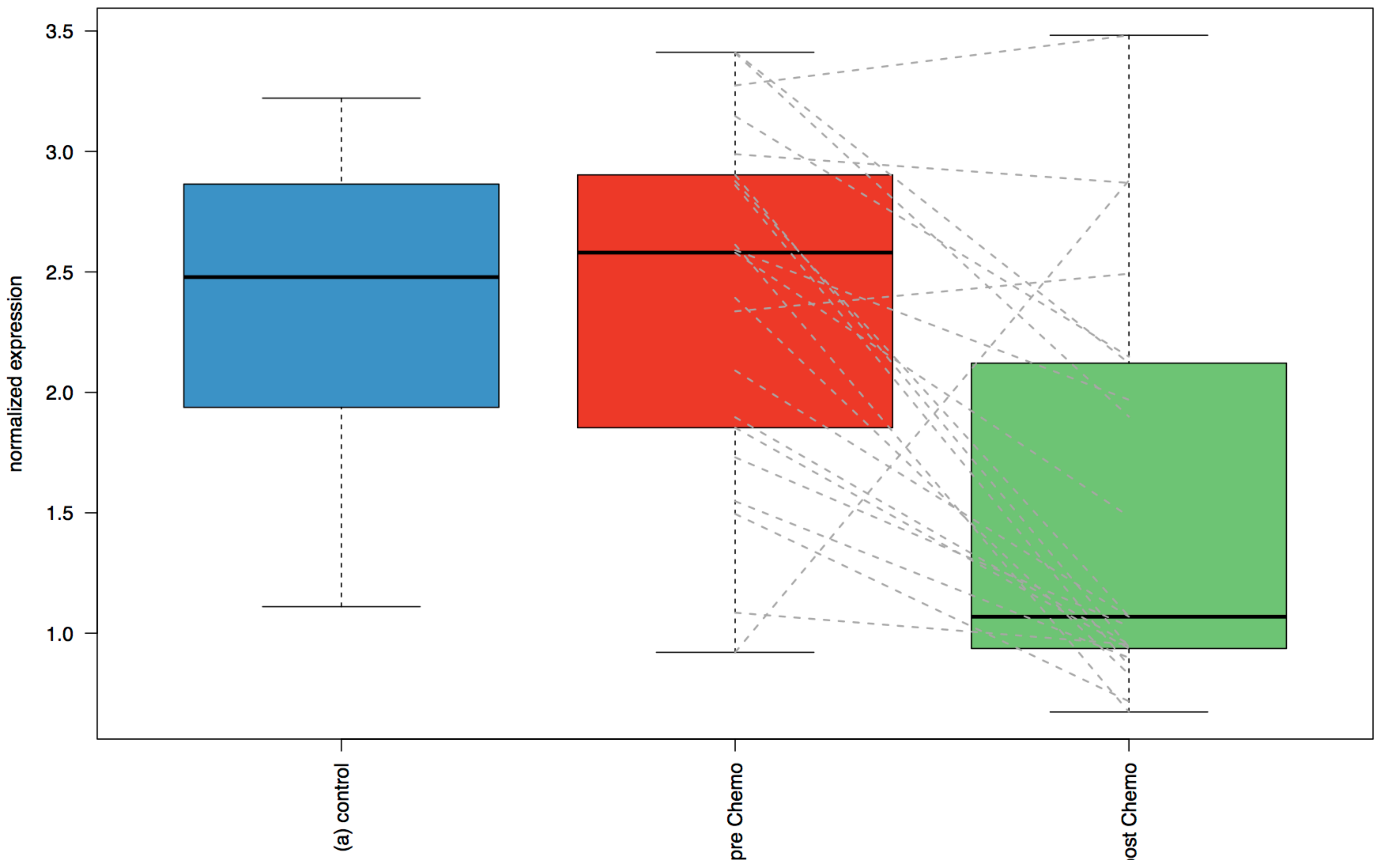
normalized expression
3.5
3.0
2.5
2.0
1.5
1.0
(a) control
pre Chemo
ost Chemo

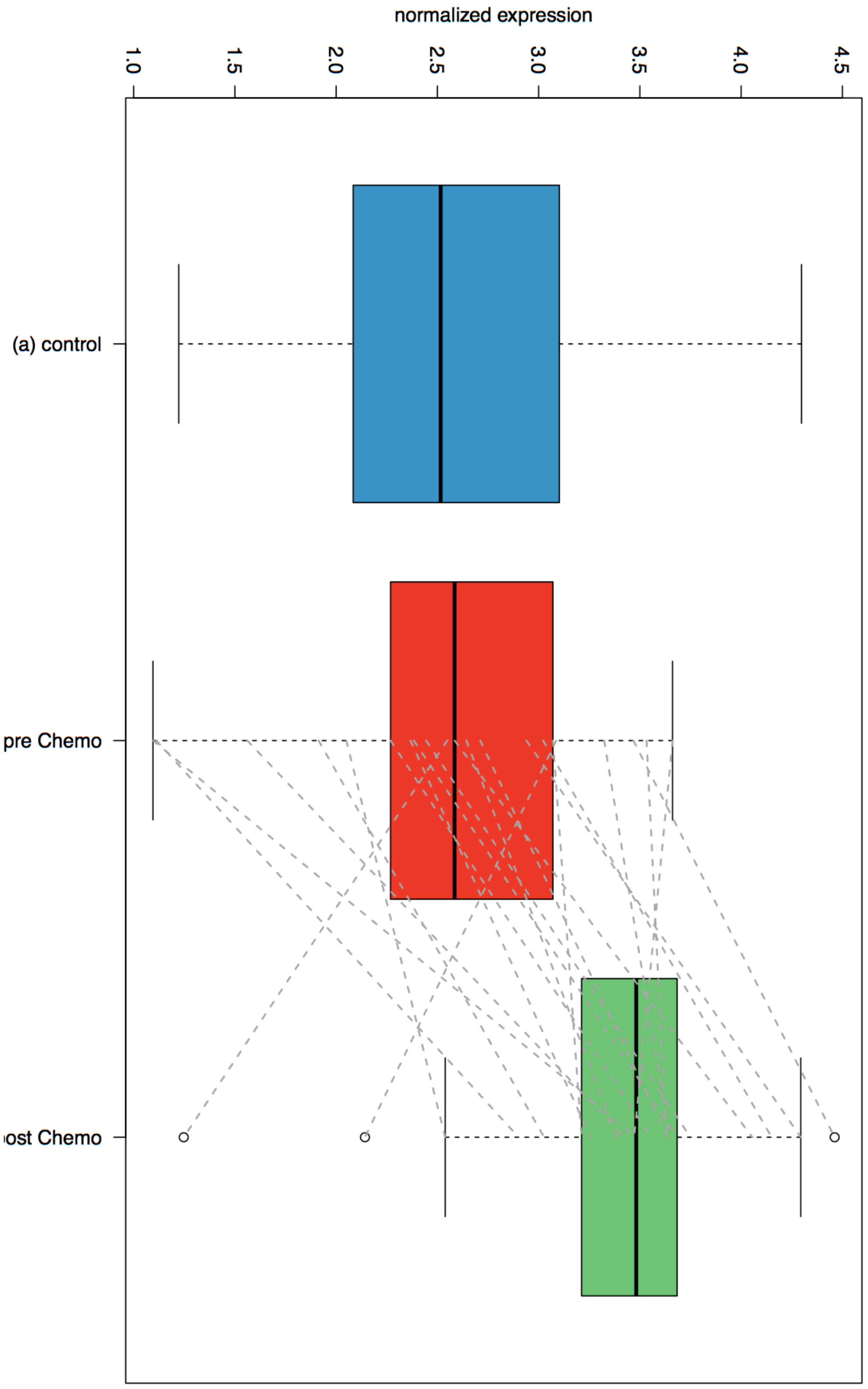
normalized expression
1.0
1.5
2.0
2.5
3.0
3.5
4.0
4.5
(a) control
pre Chemo
ost Chemo

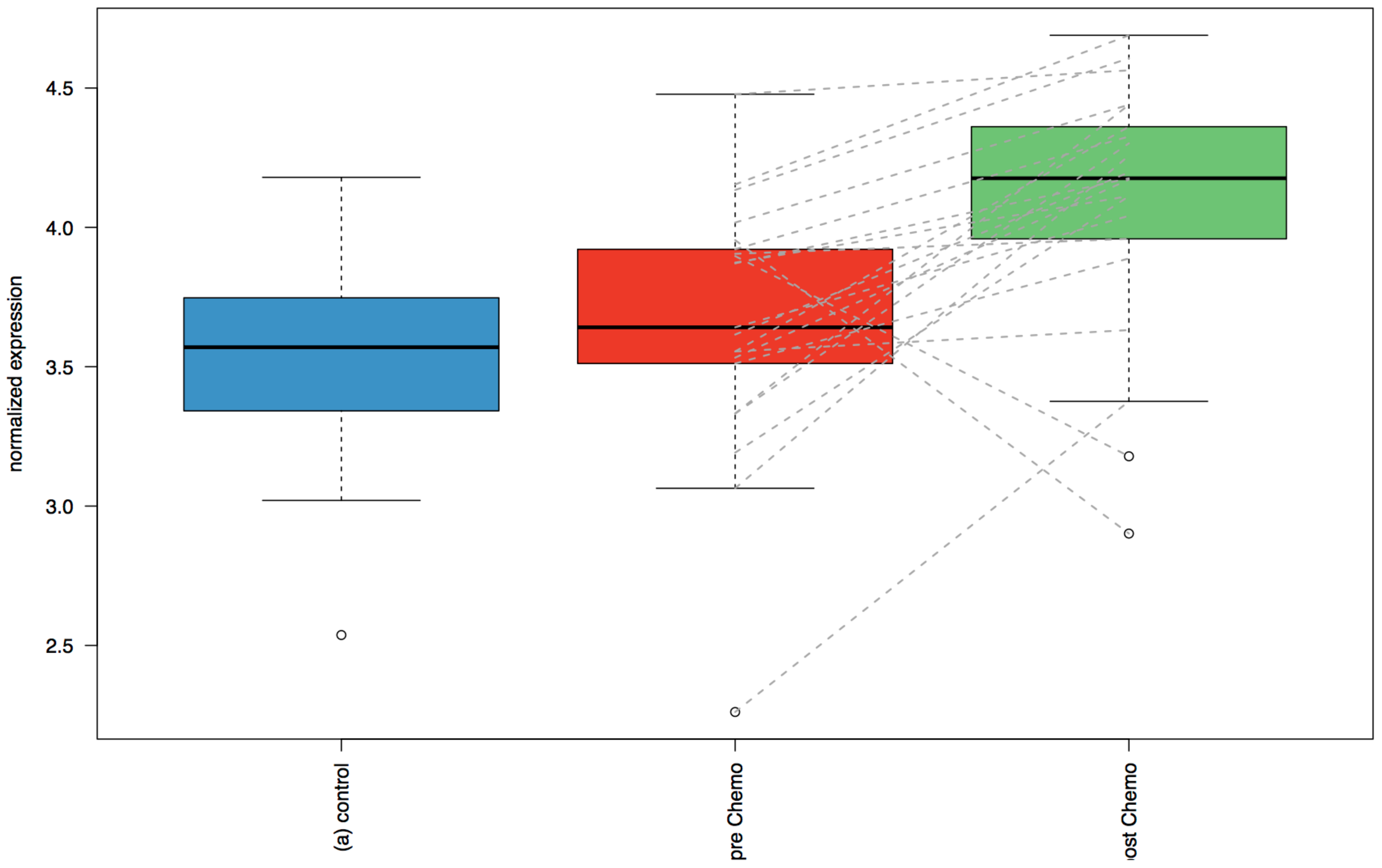
normalized expression
4.5
4.0
3.5
3.0
2.5
(a) control
pre Chemo
ost Chemo

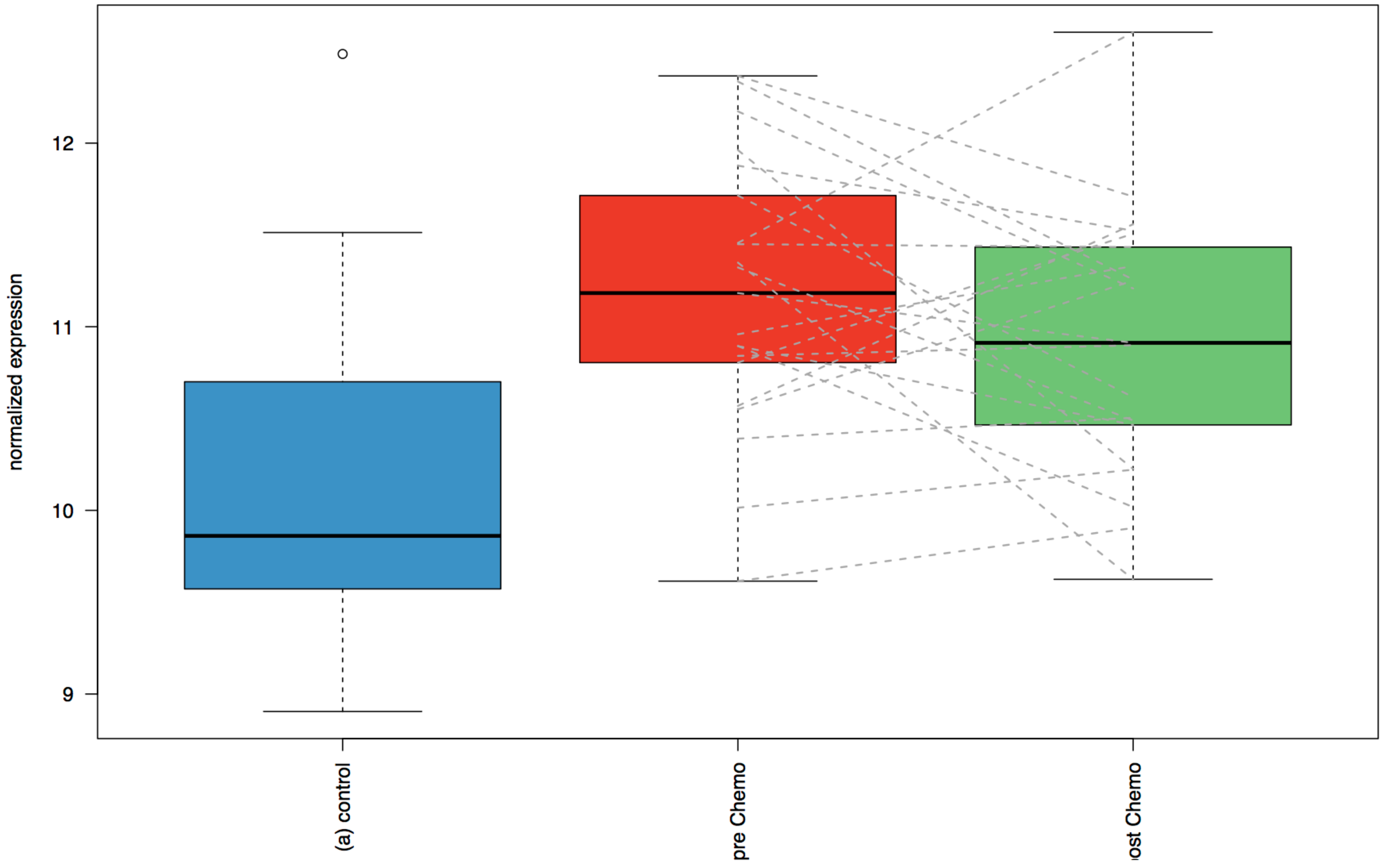
normalized expression
12
11
10
9
(a) control
pre Chemo
ost Chemo

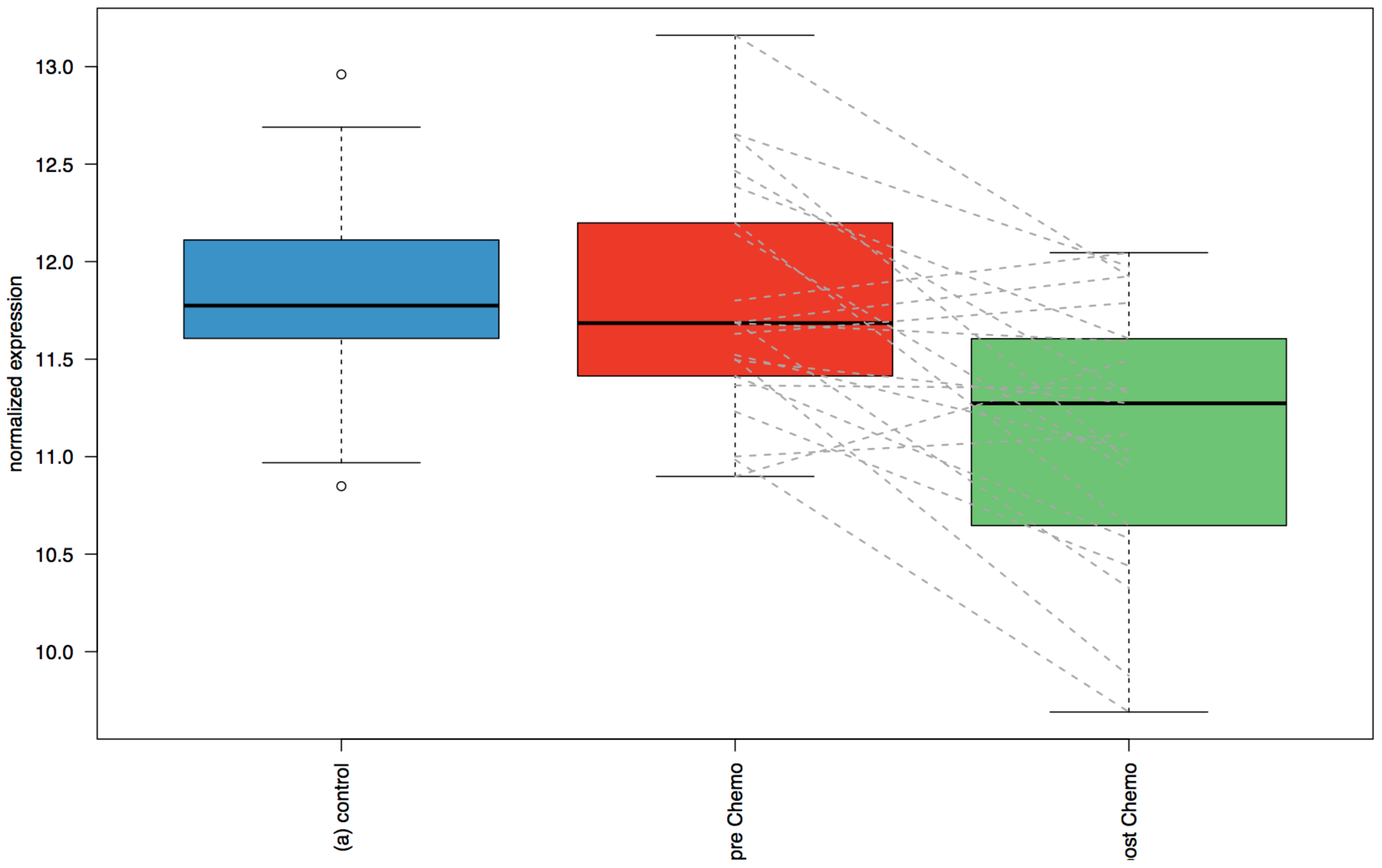
normalized expression
13.0
12.5
12.0
11.5
11.0
10.5
10.0
(a) control
pre Chemo
ost Chemo

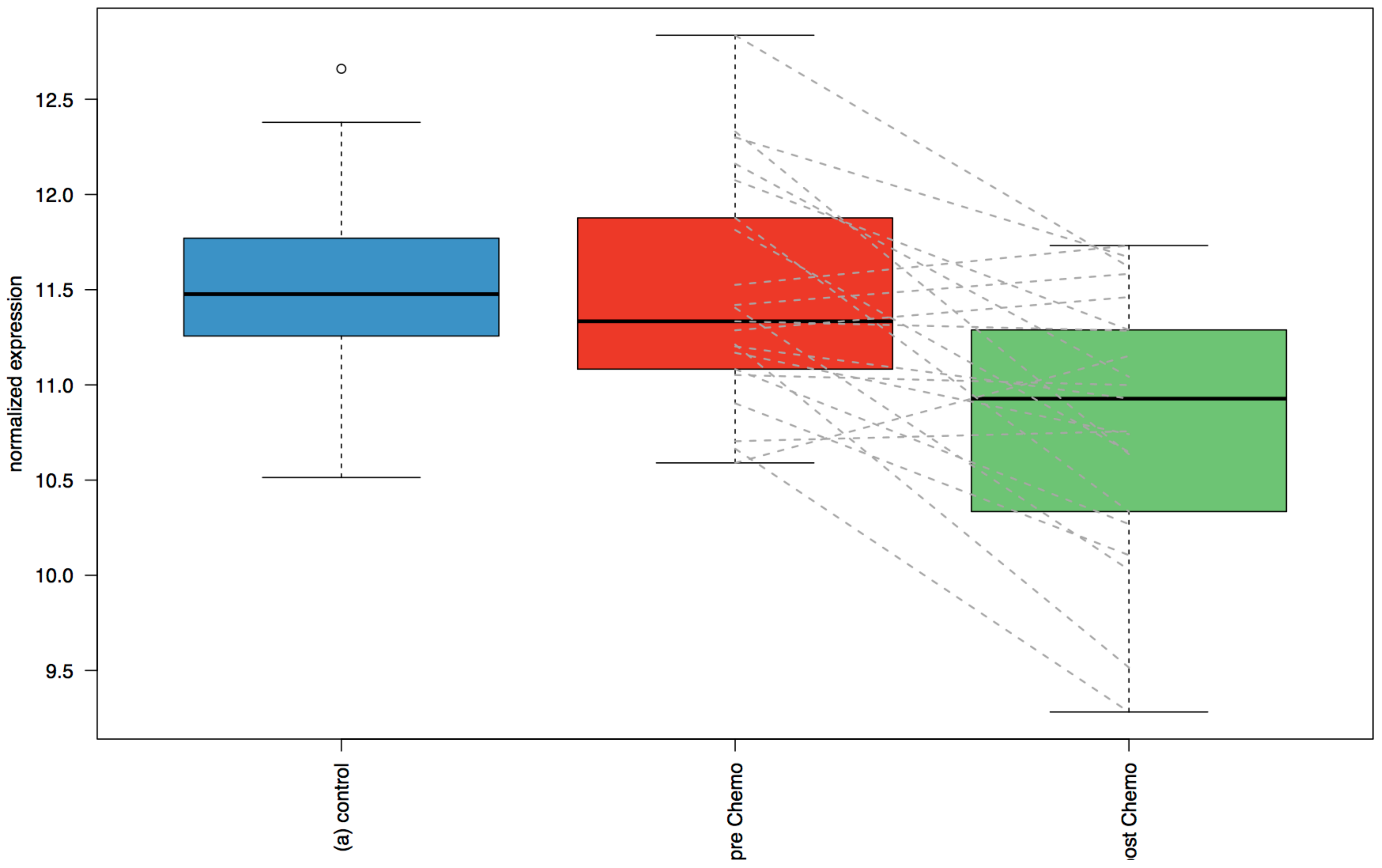

normalized expression
12.5
12.0
11.5
11.0
10.5
10.0
9.5
(a) control
pre Chemo
ost Chemo

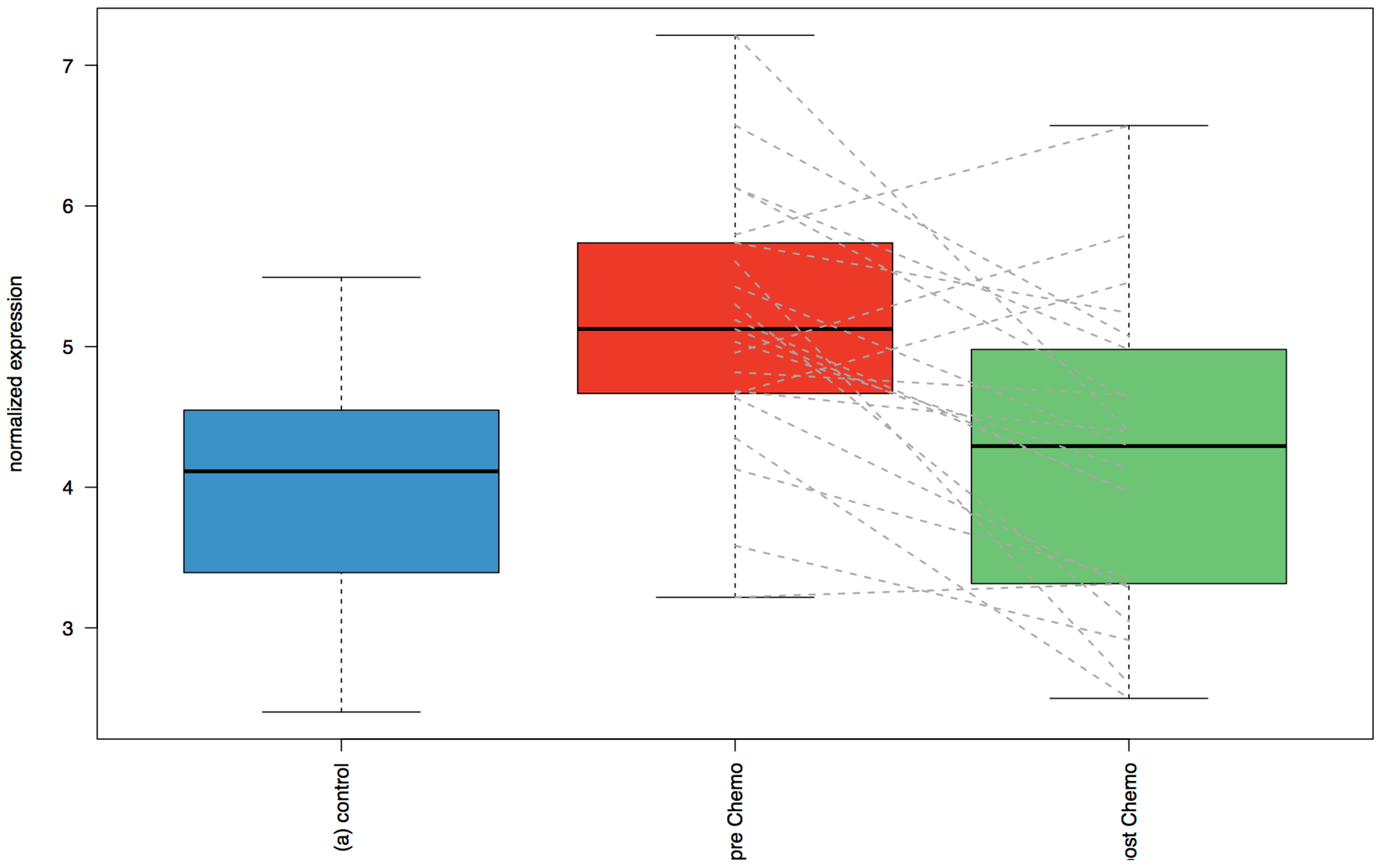
normalized expression
7
6
5
4
3
(a) control
pre Chemo
ost Chemo

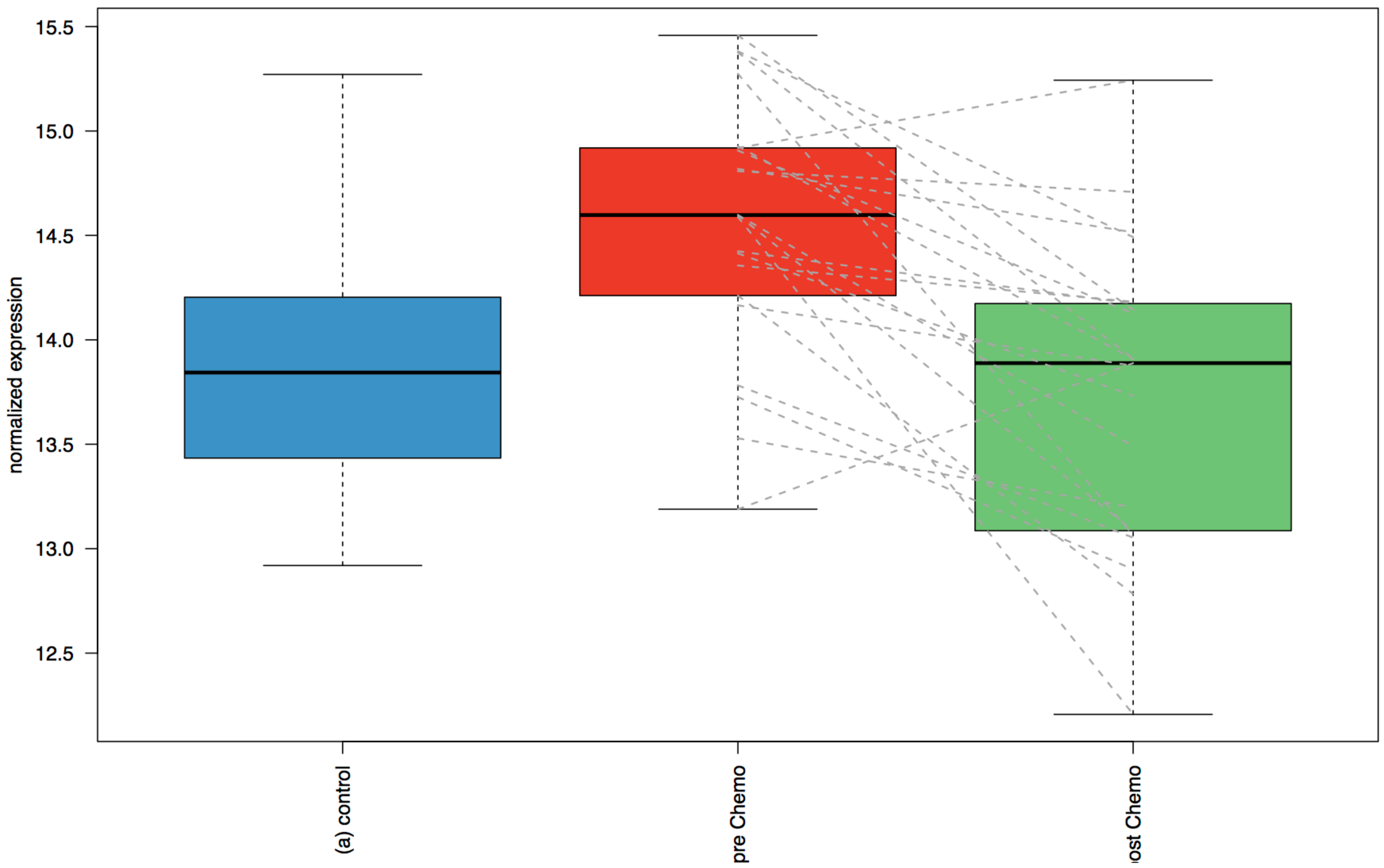
normalized expression
15.5
15.0
14.5
14.0
13.5
13.0
12.5
(a) control
pre Chemo
ost Chemo

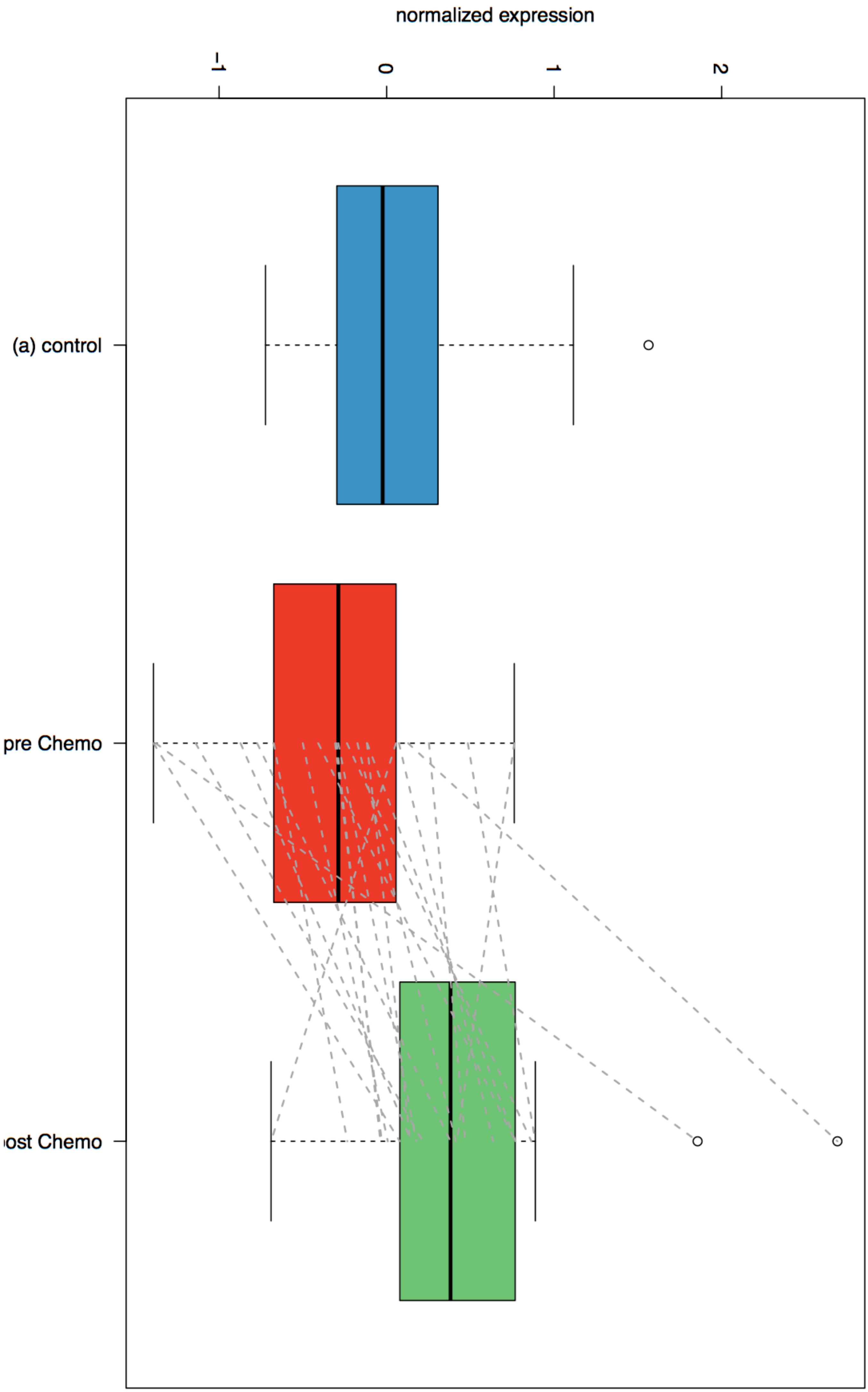
normalized expression
−1
0
1
2
(a) control
pre Chemo
ost Chemo

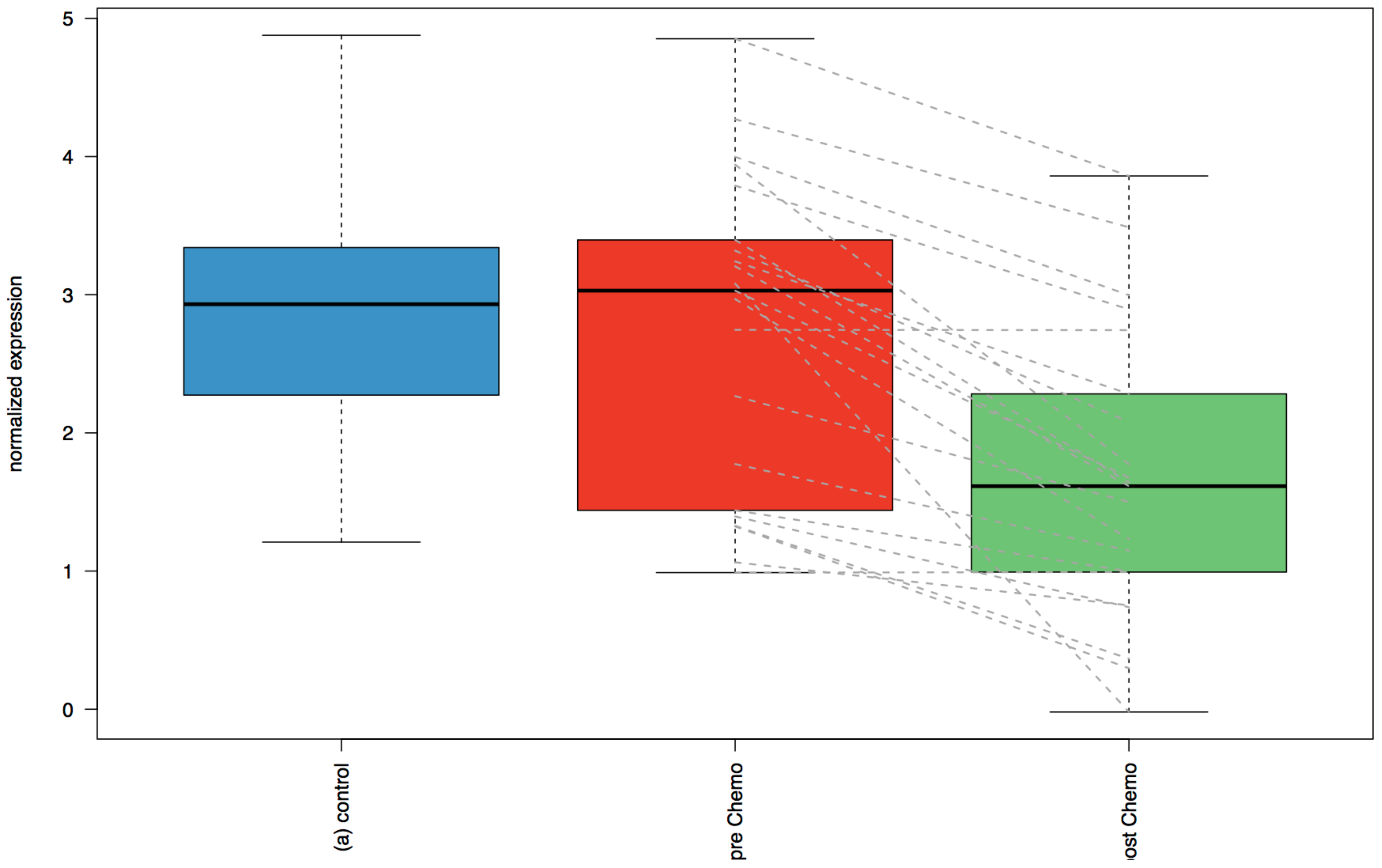
normalized expression
5
4
3
2
1
0
(a) control
pre Chemo
ost Chemo

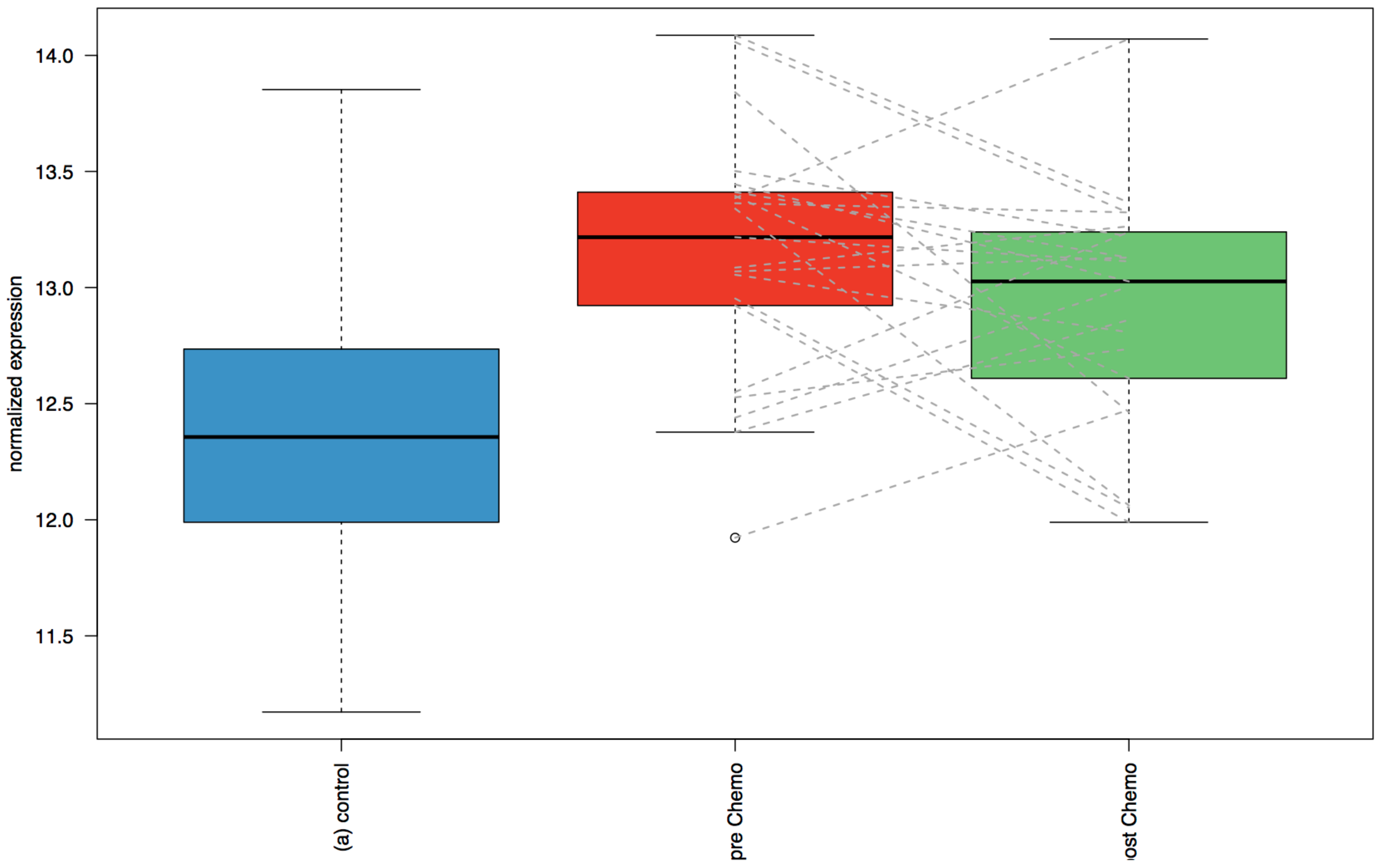
normalized expression
14.0
13.5
13.0
12.5
12.0
11.5
(a) control
pre Chemo
ost Chemo

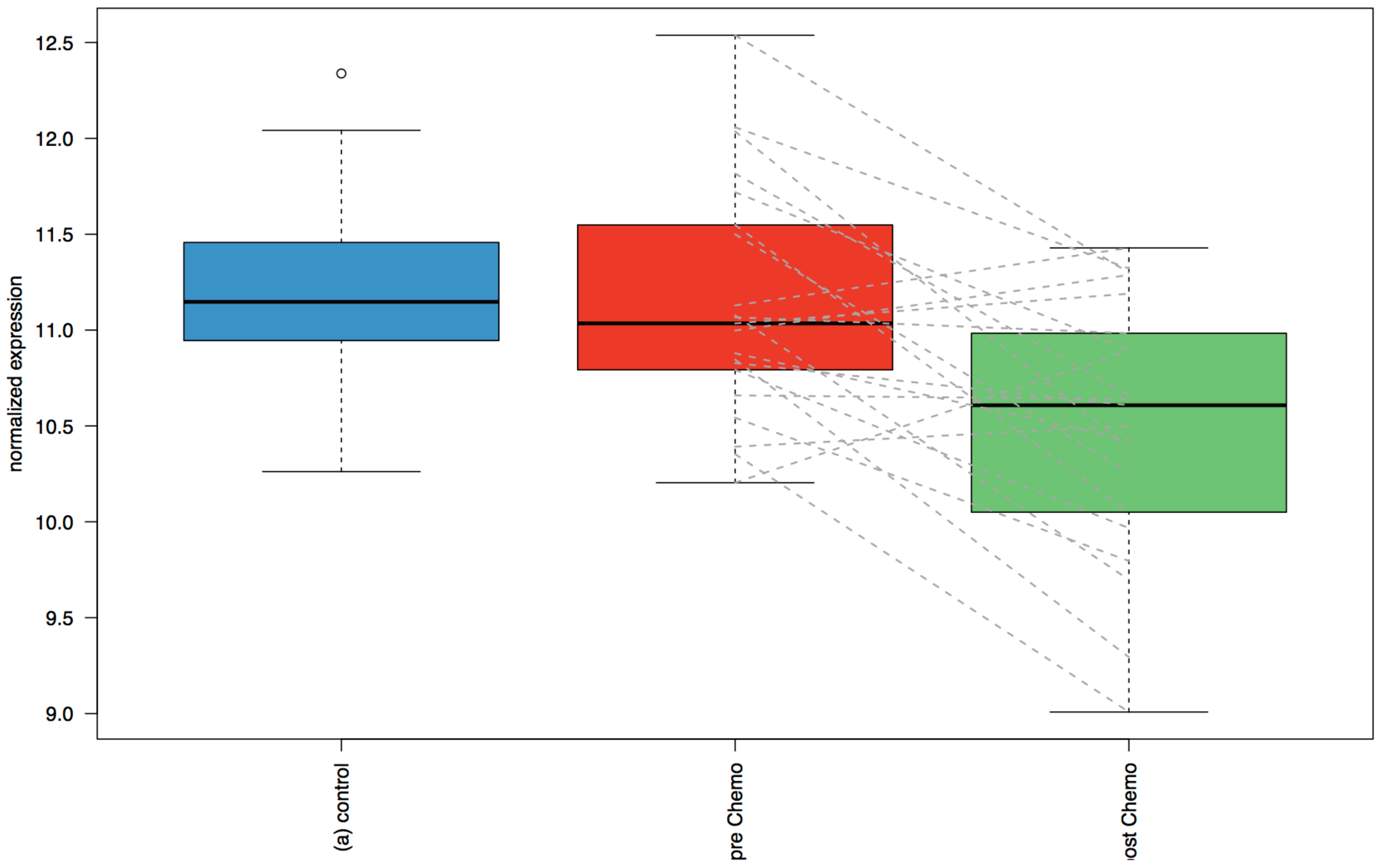
normalized expression
12.5
12.0
11.5
11.0
10.5
10.0
9.5
9.0
(a) control
pre Chemo
ost Chemo

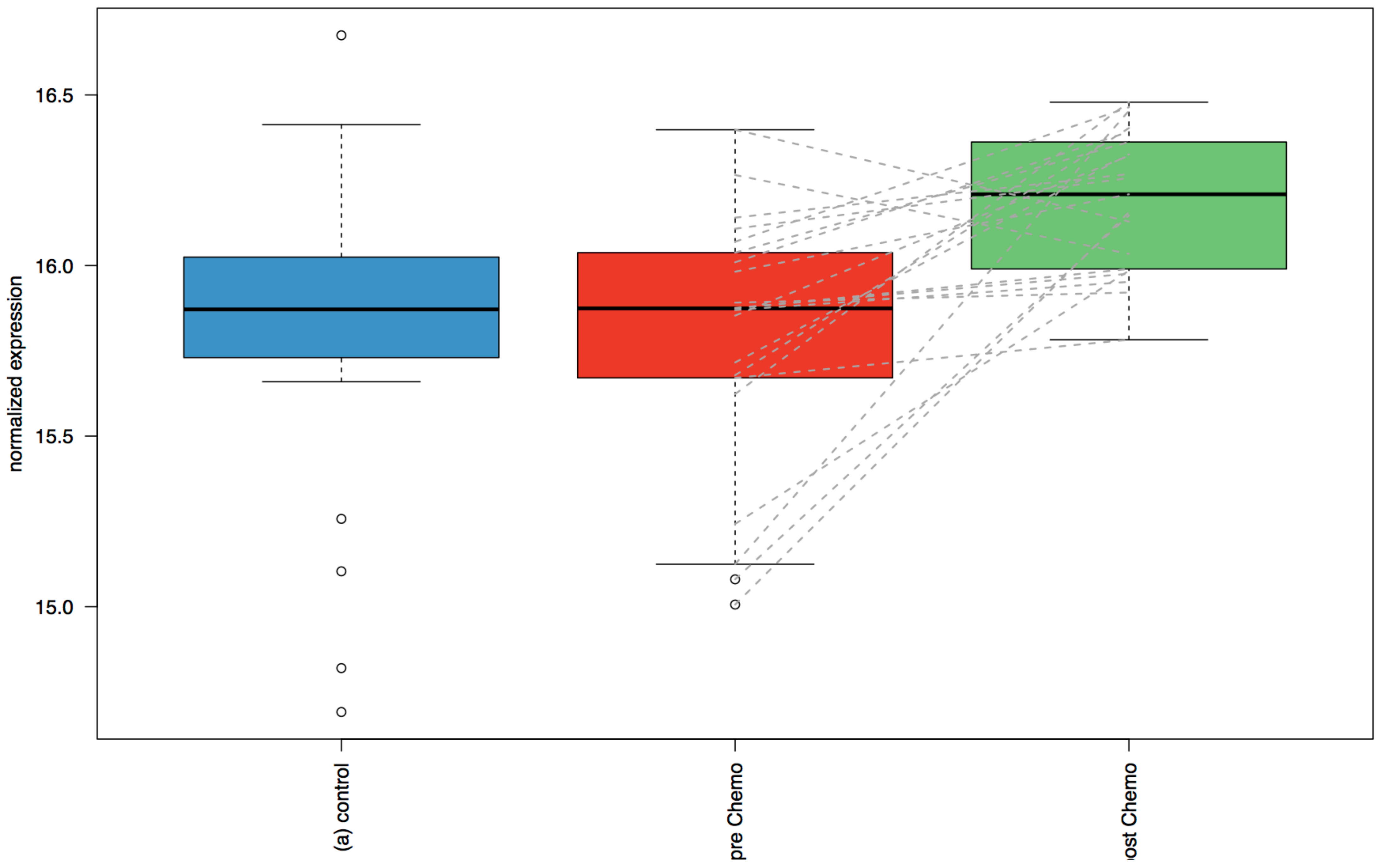
normalized expression
16.5
16.0
15.5
15.0
(a) control
pre Chemo
ost Chemo

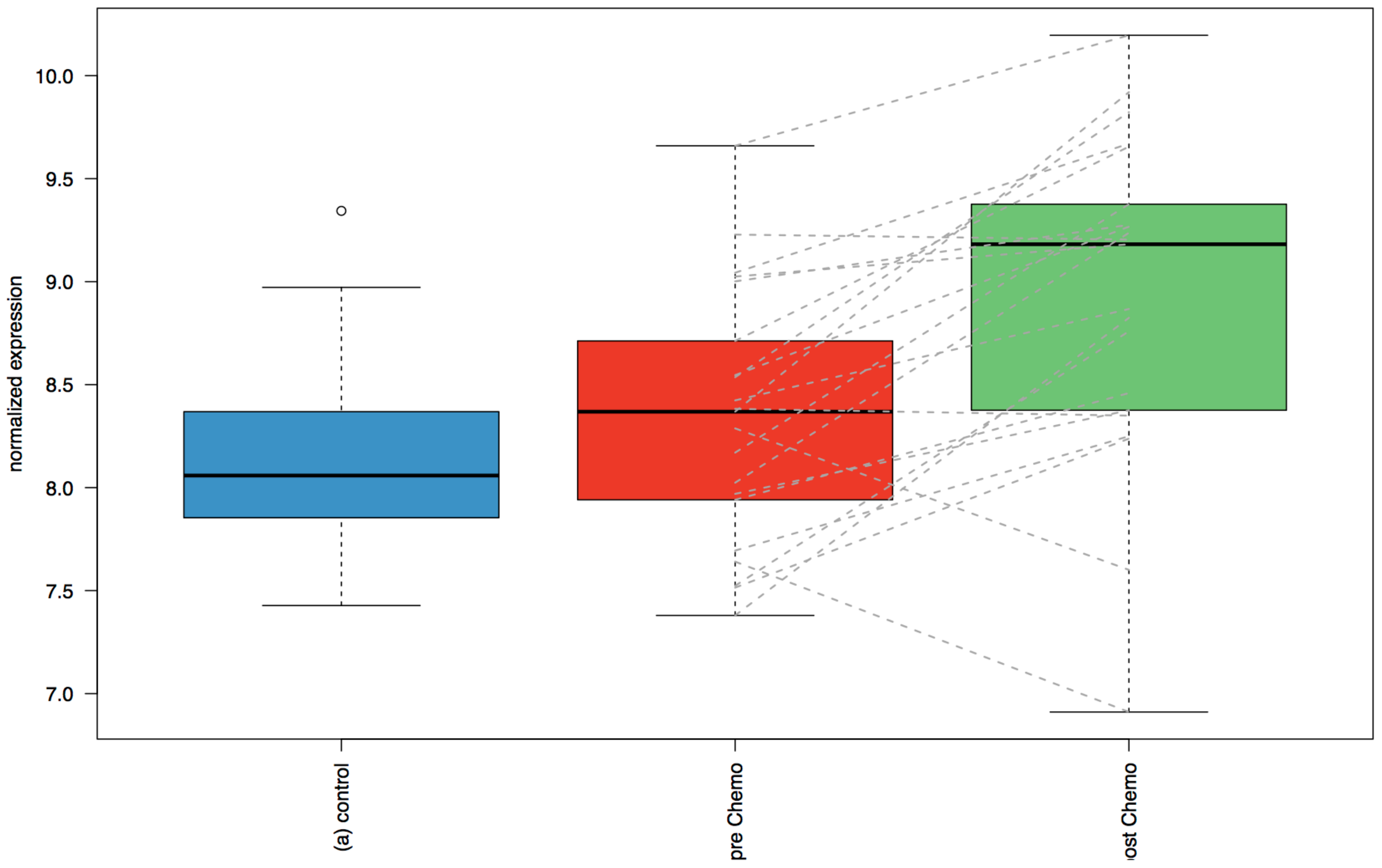
normalized expression
10.0
9.5
9.0
8.5
8.0
7.5
7.0
(a) control
pre Chemo
ost Chemo

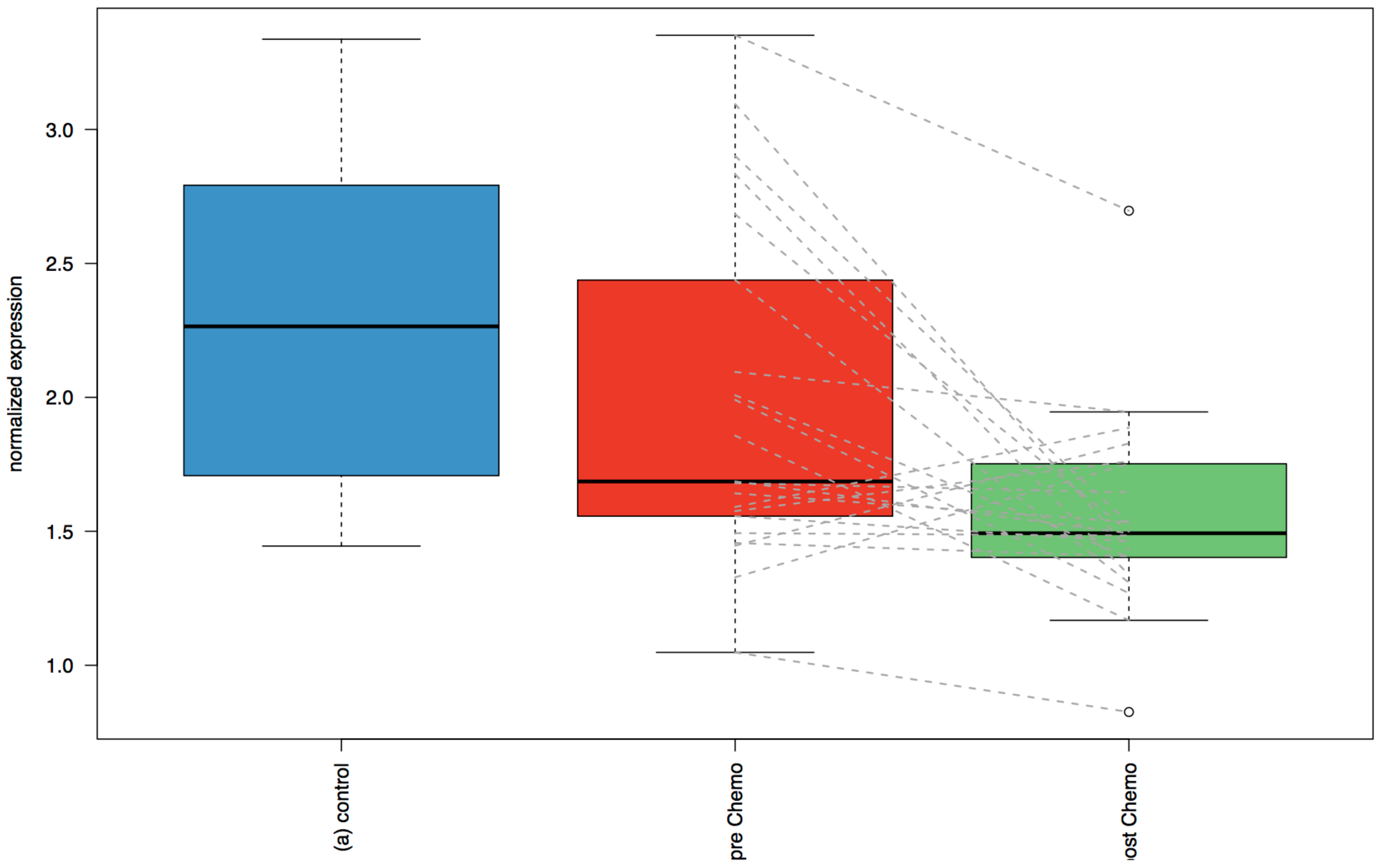
normalized expression
3.0
2.5
2.0
1.5
1.0
(a) control
pre Chemo
ost Chemo

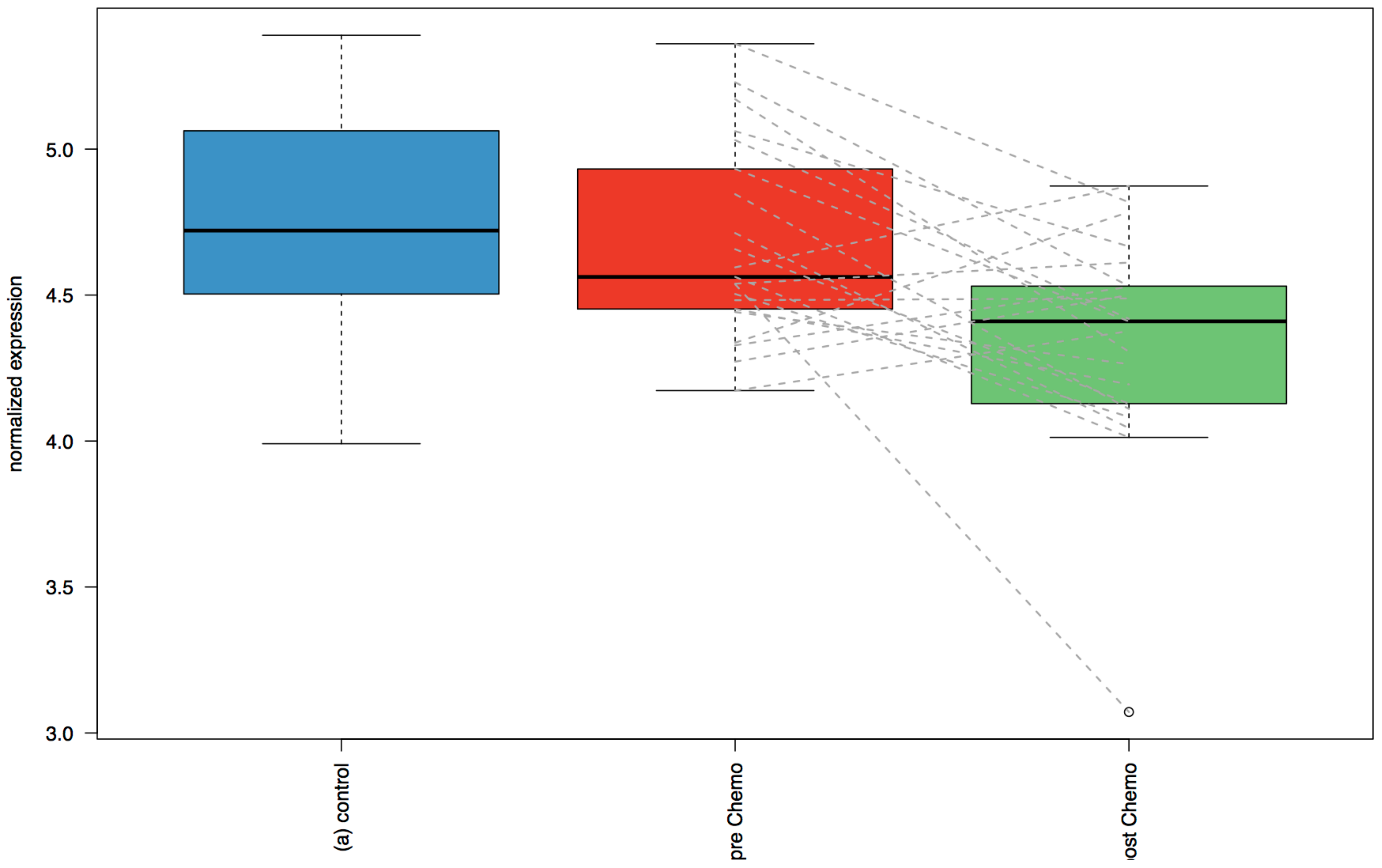
normalized expression
5.0
4.5
4.0
3.5
3.0
(a) control
pre Chemo
ost Chemo

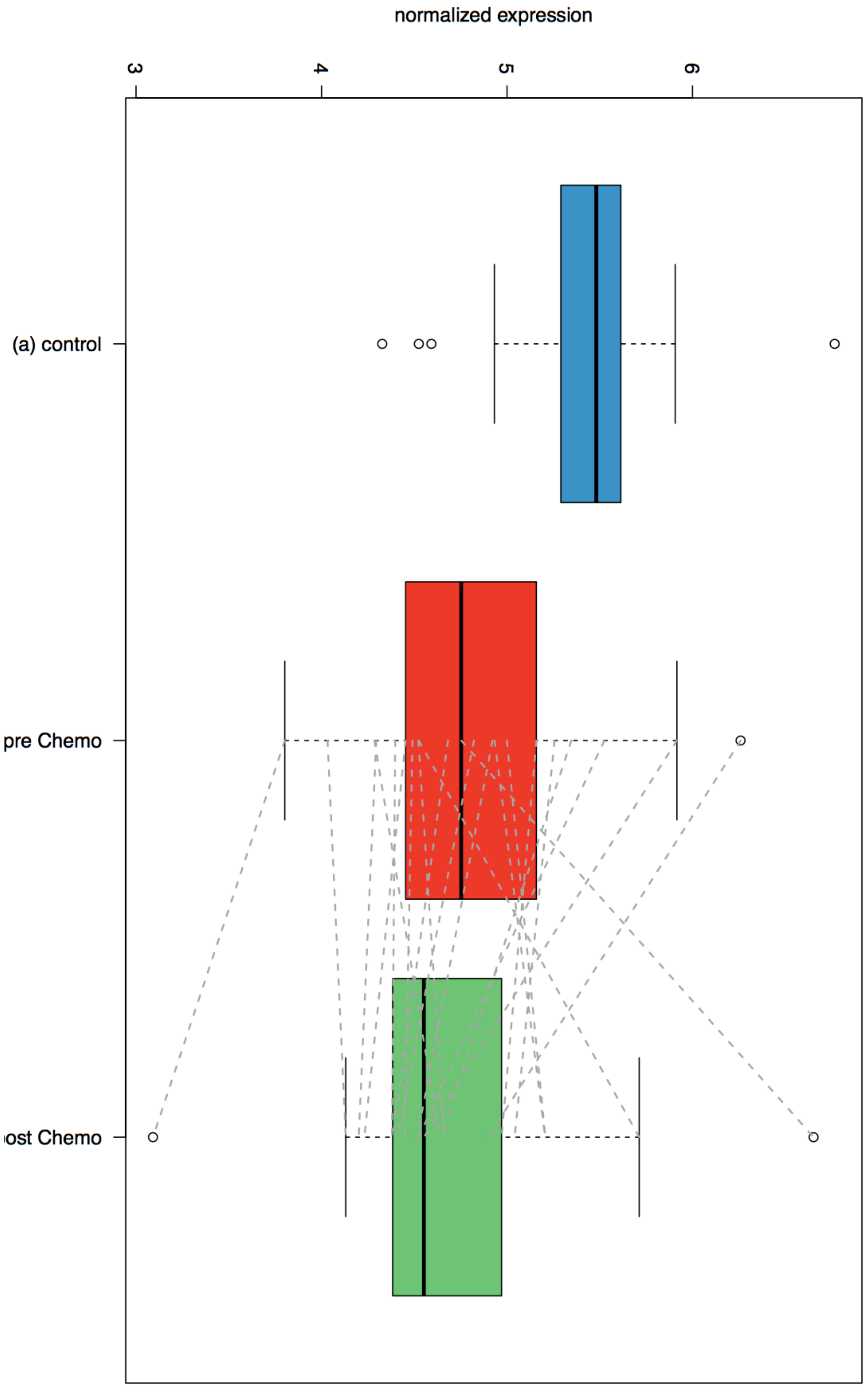
normalized expression
3
4
5
6
(a) control
pre Chemo
ost Chemo

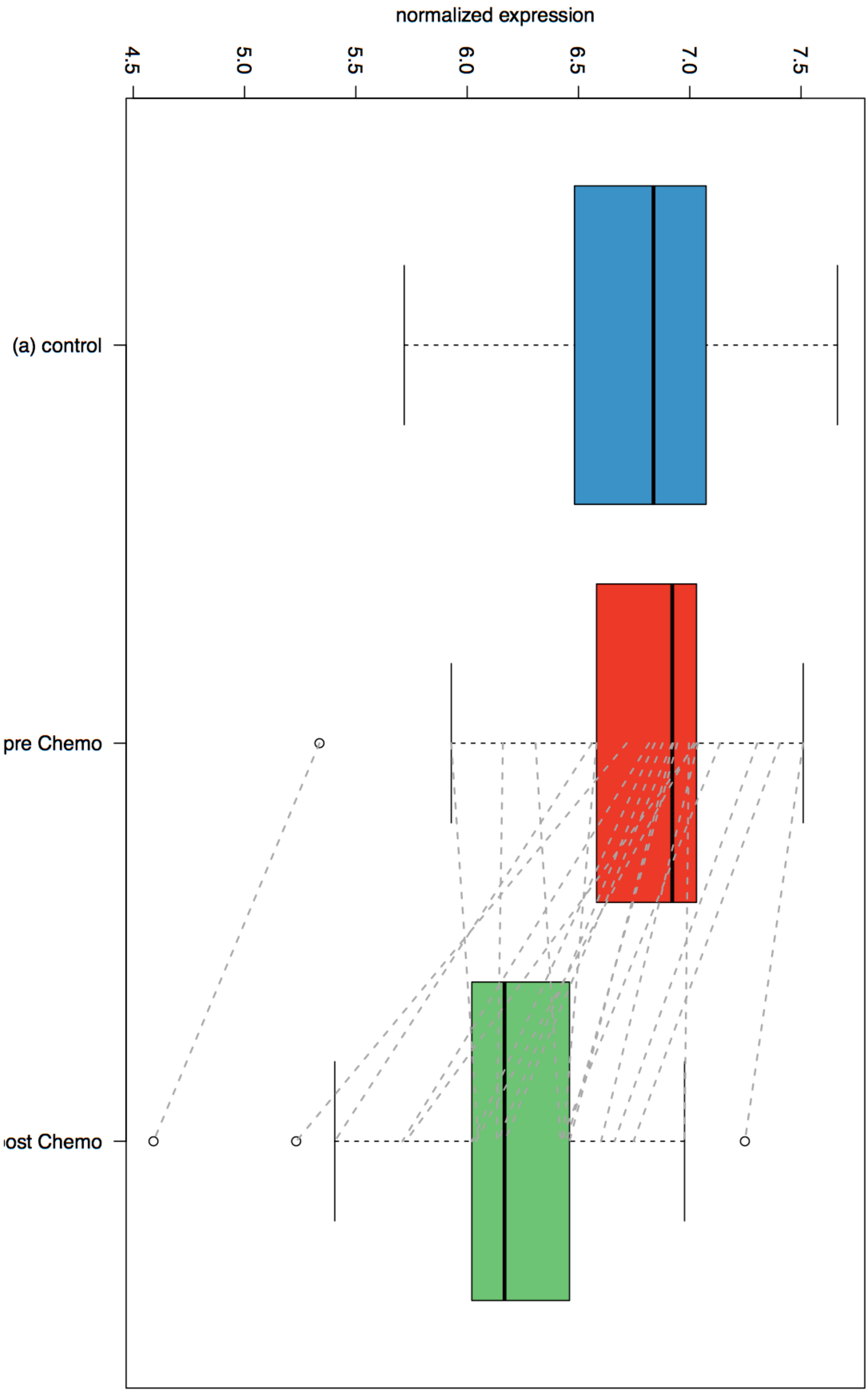

normalized expression
4.5
5.0
5.5
6.0
6.5
7.0
7.5
(a) control
pre Chemo
ost Chemo

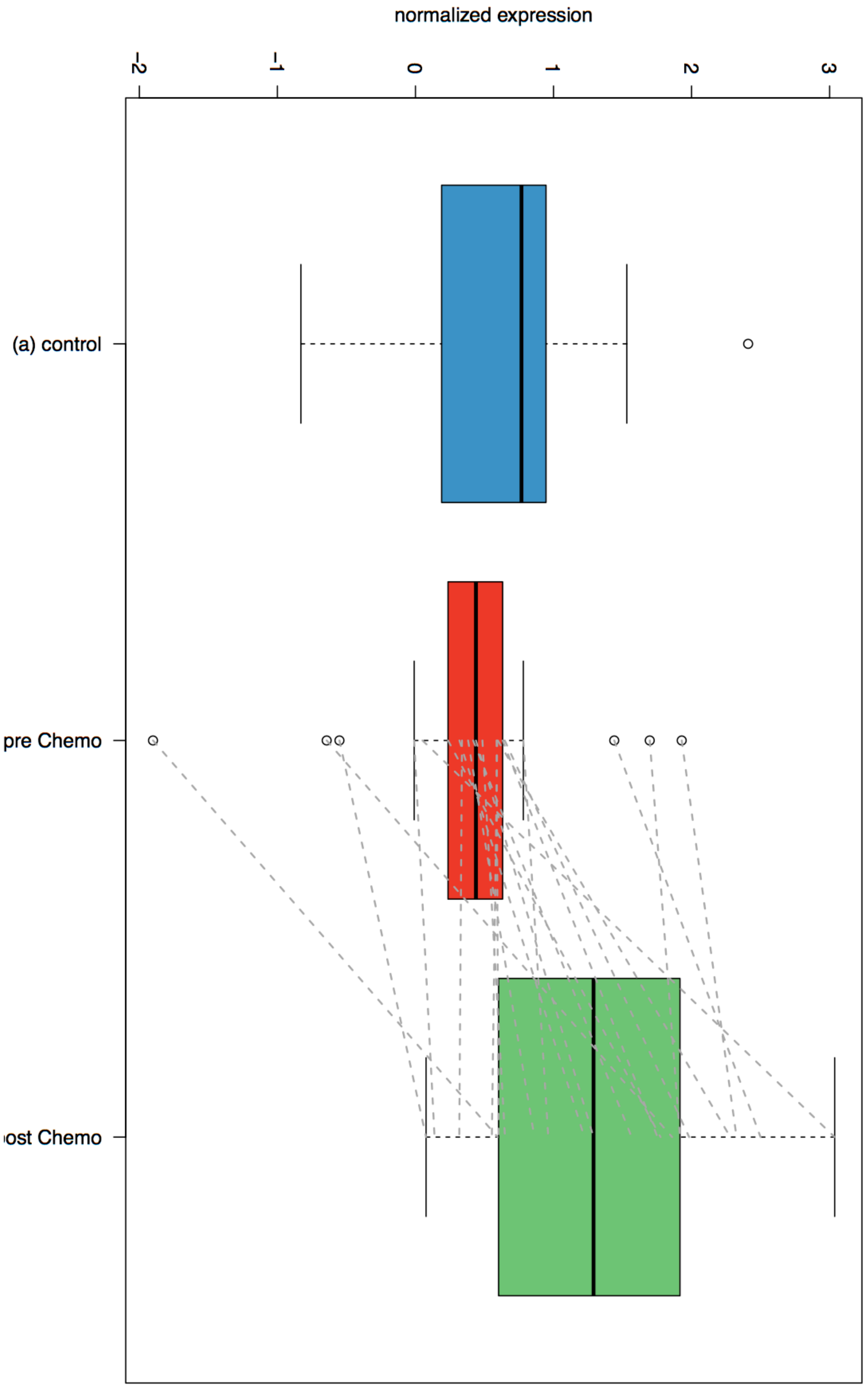
normalized expression
-2
-1
0
1
2
3
(a) control
pre Chemo
post Chemo

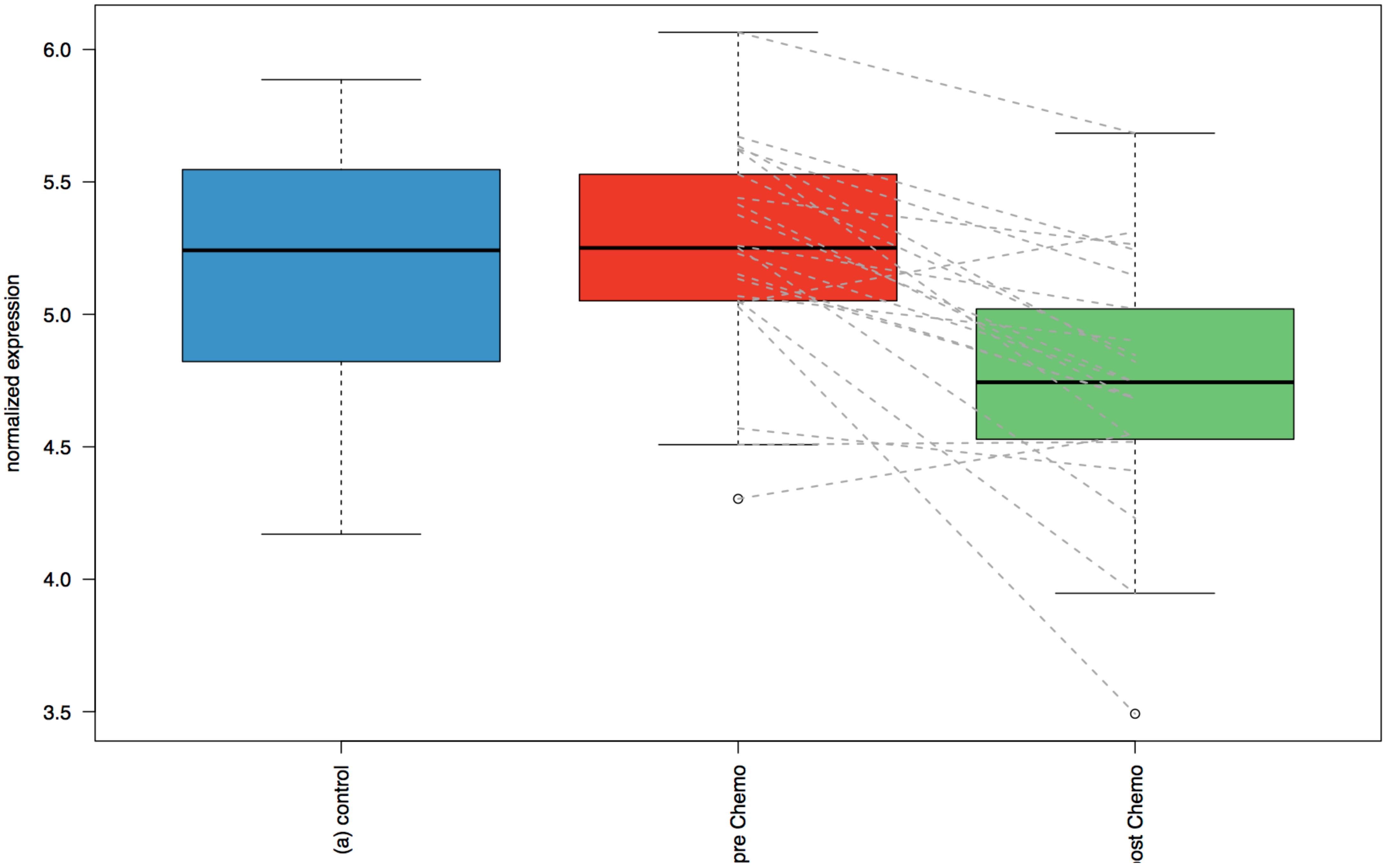
normalized expression
6.0
5.5
5.0
4.5
4.0
3.5
(a) control
pre Chemo
ost Chemo

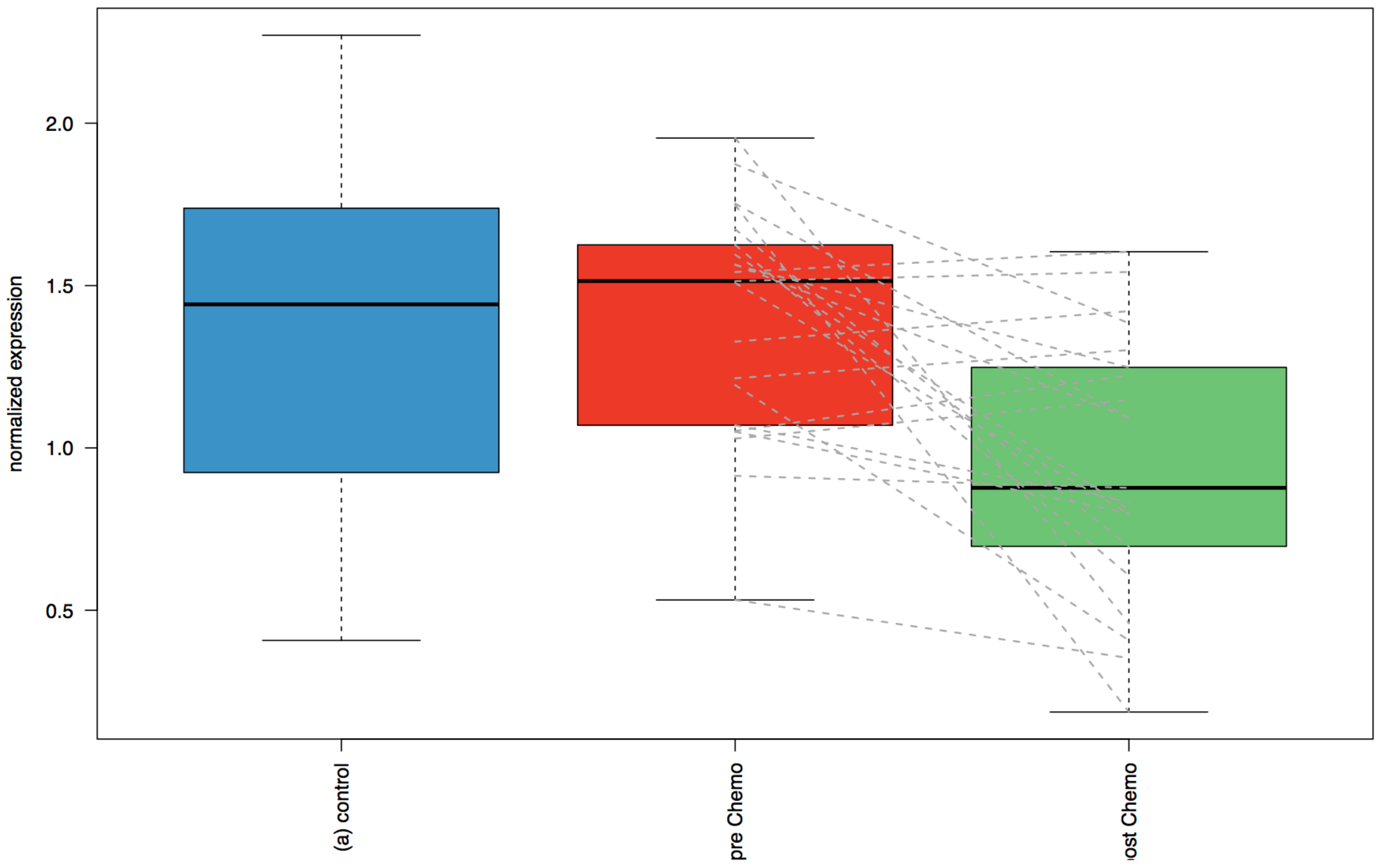

normalized expression
2.0
1.5
1.0
0.5
(a) control
pre Chemo
ost Chemo

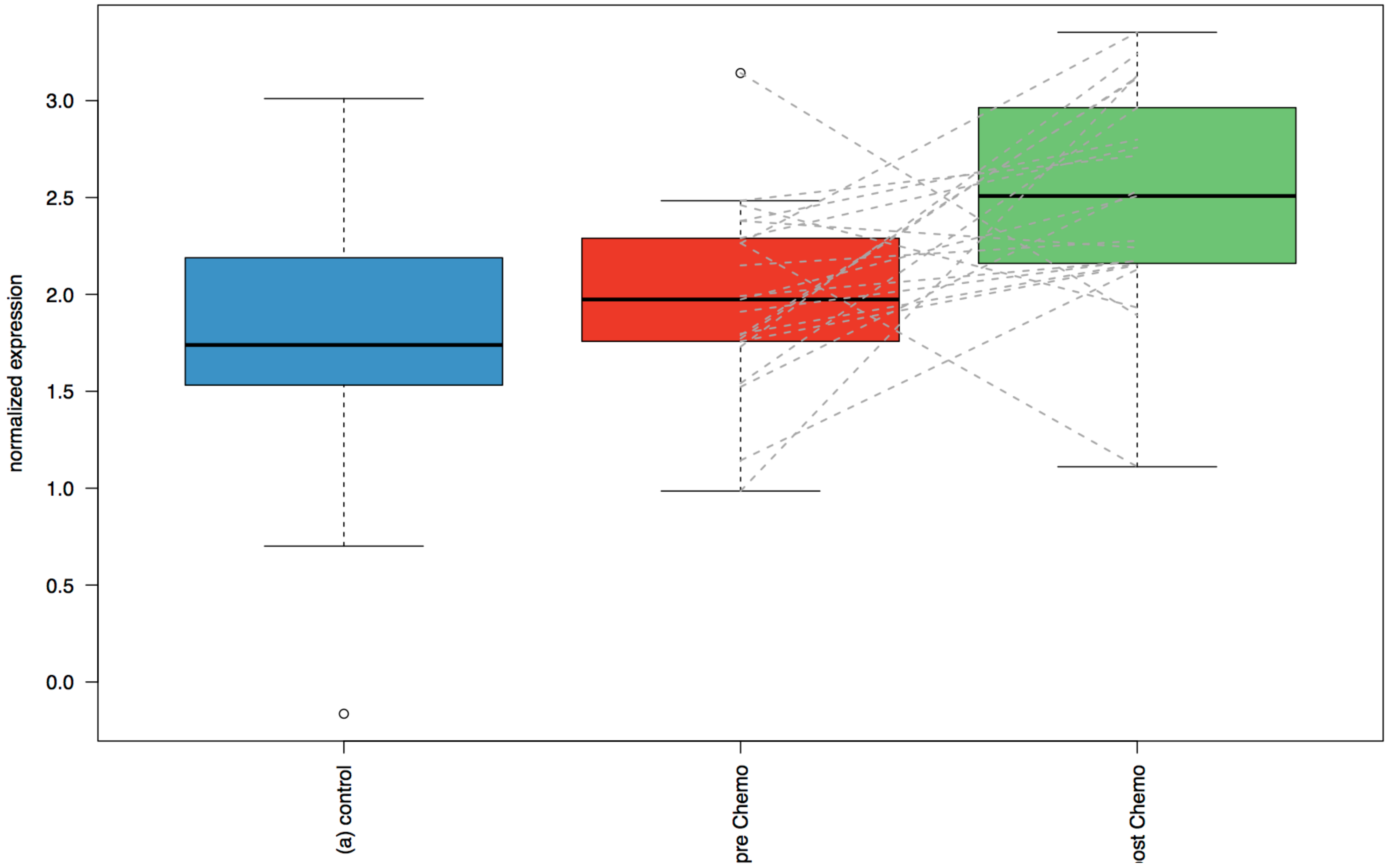
normalized expression
3.0
2.5
2.0
1.5
1.0
0.5
0.0
(a) control
pre Chemo
ost Chemo

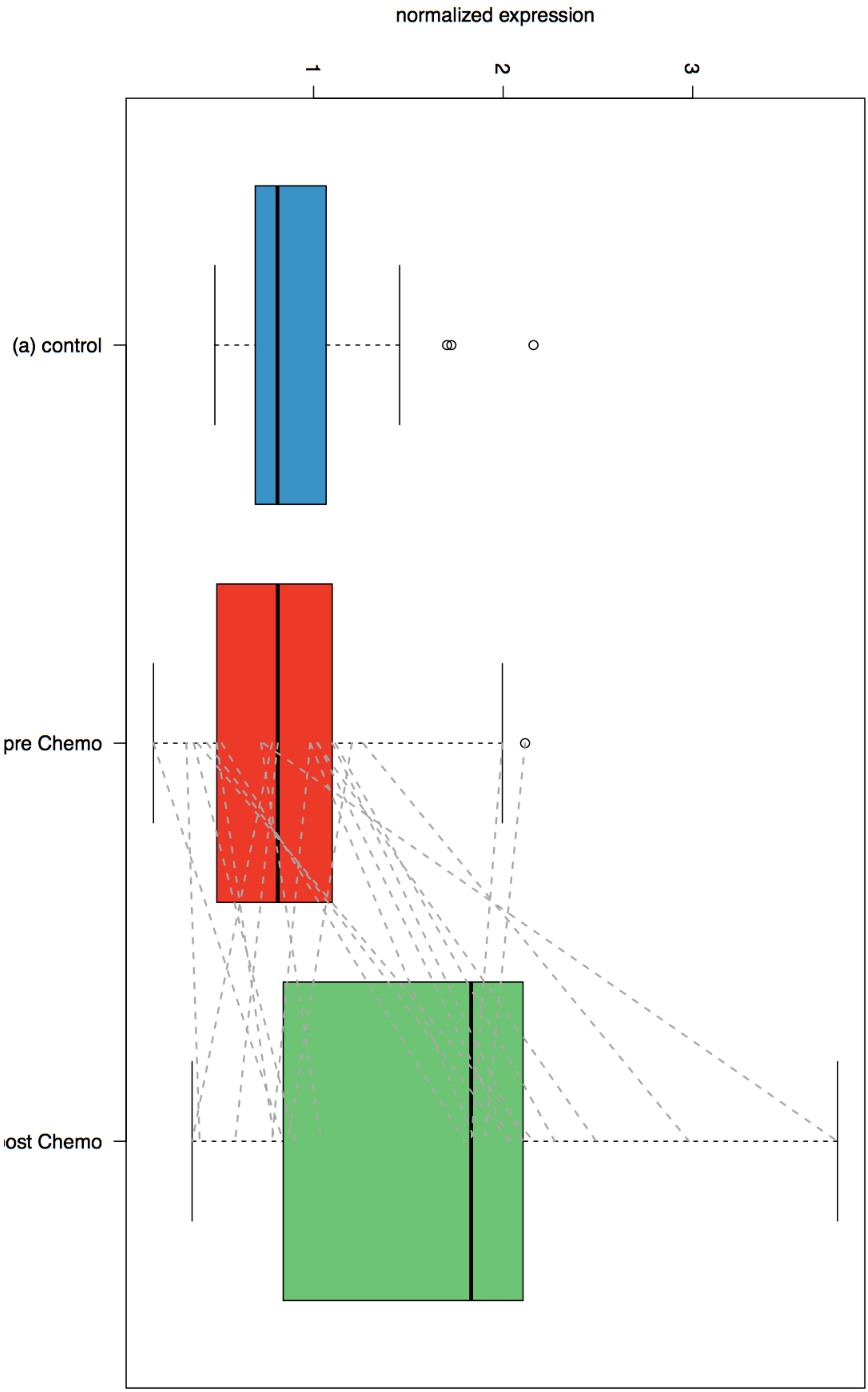
normalized expression
1
2
3
(a) control
pre Chemo
ost Chemo

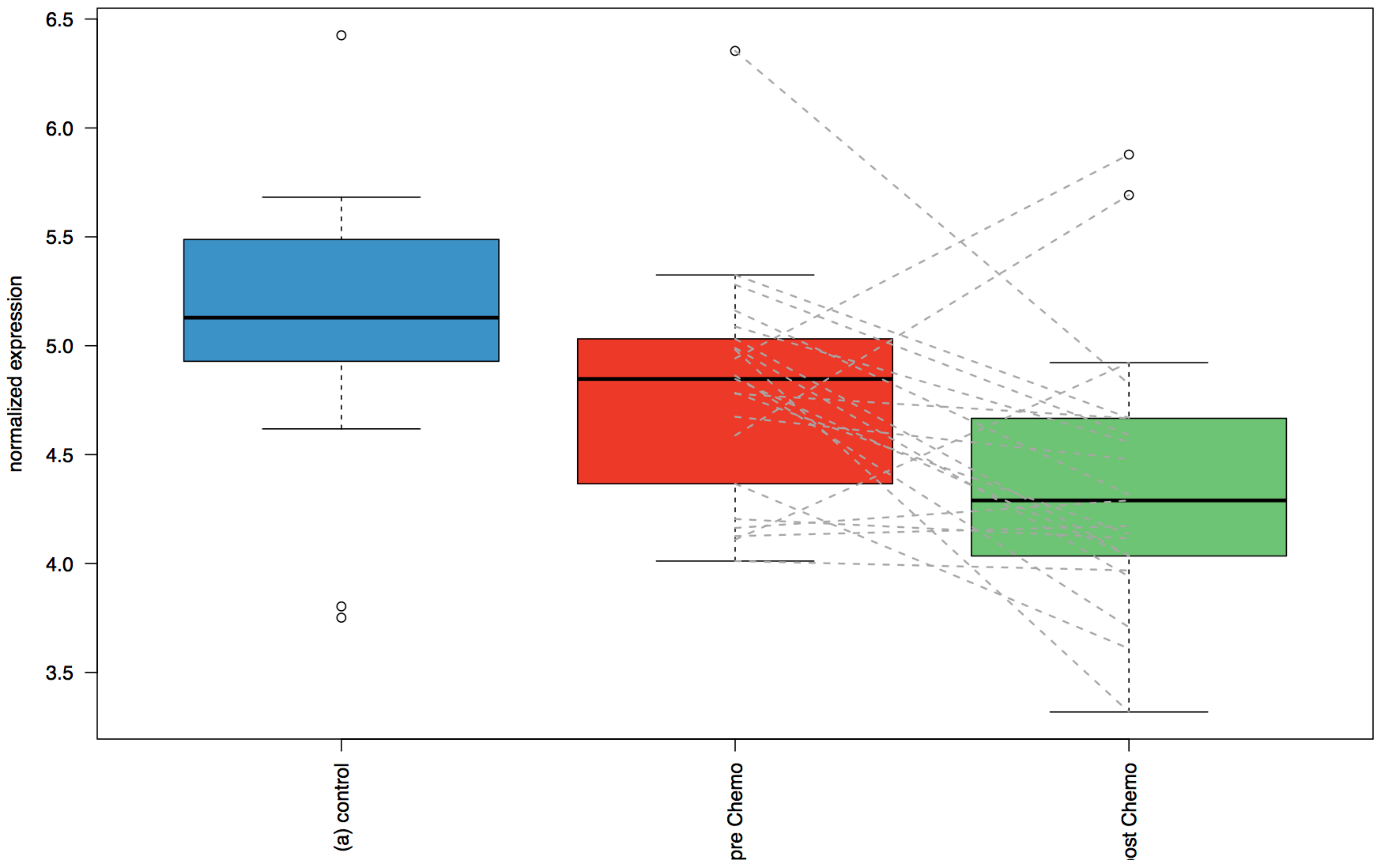
normalized expression
6.5
6.0
5.5
5.0
4.5
4.0
3.5
(a) control
pre Chemo
ost Chemo

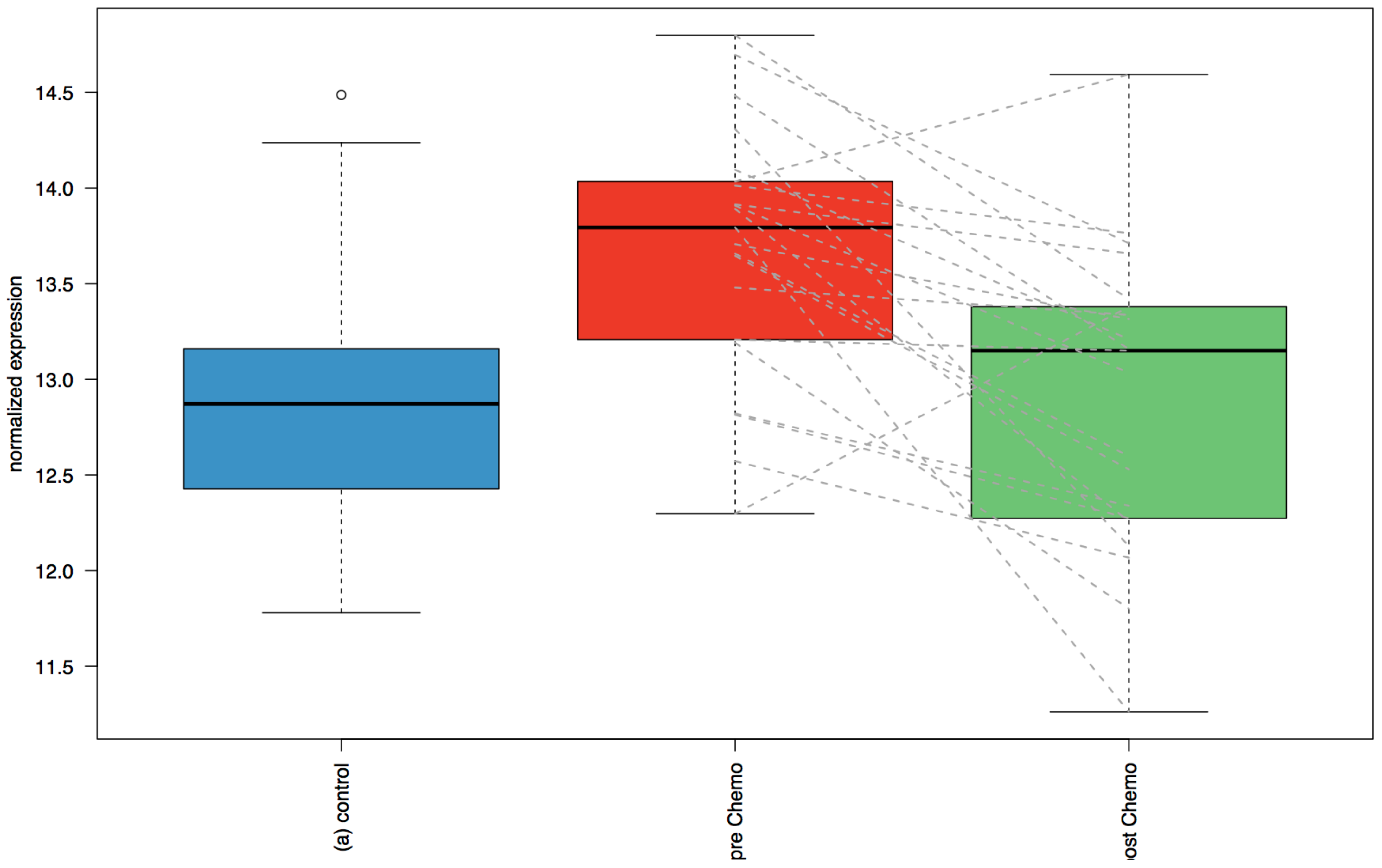
normalized expression
14.5
14.0
13.5
13.0
12.5
12.0
11.5
(a) control
pre Chemo
ost Chemo

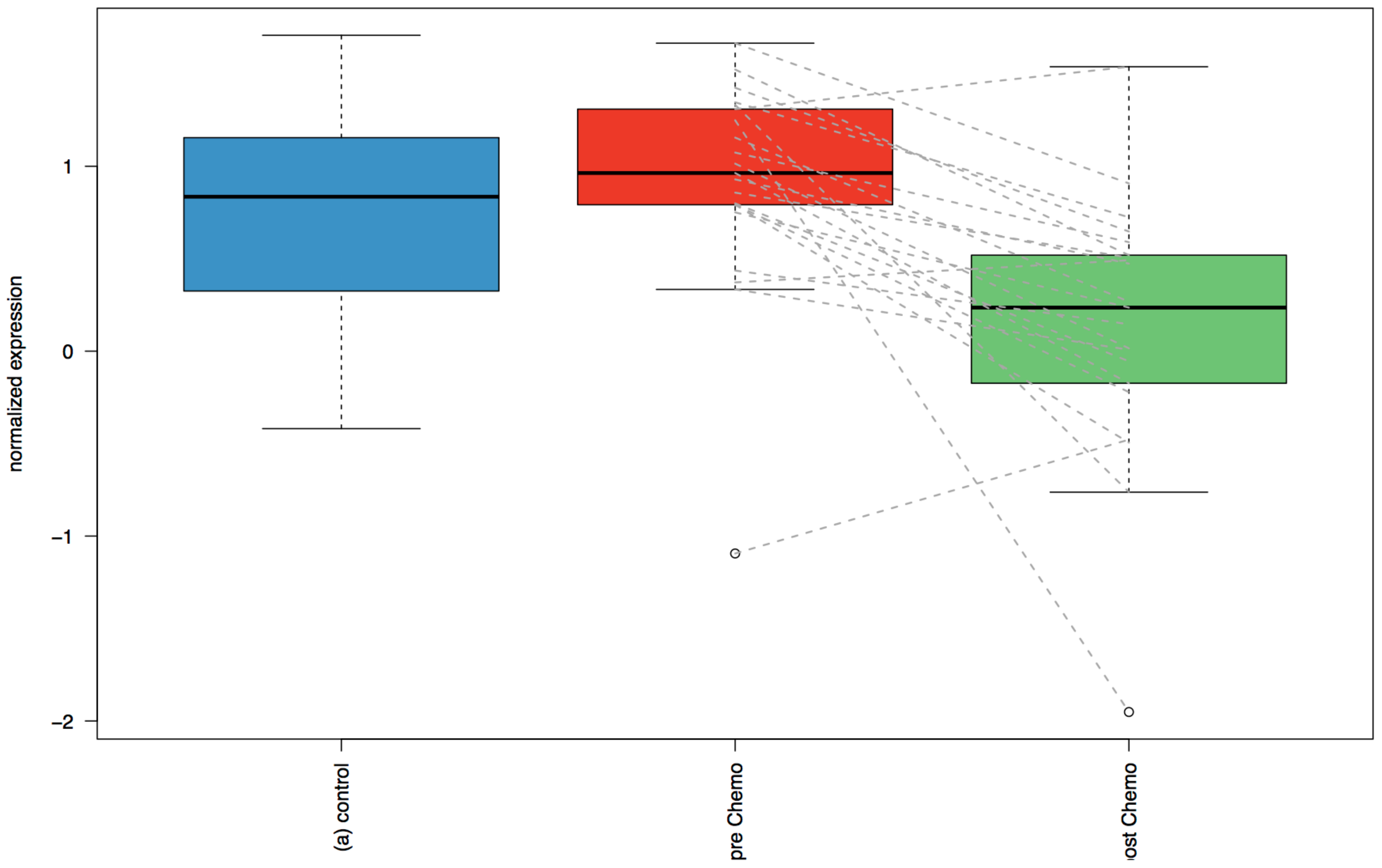
normalized expression
1
0
−1
−2
(a) control
pre Chemo
ost Chemo

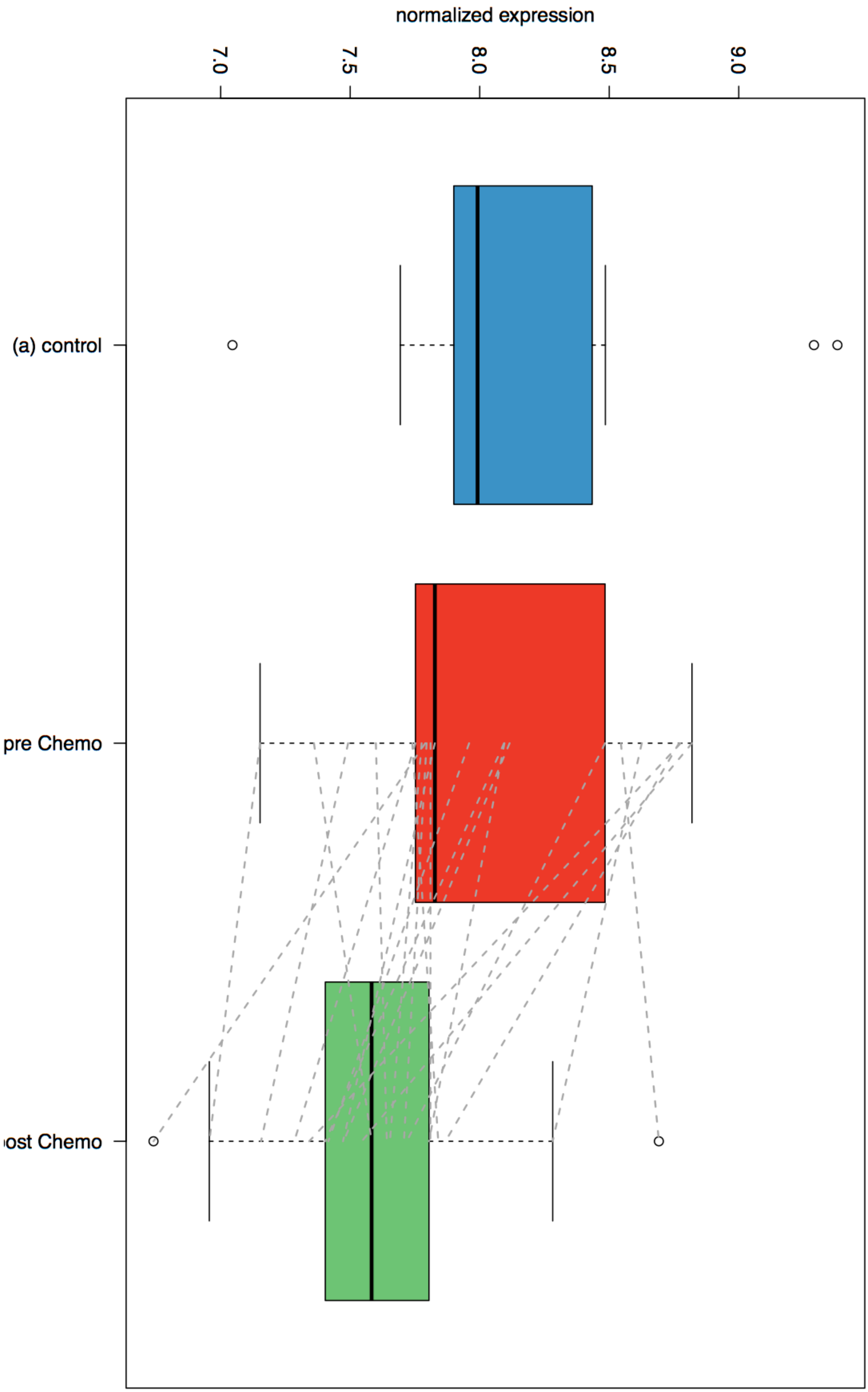
normalized expression
7.0
7.5
8.0
8.5
9.0
(a) control
pre Chemo
ost Chemo

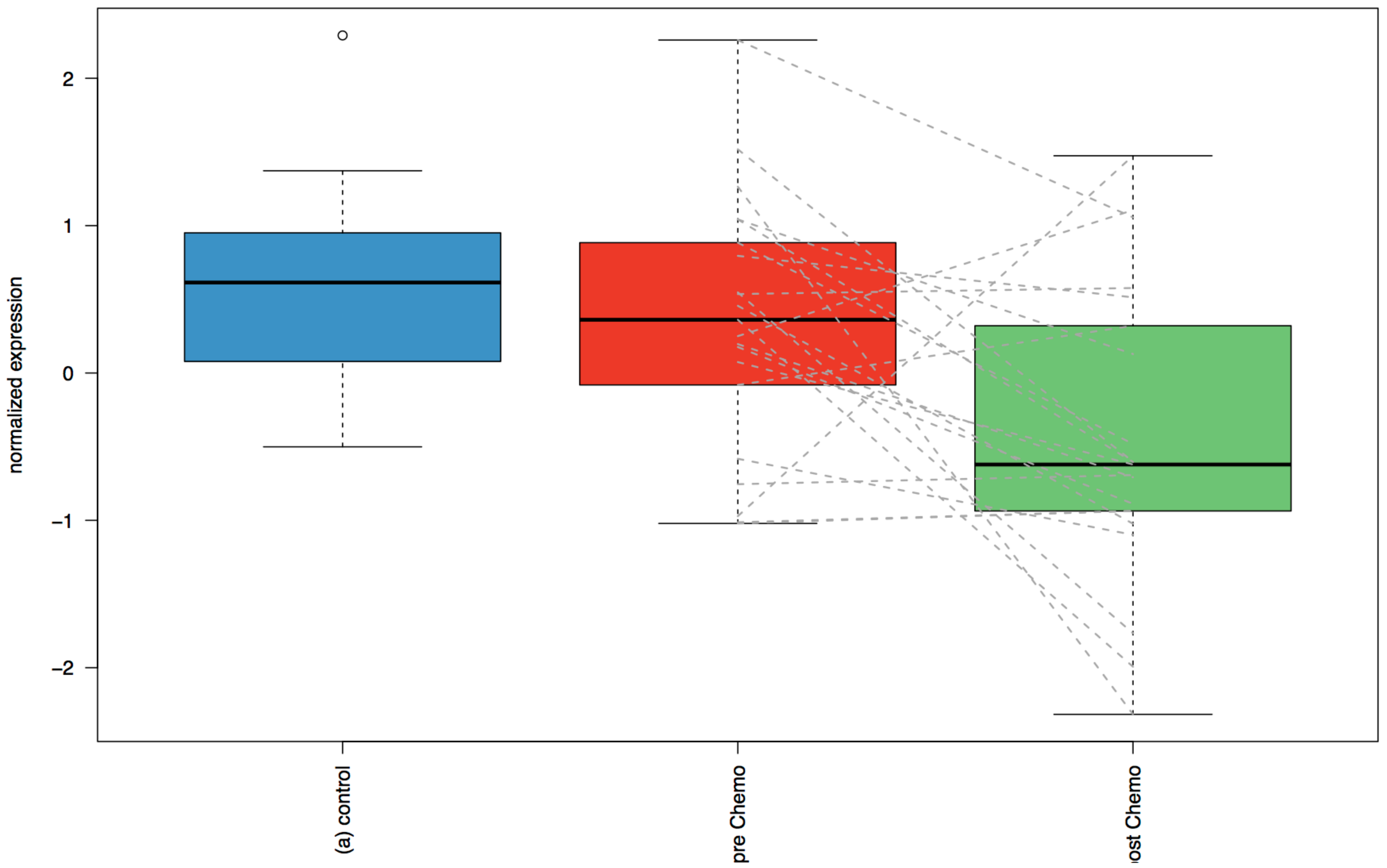
normalized expression
2
1
0
−1
−2
(a) control
pre Chemo
ost Chemo

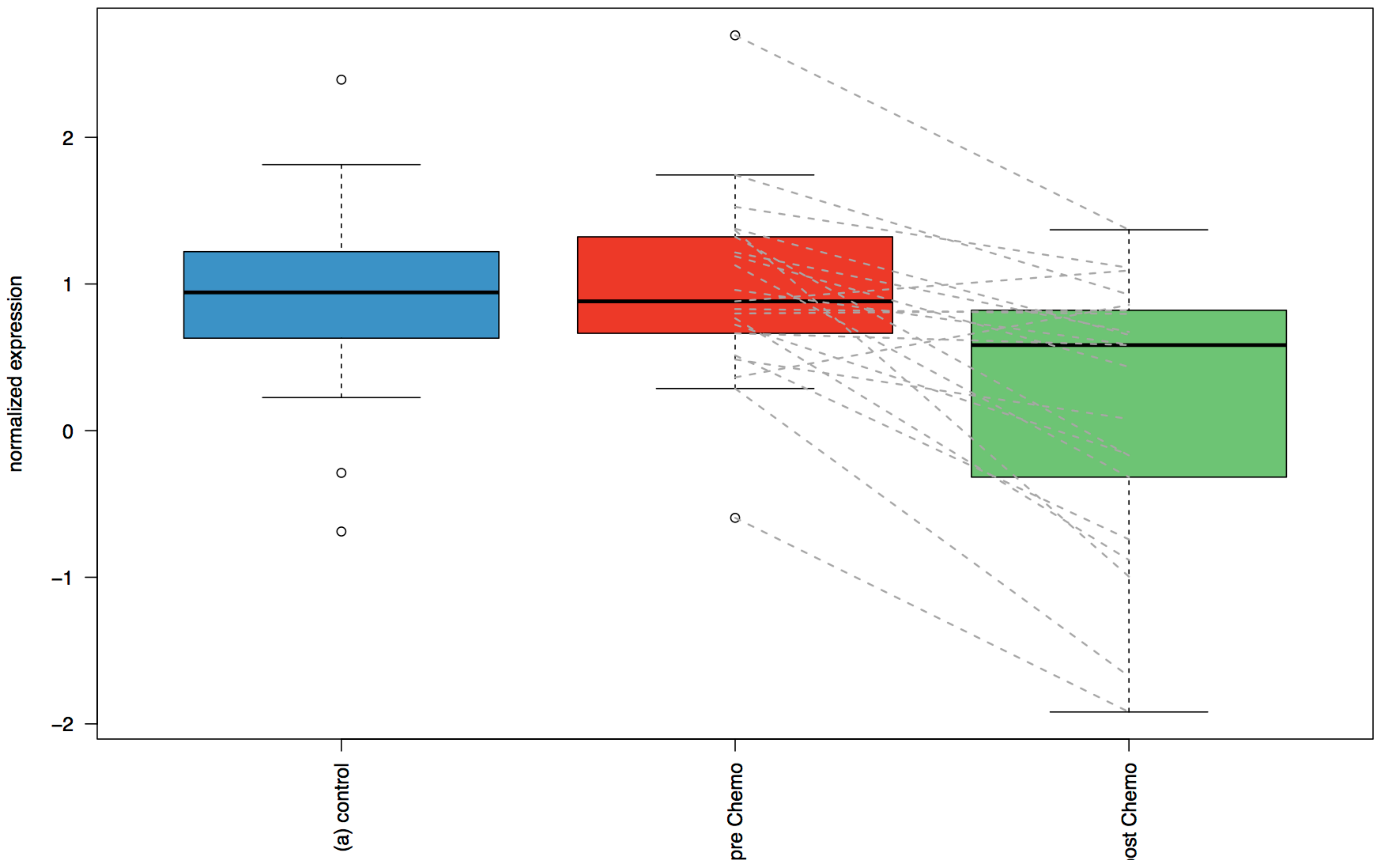
normalized expression
2
1
0
−1
−2
(a) control
pre Chemo
ost Chemo

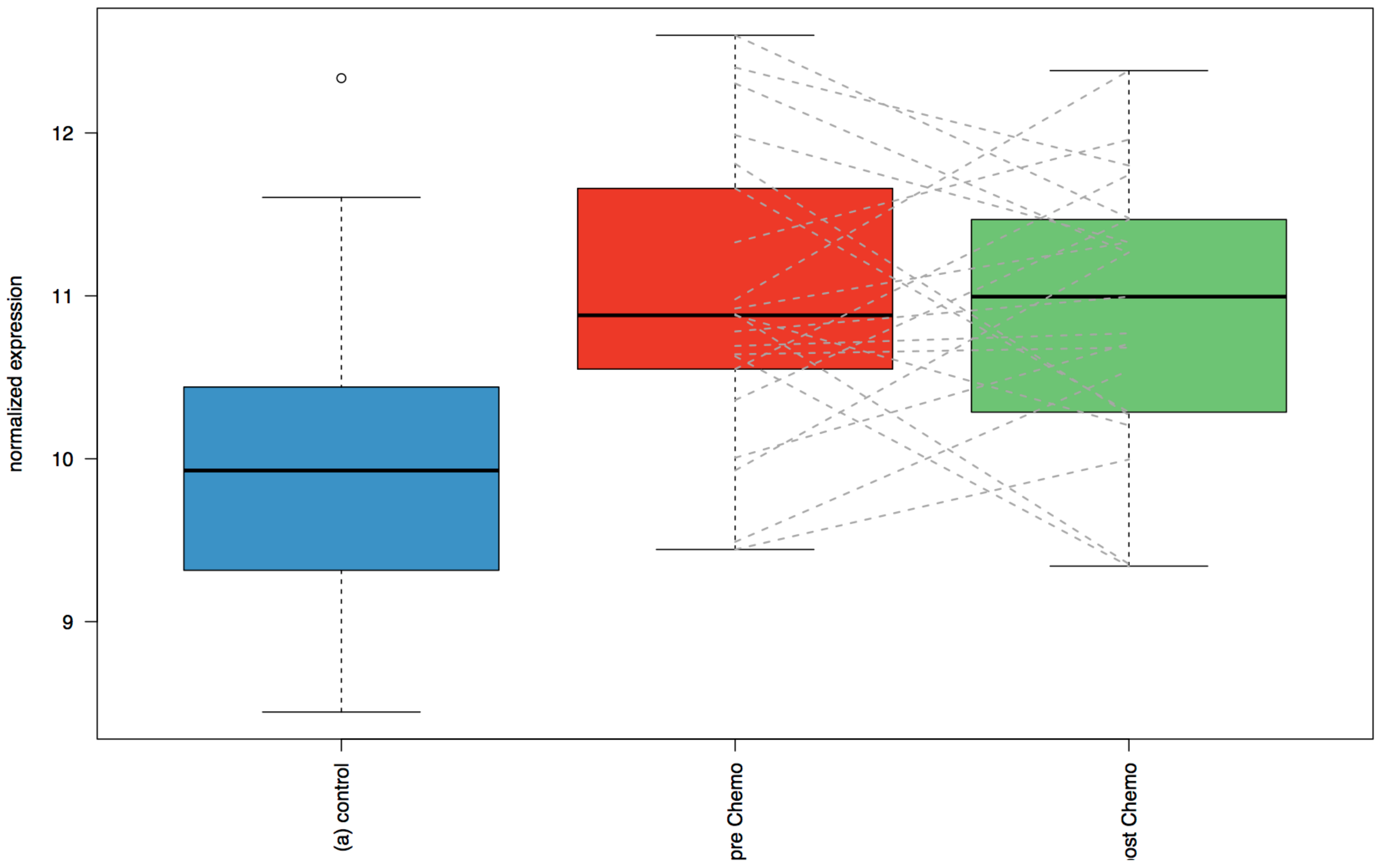

normalized expression
12
11
10
9
(a) control
pre Chemo
ost Chemo

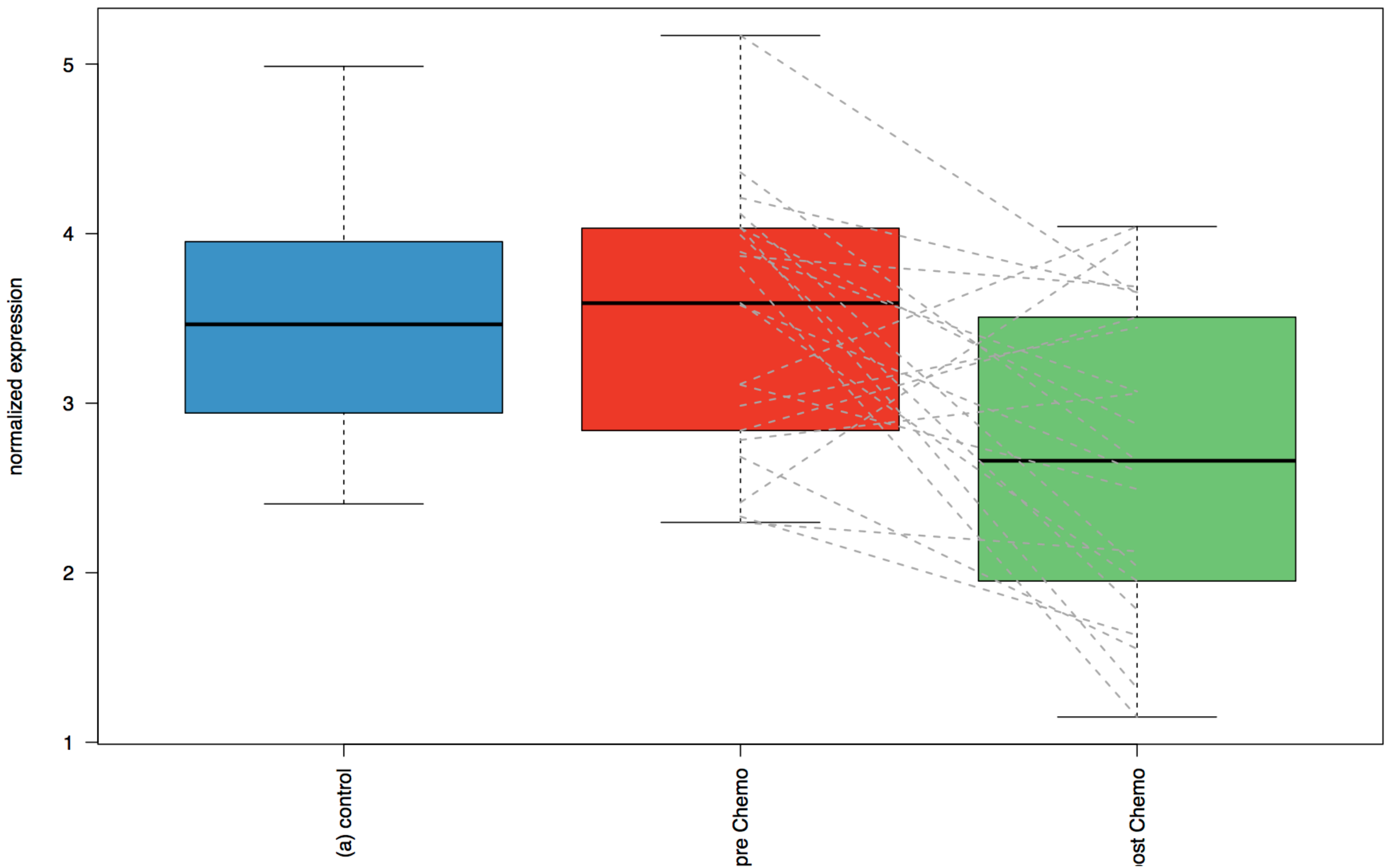
normalized expression
5
4
3
2
1
(a) control
pre Chemo
ost Chemo

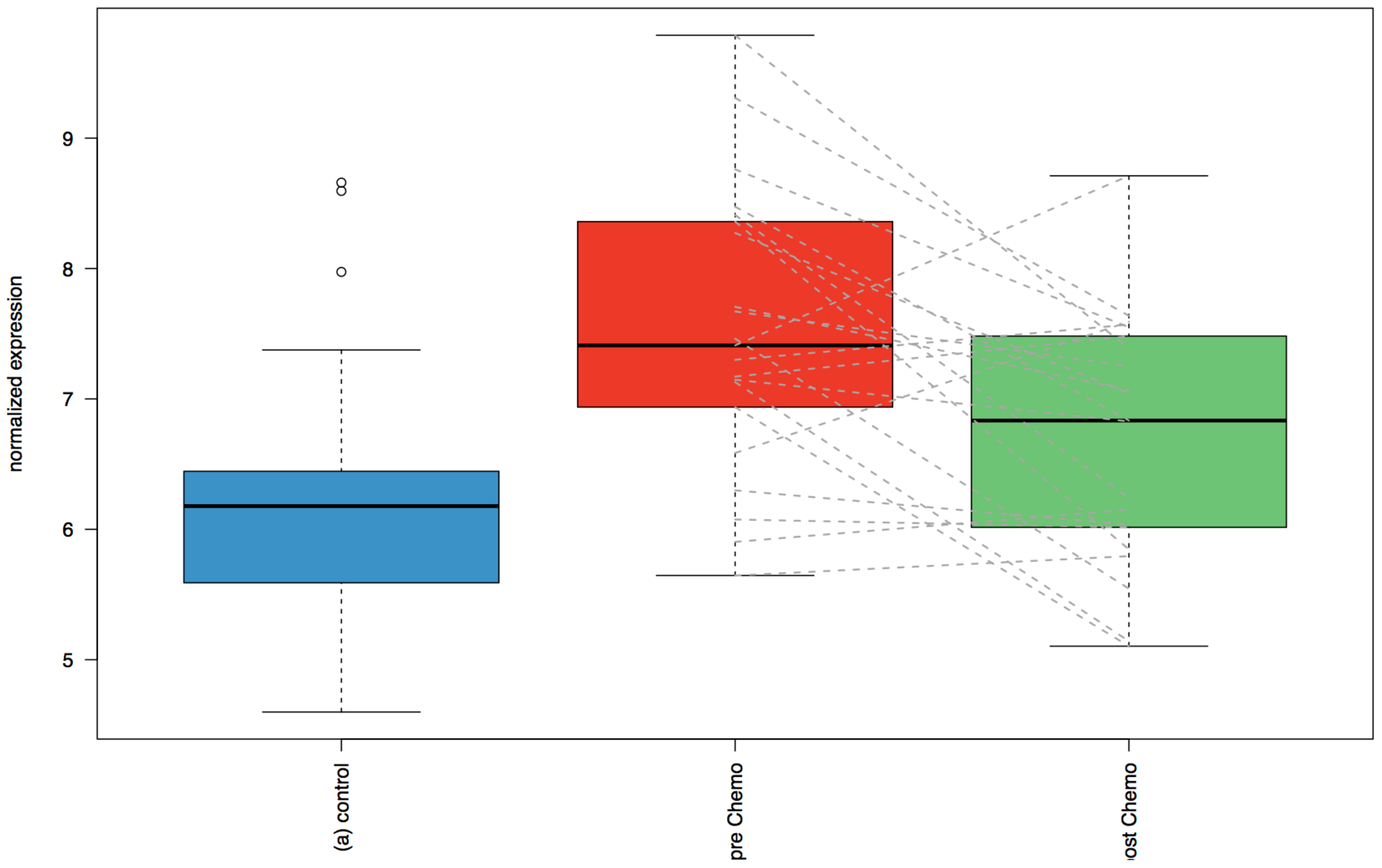
normalized expression
9
8
7
6
5
(a) control
pre Chemo
ost Chemo

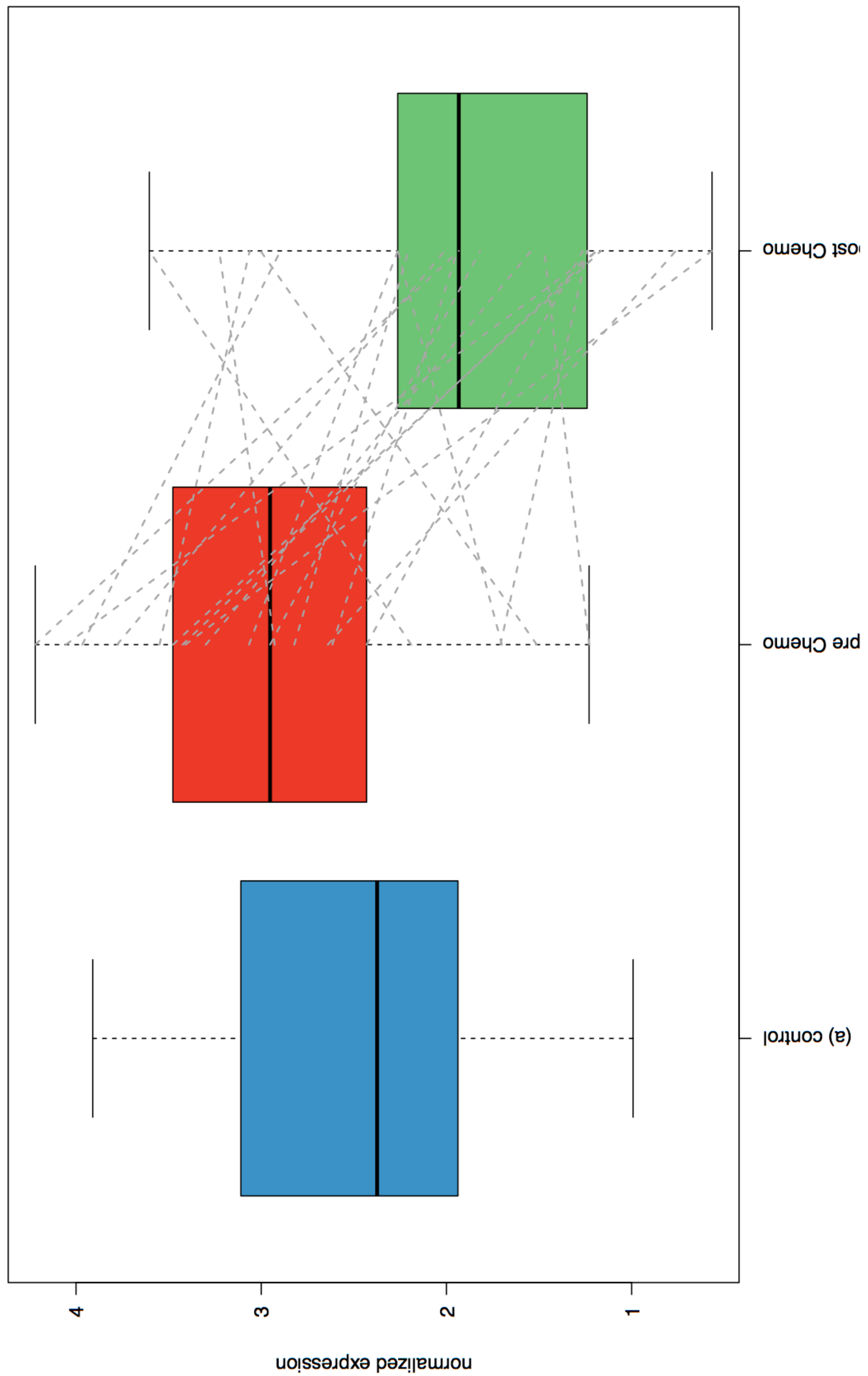

normalized expression
1
2
3
4
(a) control
pre Chemo
ost Chemo

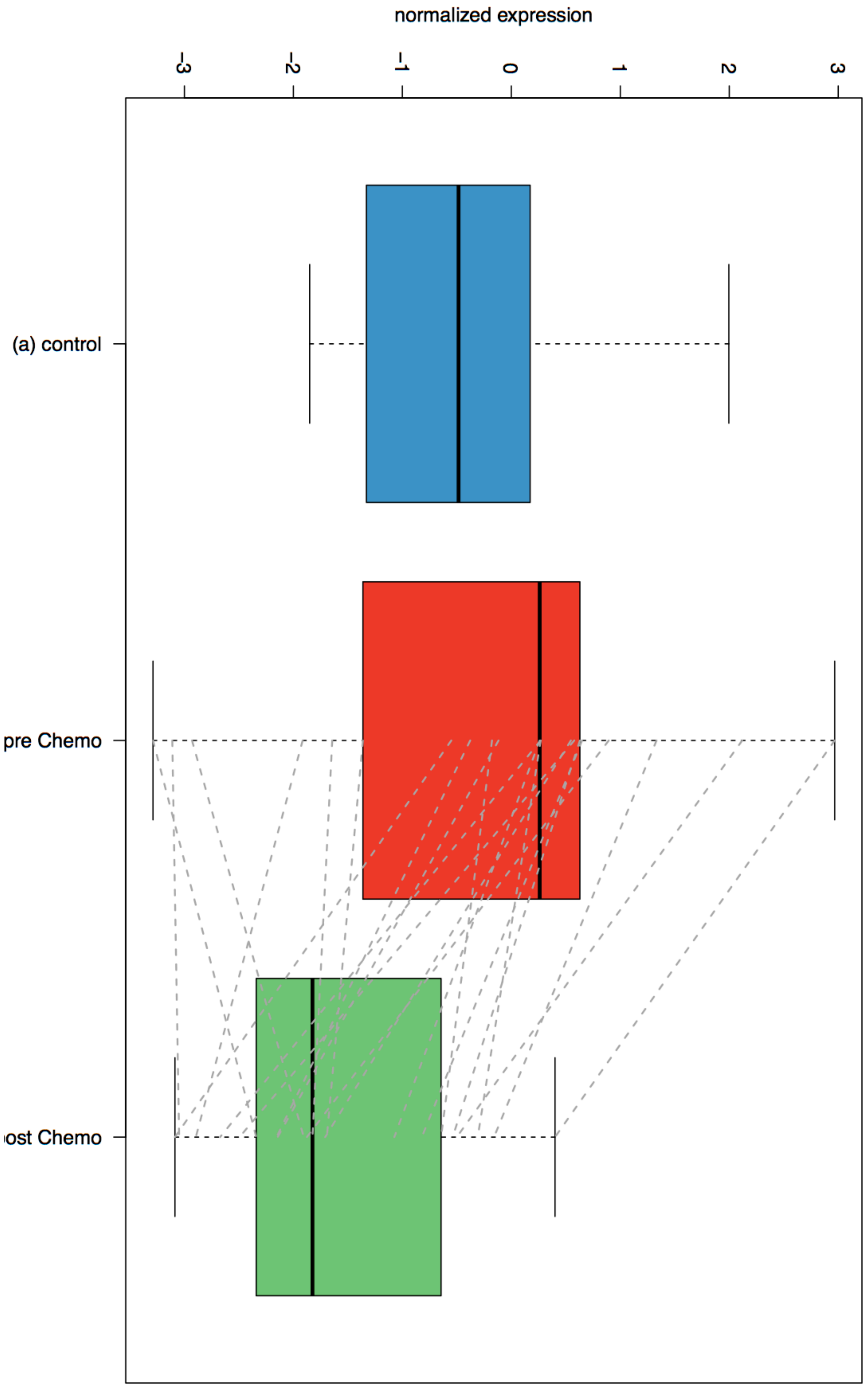
normalized expression
-3
-2
-1
0
1
2
3
(a) control
pre Chemo
ost Chemo

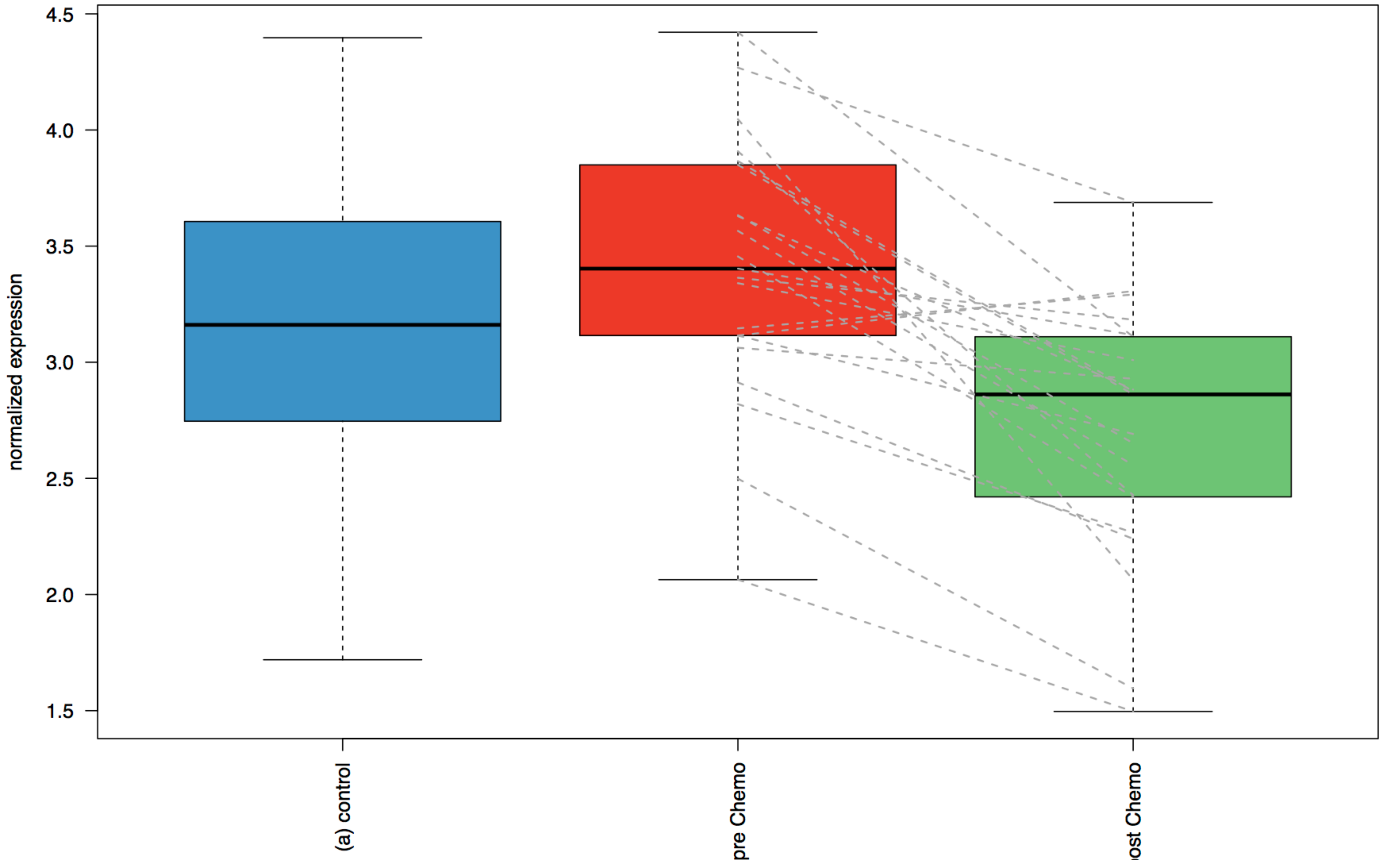
normalized expression
4.5
4.0
3.5
3.0
2.5
2.0
1.5
(a) control
pre Chemo
ost Chemo

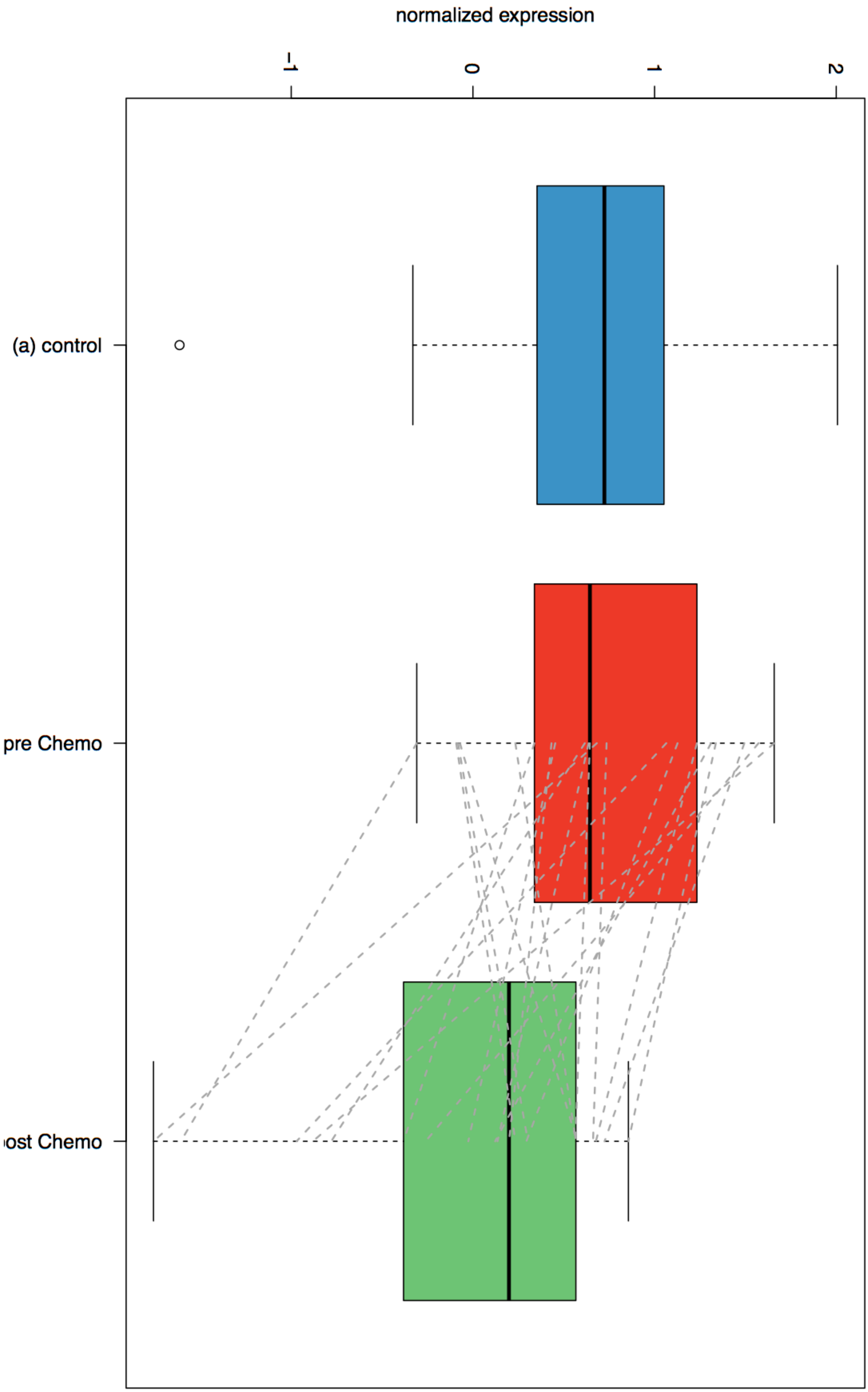

normalized expression
-1
0
1
2
(a) control
pre Chemo
ost Chemo

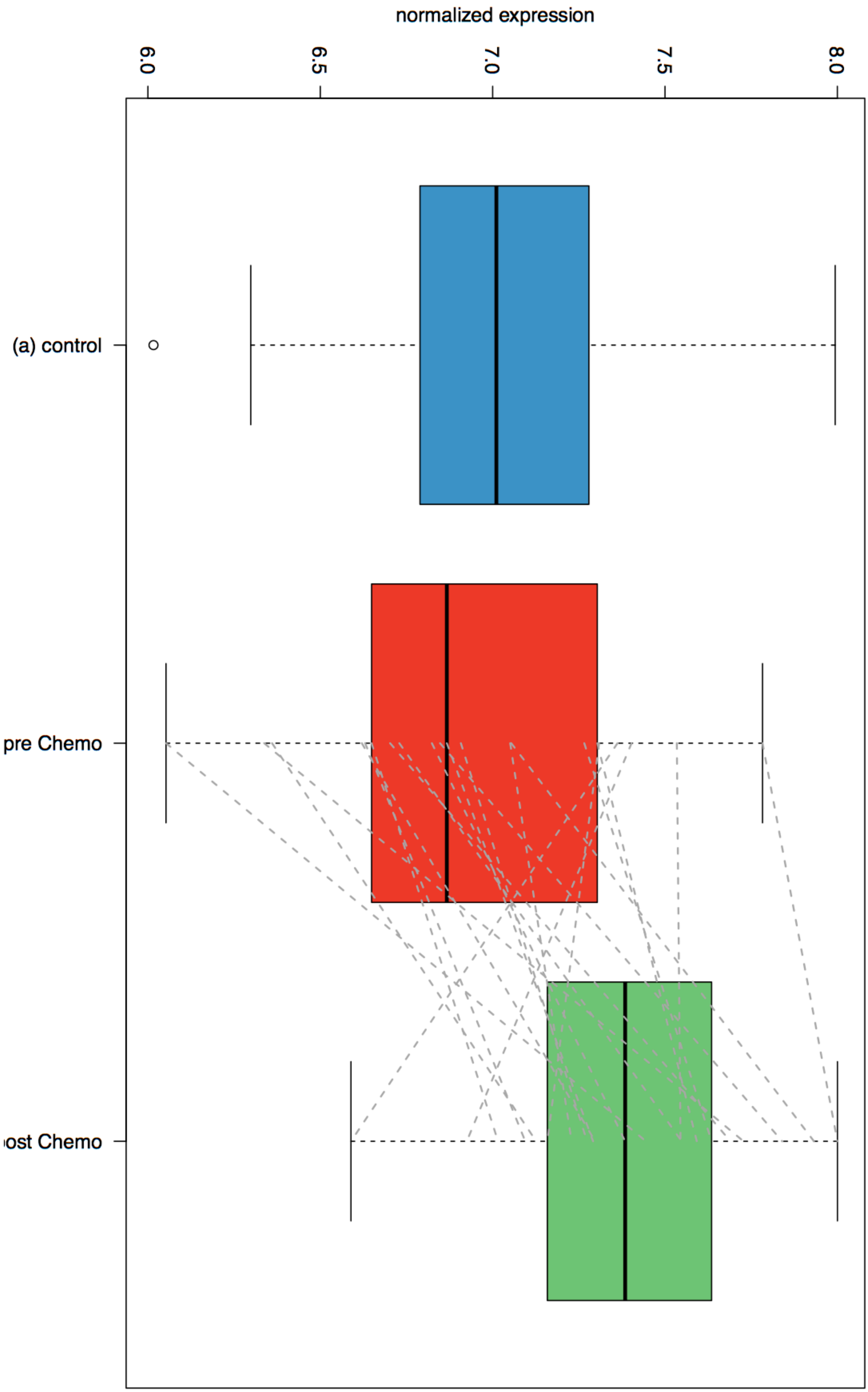

normalized expression
6.0
6.5
7.0
7.5
8.0
(a) control
pre Chemo
ost Chemo

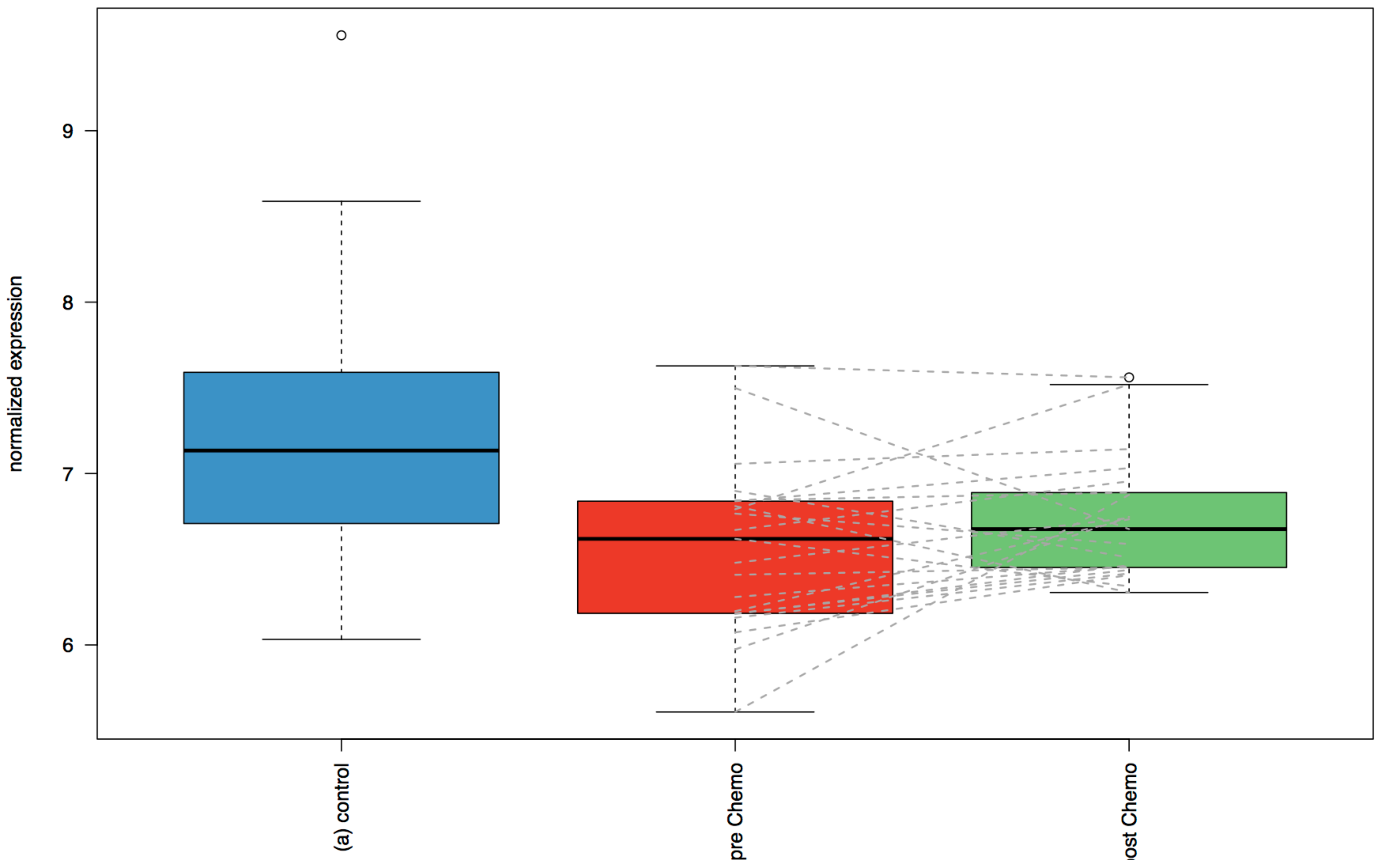
normalized expression
9
8
7
6
(a) control
pre Chemo
ost Chemo

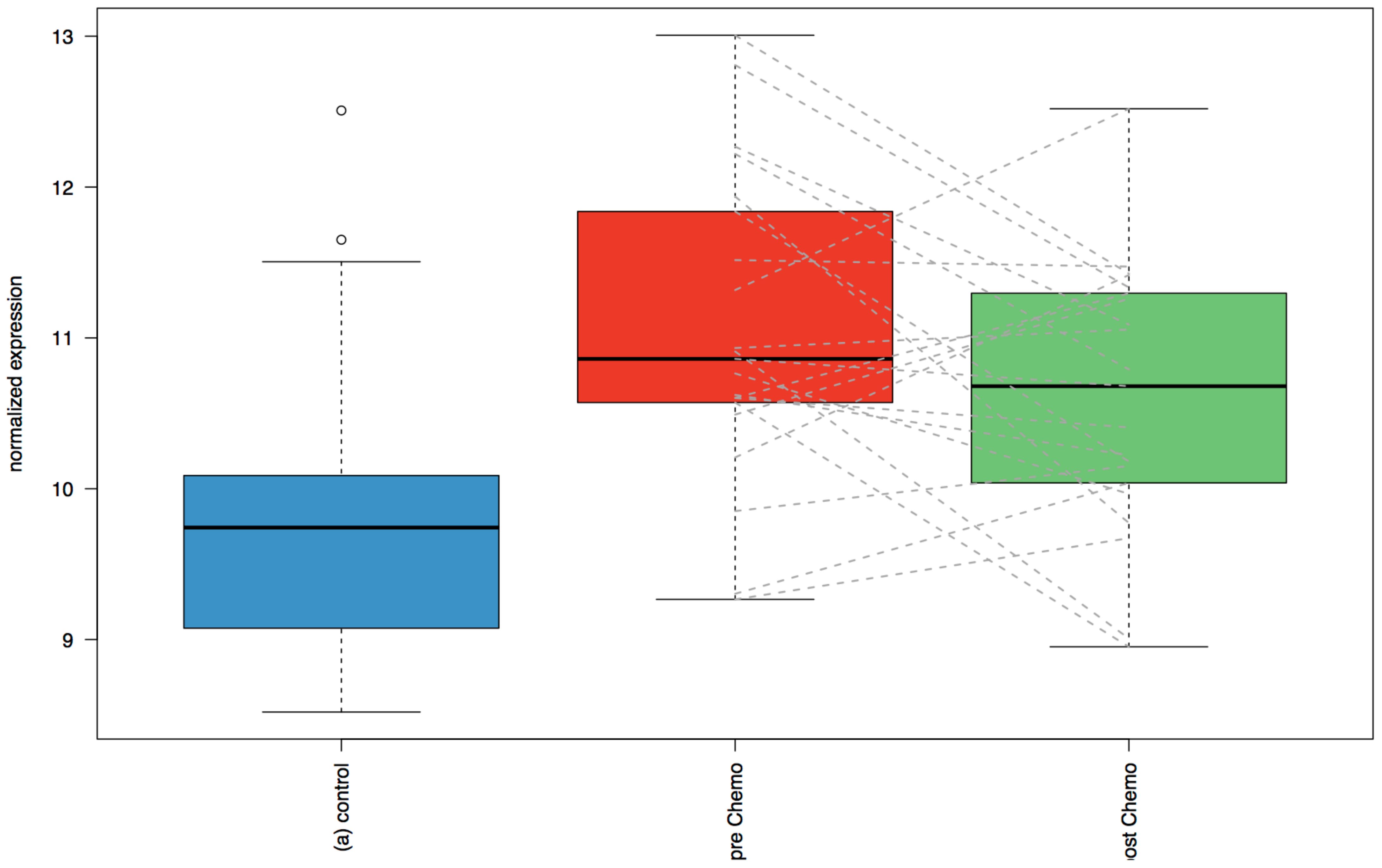

normalized expression
13
12
11
10
9
(a) control
pre Chemo
ost Chemo

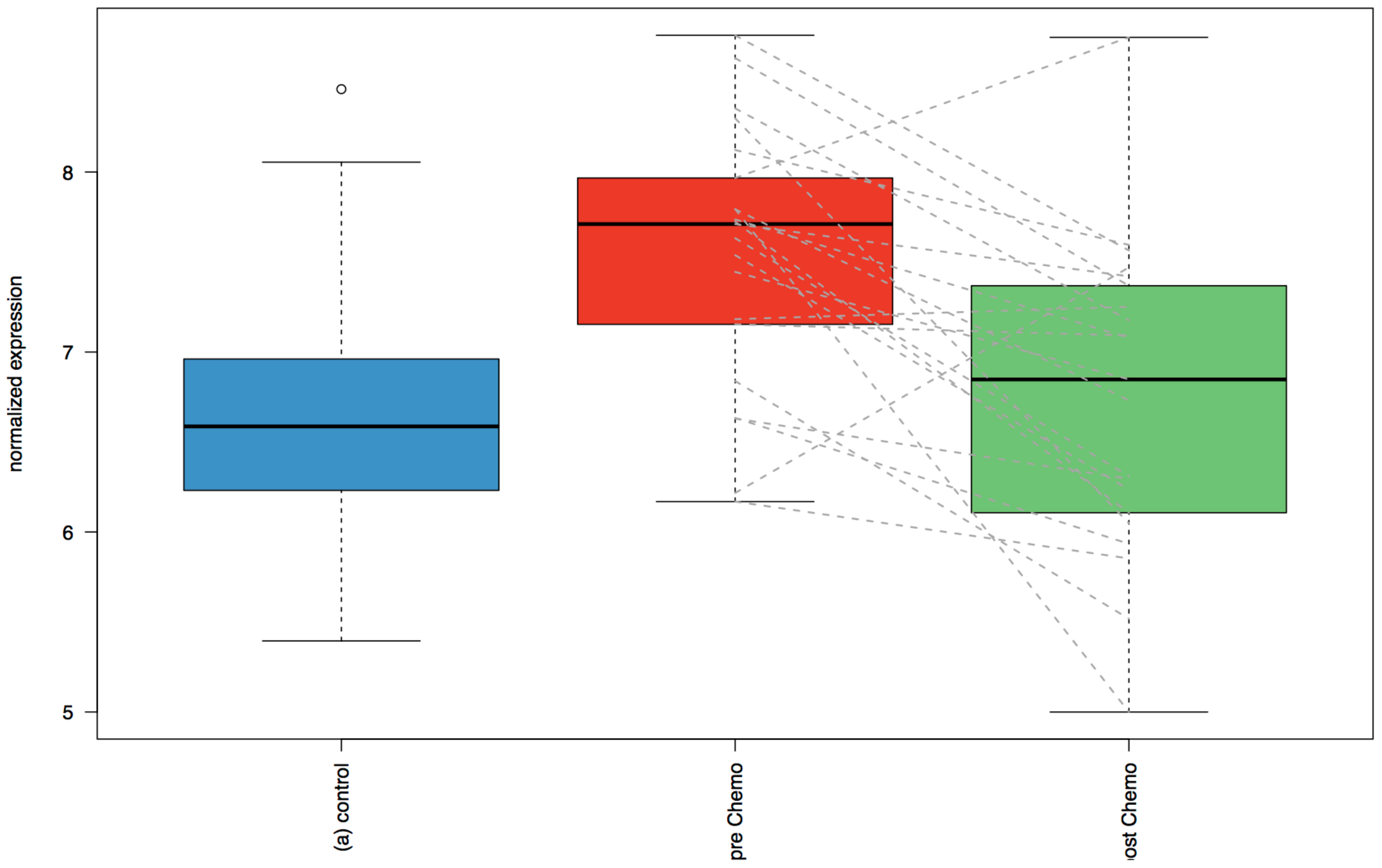
normalized expression
8
7
6
5
(a) control
pre Chemo
ost Chemo

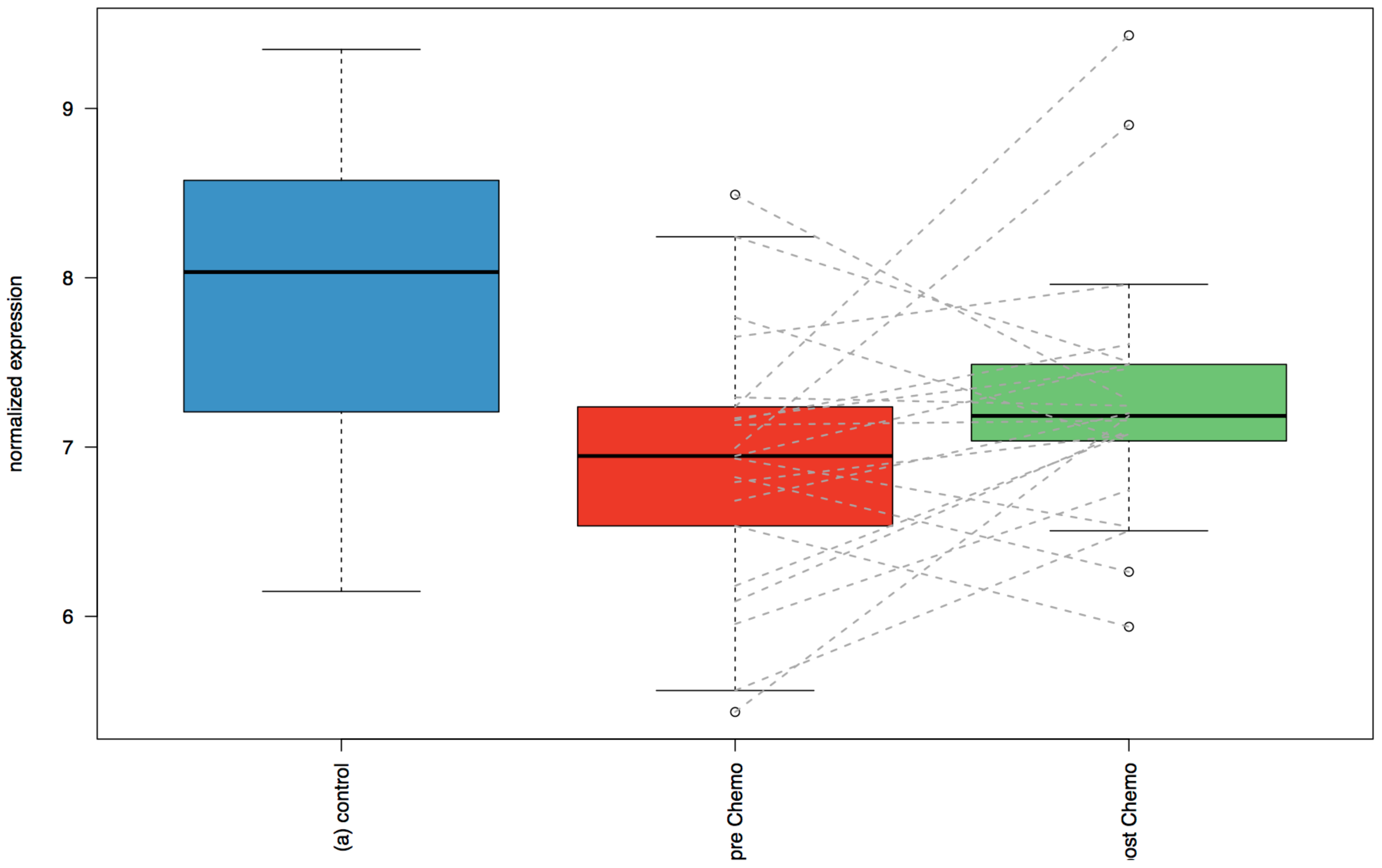
normalized expression
9
8
7
6
(a) control
pre Chemo
ost Chemo

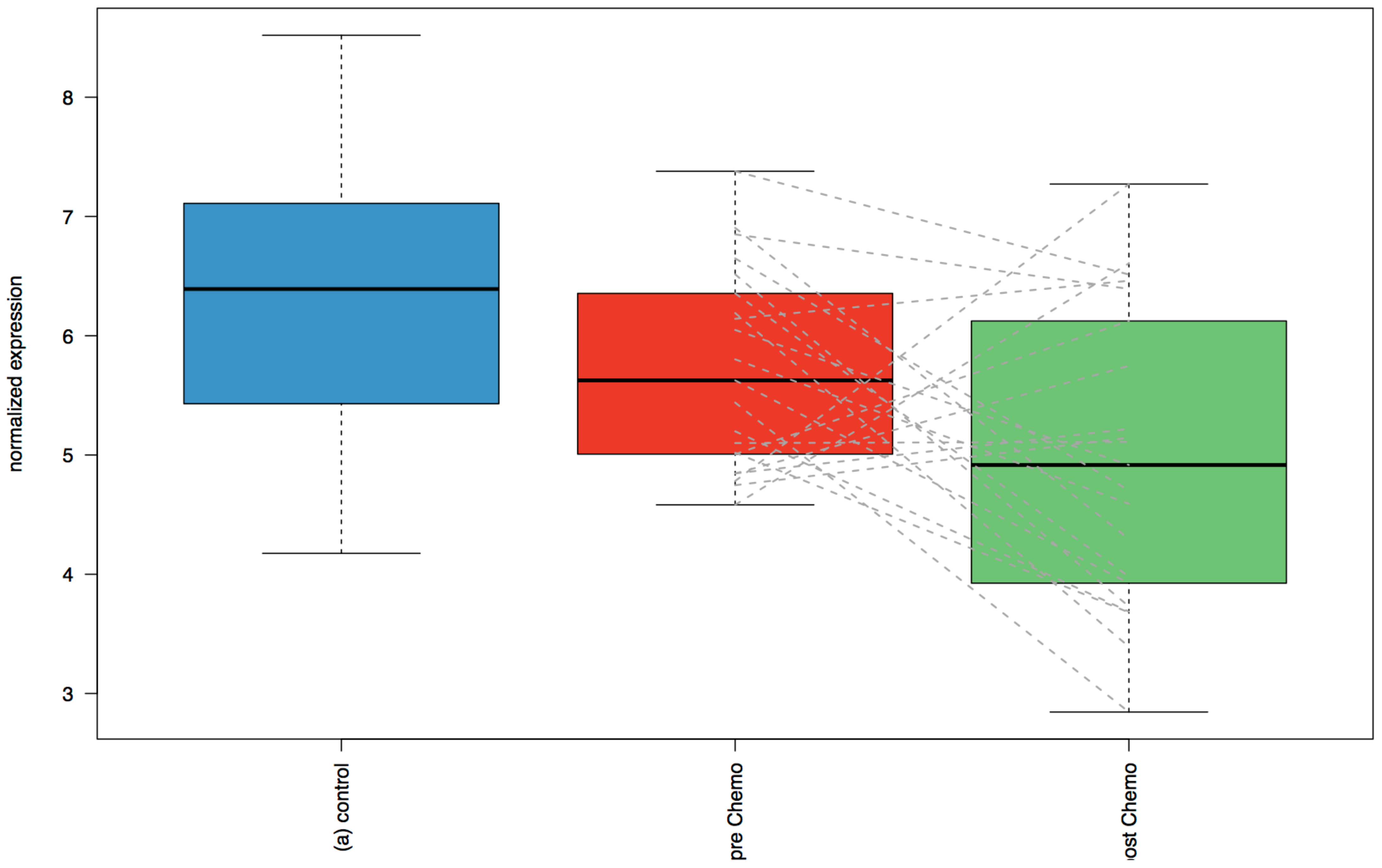

normalized expression
8
7
6
5
4
3
(a) control
pre Chemo
ost Chemo

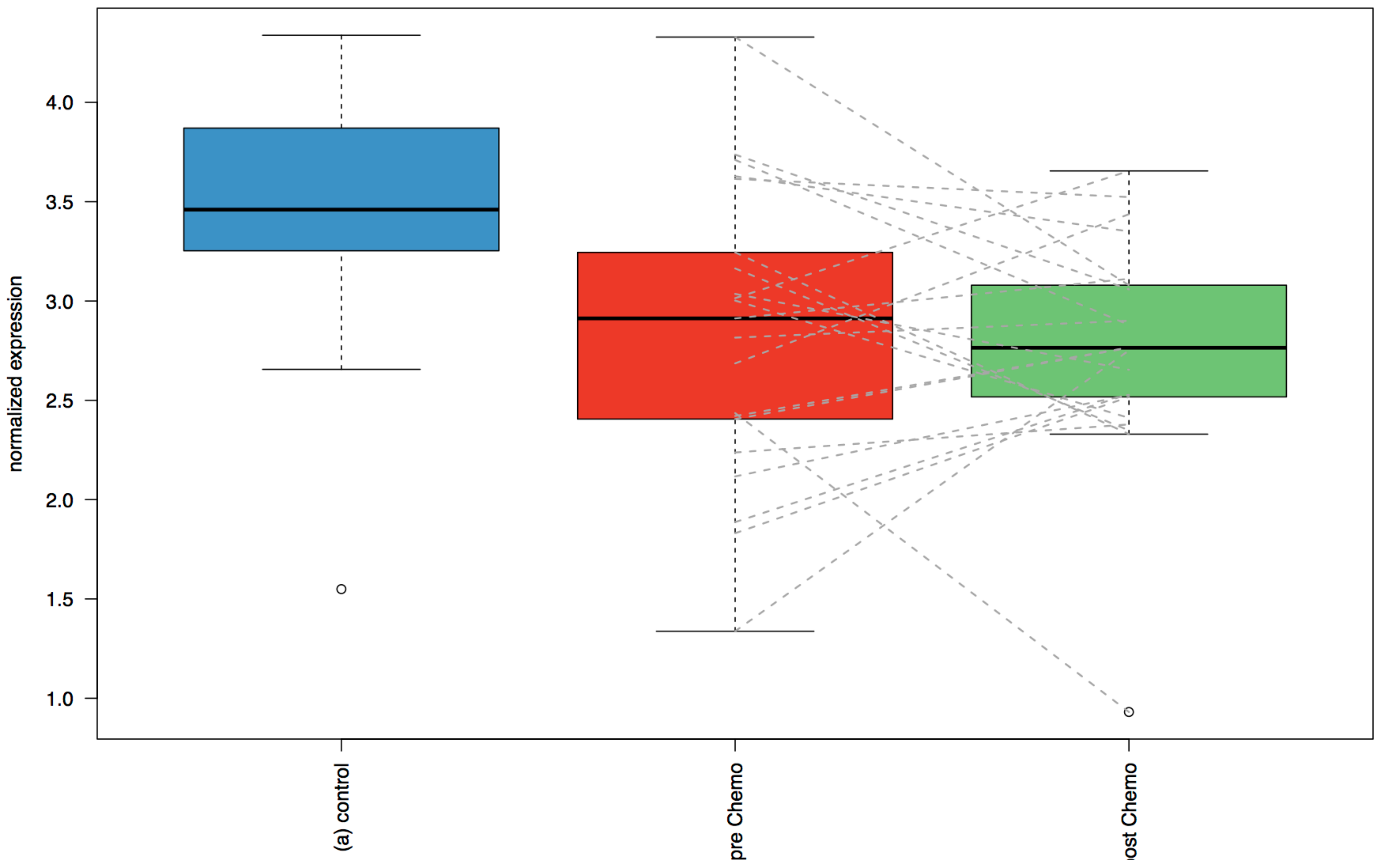
normalized expression
4.0
3.5
3.0
2.5
2.0
1.5
1.0
(a) control
pre Chemo
ost Chemo

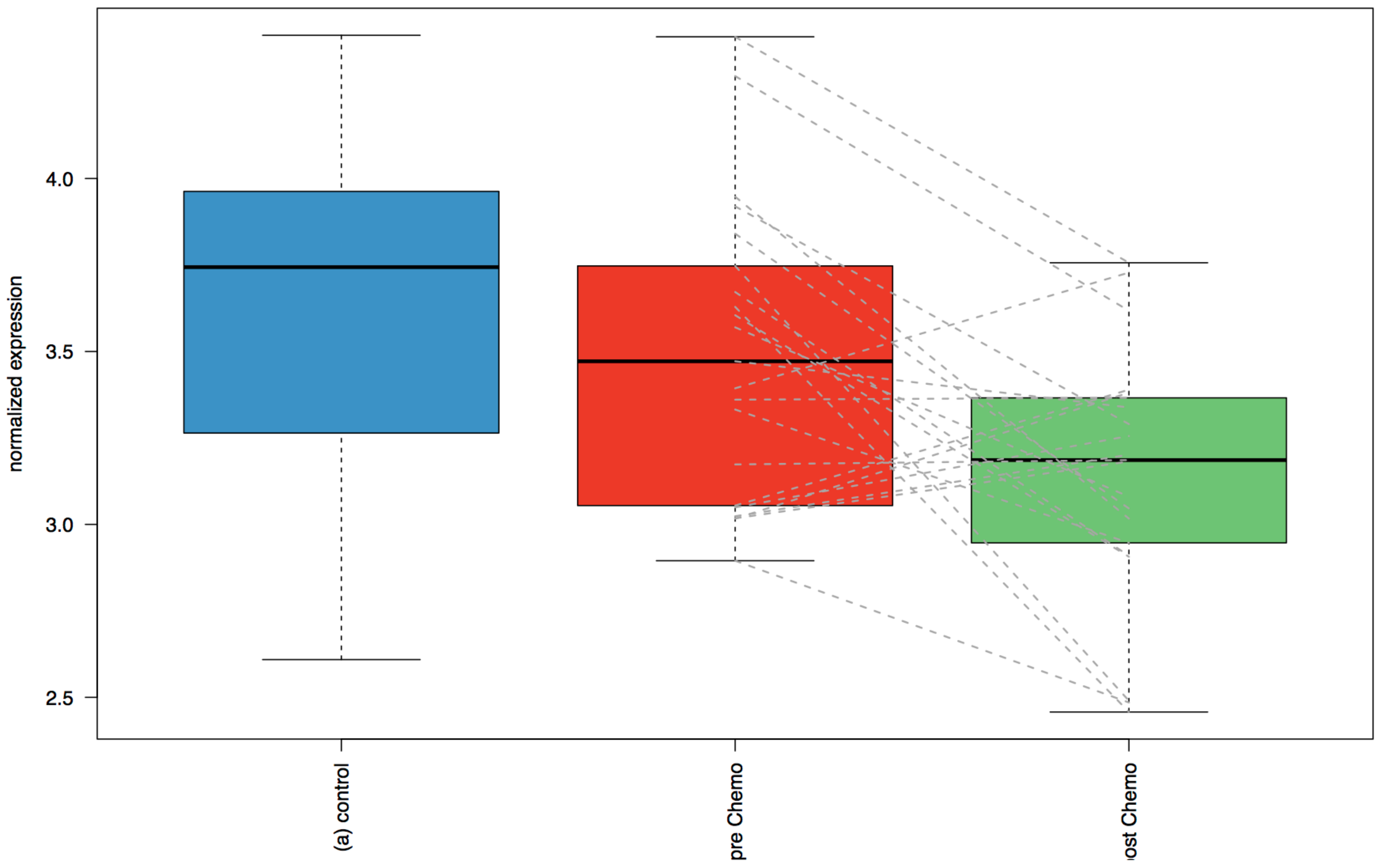
normalized expression
4.0
3.5
3.0
2.5
(a) control
pre Chemo
ost Chemo

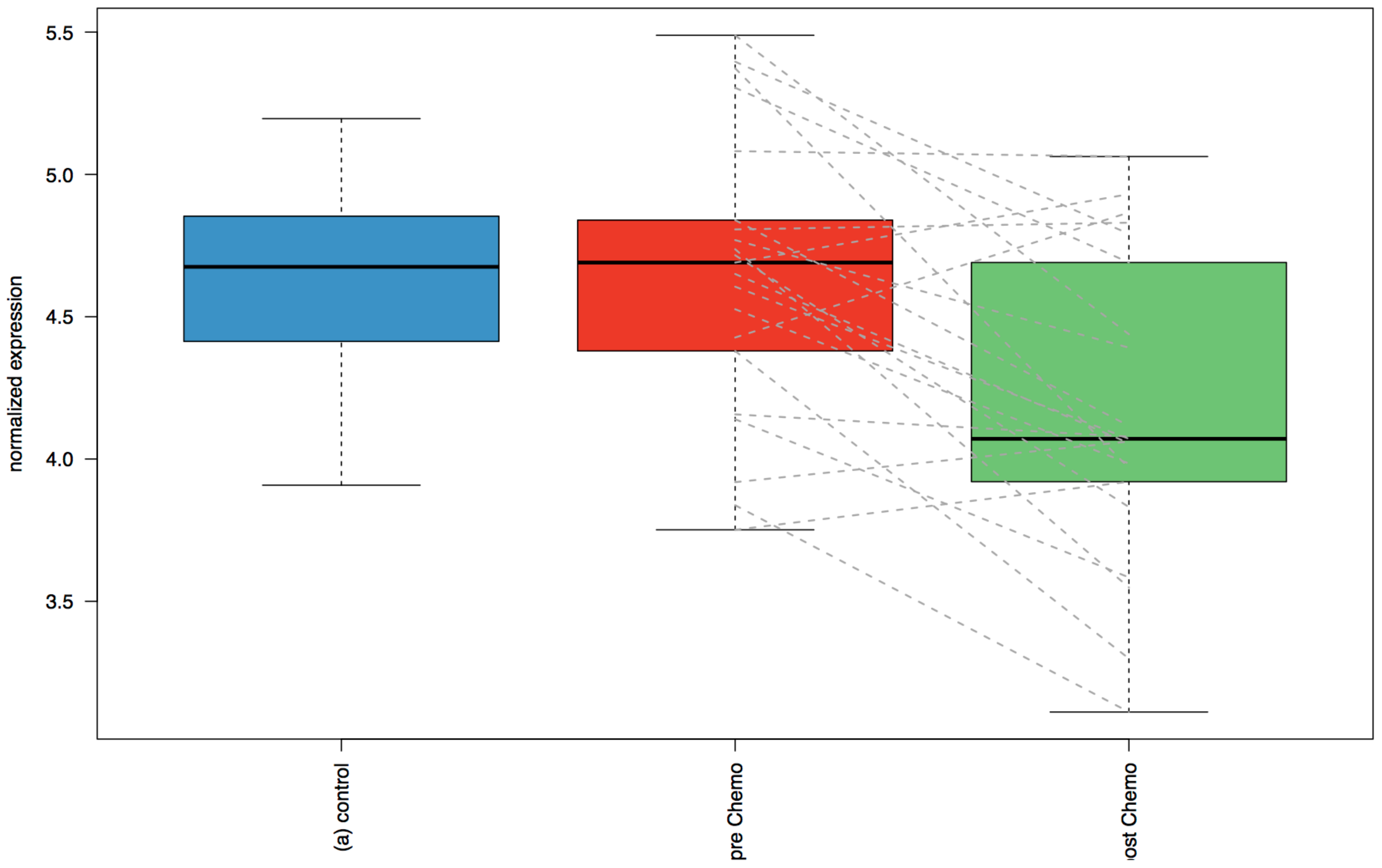
normalized expression
5.5
5.0
4.5
4.0
3.5
(a) control
pre Chemo
ost Chemo

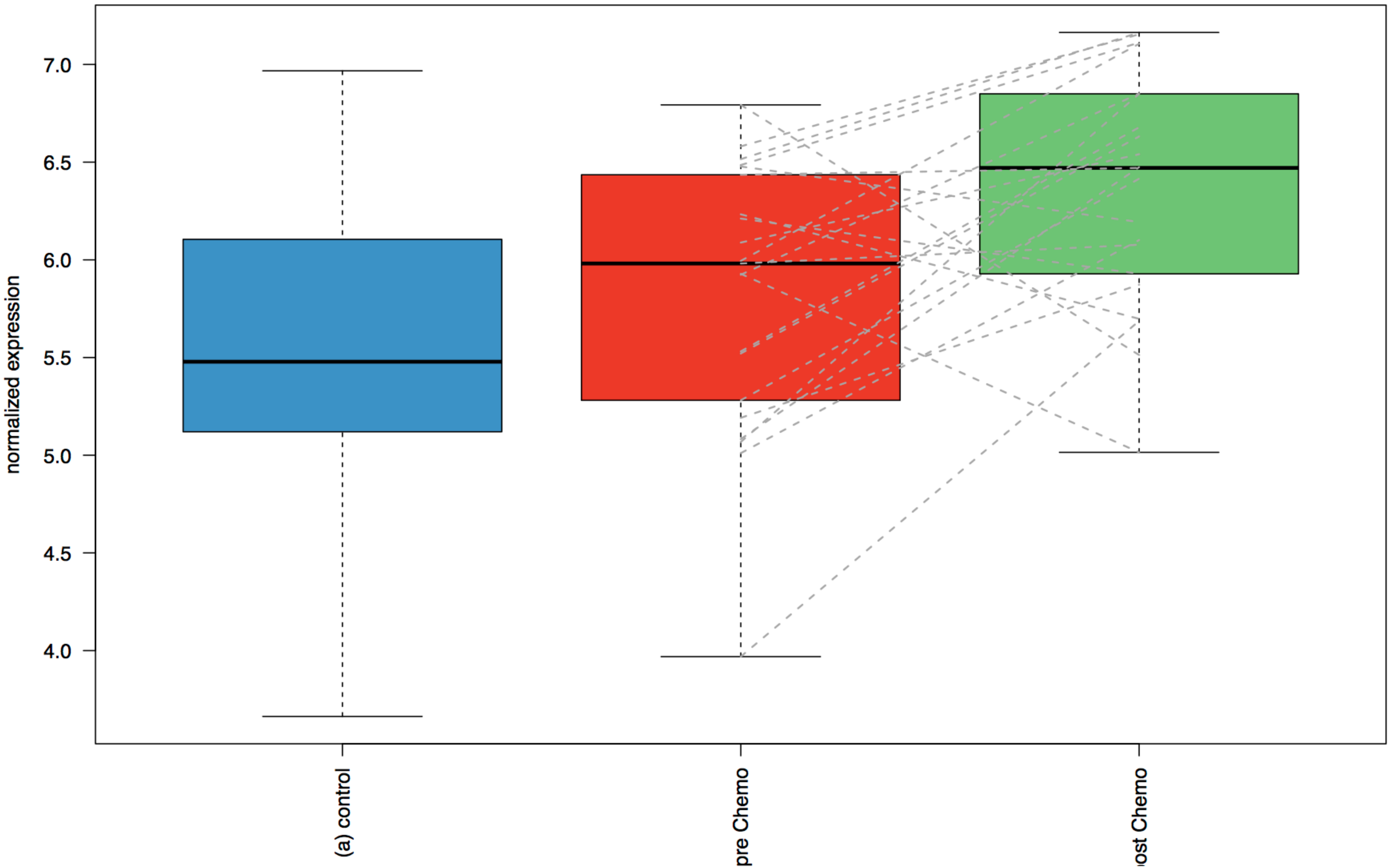
normalized expression
7.0
6.5
6.0
5.5
5.0
4.5
4.0
(a) control
pre Chemo
ost Chemo

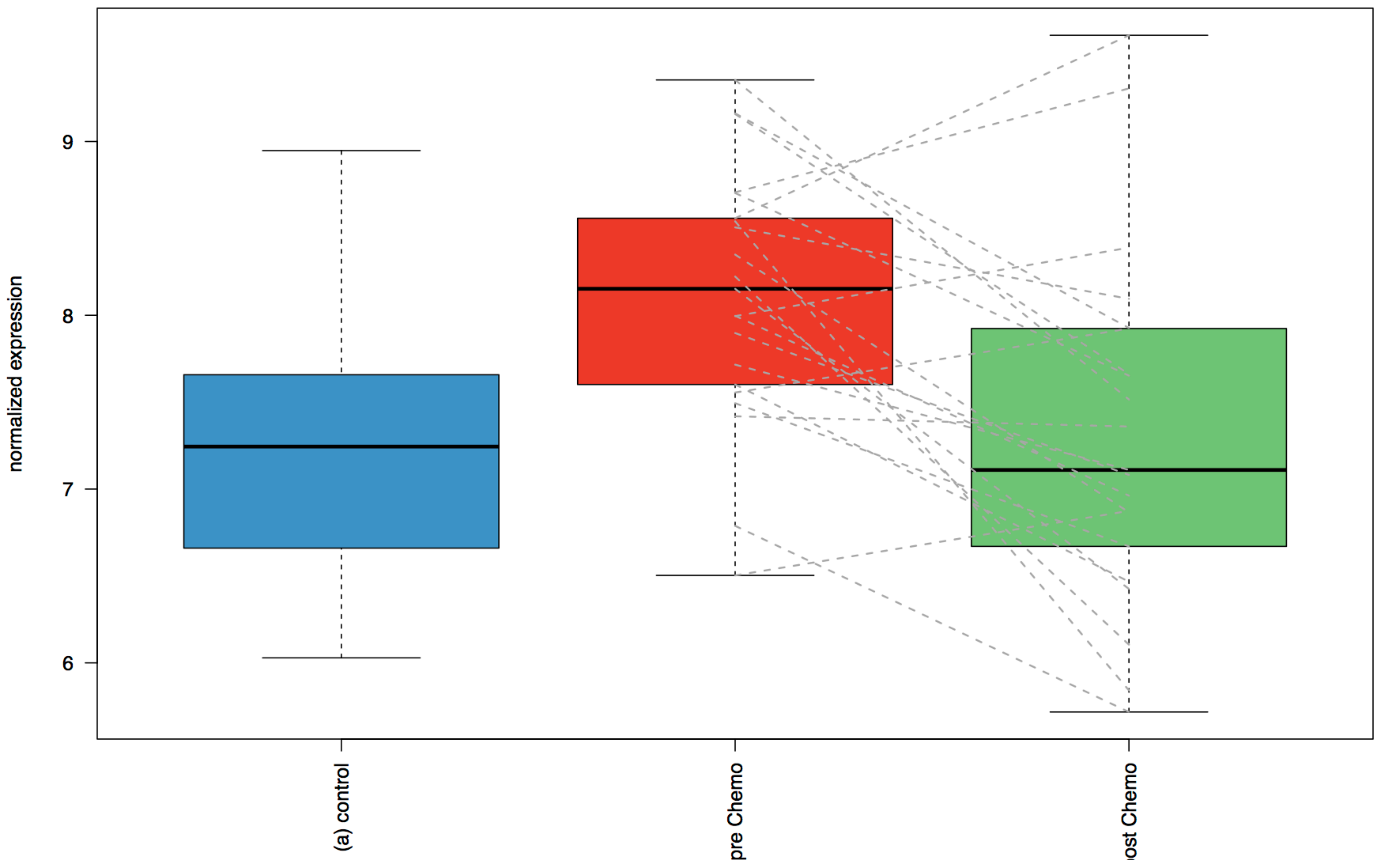
normalized expression
9
8
7
6
(a) control
pre Chemo
ost Chemo

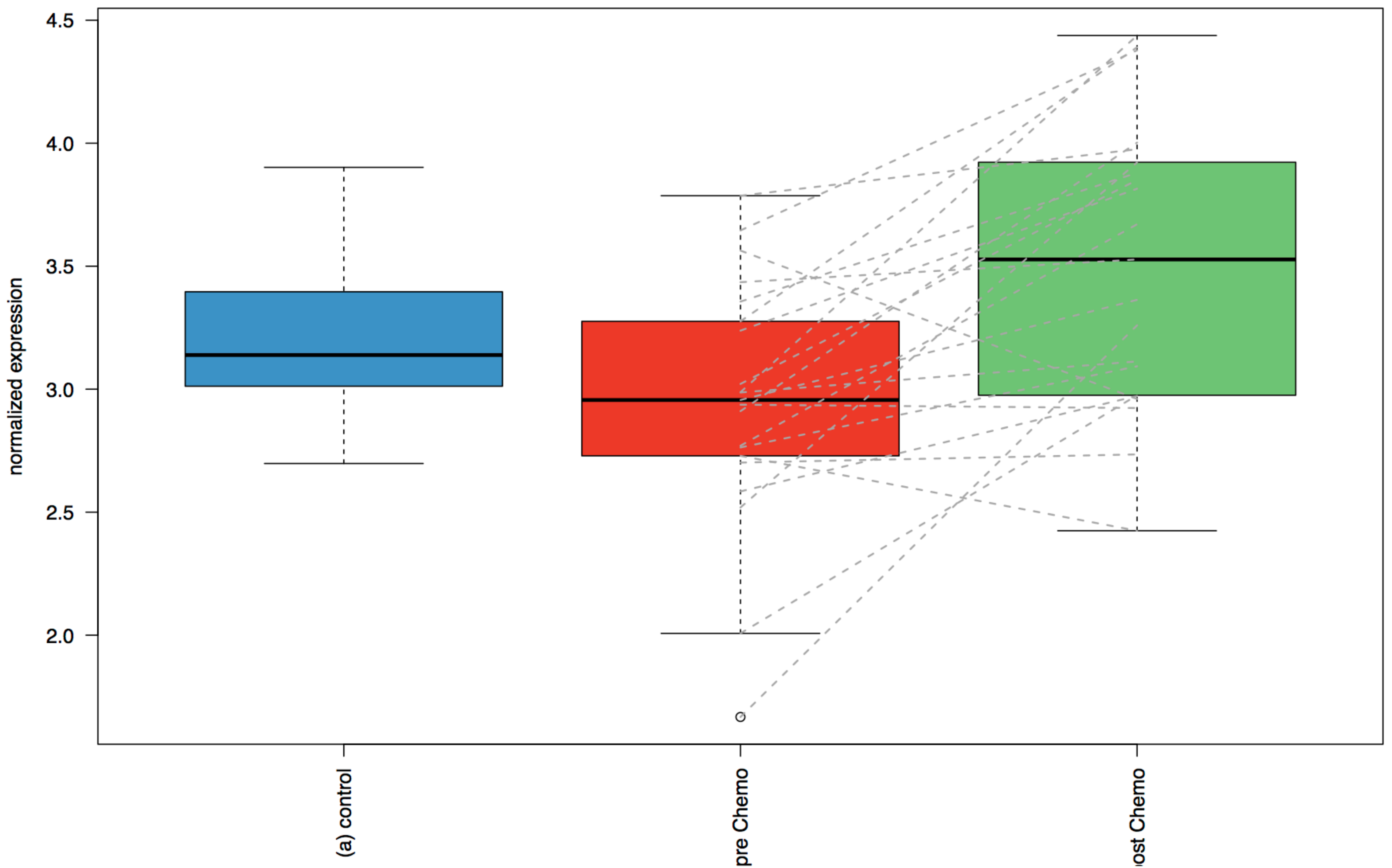
normalized expression
4.5
4.0
3.5
3.0
2.5
2.0
(a) control
pre Chemo
ost Chemo

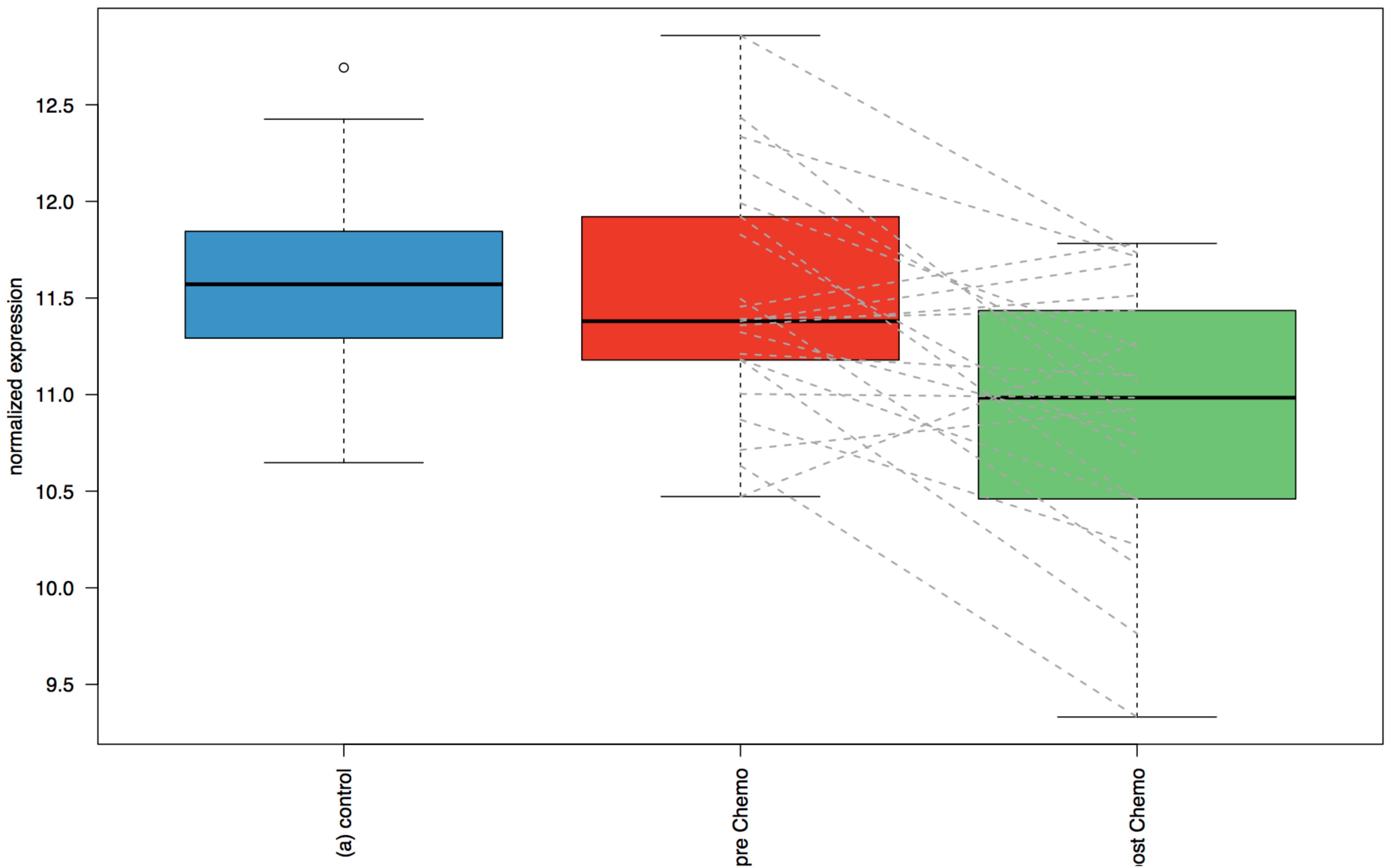
normalized expression
12.5
12.0
11.5
11.0
10.5
10.0
9.5
(a) control
pre Chemo
ost Chemo

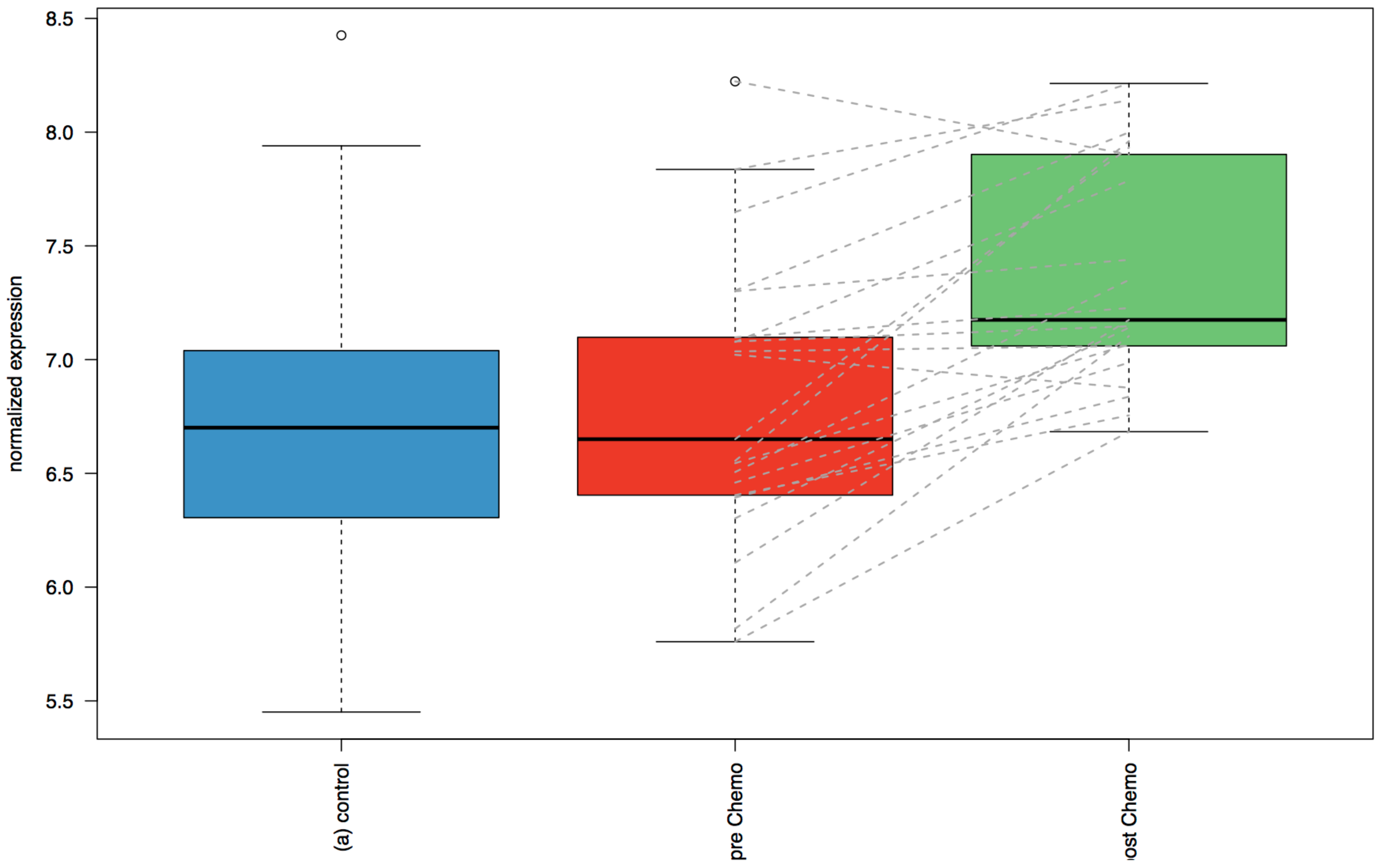
normalized expression
8.5
8.0
7.5
7.0
6.5
6.0
5.5
(a) control
pre Chemo
ost Chemo

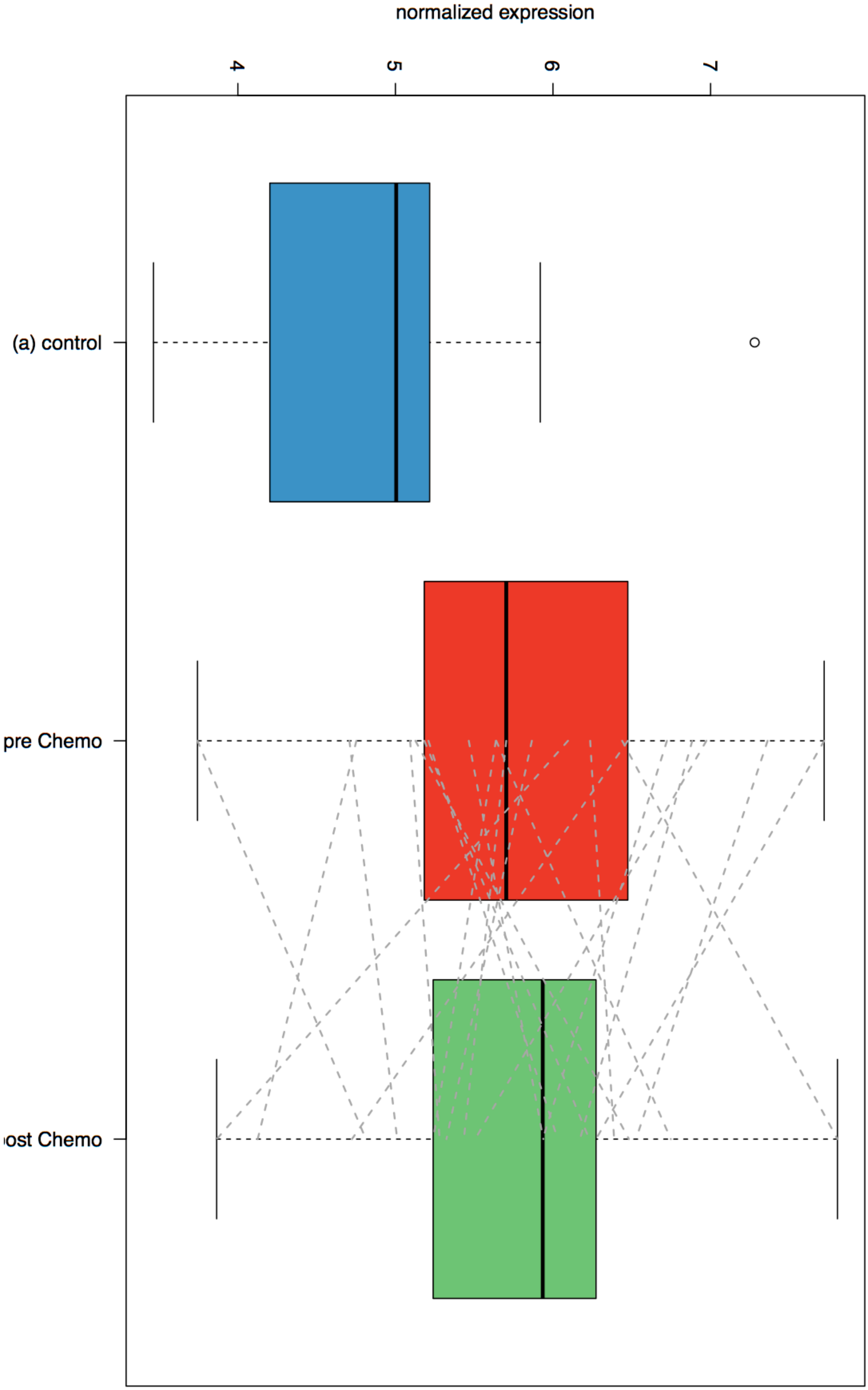

normalized expression
4
5
6
7
(a) control
pre Chemo
ost Chemo

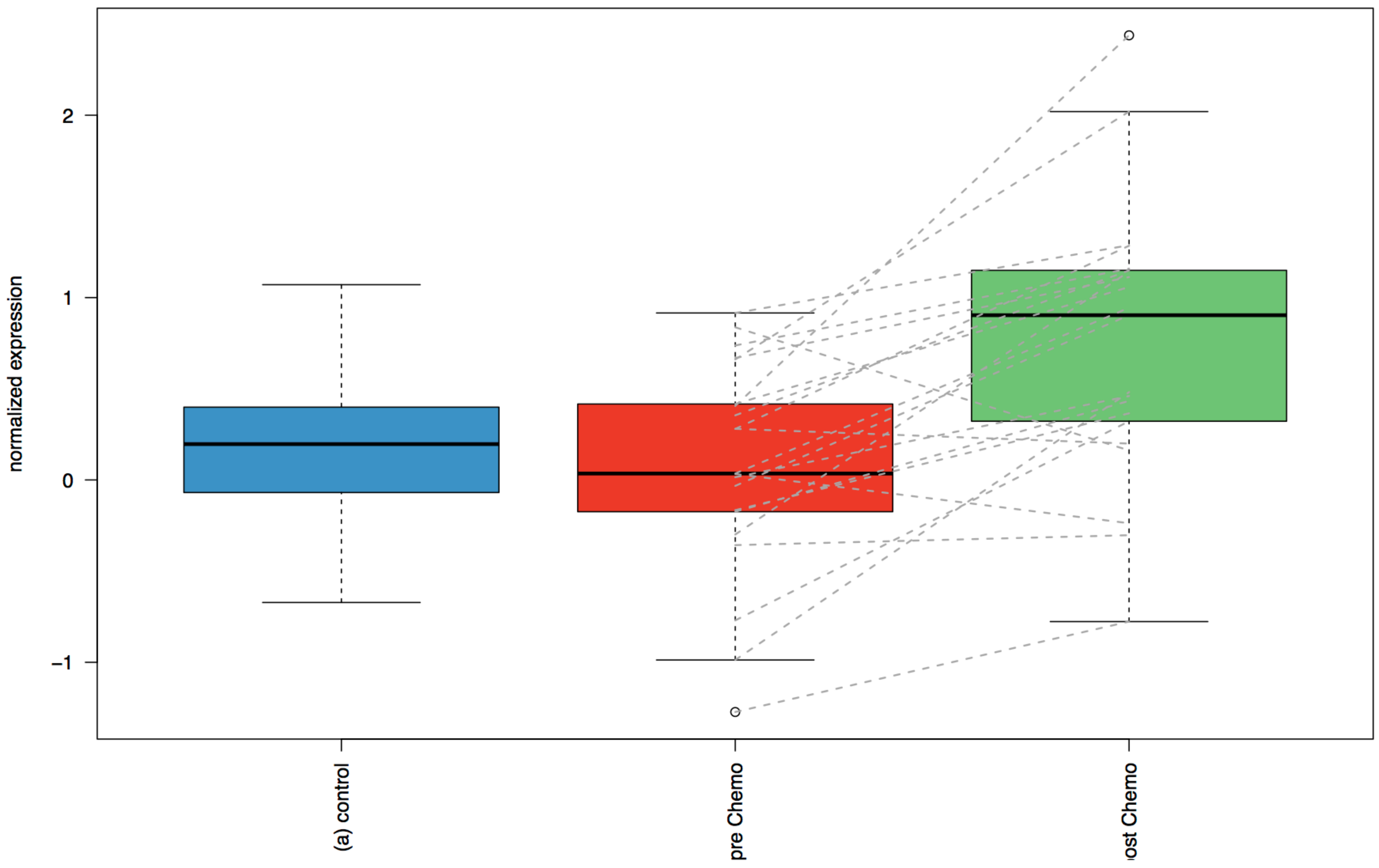
normalized expression
2
1
0
−1
(a) control
pre Chemo
ost Chemo

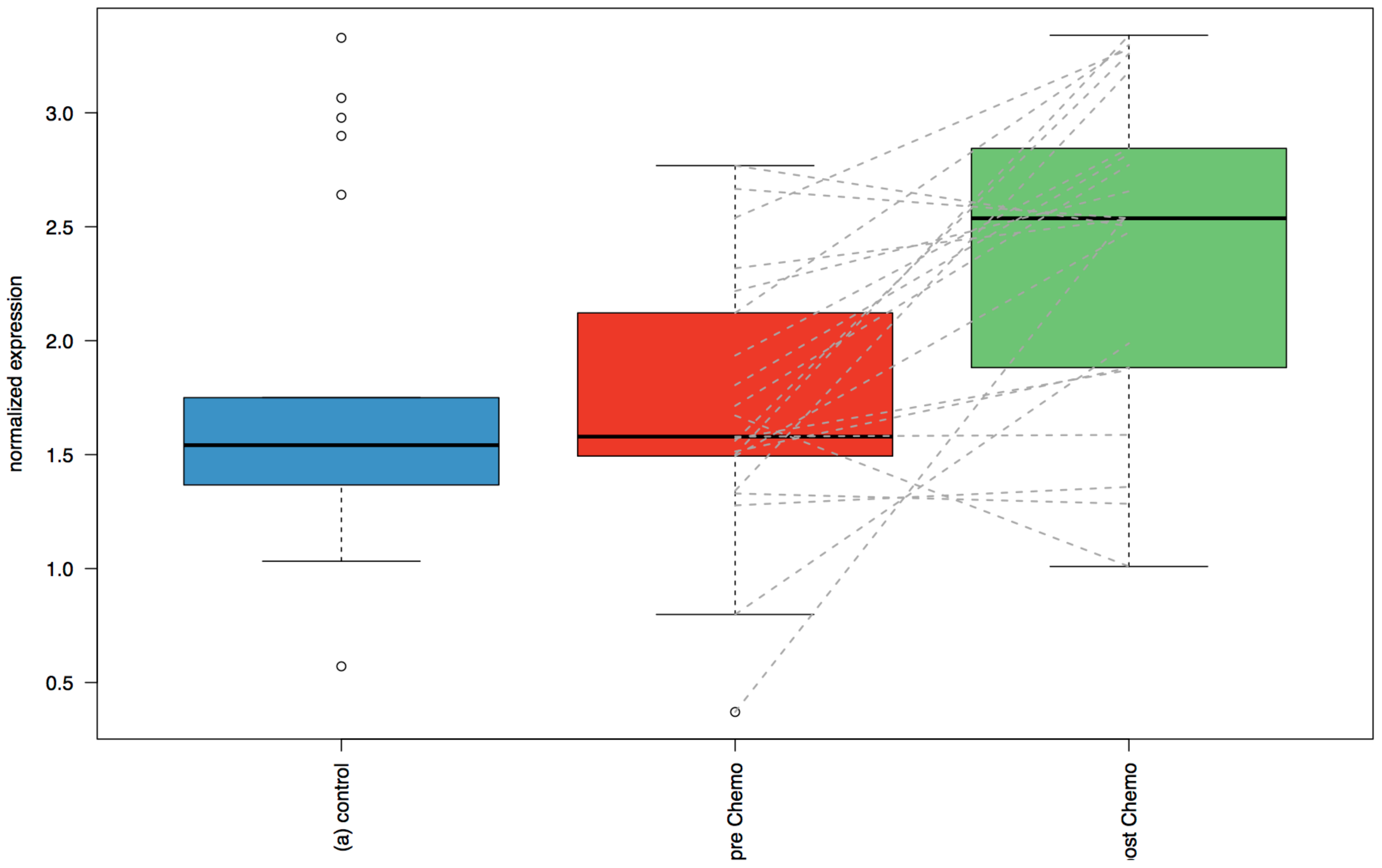
normalized expression
3.0
2.5
2.0
1.5
1.0
0.5
(a) control
pre Chemo
ost Chemo

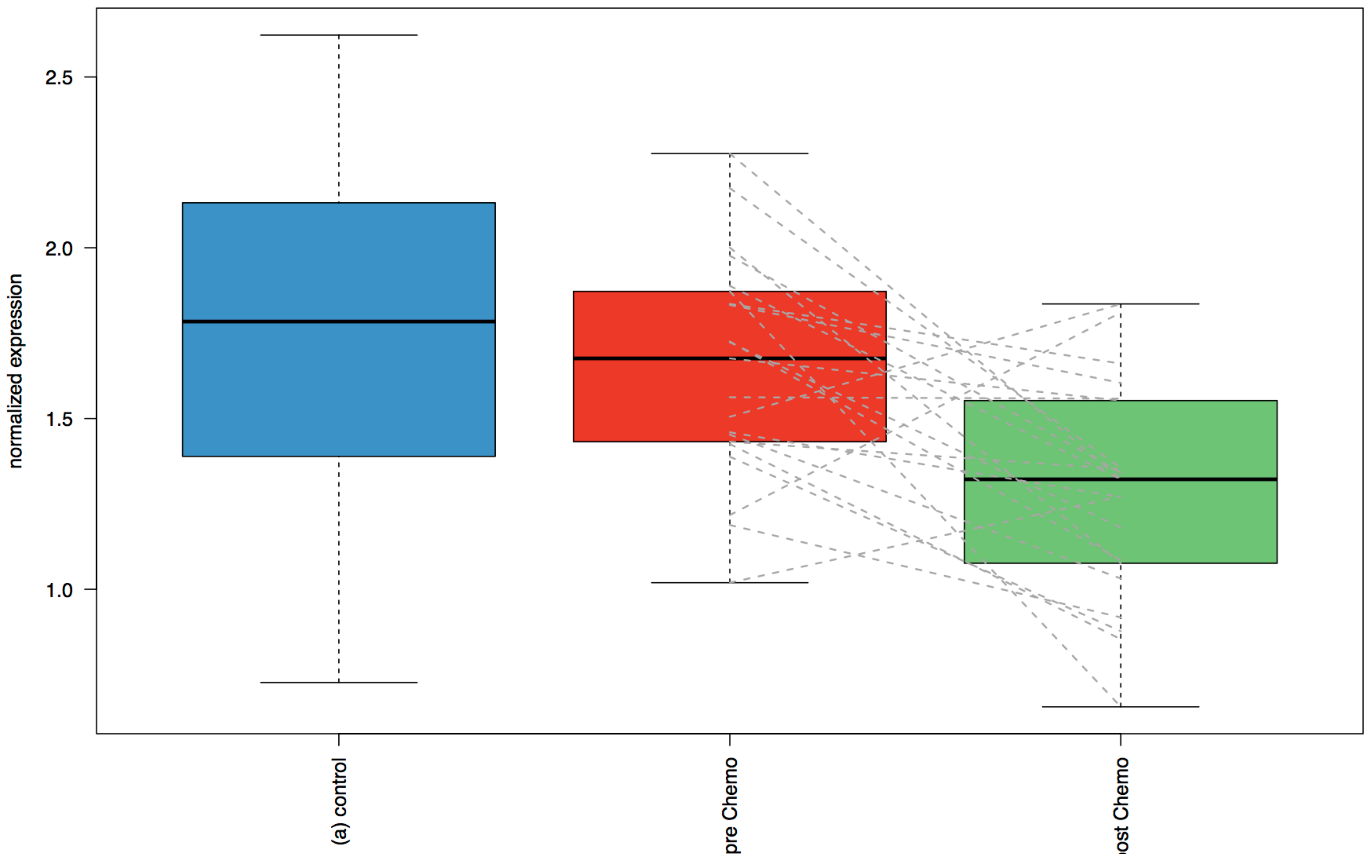
normalized expression
2.5
2.0
1.5
1.0
(a) control
pre Chemo
ost Chemo

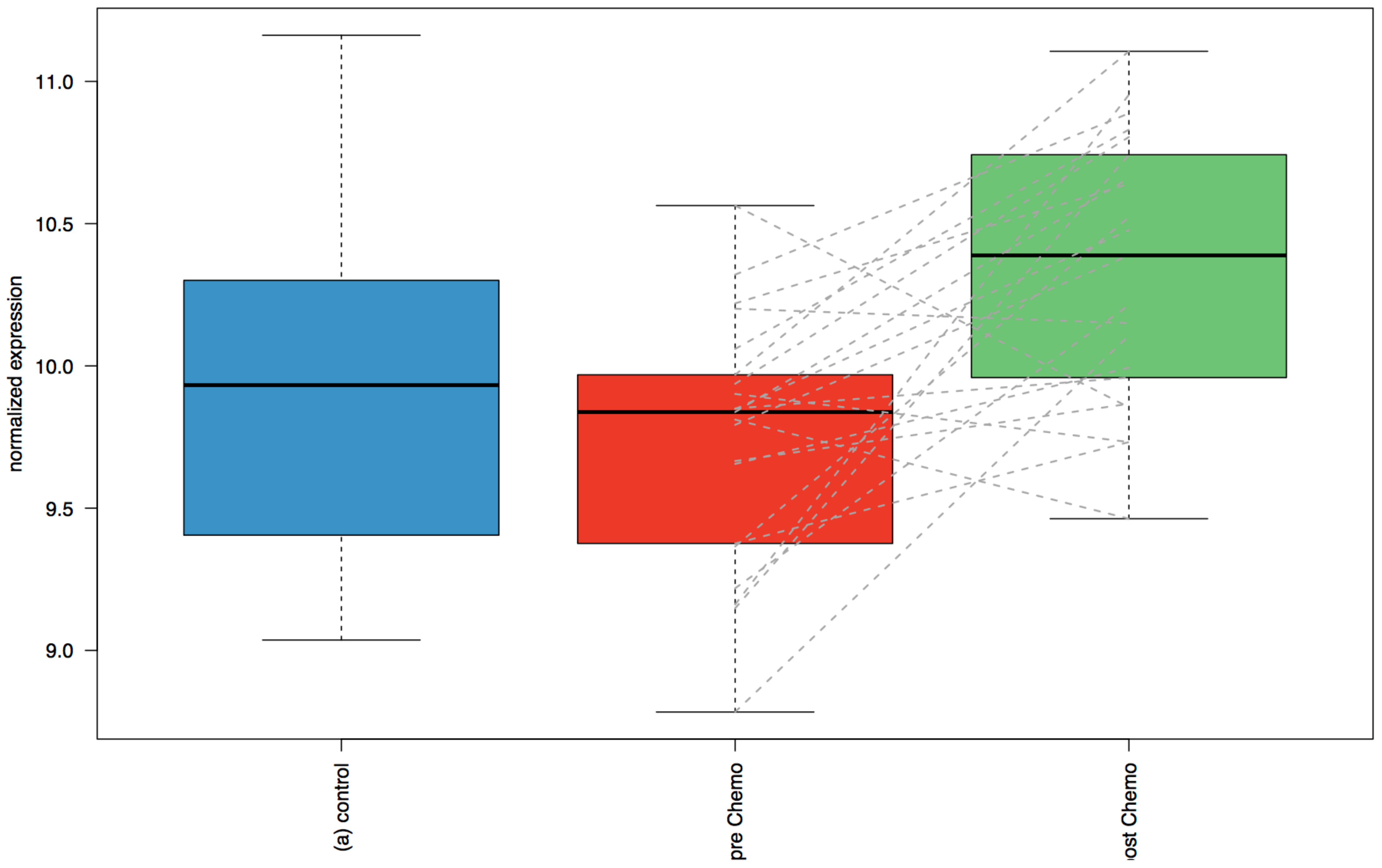
normalized expression
11.0
10.5
10.0
9.5
9.0
(a) control
pre Chemo
ost Chemo

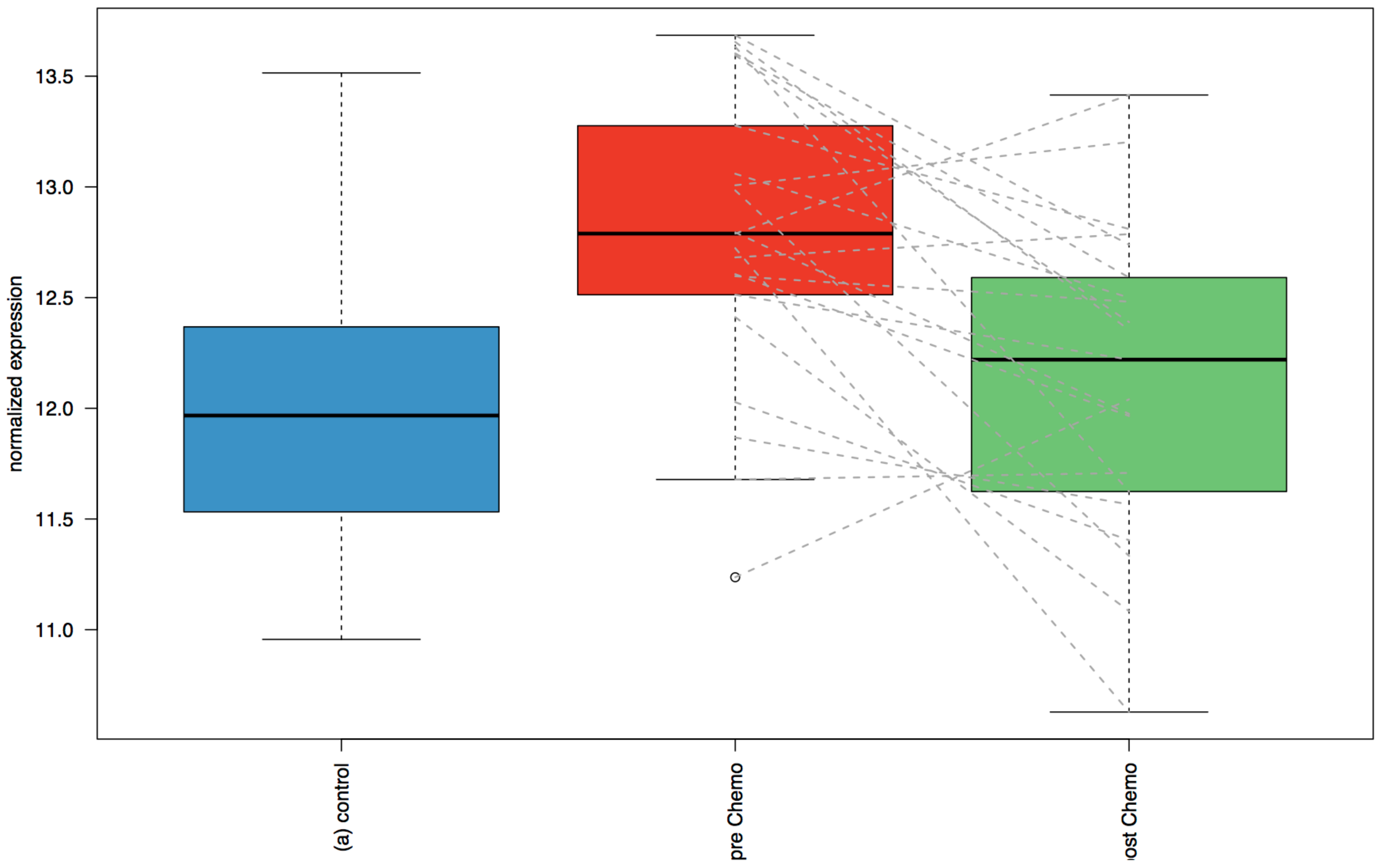

normalized expression
13.5
13.0
12.5
12.0
11.5
11.0
(a) control
pre Chemo
ost Chemo

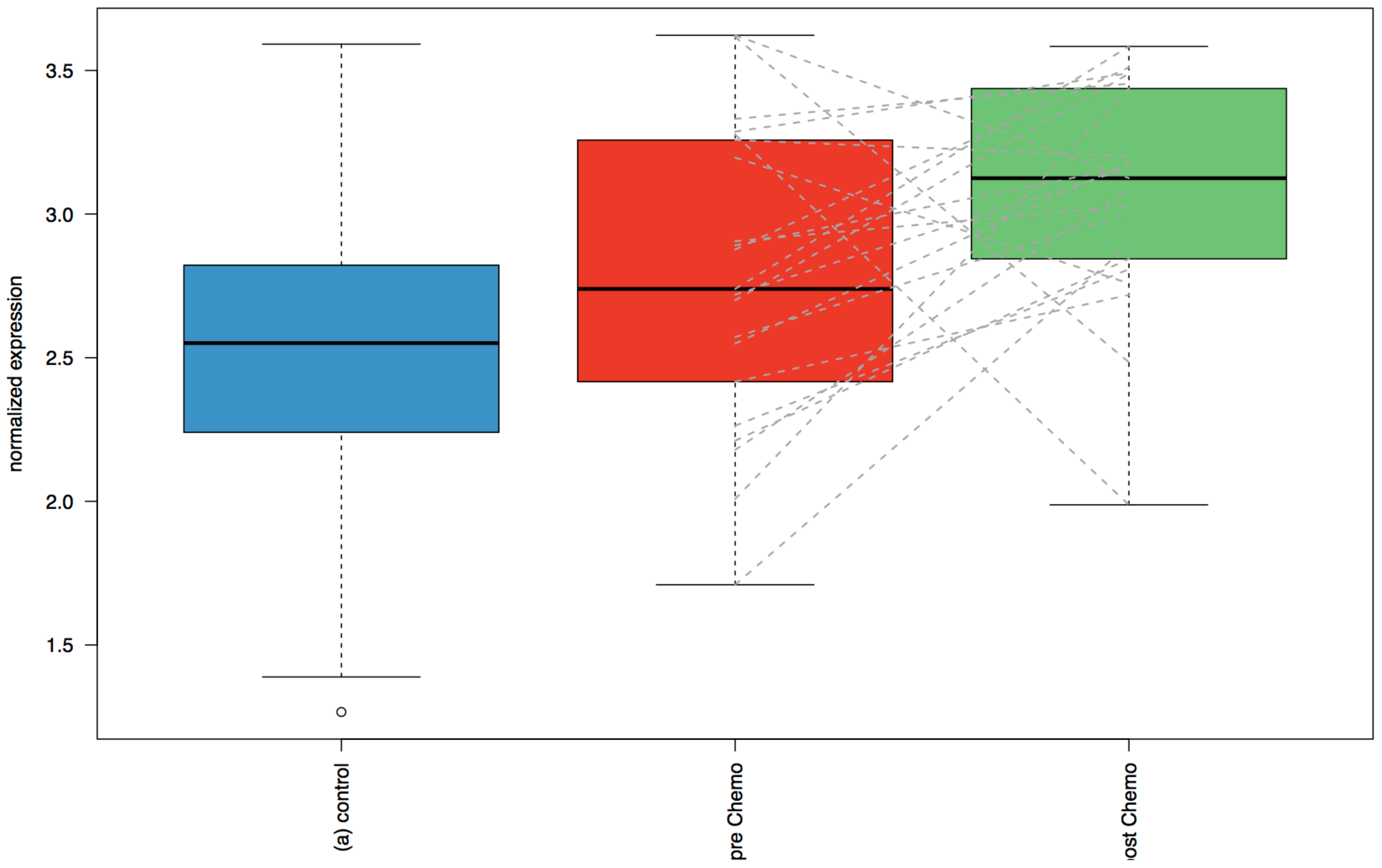
normalized expression
3.5
3.0
2.5
2.0
1.5
(a) control
pre Chemo
ost Chemo

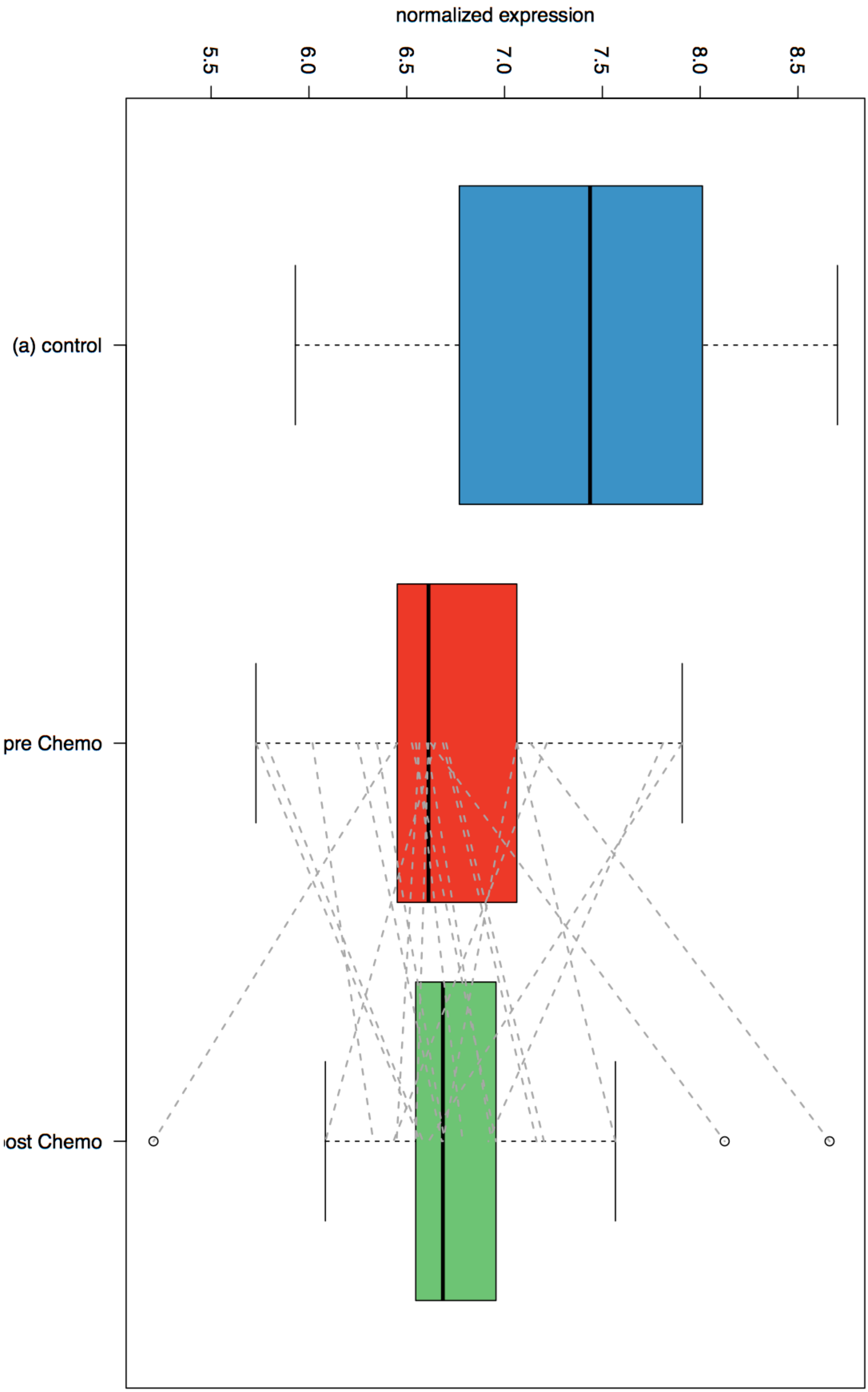
normalized expression
5.5
6.0
6.5
7.0
7.5
8.0
8.5
(a) control
pre Chemo
ost Chemo

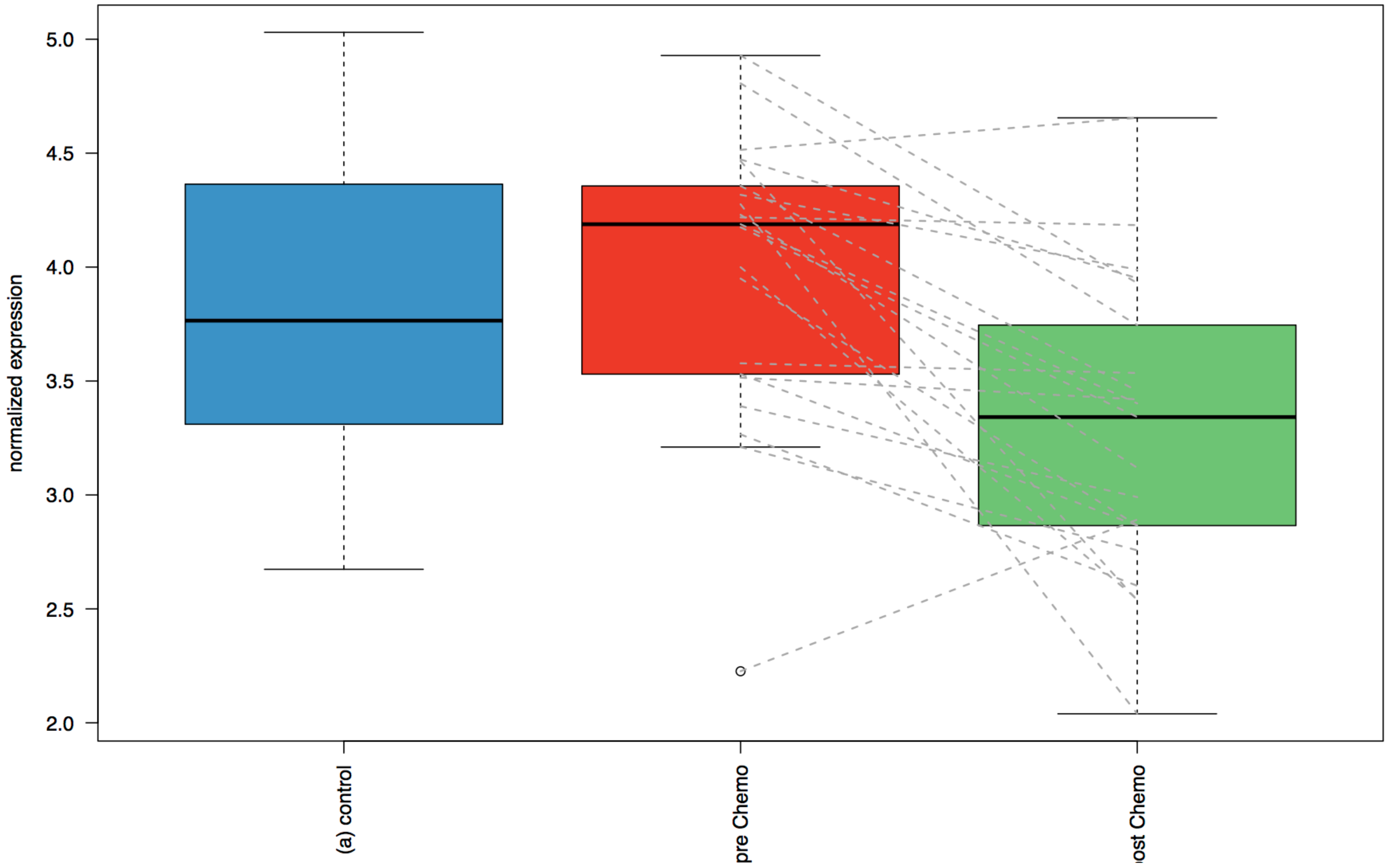
normalized expression
5.0
4.5
4.0
3.5
3.0
2.5
2.0
(a) control
pre Chemo
ost Chemo

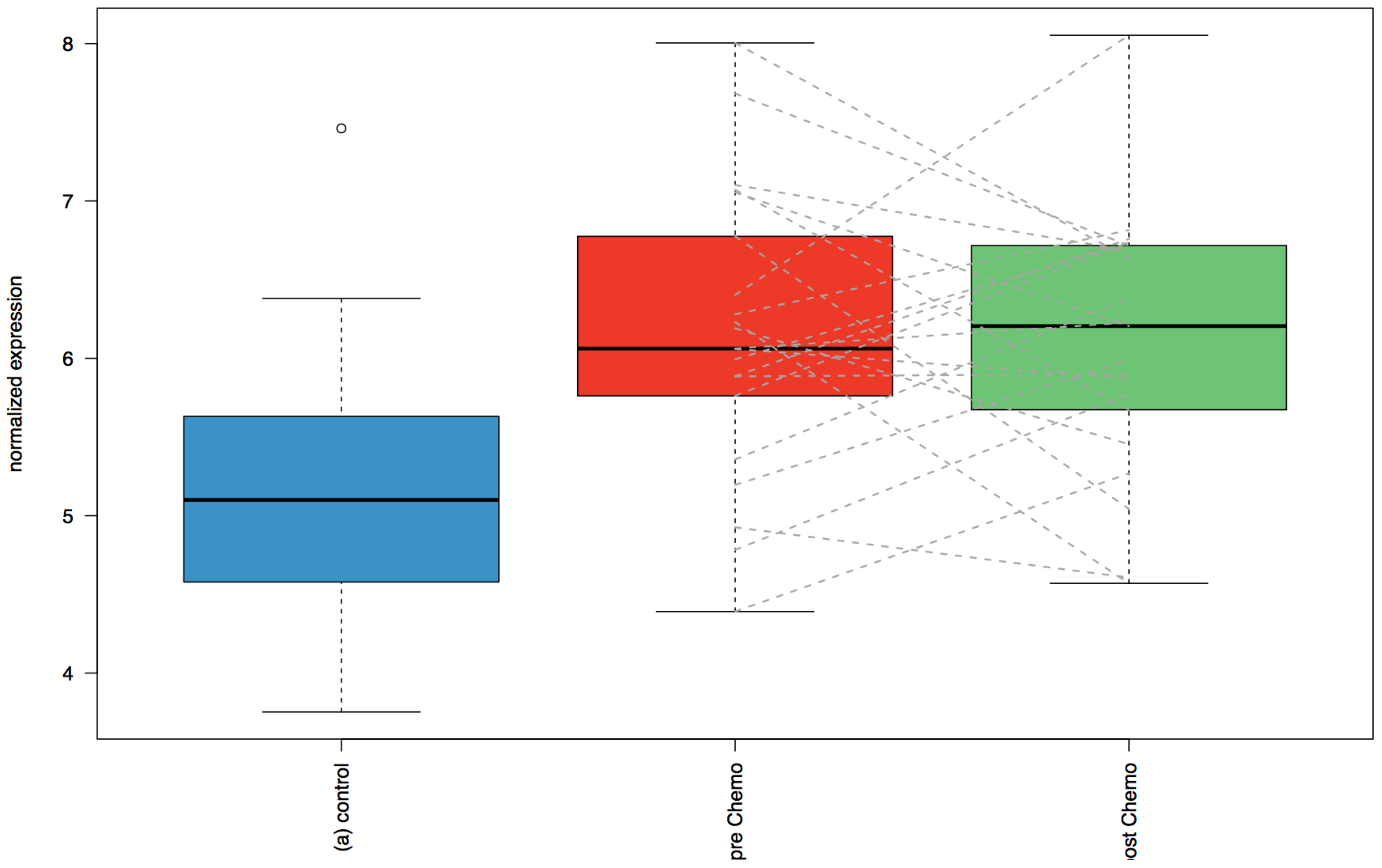
normalized expression
8
7
6
5
4
(a) control
pre Chemo
ost Chemo

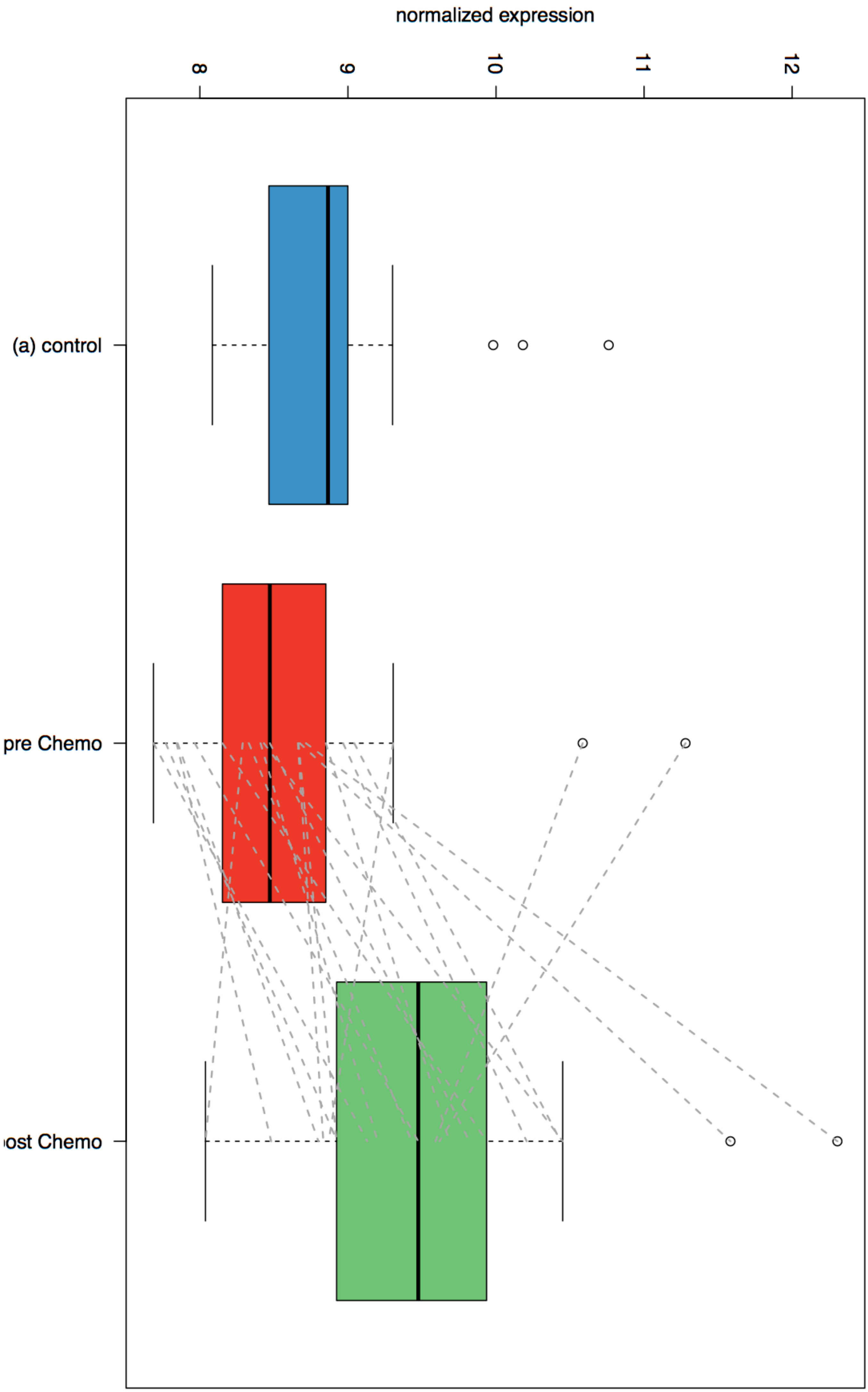
normalized expression
8
9
10
11
12
(a) control
pre Chemo
ost Chemo

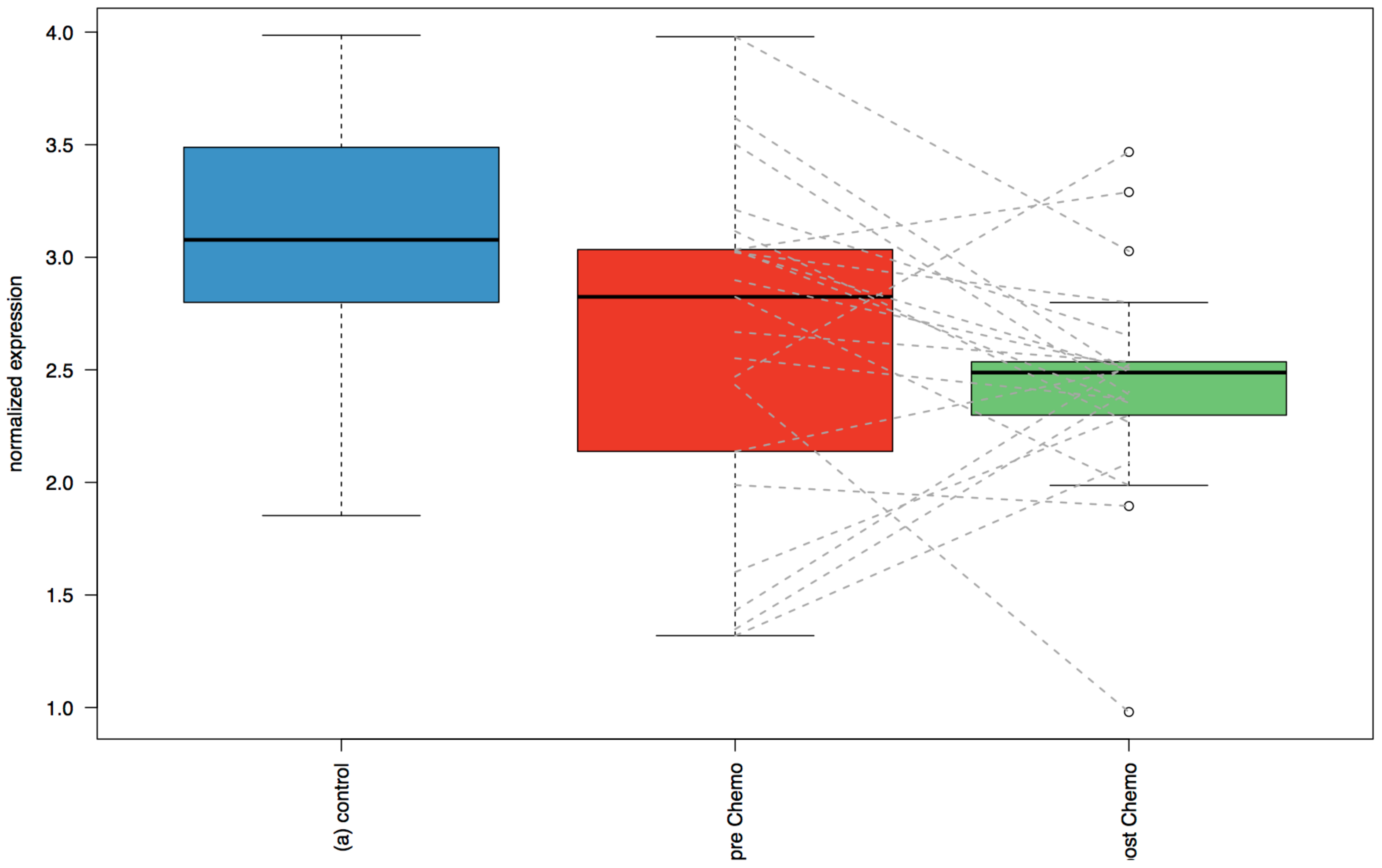
normalized expression
4.0
3.5
3.0
2.5
2.0
1.5
1.0
(a) control
pre Chemo
ost Chemo

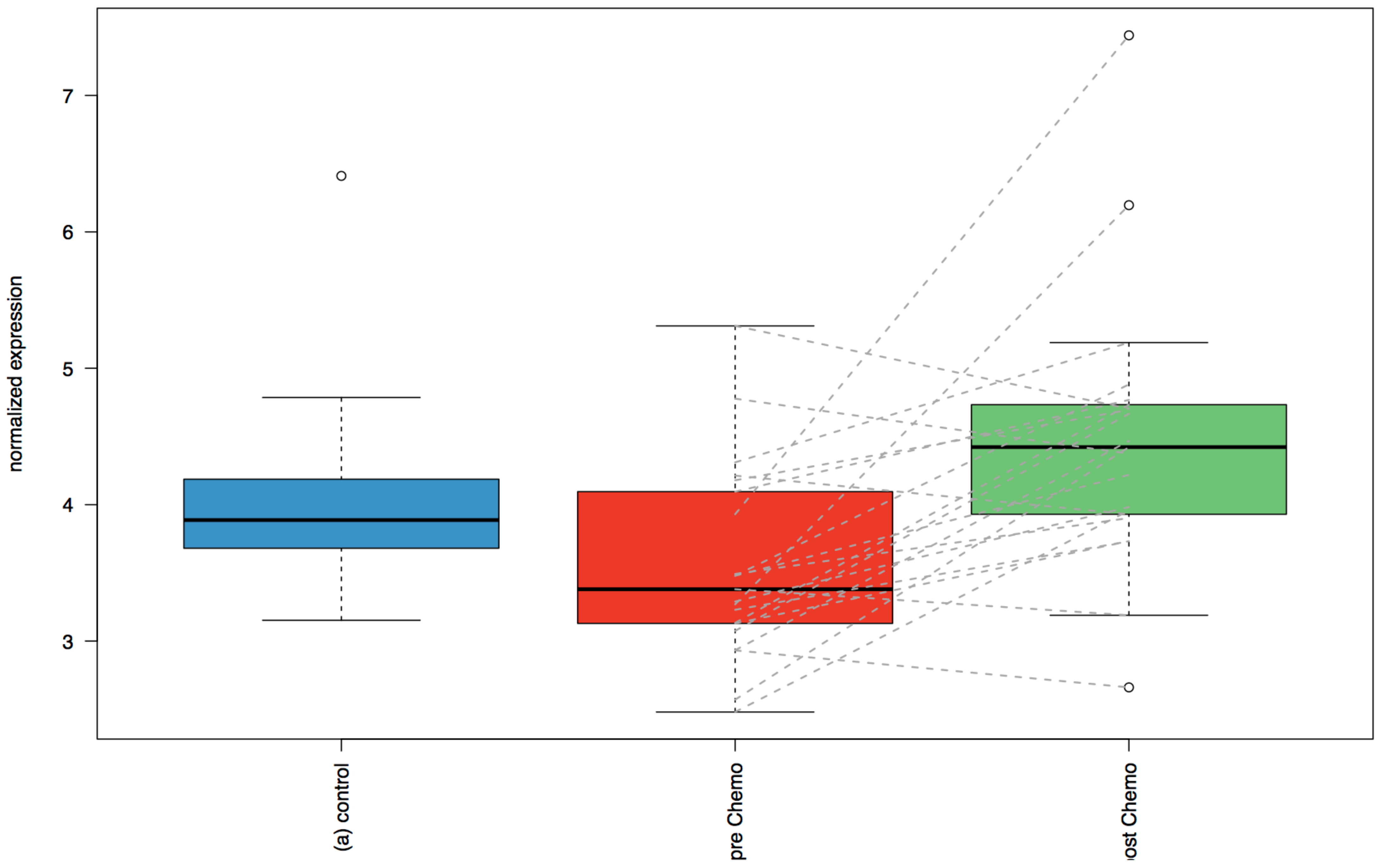
normalized expression
7
6
5
4
3
(a) control
pre Chemo
ost Chemo

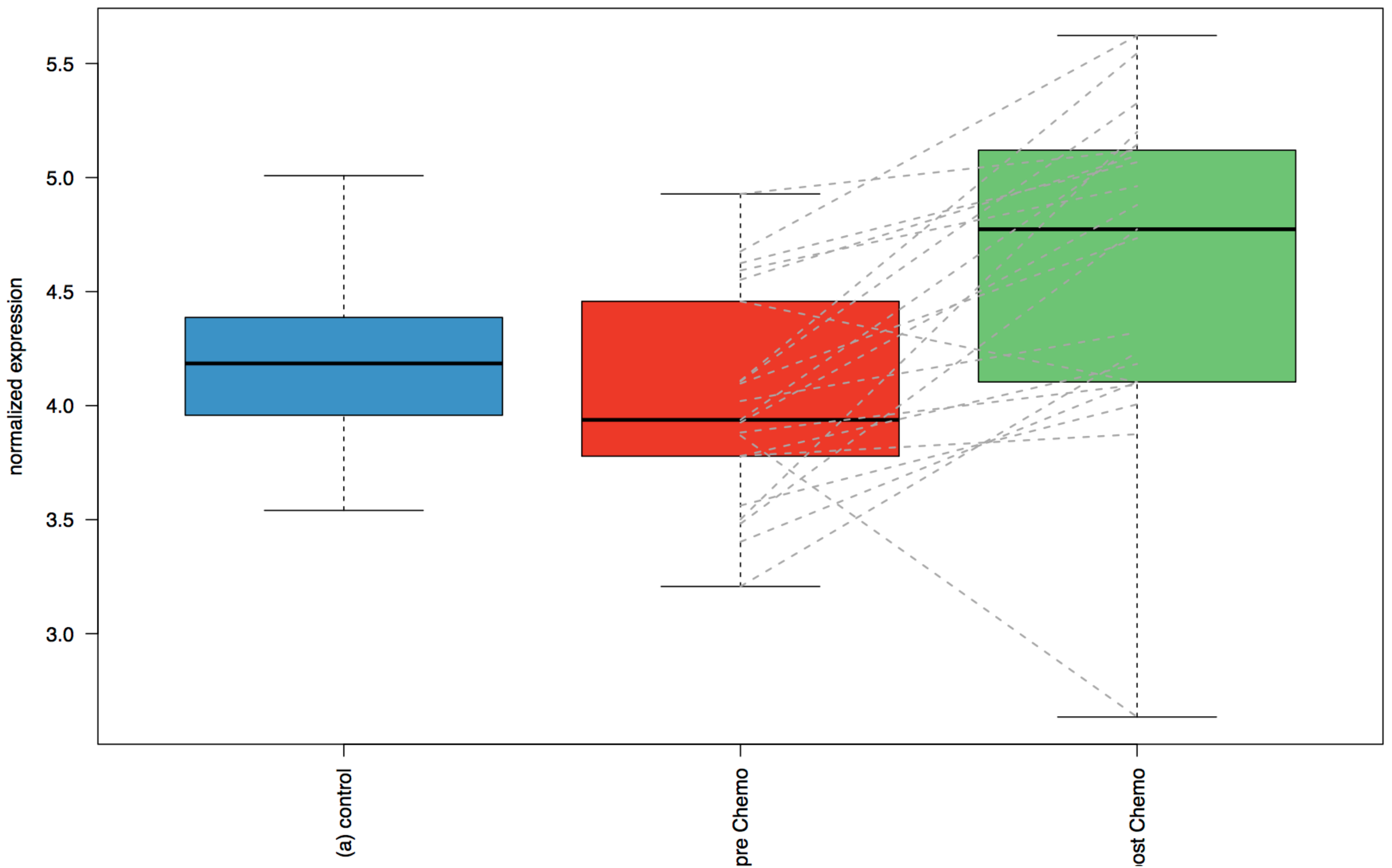
normalized expression
5.5
5.0
4.5
4.0
3.5
3.0
(a) control
pre Chemo
ost Chemo

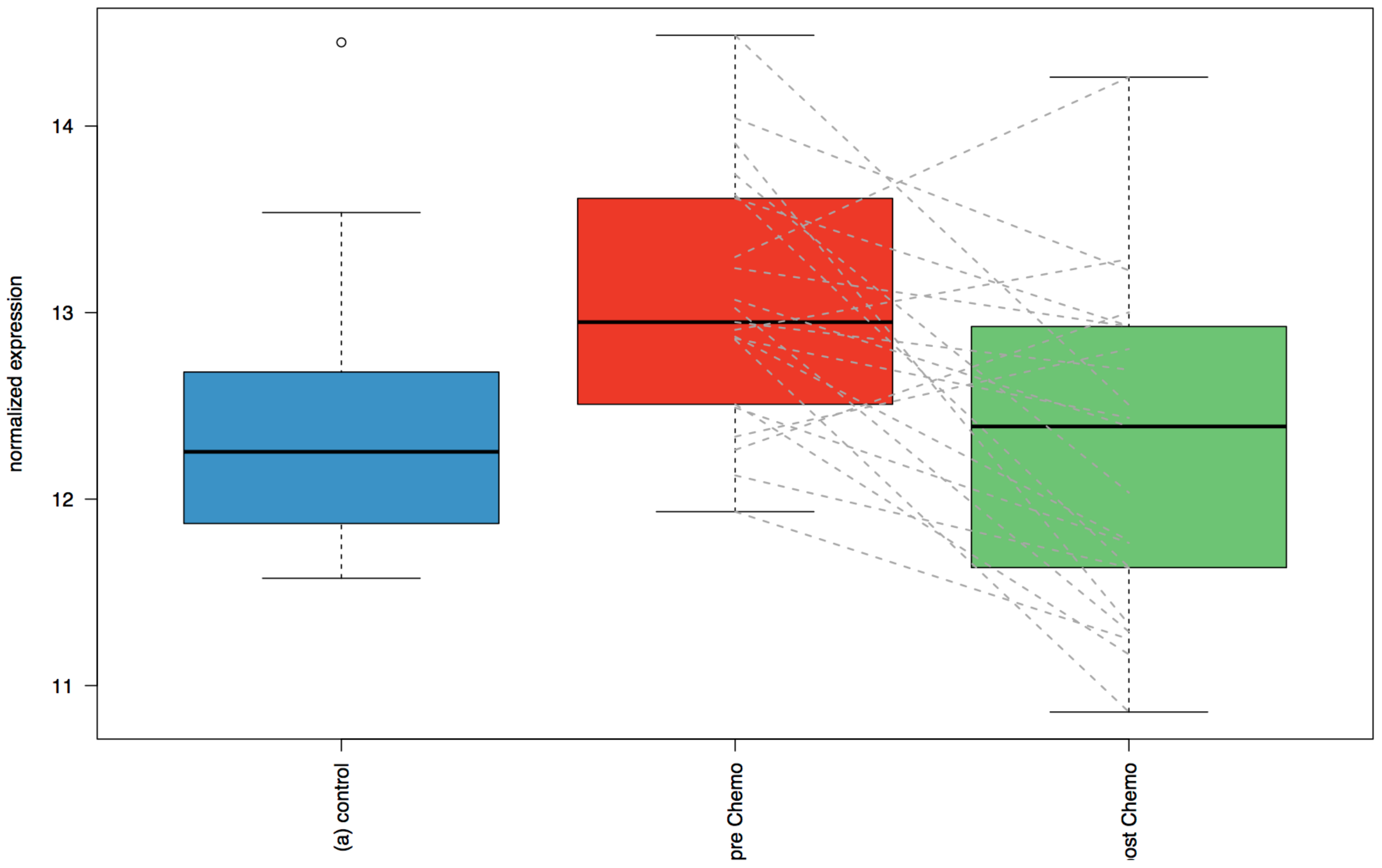
normalized expression
14
13
12
11
(a) control
pre Chemo
ost Chemo

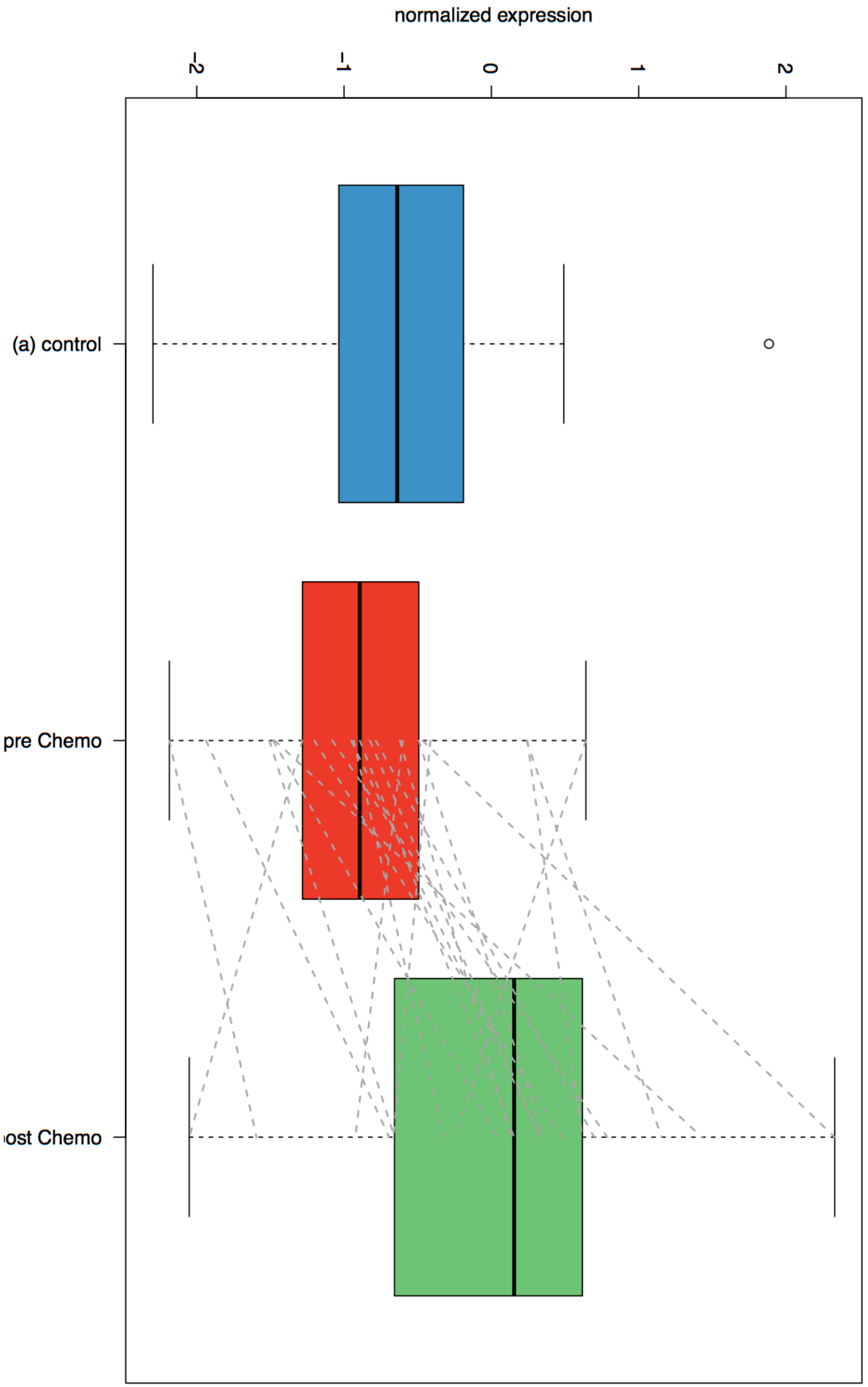

normalized expression
-2
-1
0
1
2
(a) control
pre Chemo
ost Chemo

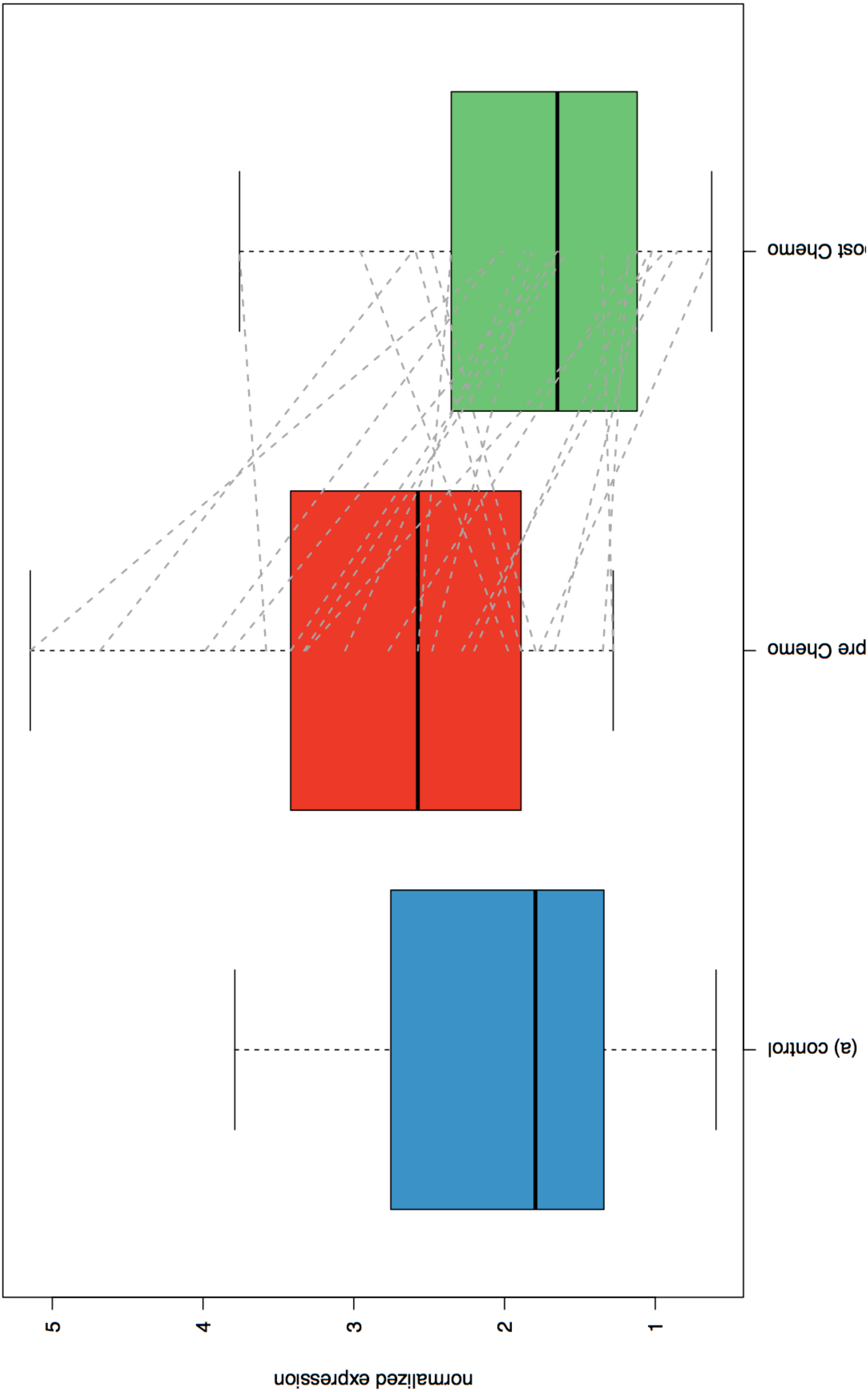
(a) control
pre Chemo
ost Chemo
normalized expression
1
2
3
4
5

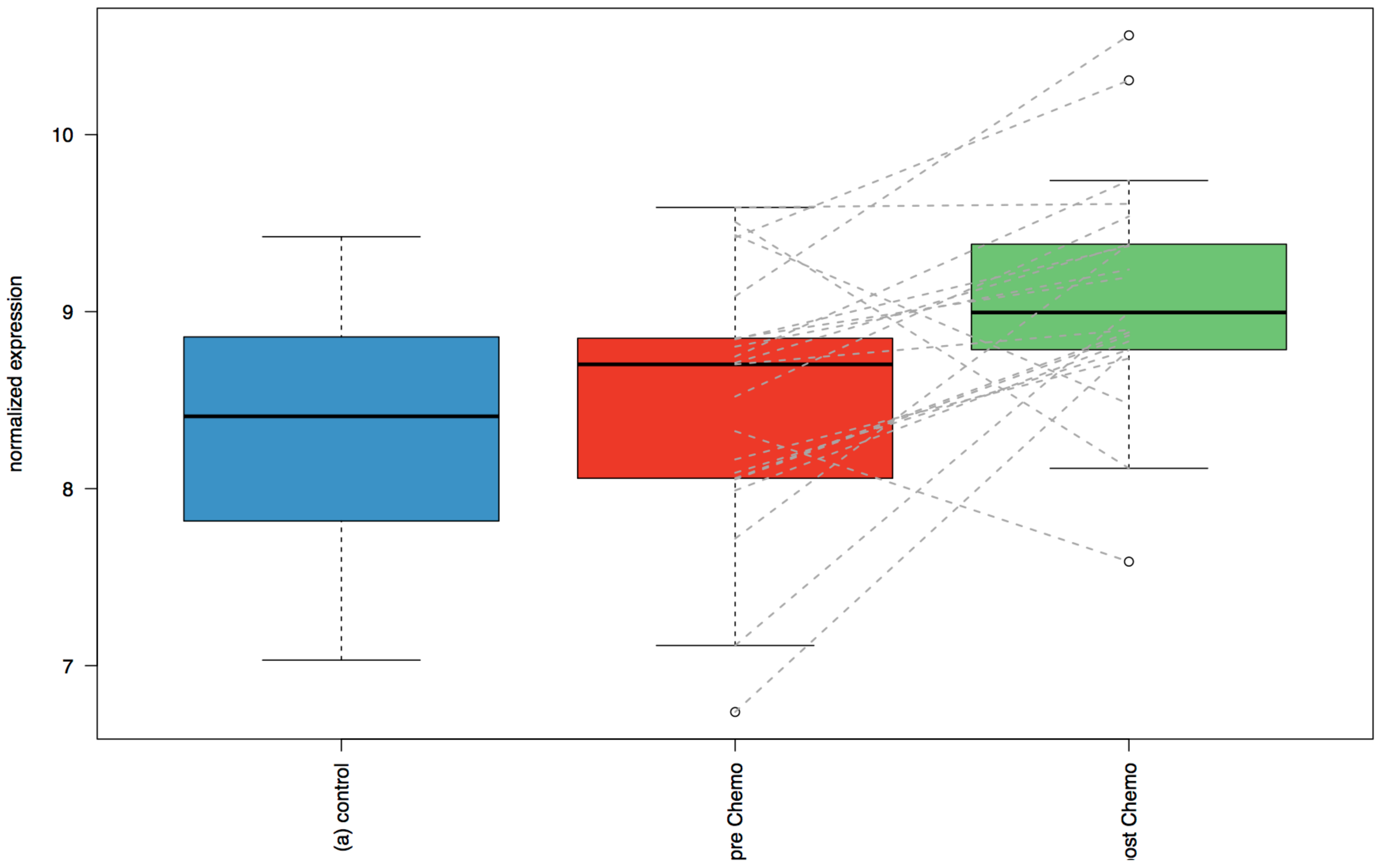
normalized expression
10
9
8
7
(a) control
pre Chemo
ost Chemo

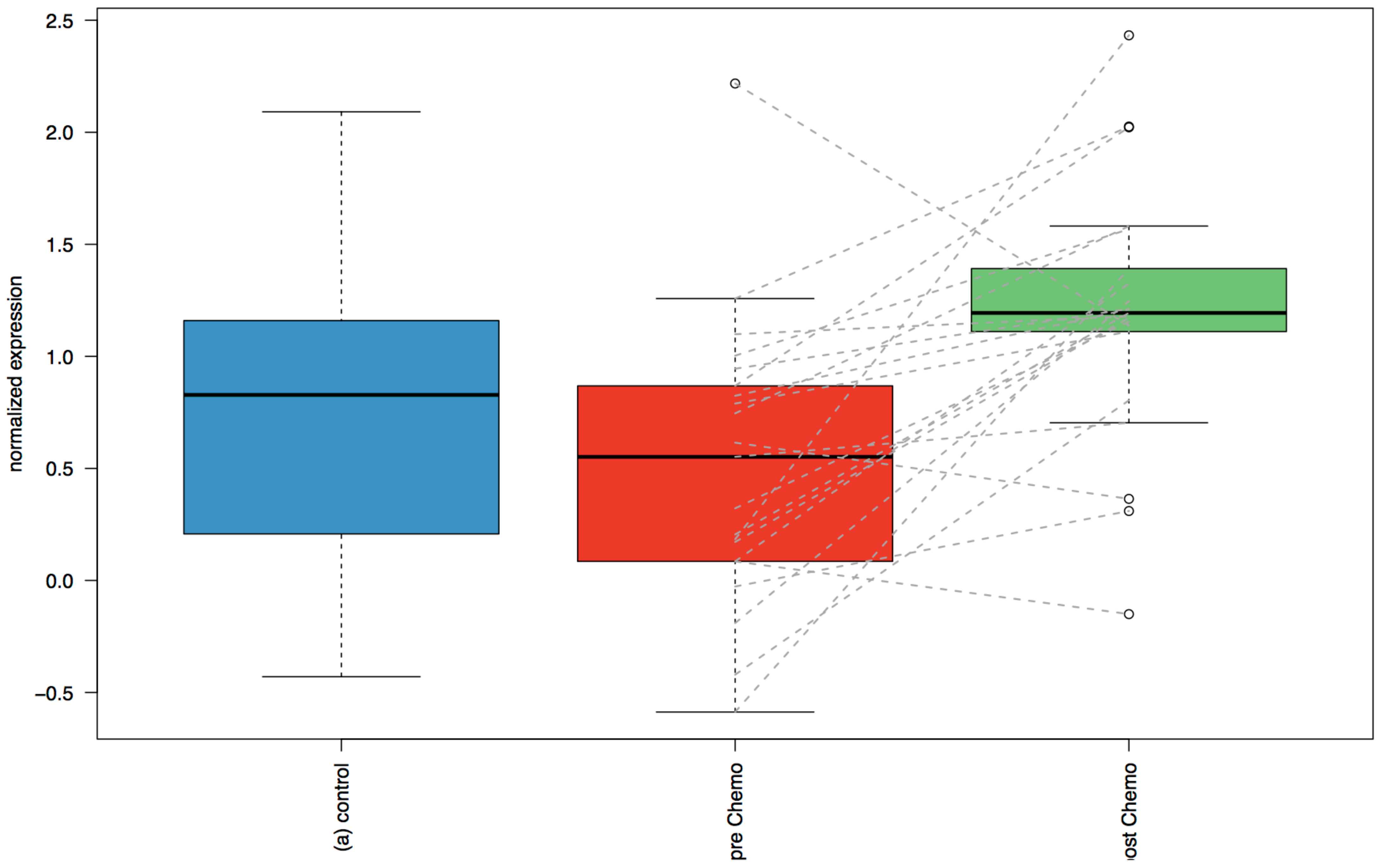
normalized expression
2.5
2.0
1.5
1.0
0.5
0.0
−0.5
(a) control
pre Chemo
ost Chemo

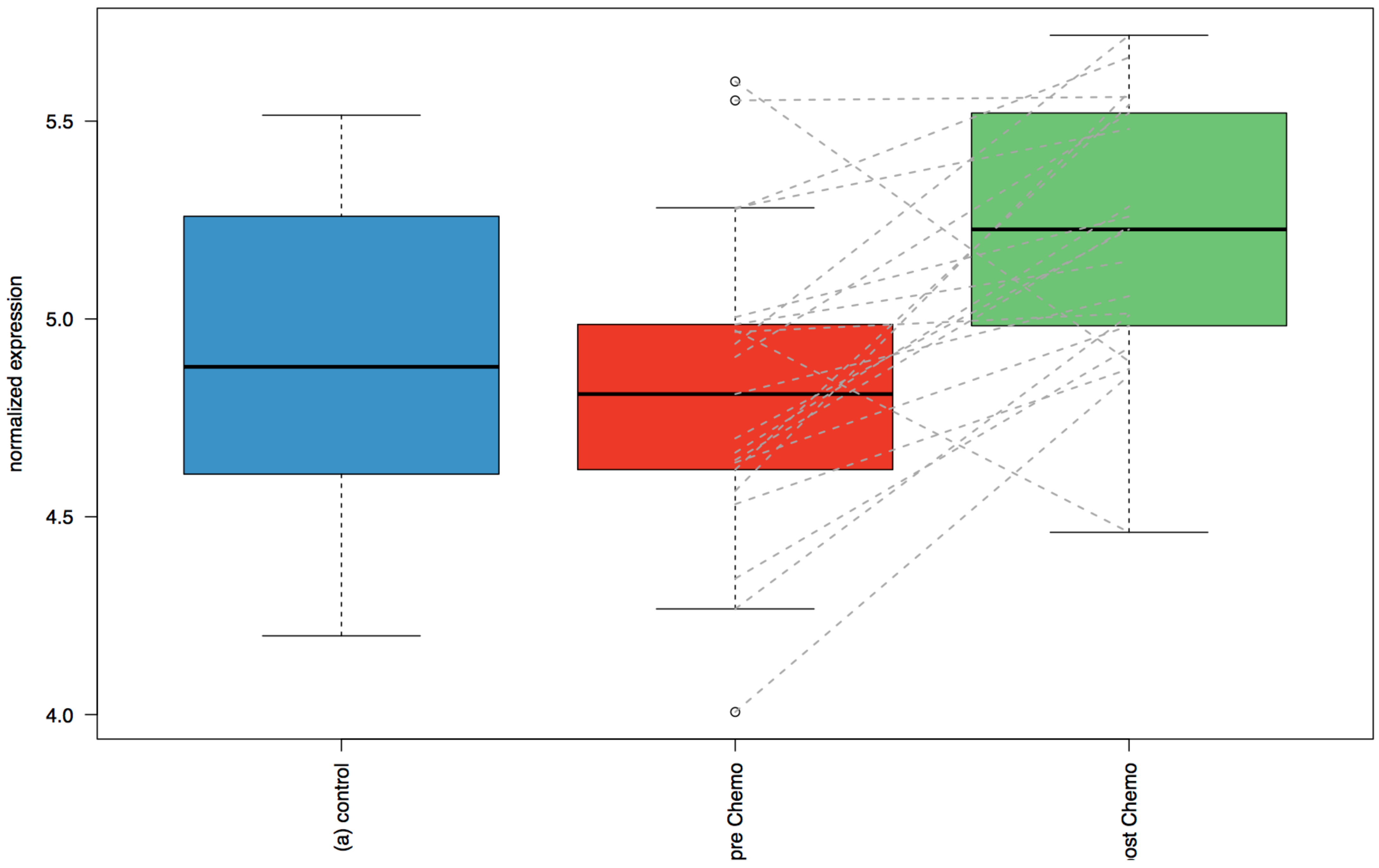

normalized expression
5.5
5.0
4.5
4.0
(a) control
pre Chemo
ost Chemo

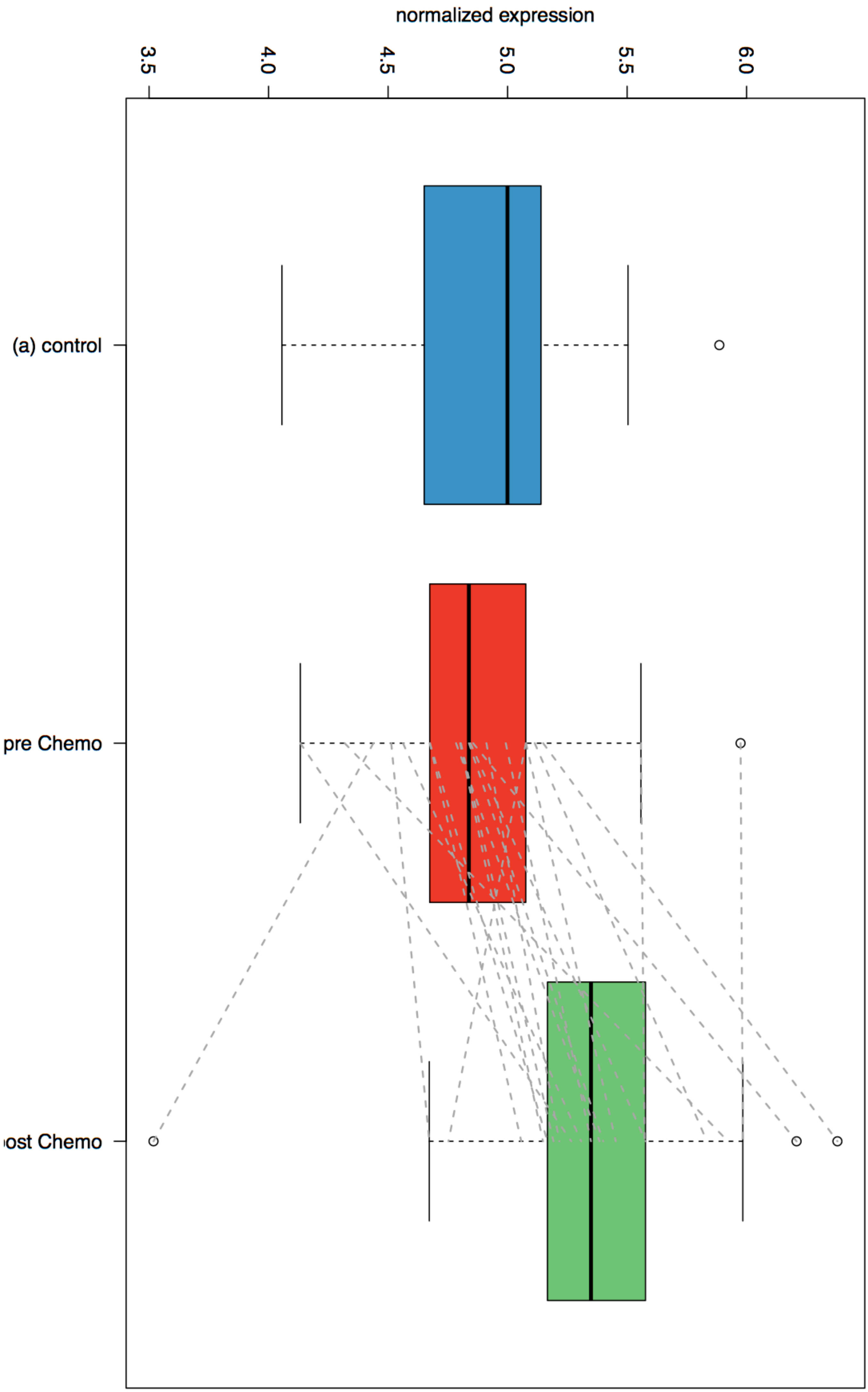
normalized expression
3.5
4.0
4.5
5.0
5.5
6.0
(a) control
pre Chemo
ost Chemo

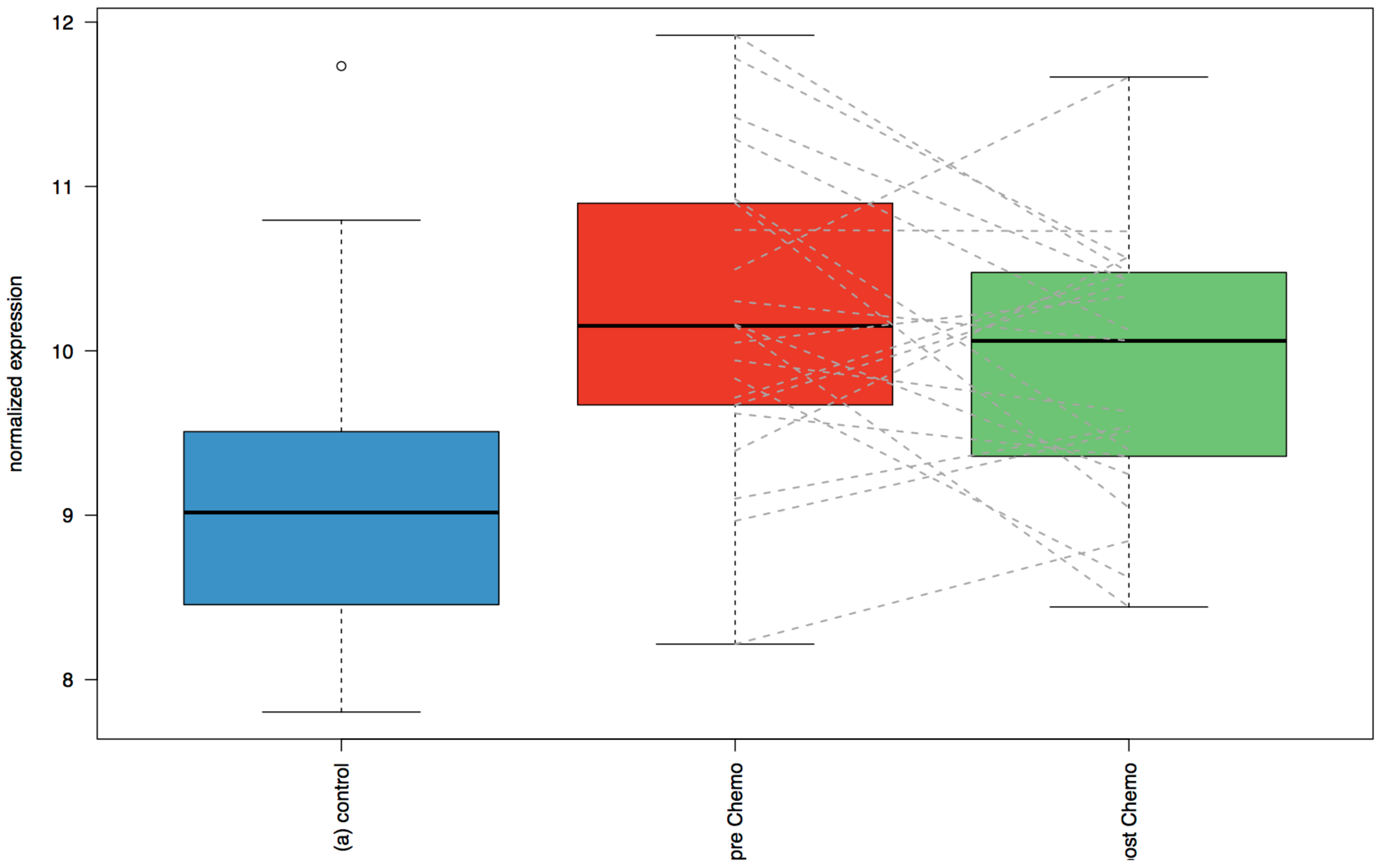
normalized expression
12
11
10
9
8
(a) control
pre Chemo
ost Chemo

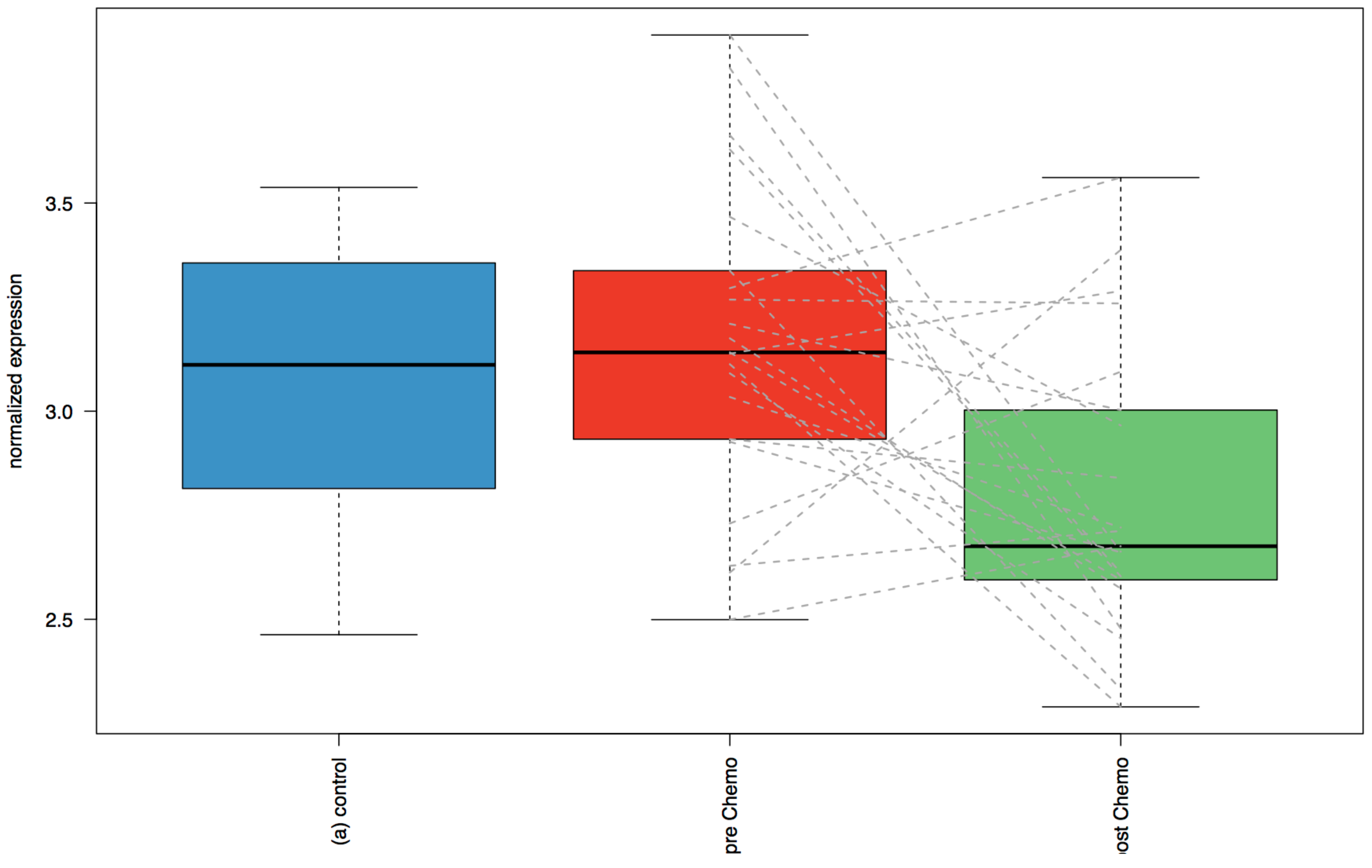
normalized expression
3.5
3.0
2.5
(a) control
pre Chemo
ost Chemo

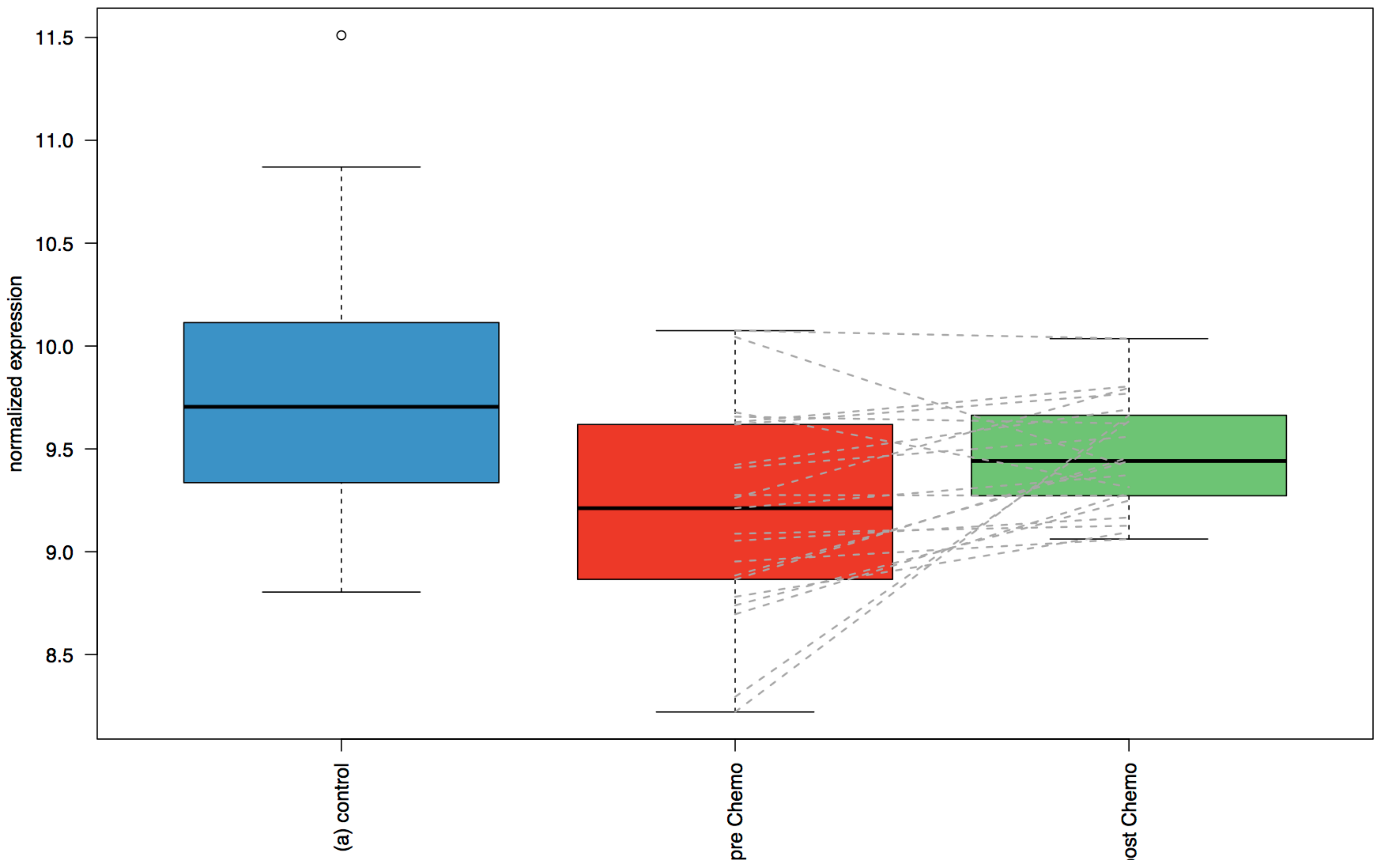
normalized expression
11.5
11.0
10.5
10.0
9.5
9.0
8.5
(a) control
pre Chemo
ost Chemo

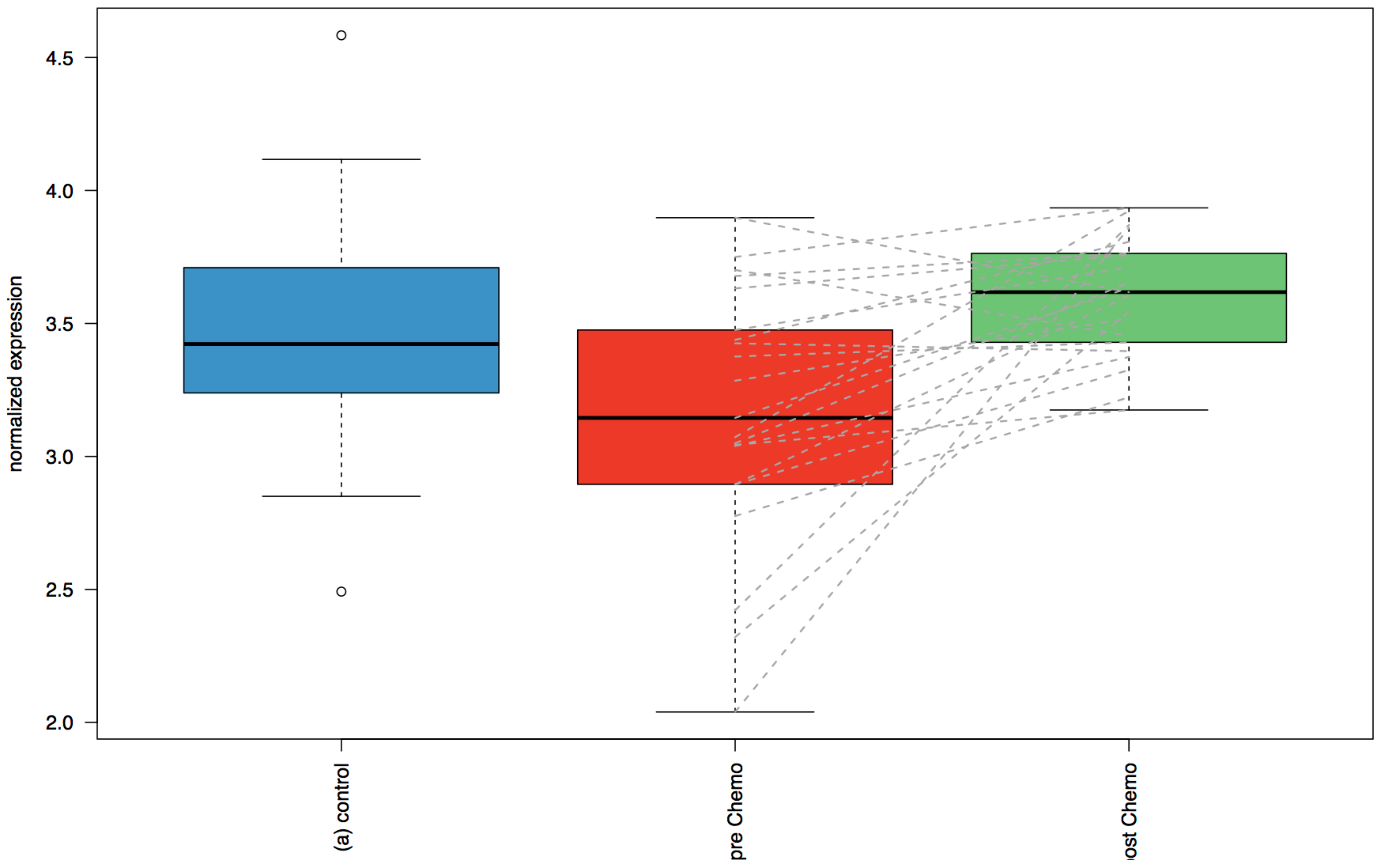
normalized expression
4.5
4.0
3.5
3.0
2.5
2.0
(a) control
pre Chemo
ost Chemo

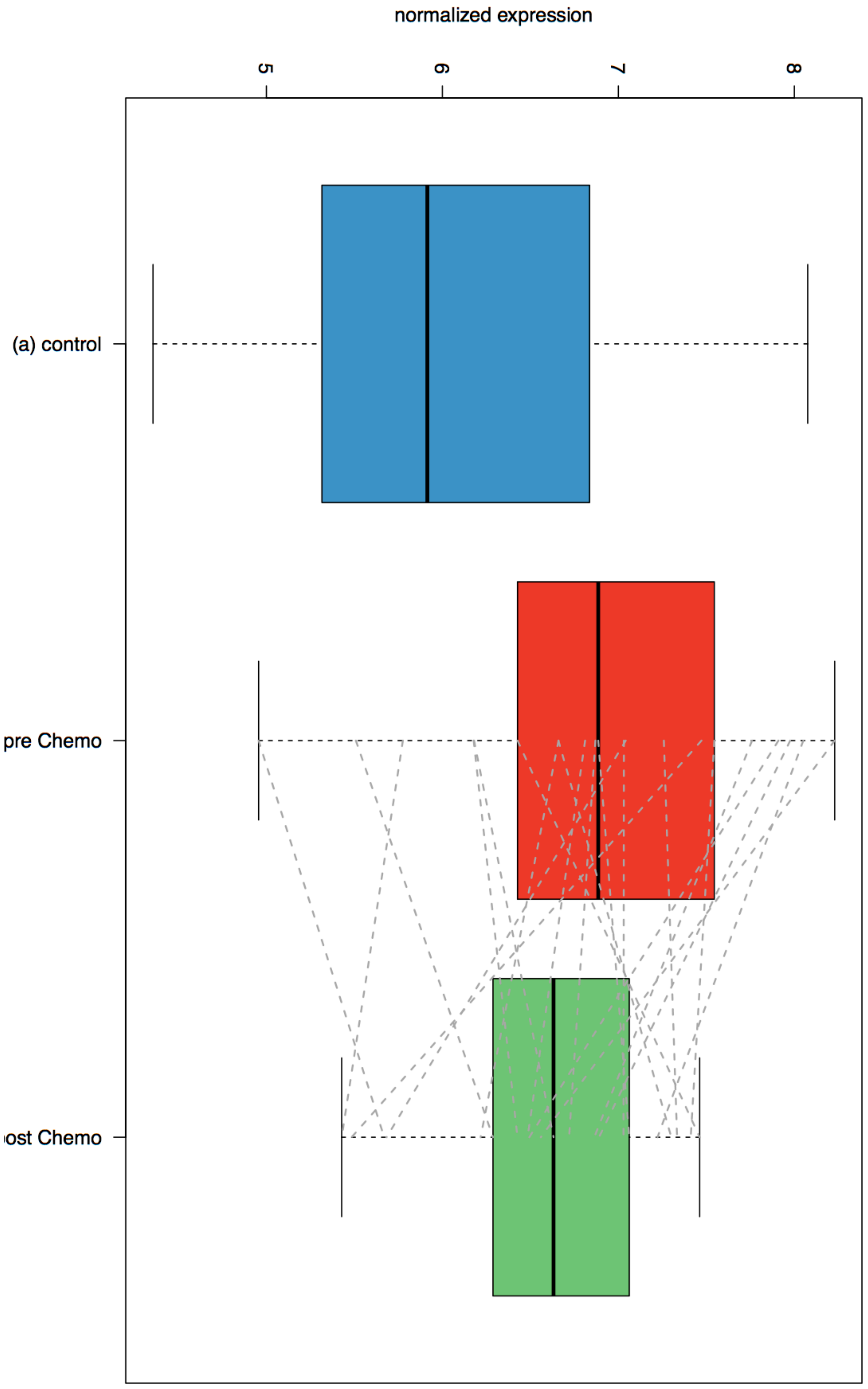
normalized expression
5
6
7
8
(a) control
pre Chemo
ost Chemo

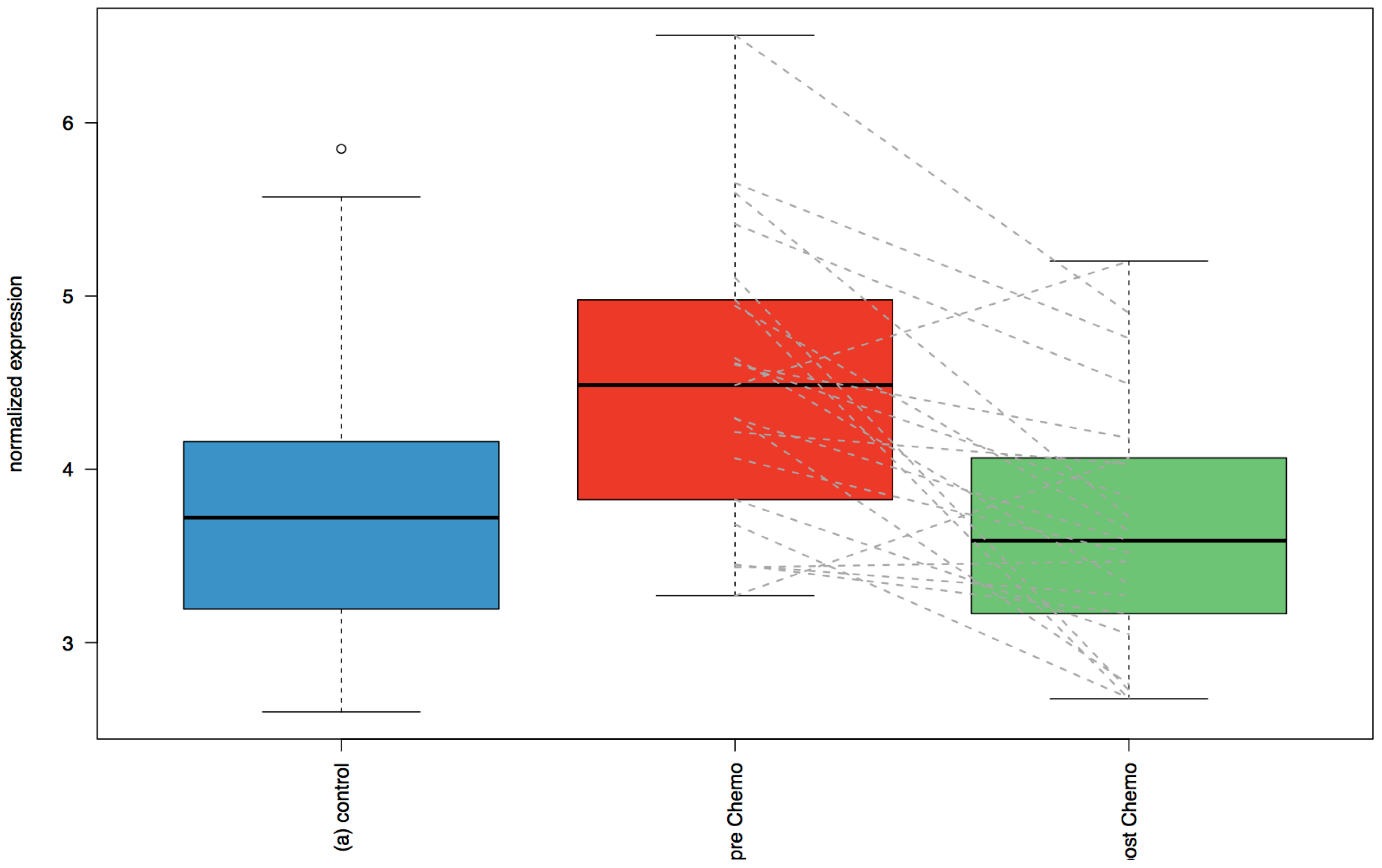
normalized expression
6
5
4
3
(a) control
pre Chemo
ost Chemo

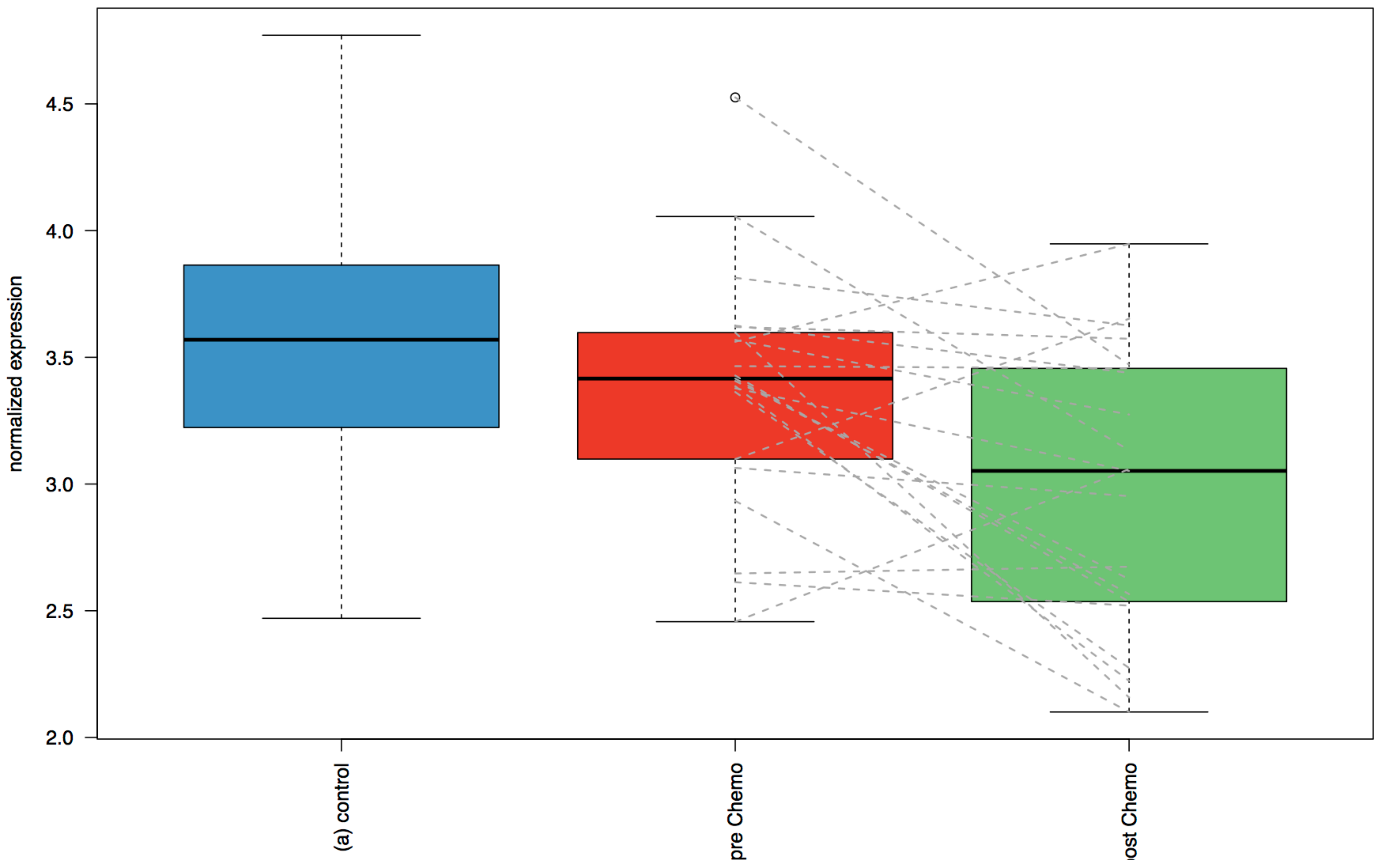
normalized expression
4.5
4.0
3.5
3.0
2.5
2.0
(a) control
pre Chemo
ost Chemo

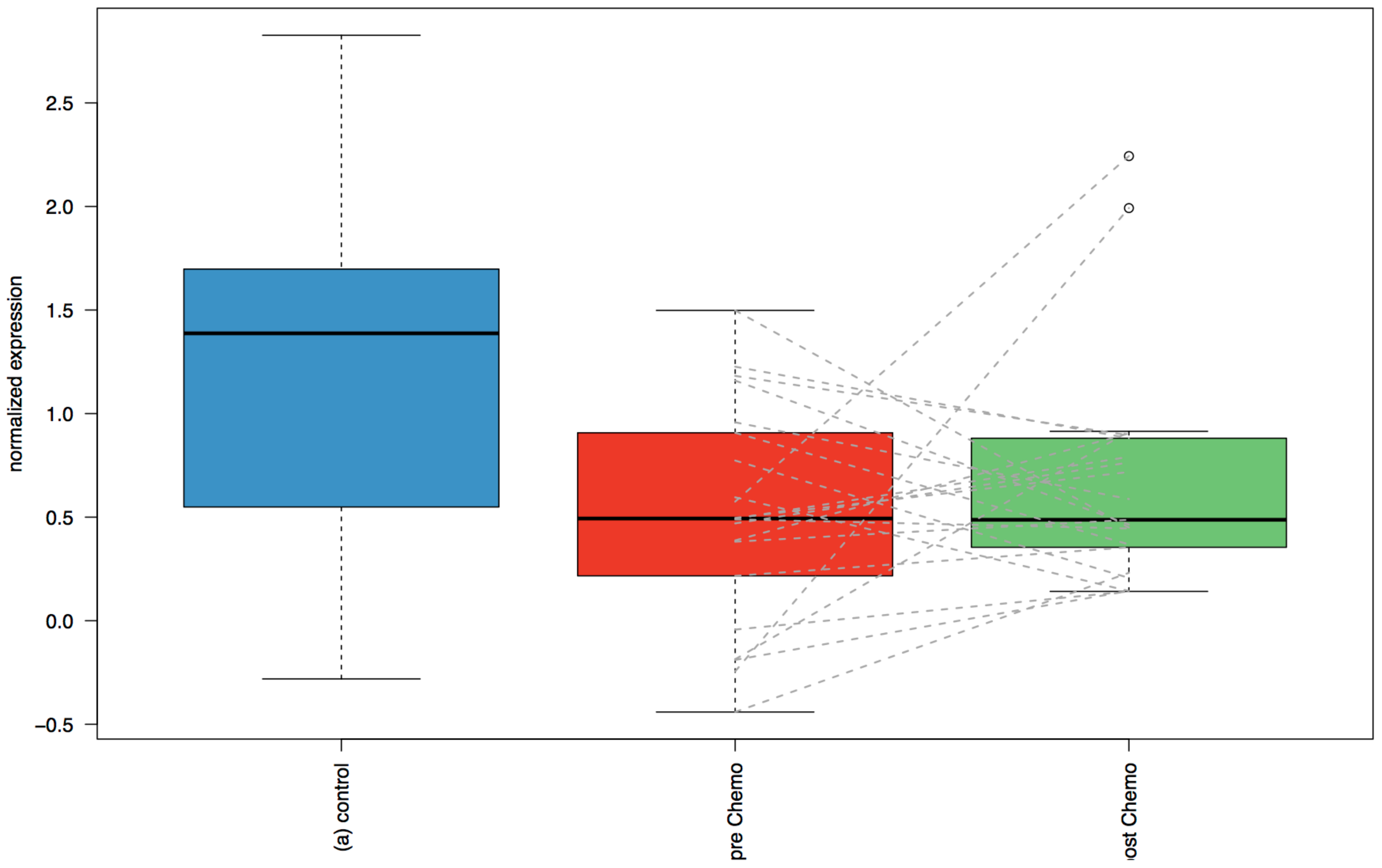
normalized expression
2.5
2.0
1.5
1.0
0.5
0.0
−0.5
(a) control
pre Chemo
ost Chemo

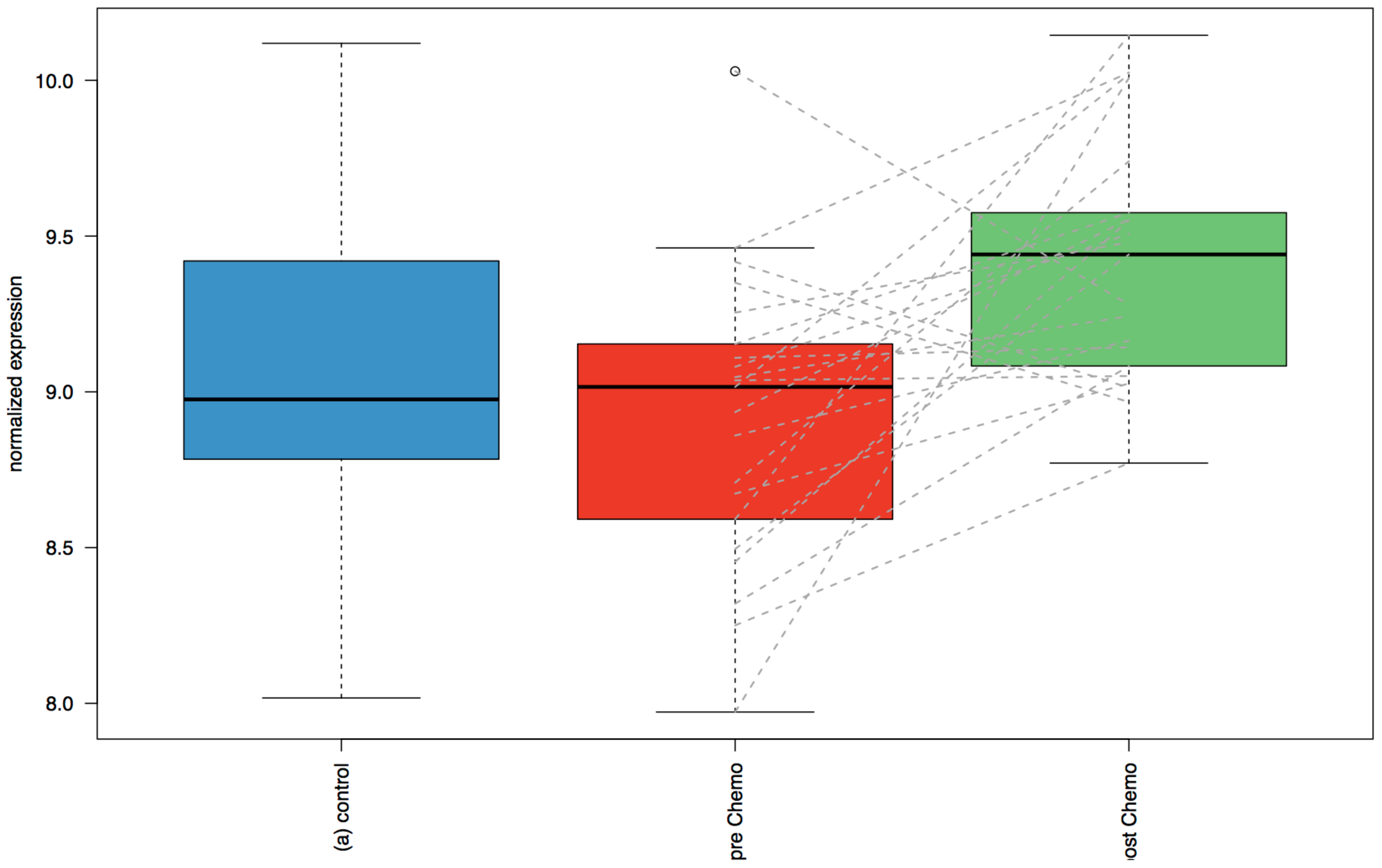
normalized expression
10.0
9.5
9.0
8.5
8.0
(a) control
pre Chemo
ost Chemo

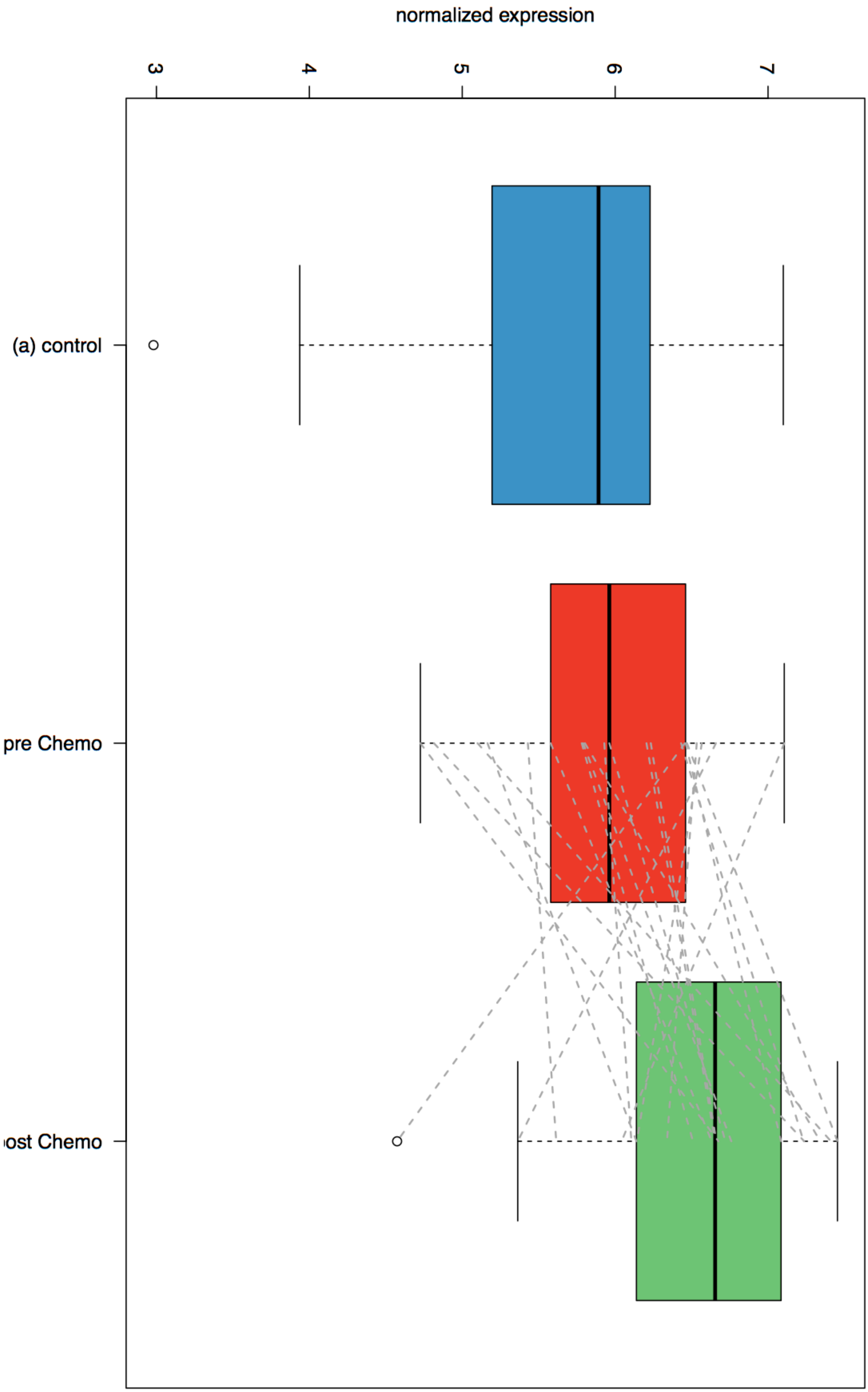
normalized expression
3
4
5
6
7
(a) control
pre Chemo
ost Chemo

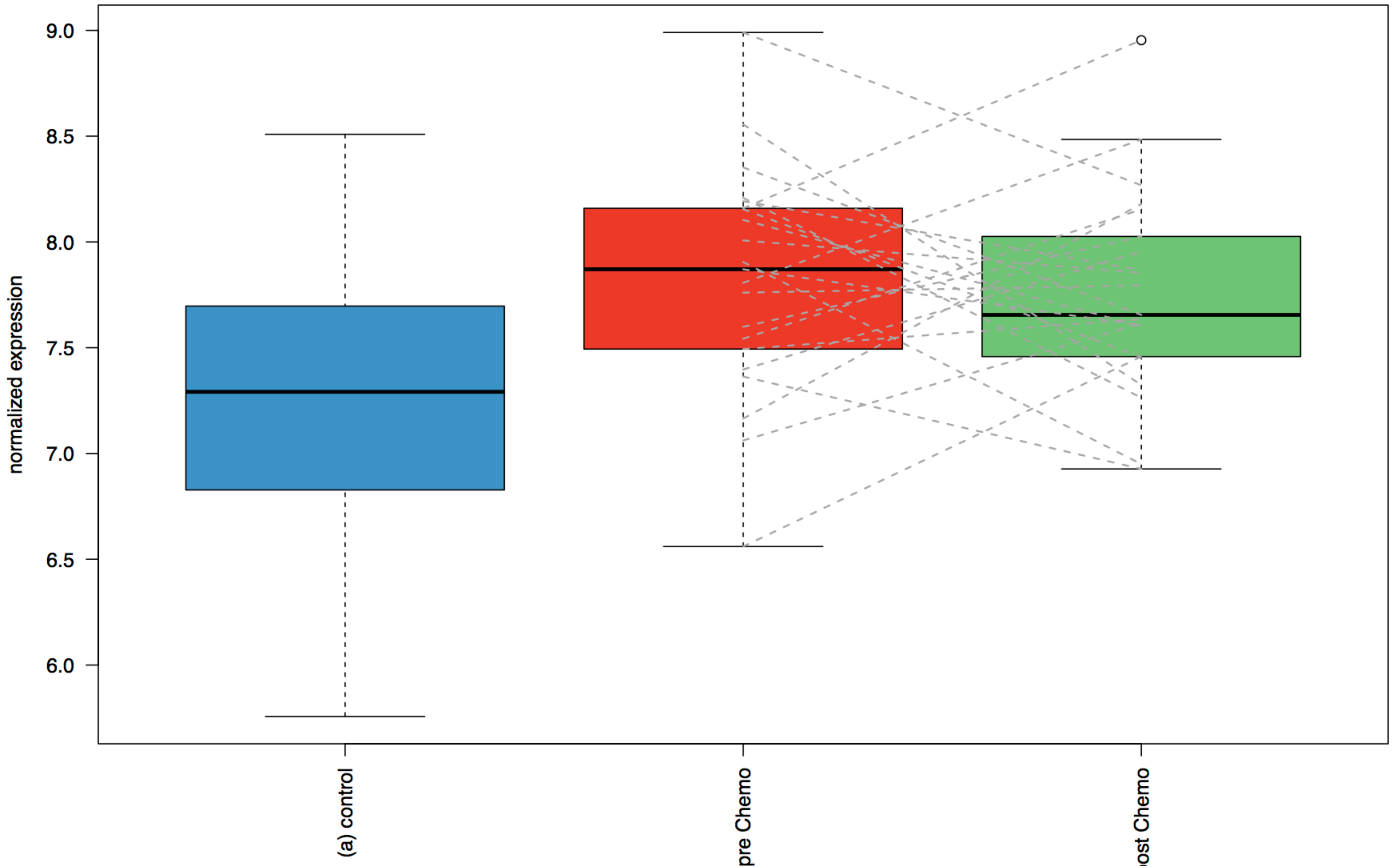
normalized expression
9.0
8.5
8.0
7.5
7.0
6.5
6.0
(a) control
pre Chemo
ost Chemo

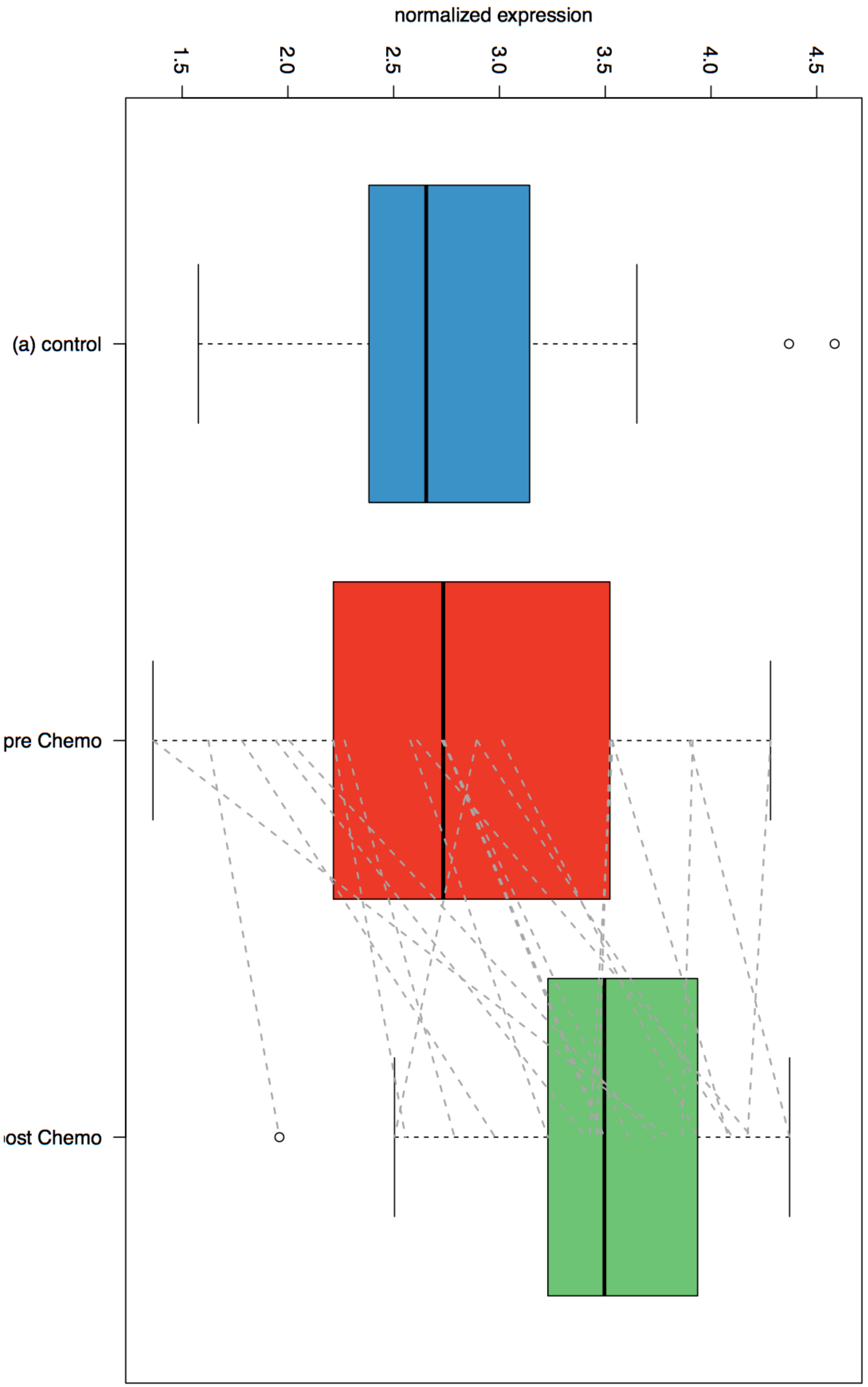
normalized expression
1.5
2.0
2.5
3.0
3.5
4.0
4.5
(a) control
pre Chemo
ost Chemo

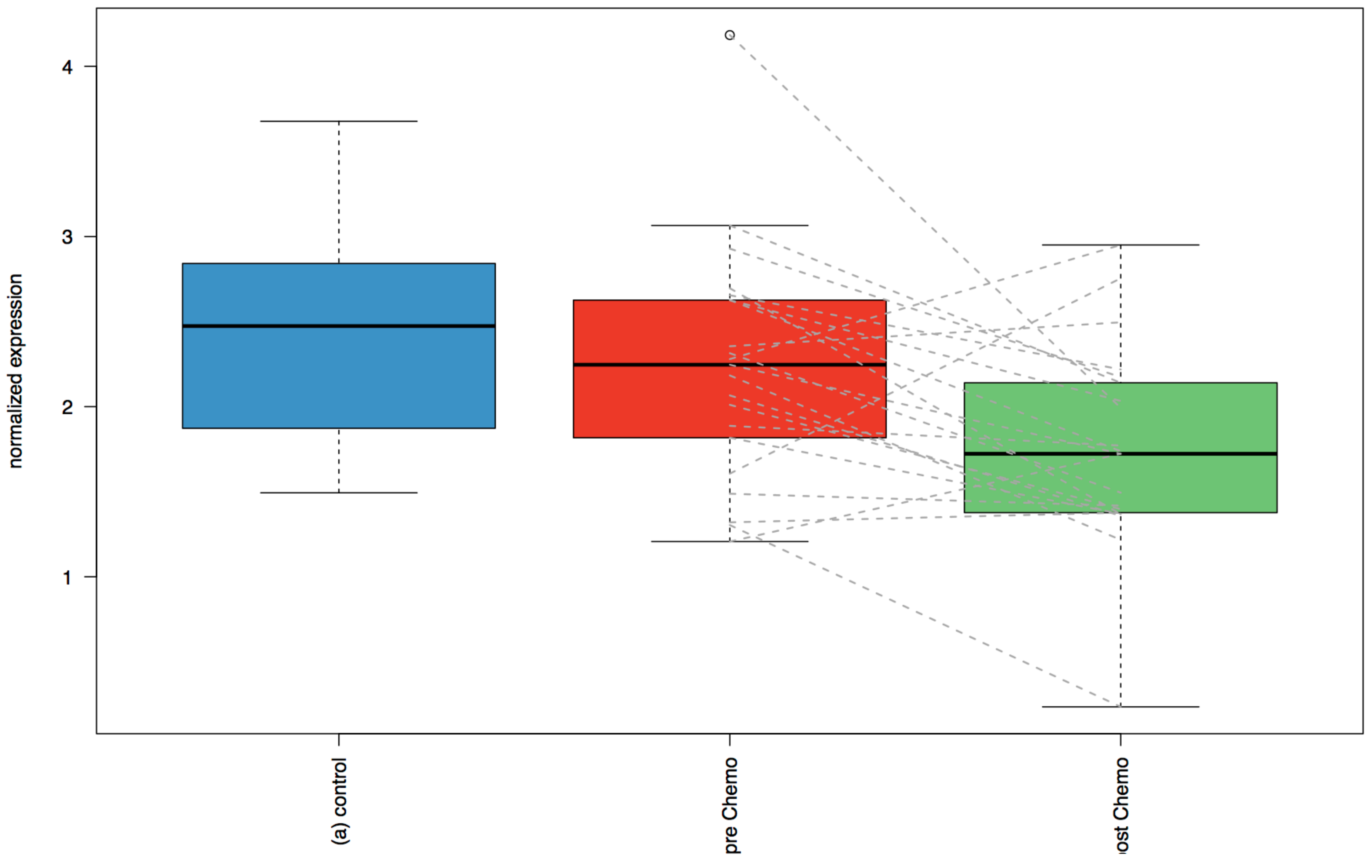
normalized expression
4
3
2
1
(a) control
pre Chemo
ost Chemo

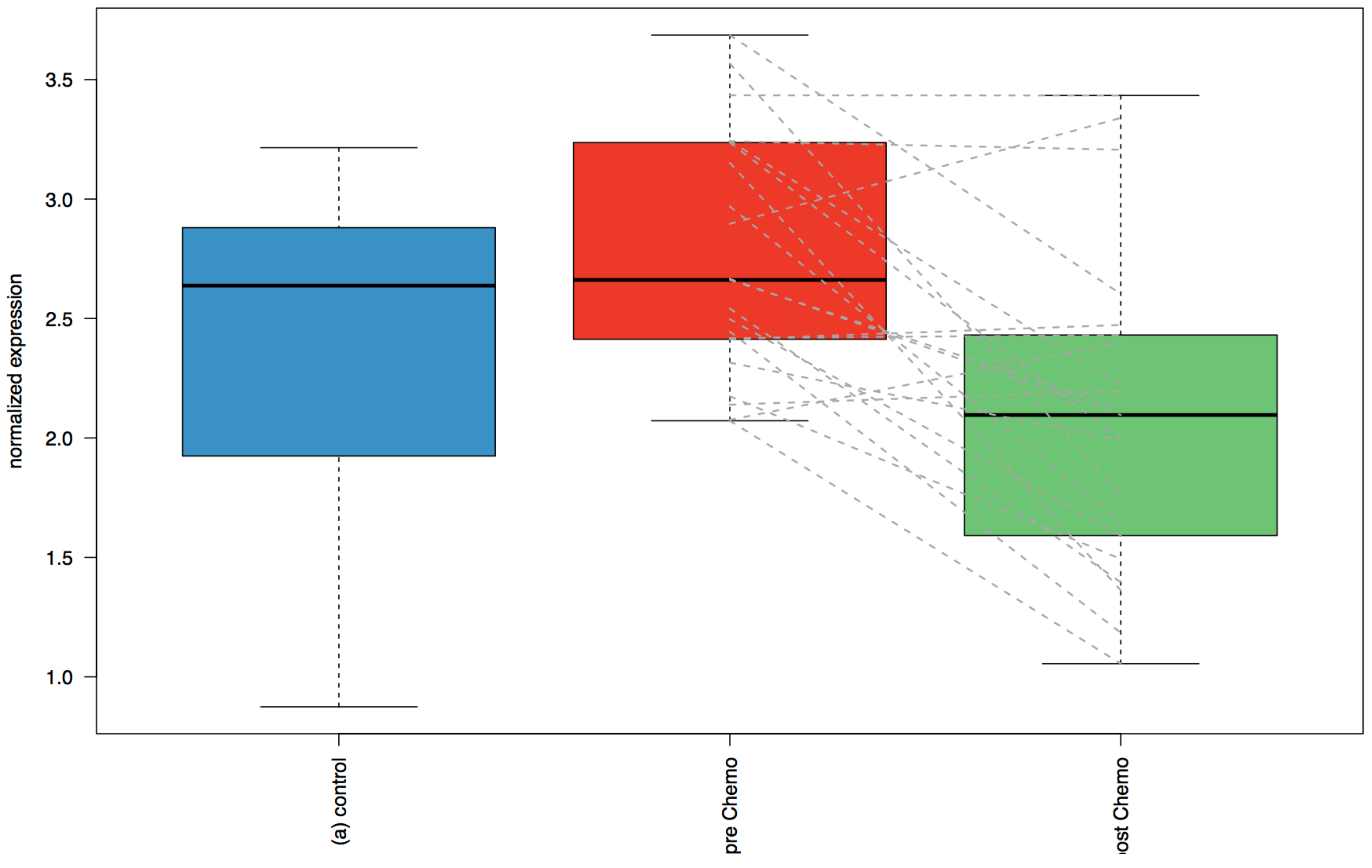
normalized expression
3.5
3.0
2.5
2.0
1.5
1.0
(a) control
pre Chemo
ost Chemo

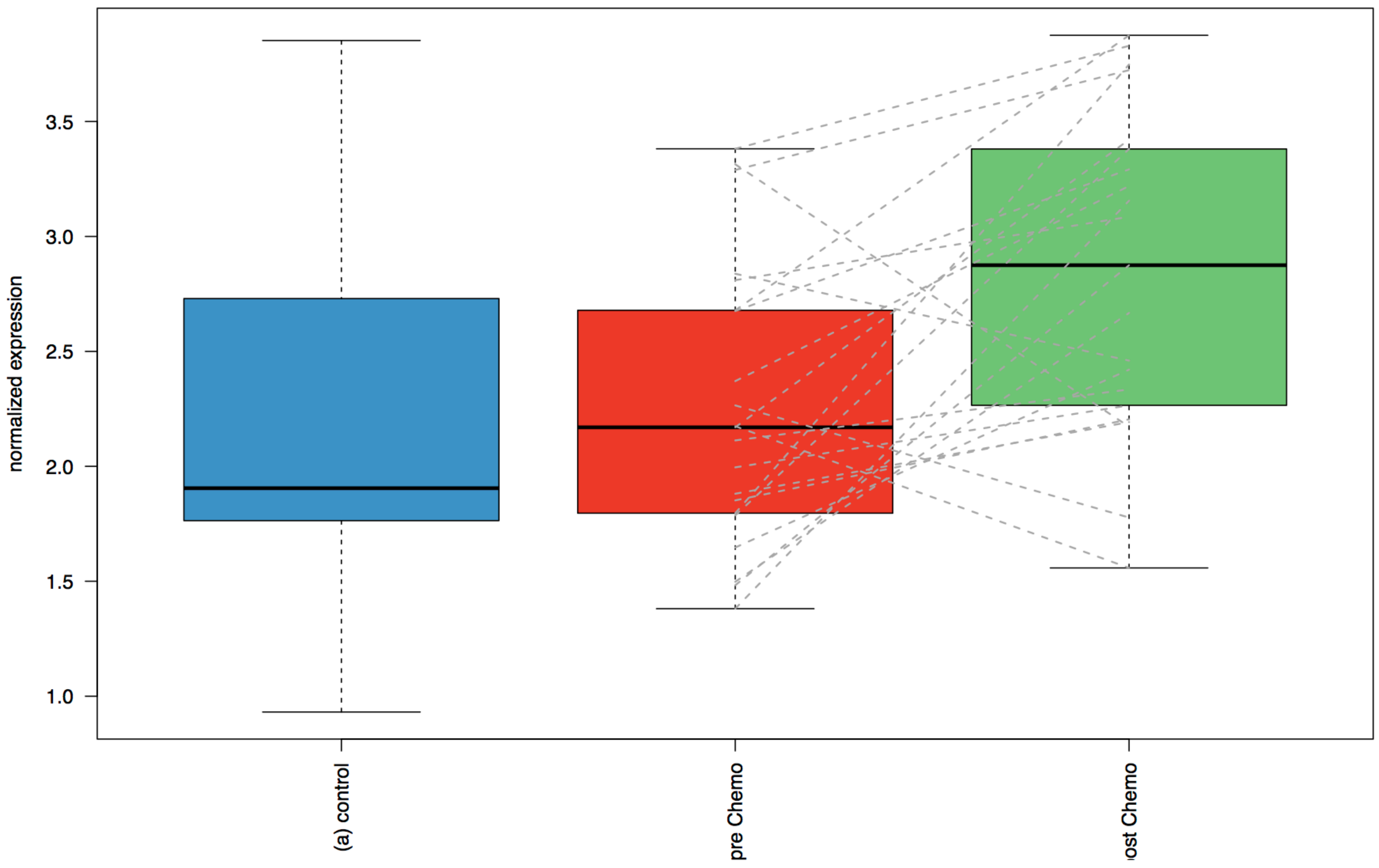
normalized expression
3.5
3.0
2.5
2.0
1.5
1.0
(a) control
pre Chemo
ost Chemo

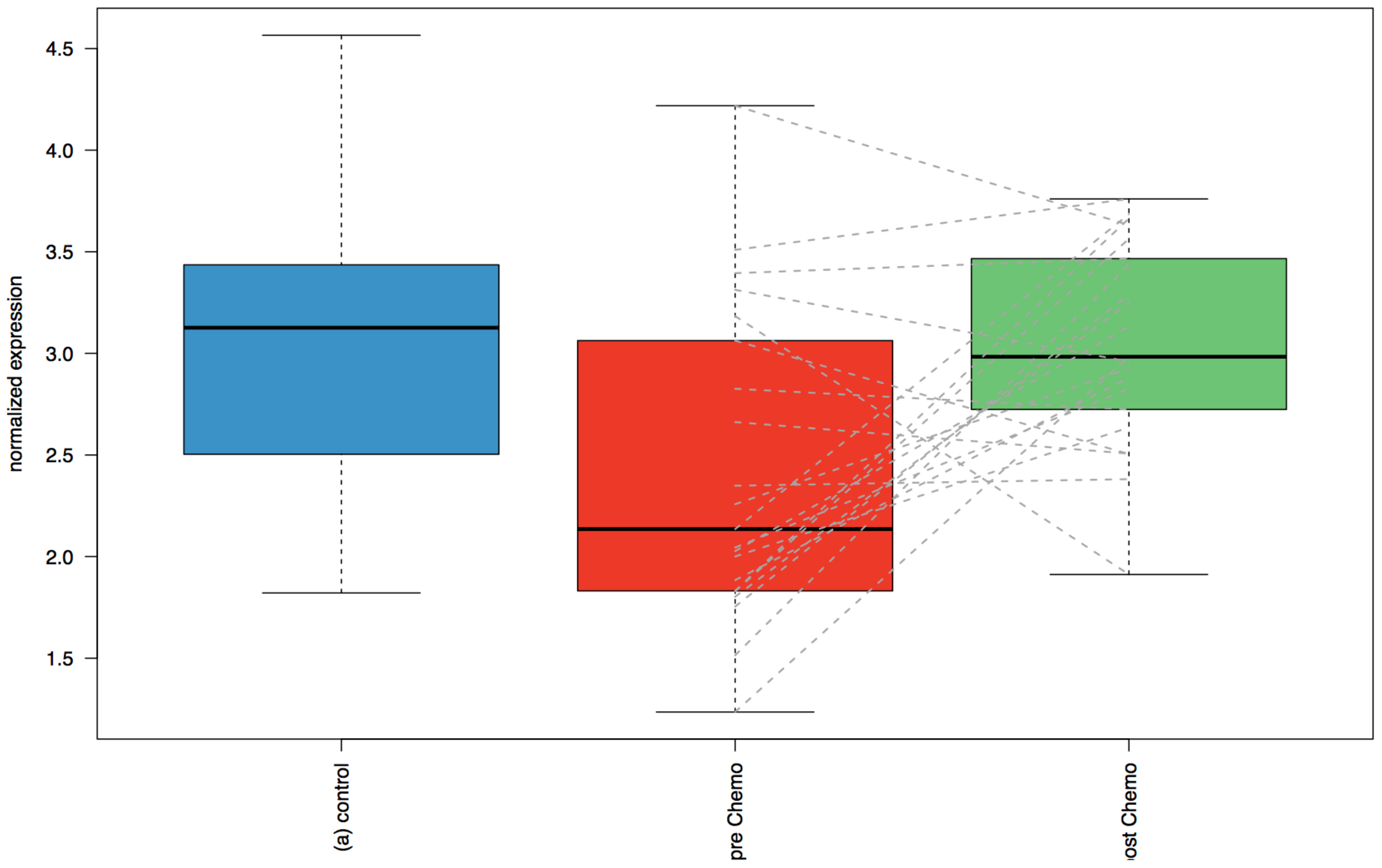
normalized expression
4.5
4.0
3.5
3.0
2.5
2.0
1.5
(a) control
pre Chemo
ost Chemo

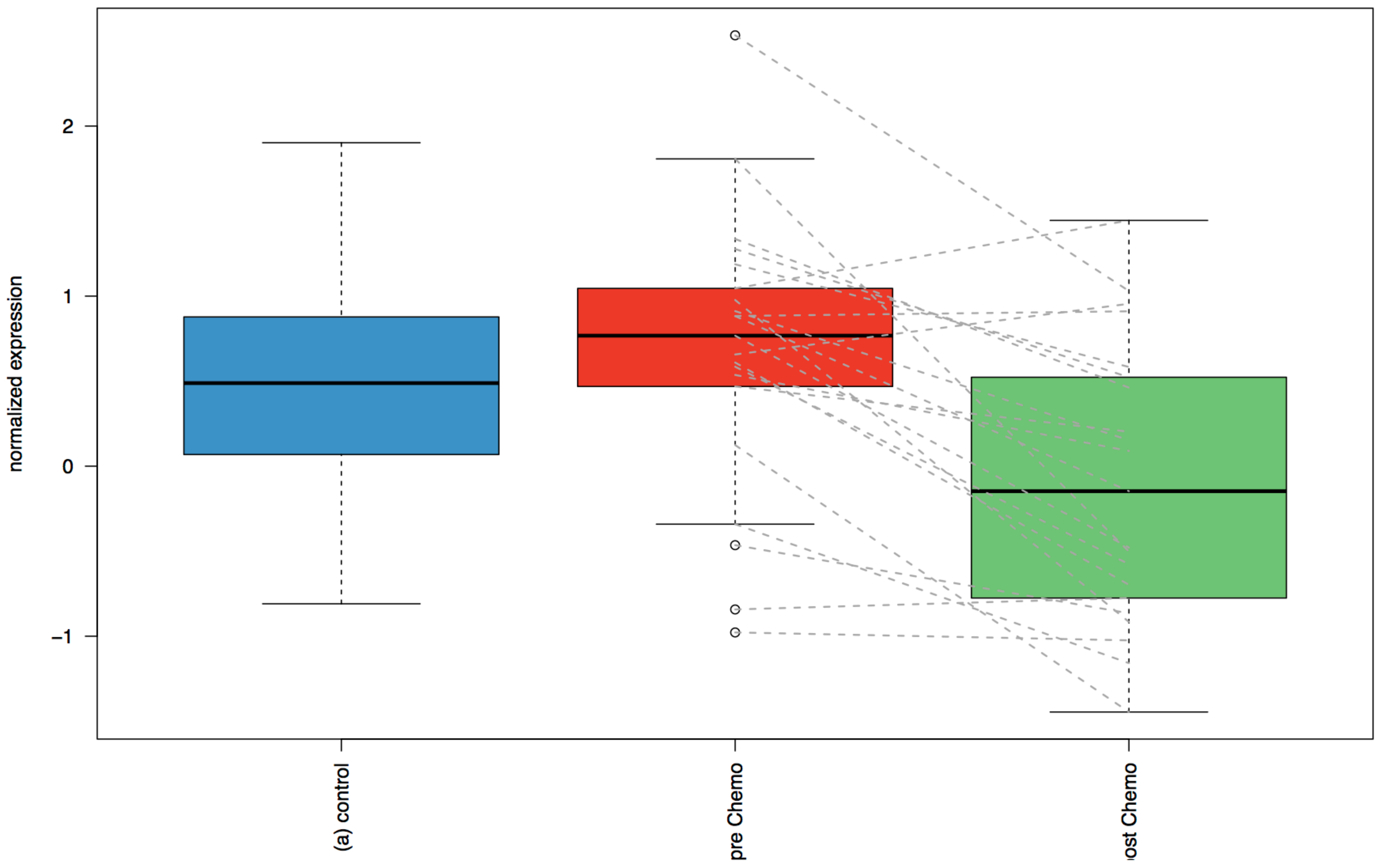
normalized expression
2
1
0
−1
(a) control
pre Chemo
ost Chemo

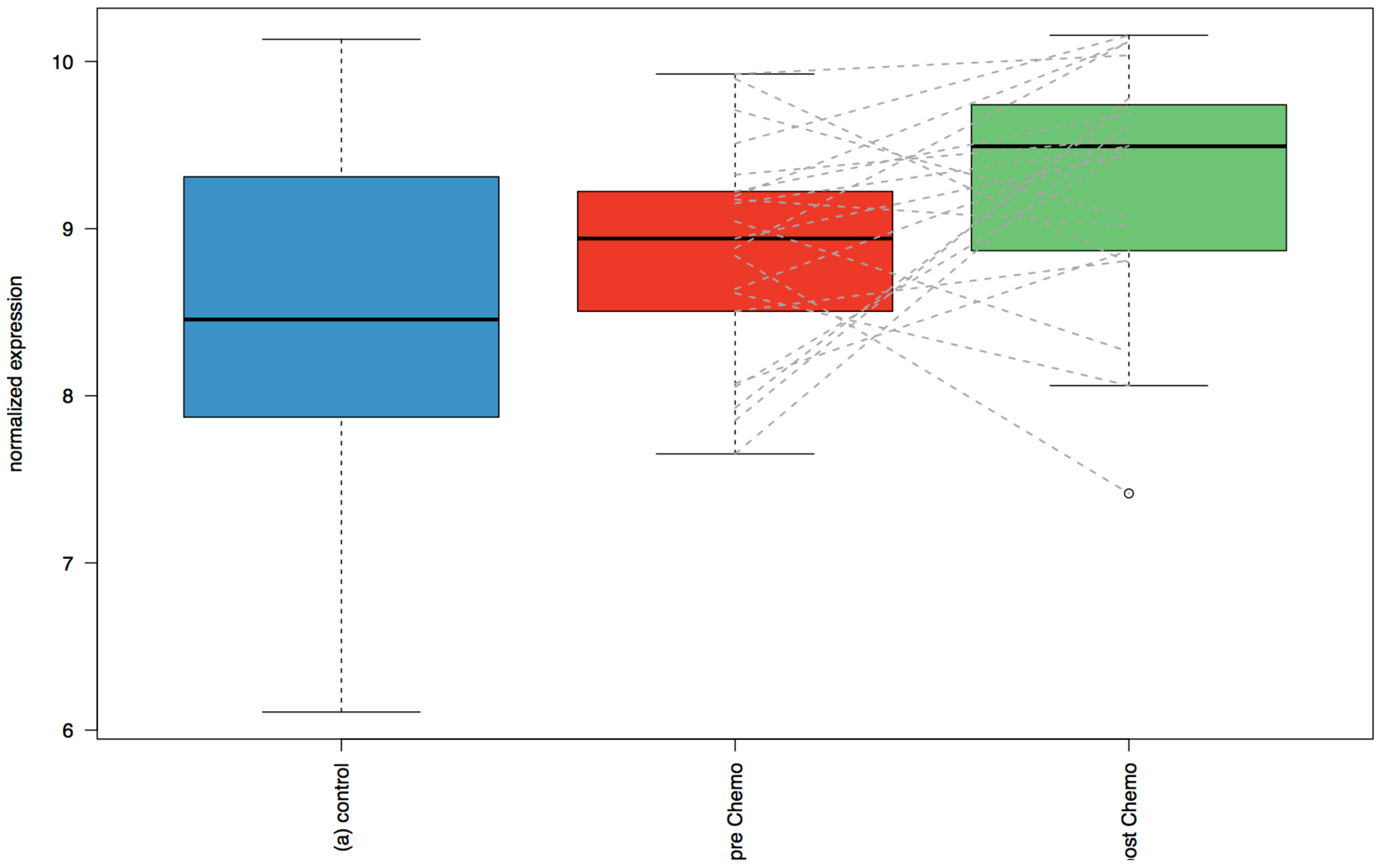
normalized expression
10
9
8
7
6
(a) control
pre Chemo
ost Chemo

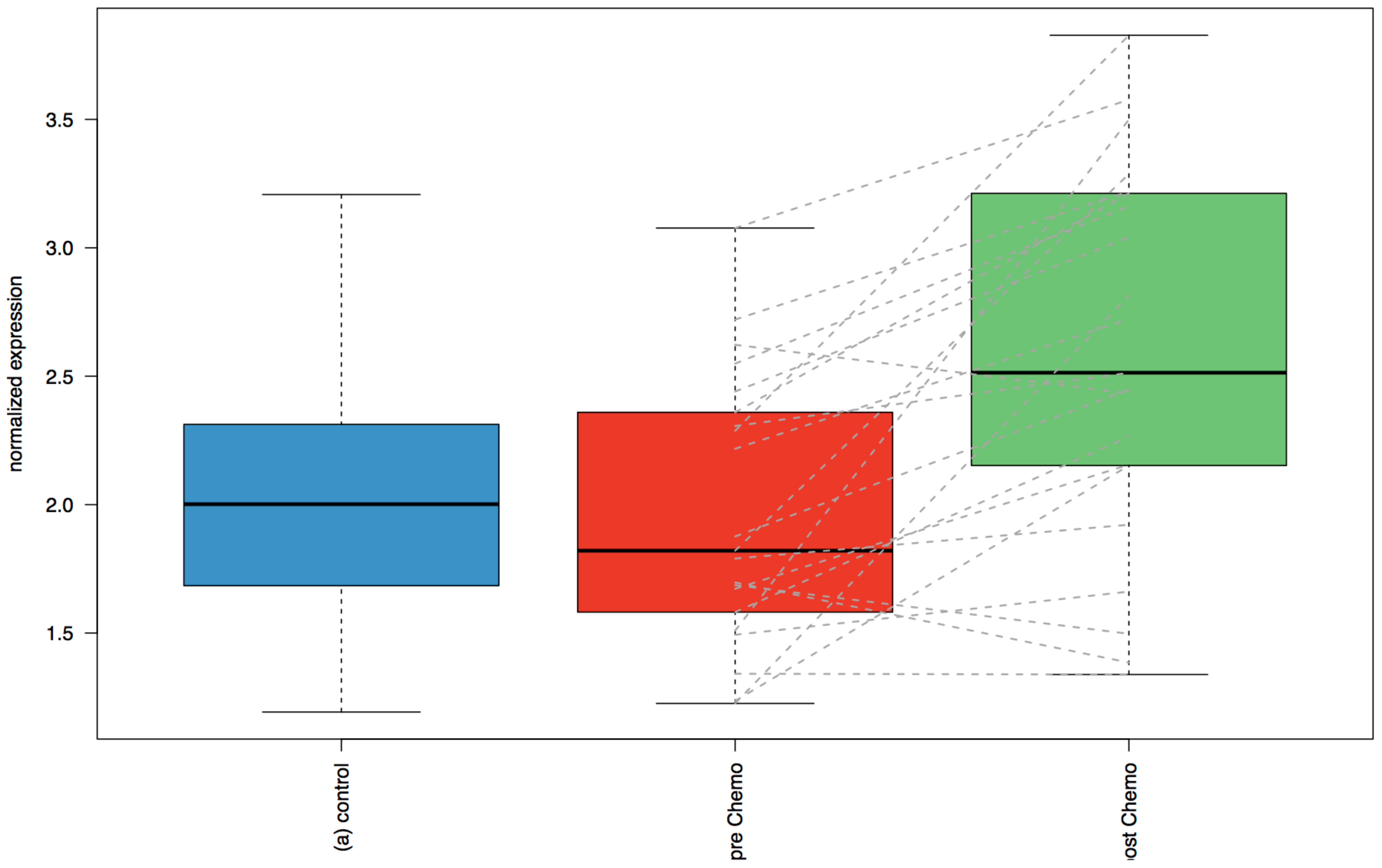
normalized expression
3.5
3.0
2.5
2.0
1.5
(a) control
pre Chemo
ost Chemo

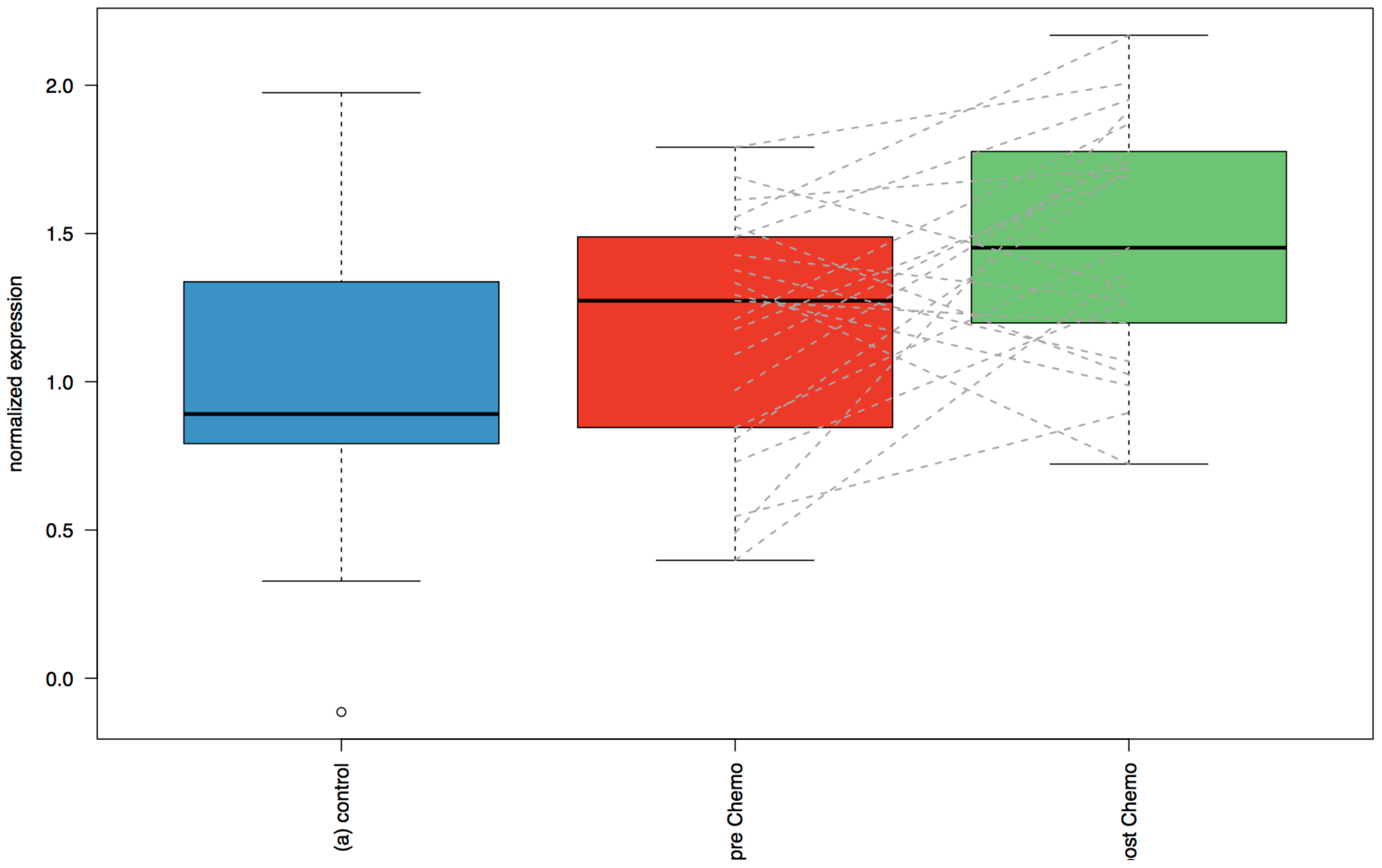
normalized expression
2.0
1.5
1.0
0.5
0.0
(a) control
pre Chemo
ost Chemo

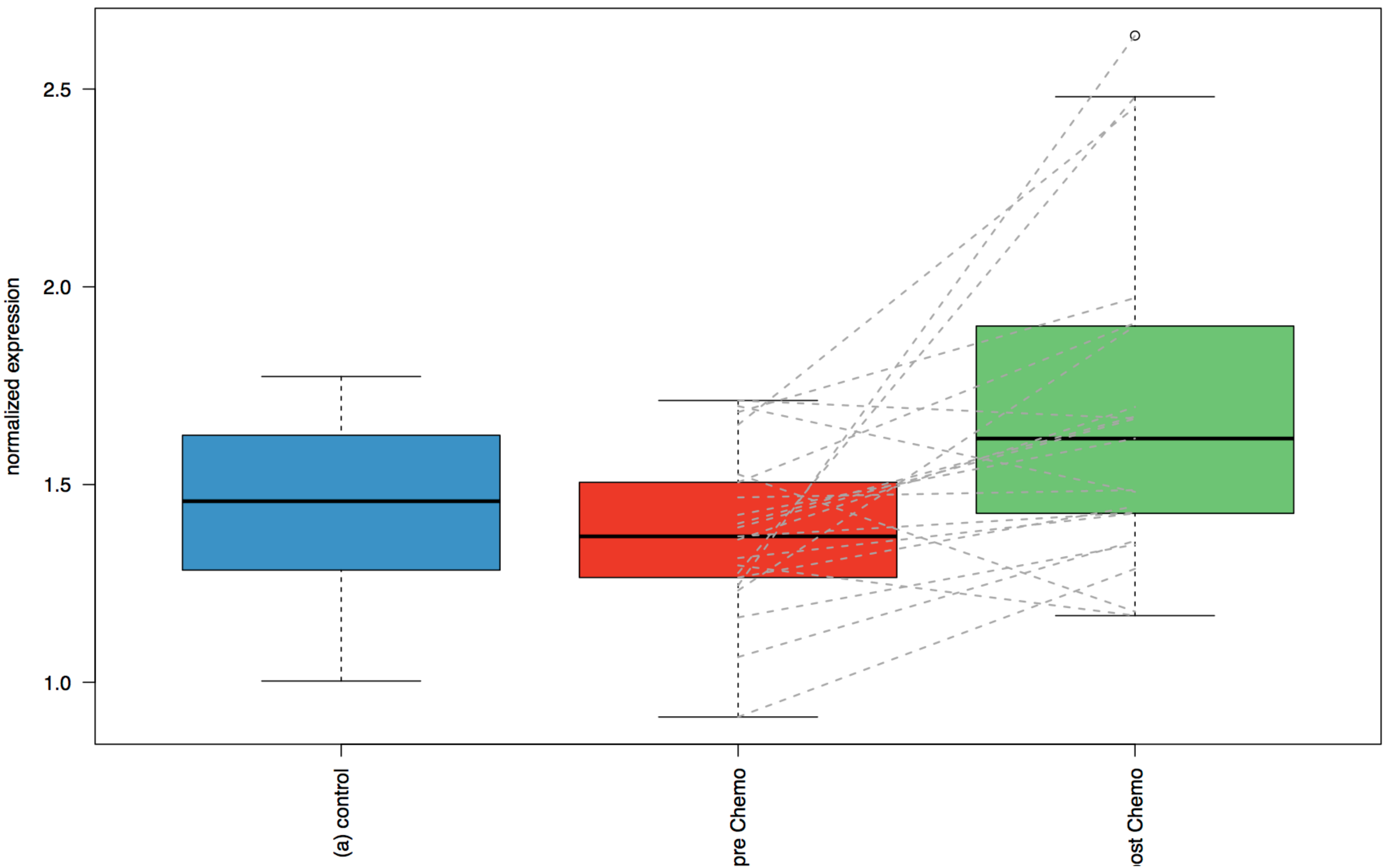
normalized expression
2.5
2.0
1.5
1.0
(a) control
pre Chemo
ost Chemo

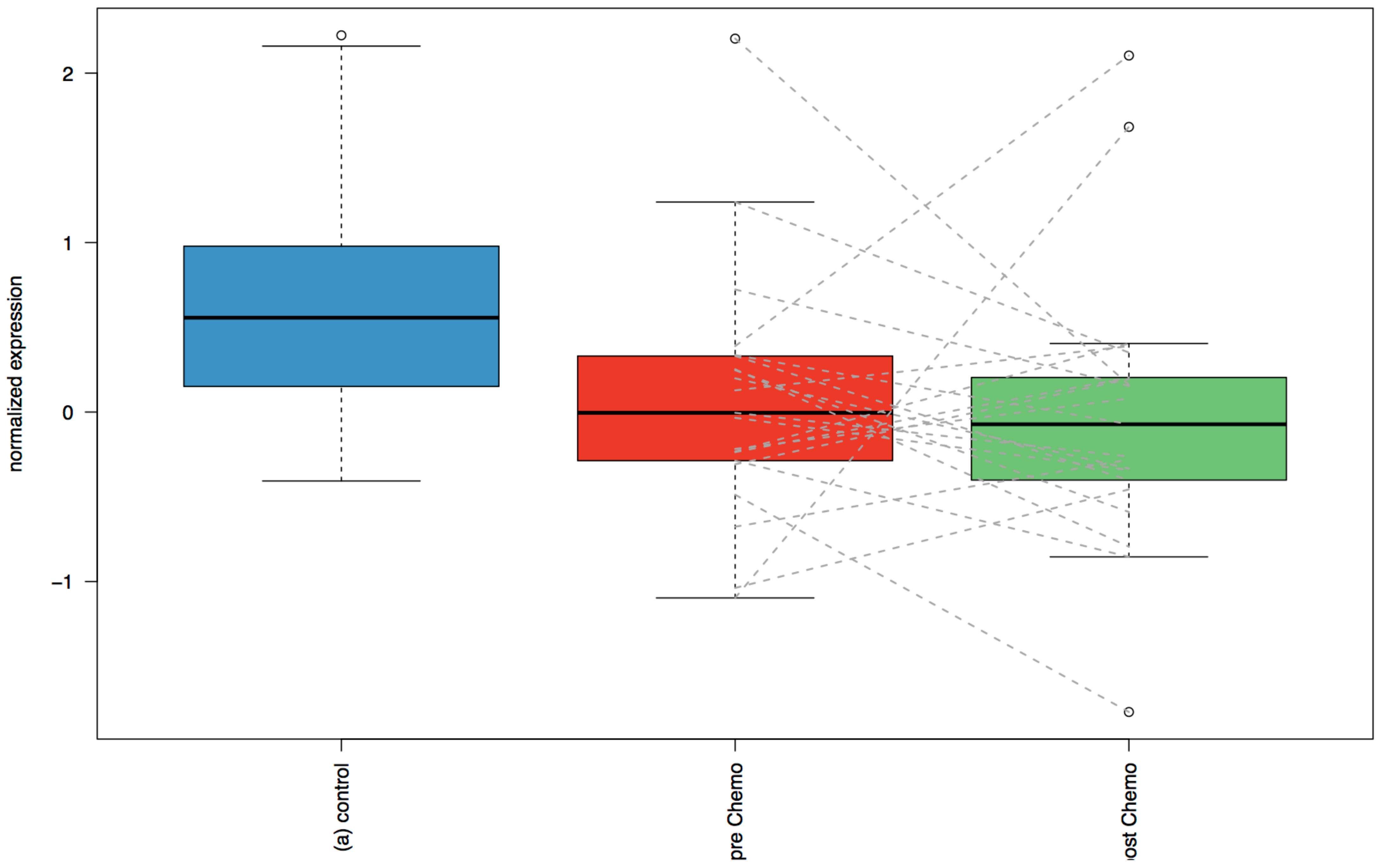
normalized expression
2
1
0
−1
(a) control
pre Chemo
ost Chemo

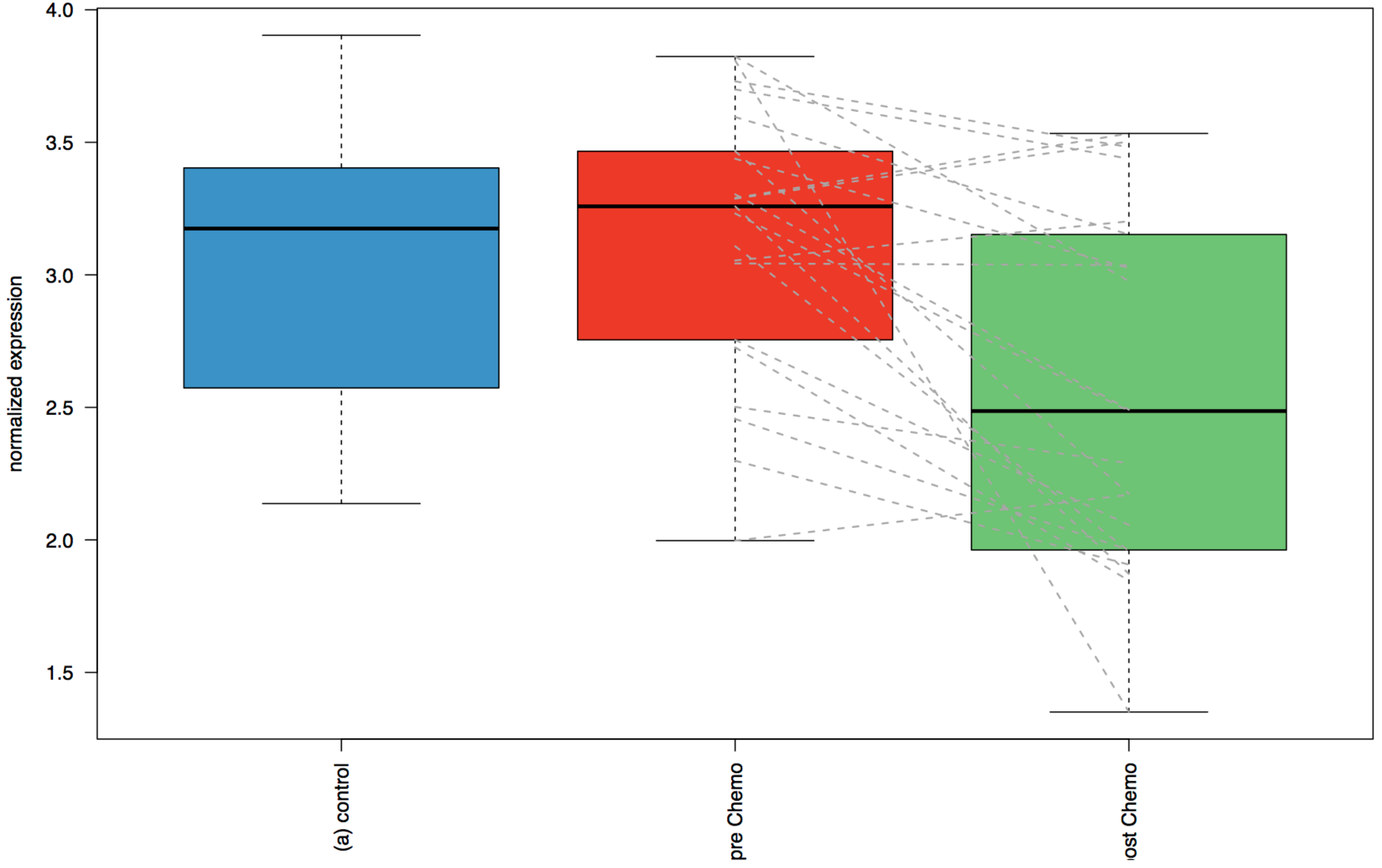
normalized expression
4.0
3.5
3.0
2.5
2.0
1.5
(a) control
pre Chemo
ost Chemo

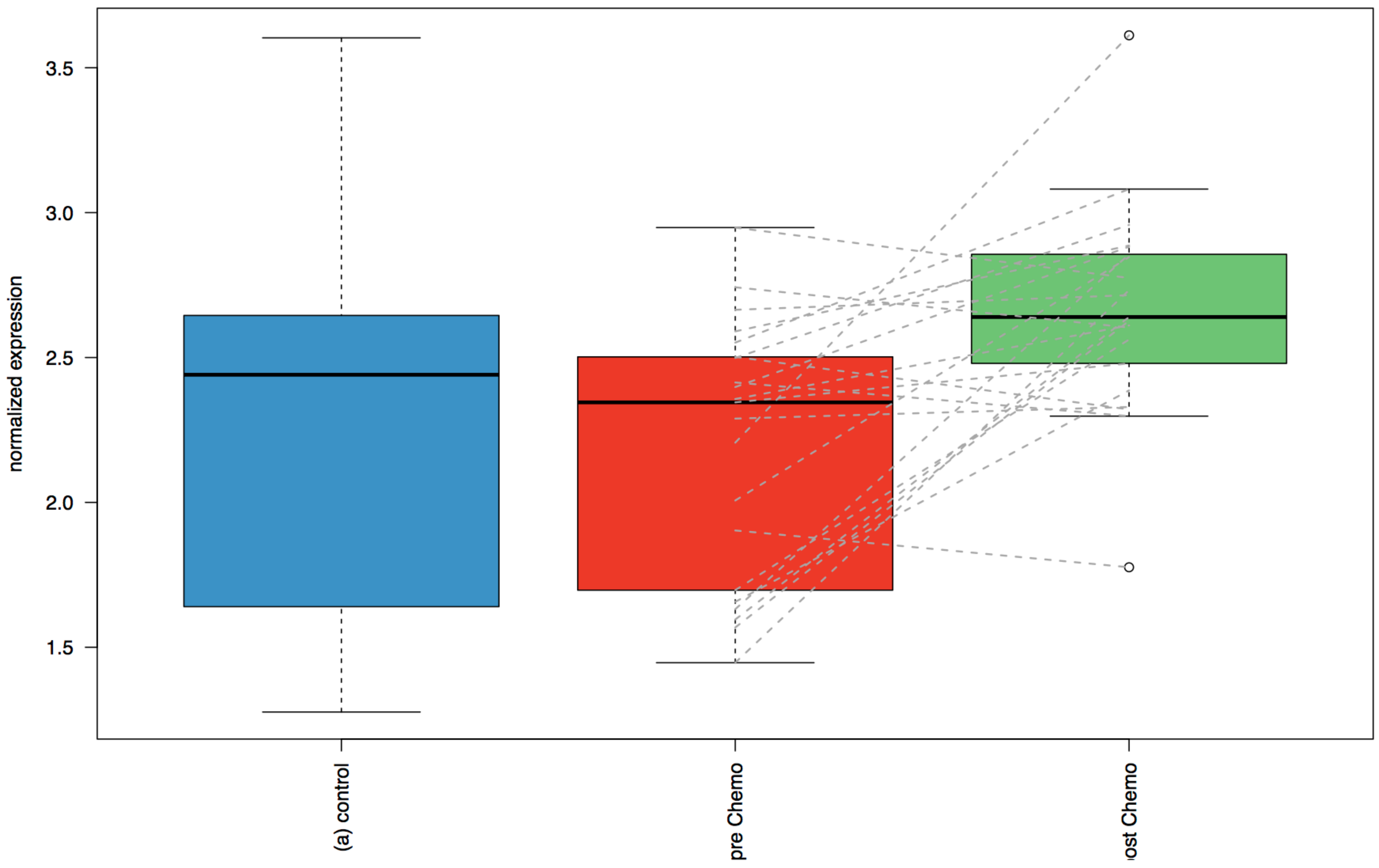
normalized expression
3.5
3.0
2.5
2.0
1.5
(a) control
pre Chemo
ost Chemo

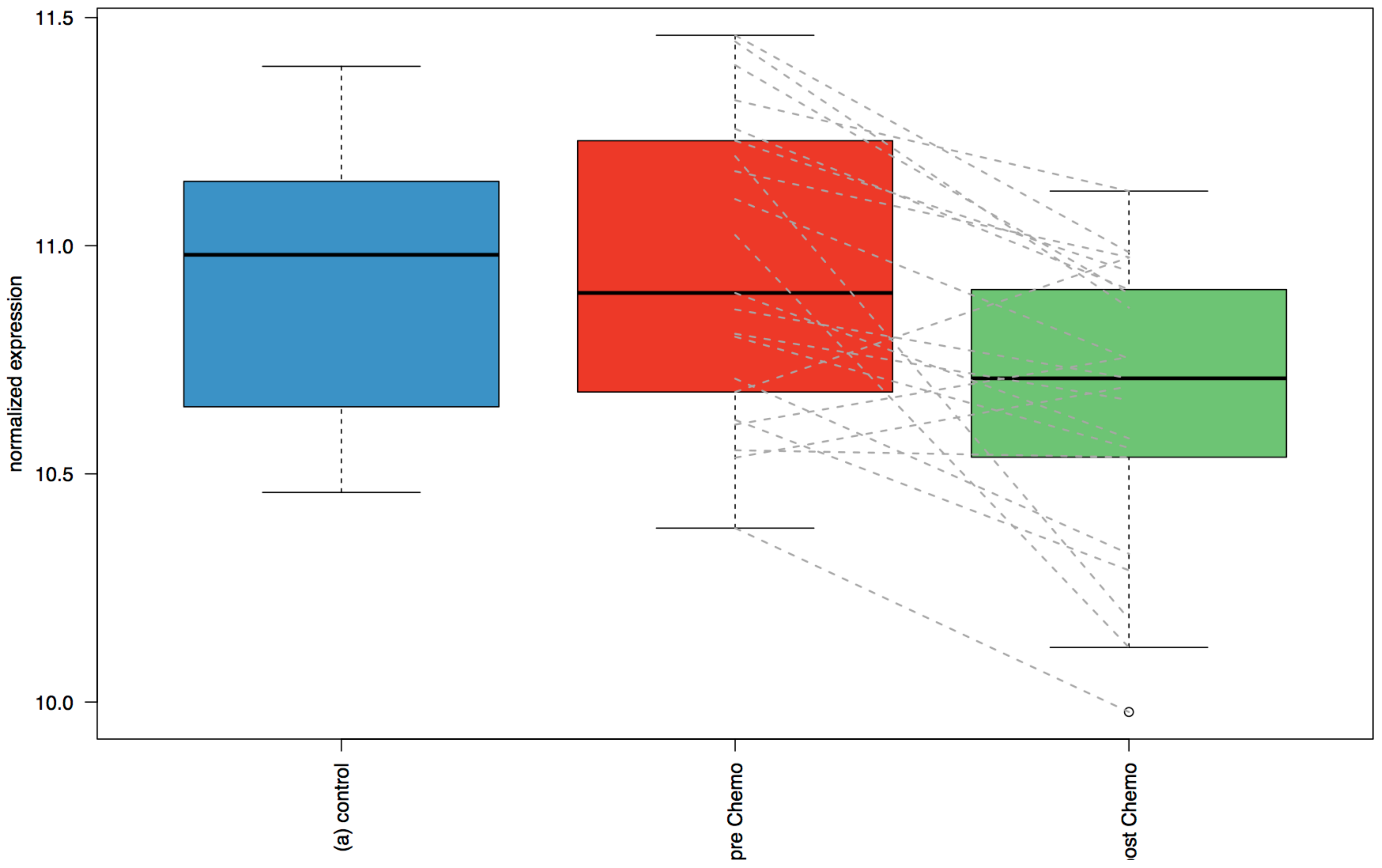

normalized expression
11.5
11.0
10.5
10.0
(a) control
pre Chemo
ost Chemo

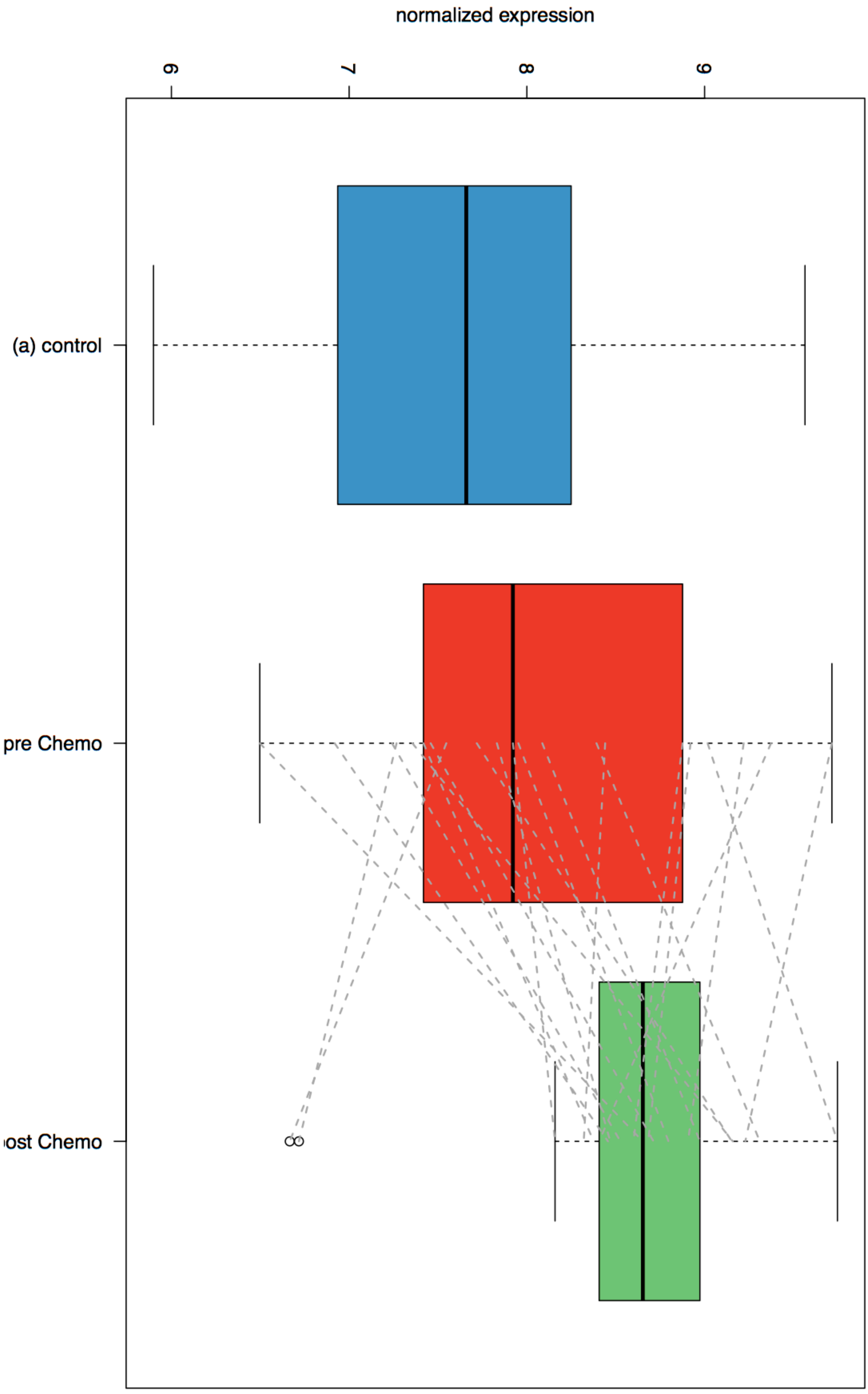
normalized expression
6
7
8
9
(a) control
pre Chemo
ost Chemo

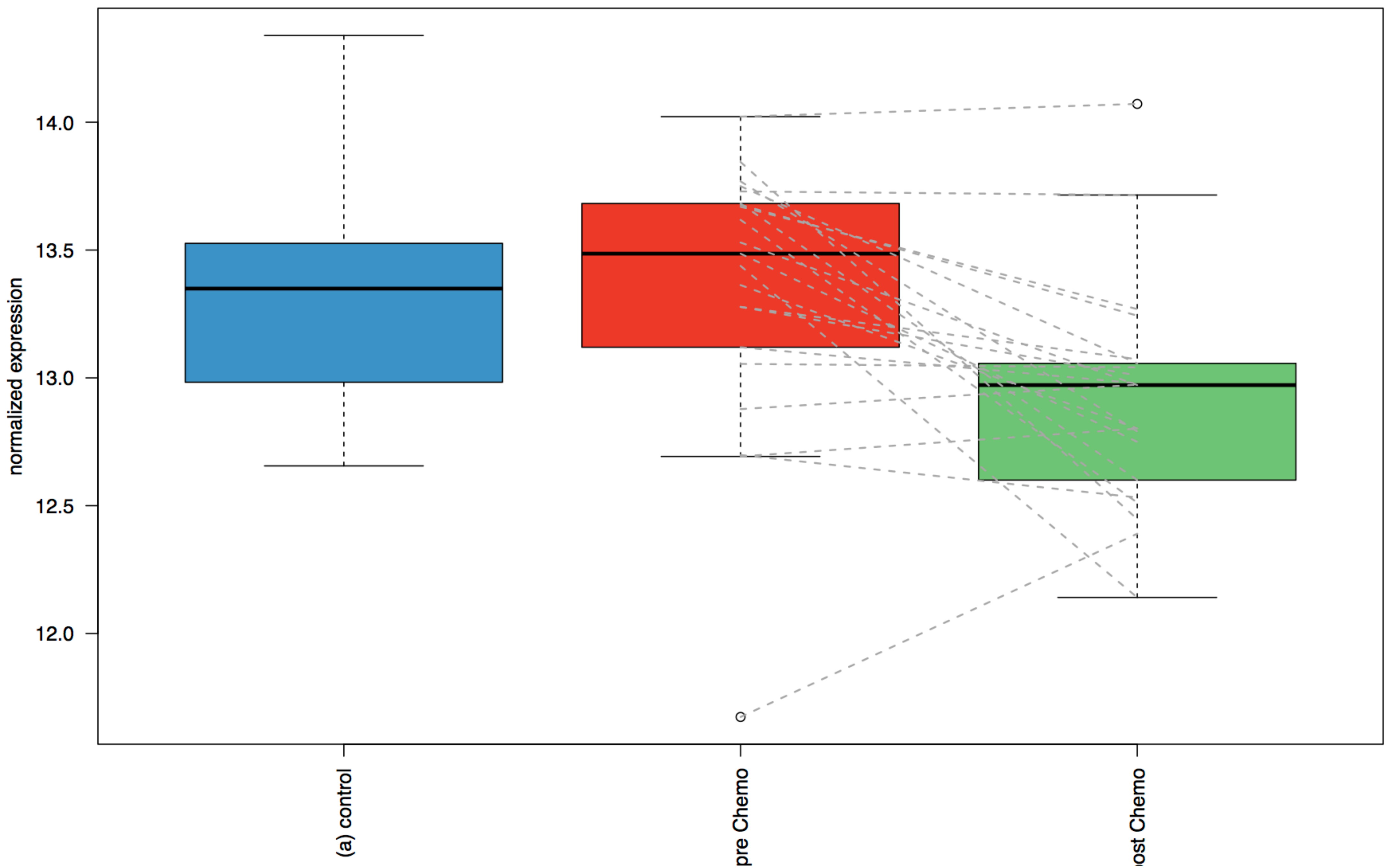
normalized expression
14.0
13.5
13.0
12.5
12.0
(a) control
pre Chemo
ost Chemo

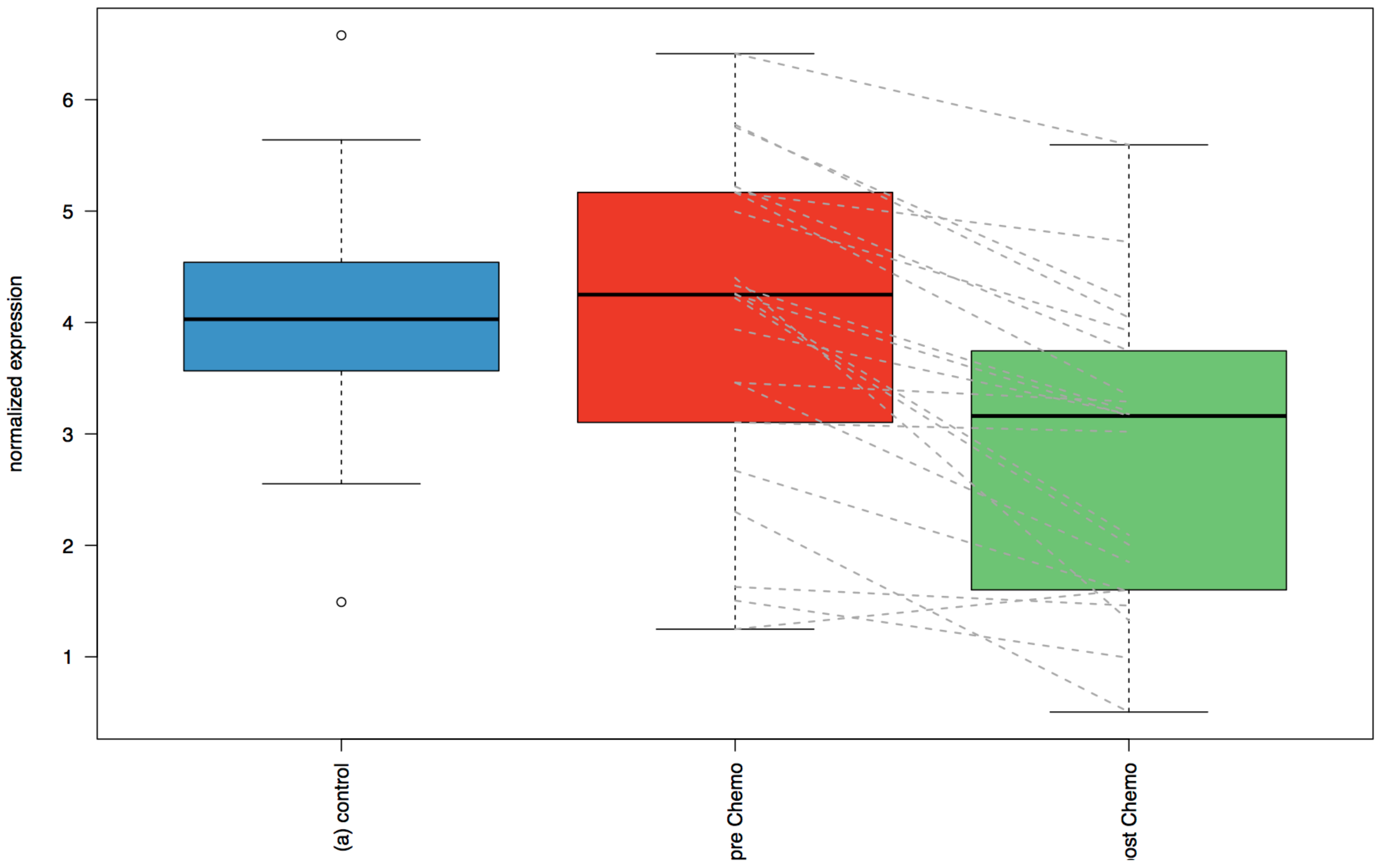
normalized expression
1
2
3
4
5
6
(a) control
pre Chemo
ost Chemo

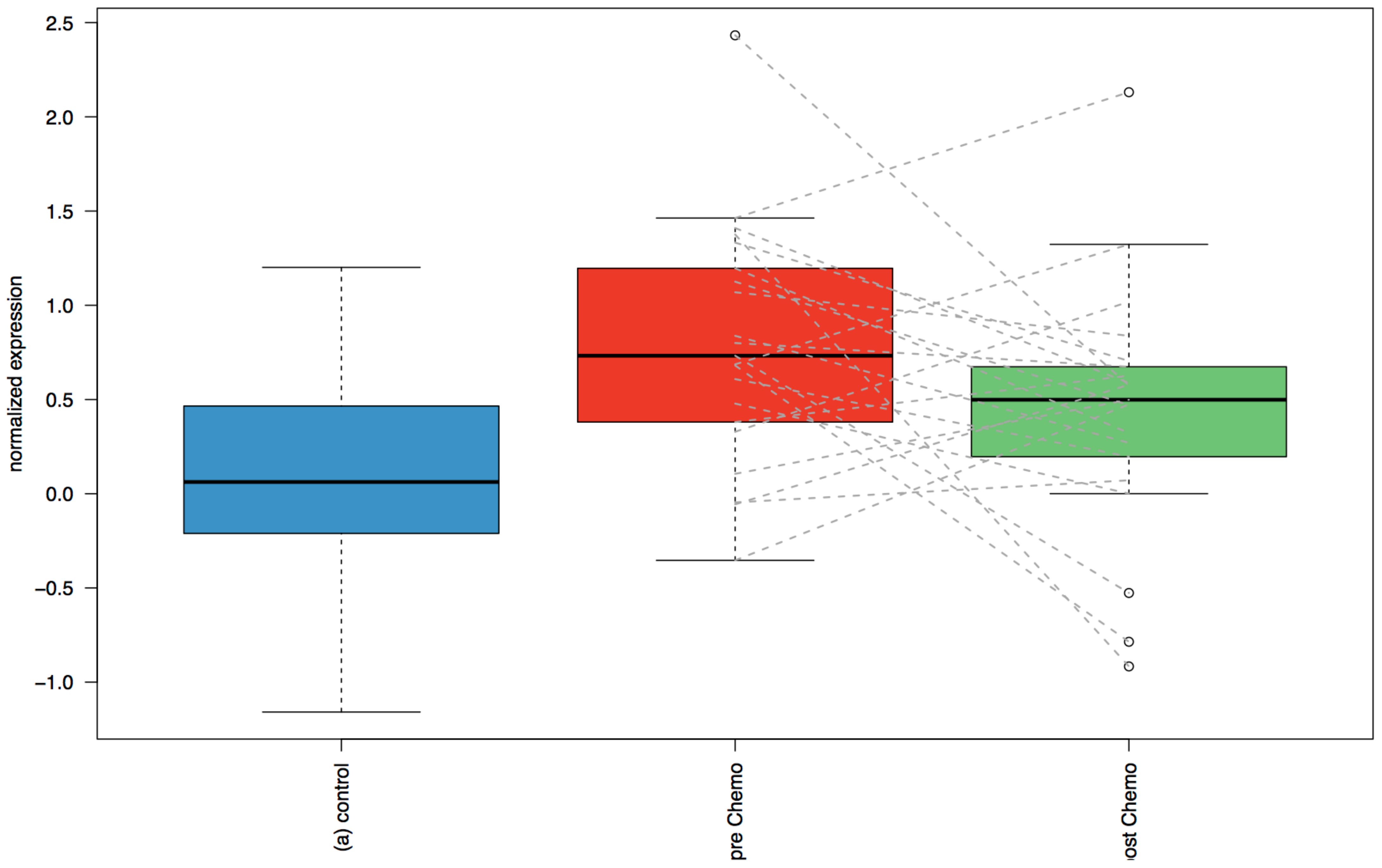
normalized expression
2.5
2.0
1.5
1.0
0.5
0.0
−0.5
−1.0
(a) control
pre Chemo
ost Chemo

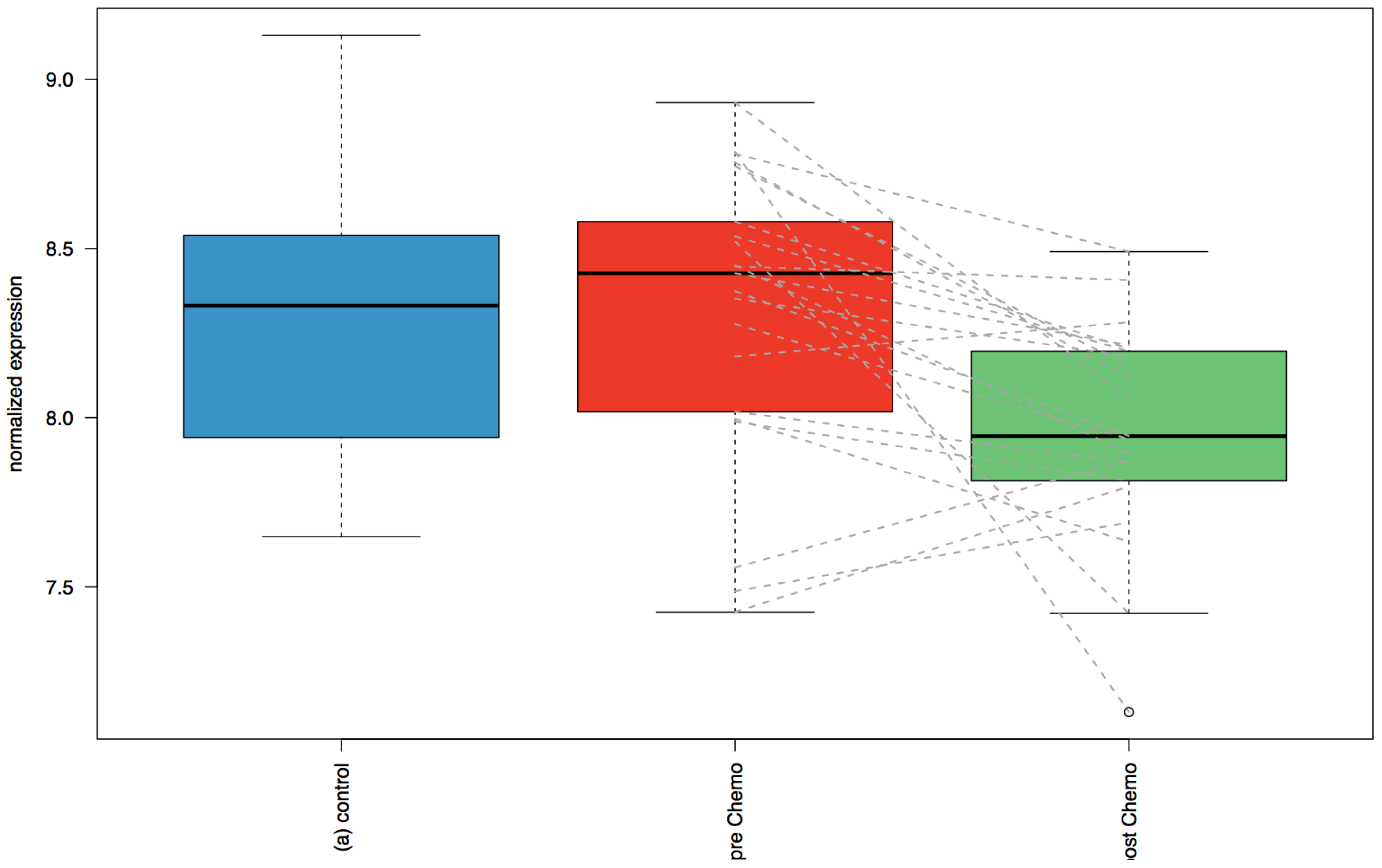
normalized expression
9.0
8.5
8.0
7.5
(a) control
pre Chemo
ost Chemo

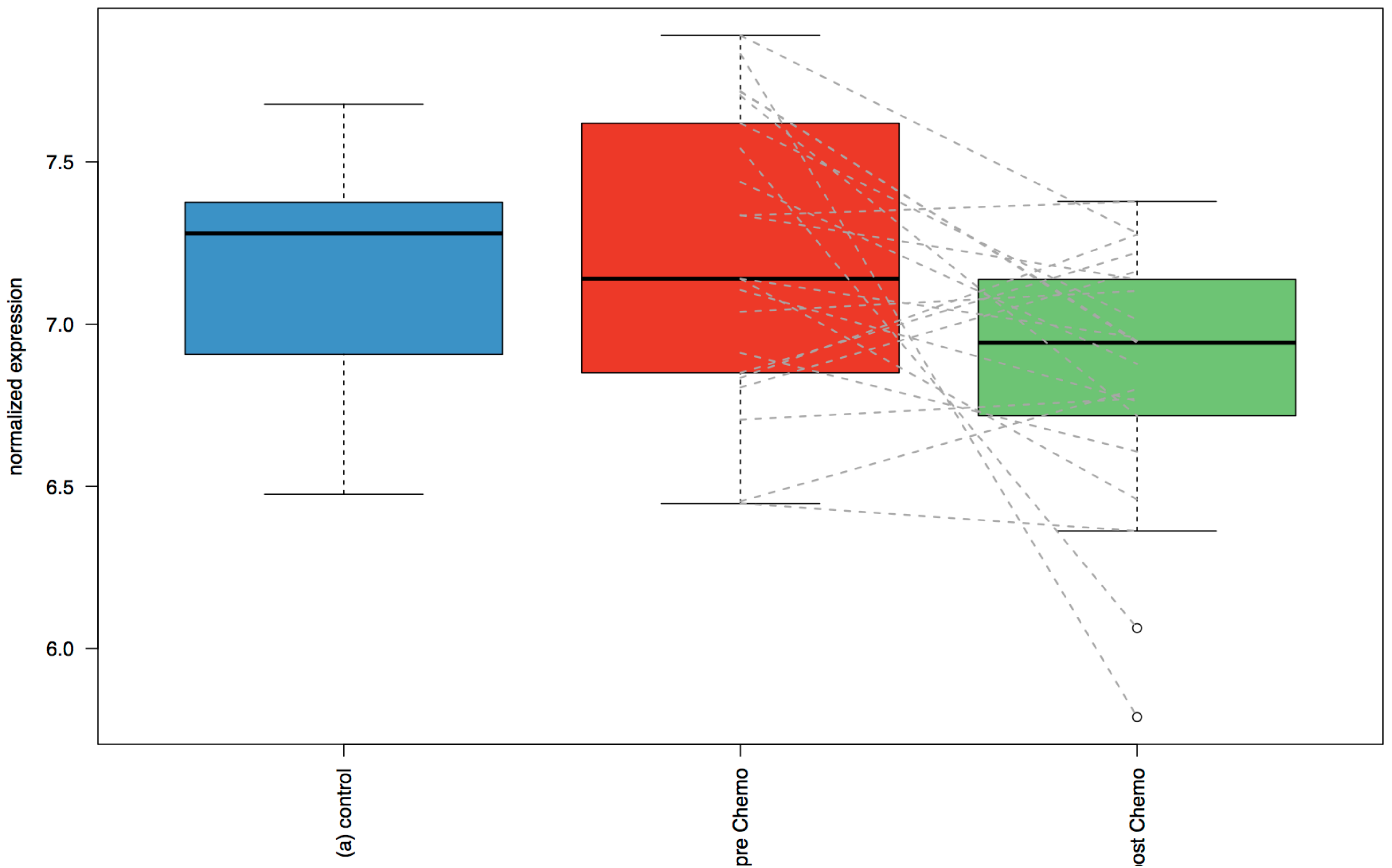
normalized expression
7.5
7.0
6.5
6.0
(a) control
pre Chemo
ost Chemo

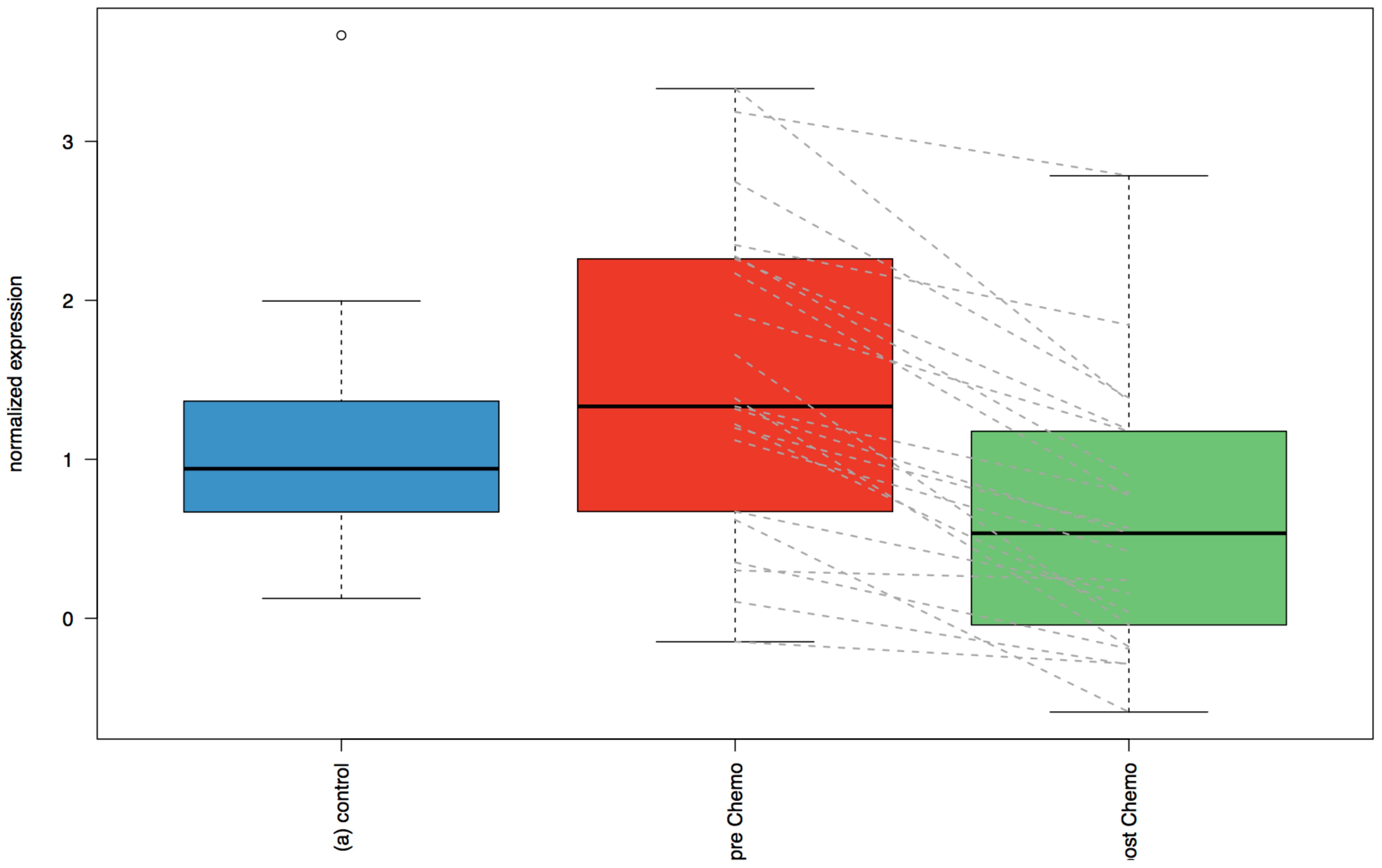
normalized expression
0
1
2
3
(a) control
pre Chemo
ost Chemo

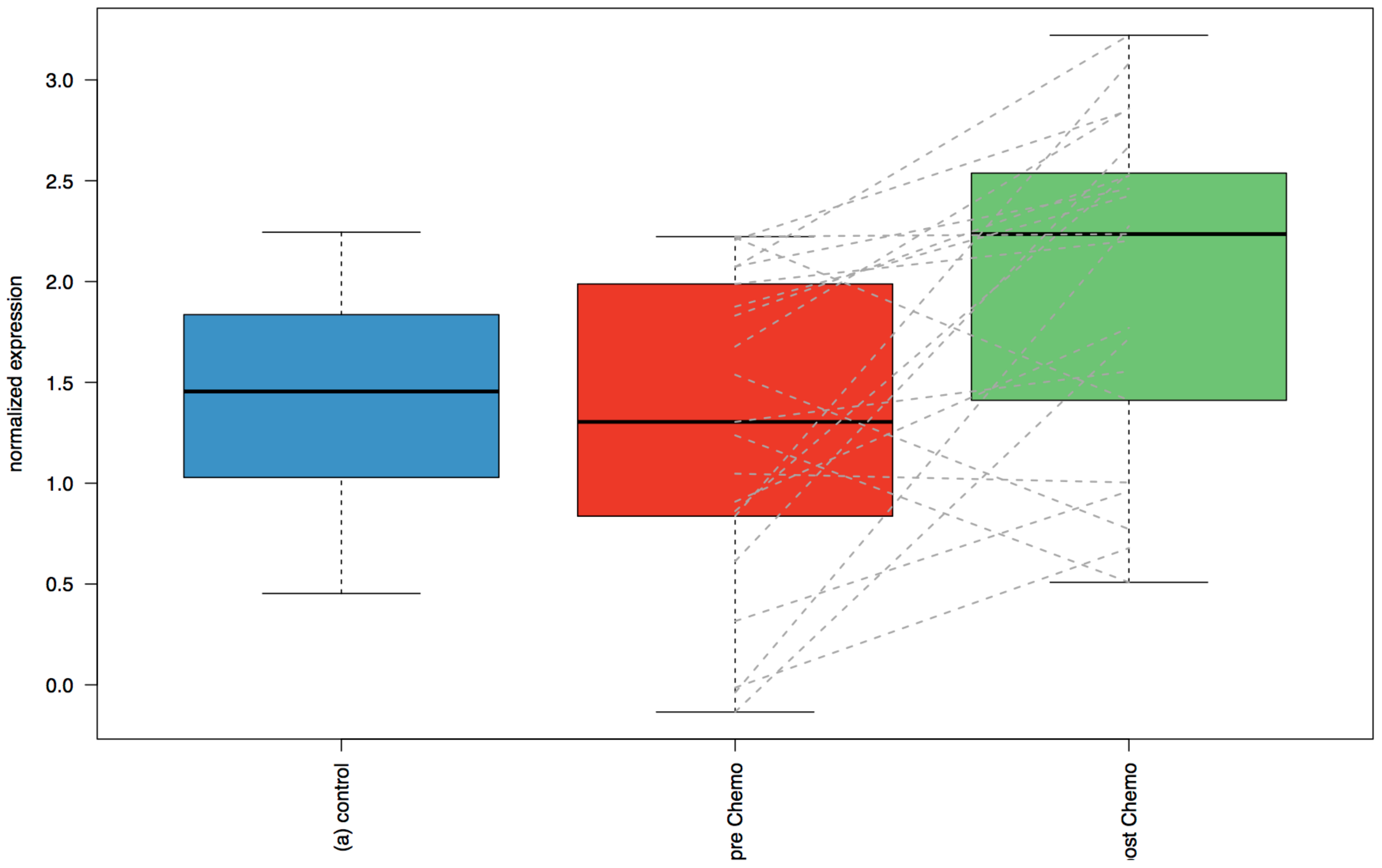
normalized expression
0.0
0.5
1.0
1.5
2.0
2.5
3.0
(a) control
pre Chemo
ost Chemo

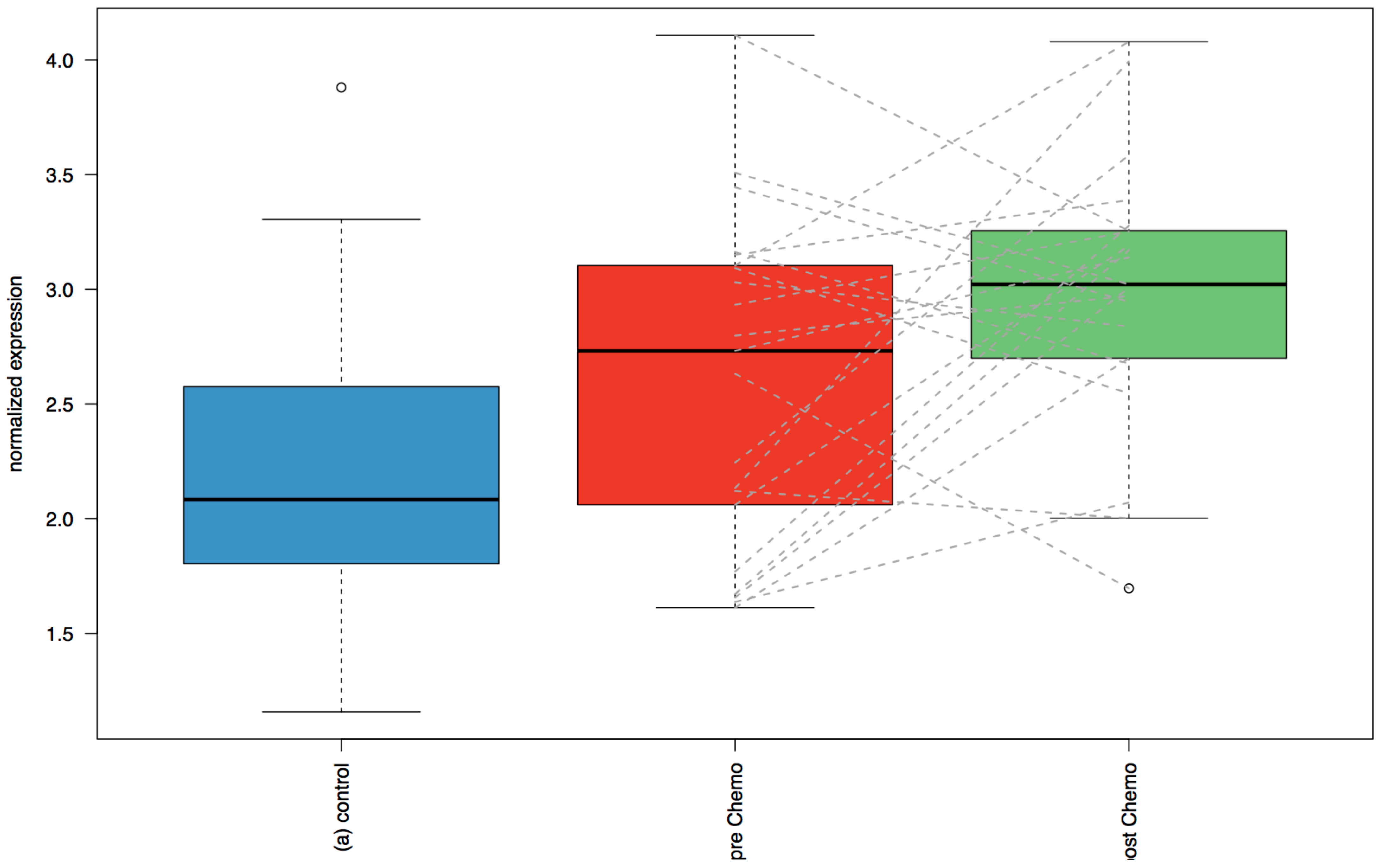
normalized expression
4.0
3.5
3.0
2.5
2.0
1.5
(a) control
pre Chemo
ost Chemo

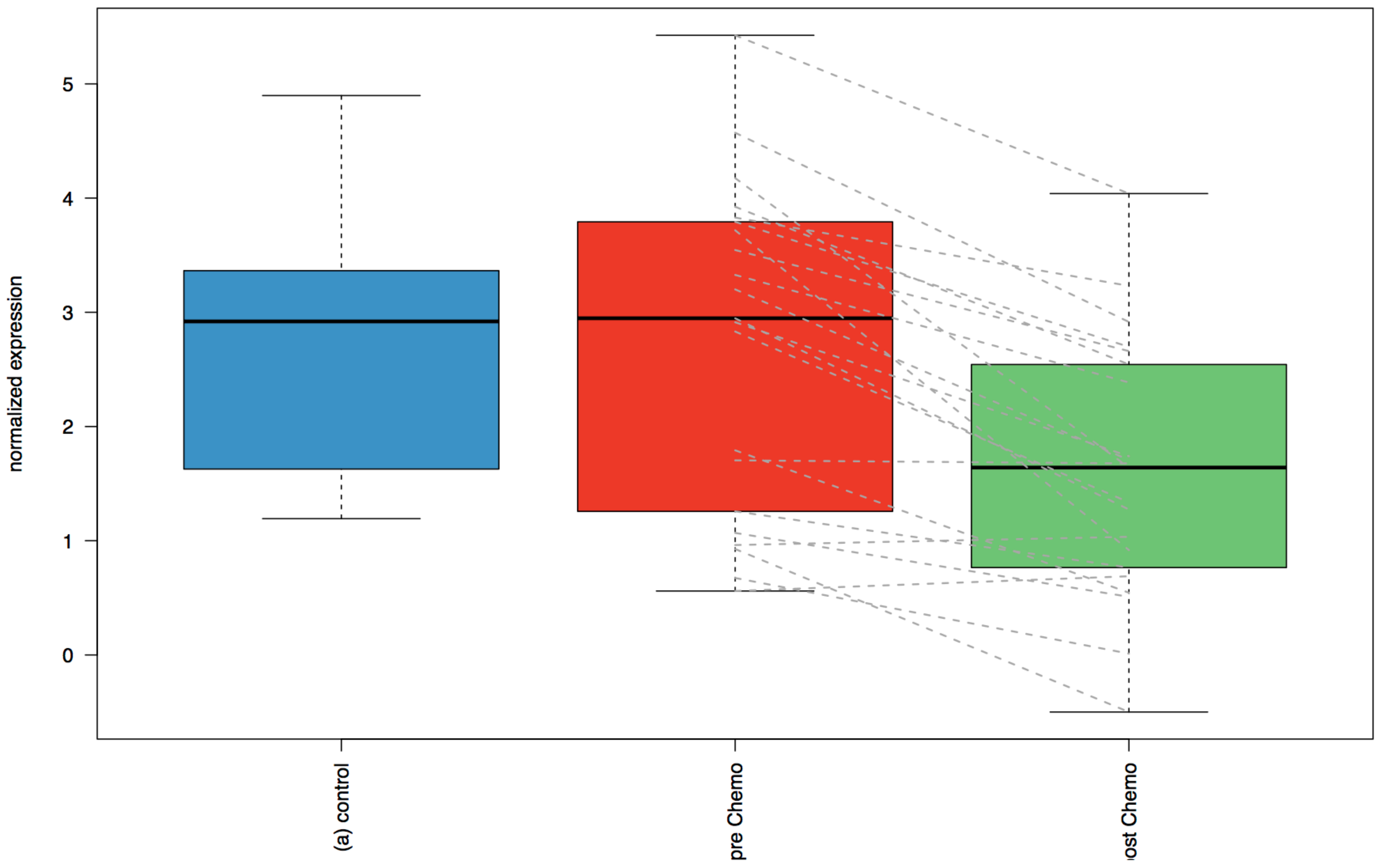
normalized expression
5
4
3
2
1
0
(a) control
pre Chemo
ost Chemo

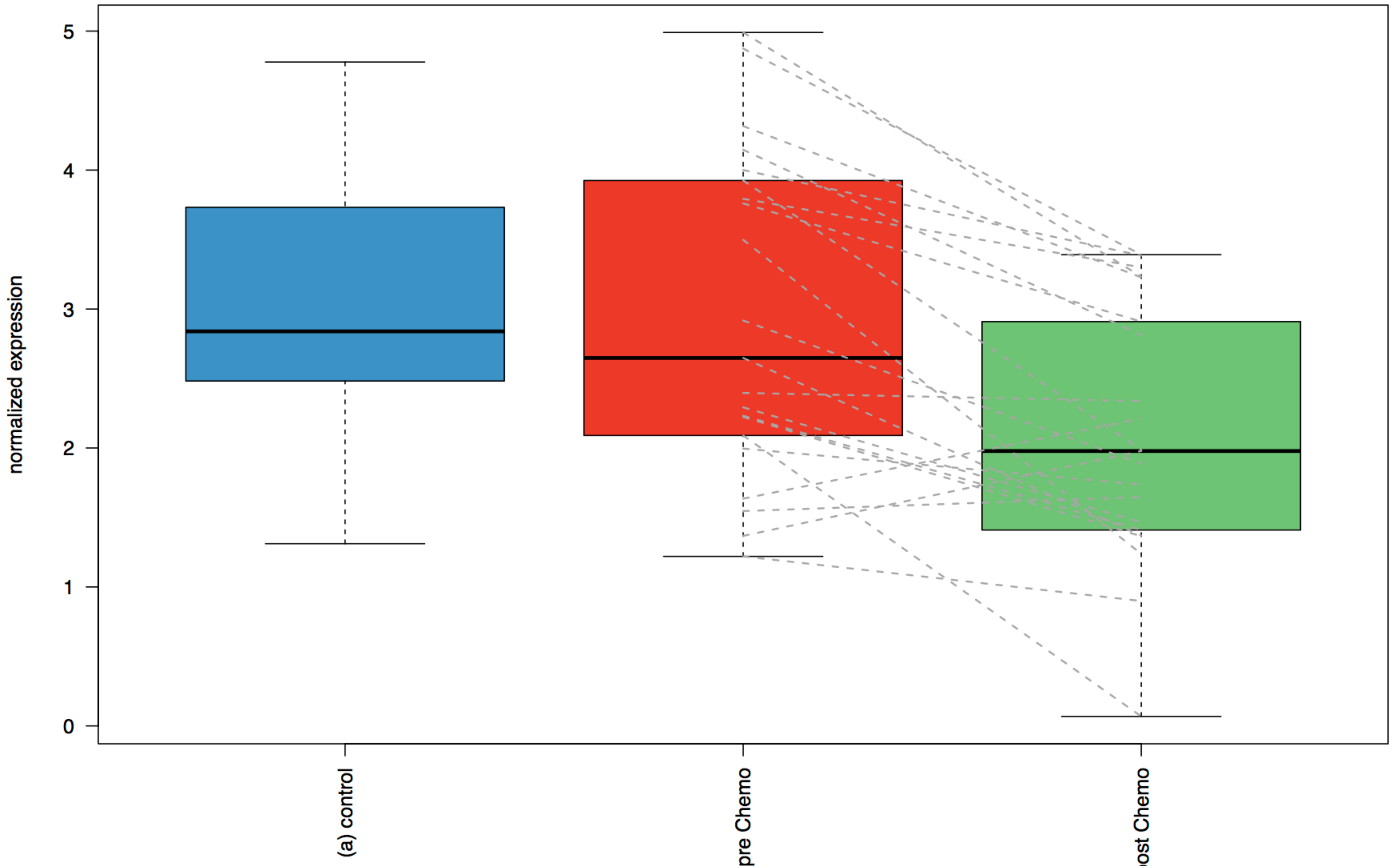

normalized expression
0
1
2
3
4
5
(a) control
pre Chemo
ost Chemo

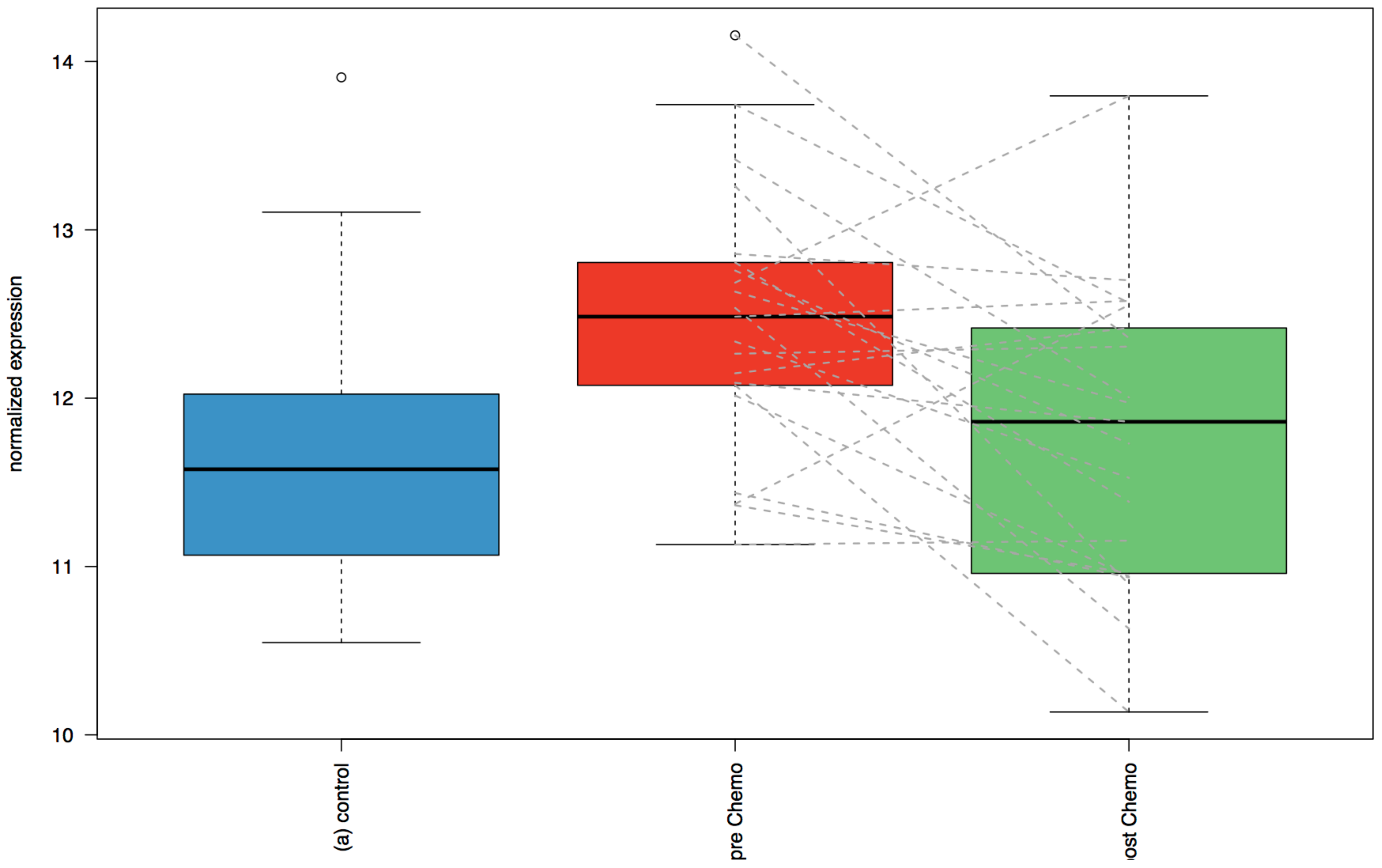

normalized expression
14
13
12
11
10
(a) control
pre Chemo
ost Chemo

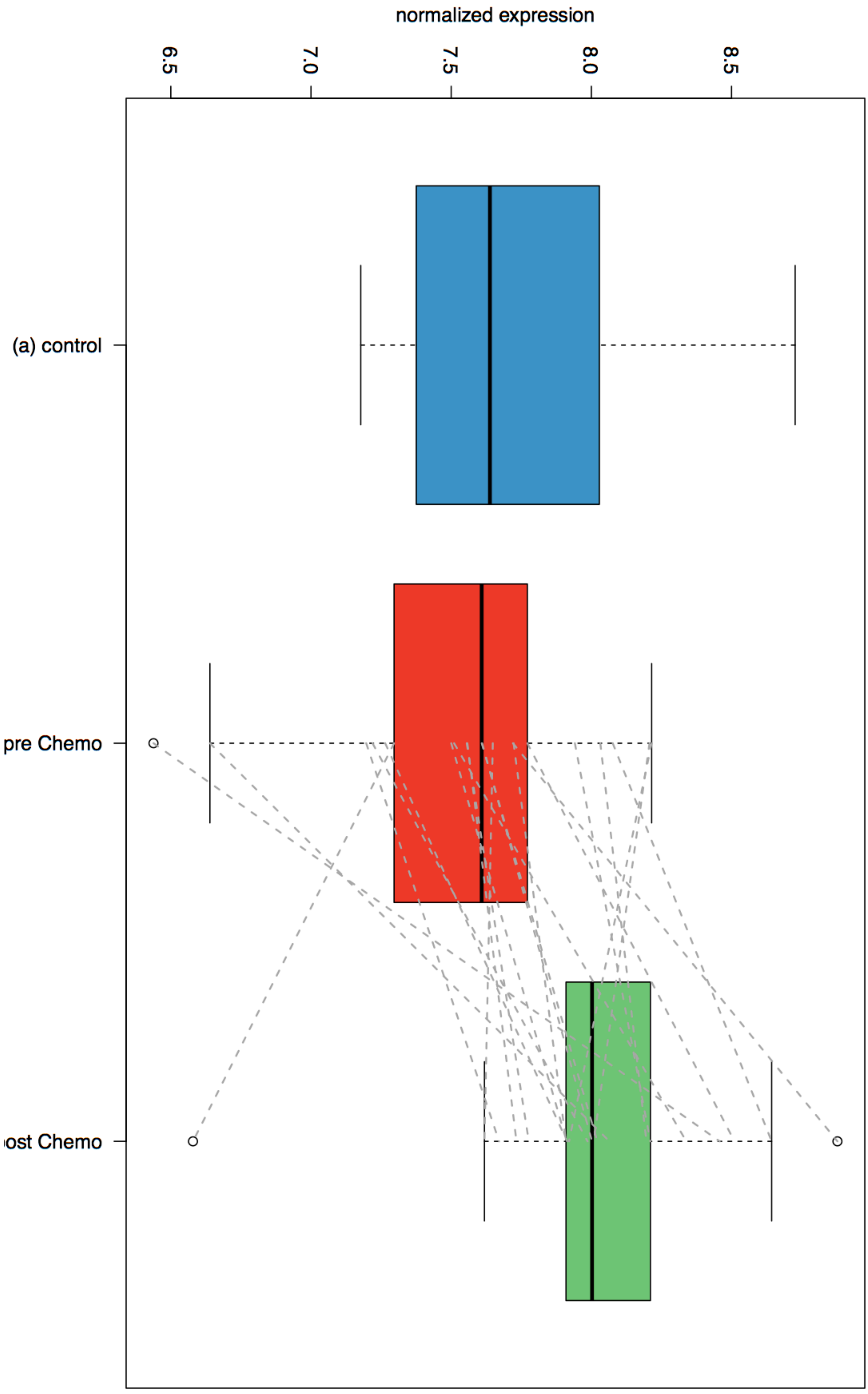
normalized expression
6.5
7.0
7.5
8.0
8.5
(a) control
pre Chemo
ost Chemo

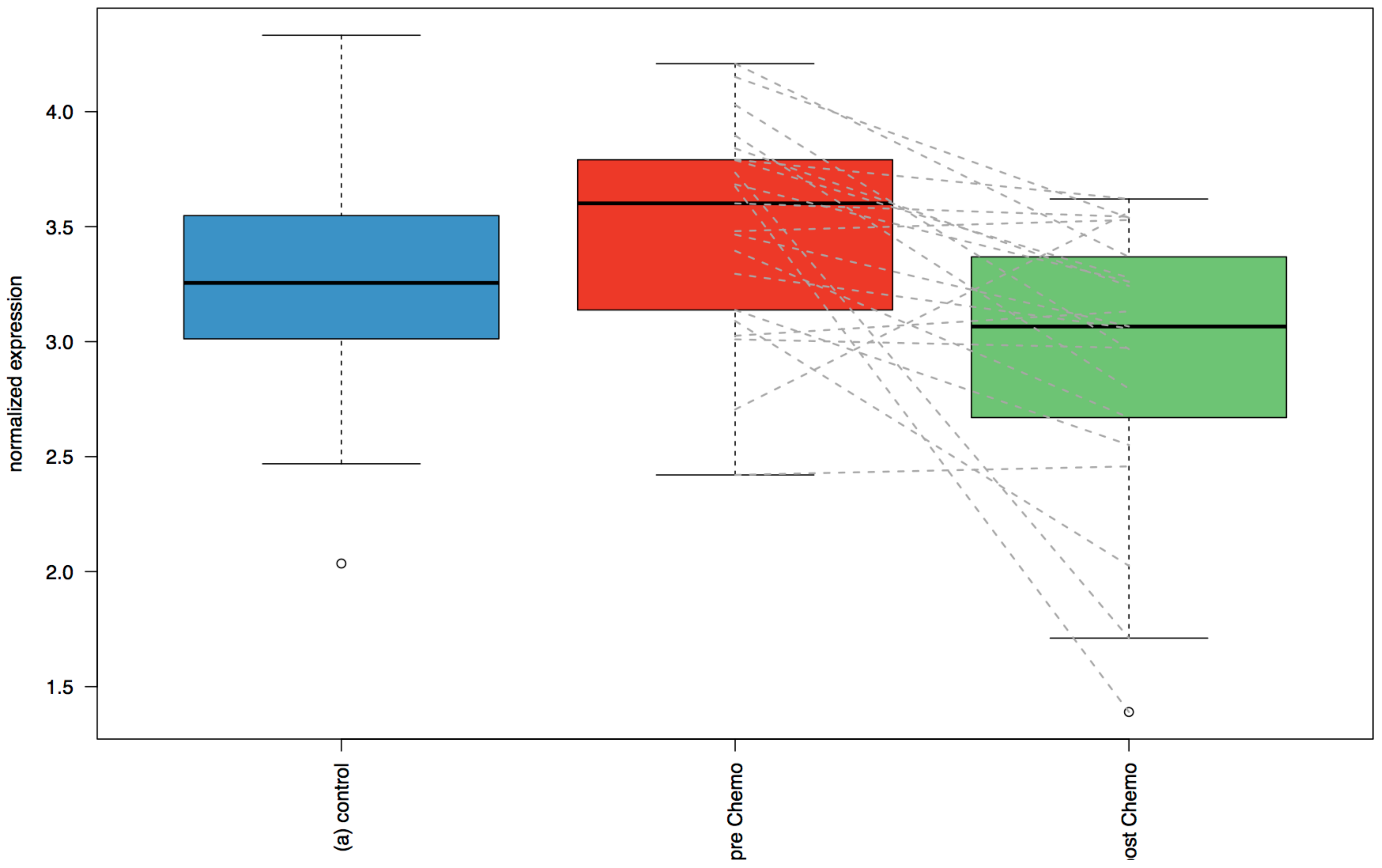
normalized expression
4.0
3.5
3.0
2.5
2.0
1.5
(a) control
pre Chemo
ost Chemo

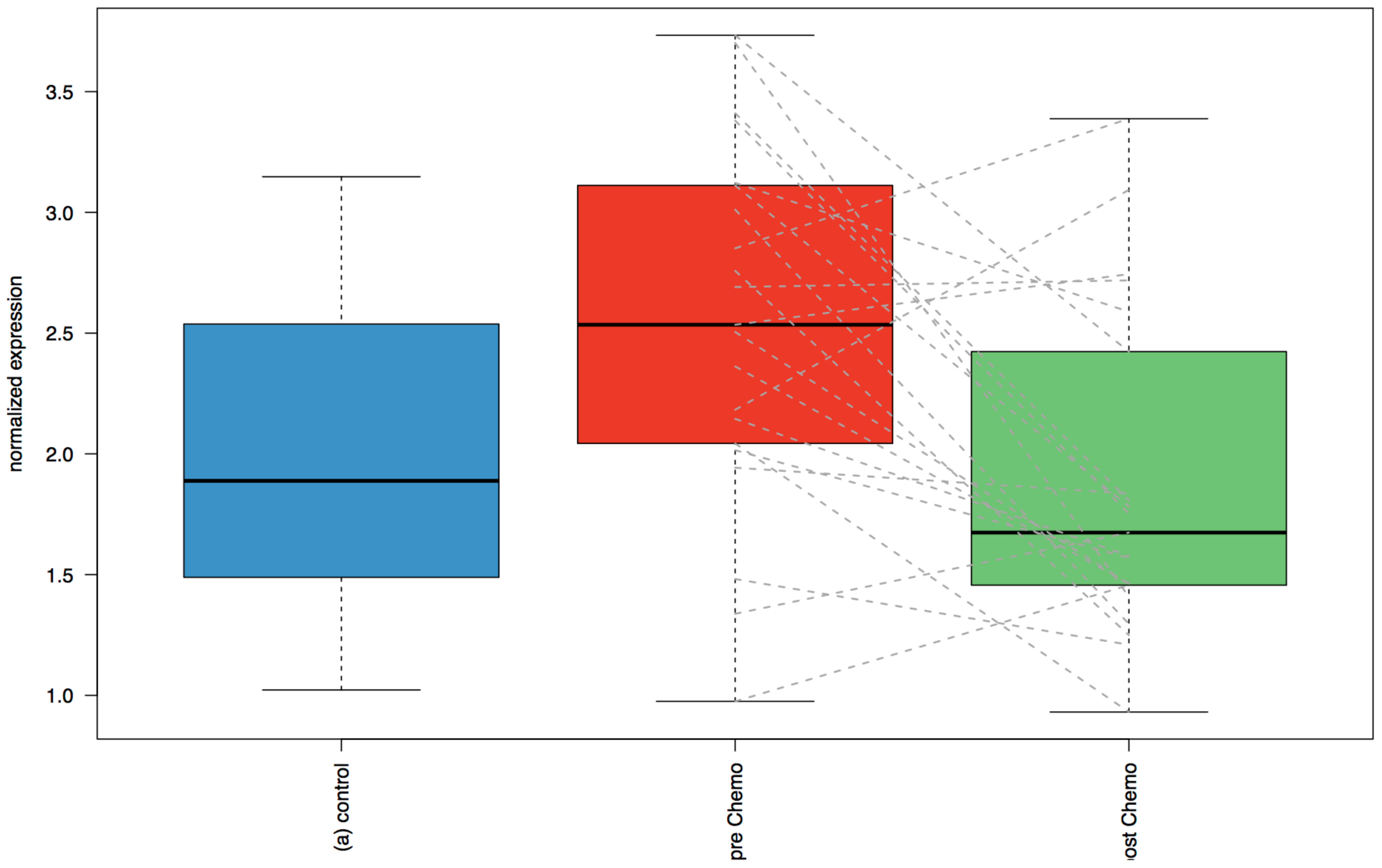
normalized expression
3.5
3.0
2.5
2.0
1.5
1.0
(a) control
pre Chemo
ost Chemo

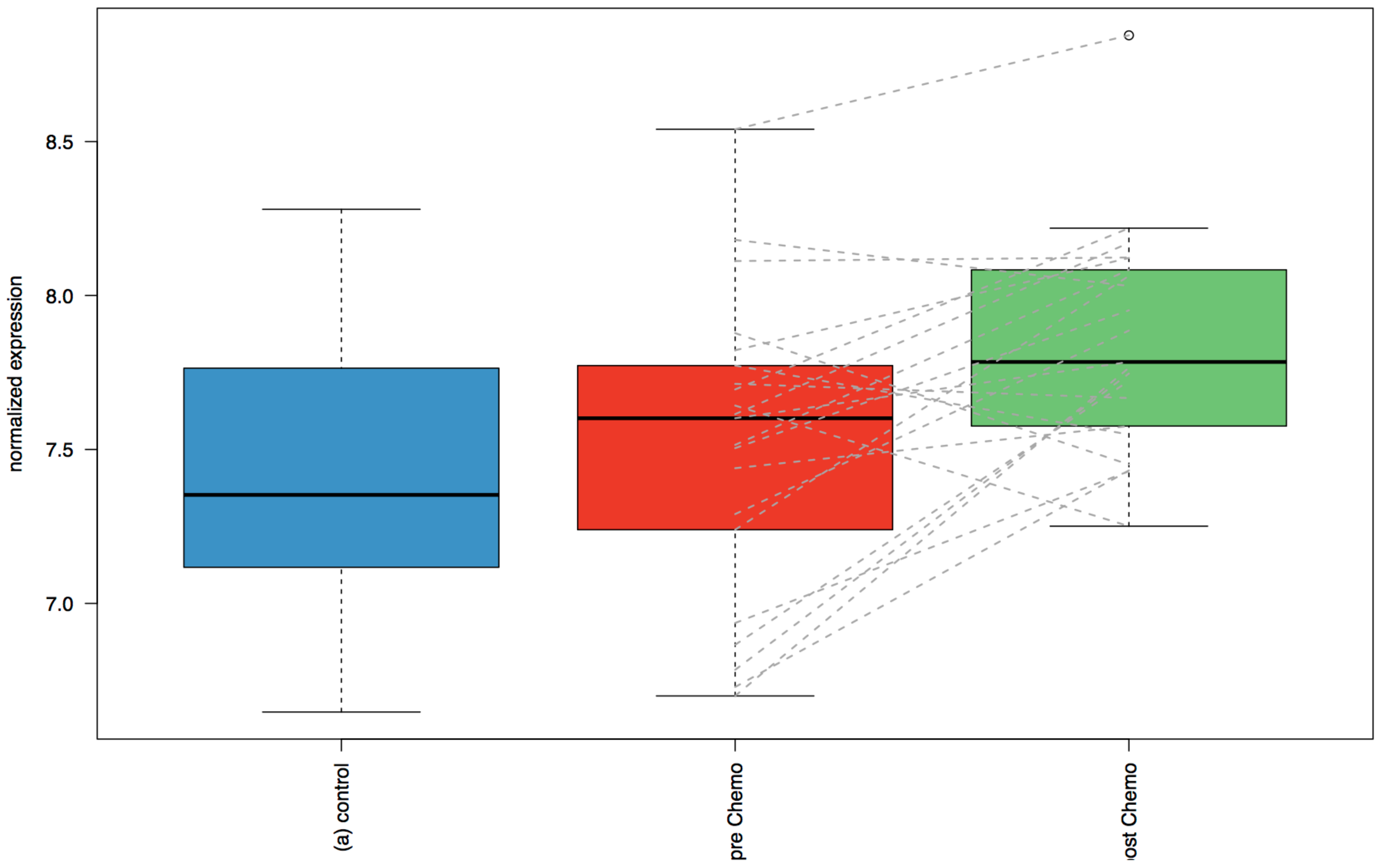
normalized expression
8.5
8.0
7.5
7.0
(a) control
pre Chemo
ost Chemo

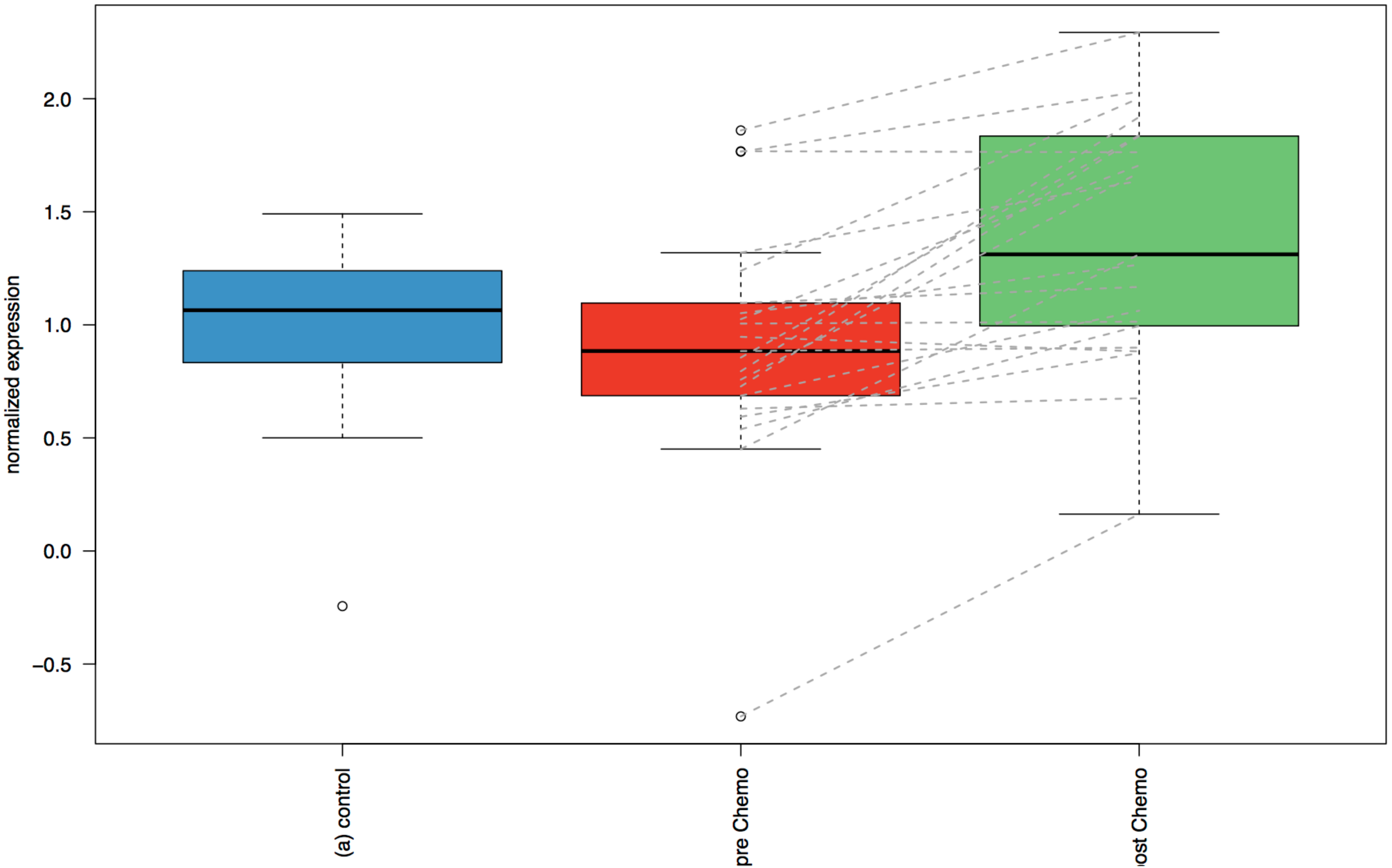
normalized expression
2.0
1.5
1.0
0.5
0.0
−0.5
(a) control
pre Chemo
ost Chemo

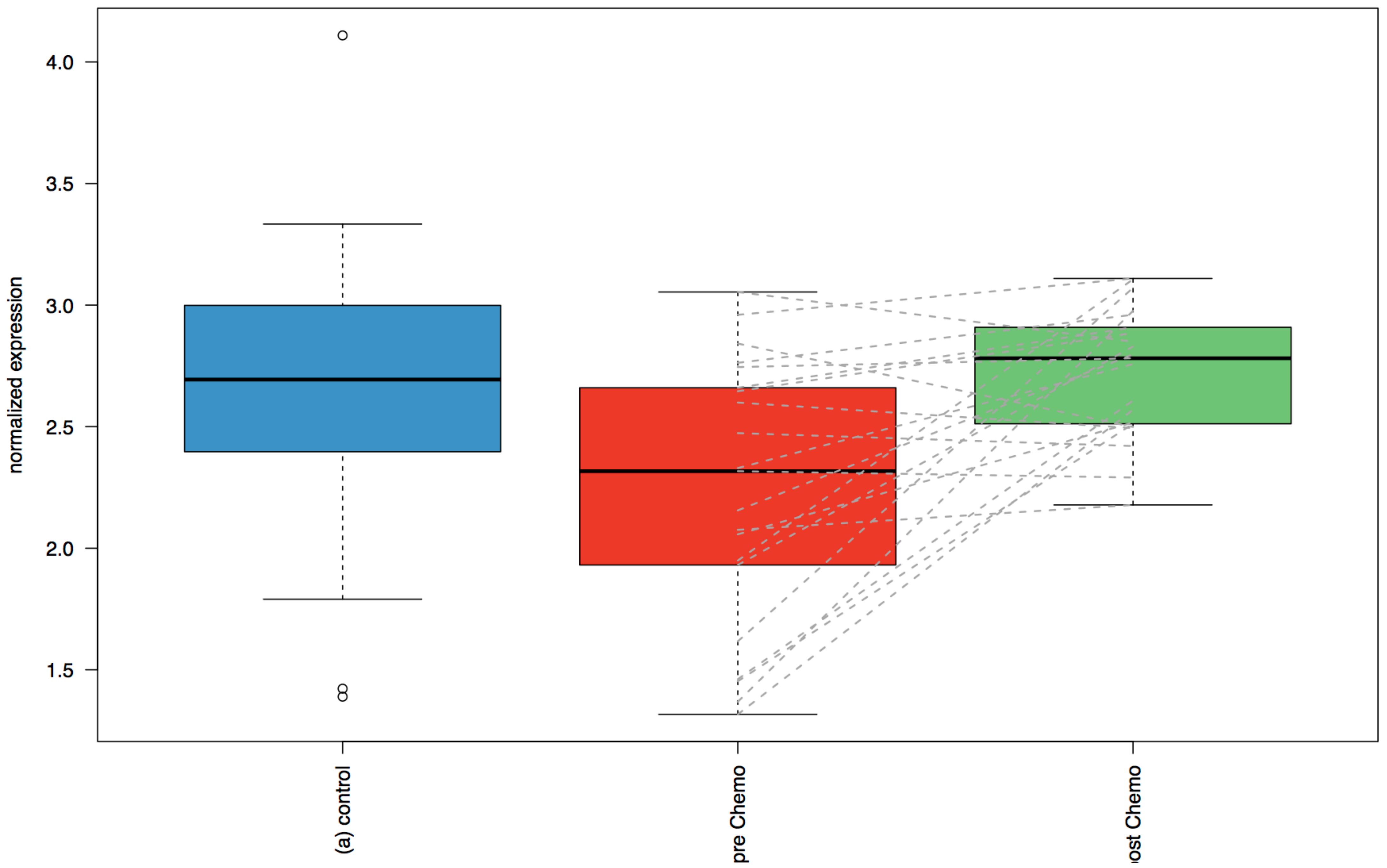
normalized expression
4.0
3.5
3.0
2.5
2.0
1.5
(a) control
pre Chemo
ost Chemo

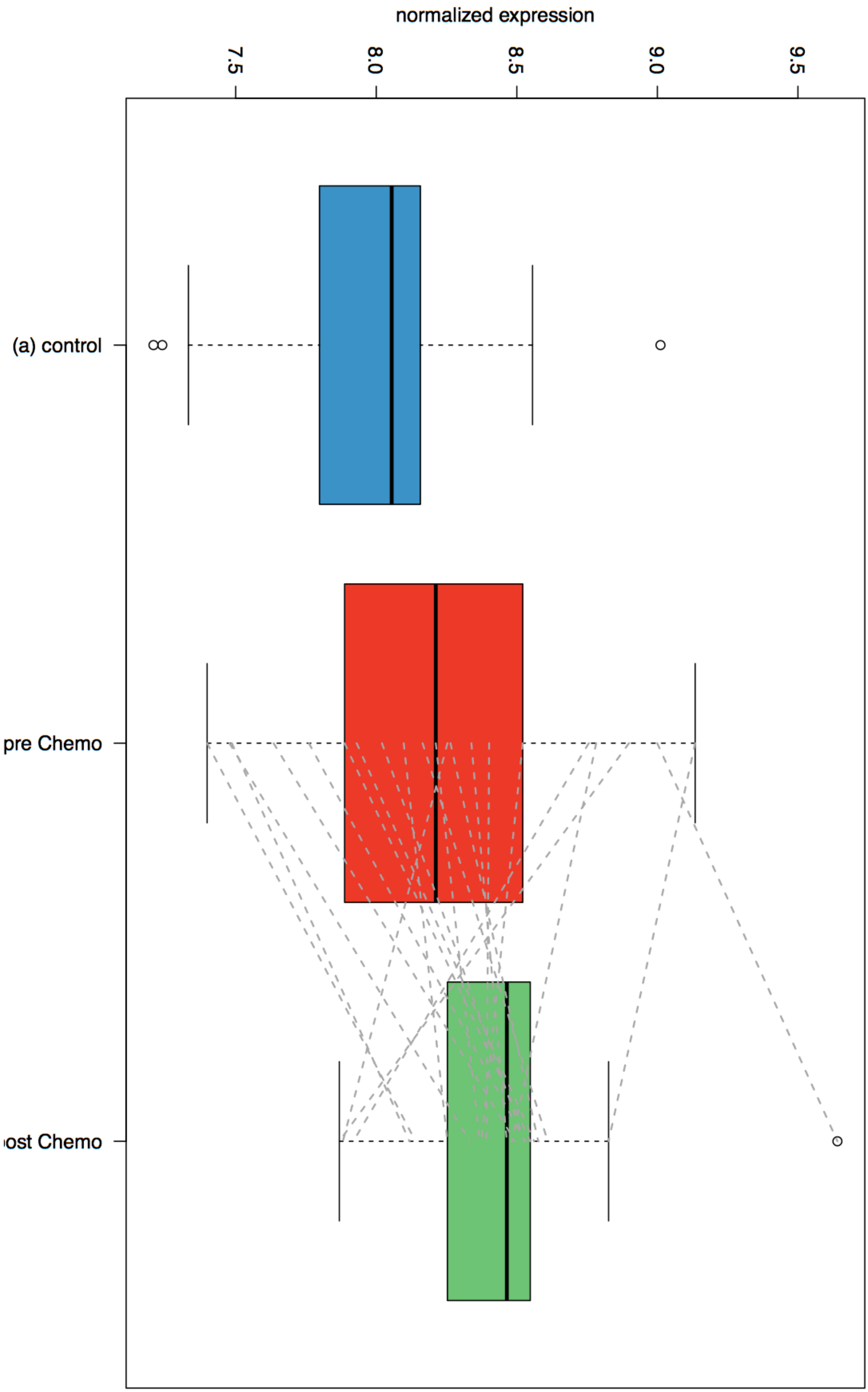
normalized expression
7.5
8.0
8.5
9.0
9.5
(a) control
pre Chemo
ost Chemo

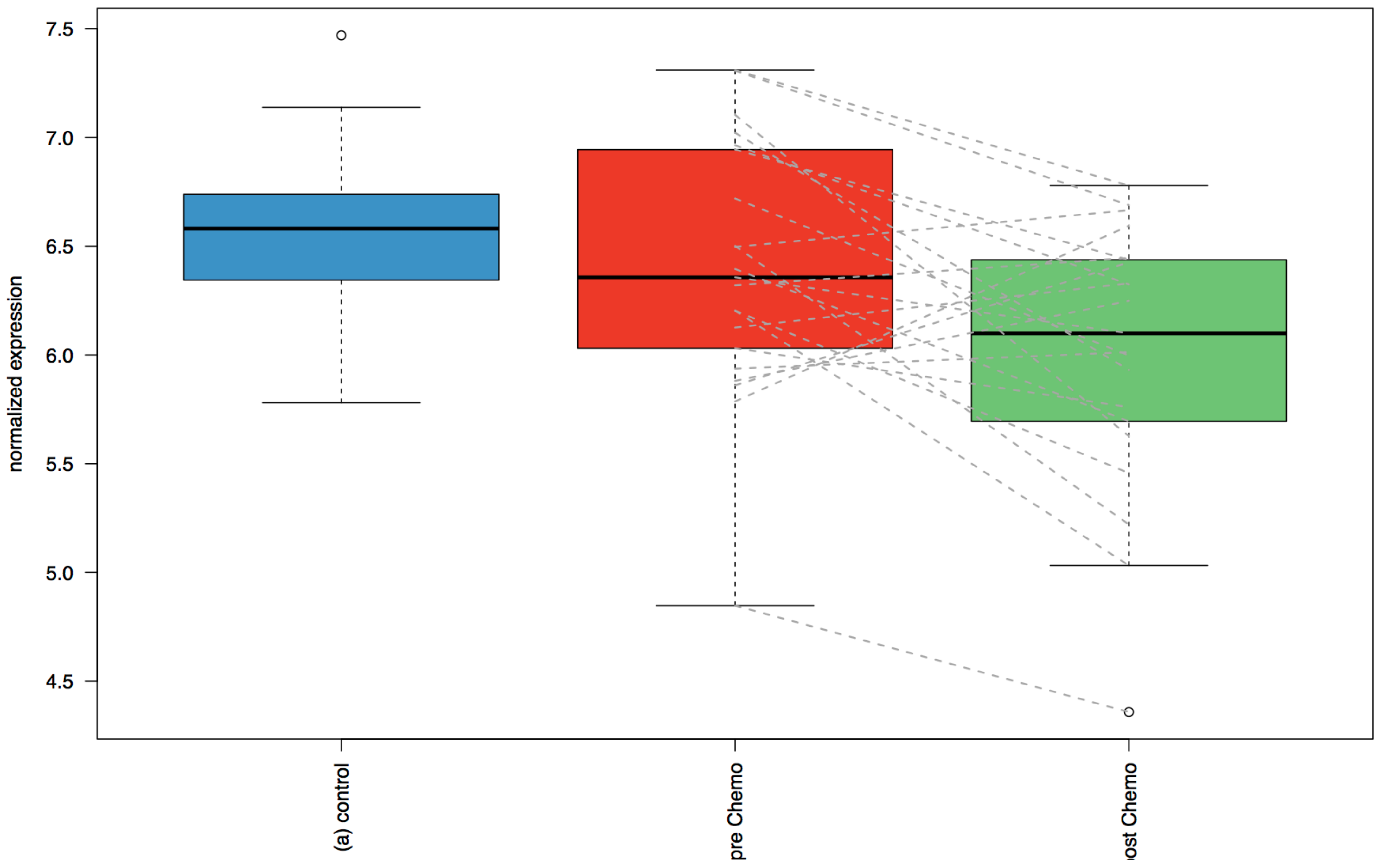

normalized expression
7.5
7.0
6.5
6.0
5.5
5.0
4.5
(a) control
pre Chemo
ost Chemo

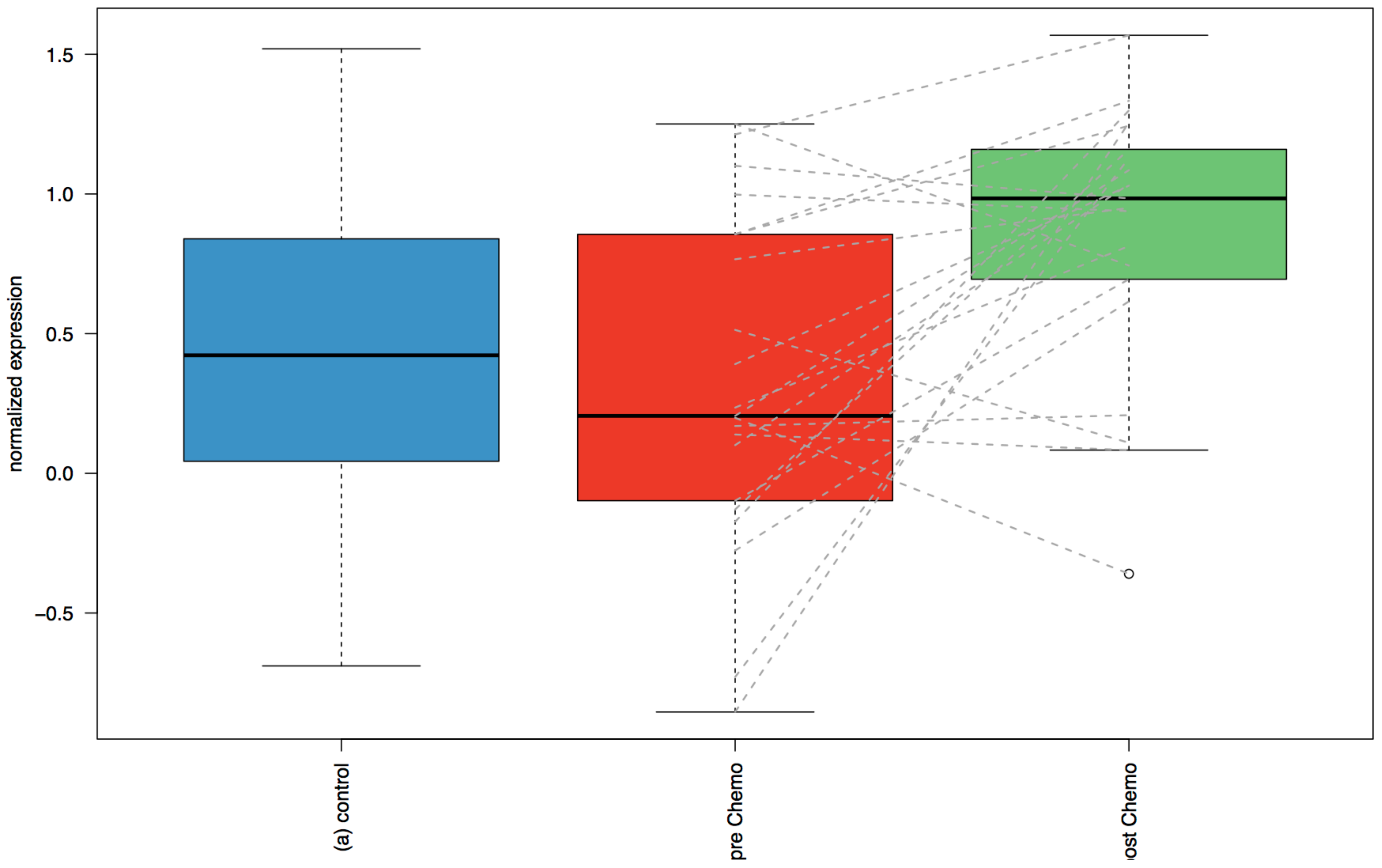
normalized expression
1.5
1.0
0.5
0.0
−0.5
(a) control
pre Chemo
ost Chemo

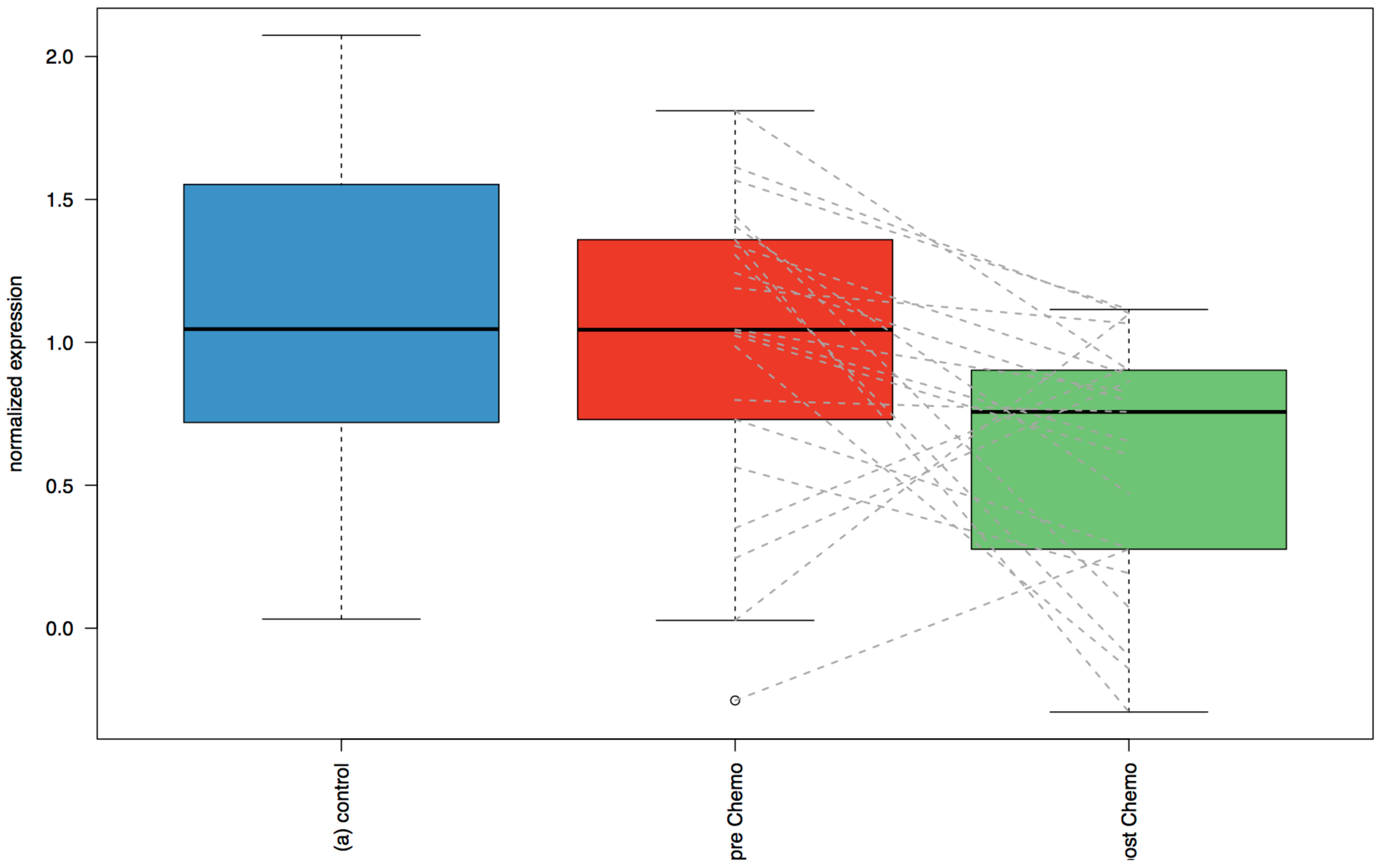
normalized expression
2.0
1.5
1.0
0.5
0.0
(a) control
pre Chemo
ost Chemo

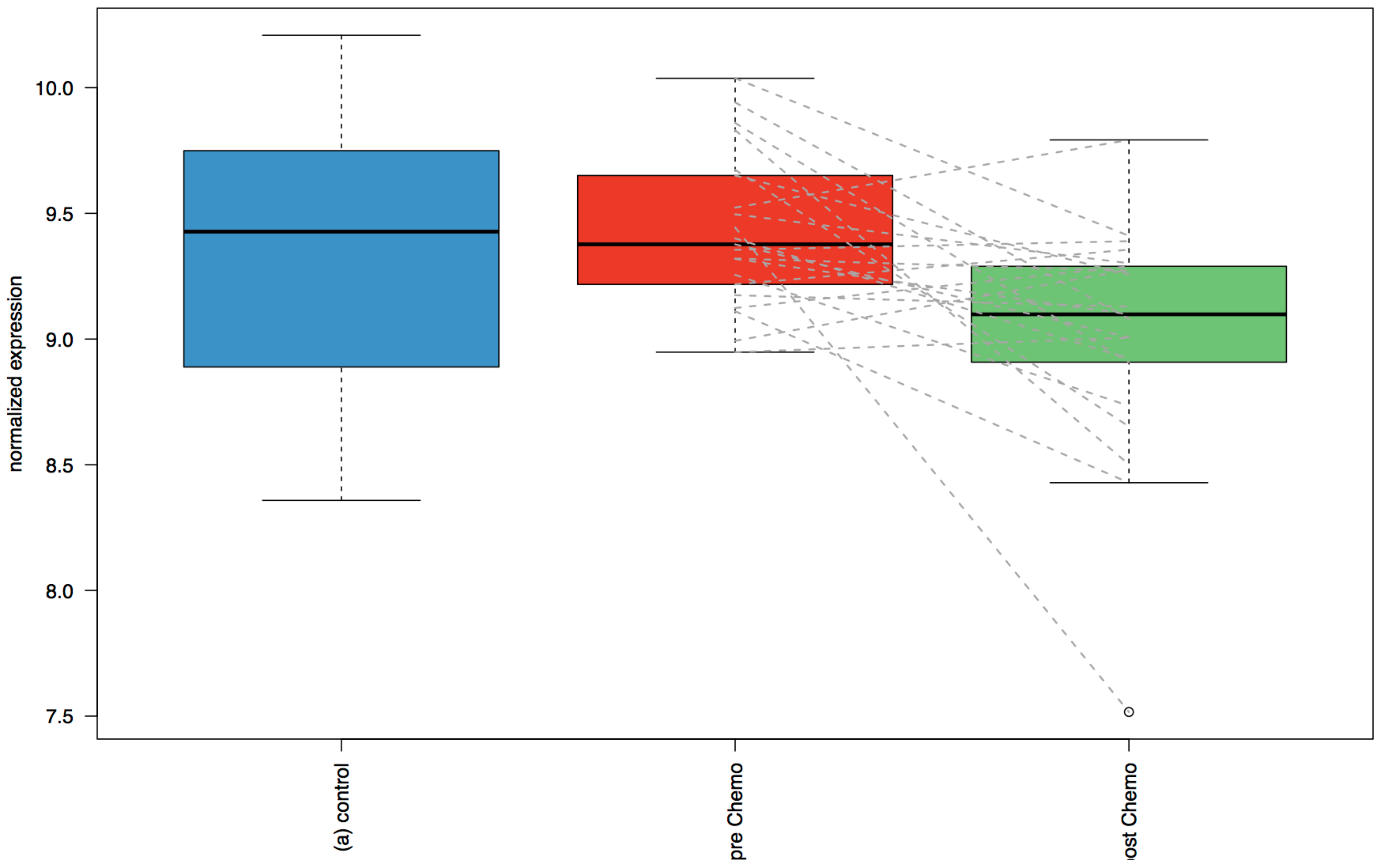
normalized expression
10.0
9.5
9.0
8.5
8.0
7.5
(a) control
pre Chemo
ost Chemo

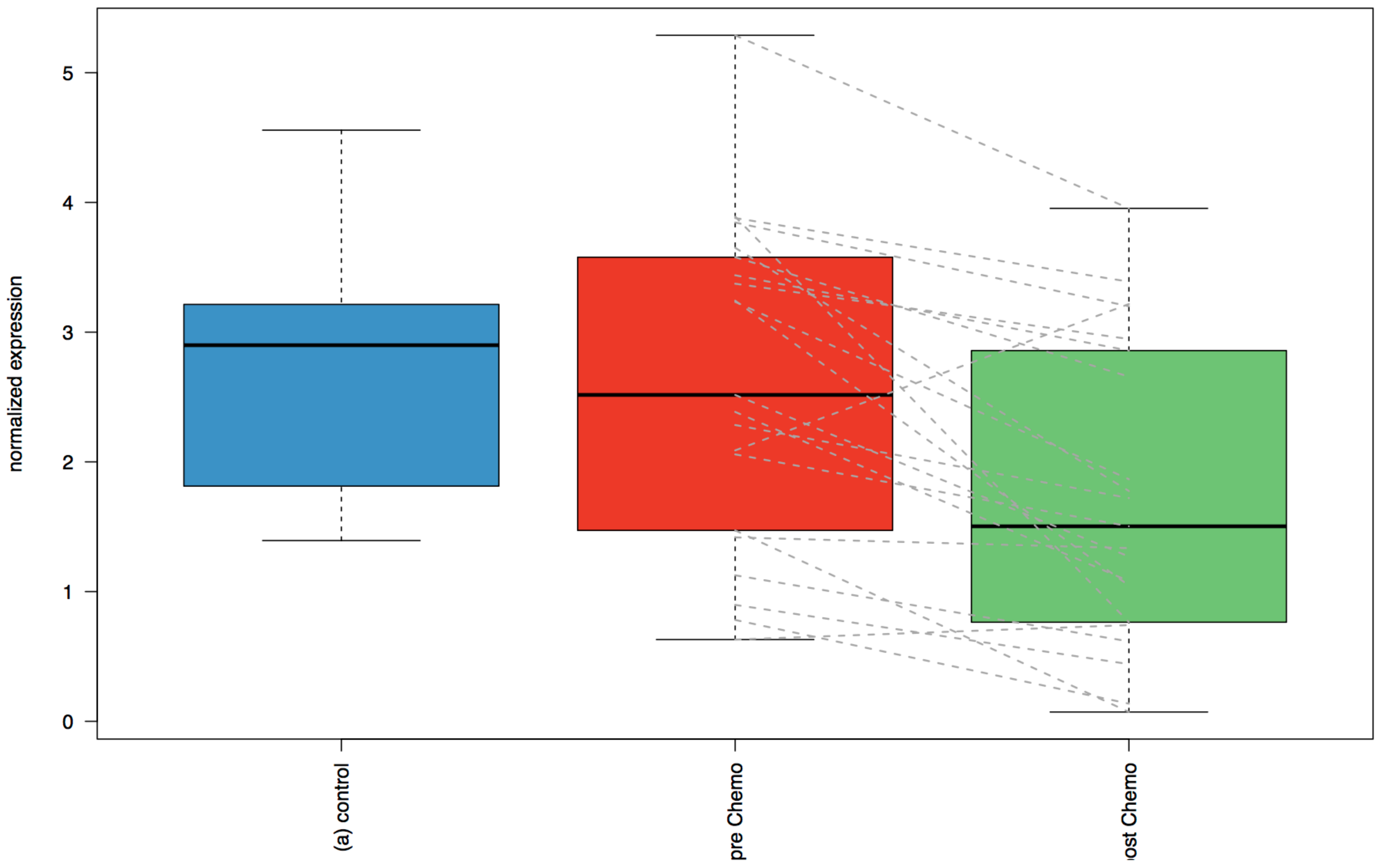

normalized expression
0
1
2
3
4
5
(a) control
pre Chemo
ost Chemo

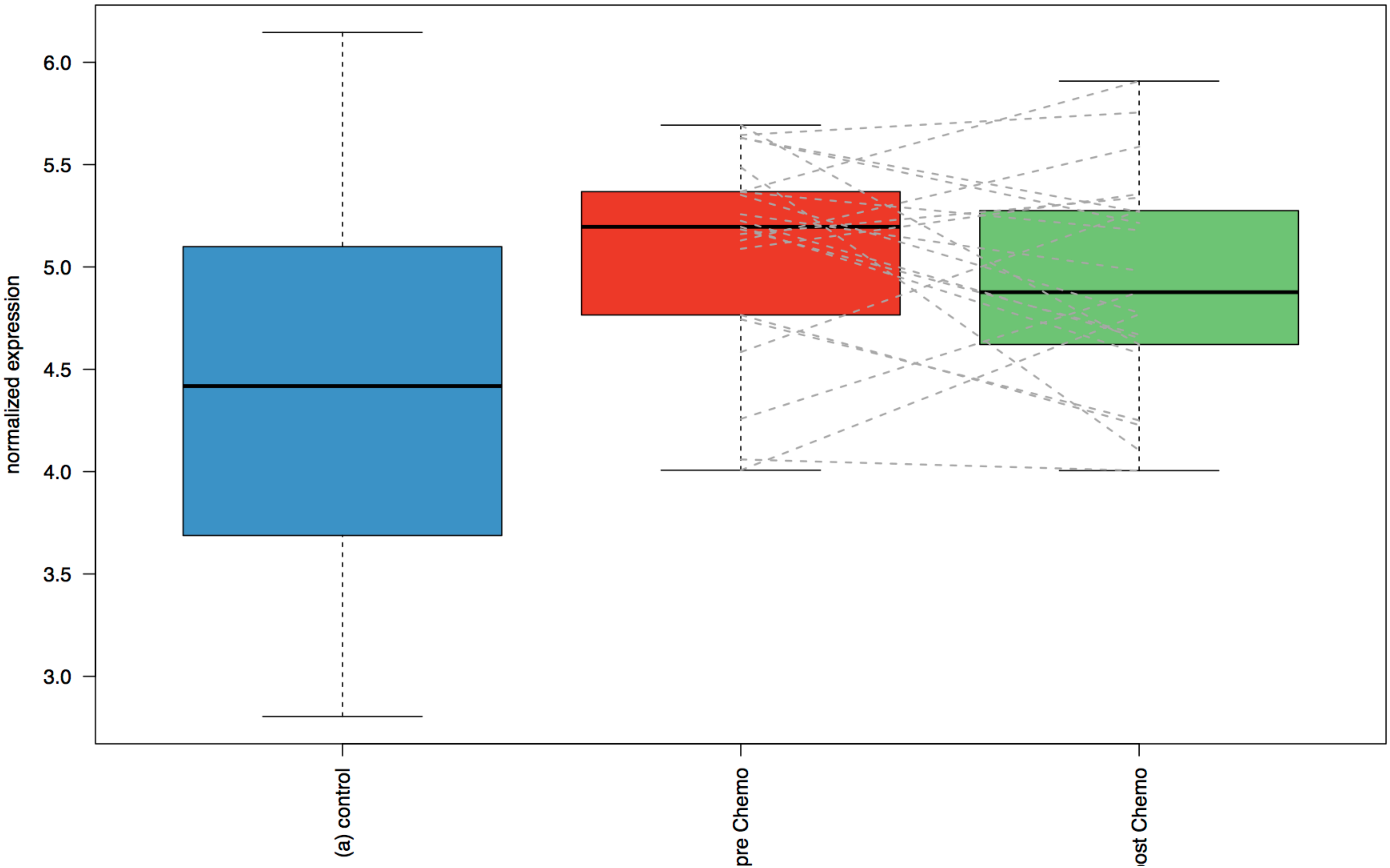

normalized expression
6.0
5.5
5.0
4.5
4.0
3.5
3.0
(a) control
pre Chemo
ost Chemo

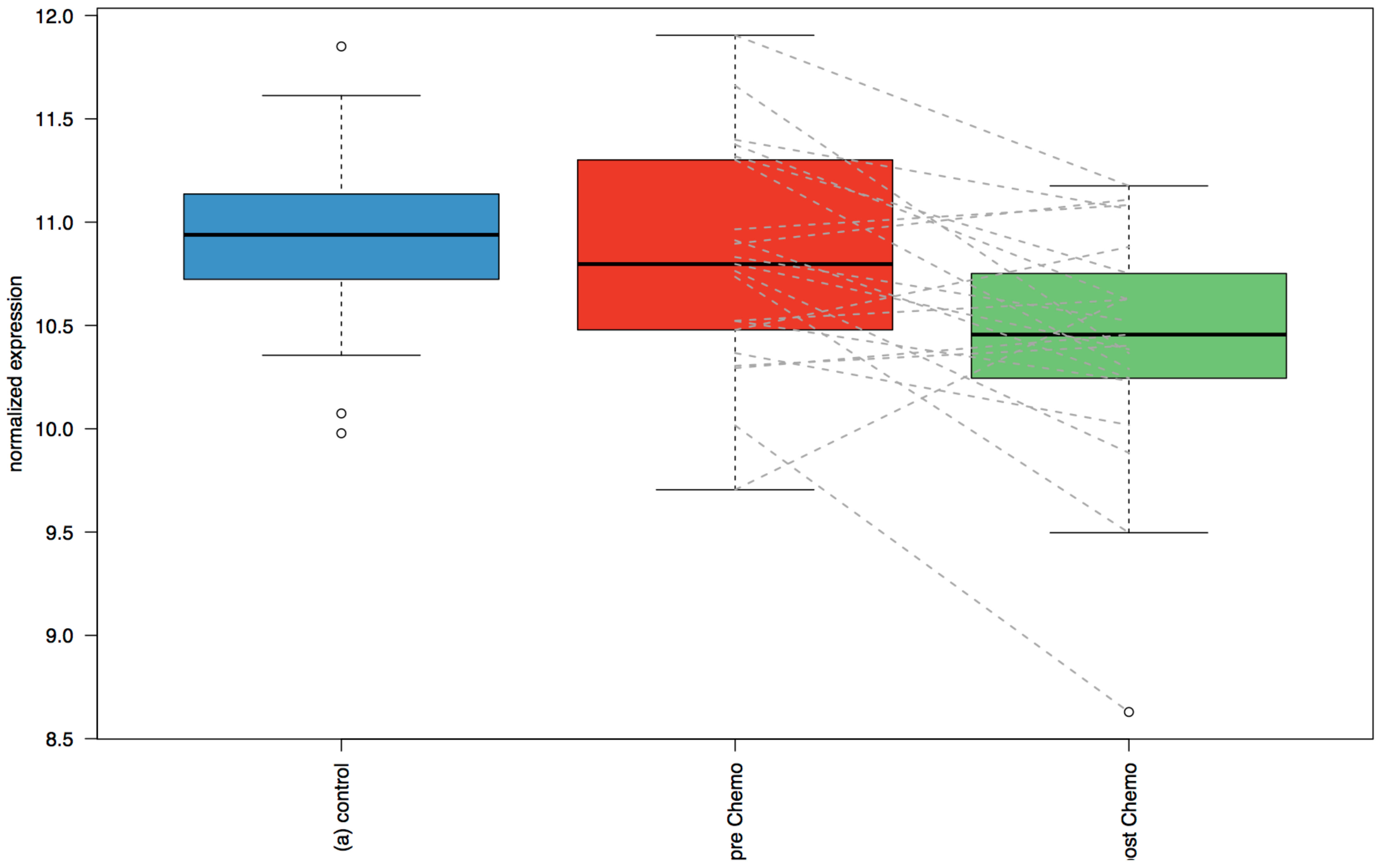

normalized expression
12.0
11.5
11.0
10.5
10.0
9.5
9.0
8.5
(a) control
pre Chemo
ost Chemo

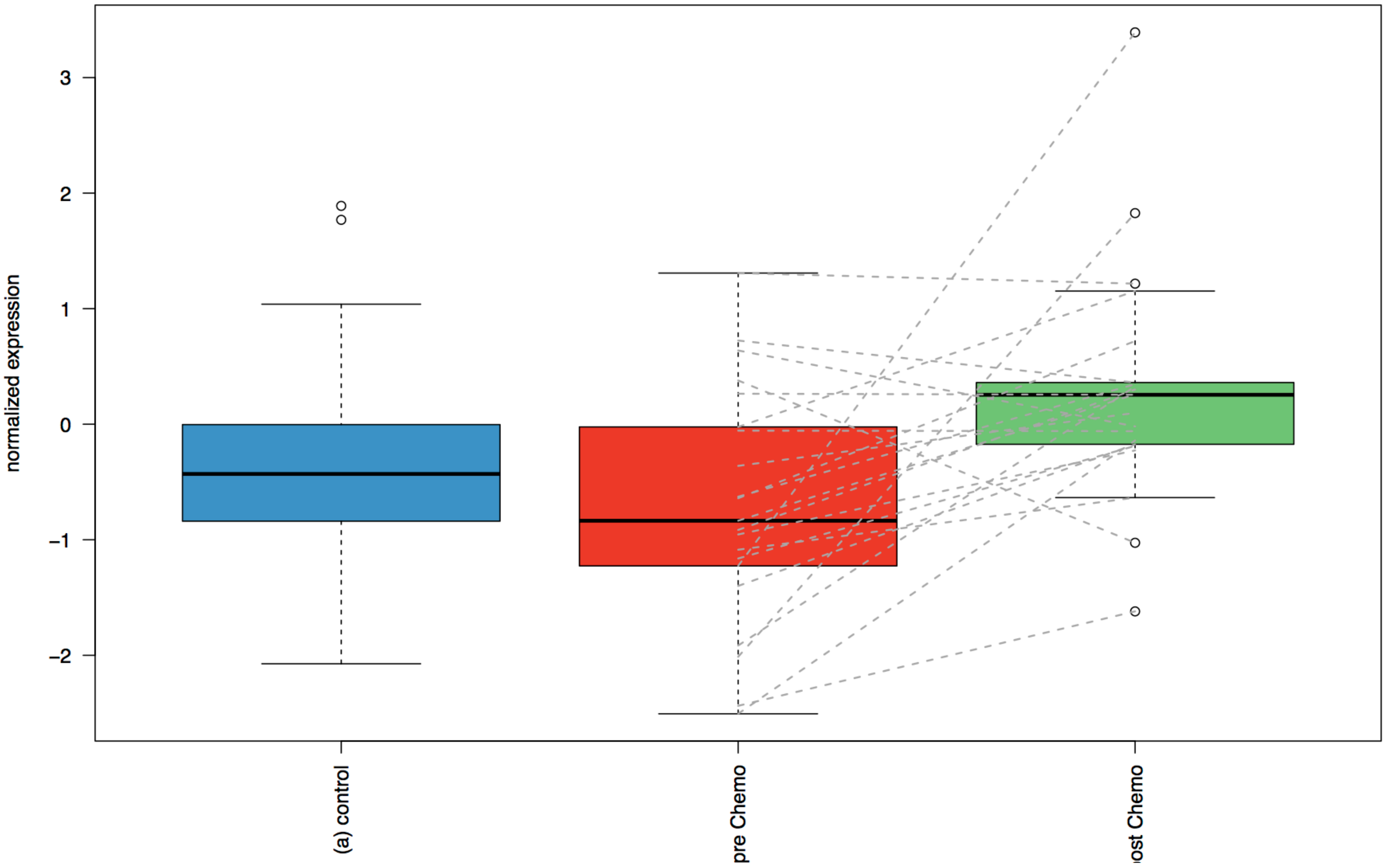
normalized expression
3
2
1
0
−1
−2
(a) control
pre Chemo
ost Chemo

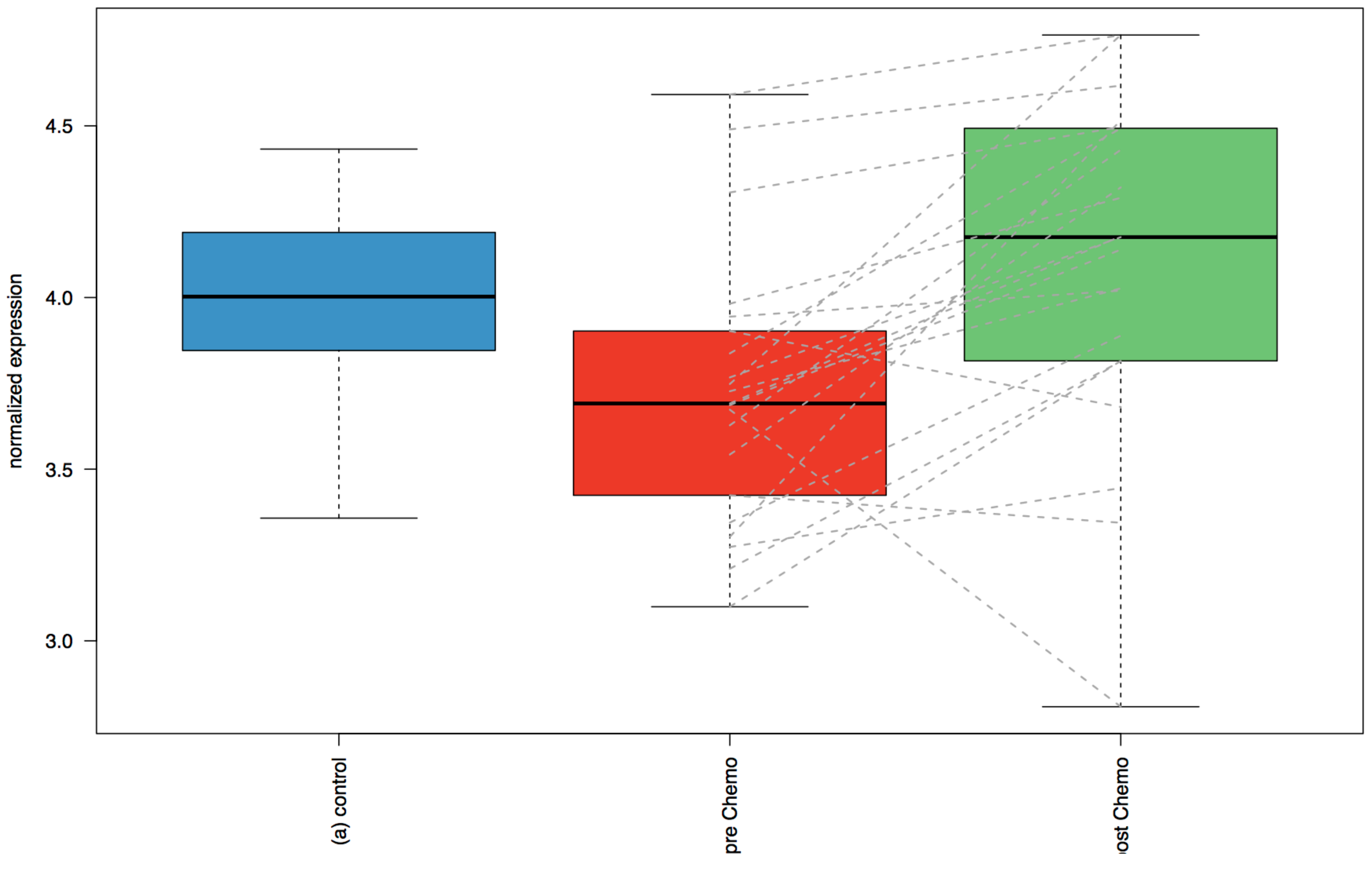
normalized expression
4.5
4.0
3.5
3.0
(a) control
pre Chemo
ost Chemo

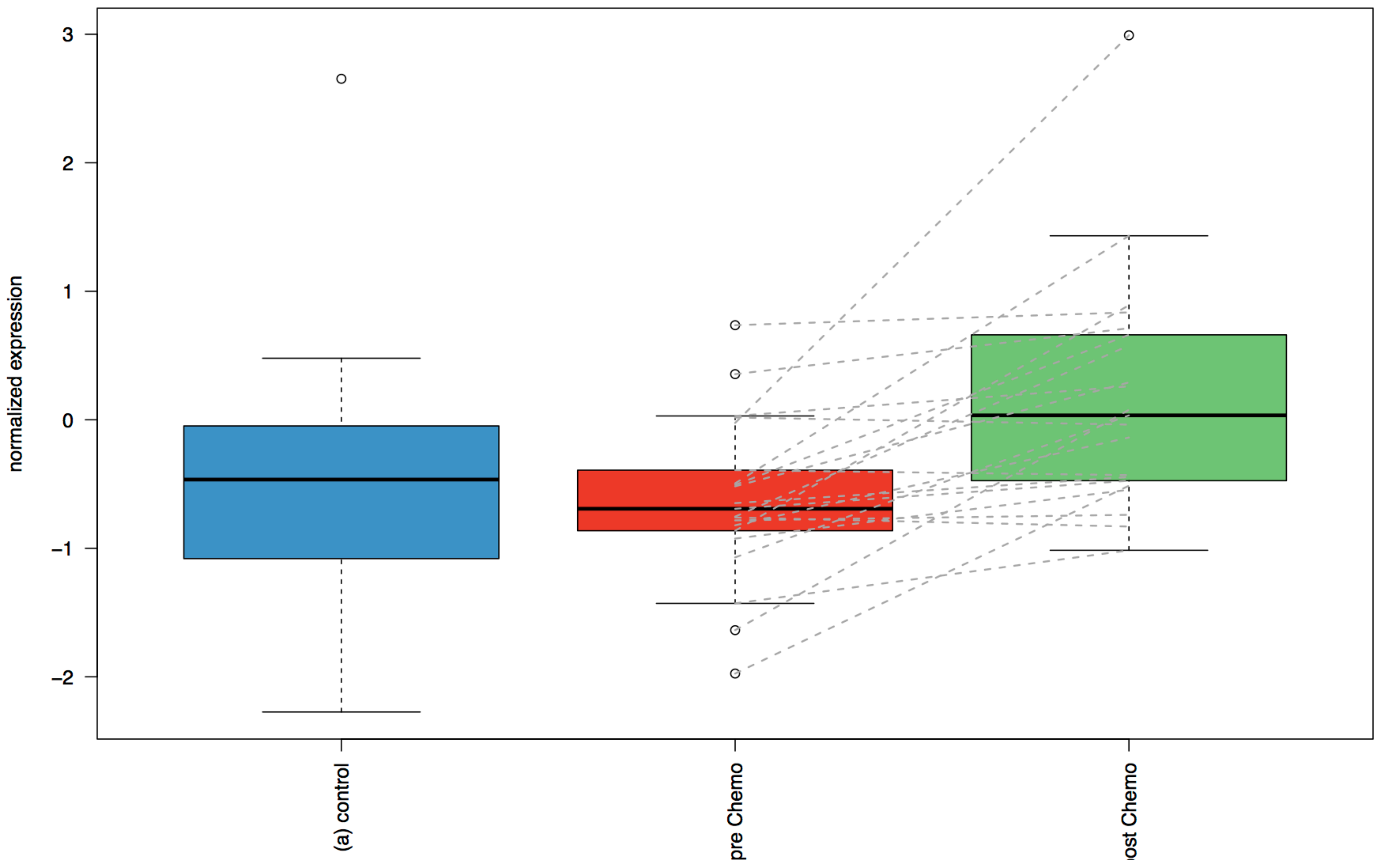
normalized expression
3
2
1
0
−1
−2
(a) control
pre Chemo
ost Chemo

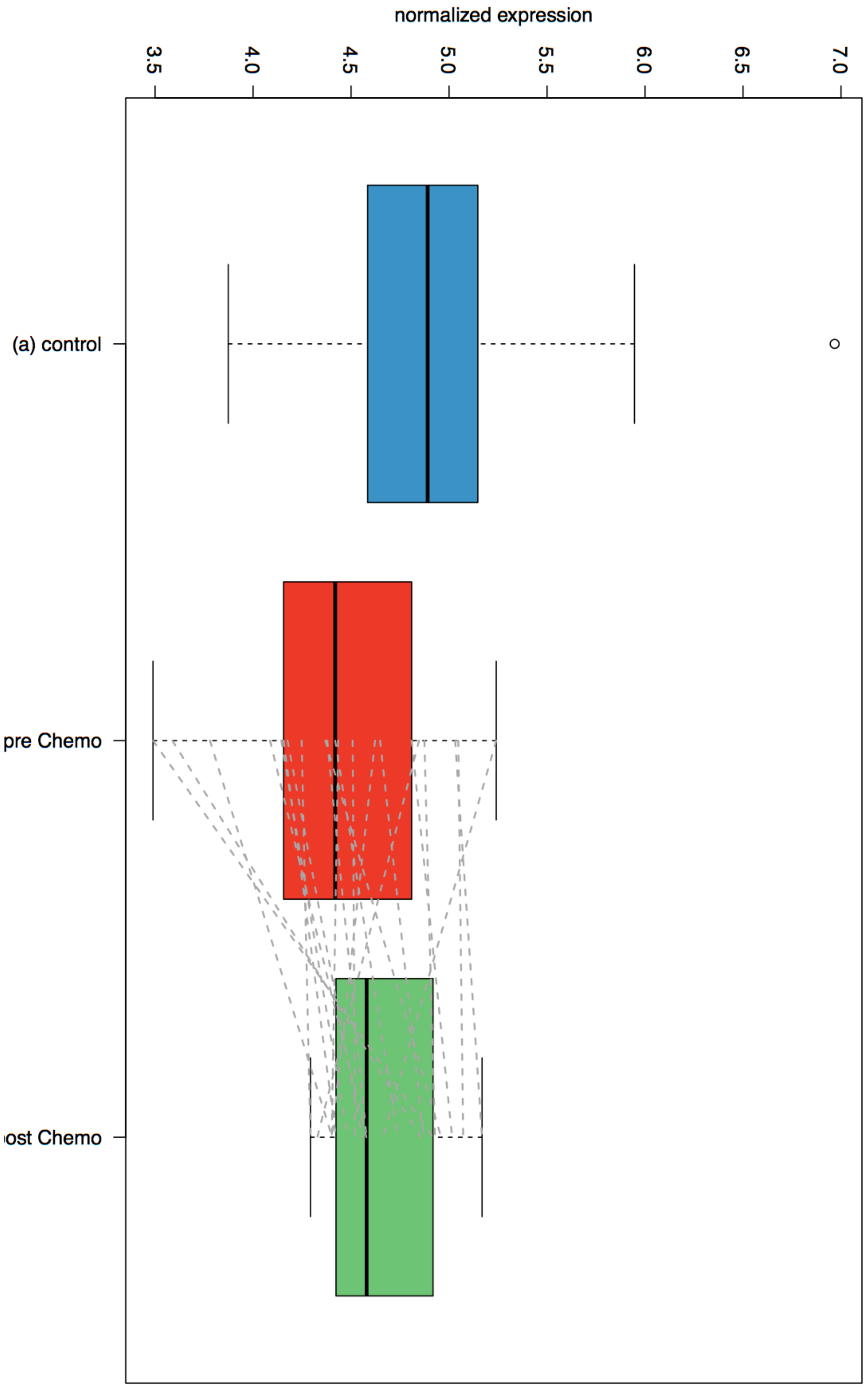
normalized expression
3.5
4.0
4.5
5.0
5.5
6.0
6.5
7.0
(a) control
pre Chemo
ost Chemo

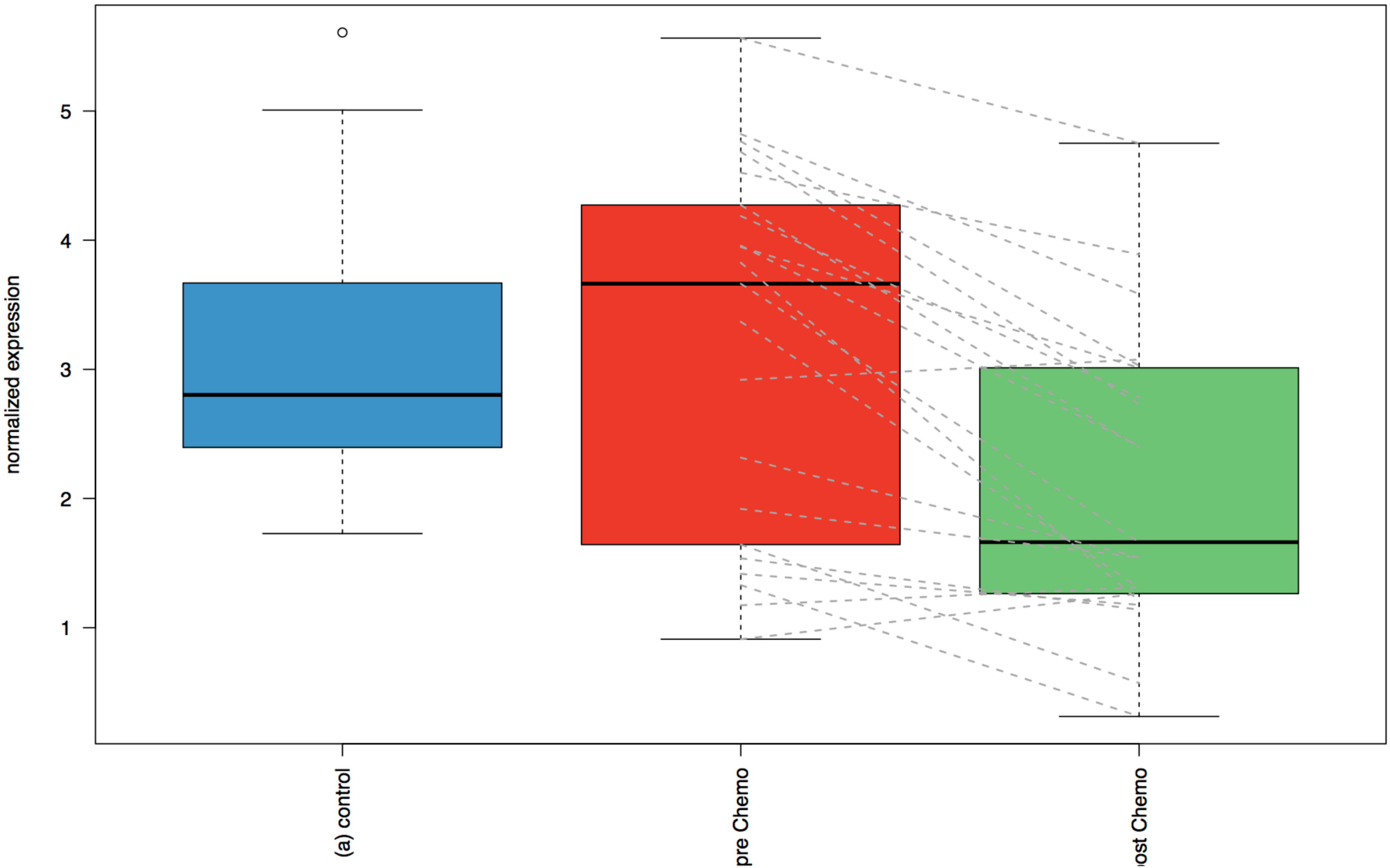
normalized expression
5
4
3
2
1
(a) control
pre Chemo
ost Chemo

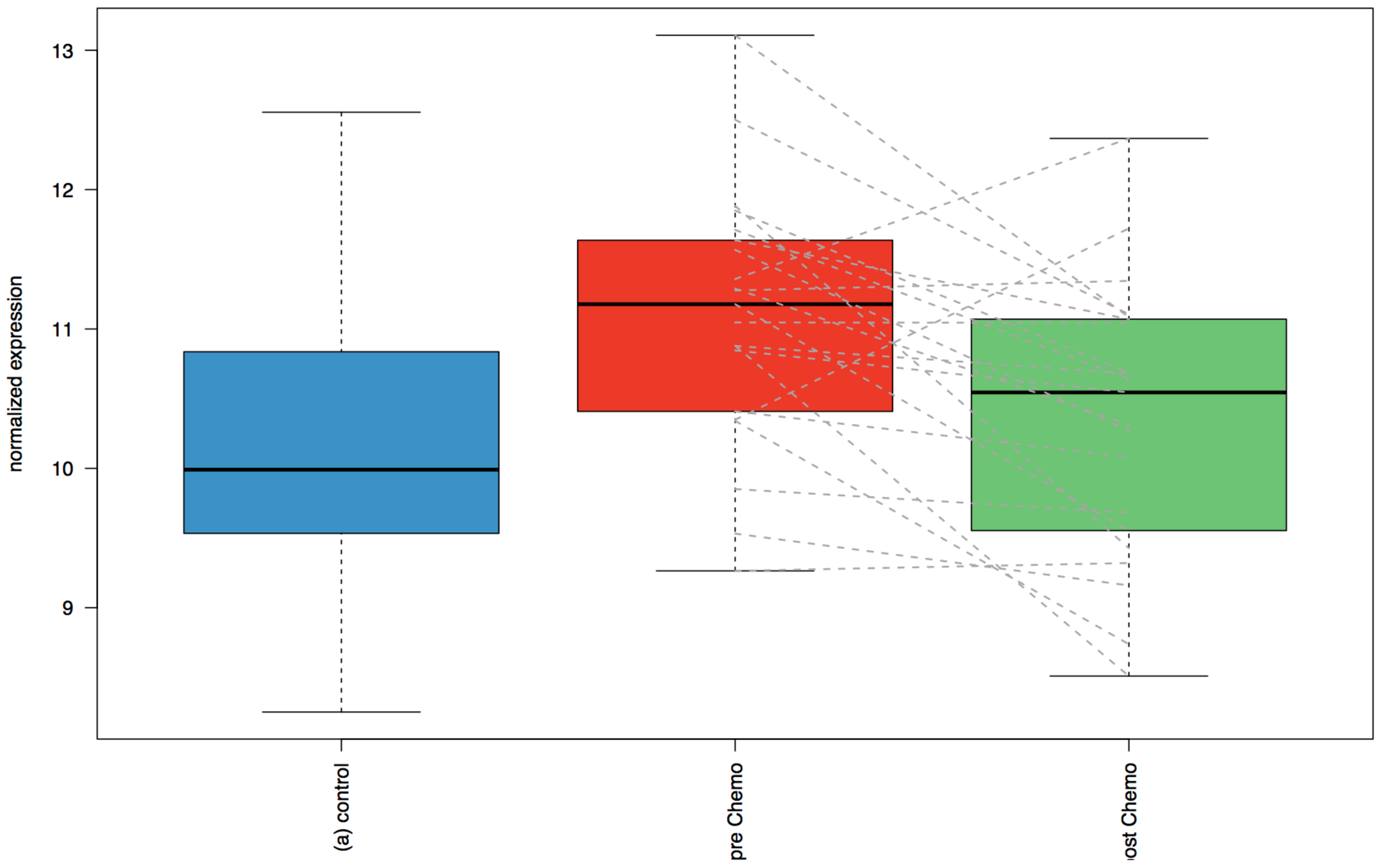
normalized expression
13
12
11
10
9
(a) control
pre Chemo
ost Chemo

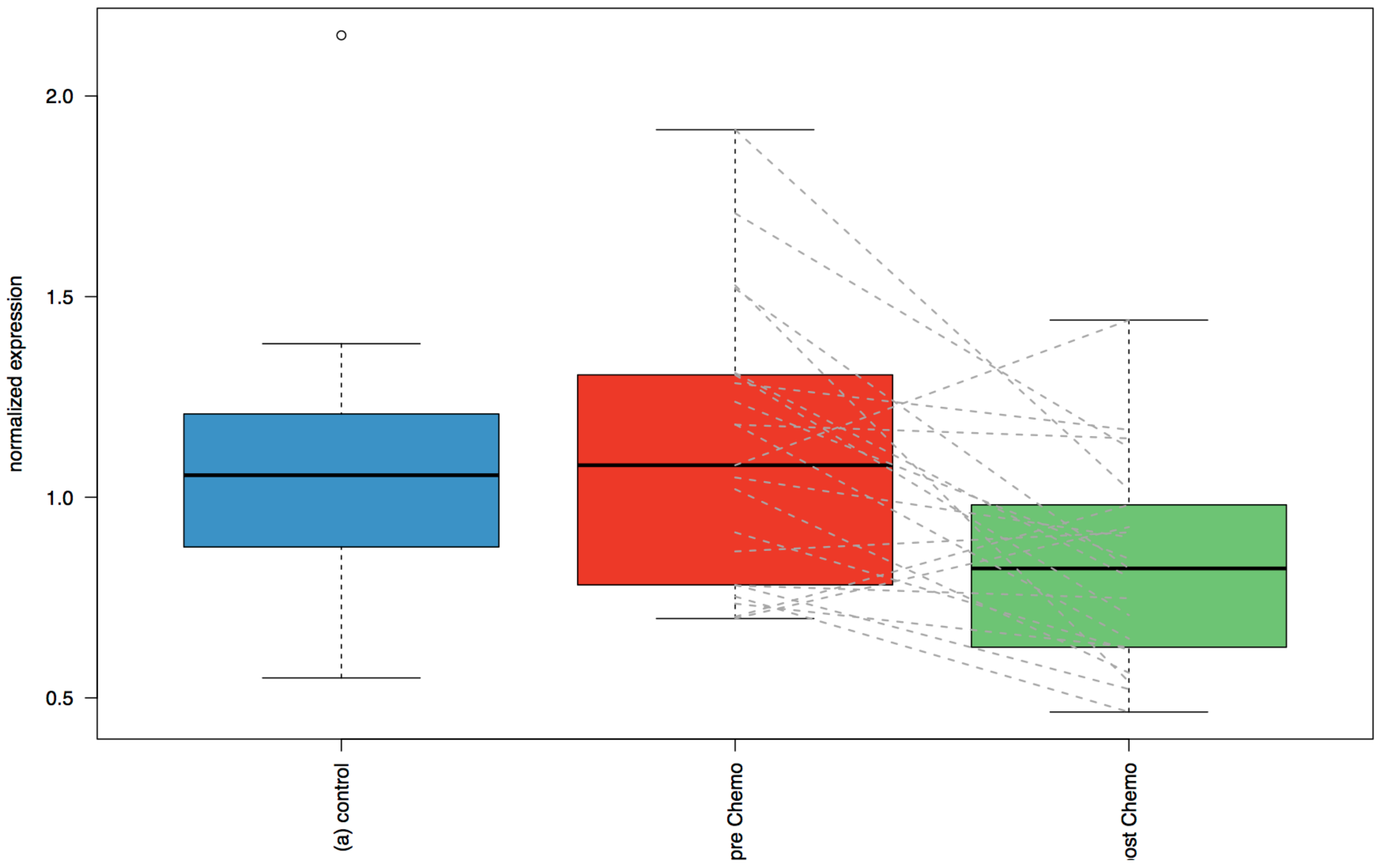
normalized expression
2.0
1.5
1.0
0.5
(a) control
pre Chemo
ost Chemo

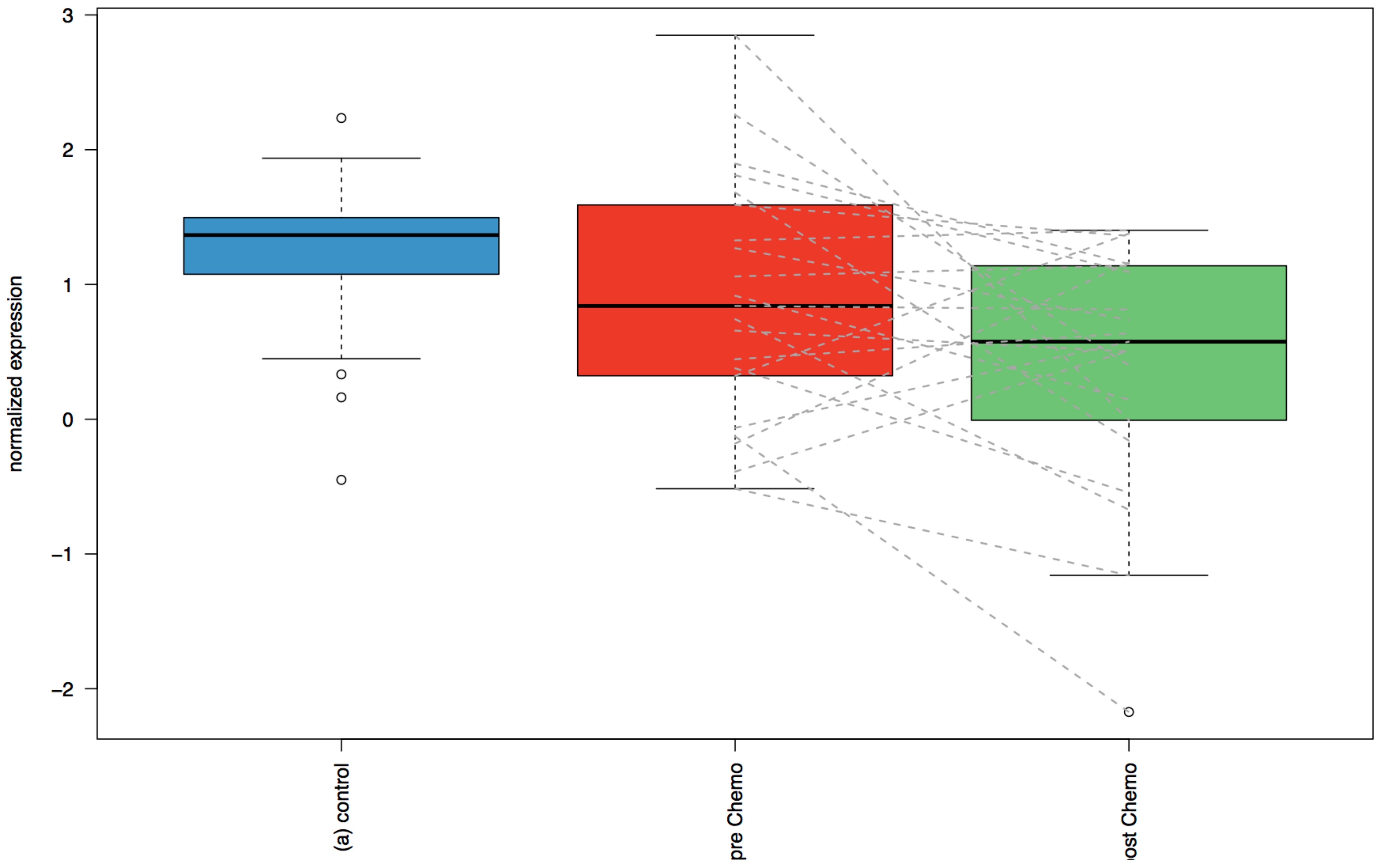
normalized expression
3
2
1
0
−1
−2
(a) control
pre Chemo
ost Chemo

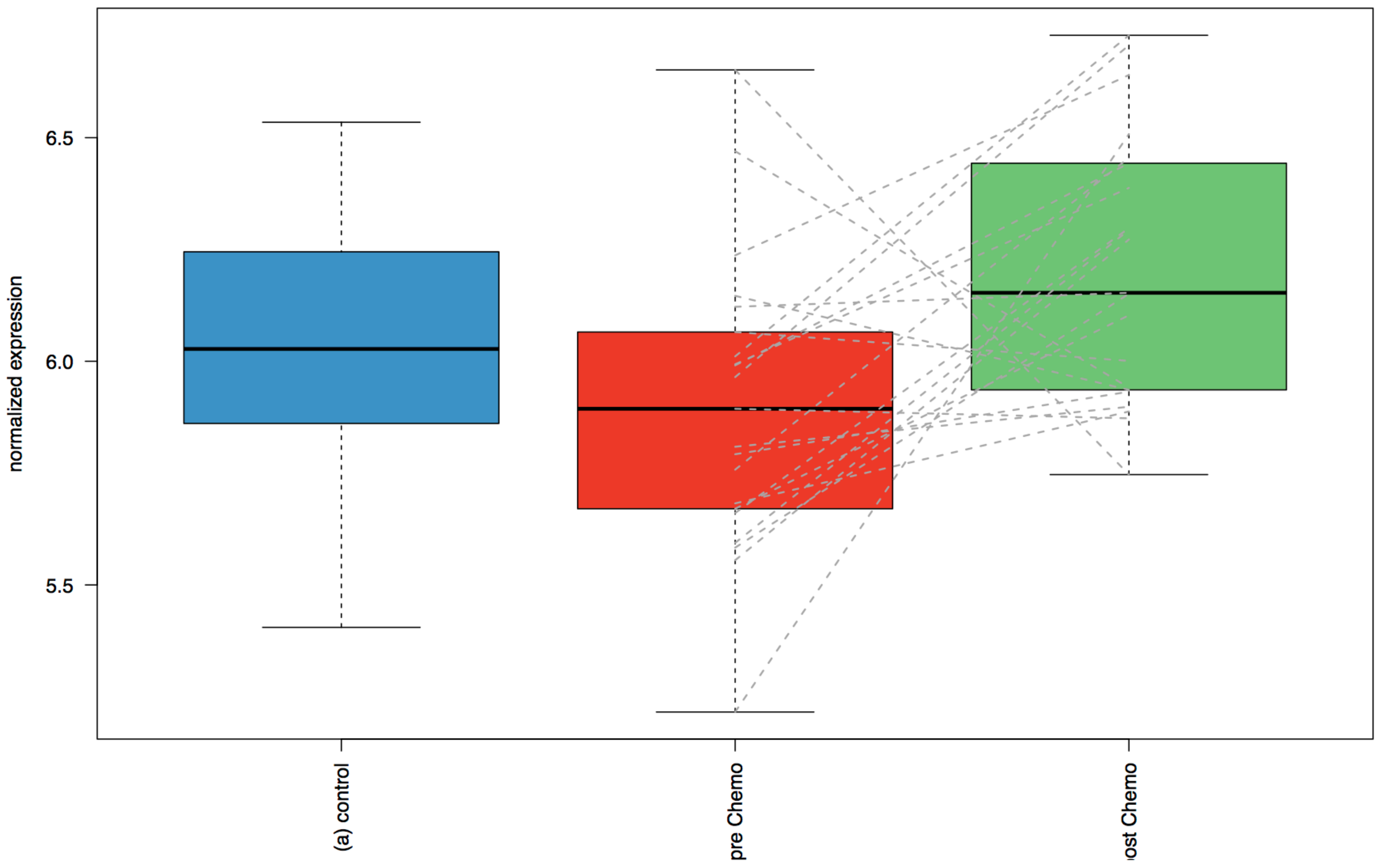

normalized expression
6.5
6.0
5.5
(a) control
pre Chemo
ost Chemo

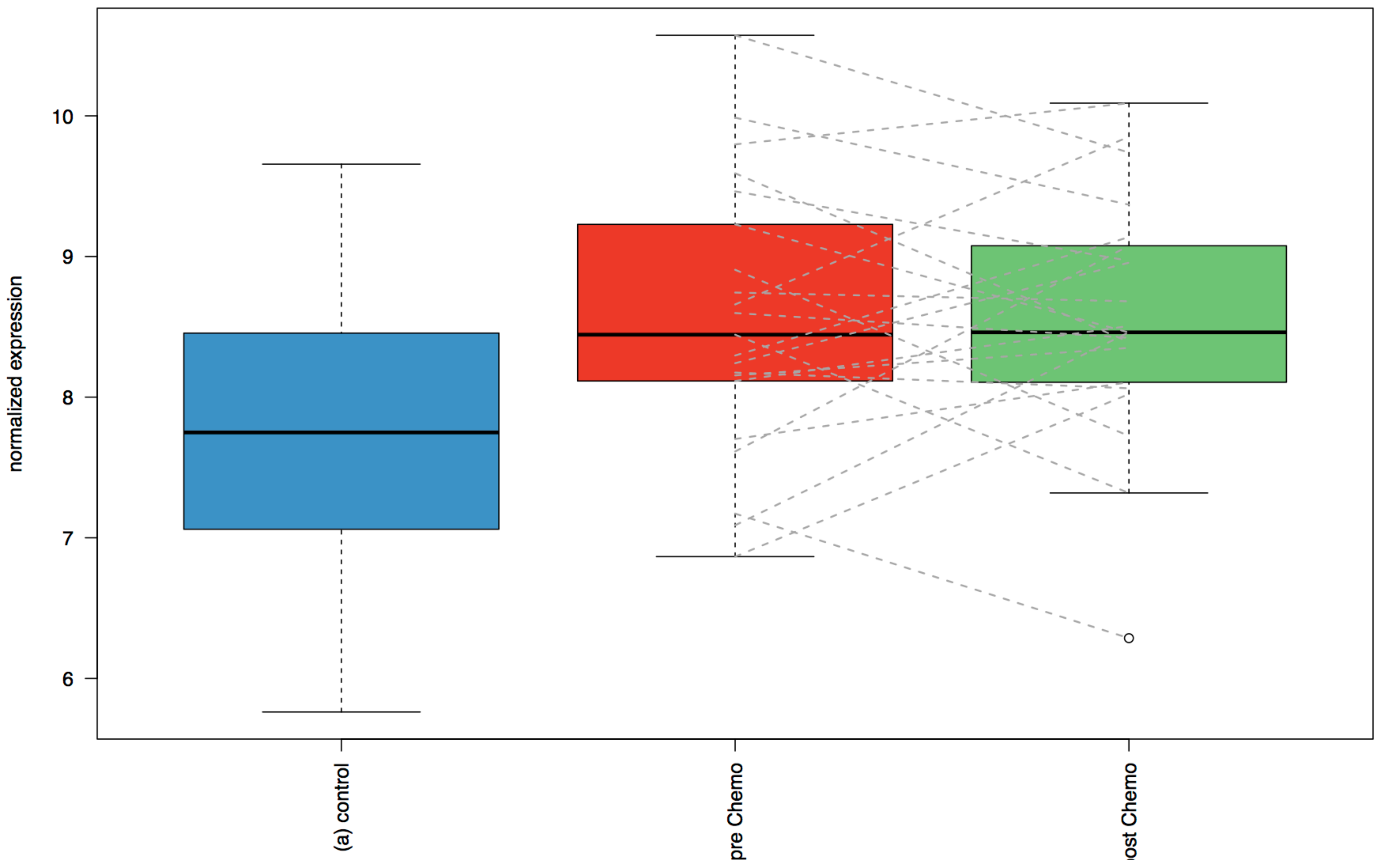
normalized expression
10
9
8
7
6
(a) control
pre Chemo
ost Chemo

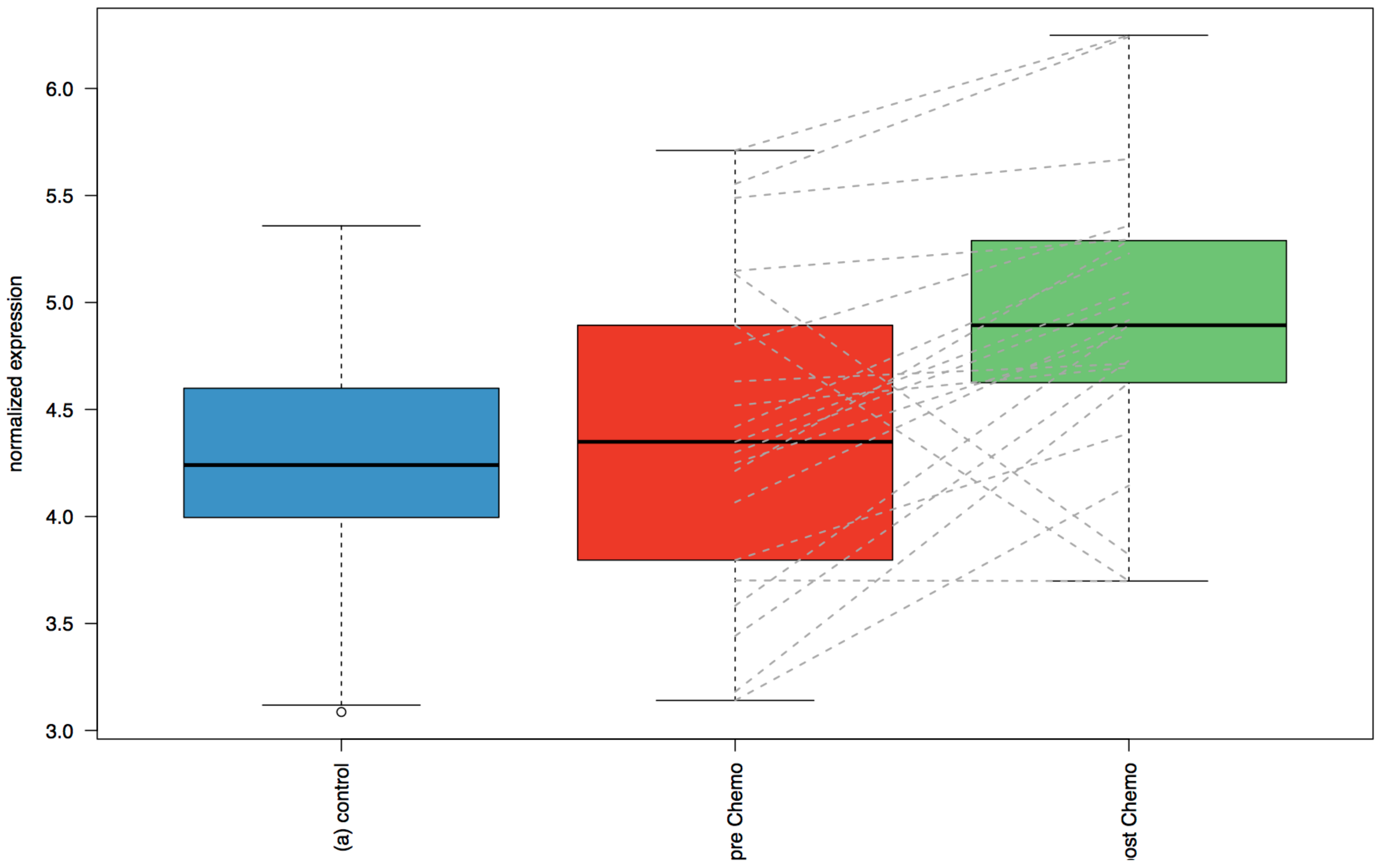
normalized expression
6.0
5.5
5.0
4.5
4.0
3.5
3.0
(a) control
pre Chemo
ost Chemo

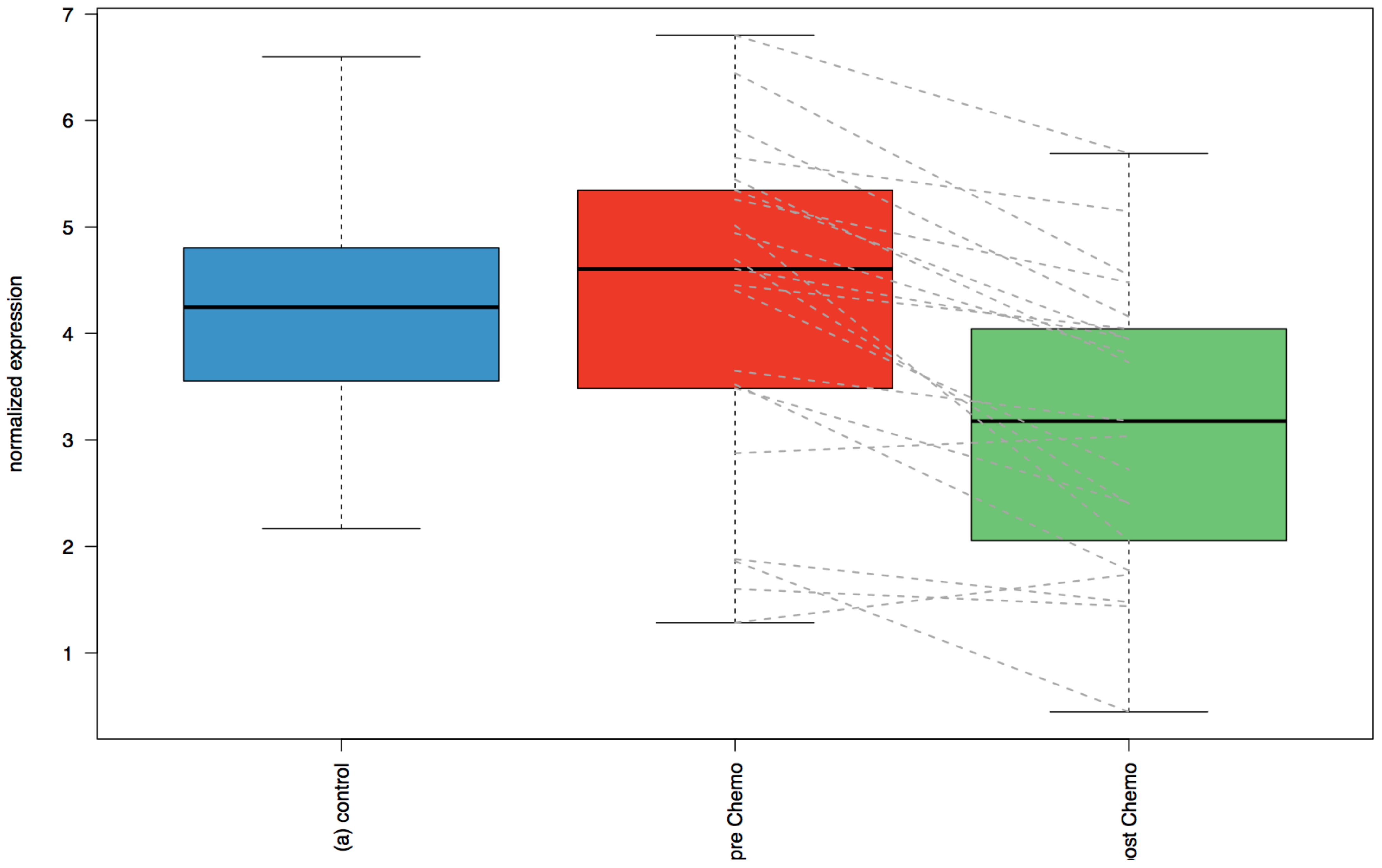
normalized expression
7
6
5
4
3
2
1
(a) control
pre Chemo
ost Chemo

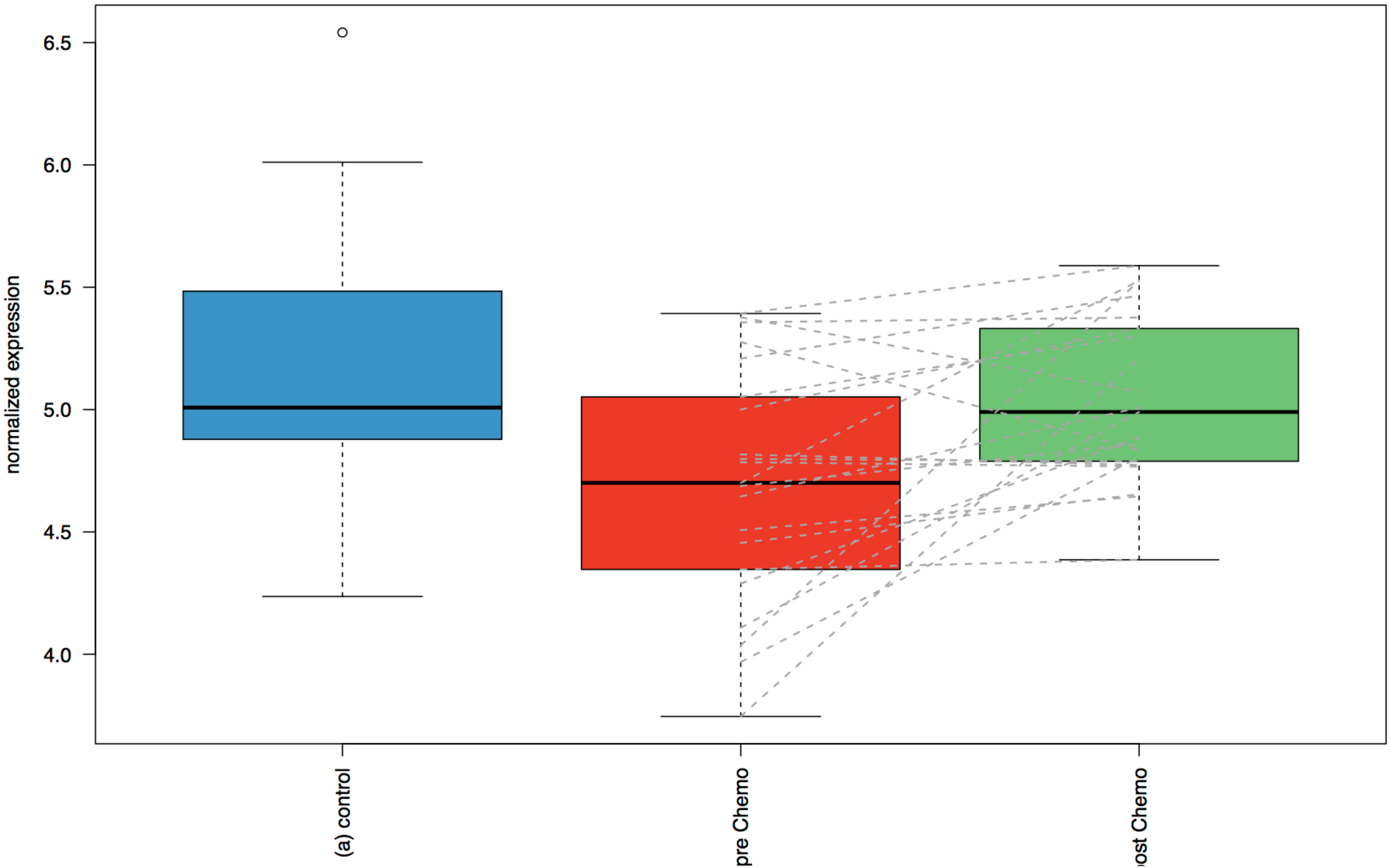
normalized expression
6.5
6.0
5.5
5.0
4.5
4.0
(a) control
pre Chemo
ost Chemo

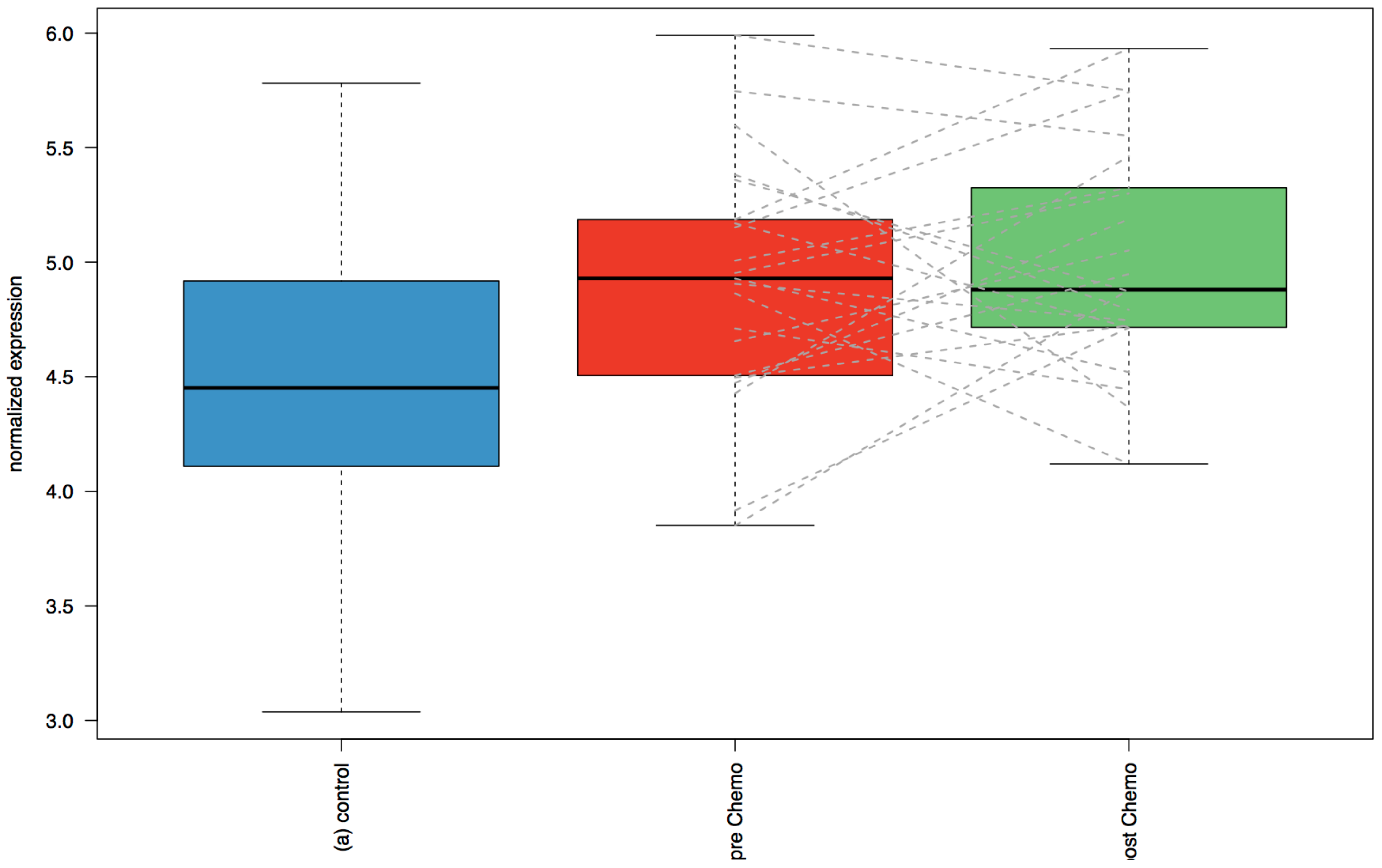
normalized expression
6.0
5.5
5.0
4.5
4.0
3.5
3.0
(a) control
pre Chemo
ost Chemo

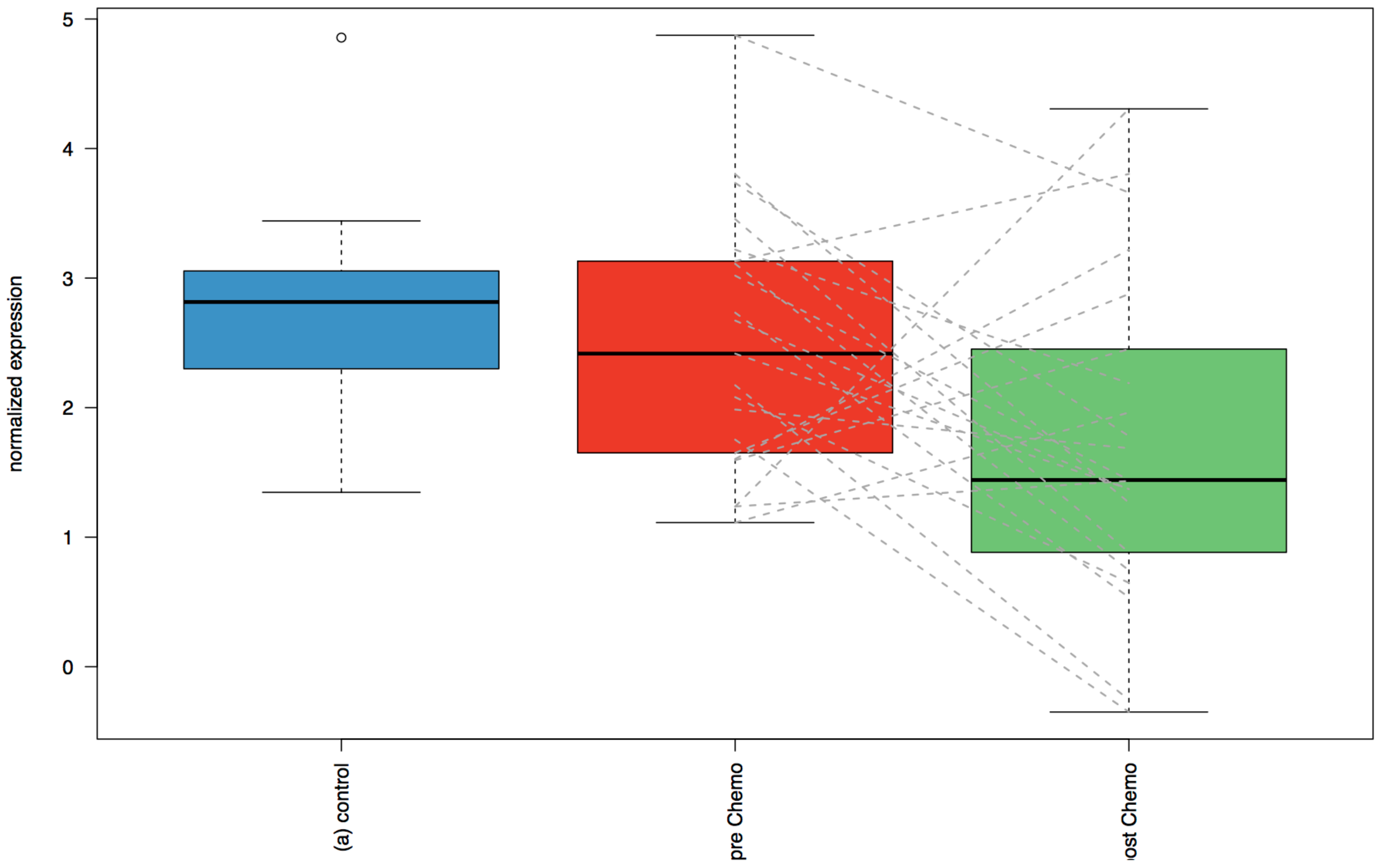
normalized expression
0
1
2
3
4
5
(a) control
pre Chemo
ost Chemo

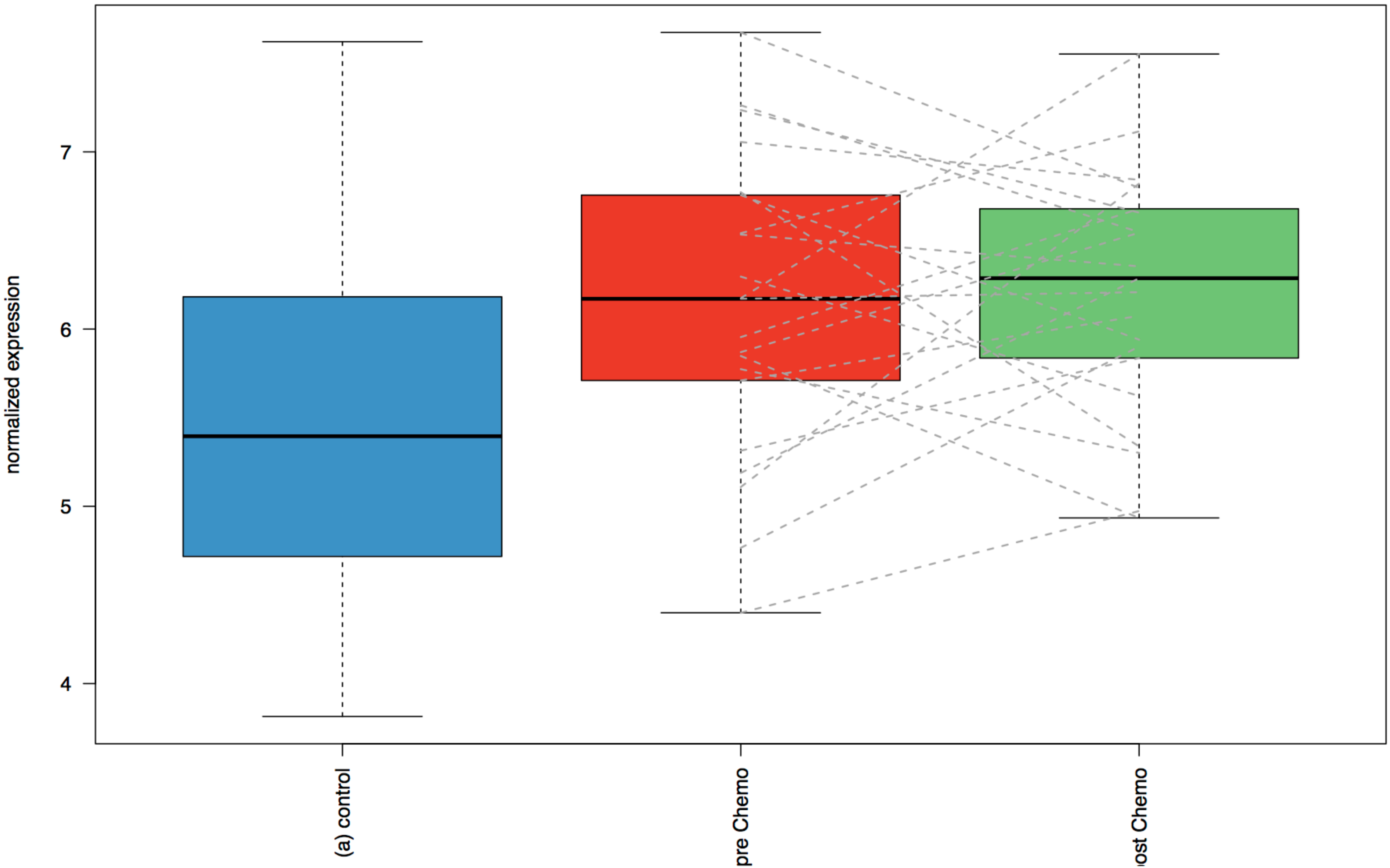
normalized expression
7
6
5
4
(a) control
pre Chemo
ost Chemo

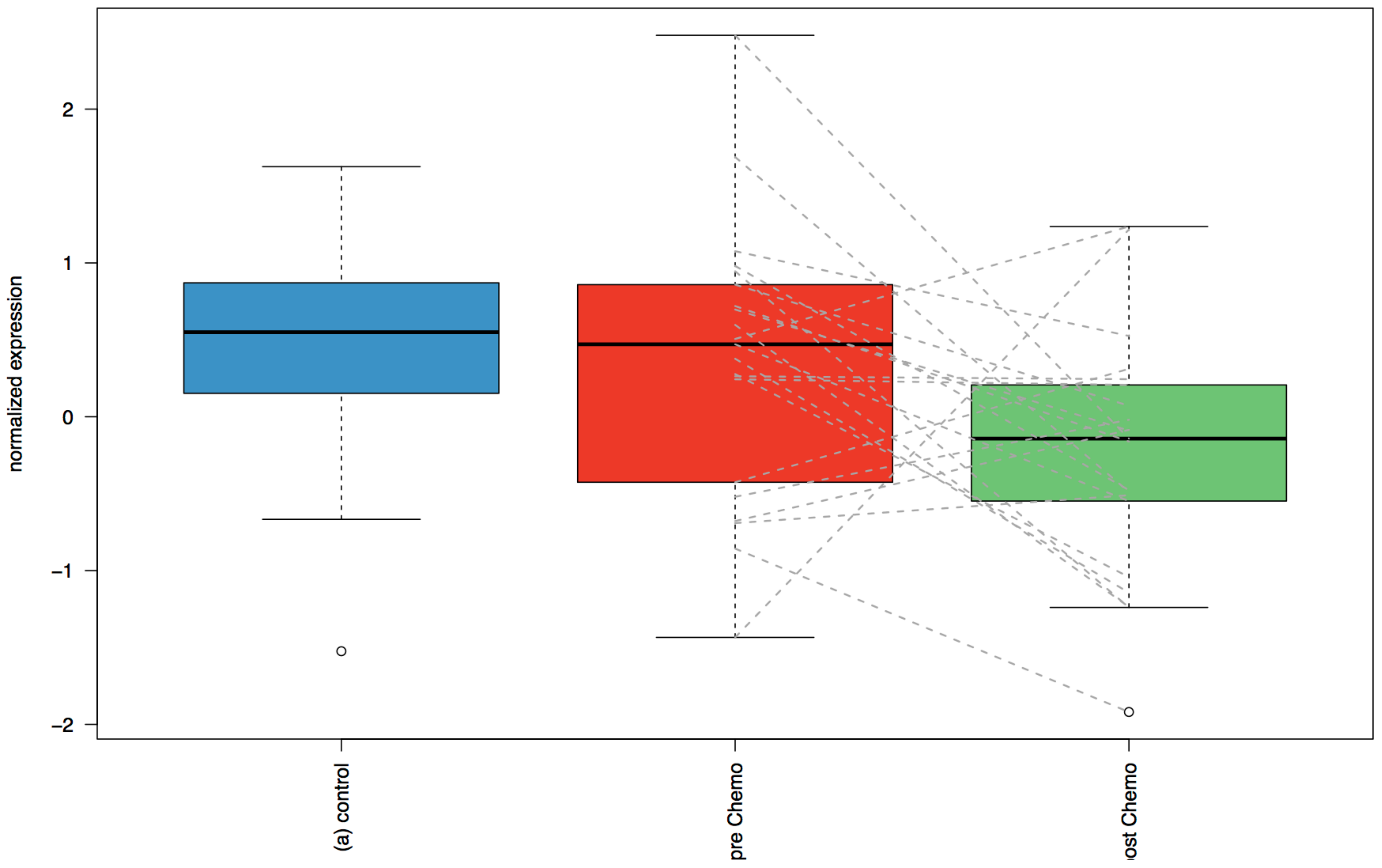
normalized expression
2
1
0
−1
−2
(a) control
pre Chemo
ost Chemo

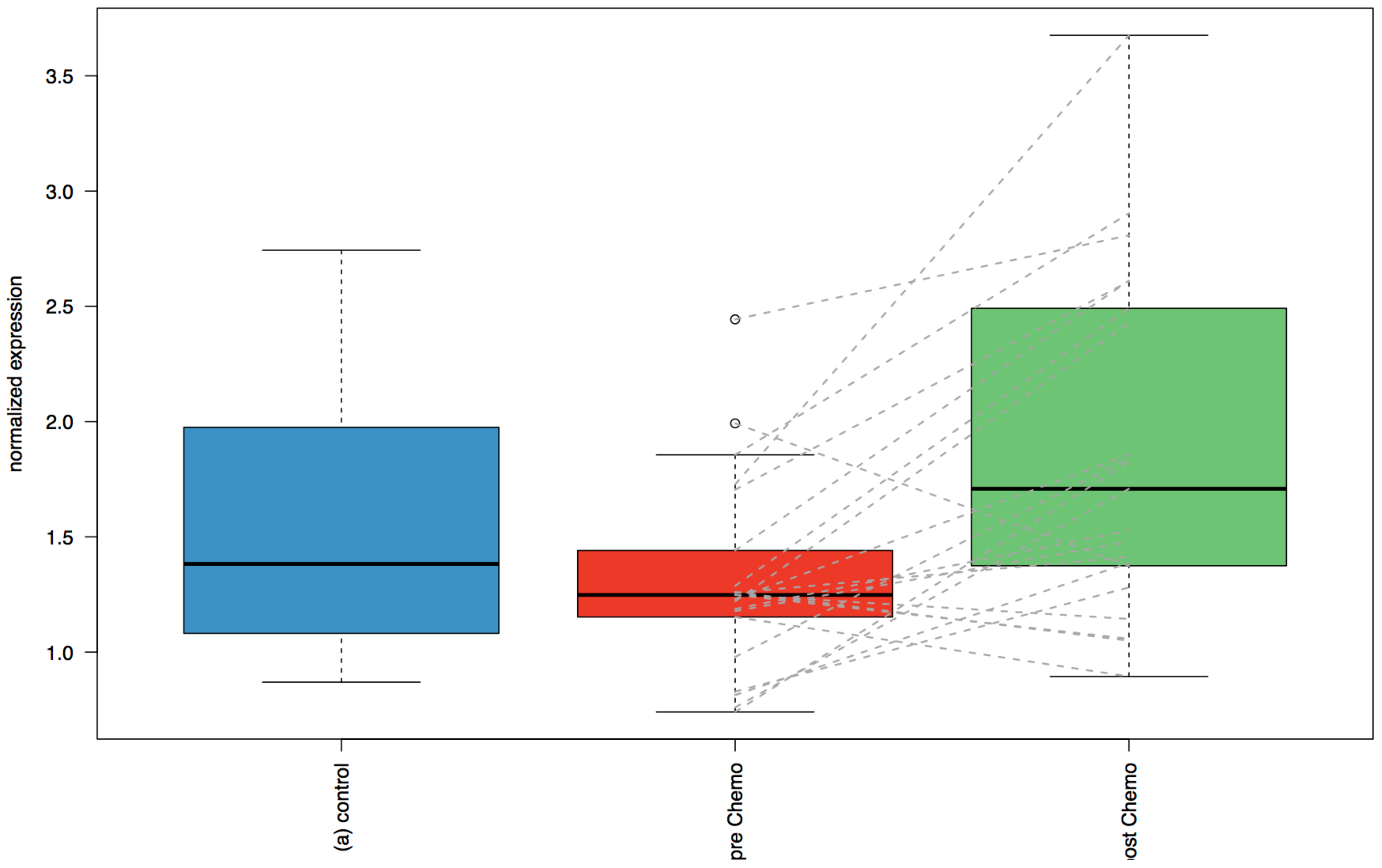
normalized expression
3.5
3.0
2.5
2.0
1.5
1.0
(a) control
pre Chemo
ost Chemo

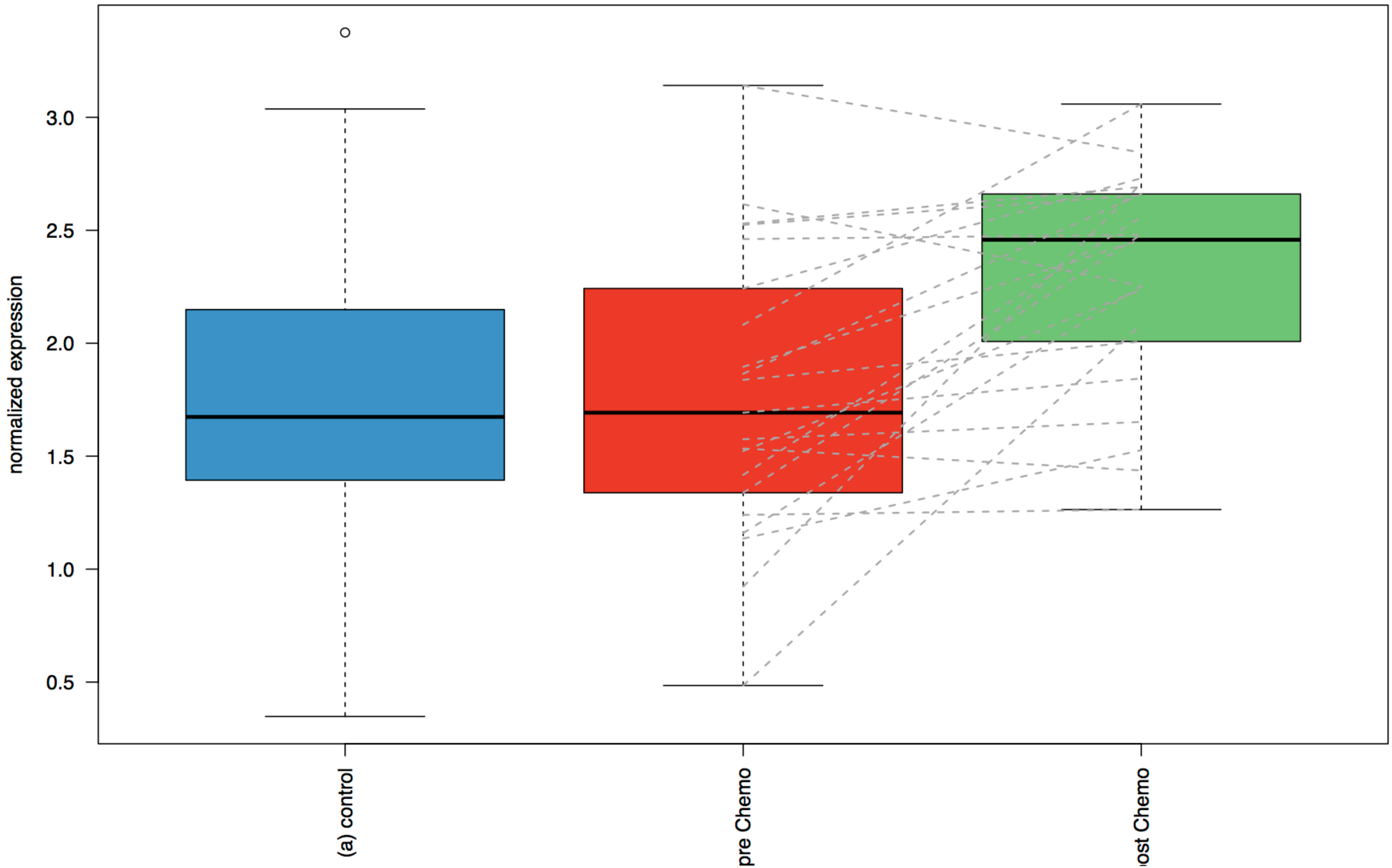

normalized expression
3.0
2.5
2.0
1.5
1.0
0.5
(a) control
pre Chemo
ost Chemo

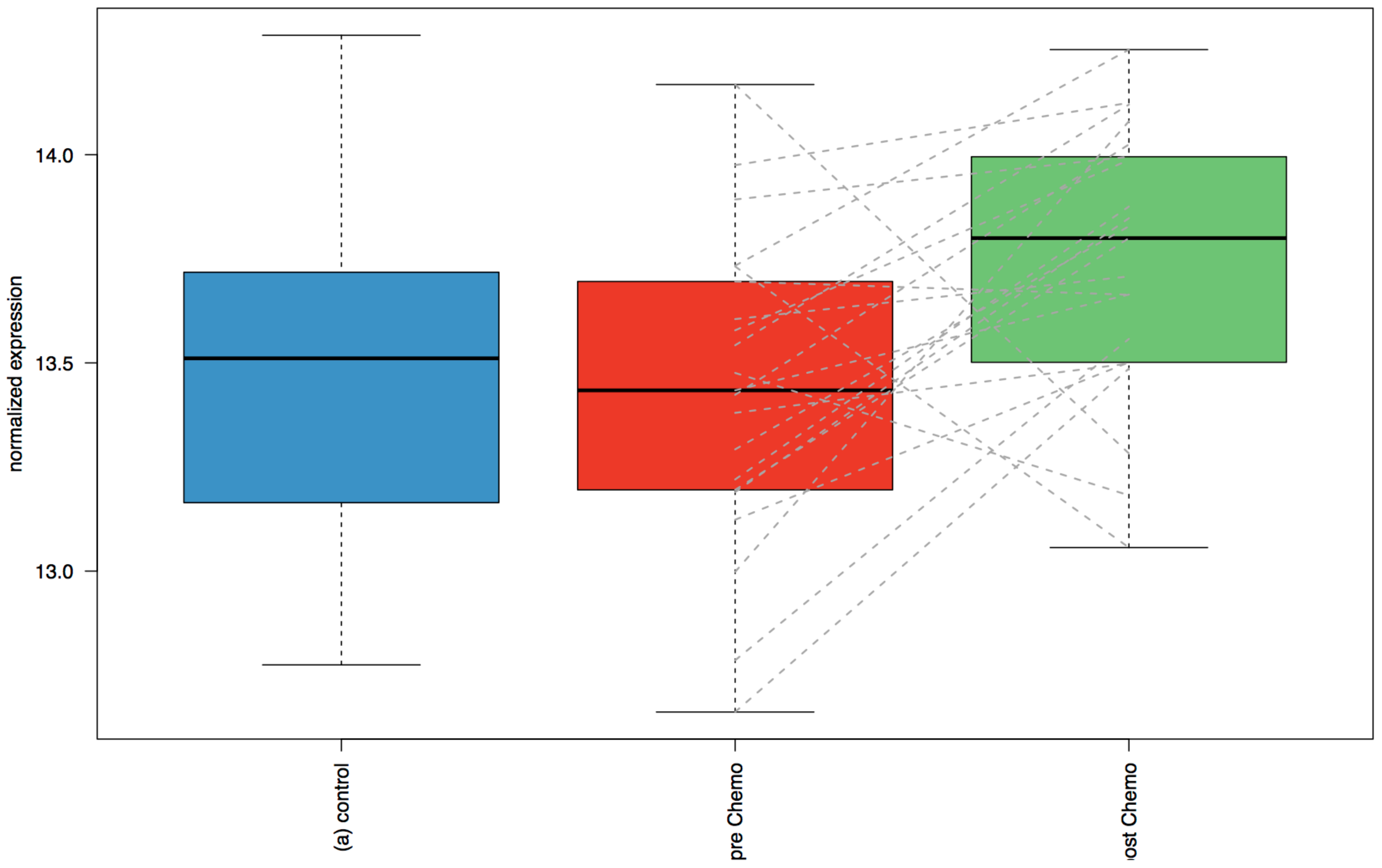
normalized expression
14.0
13.5
13.0
(a) control
pre Chemo
ost Chemo

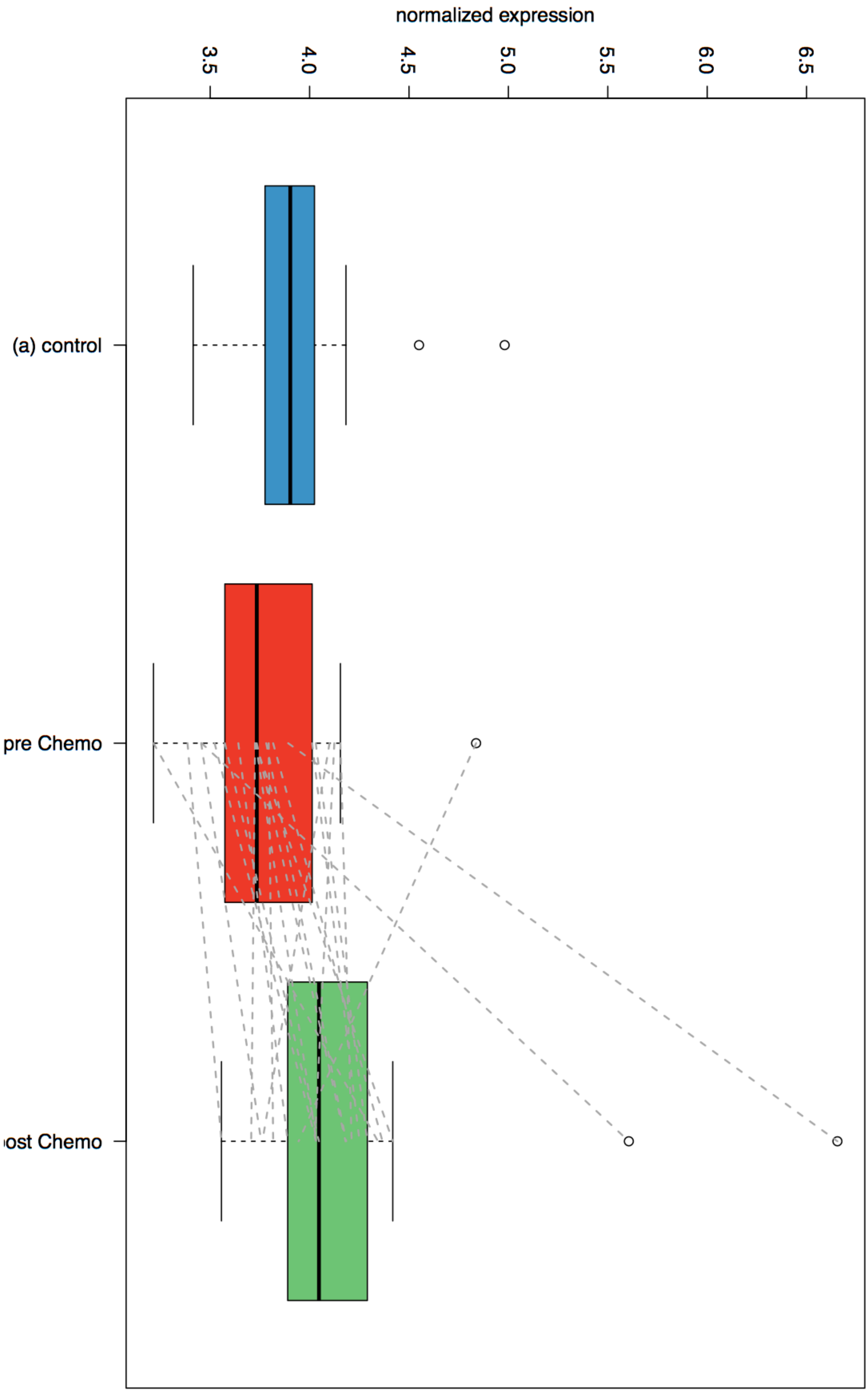
normalized expression
3.5
4.0
4.5
5.0
5.5
6.0
6.5
(a) control
pre Chemo
ost Chemo

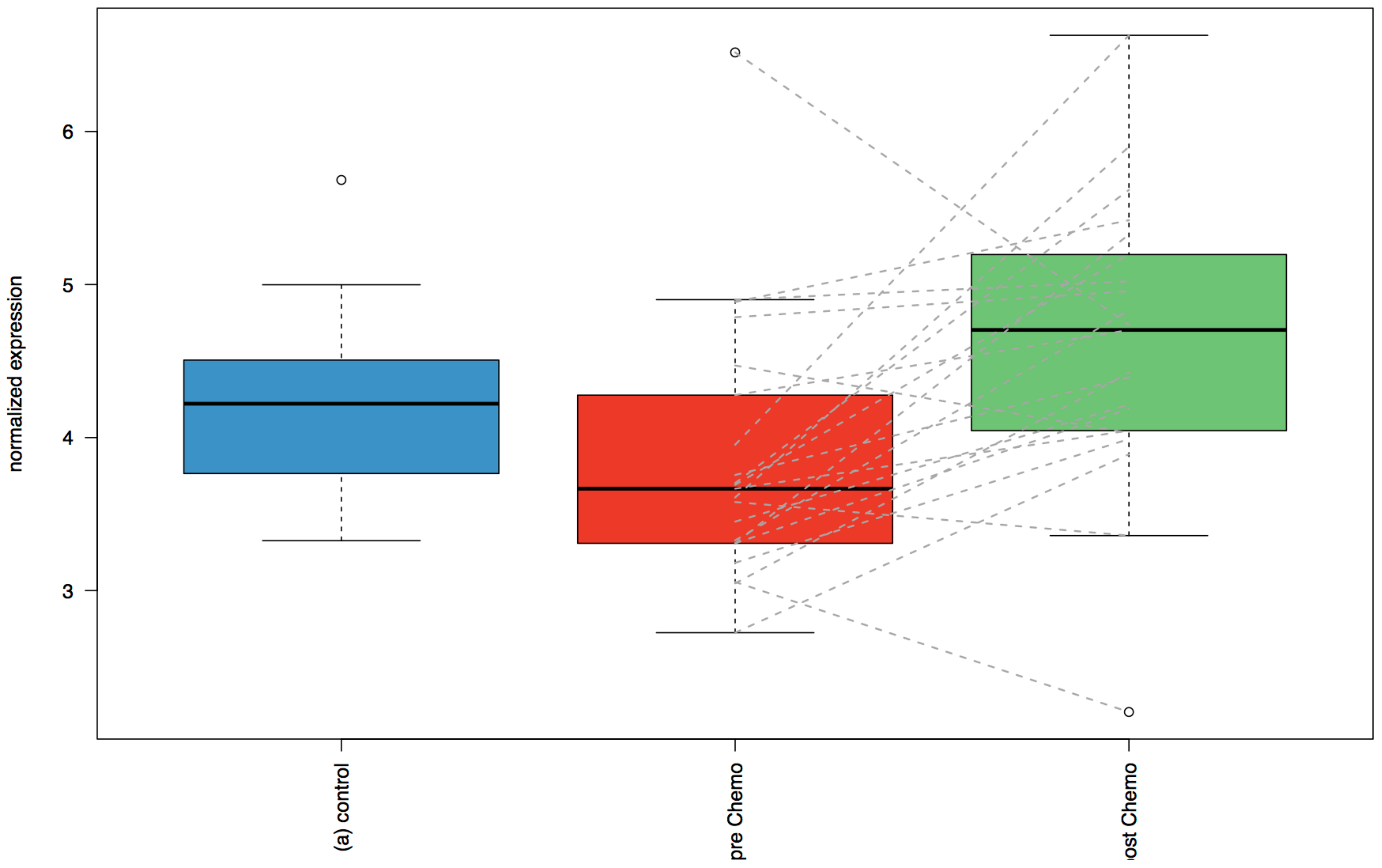
normalized expression
6
5
4
3
(a) control
pre Chemo
ost Chemo

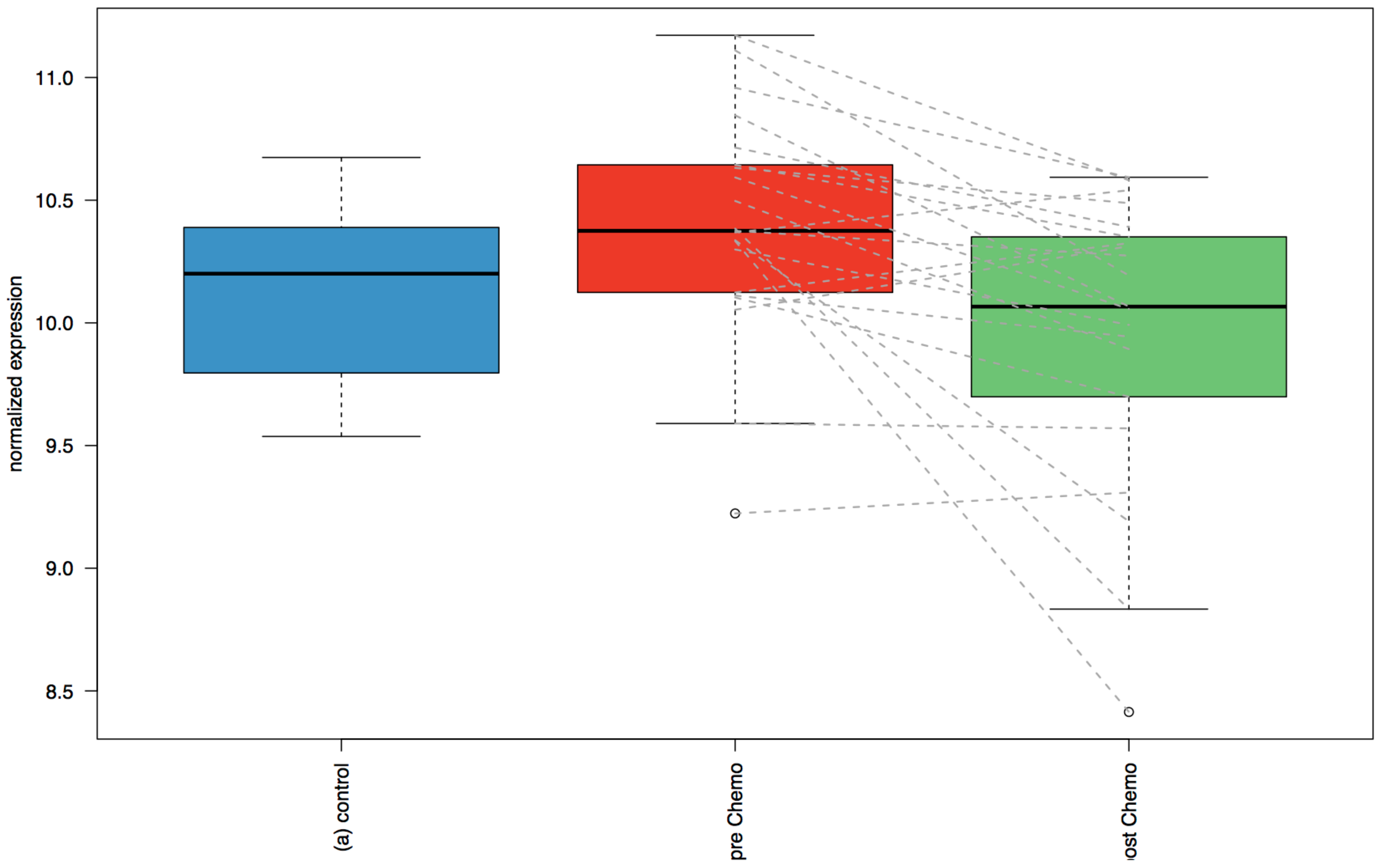
normalized expression
11.0
10.5
10.0
9.5
9.0
8.5
(a) control
pre Chemo
ost Chemo

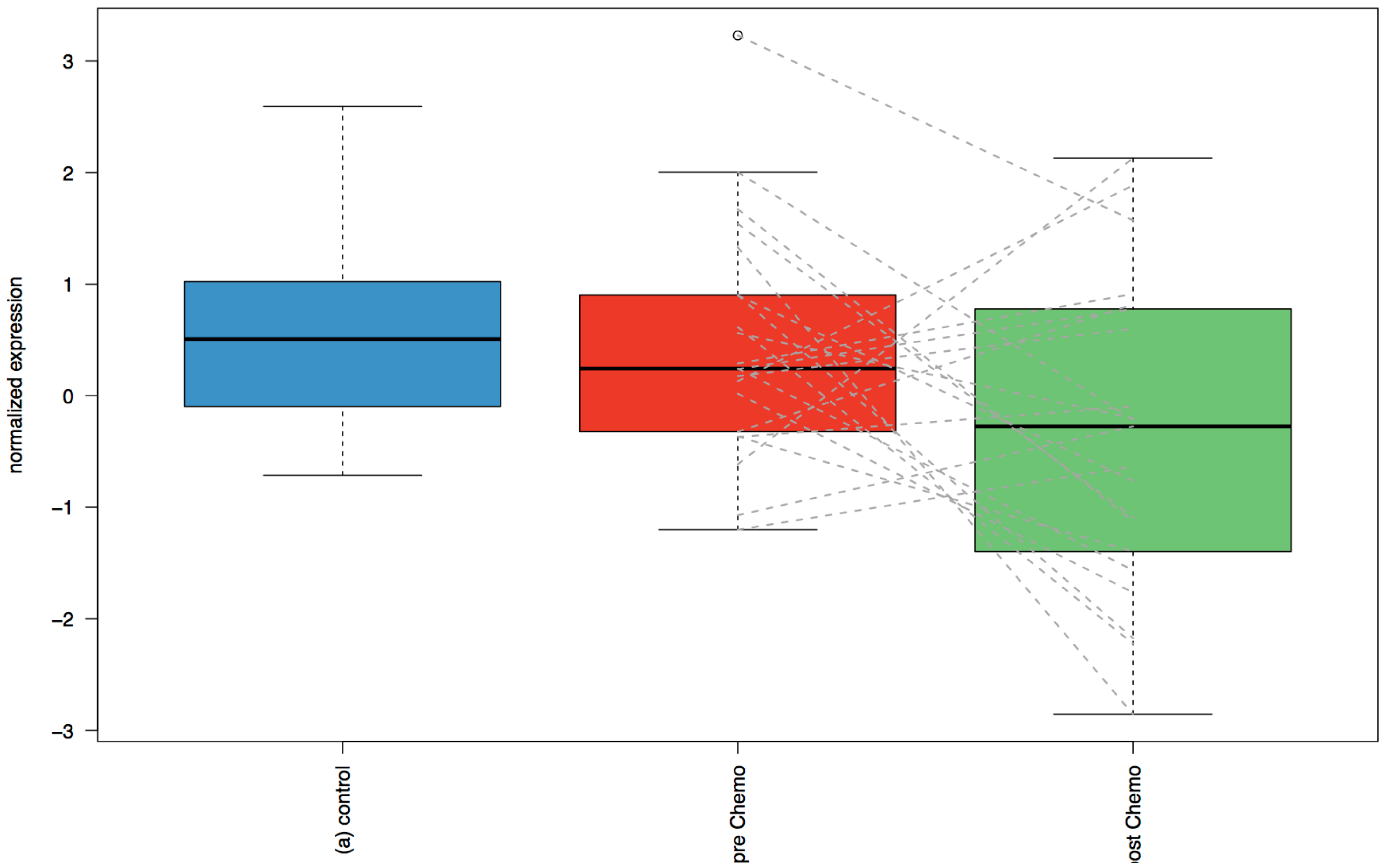
normalized expression
3
2
1
0
−1
−2
−3
(a) control
pre Chemo
ost Chemo

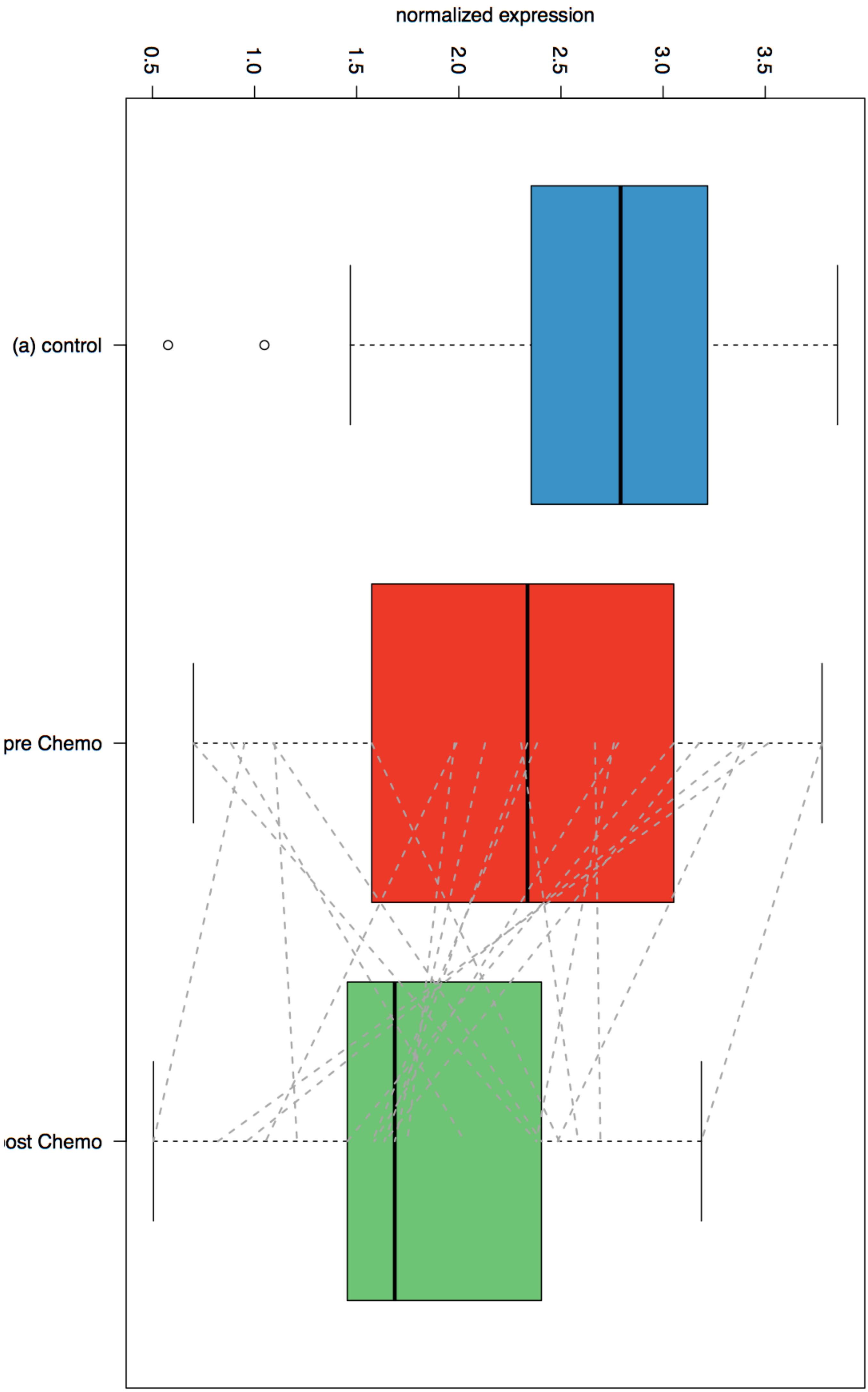

normalized expression
0.5
1.0
1.5
2.0
2.5
3.0
3.5
(a) control
pre Chemo
ost Chemo

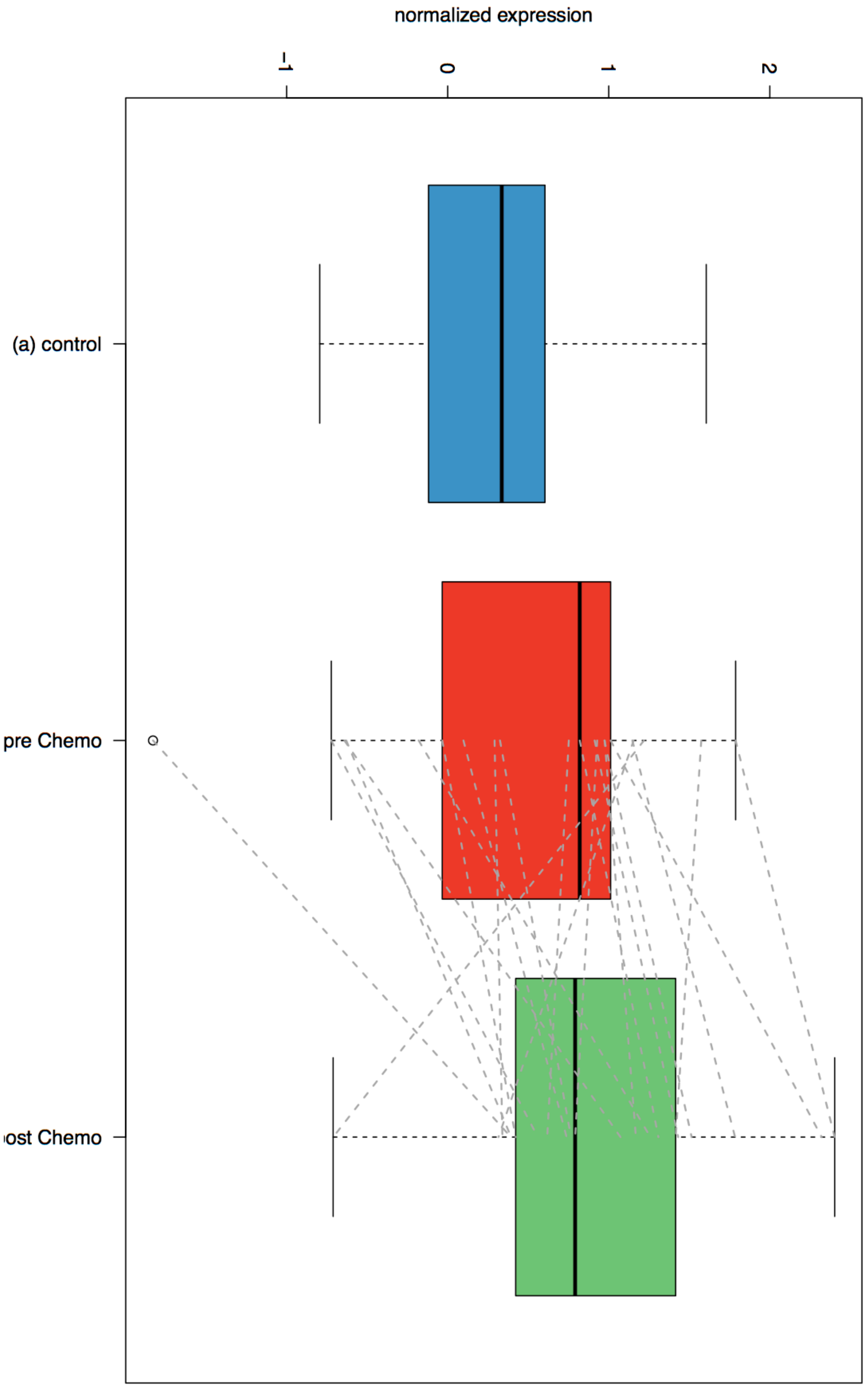
normalized expression
-1
0
1
2
(a) control
pre Chemo
ost Chemo

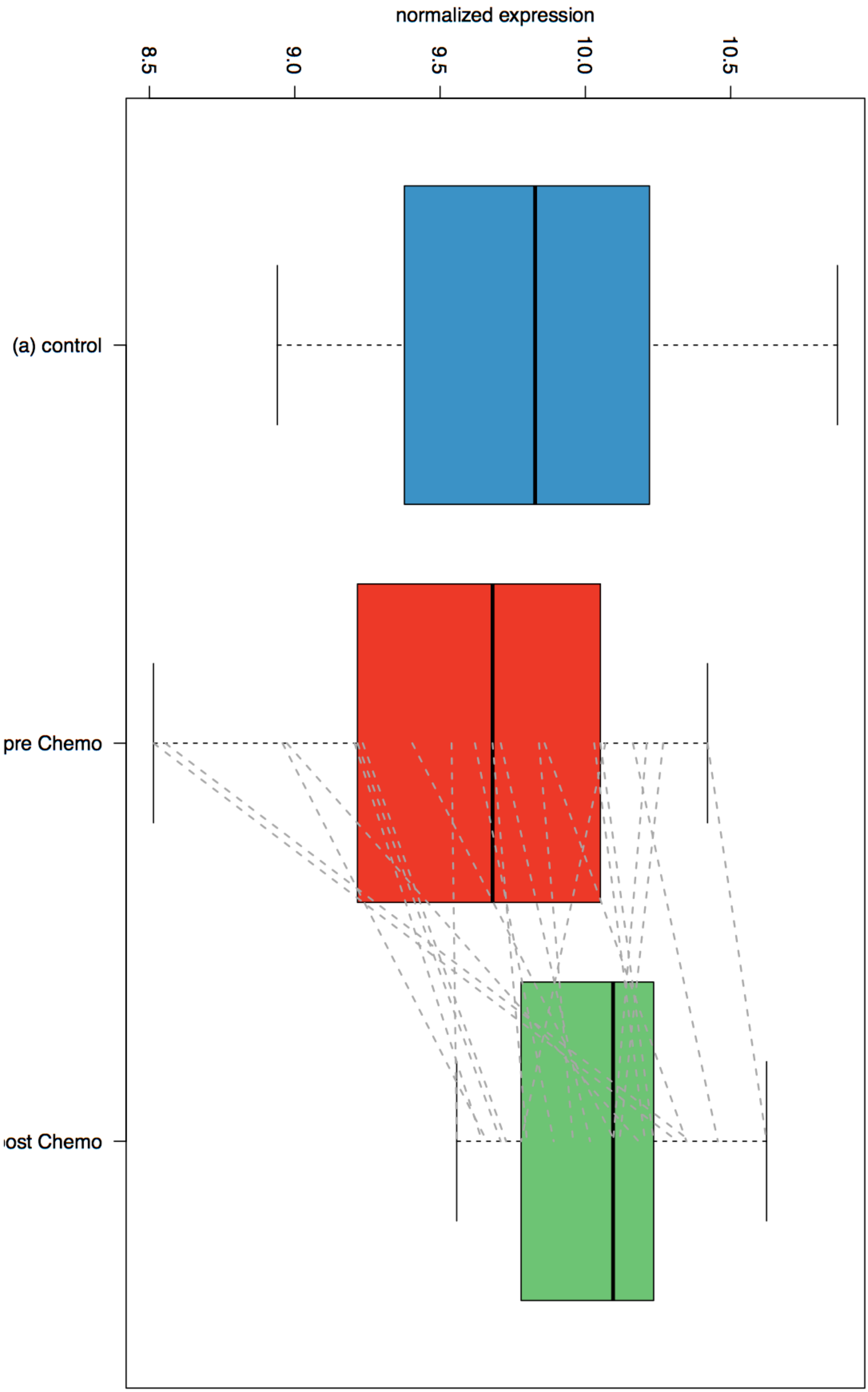
normalized expression
8.5
9.0
9.5
10.0
10.5
(a) control
pre Chemo
ost Chemo

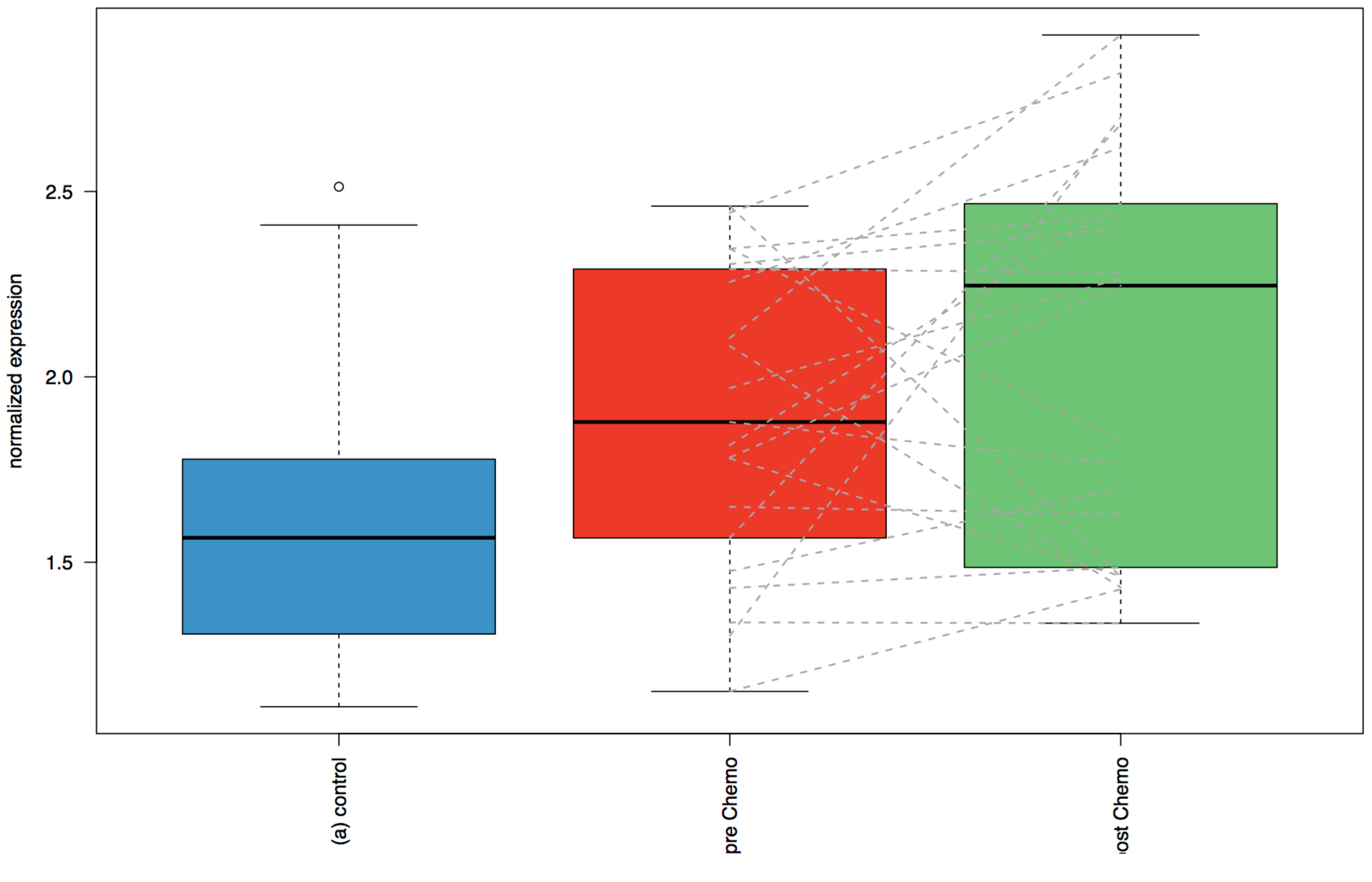

normalized expression
2.5
2.0
1.5
(a) control
pre Chemo
ost Chemo

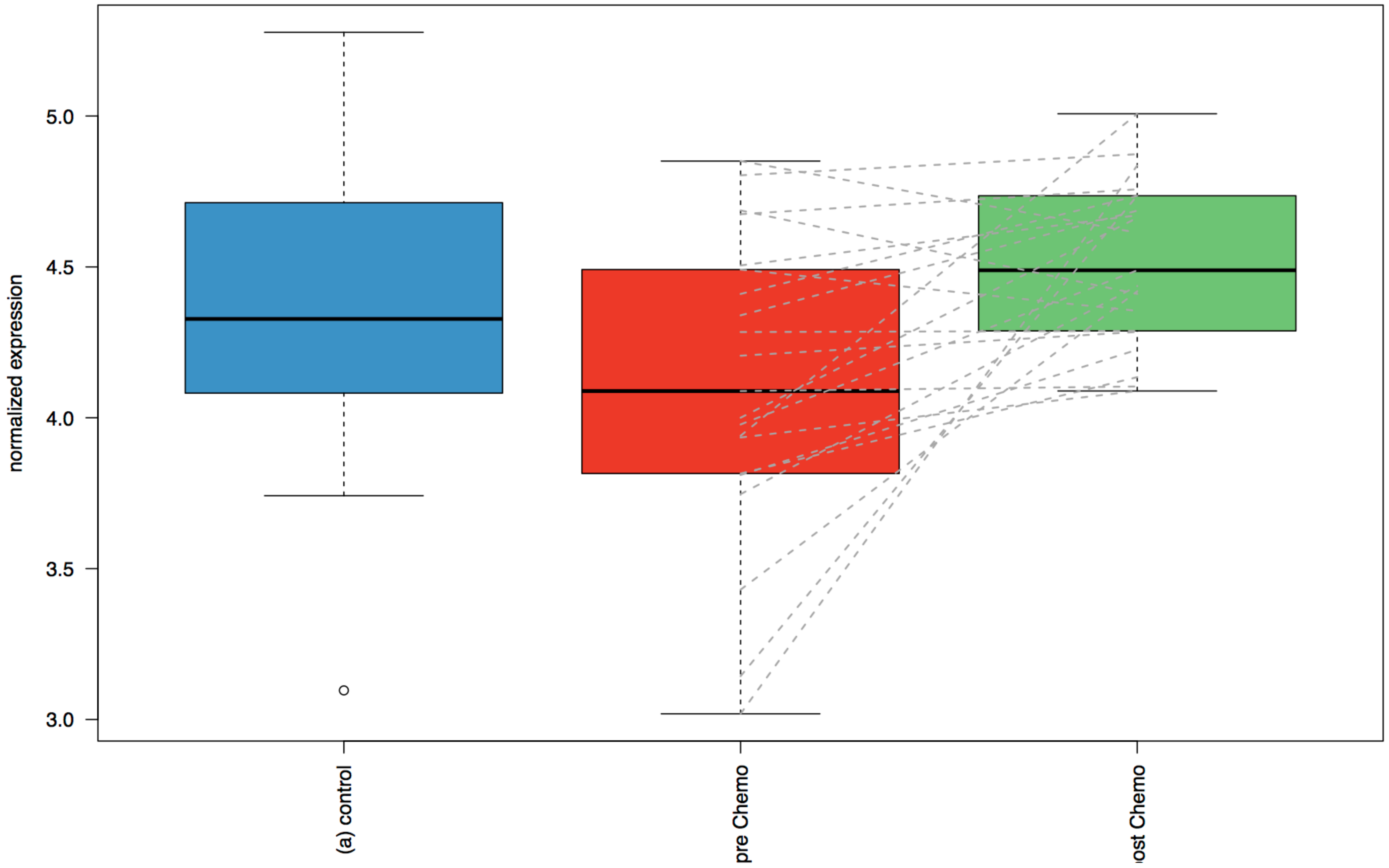
normalized expression
5.0
4.5
4.0
3.5
3.0
(a) control
pre Chemo
ost Chemo

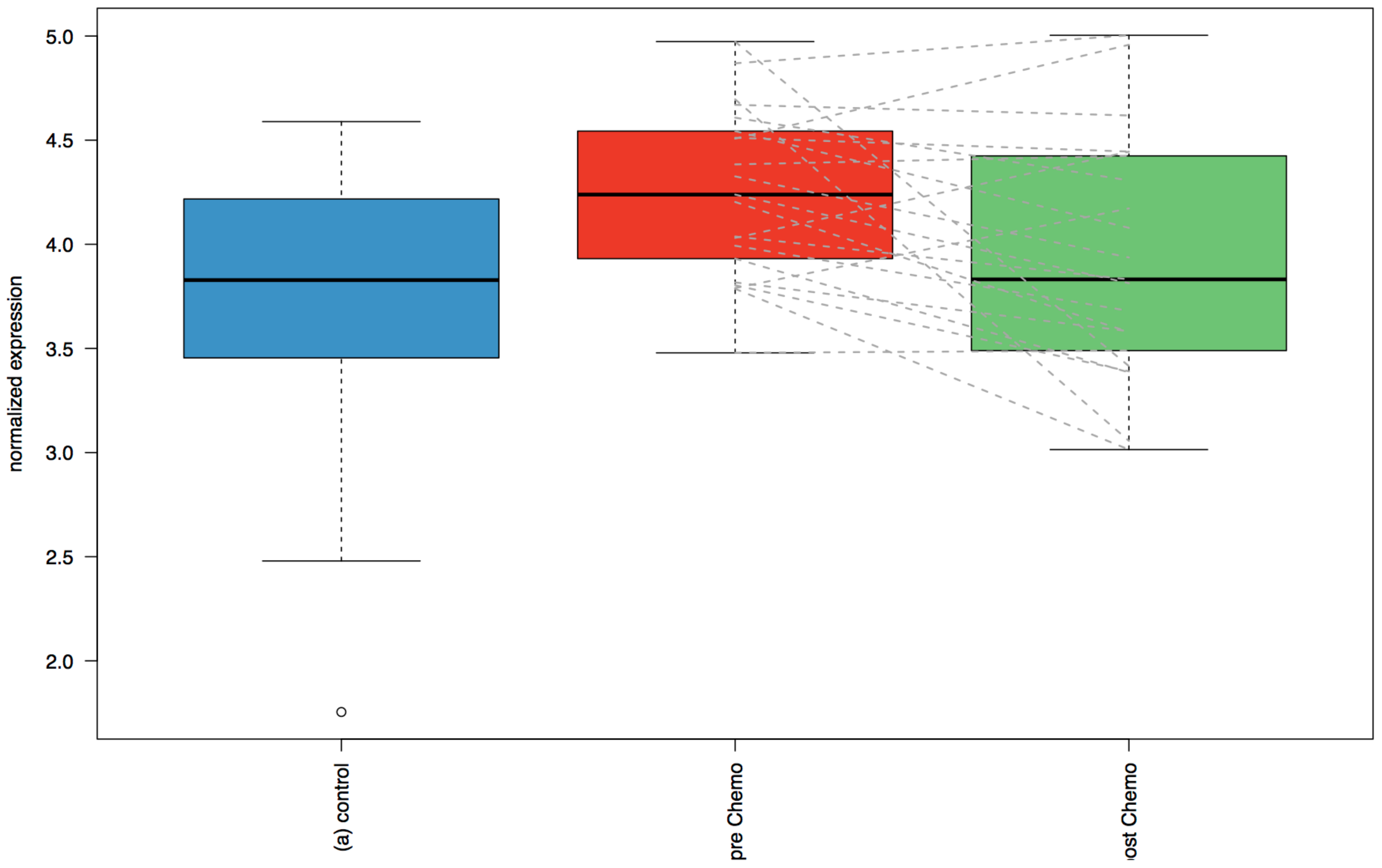
normalized expression
5.0
4.5
4.0
3.5
3.0
2.5
2.0
(a) control
pre Chemo
ost Chemo

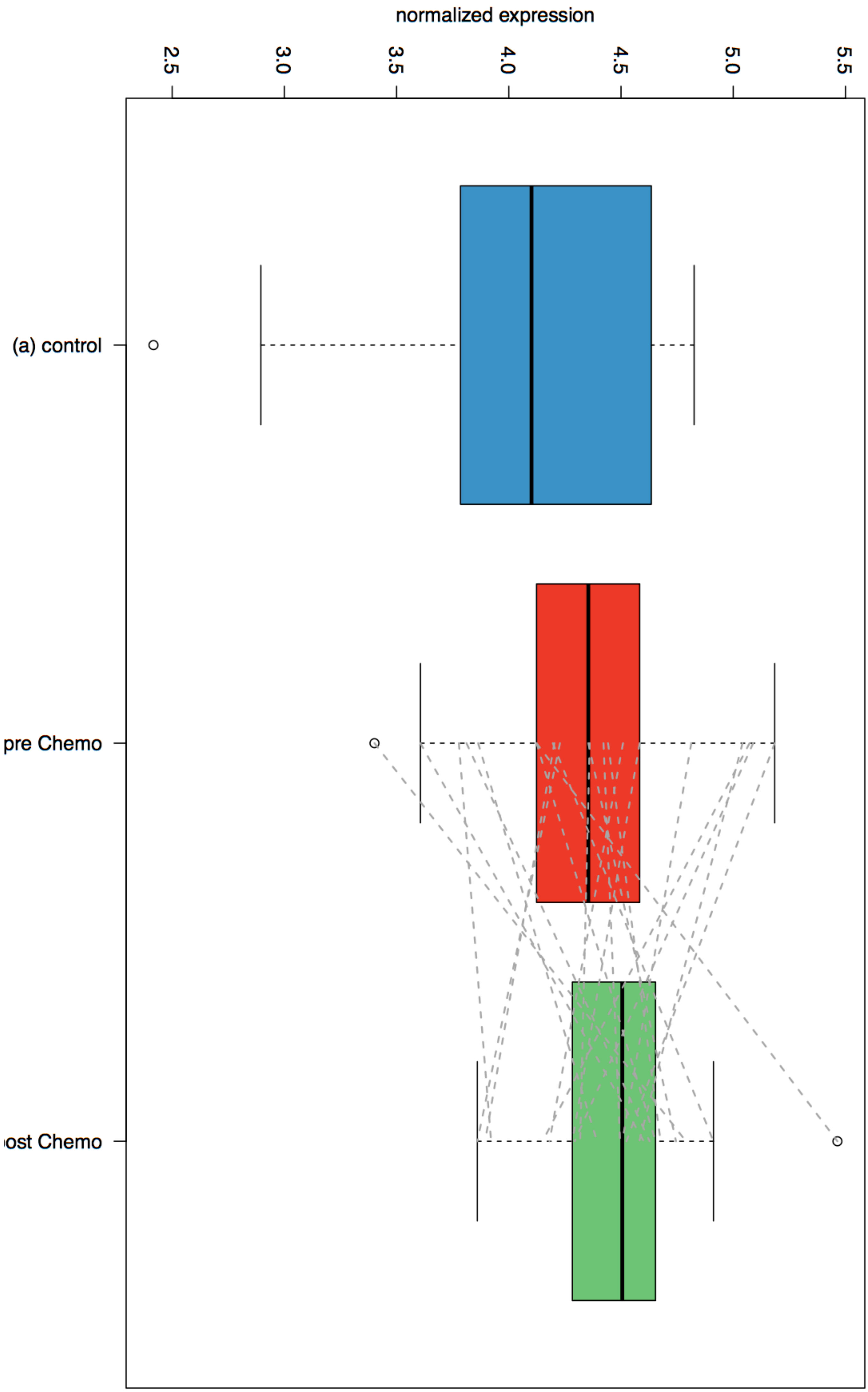
normalized expression
2.5
3.0
3.5
4.0
4.5
5.0
5.5
(a) control
pre Chemo
ost Chemo

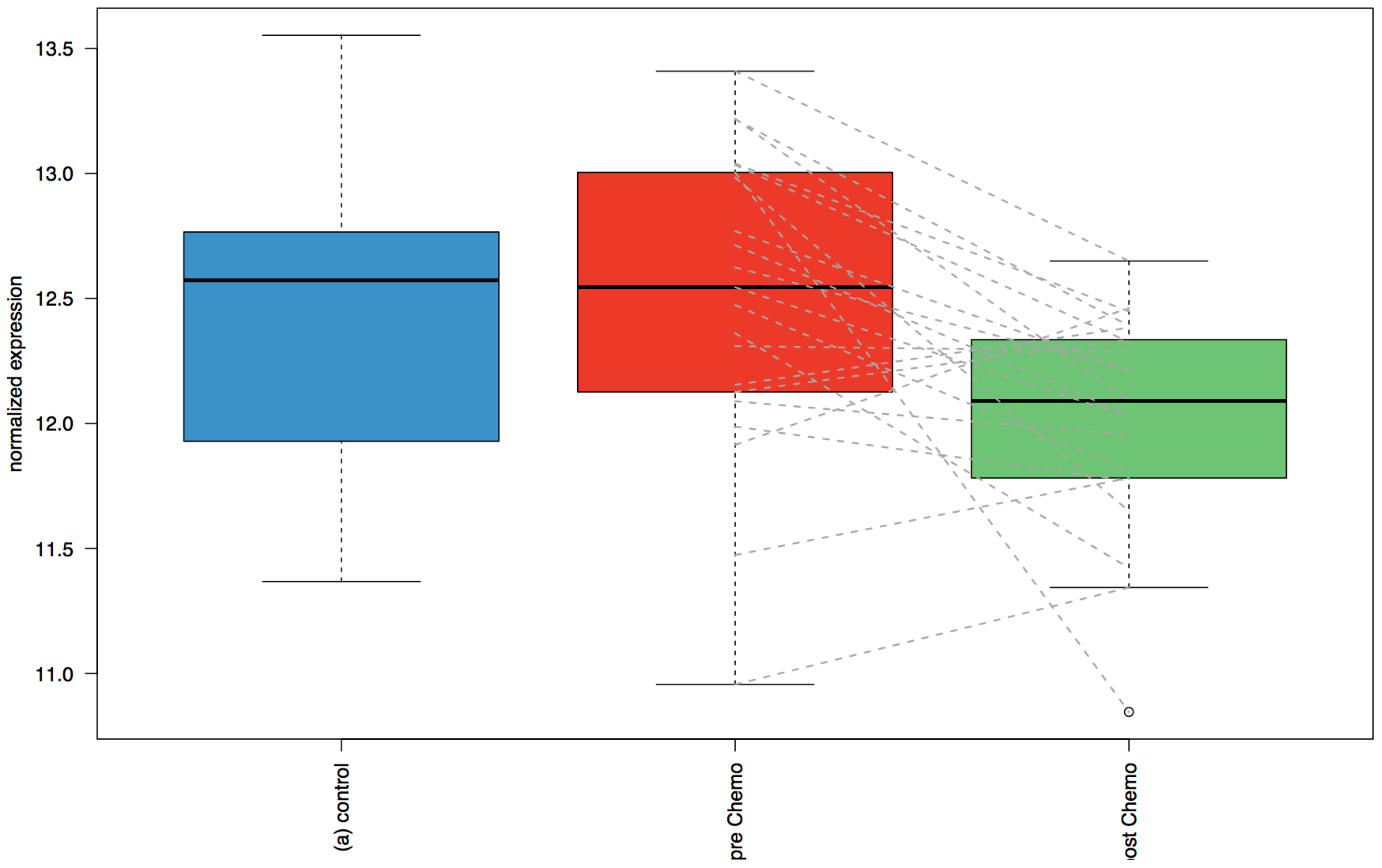

normalized expression
13.5
13.0
12.5
12.0
11.5
11.0
(a) control
pre Chemo
ost Chemo

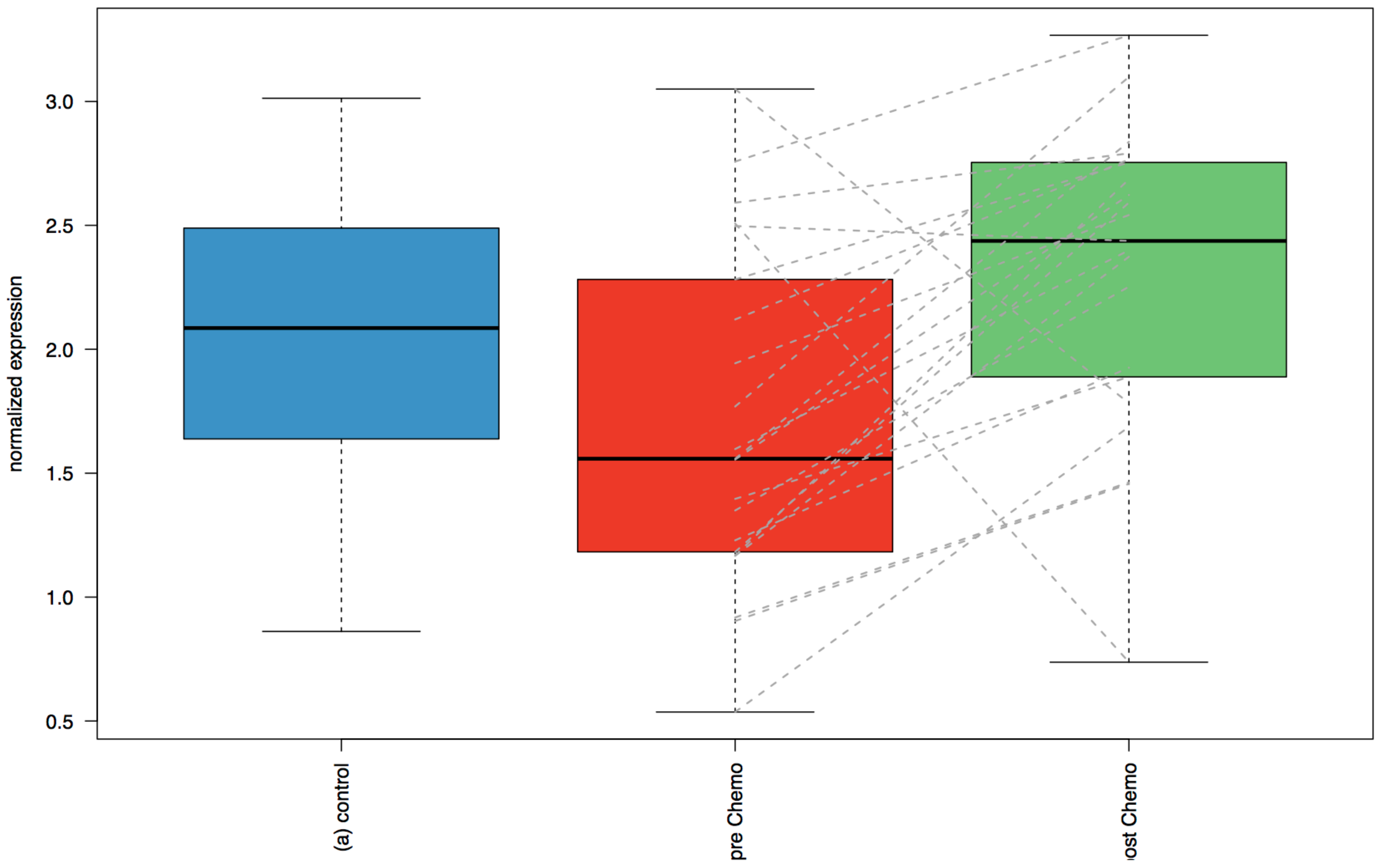
normalized expression
3.0
2.5
2.0
1.5
1.0
0.5
(a) control
pre Chemo
ost Chemo

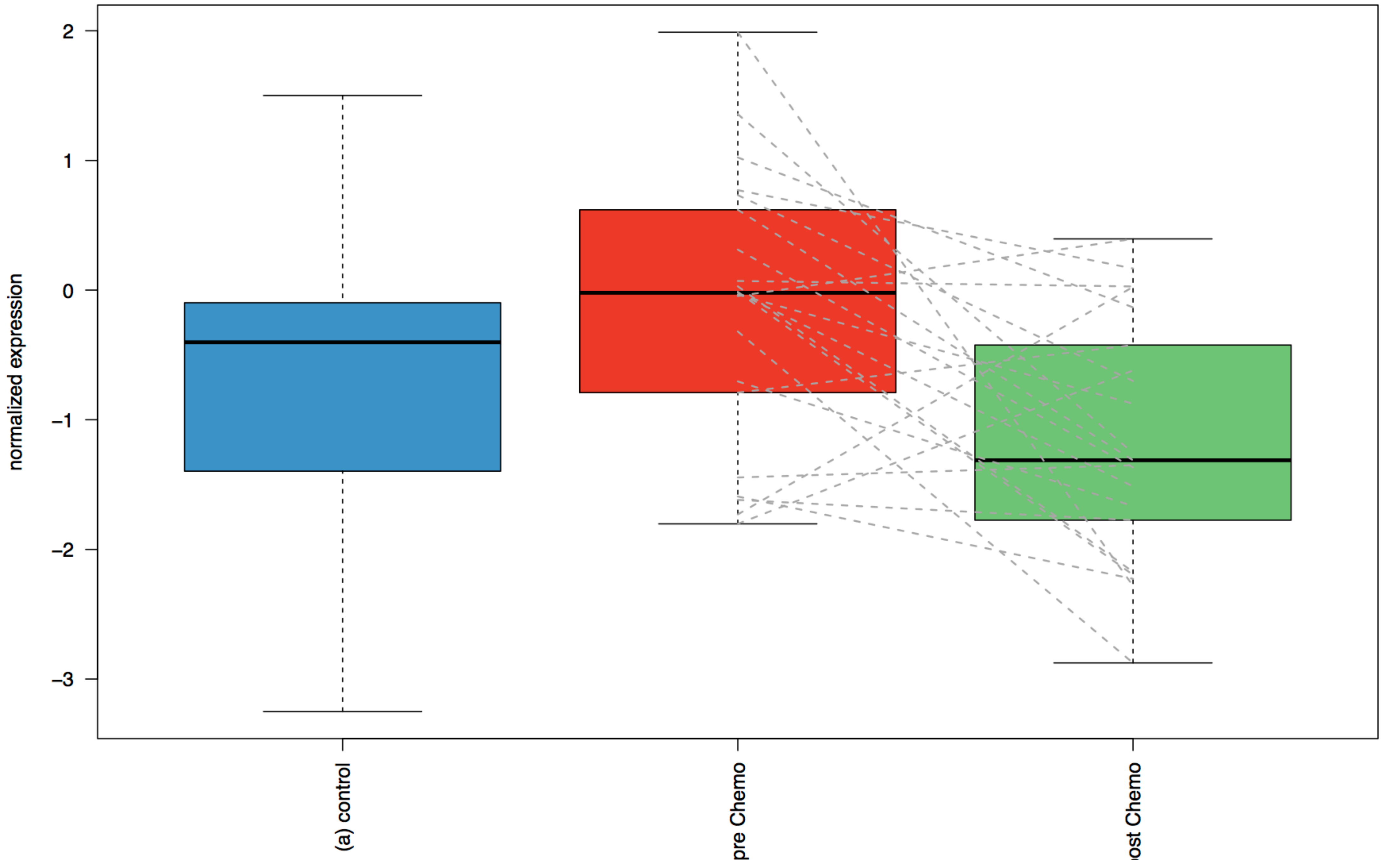
normalized expression
2
1
0
−1
−2
−3
(a) control
pre Chemo
ost Chemo

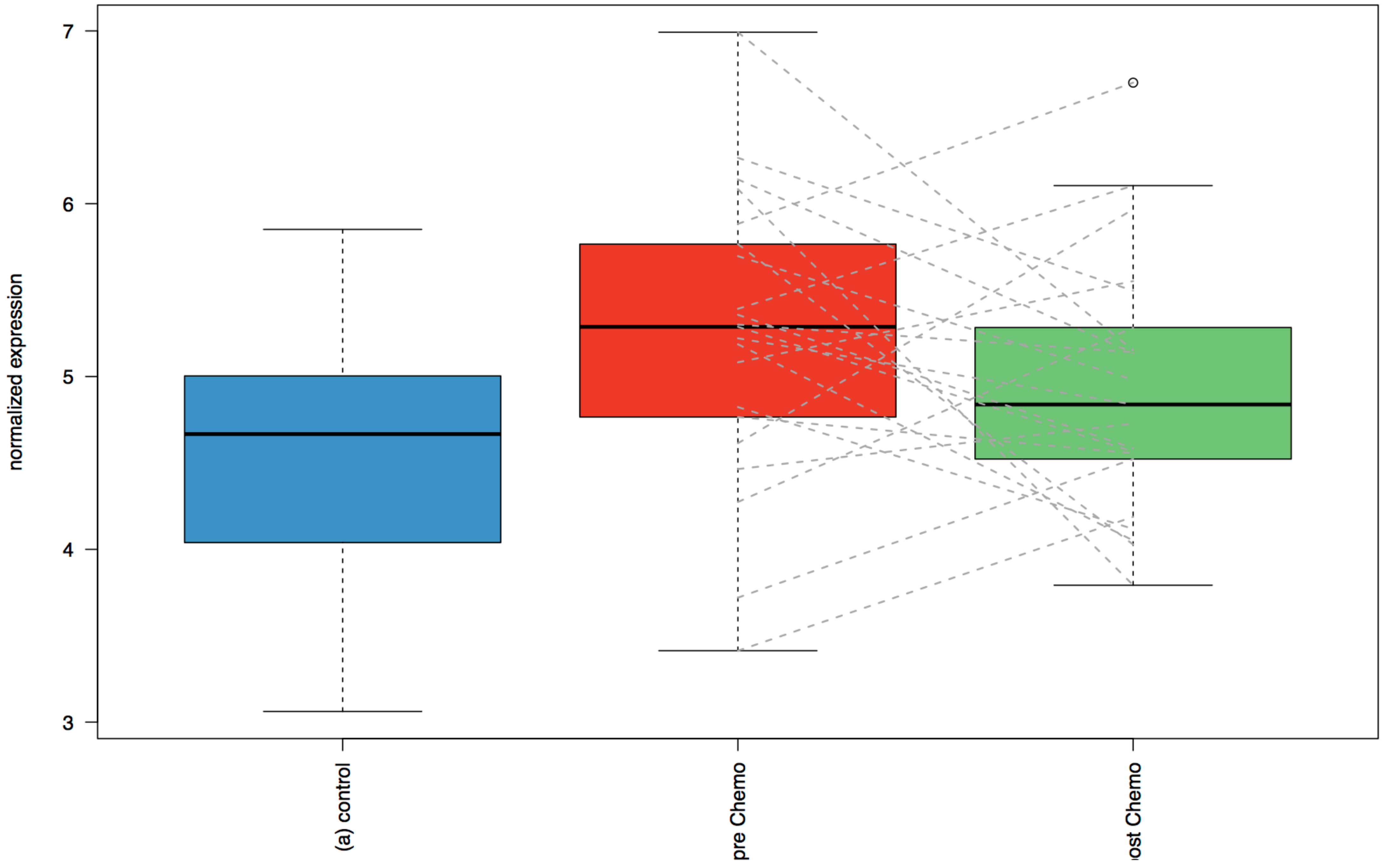
normalized expression
7
6
5
4
3
(a) control
pre Chemo
ost Chemo

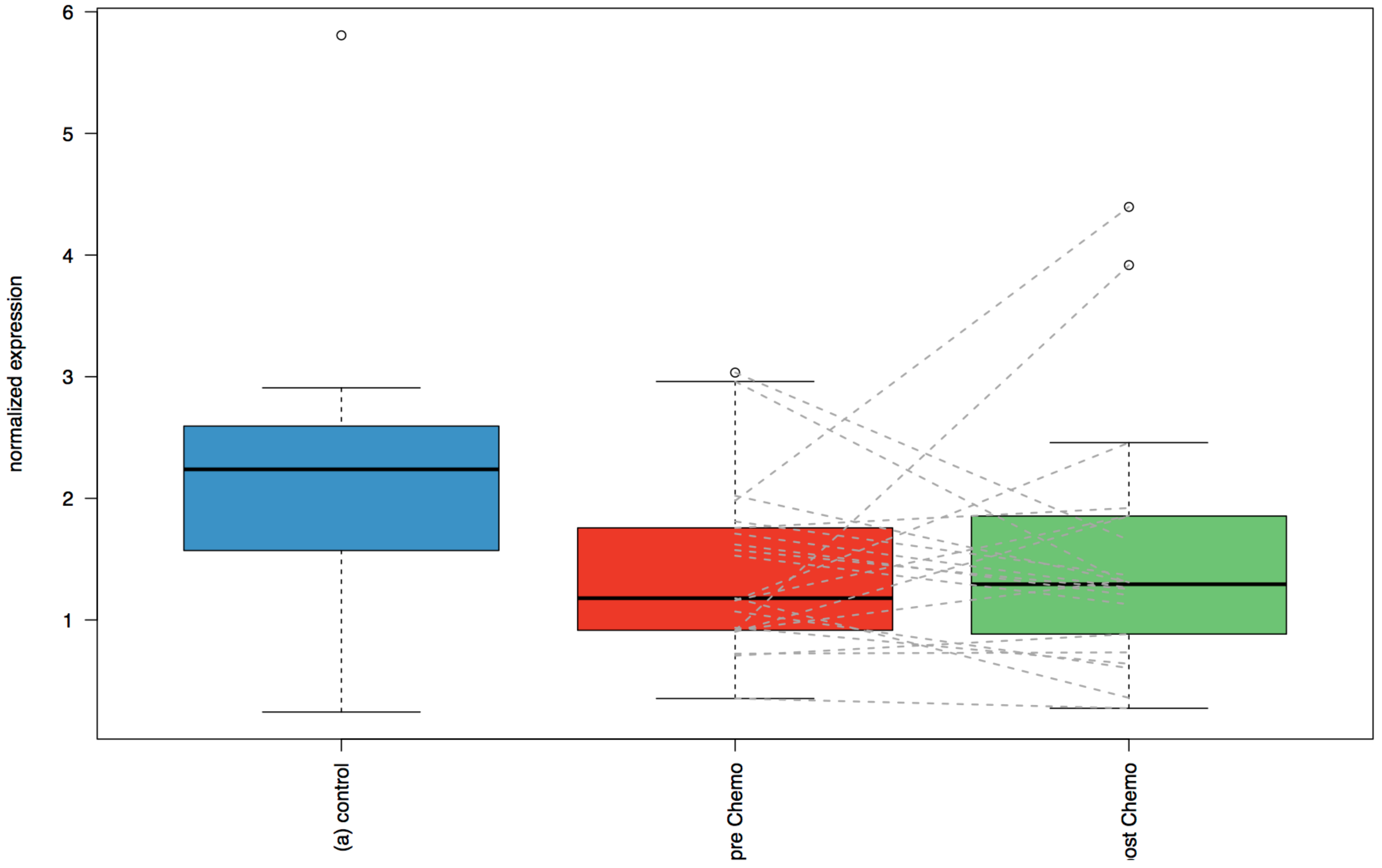
normalized expression
6
5
4
3
2
1
(a) control
pre Chemo
ost Chemo

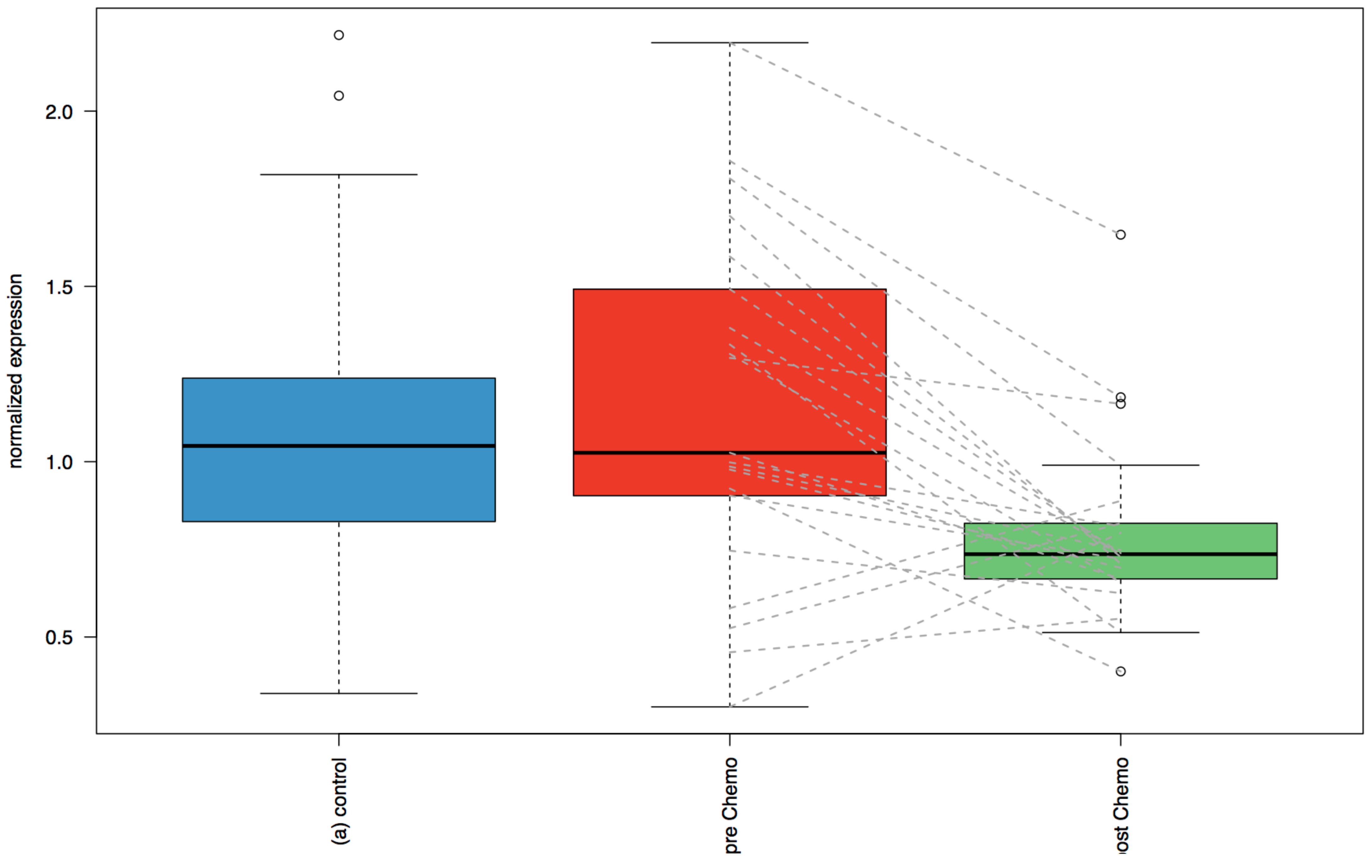
normalized expression
2.0
1.5
1.0
0.5
(a) control
pre Chemo
ost Chemo

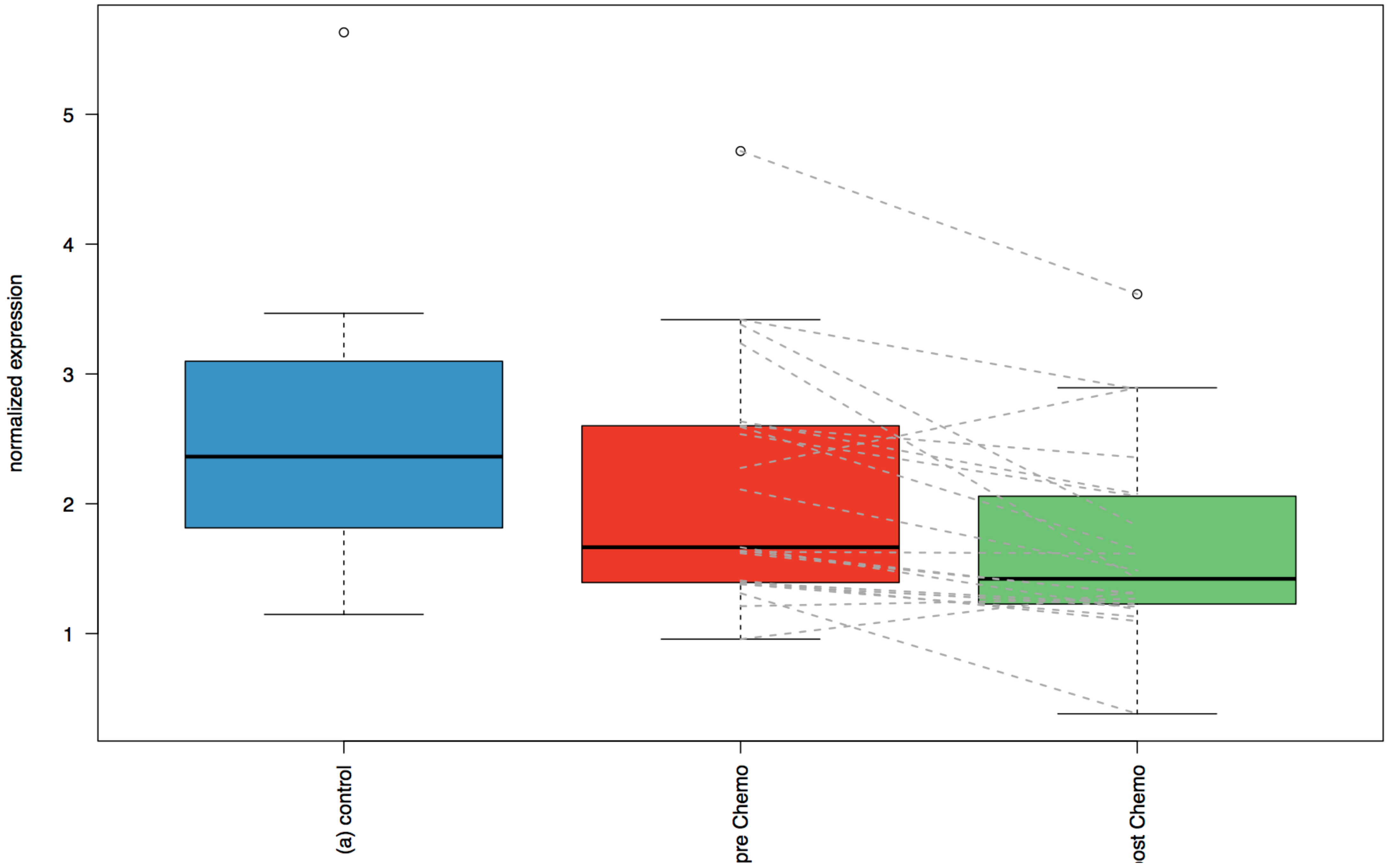

normalized expression
5
4
3
2
1
(a) control
pre Chemo
ost Chemo

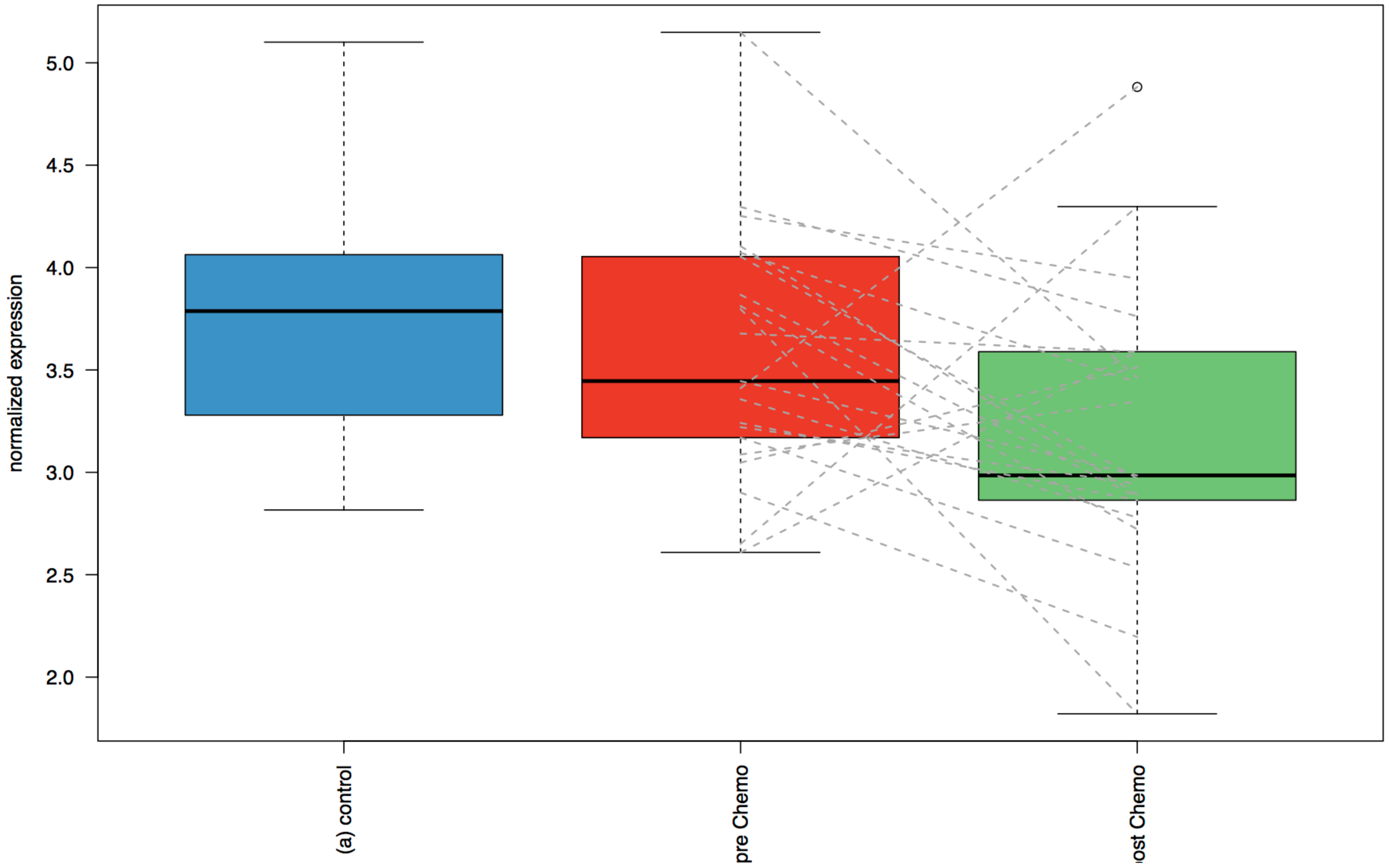
normalized expression
5.0
4.5
4.0
3.5
3.0
2.5
2.0
(a) control
pre Chemo
ost Chemo

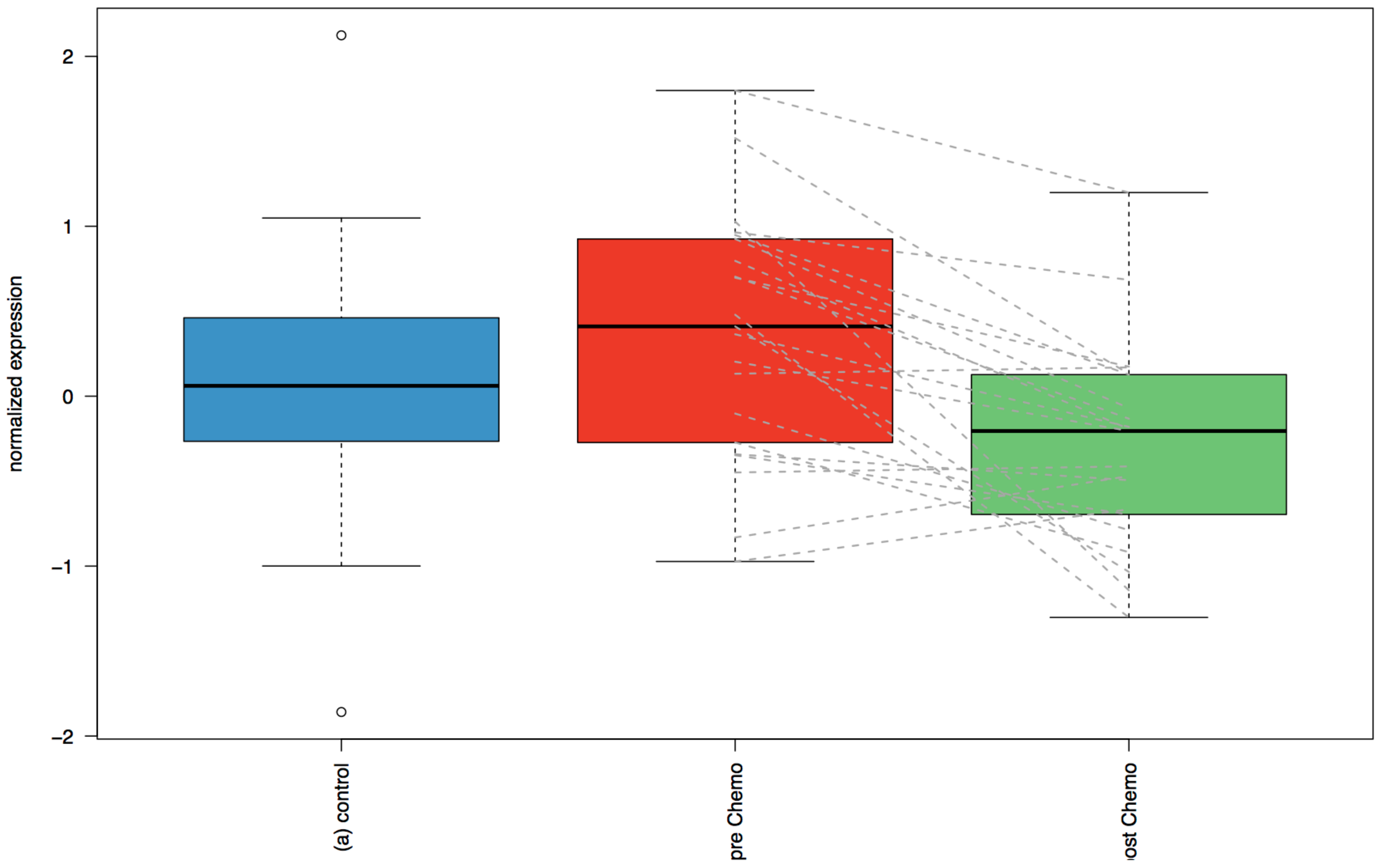

normalized expression
2
1
0
−1
−2
(a) control
pre Chemo
ost Chemo

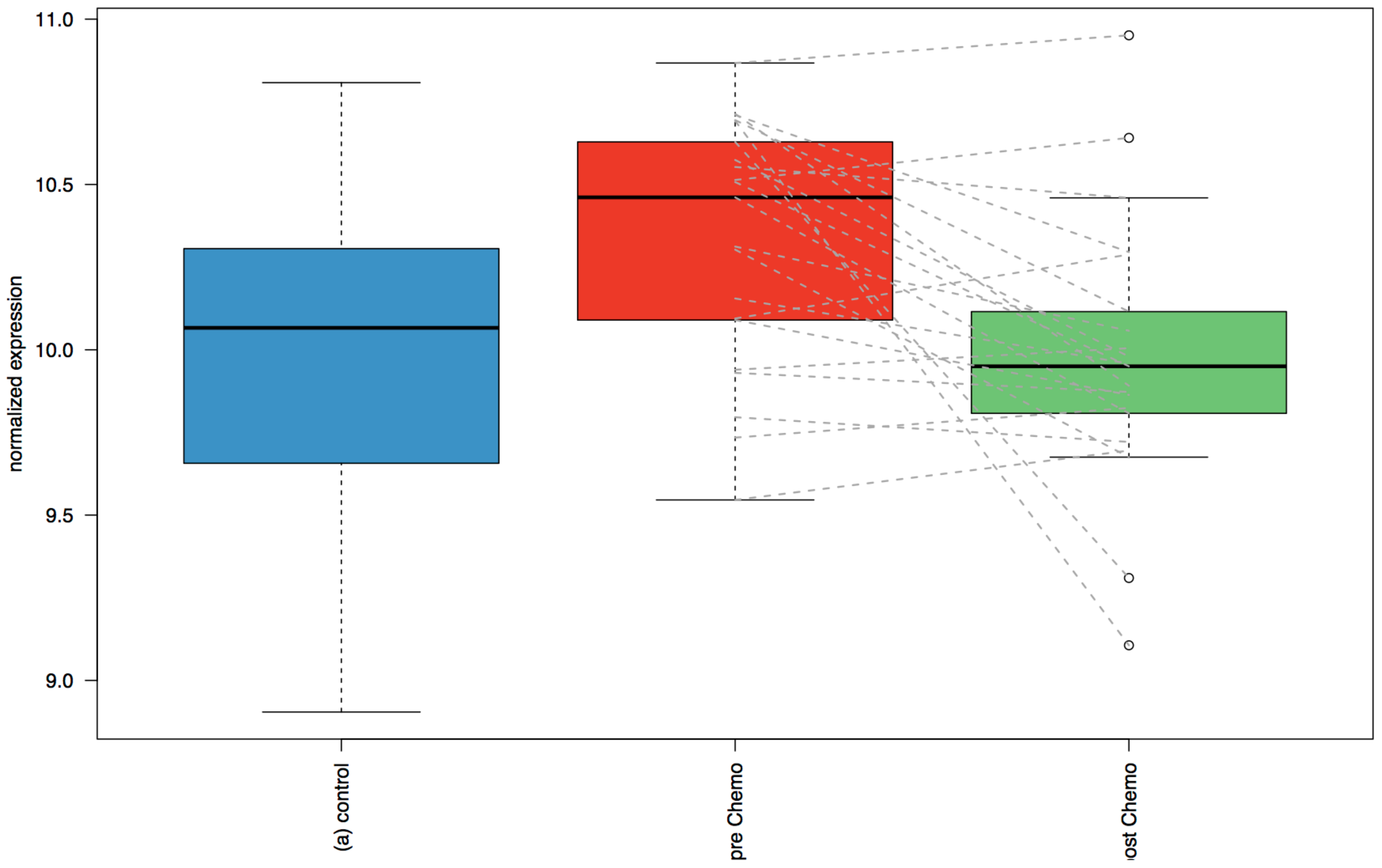

11.0
10.5
10.0
9.5
9.0
normalized expression
(a) control
pre Chemo
ost Chemo

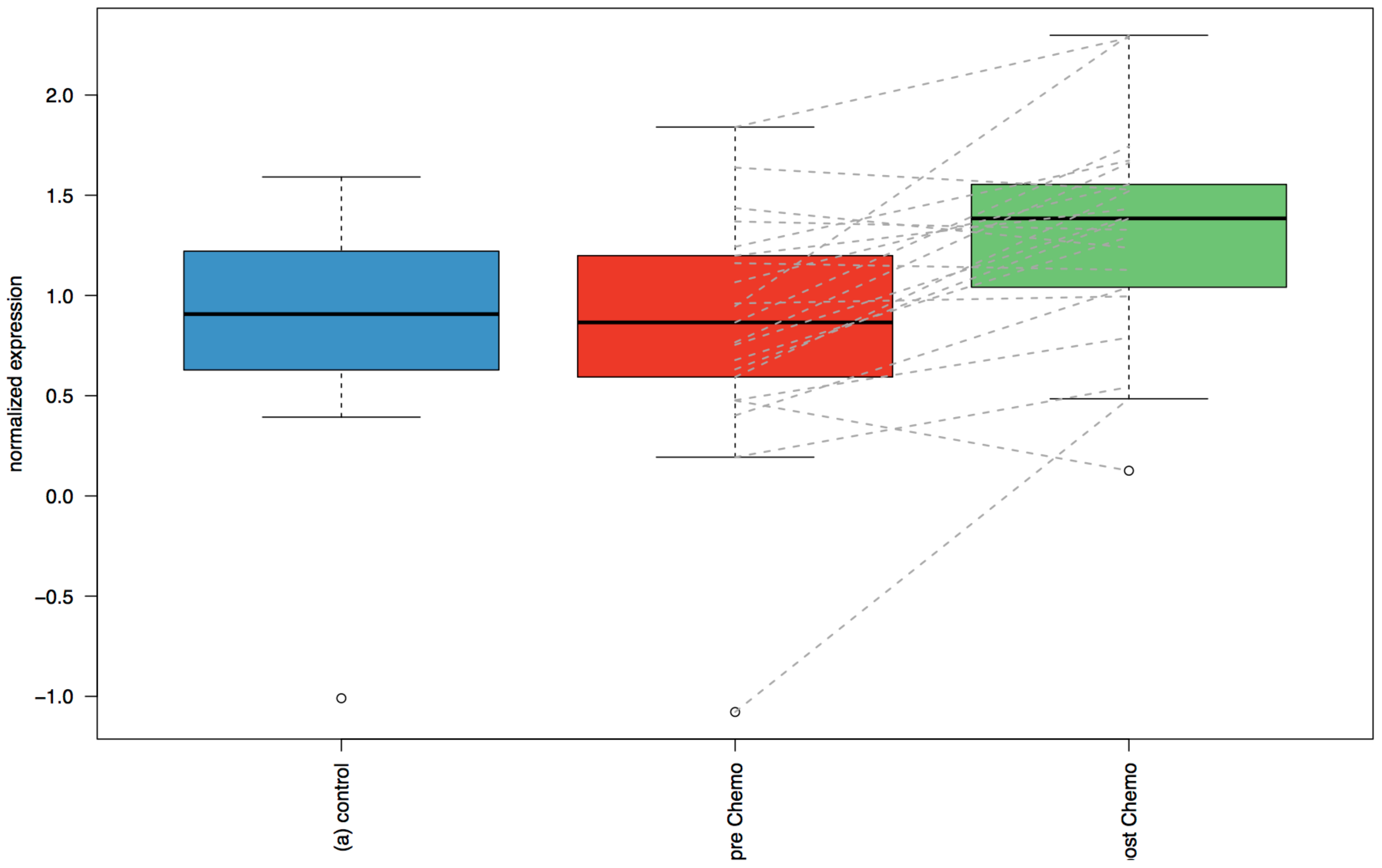
normalized expression
2.0
1.5
1.0
0.5
0.0
−0.5
−1.0
(a) control
pre Chemo
ost Chemo

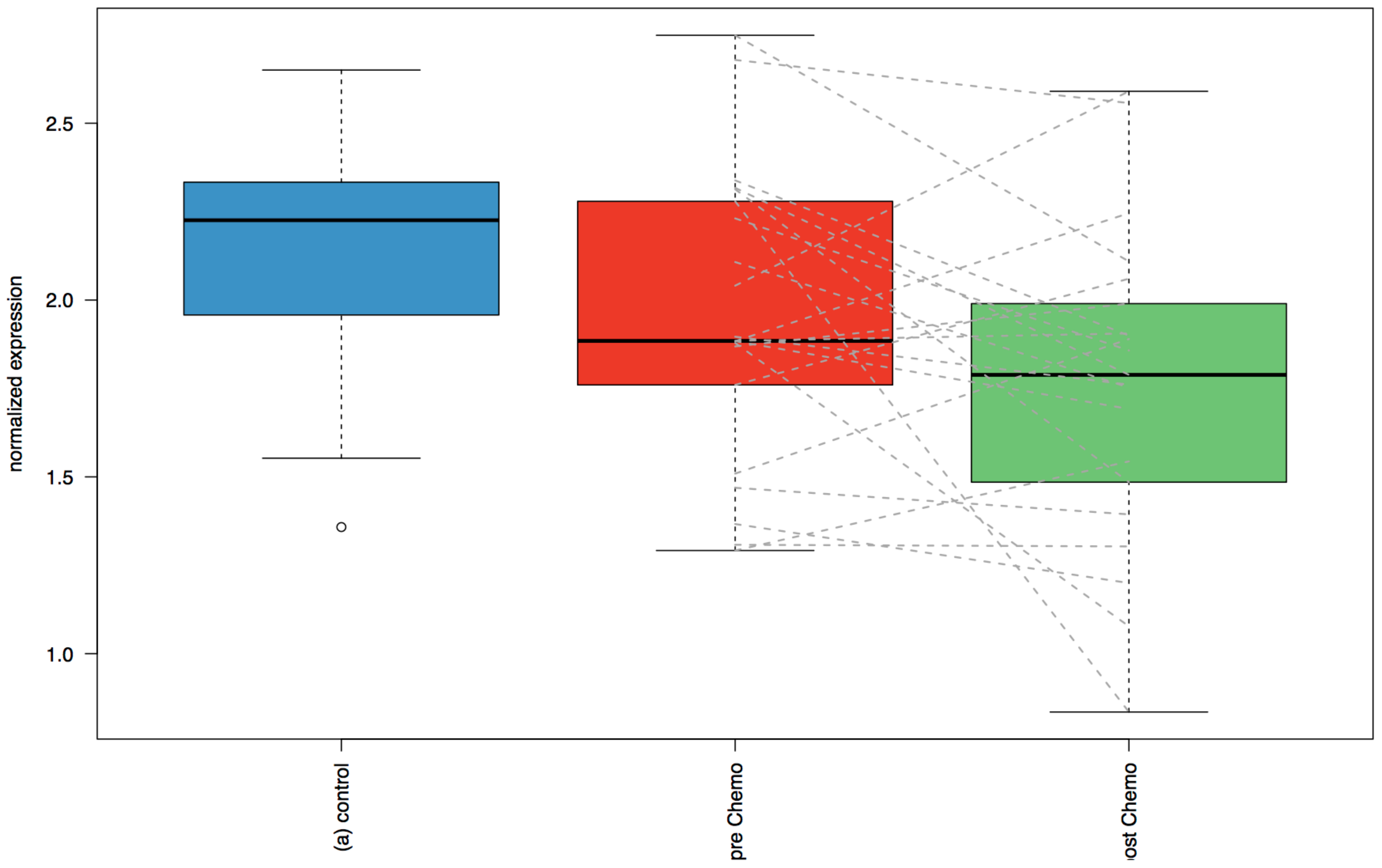
normalized expression
2.5
2.0
1.5
1.0
(a) control
pre Chemo
ost Chemo

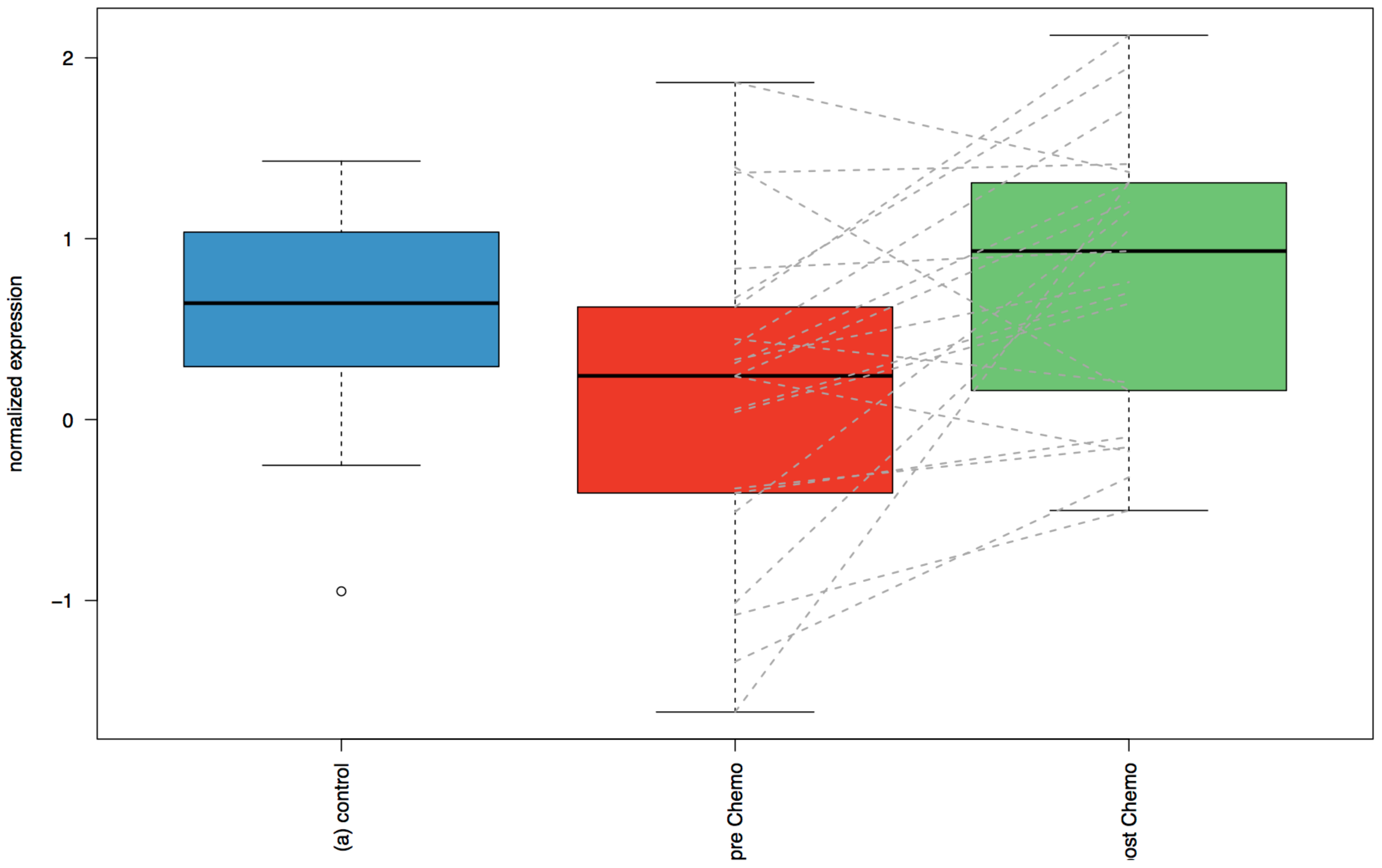
normalized expression
2
1
0
−1
(a) control
pre Chemo
ost Chemo

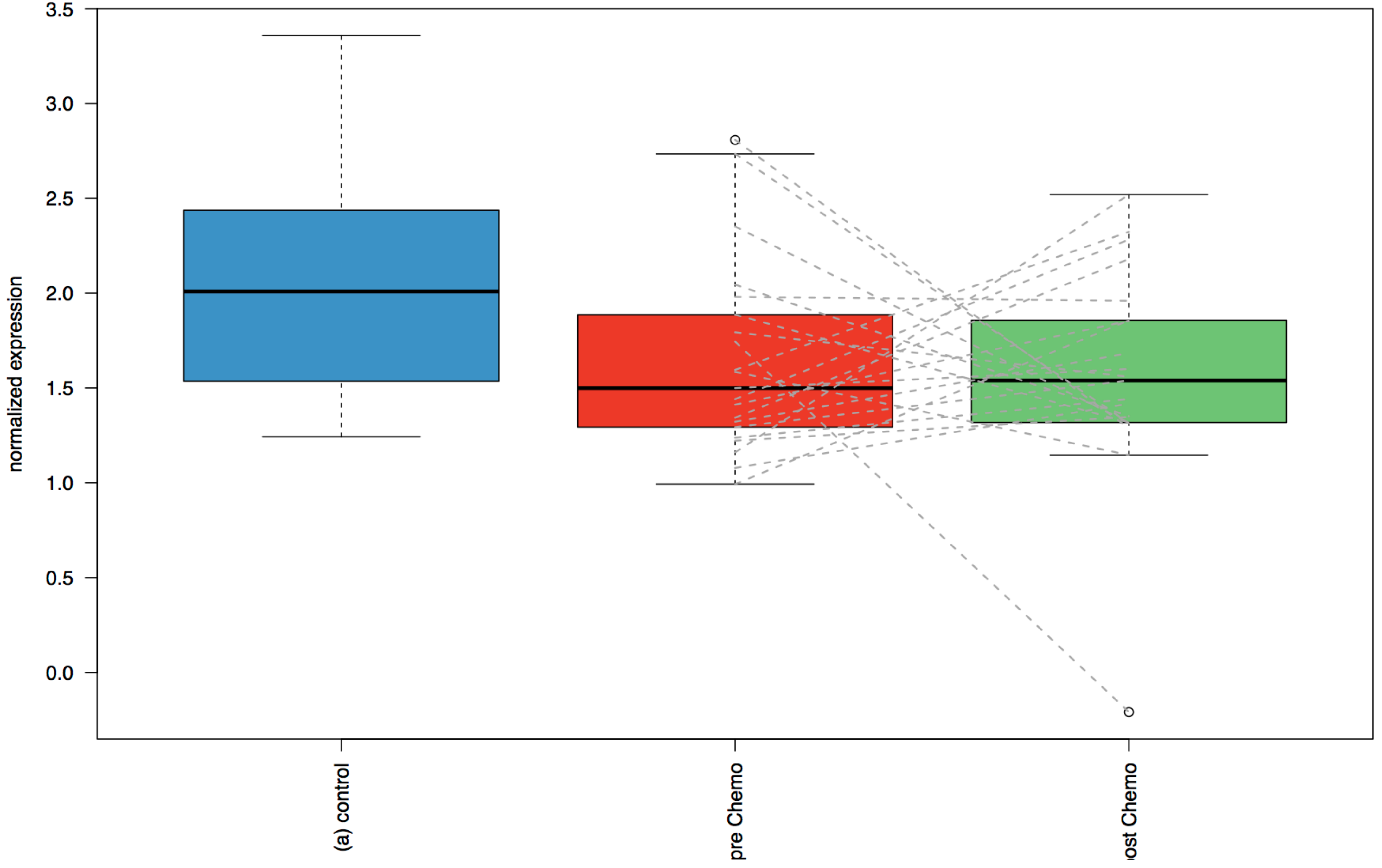

normalized expression
3.5
3.0
2.5
2.0
1.5
1.0
0.5
0.0
(a) control
pre Chemo
ost Chemo

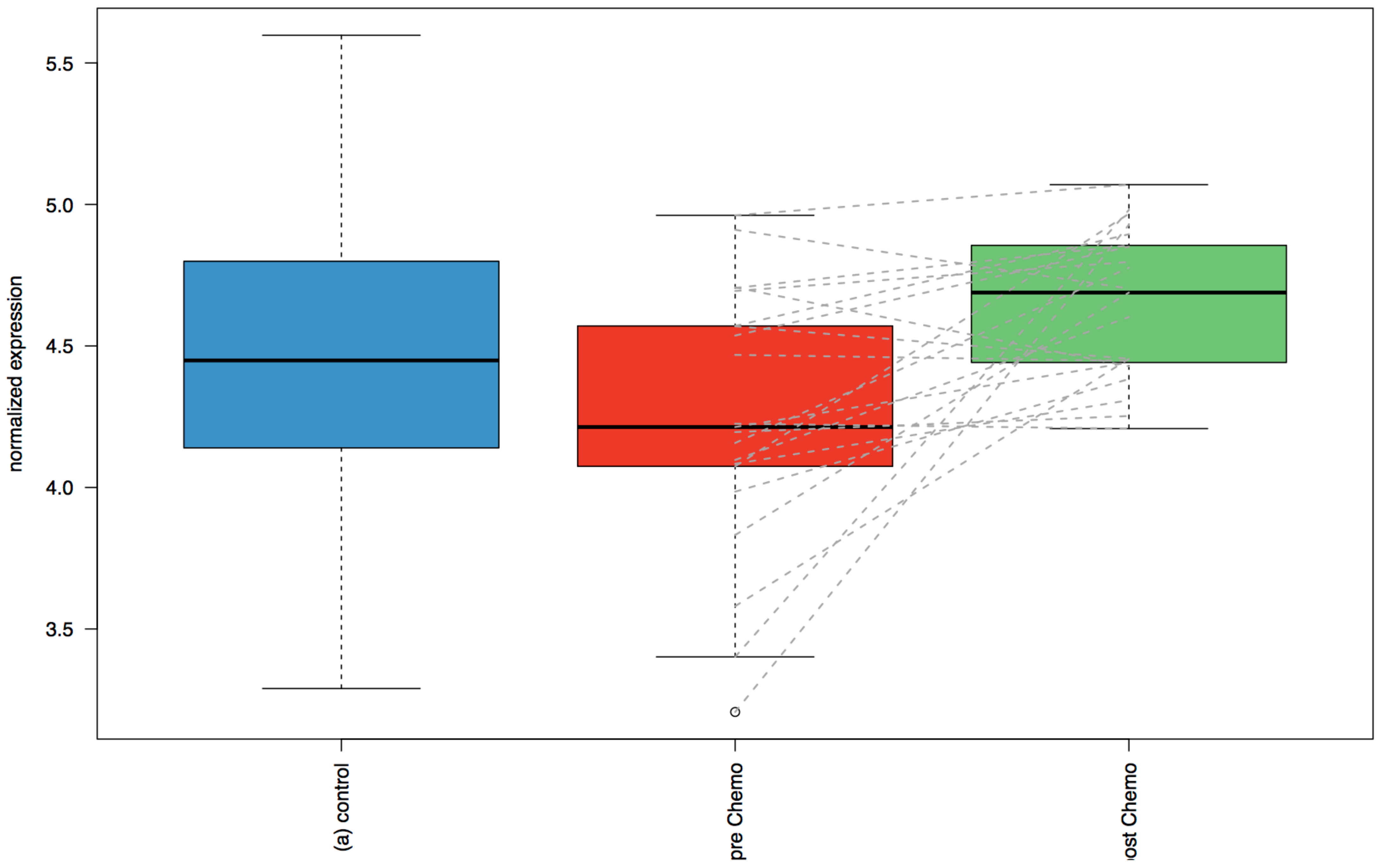

normalized expression
5.5
5.0
4.5
4.0
3.5
(a) control
pre Chemo
ost Chemo

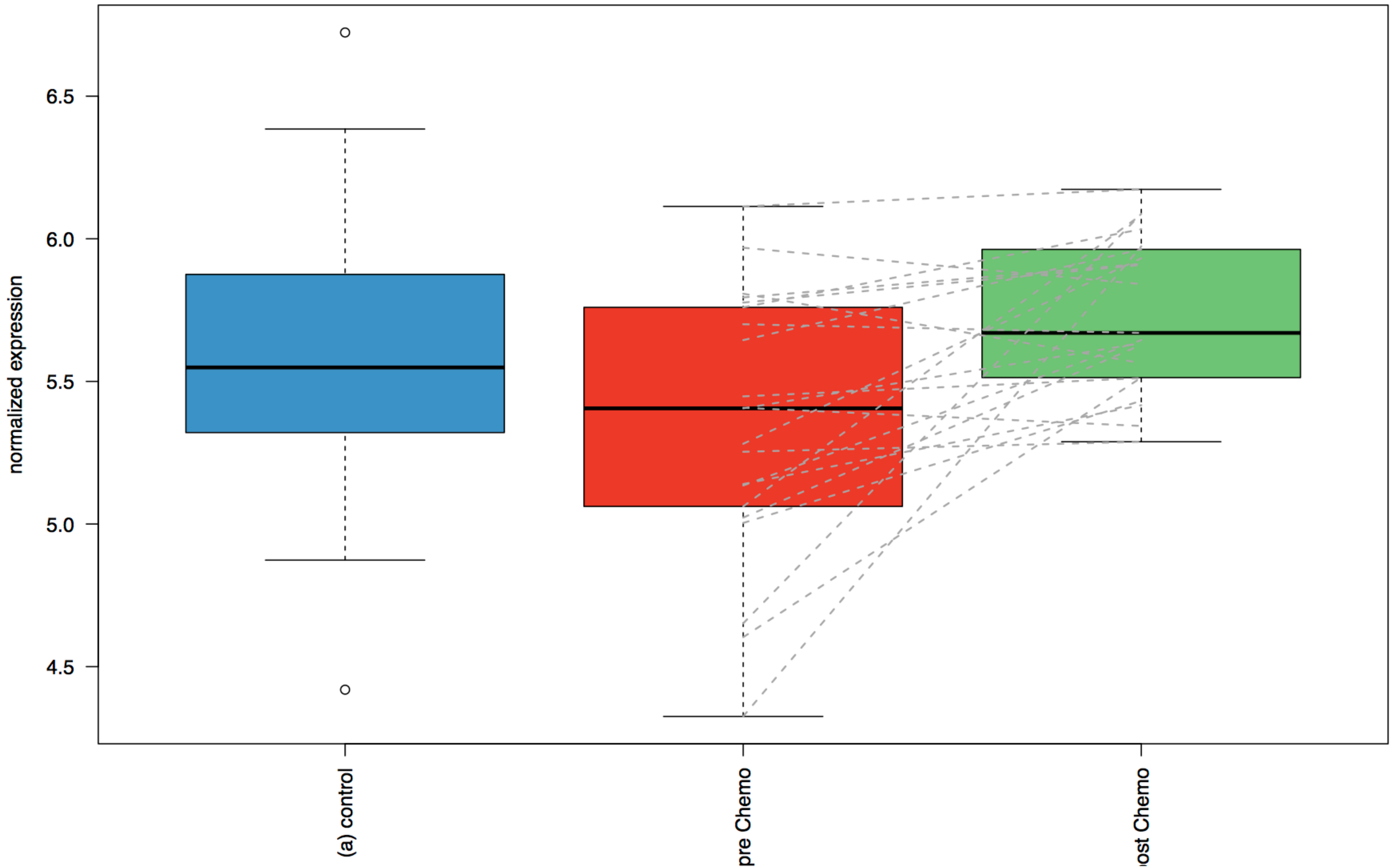
normalized expression
6.5
6.0
5.5
5.0
4.5
(a) control
pre Chemo
ost Chemo

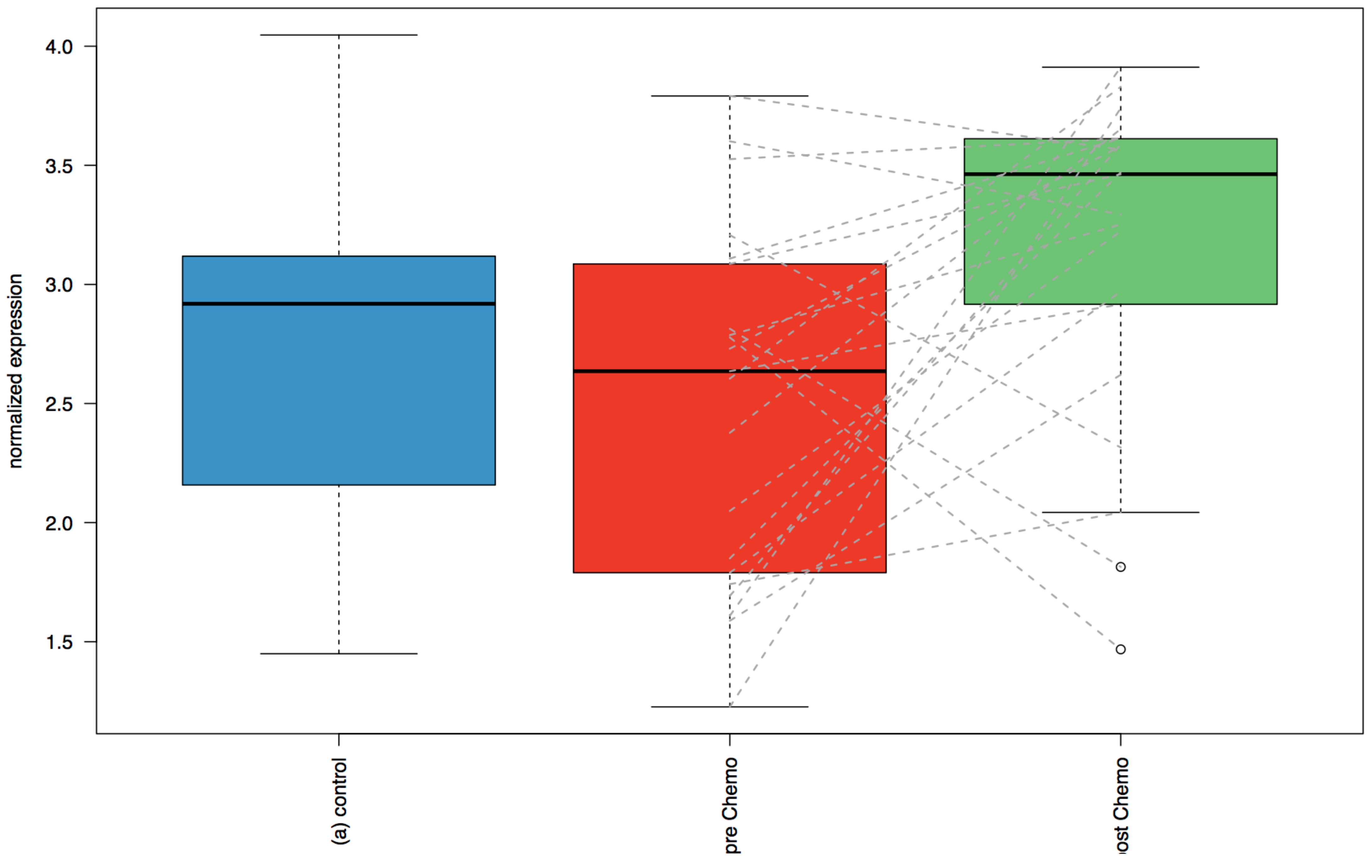
normalized expression
4.0
3.5
3.0
2.5
2.0
1.5
(a) control
pre Chemo
ost Chemo

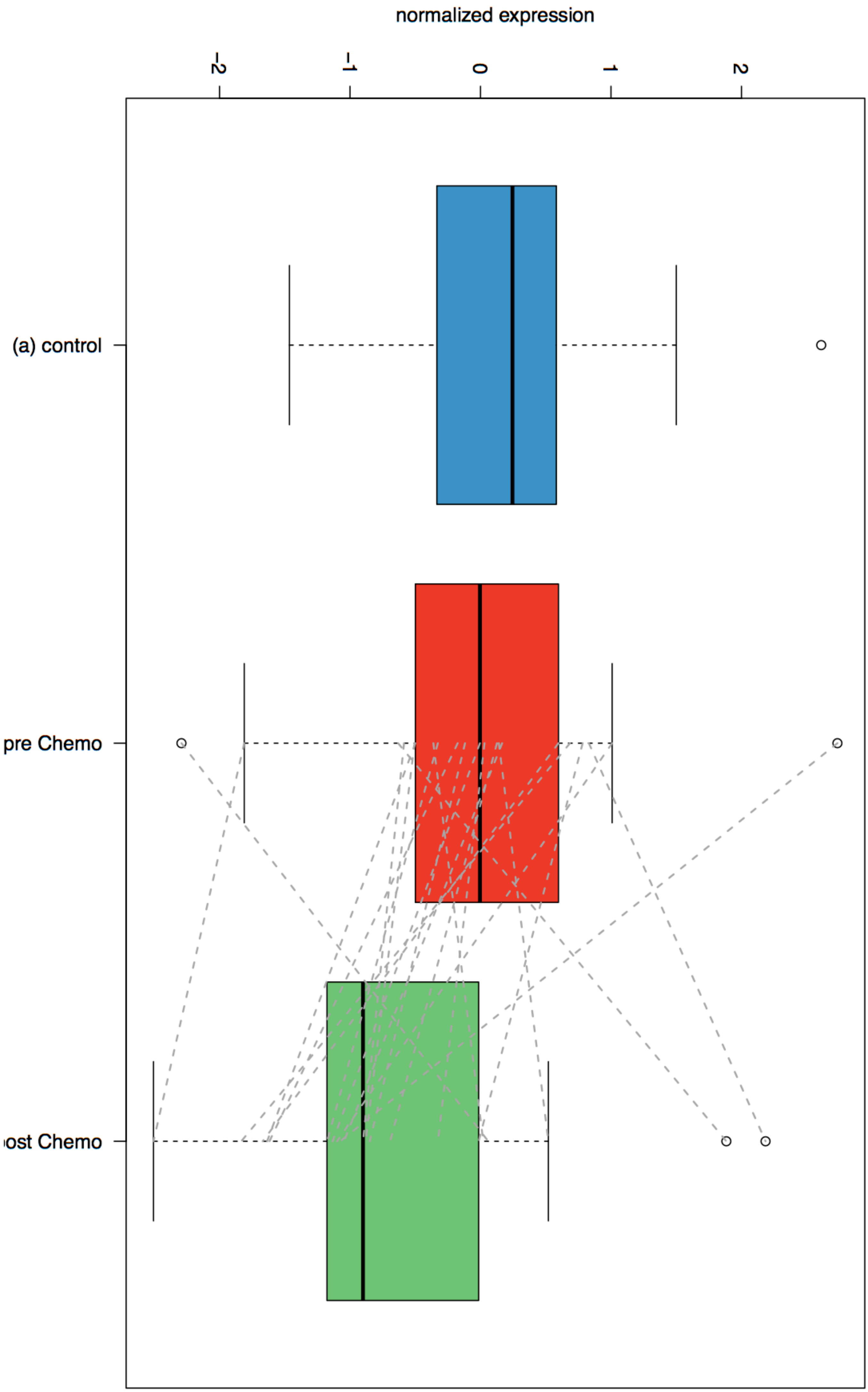
normalized expression
-2
-1
0
1
2
(a) control
pre Chemo
ost Chemo

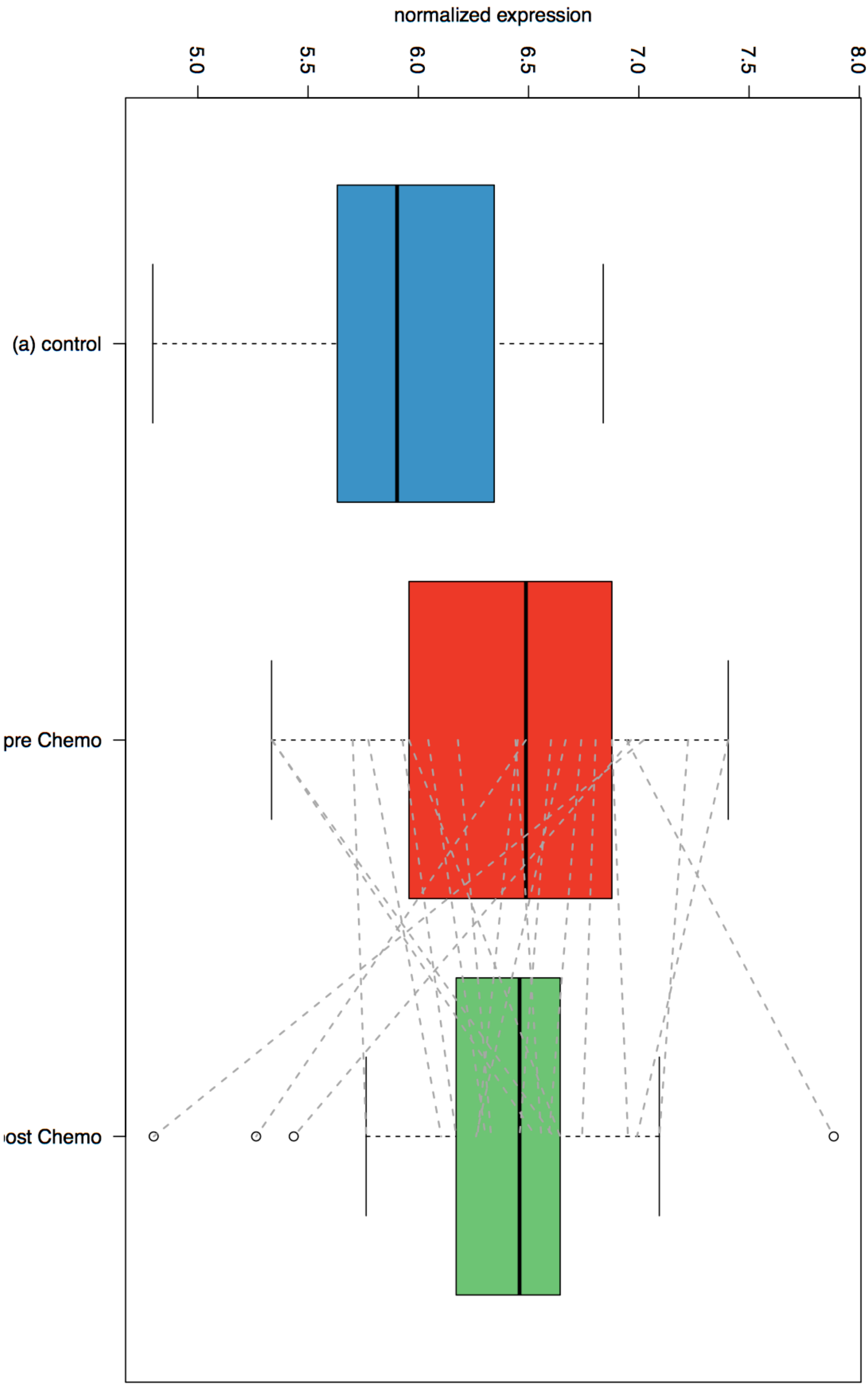

normalized expression
5.0
5.5
6.0
6.5
7.0
7.5
8.0
(a) control
pre Chemo
ost Chemo

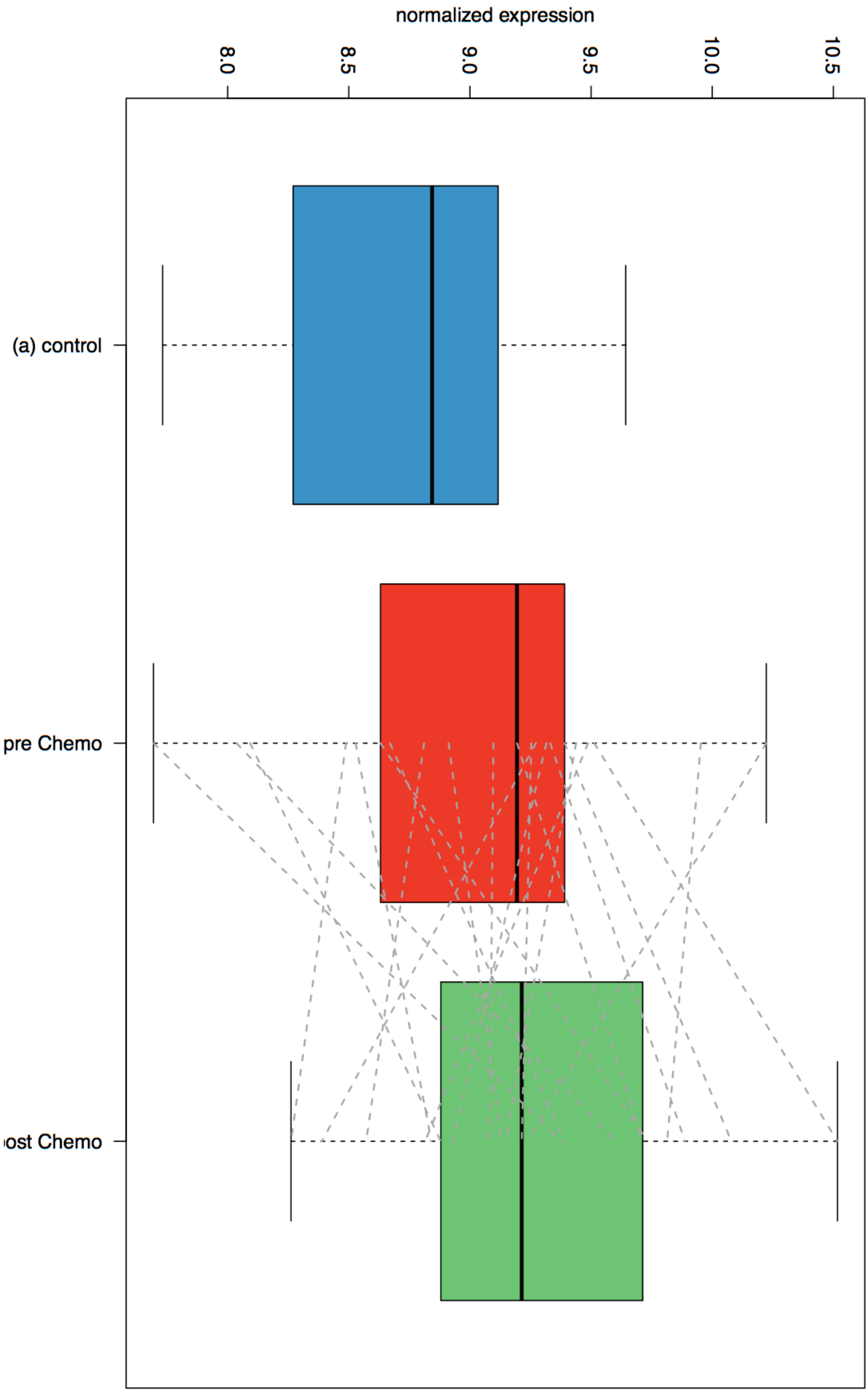
normalized expression
8.0
8.5
9.0
9.5
10.0
10.5
(a) control
pre Chemo
ost Chemo

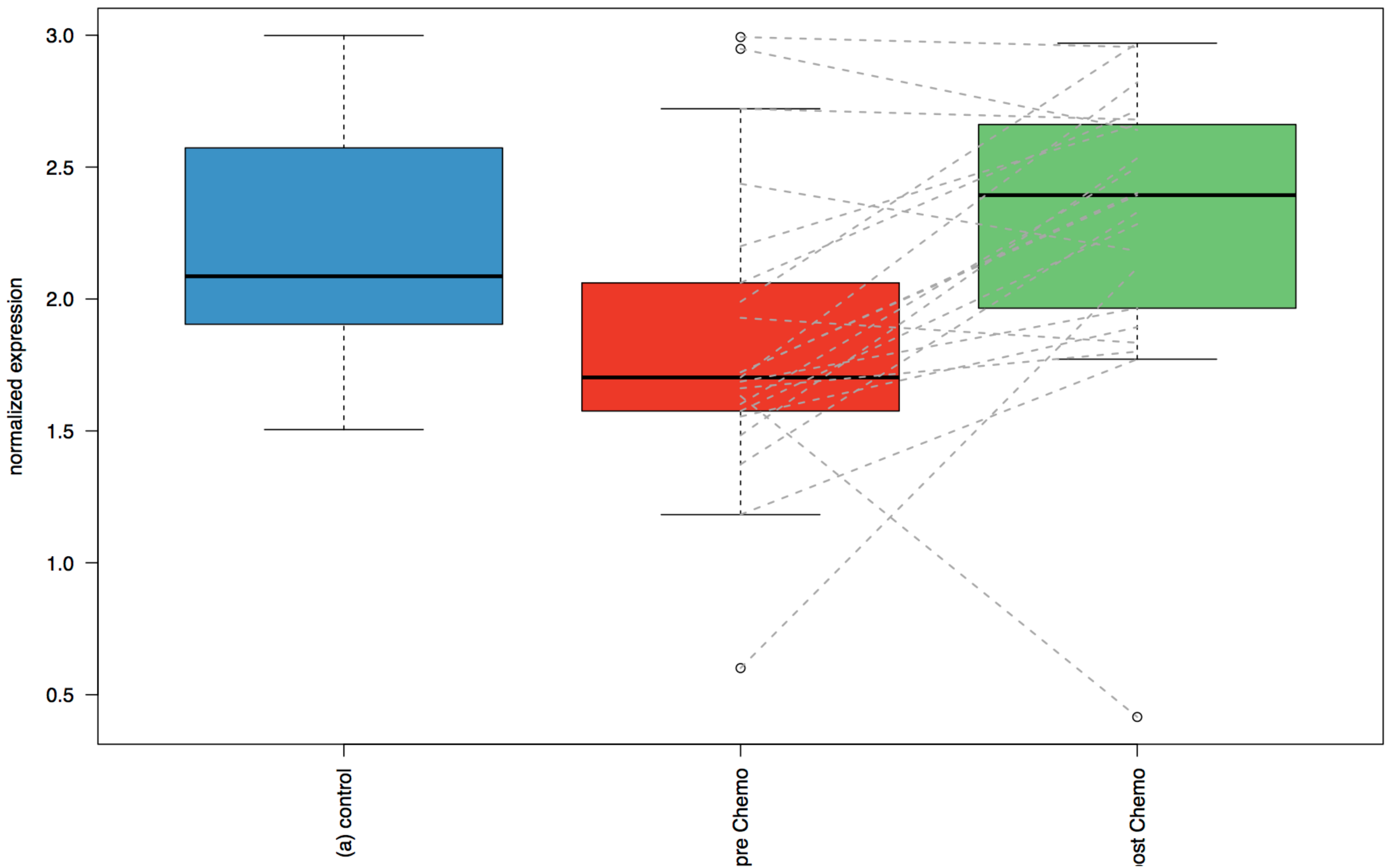
normalized expression
3.0
2.5
2.0
1.5
1.0
0.5
(a) control
pre Chemo
ost Chemo

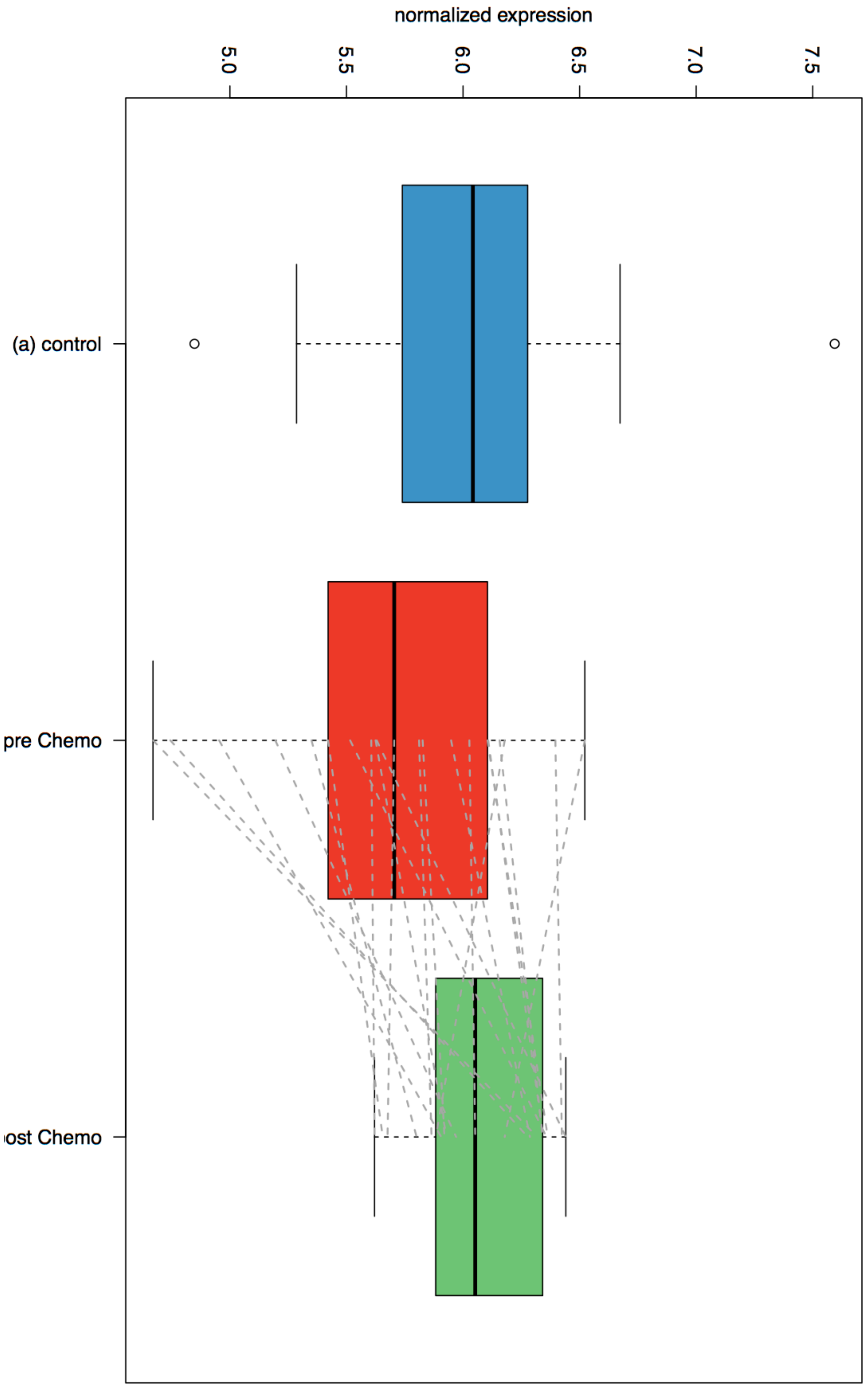
normalized expression
5.0
5.5
6.0
6.5
7.0
7.5
(a) control
pre Chemo
ost Chemo

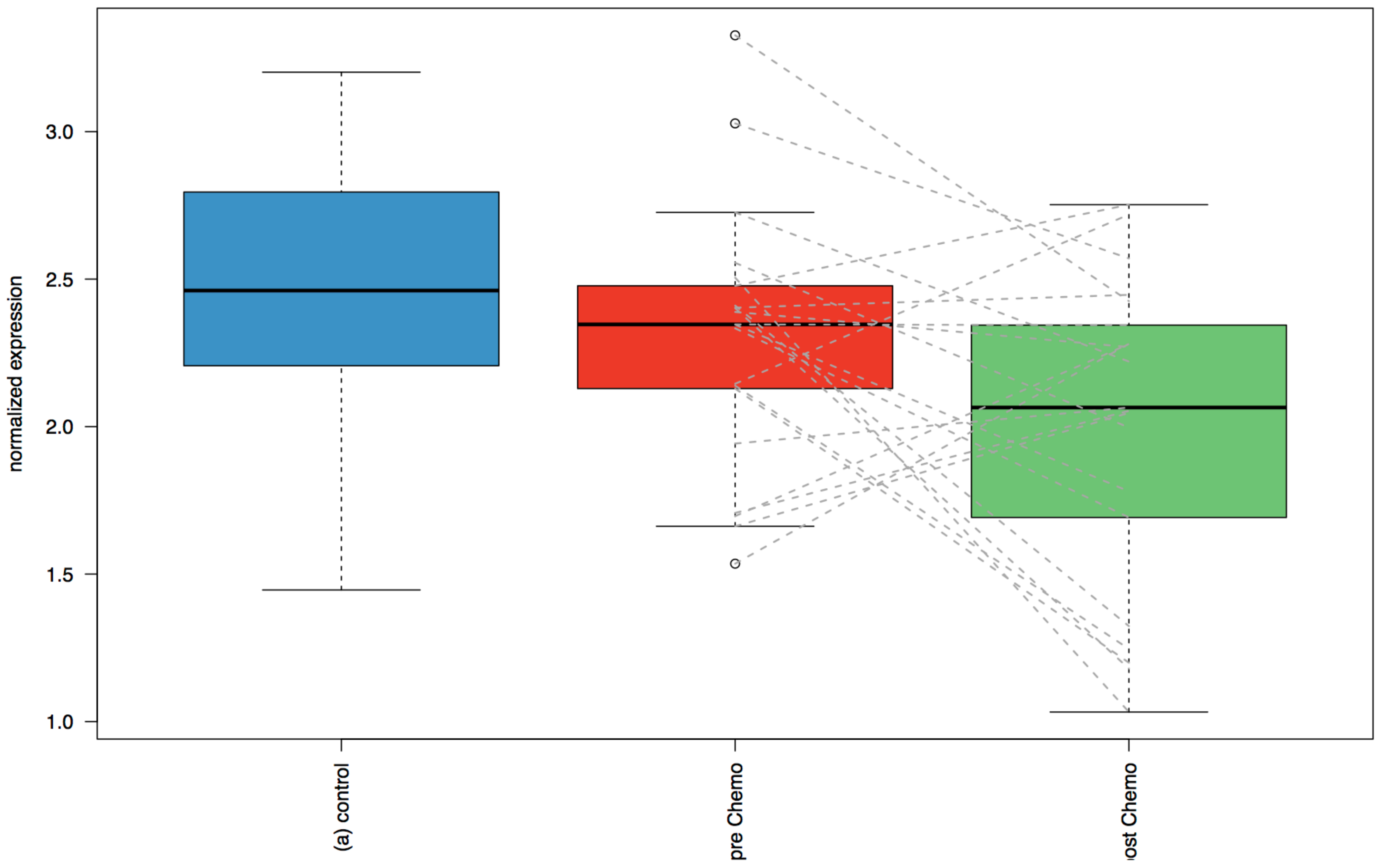
normalized expression
3.0
2.5
2.0
1.5
1.0
(a) control
pre Chemo
ost Chemo

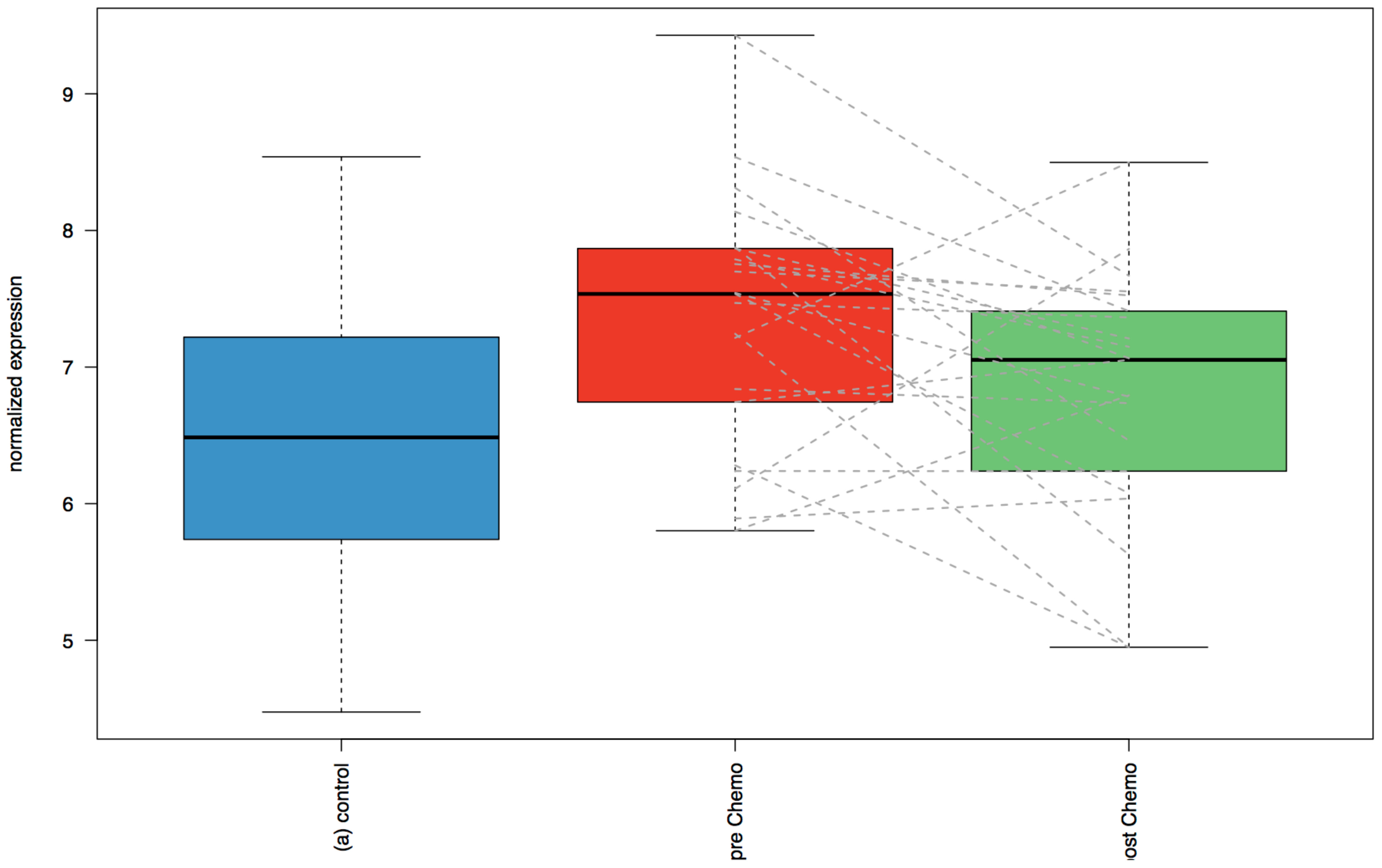
normalized expression
9
8
7
6
5
(a) control
pre Chemo
ost Chemo

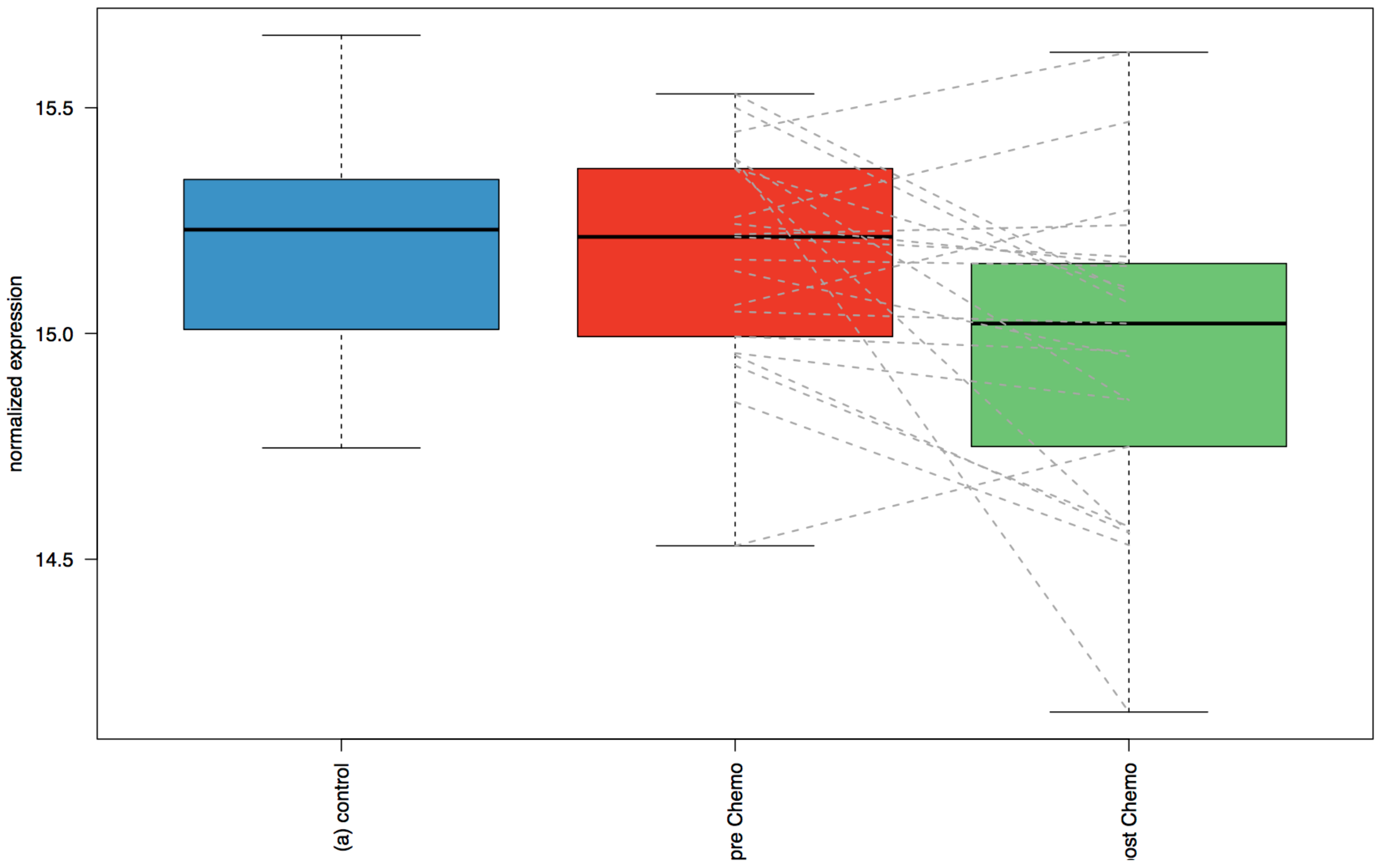

normalized expression
15.5
15.0
14.5
(a) control
pre Chemo
ost Chemo

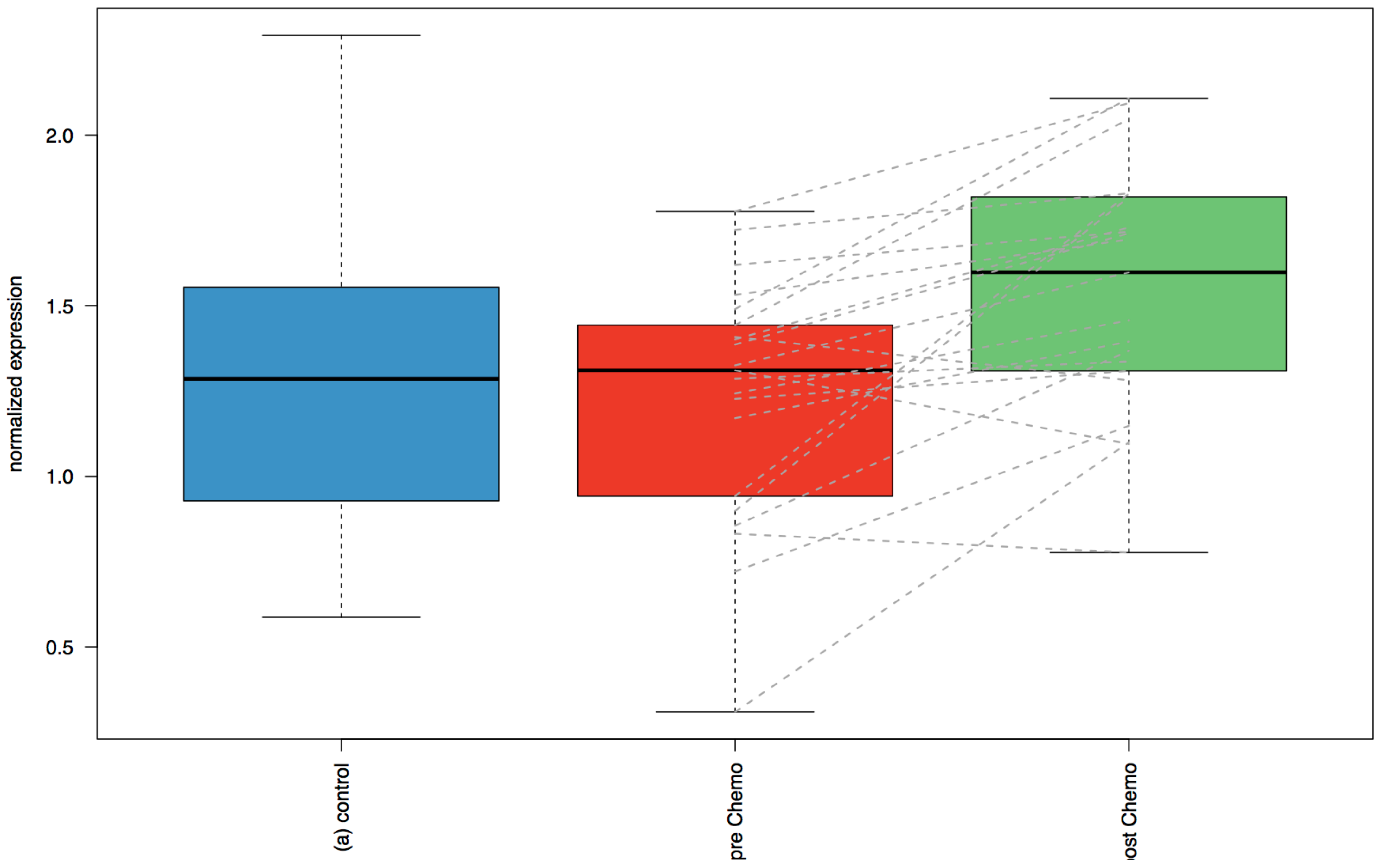

normalized expression
2.0
1.5
1.0
0.5
(a) control
pre Chemo
ost Chemo

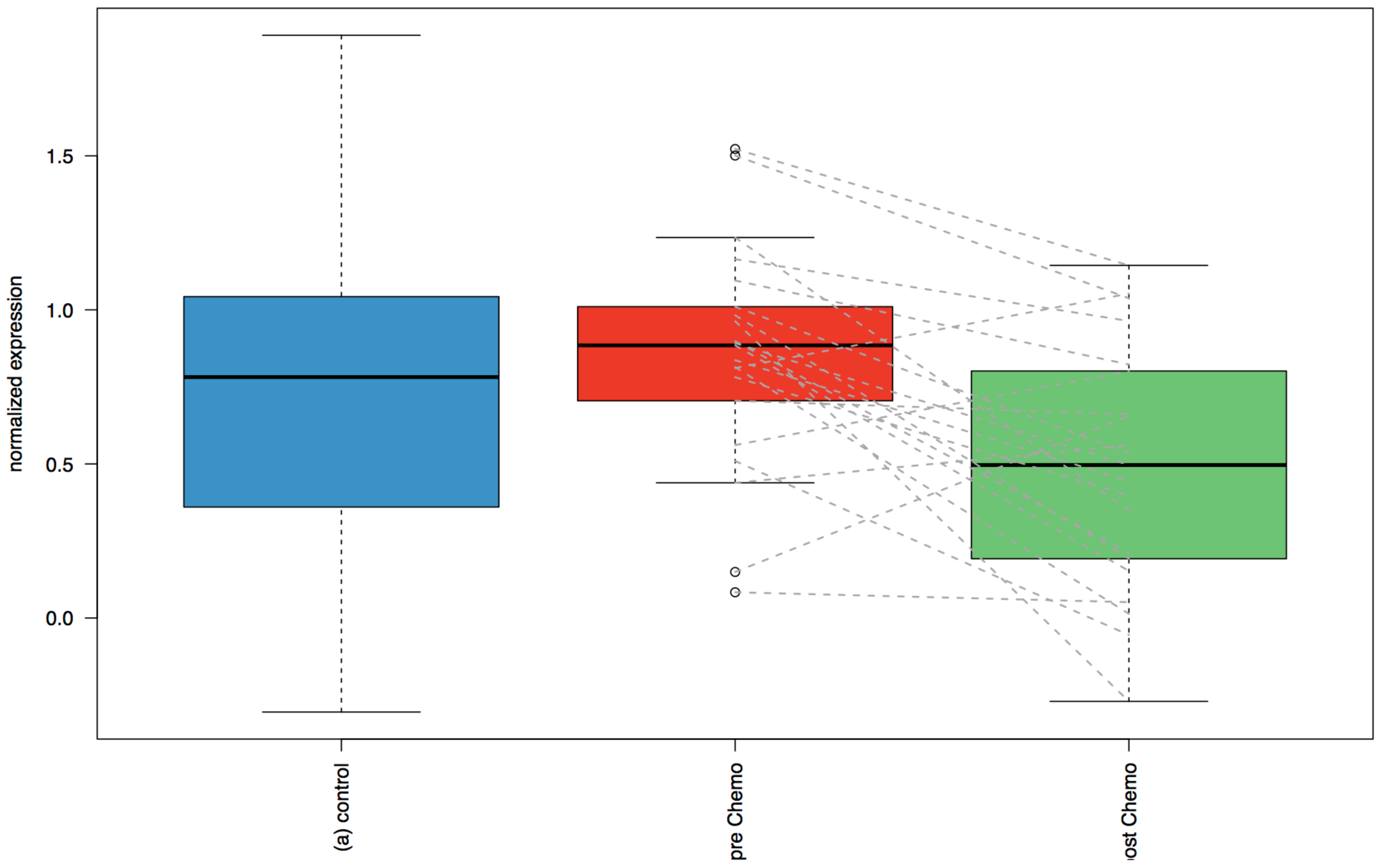

normalized expression
0.0
0.5
1.0
1.5
(a) control
pre Chemo
ost Chemo

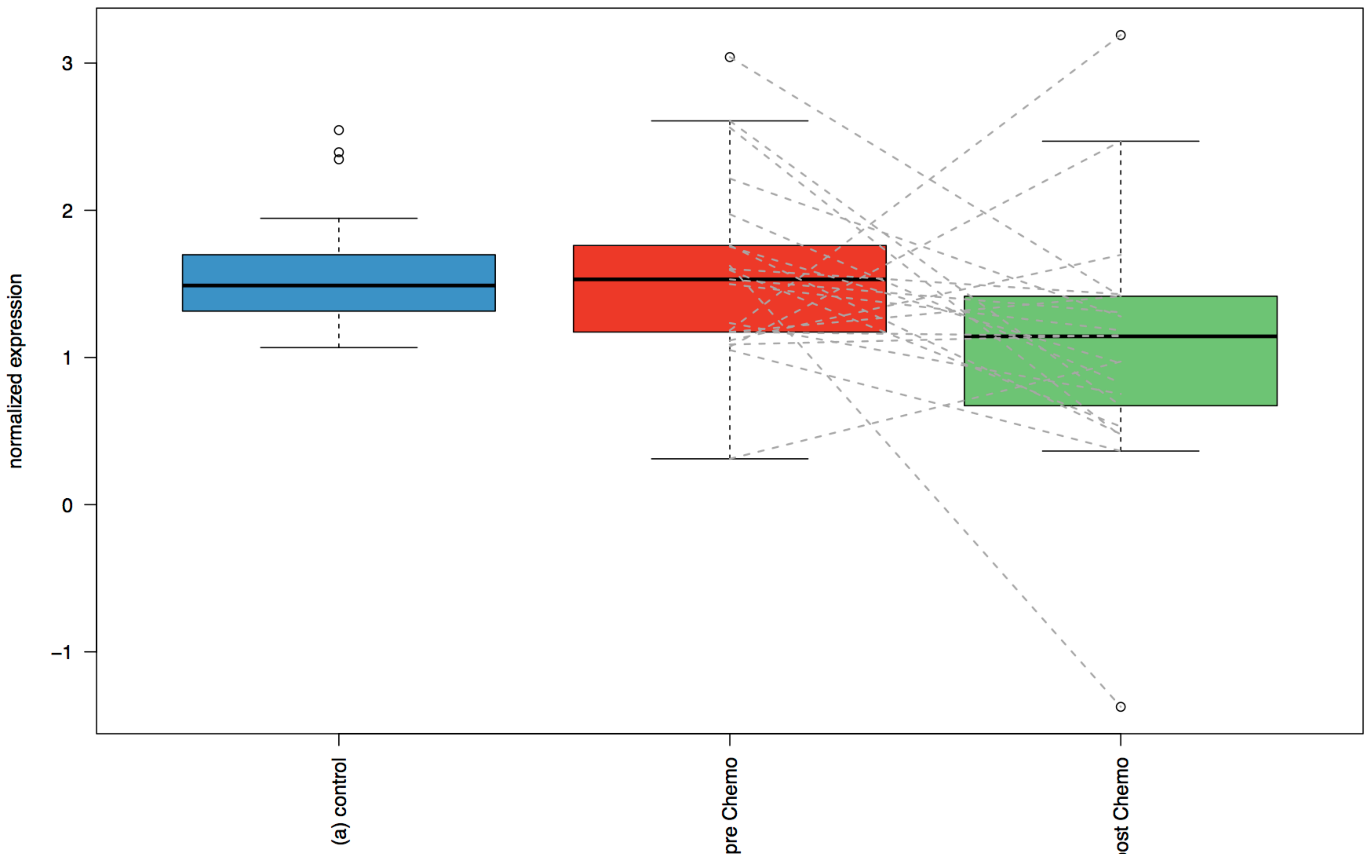

normalized expression
3
2
1
0
−1
(a) control
pre Chemo
ost Chemo

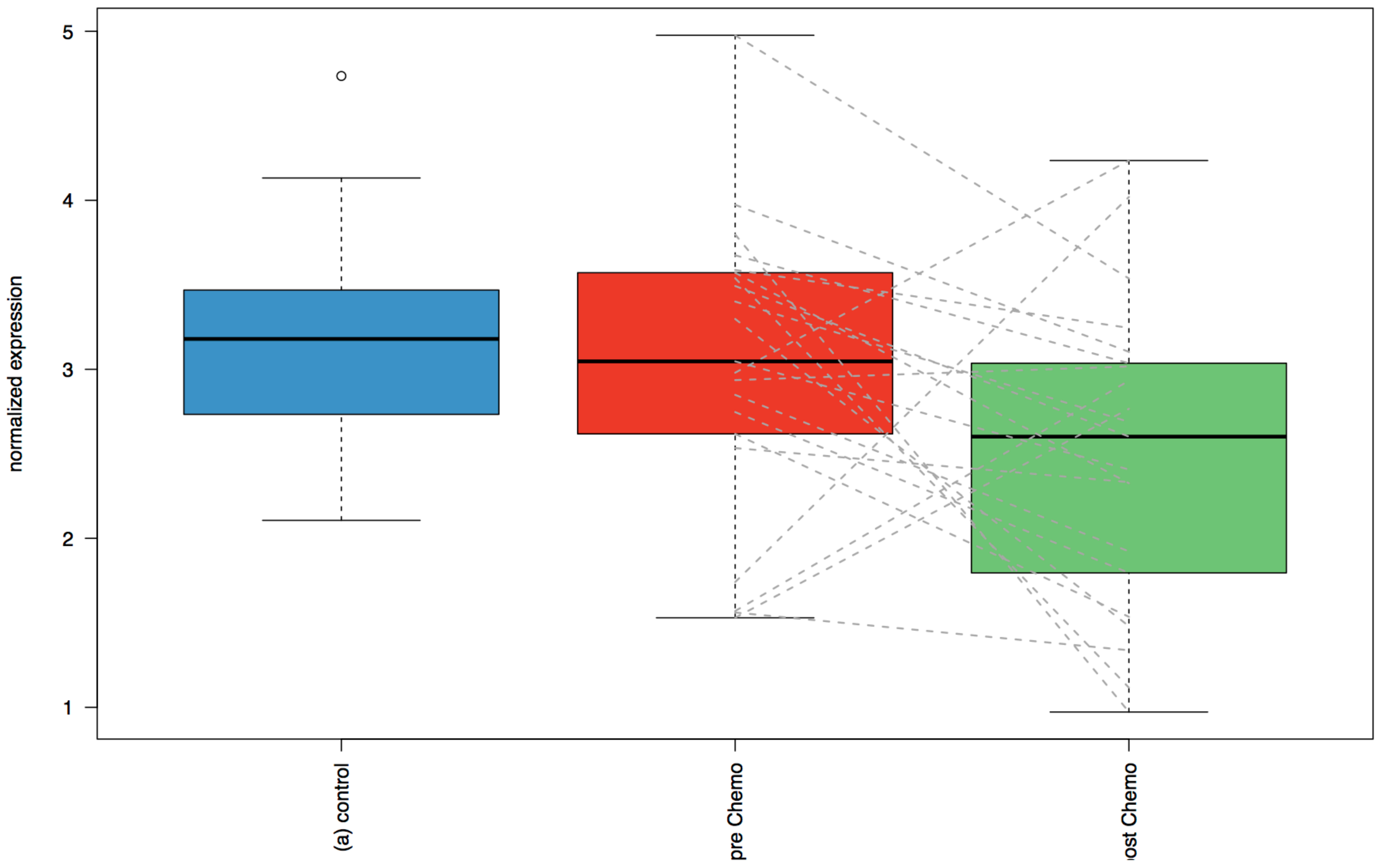
normalized expression
5
4
3
2
1
(a) control
pre Chemo
ost Chemo

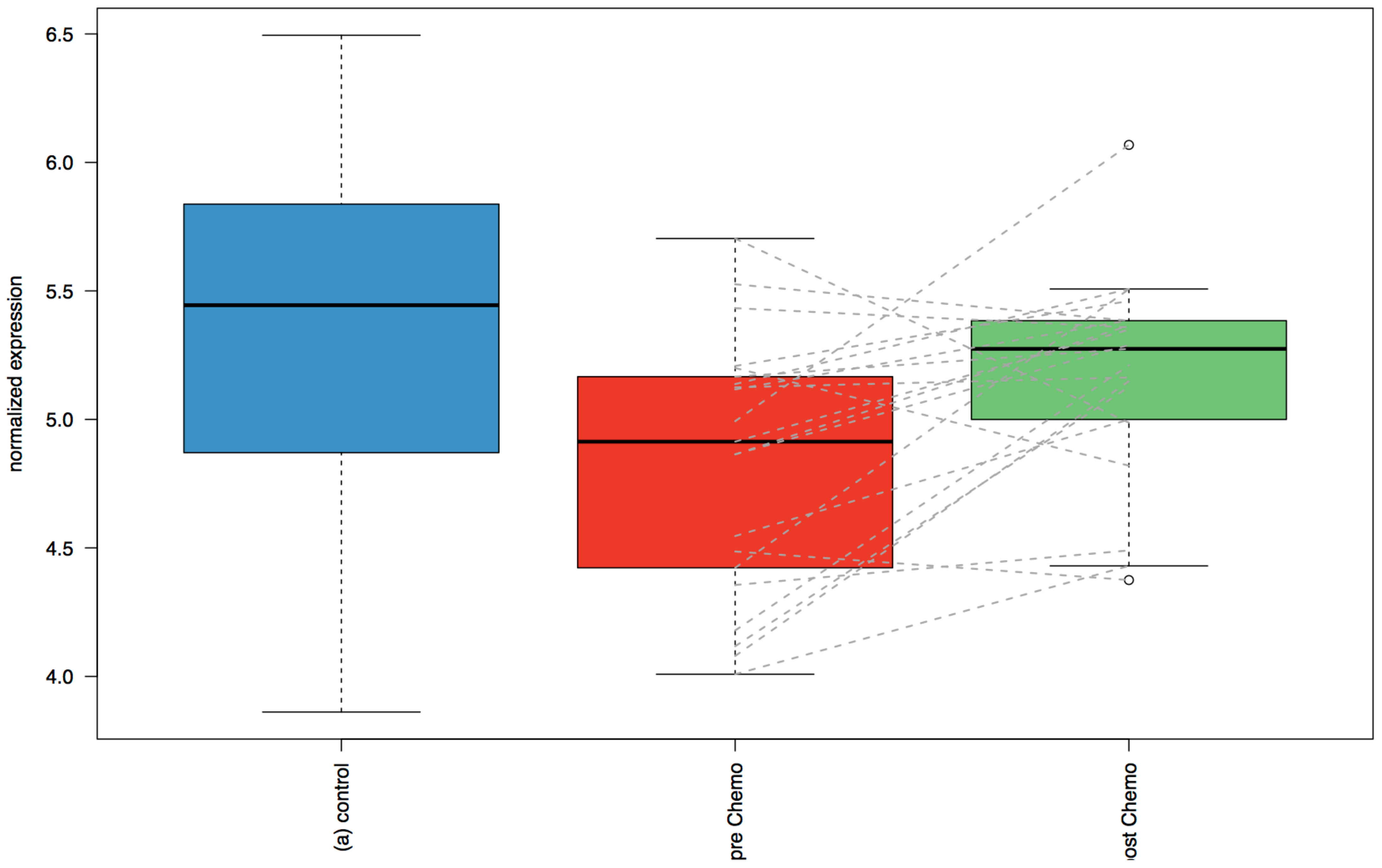
normalized expression
6.5
6.0
5.5
5.0
4.5
4.0
(a) control
pre Chemo
ost Chemo

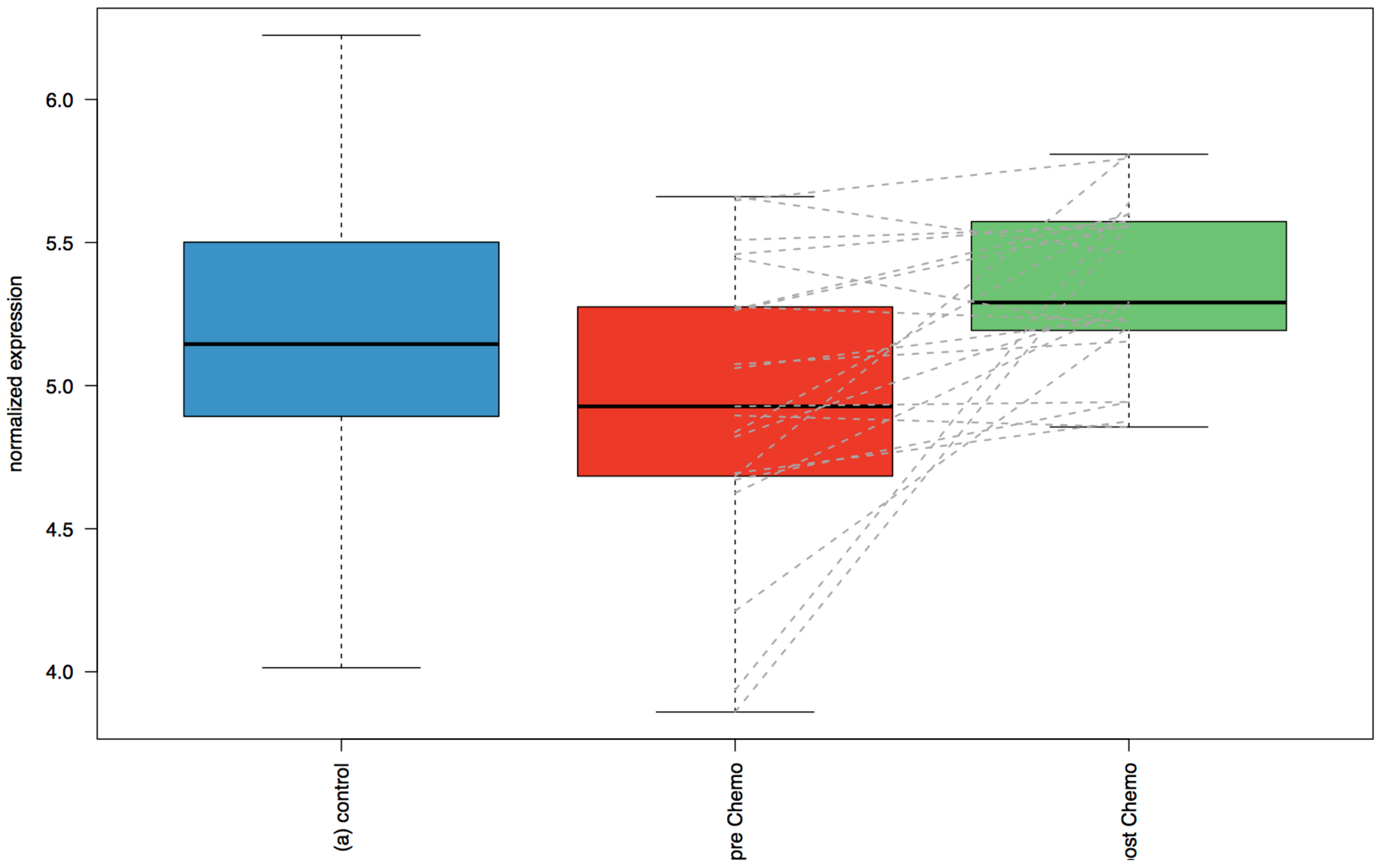
normalized expression
6.0
5.5
5.0
4.5
4.0
(a) control
pre Chemo
ost Chemo

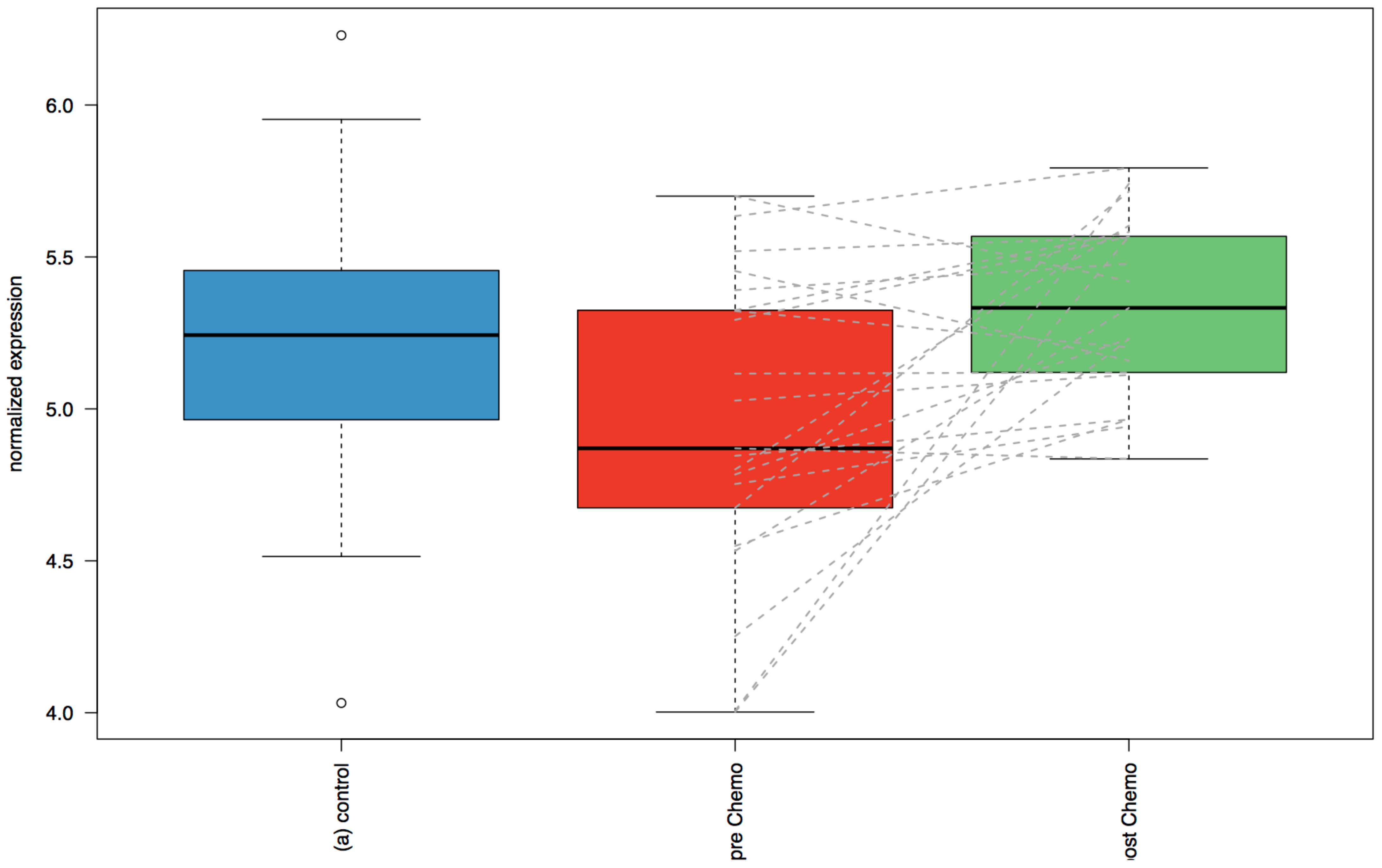
normalized expression
6.0
5.5
5.0
4.5
4.0
(a) control
pre Chemo
ost Chemo

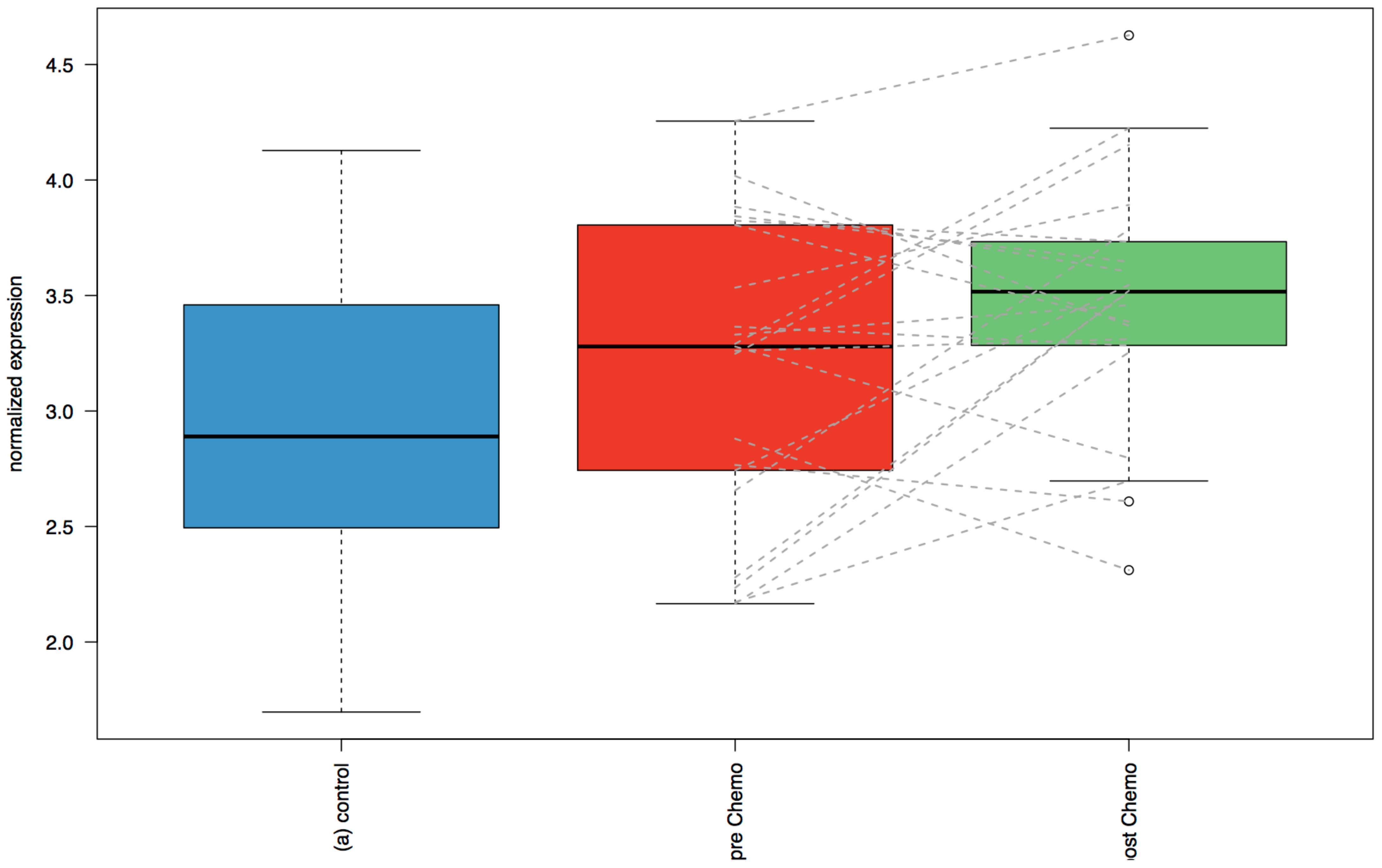
normalized expression
4.5
4.0
3.5
3.0
2.5
2.0
(a) control
pre Chemo
ost Chemo

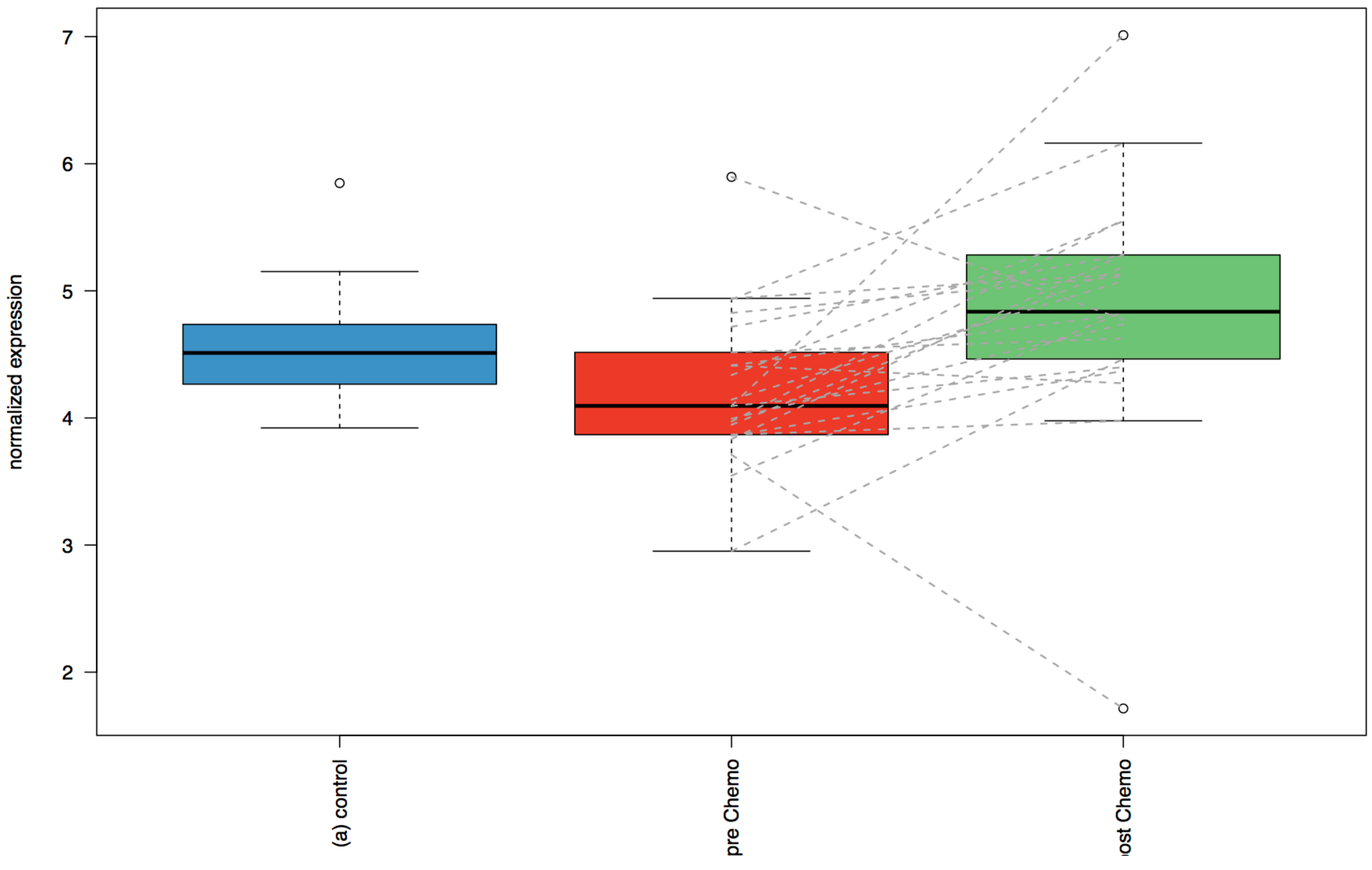
normalized expression
7
6
5
4
3
2
(a) control
pre Chemo
ost Chemo

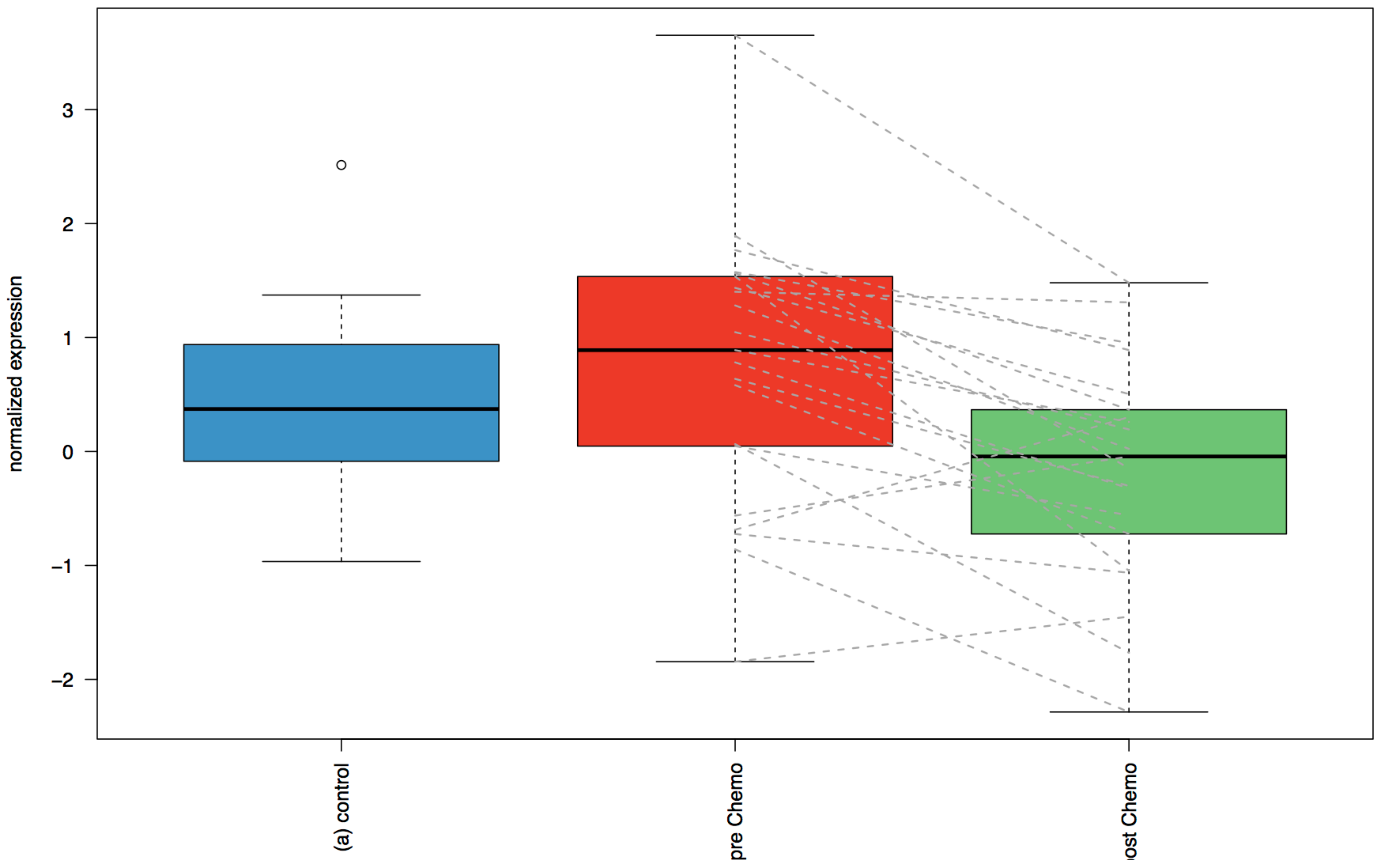
normalized expression
3
2
1
0
−1
−2
(a) control
pre Chemo
ost Chemo

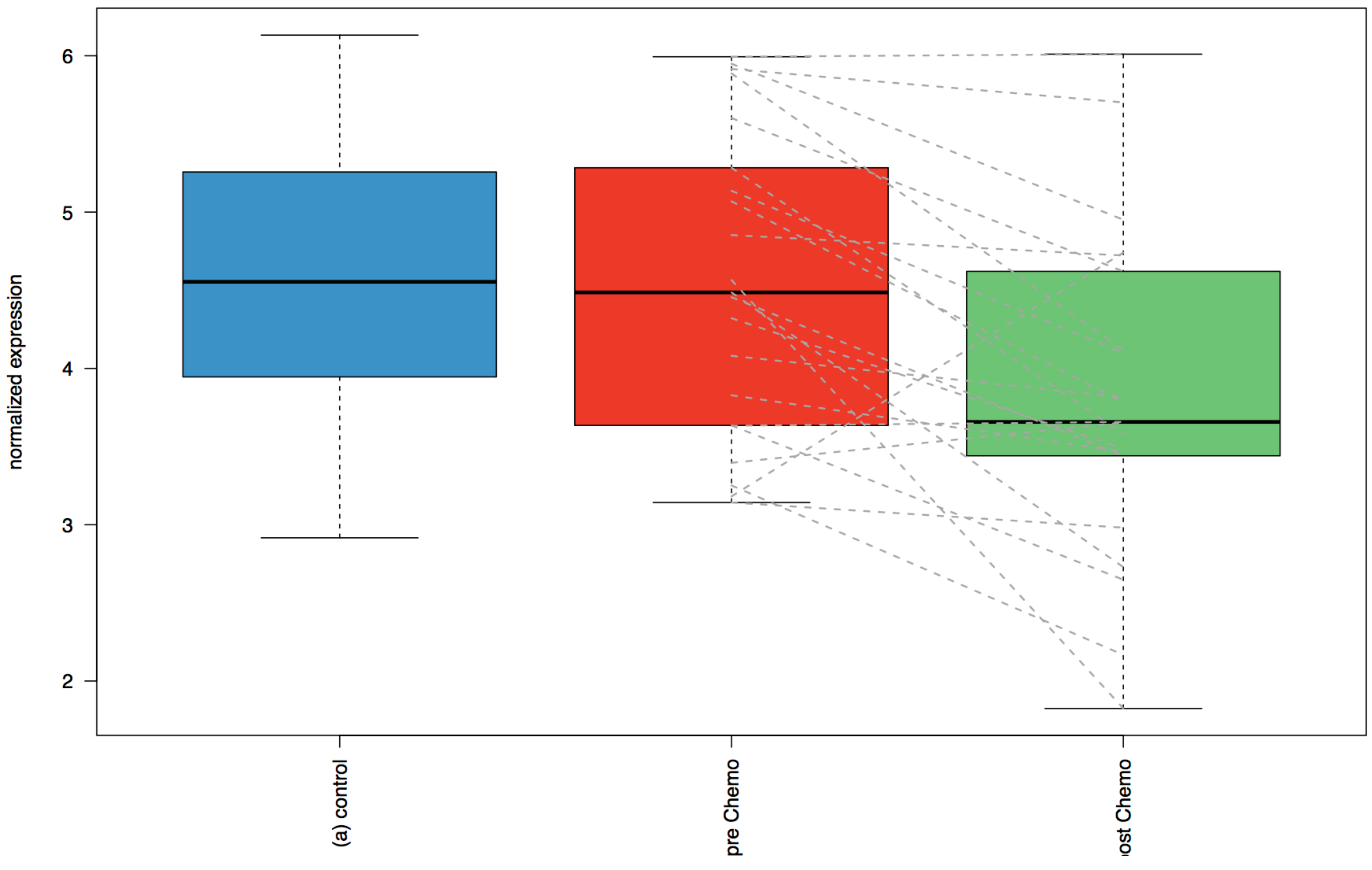
normalized expression
6
5
4
3
2
(a) control
pre Chemo
ost Chemo

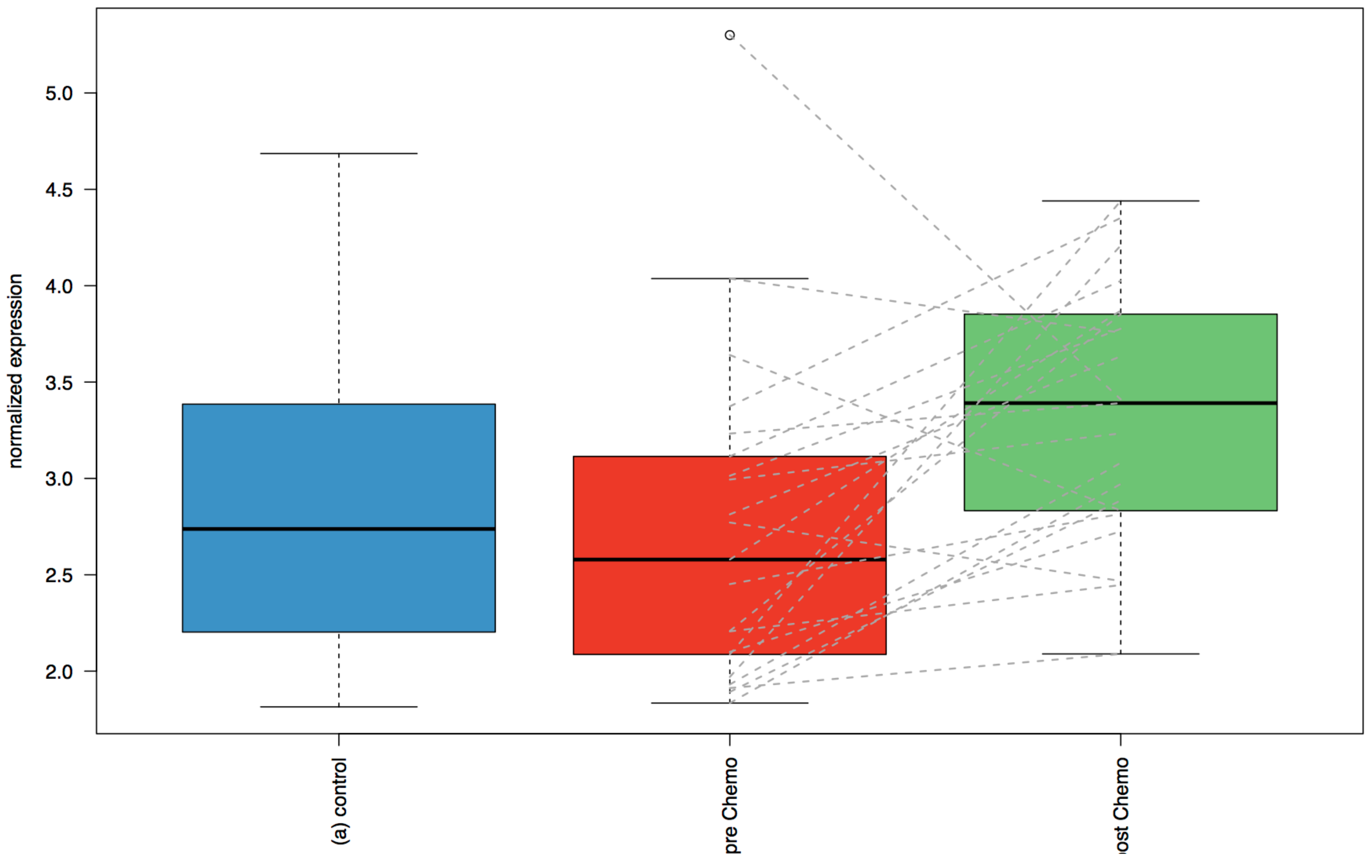

normalized expression
5.0
4.5
4.0
3.5
3.0
2.5
2.0
(a) control
pre Chemo
ost Chemo

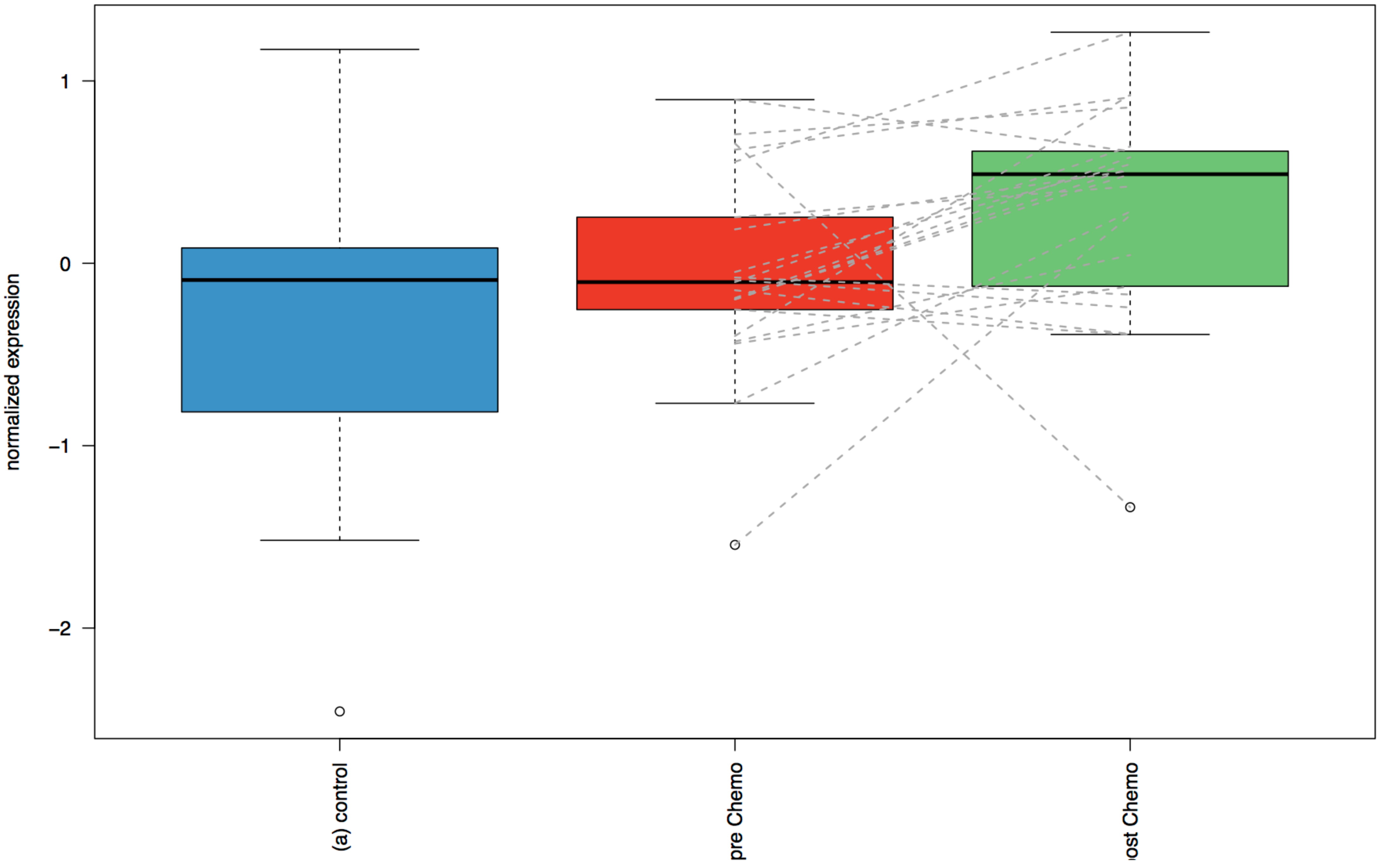
normalized expression
1
0
−1
−2
(a) control
pre Chemo
ost Chemo

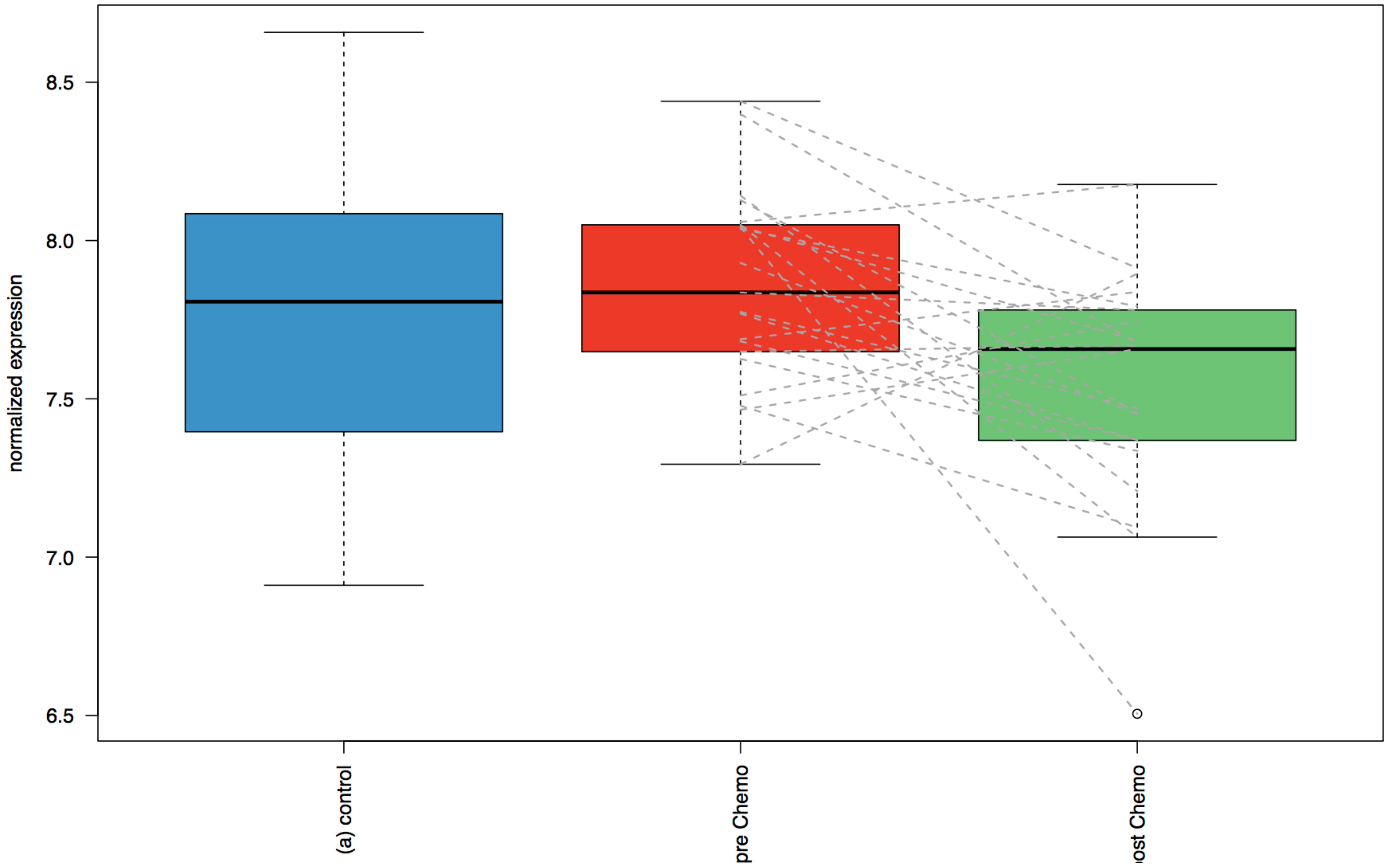
normalized expression
8.5
8.0
7.5
7.0
6.5
(a) control
pre Chemo
ost Chemo

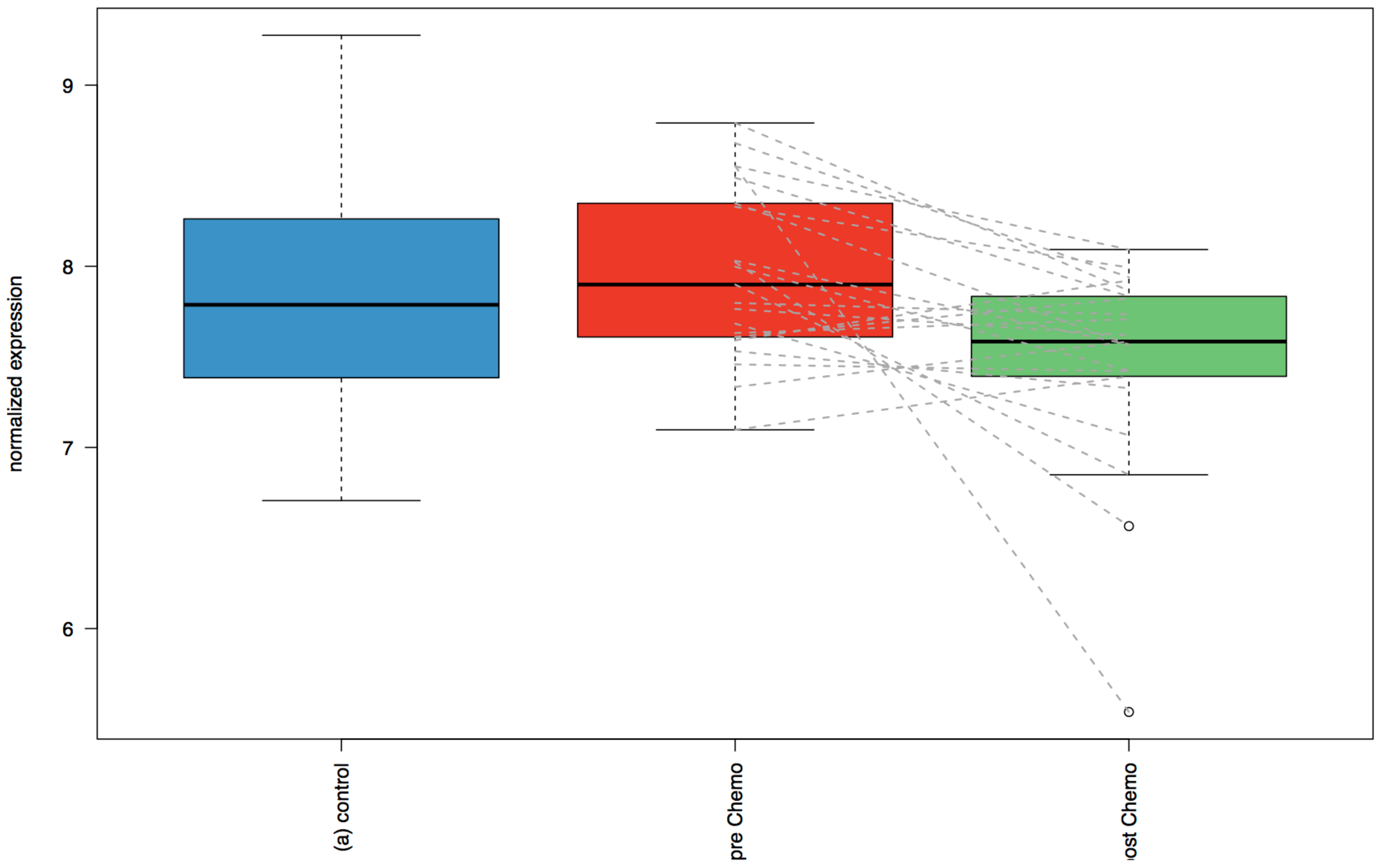
normalized expression
9
8
7
6
(a) control
pre Chemo
ost Chemo

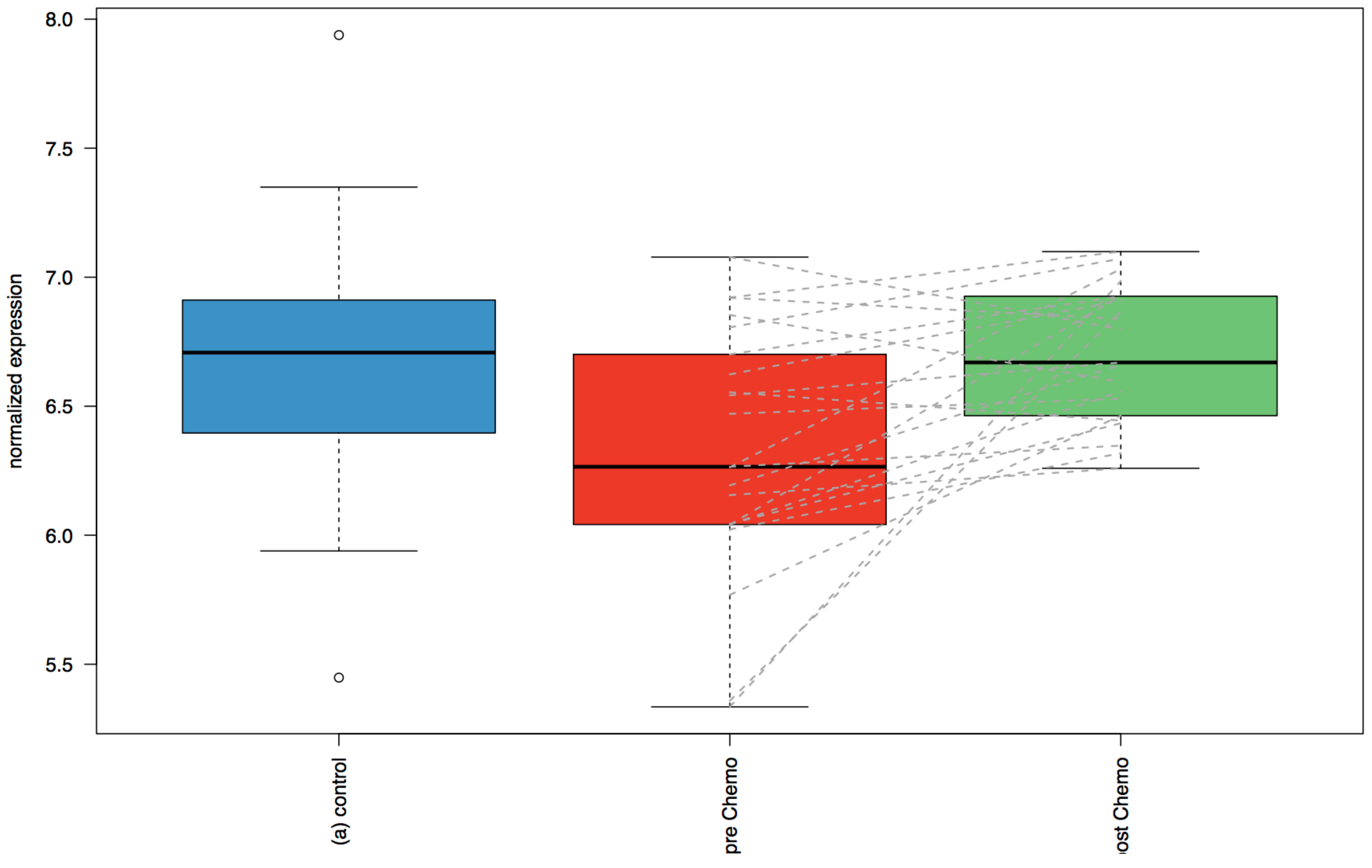

8.0
7.5
7.0
6.5
6.0
5.5
normalized expression
(a) control
pre Chemo
ost Chemo

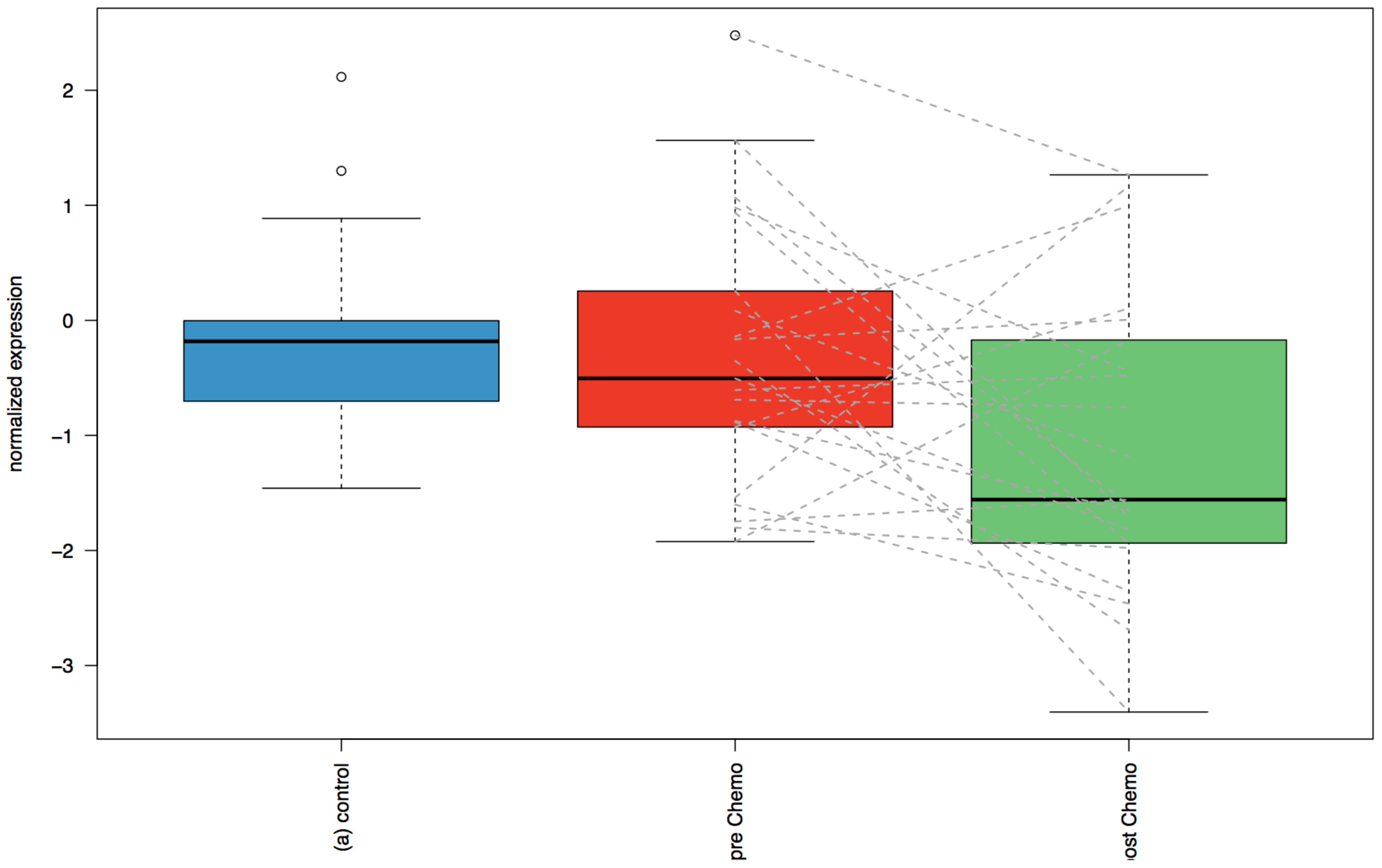
normalized expression
2
1
0
−1
−2
−3
(a) control
pre Chemo
ost Chemo

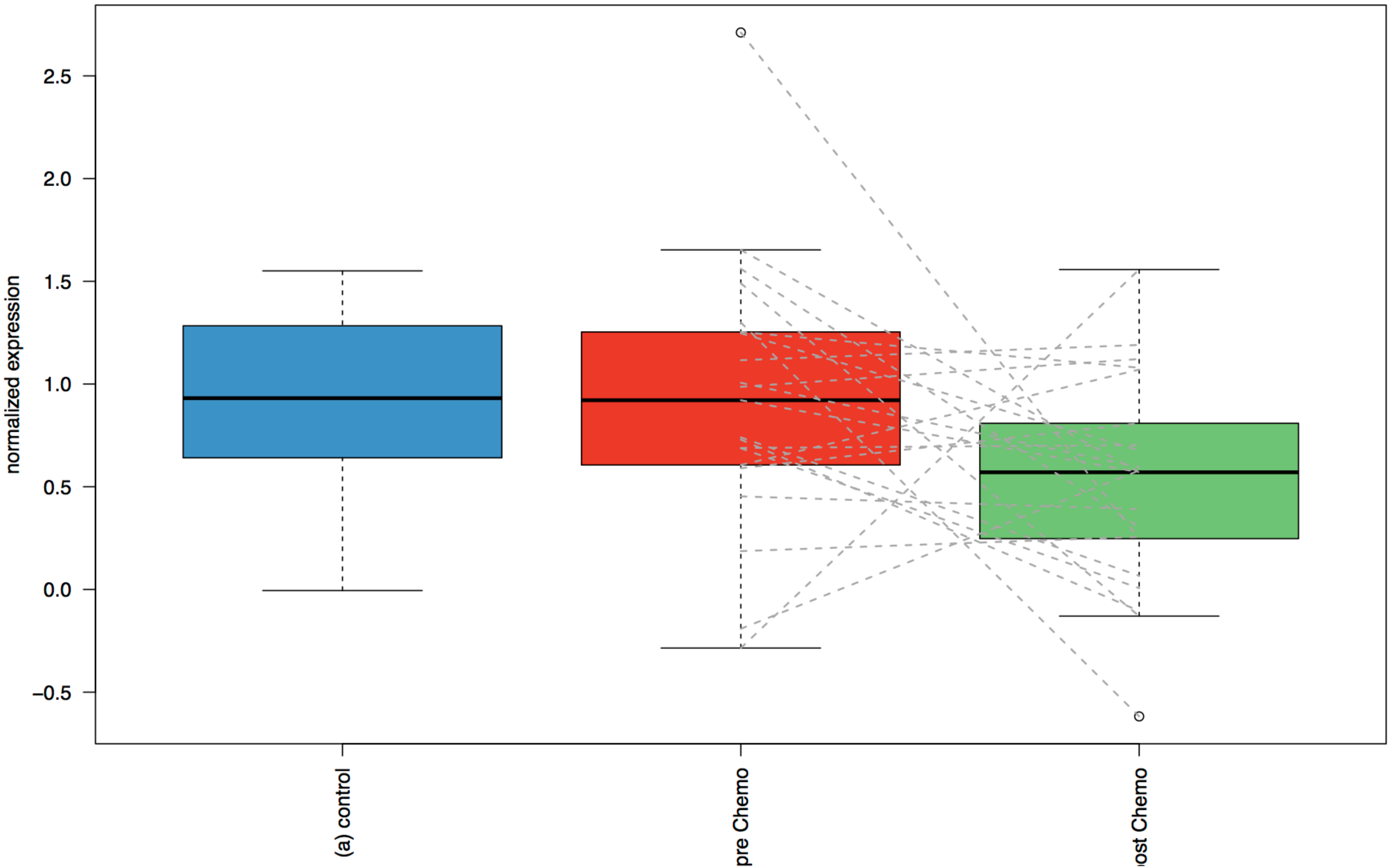
normalized expression
2.5
2.0
1.5
1.0
0.5
0.0
−0.5
(a) control
pre Chemo
ost Chemo

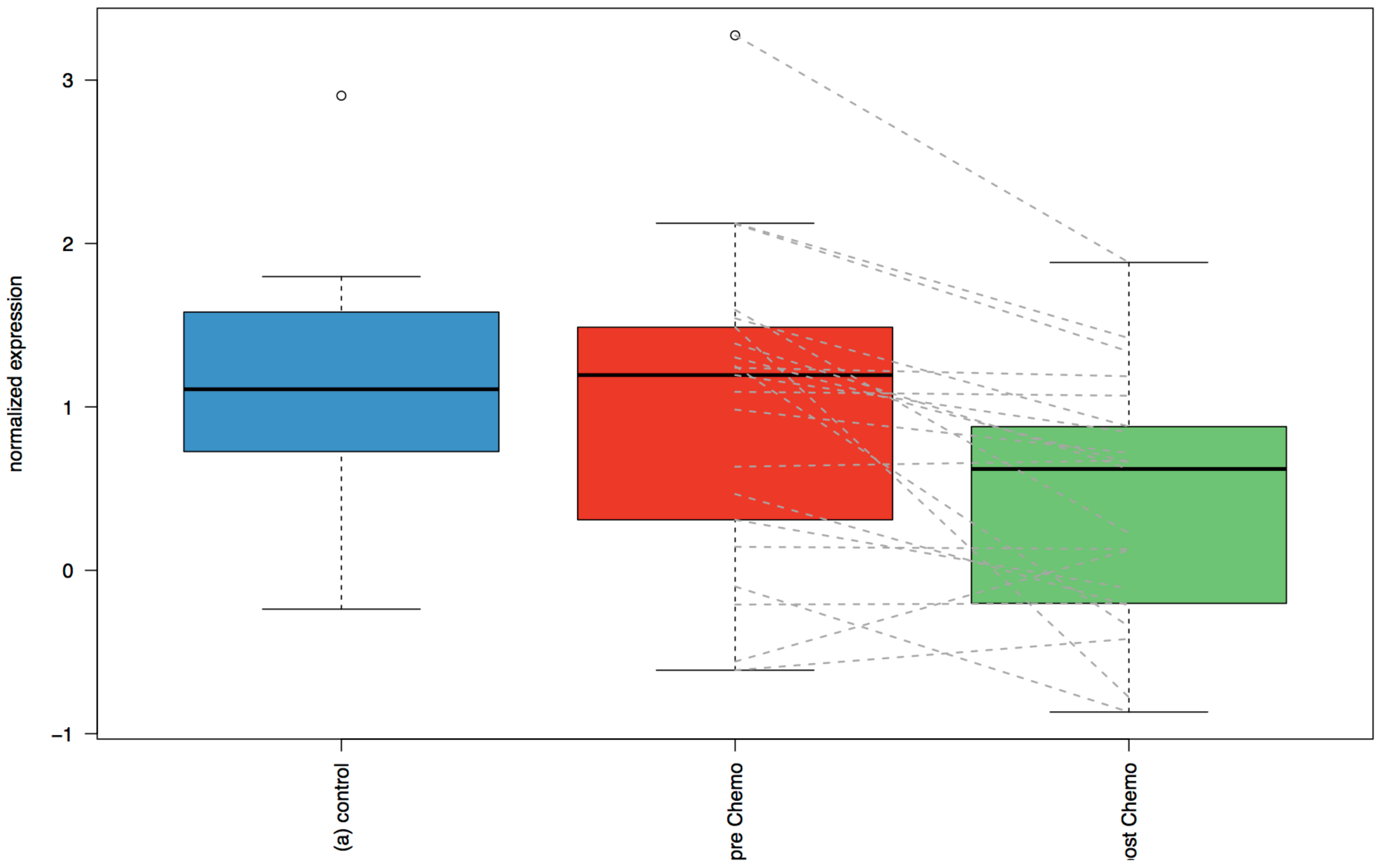
normalized expression
3
2
1
0
−1
(a) control
pre Chemo
ost Chemo

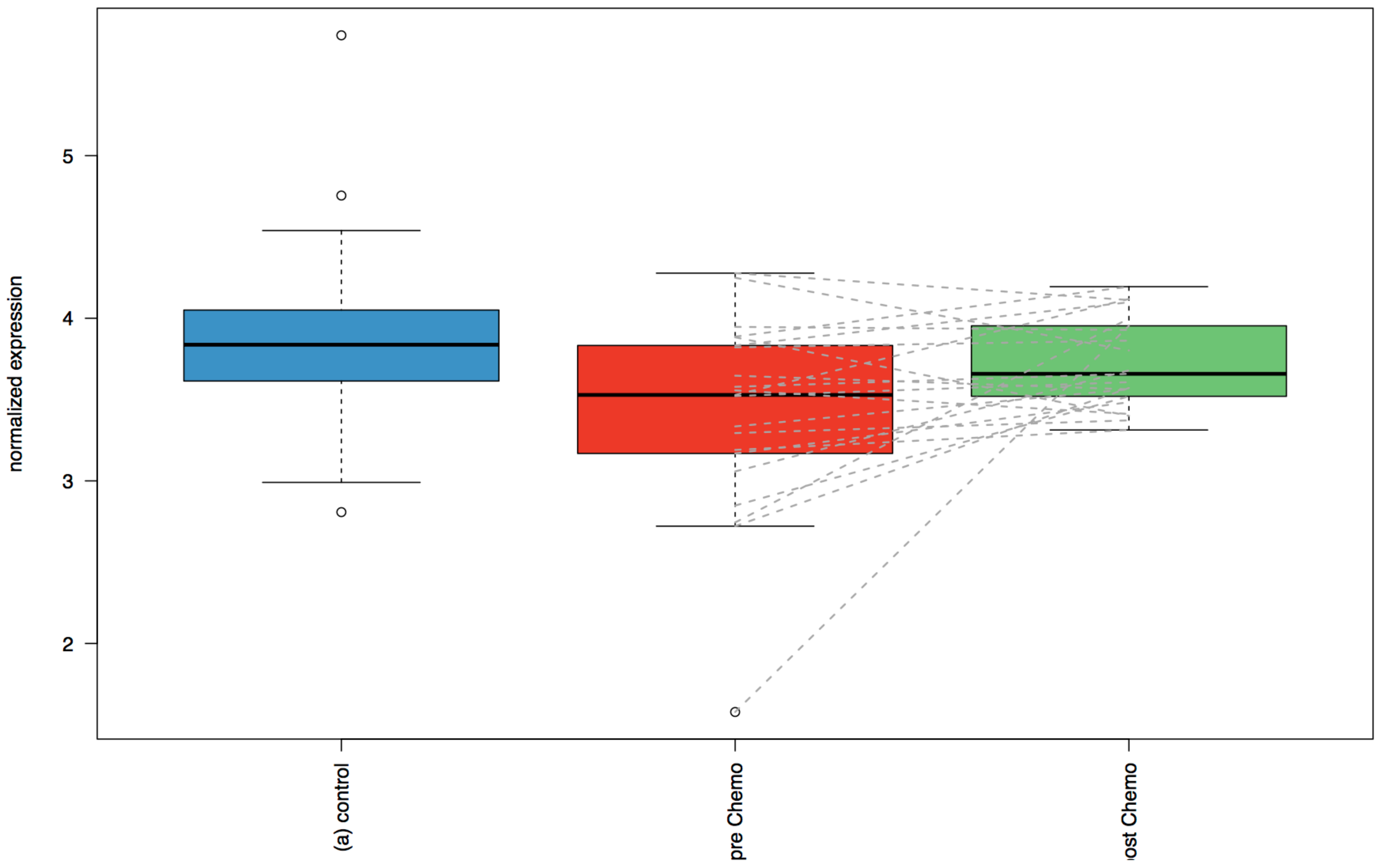
normalized expression
5
4
3
2
(a) control
pre Chemo
ost Chemo

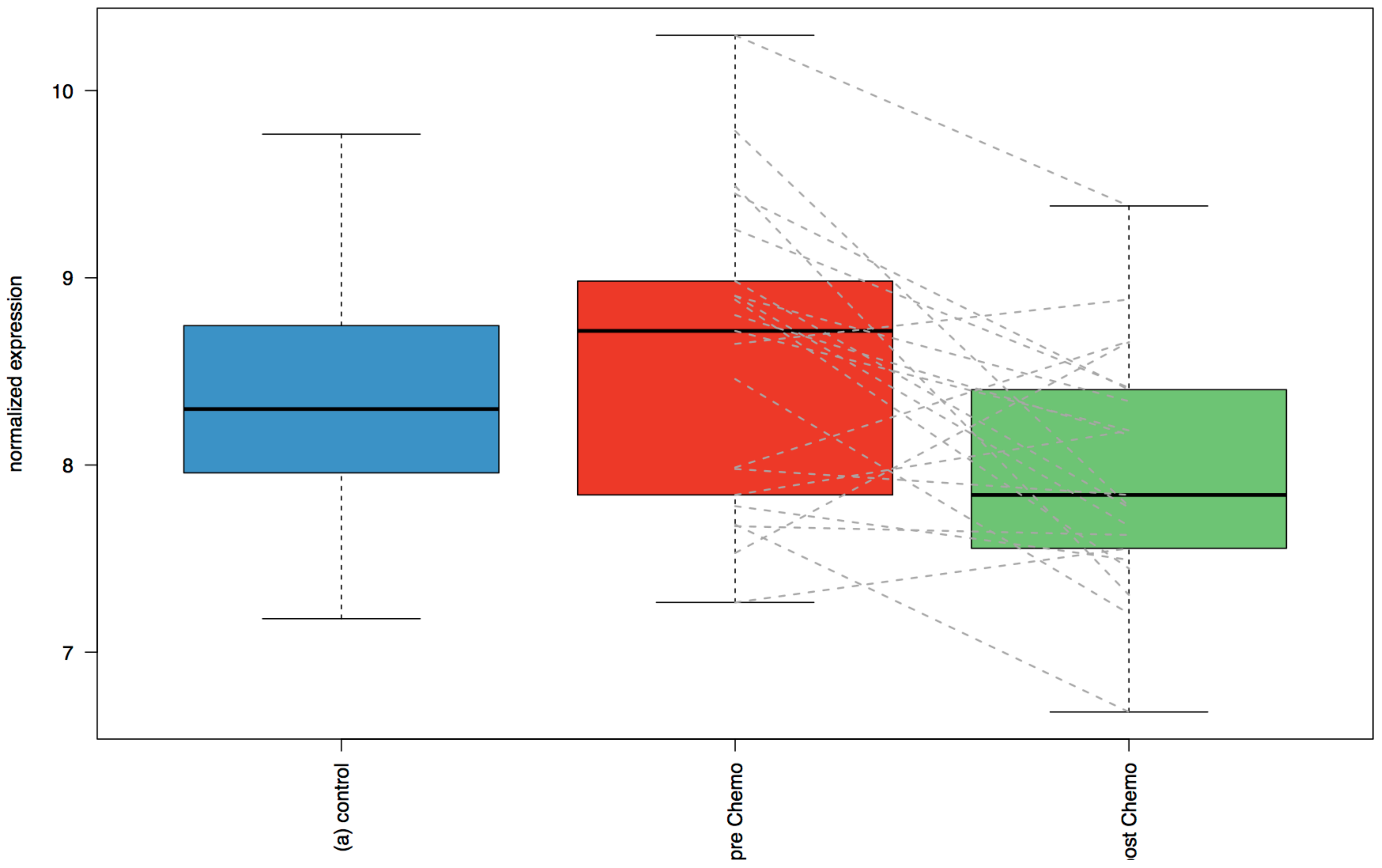
normalized expression
10
9
8
7
(a) control
pre Chemo
ost Chemo

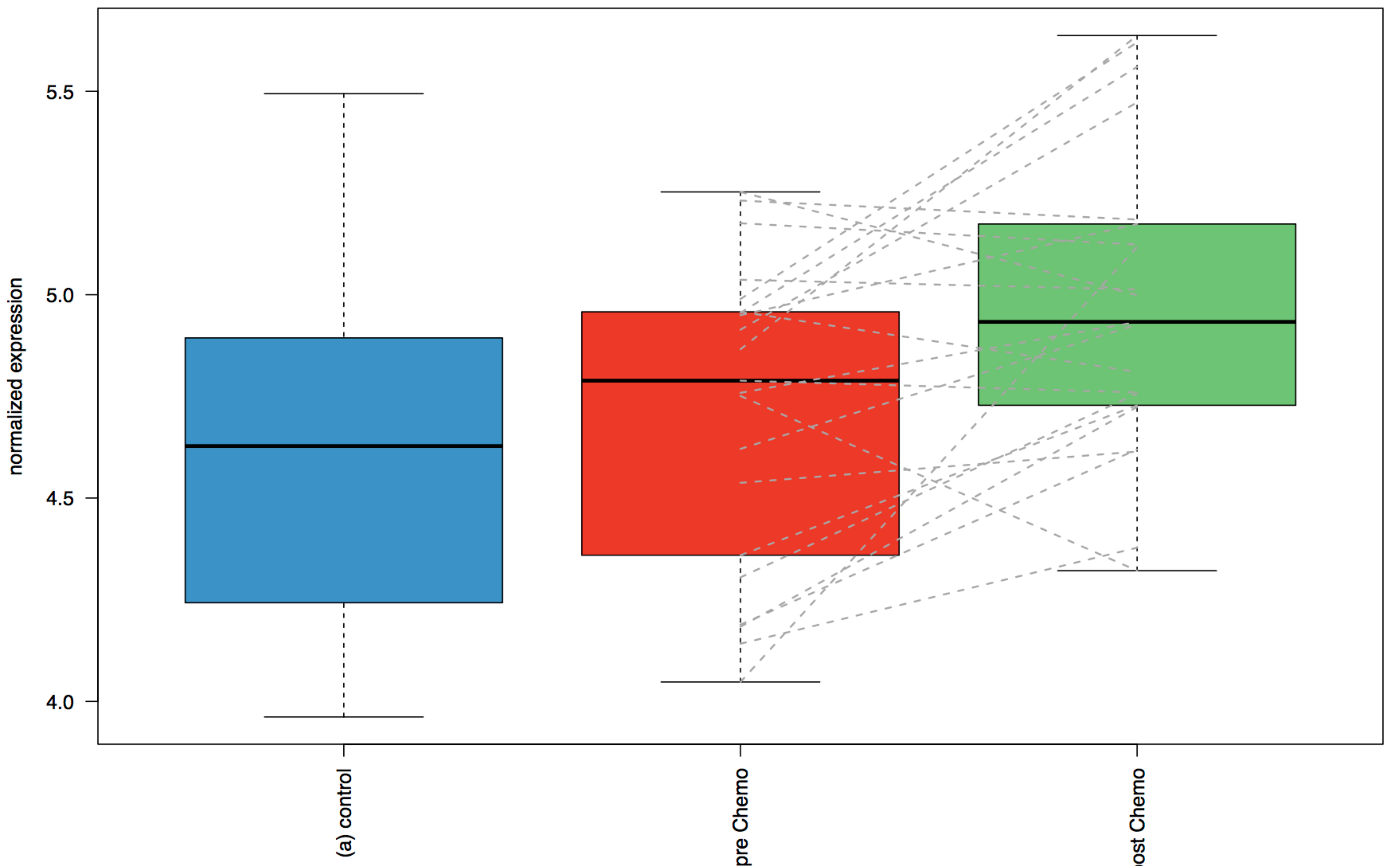
normalized expression
5.5
5.0
4.5
4.0
(a) control
pre Chemo
ost Chemo

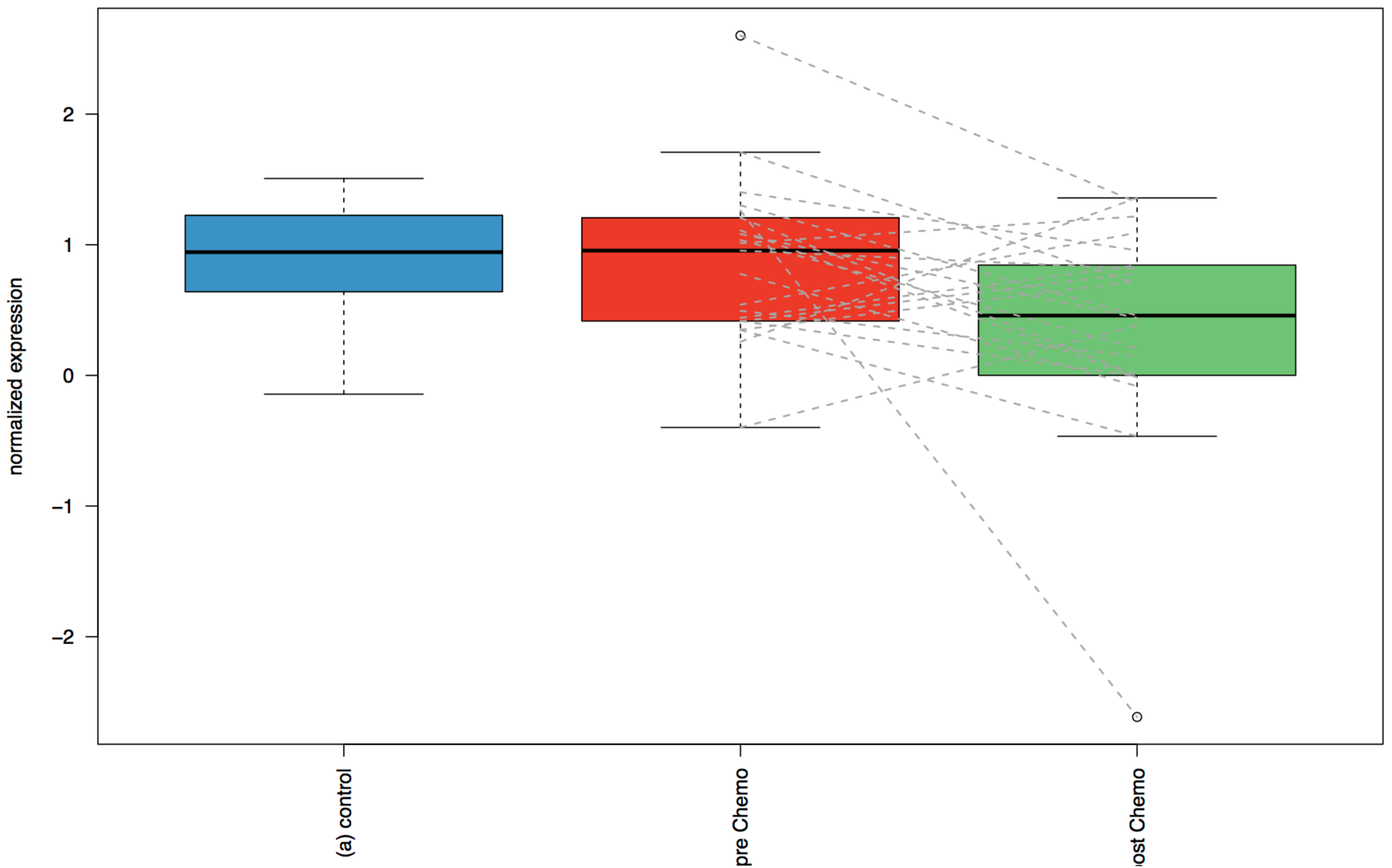
normalized expression
2
1
0
−1
−2
(a) control
pre Chemo
ost Chemo

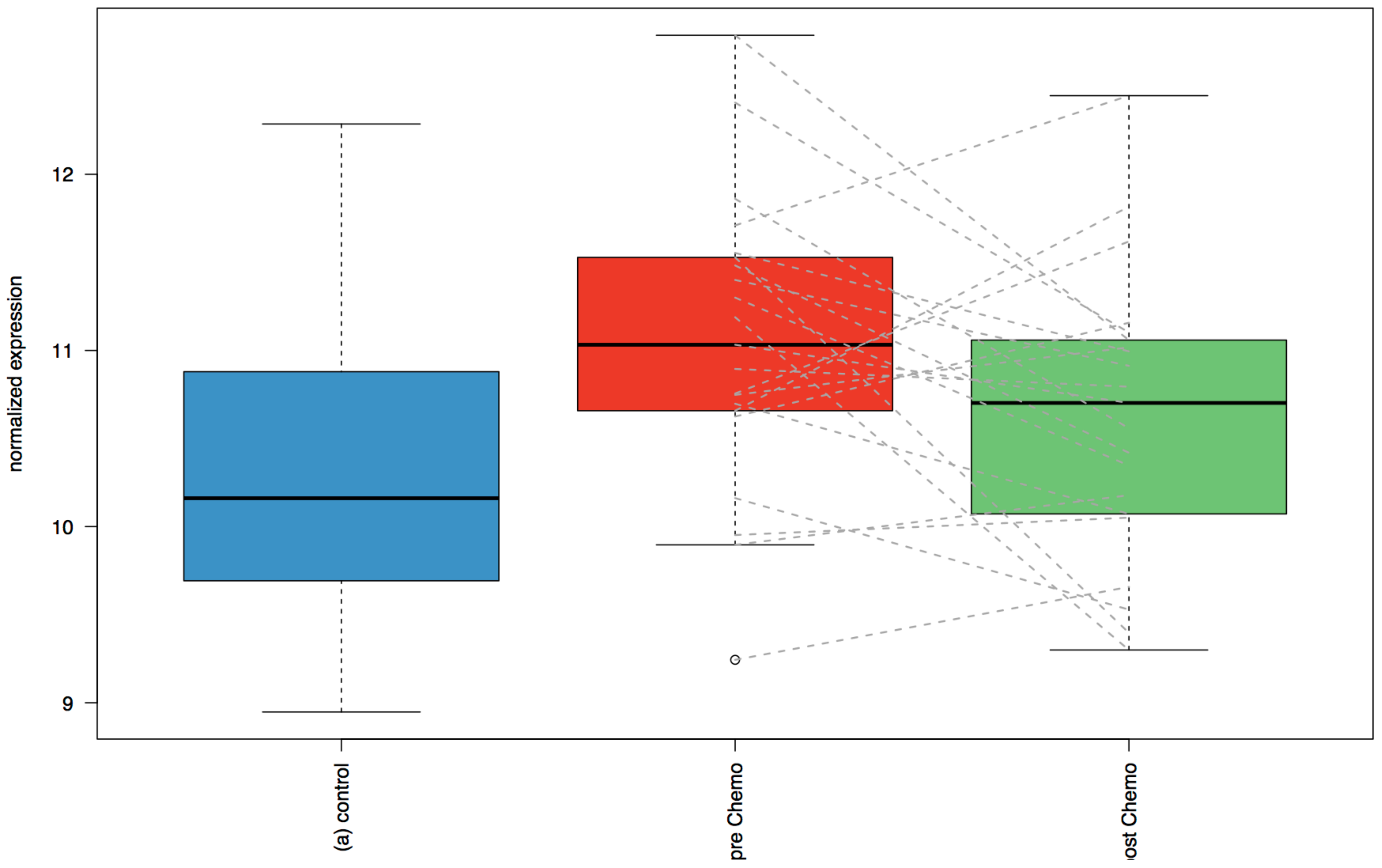
normalized expression
12
11
10
9
(a) control
pre Chemo
ost Chemo

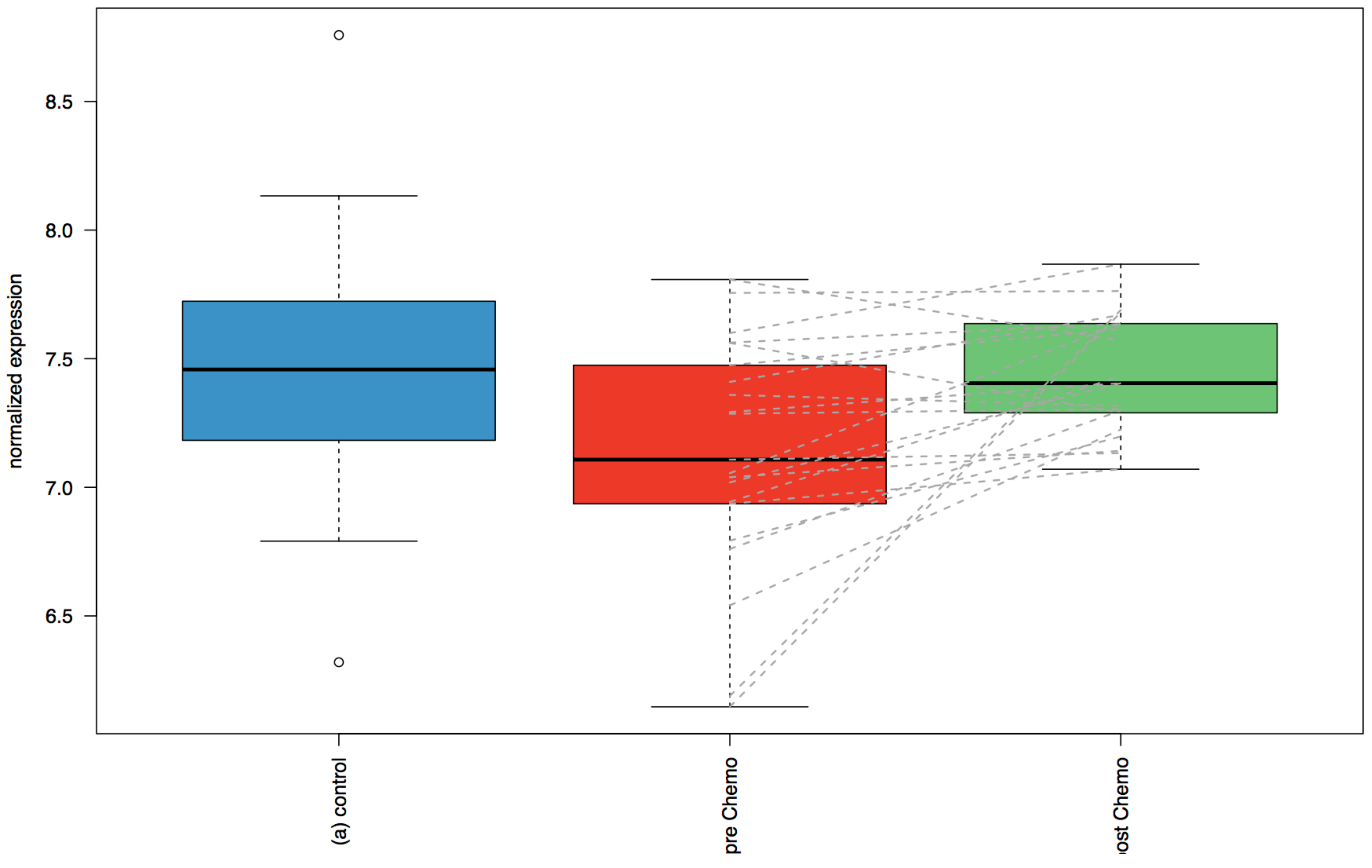
normalized expression
8.5
8.0
7.5
7.0
6.5
(a) control
pre Chemo
ost Chemo

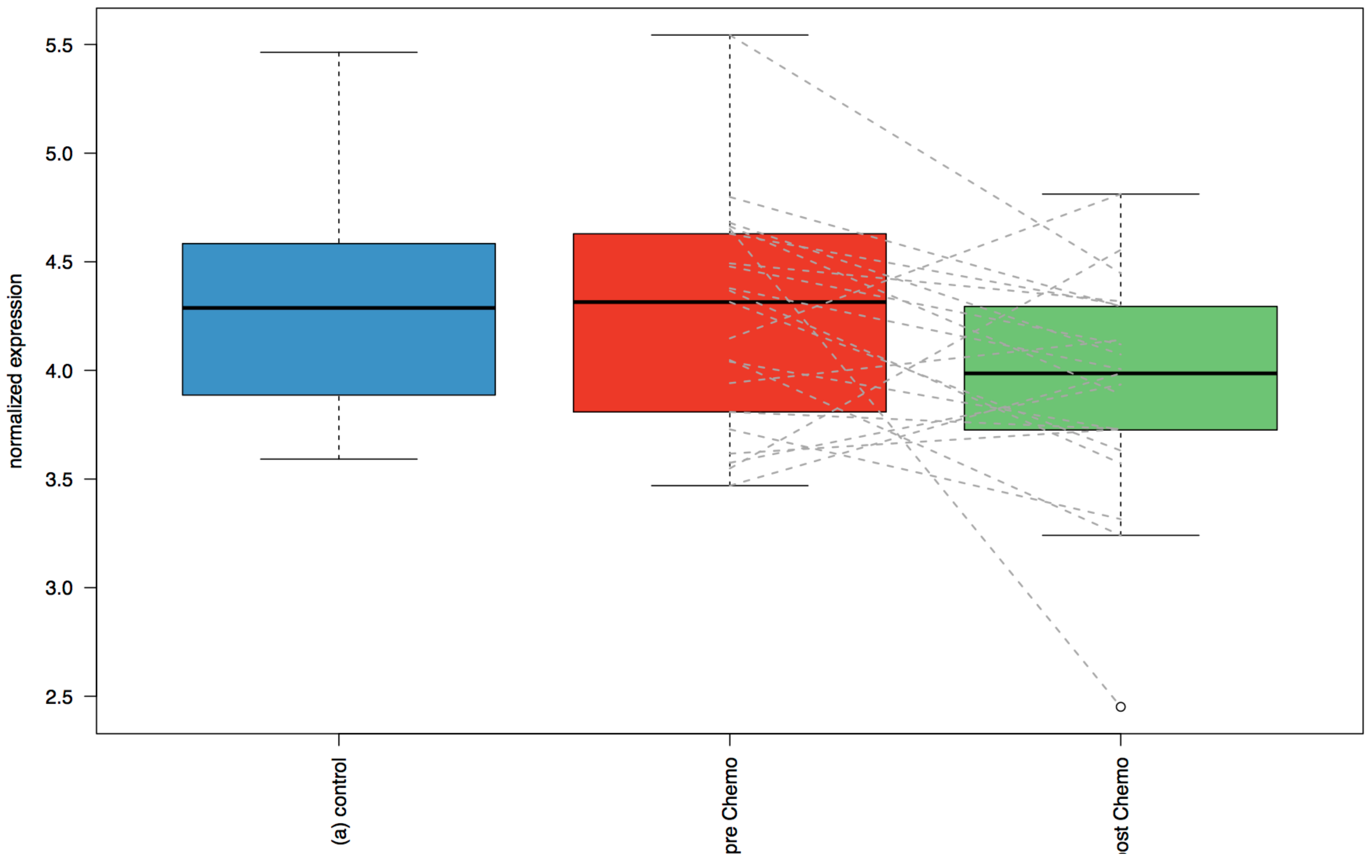
normalized expression
5.5
5.0
4.5
4.0
3.5
3.0
2.5
(a) control
pre Chemo
ost Chemo

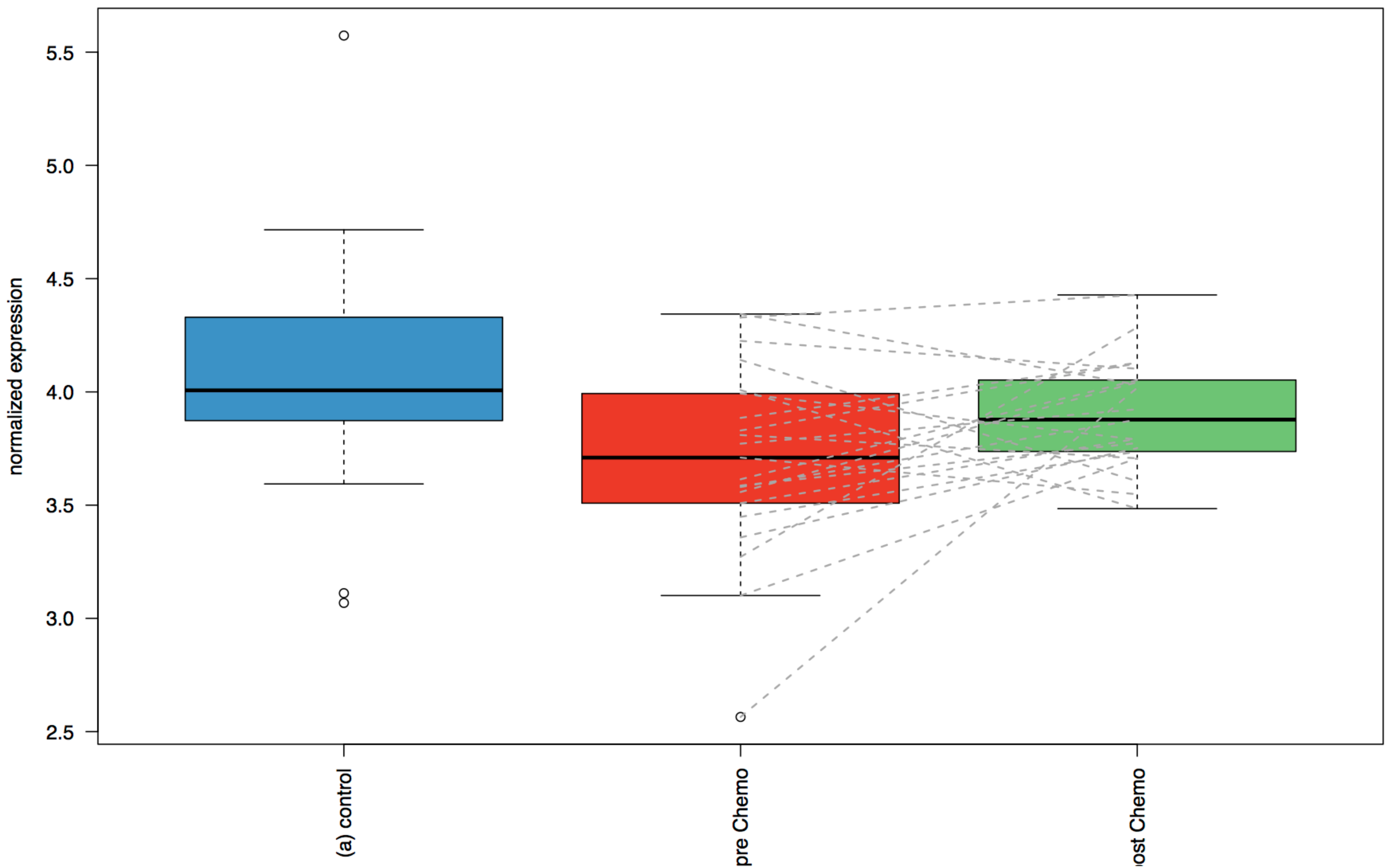
normalized expression
5.5
5.0
4.5
4.0
3.5
3.0
2.5
(a) control
pre Chemo
ost Chemo

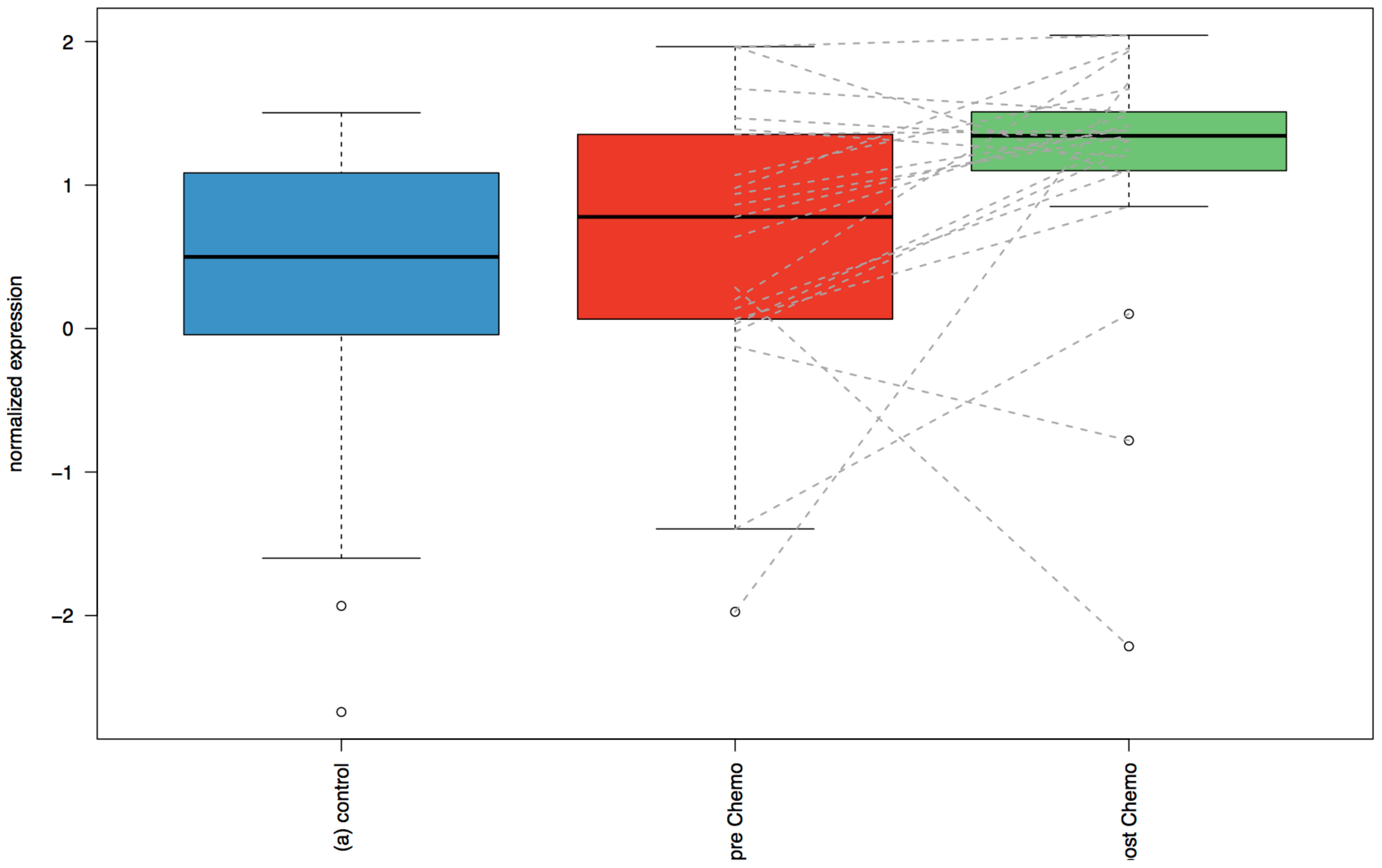
normalized expression
2
1
0
−1
−2
(a) control
pre Chemo
ost Chemo

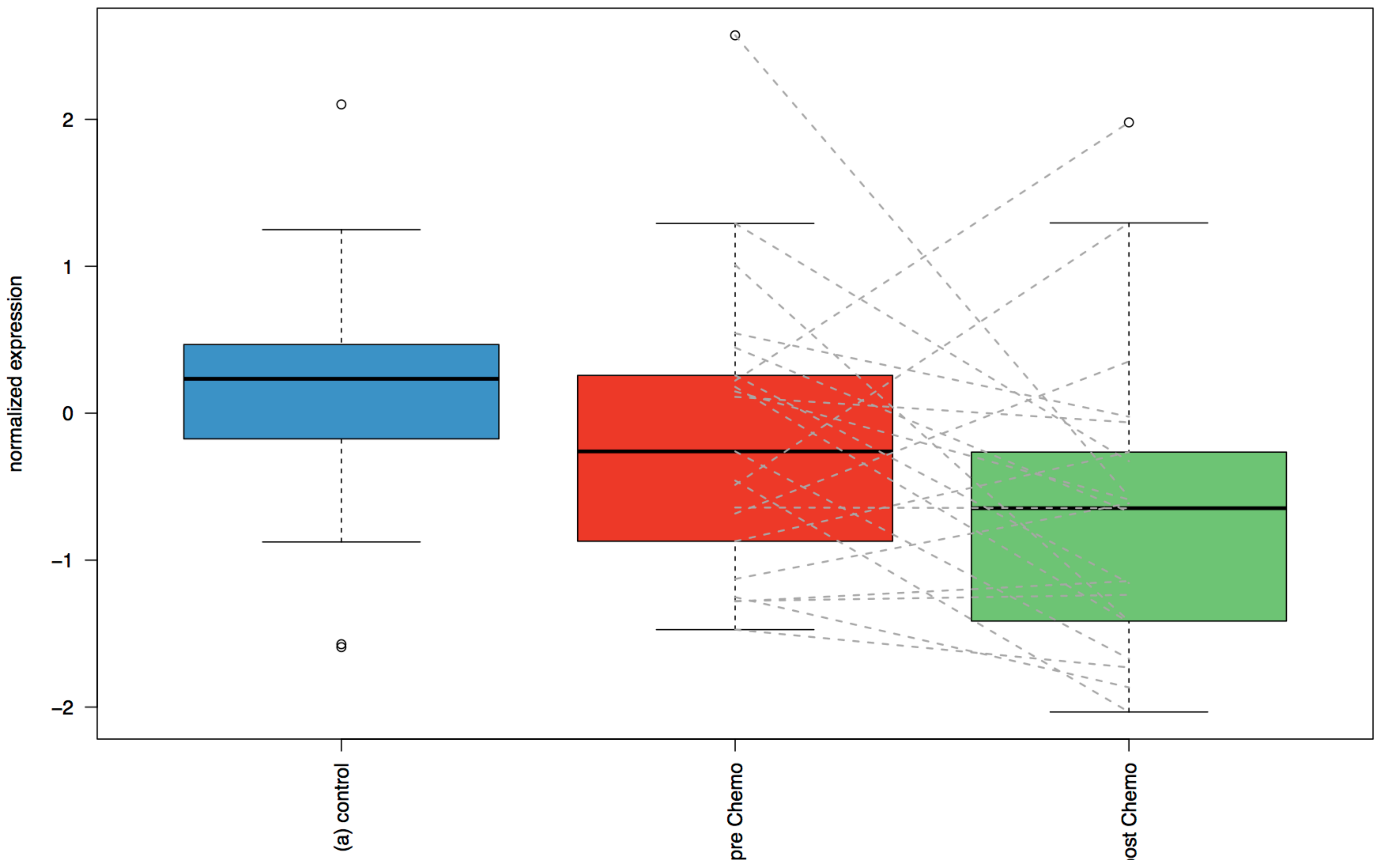
normalized expression
2
1
0
−1
−2
(a) control
pre Chemo
ost Chemo

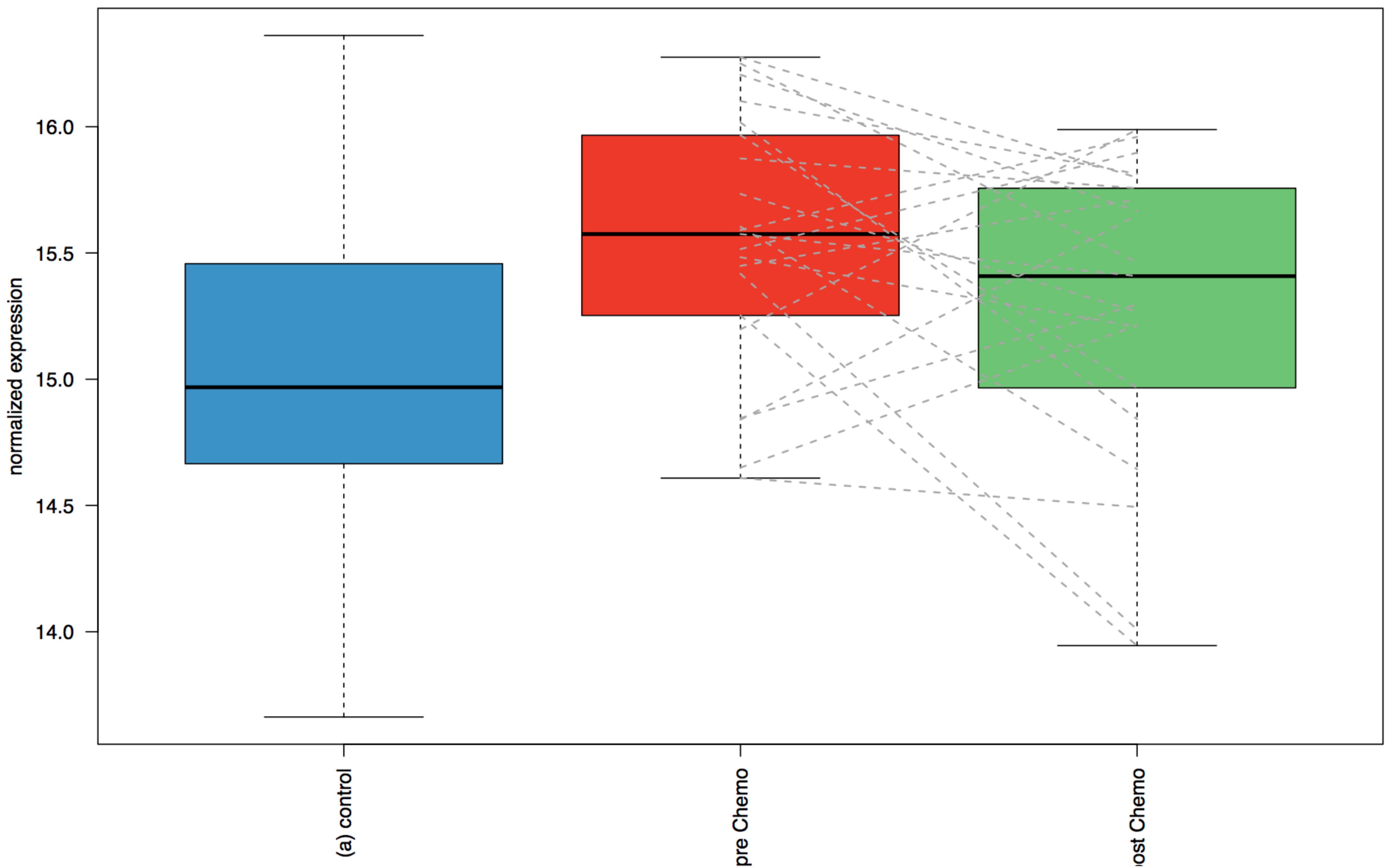
normalized expression
16.0
15.5
15.0
14.5
14.0
(a) control
pre Chemo
ost Chemo

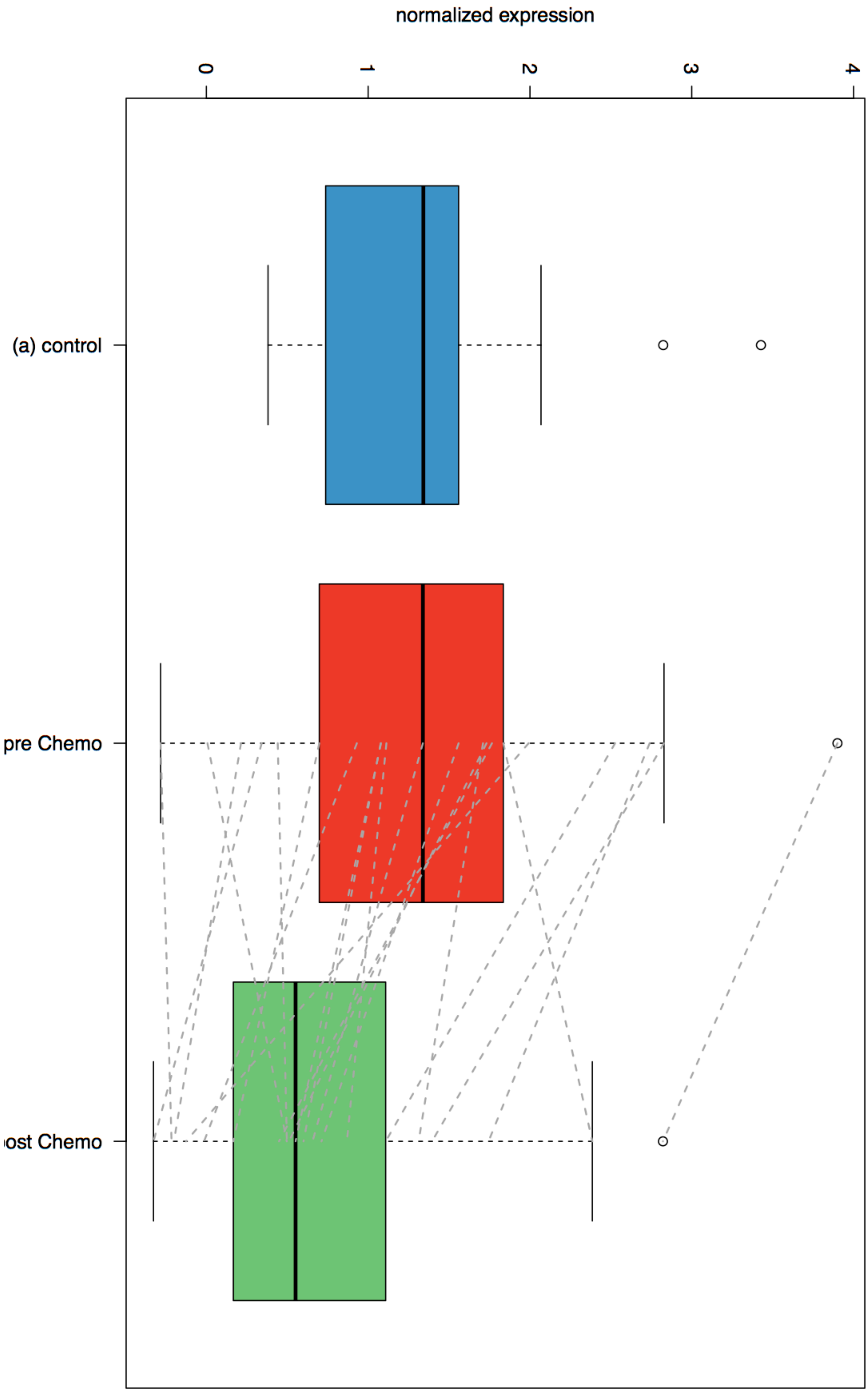
normalized expression
0
1
2
3
4
(a) control
pre Chemo
ost Chemo

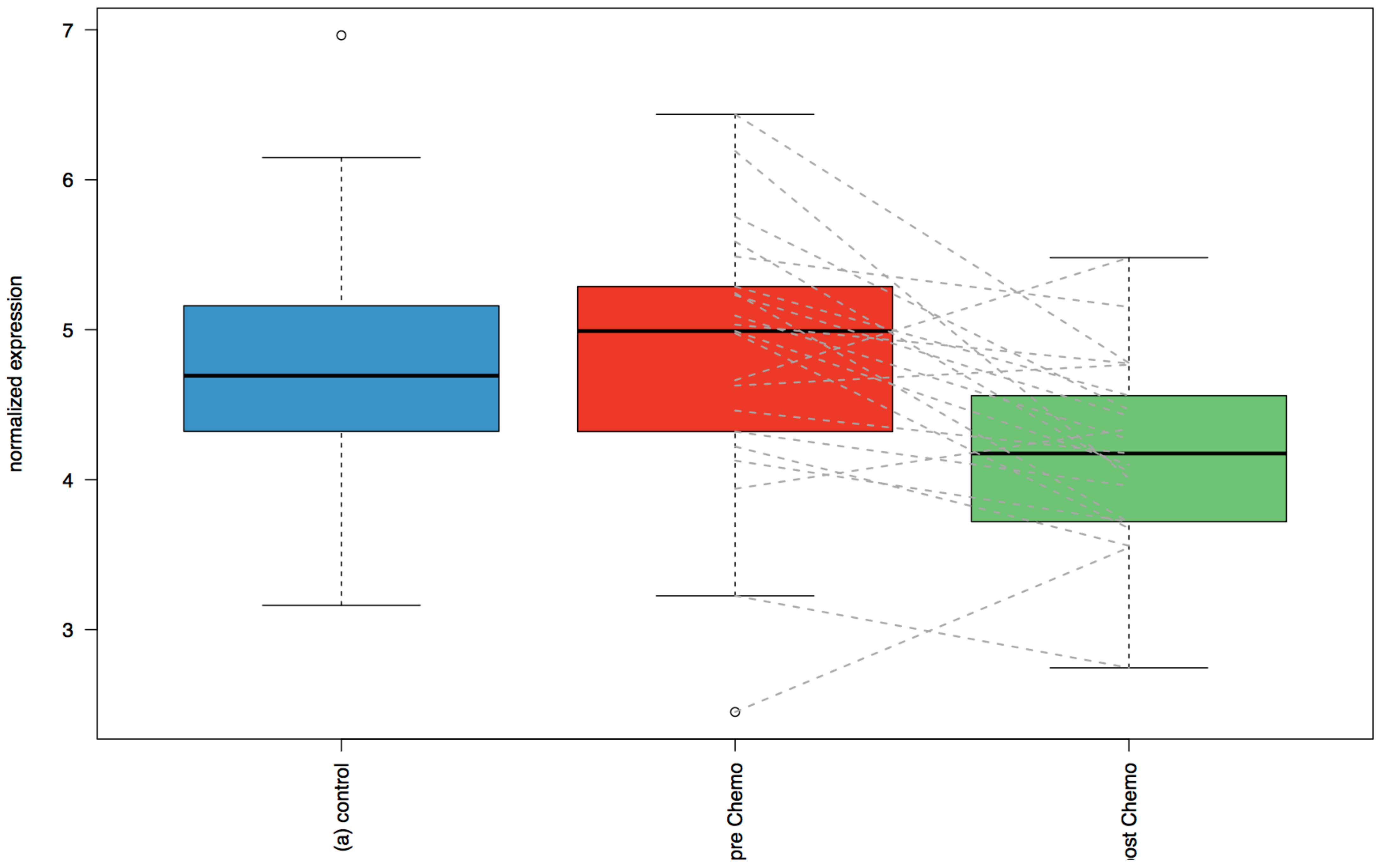

normalized expression
7
6
5
4
3
(a) control
pre Chemo
ost Chemo

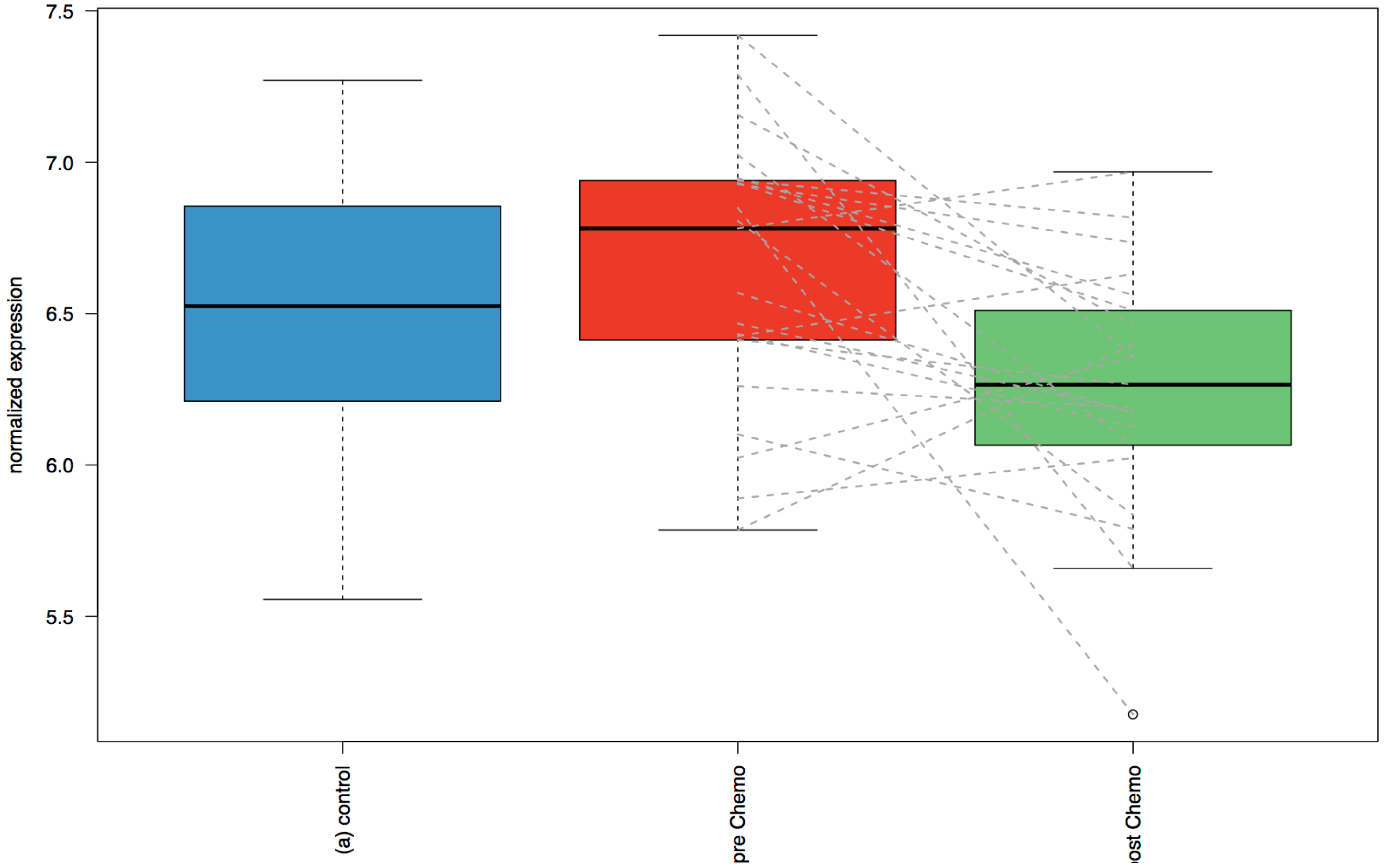
7.5
7.0
6.5
6.0
5.5
normalized expression
(a) control
pre Chemo
ost Chemo

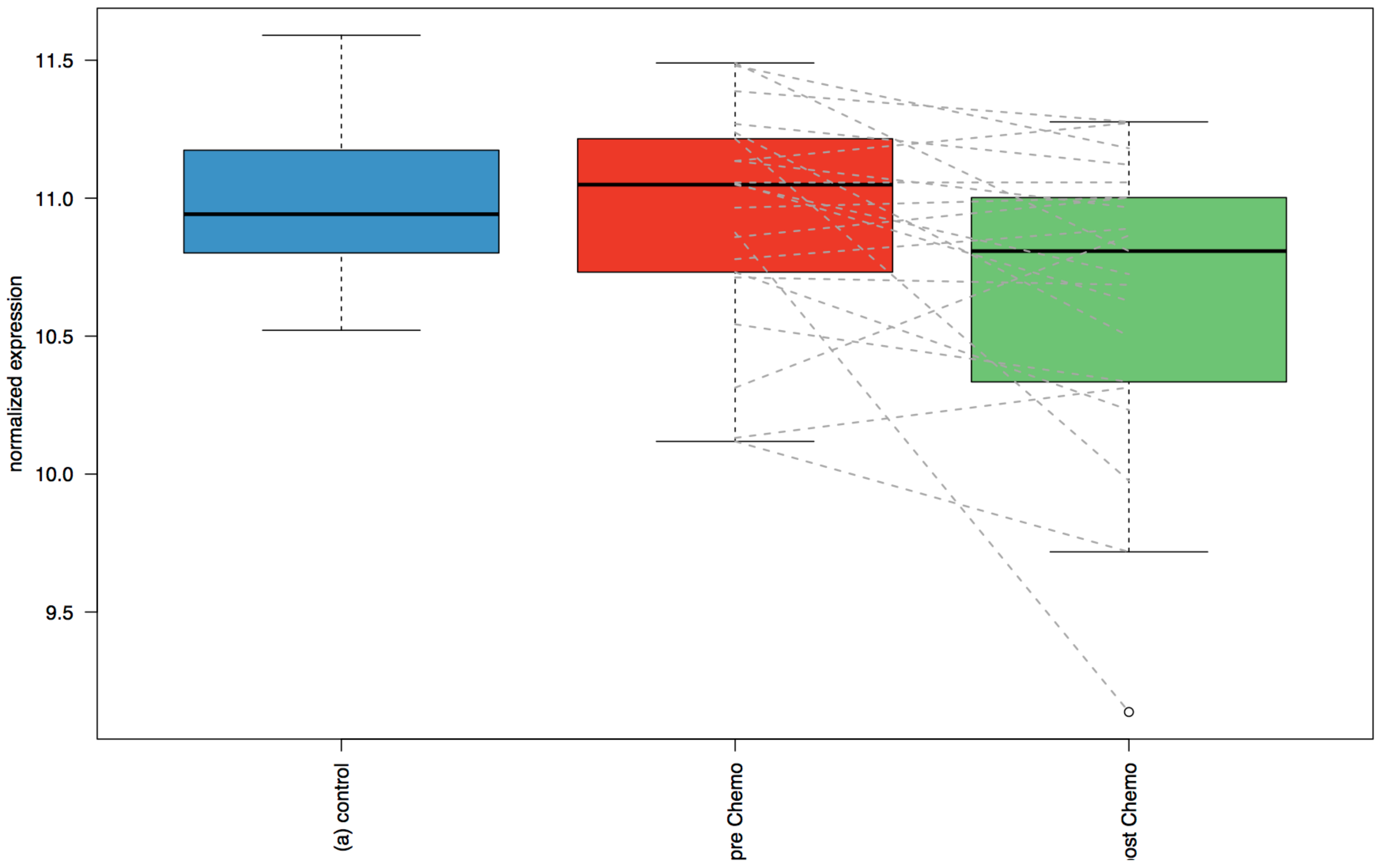

normalized expression
11.5
11.0
10.5
10.0
9.5
(a) control
pre Chemo
ost Chemo

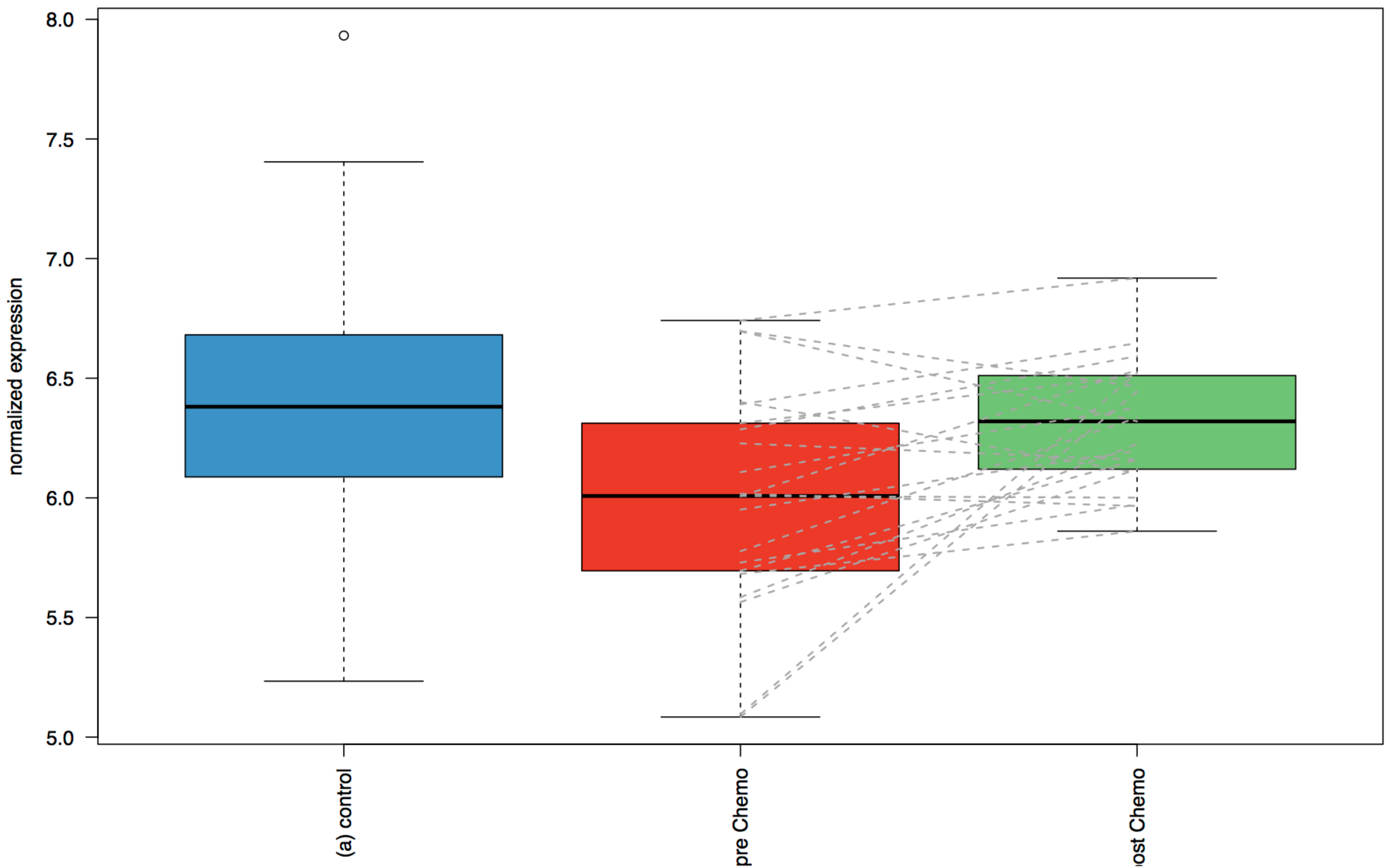
normalized expression
8.0
7.5
7.0
6.5
6.0
5.5
5.0
(a) control
pre Chemo
ost Chemo

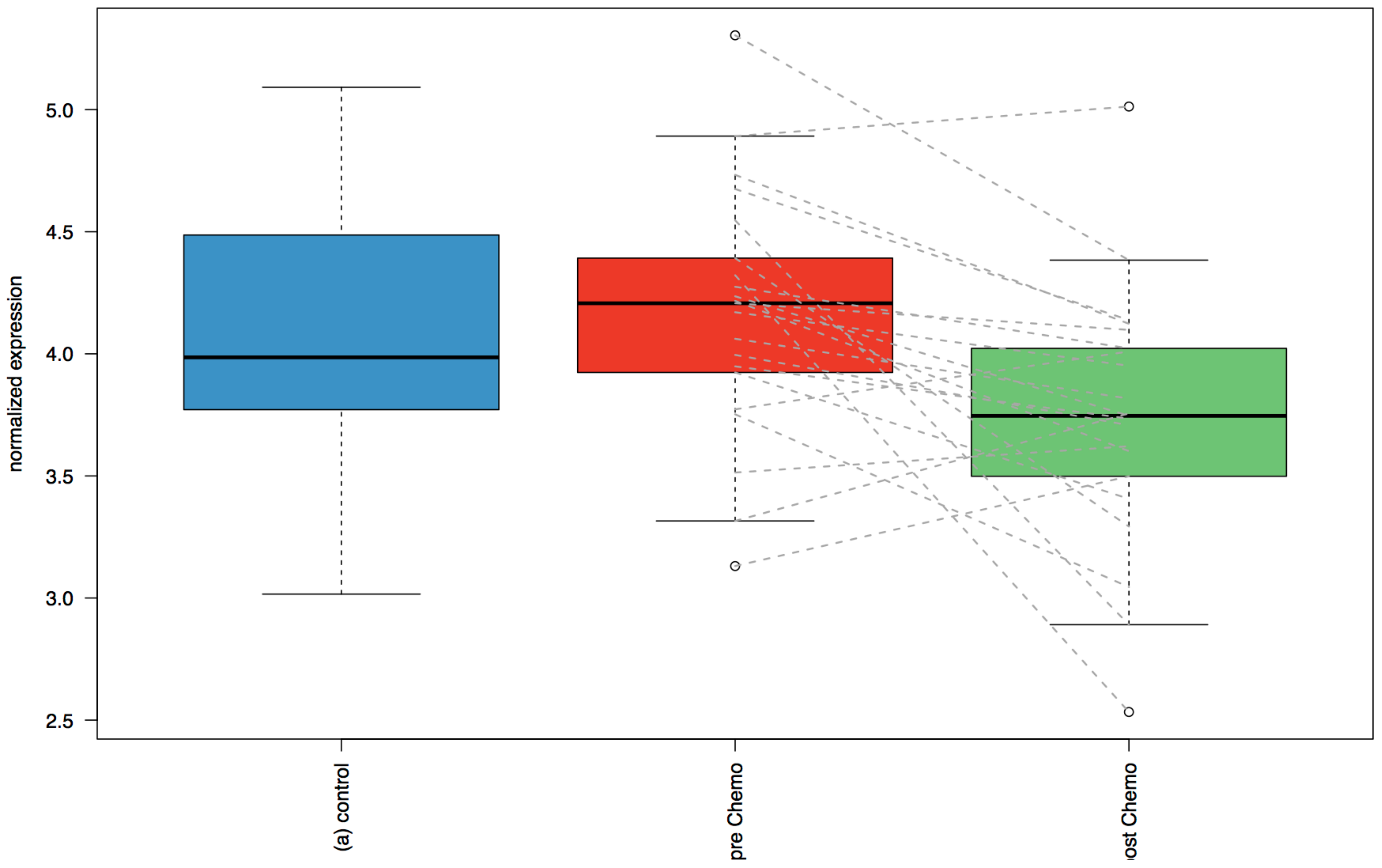
normalized expression
5.0
4.5
4.0
3.5
3.0
2.5
(a) control
pre Chemo
ost Chemo

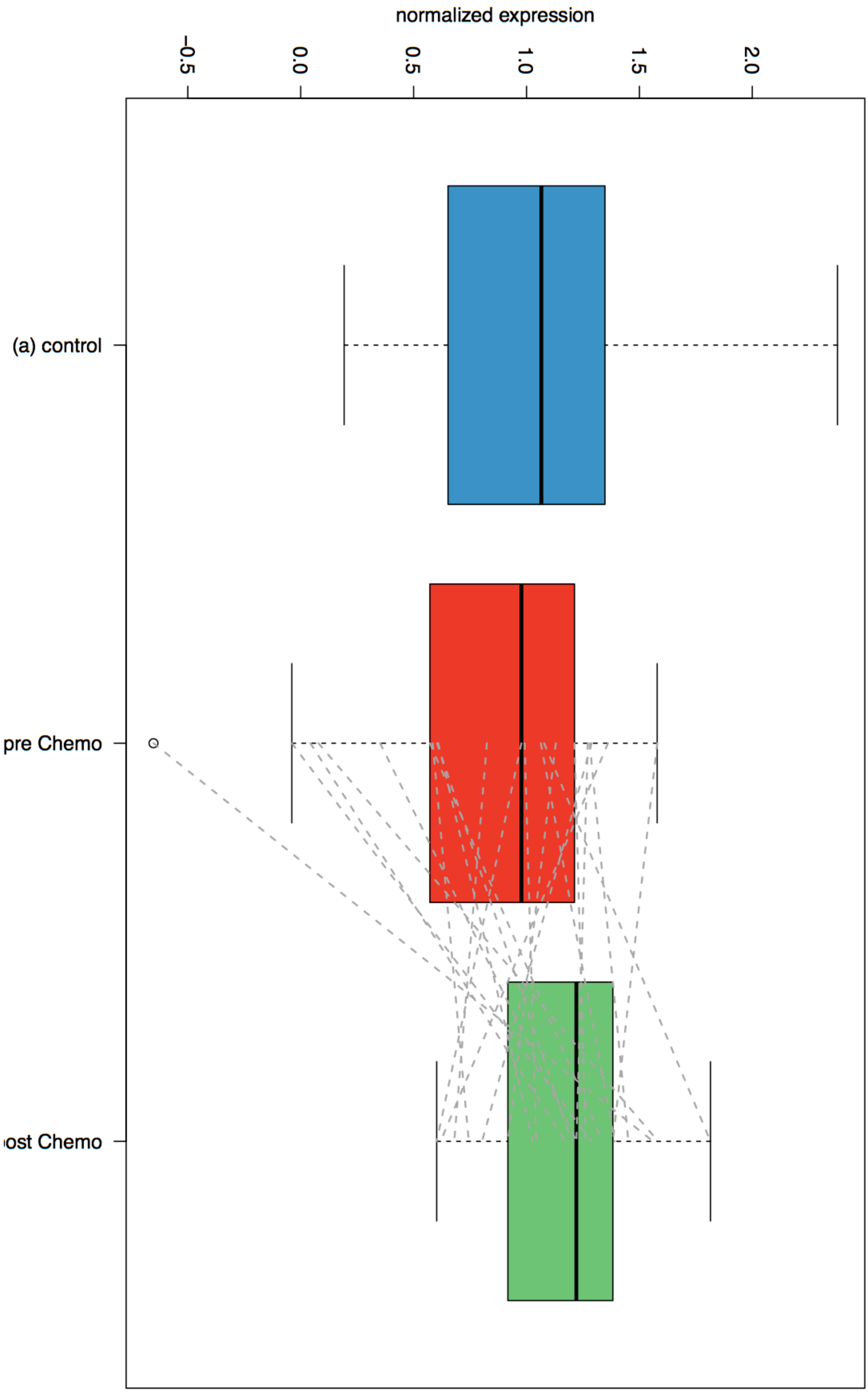
normalized expression
−0.5
0.0
0.5
1.0
1.5
2.0
(a) control
pre Chemo
ost Chemo

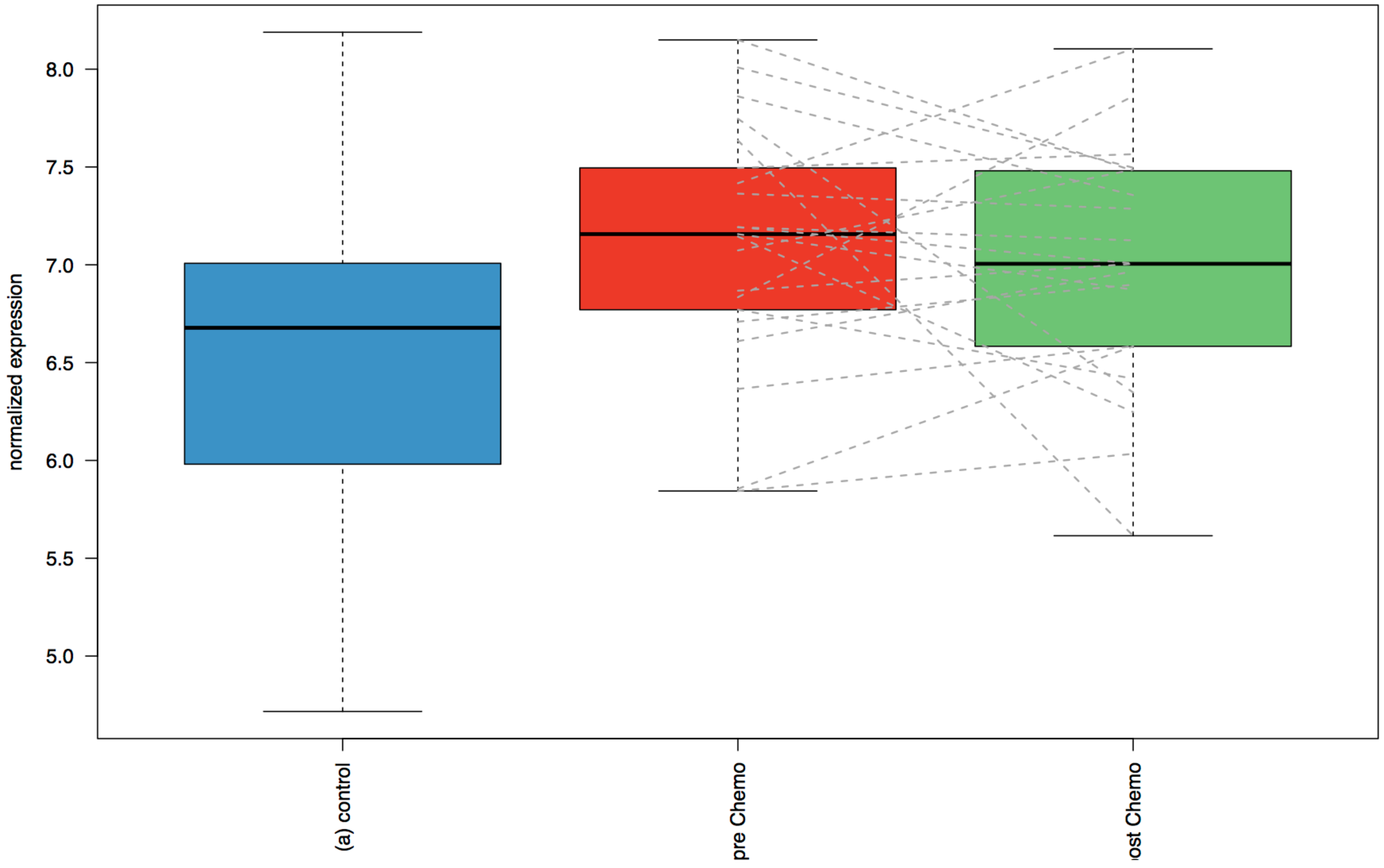
normalized expression
8.0
7.5
7.0
6.5
6.0
5.5
5.0
(a) control
pre Chemo
ost Chemo

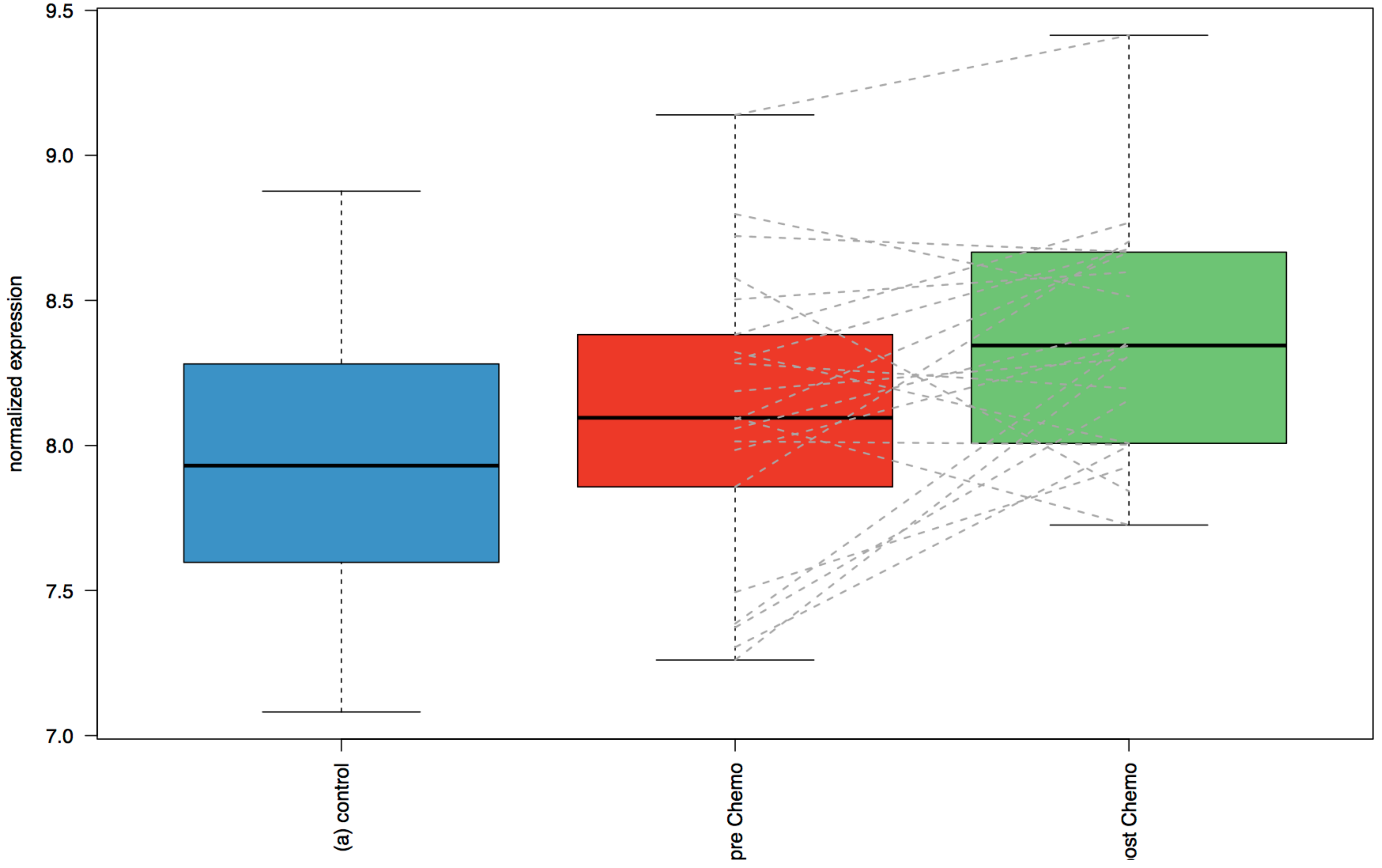

normalized expression
9.5
9.0
8.5
8.0
7.5
7.0
(a) control
pre Chemo
ost Chemo

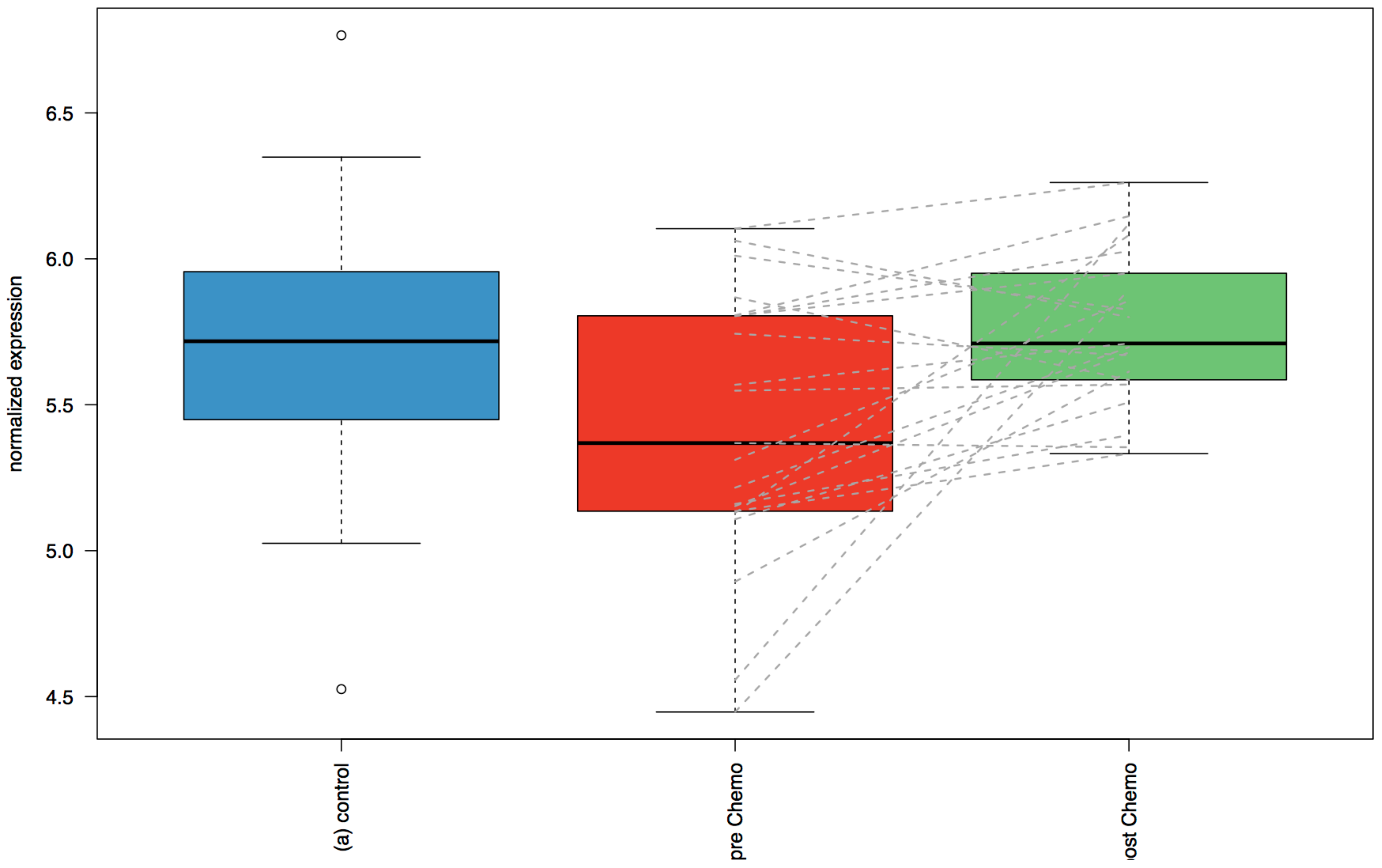
normalized expression
6.5
6.0
5.5
5.0
4.5
(a) control
pre Chemo
ost Chemo

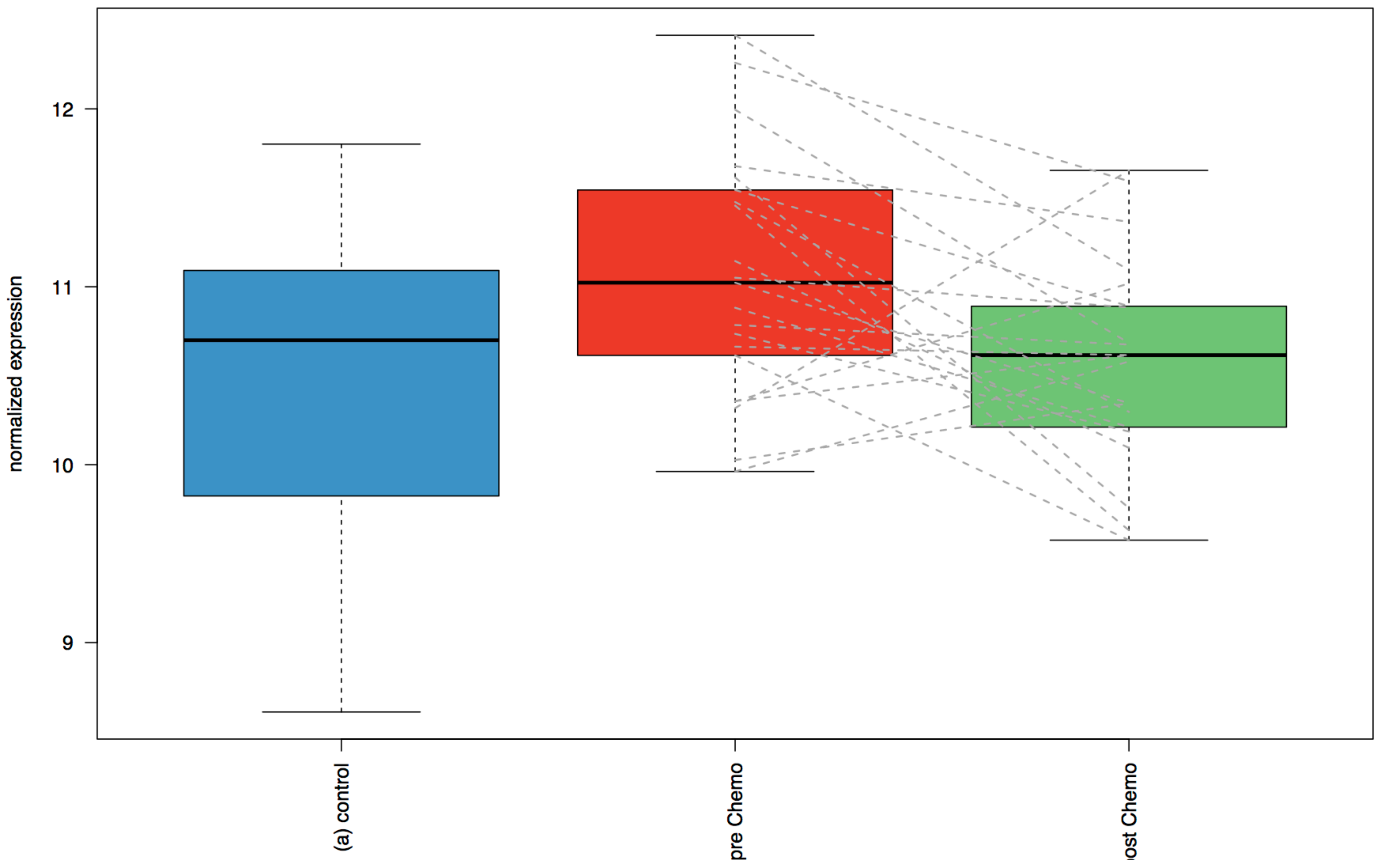
normalized expression
12
11
10
9
(a) control
pre Chemo
ost Chemo

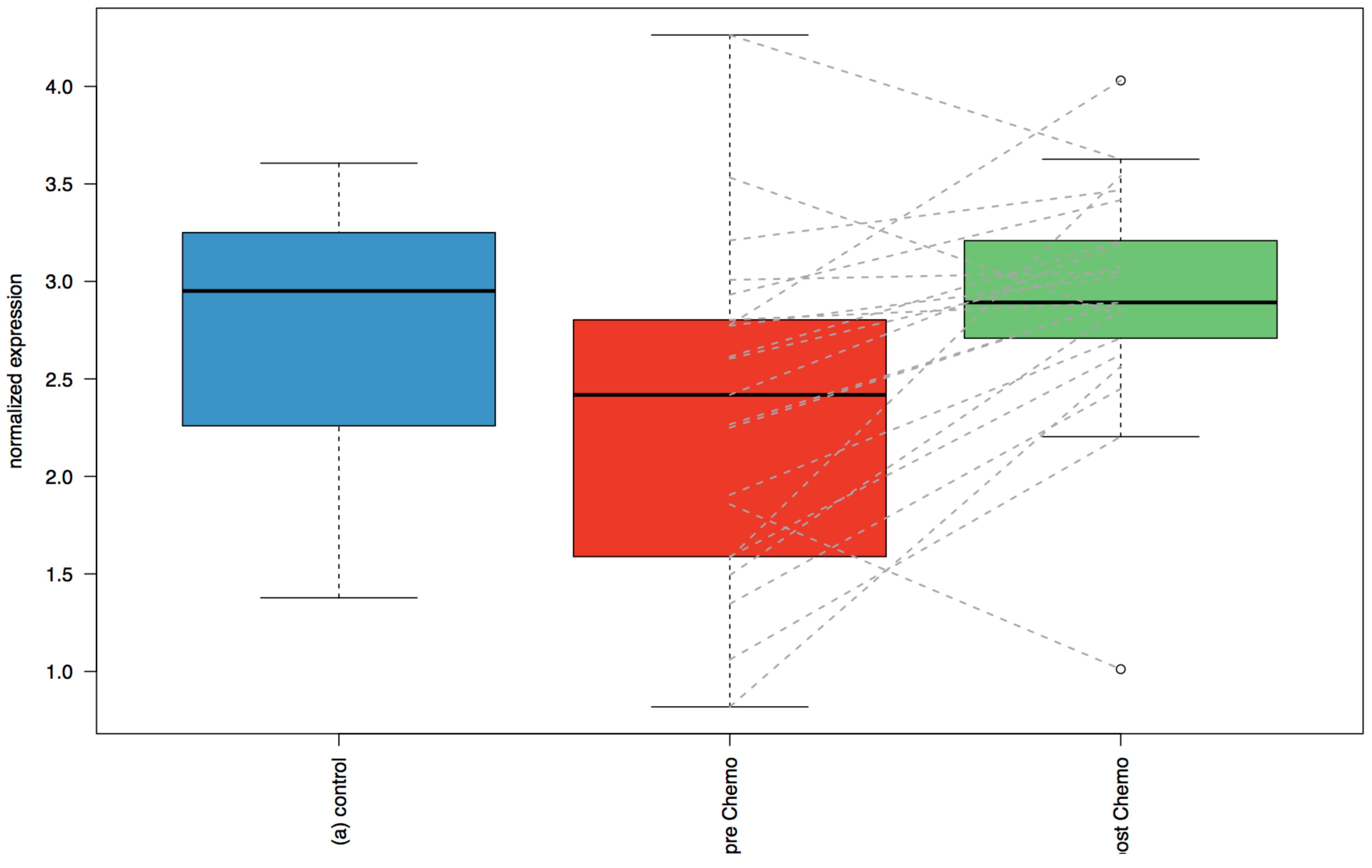
normalized expression
4.0
3.5
3.0
2.5
2.0
1.5
1.0
(a) control
pre Chemo
ost Chemo

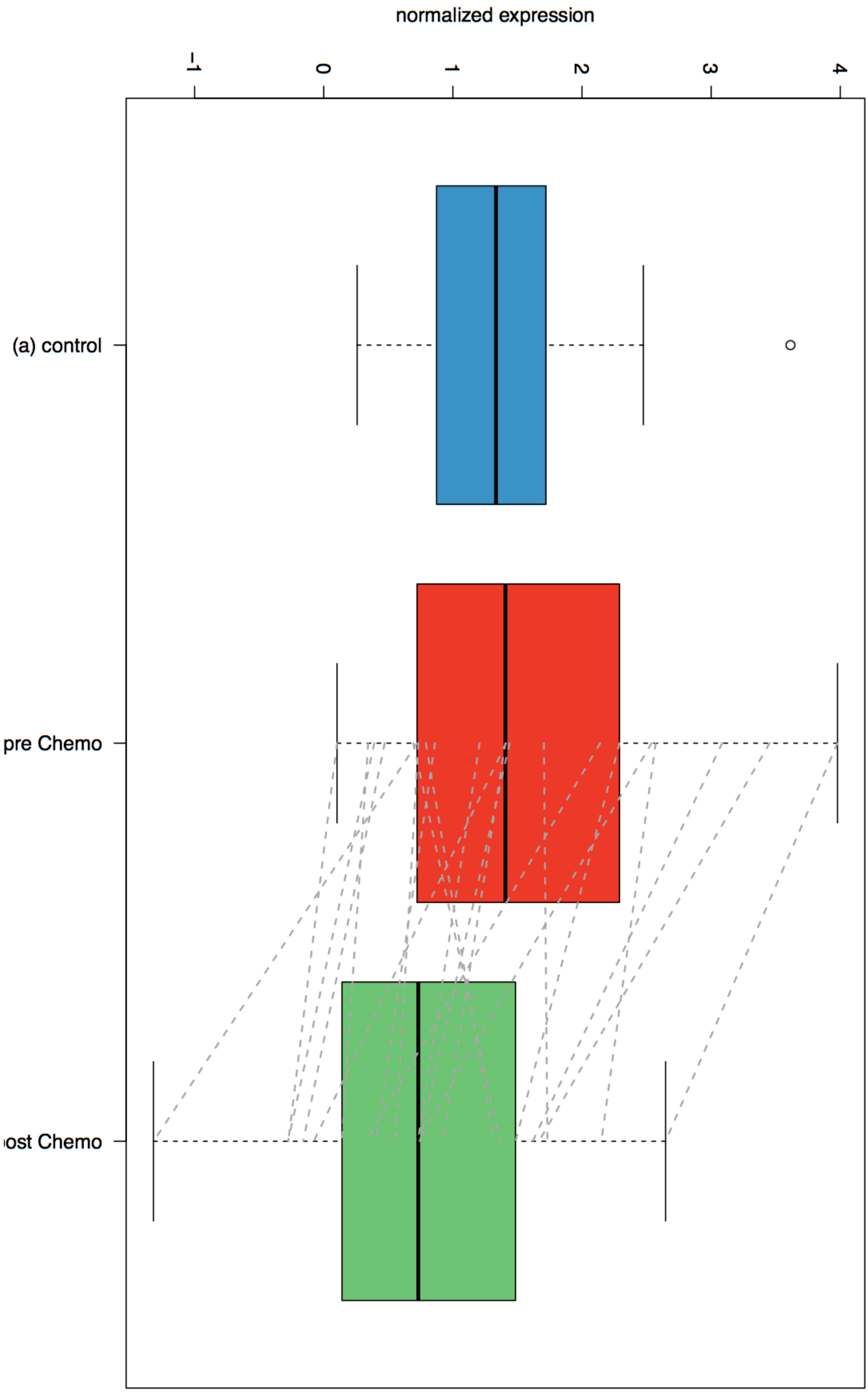
normalized expression
-1
0
1
2
3
4
(a) control
pre Chemo
ost Chemo

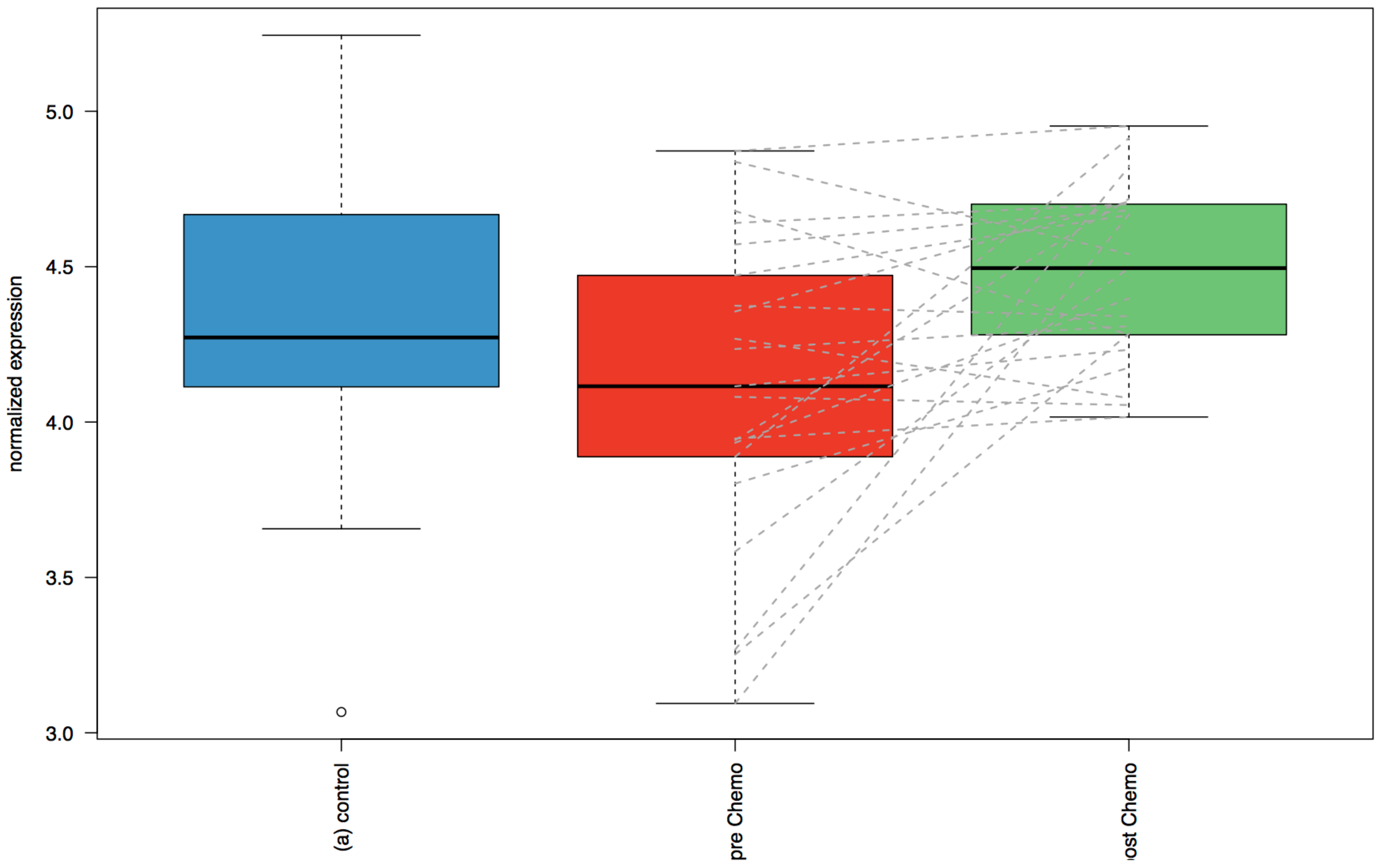
normalized expression
5.0
4.5
4.0
3.5
3.0
(a) control
pre Chemo
ost Chemo

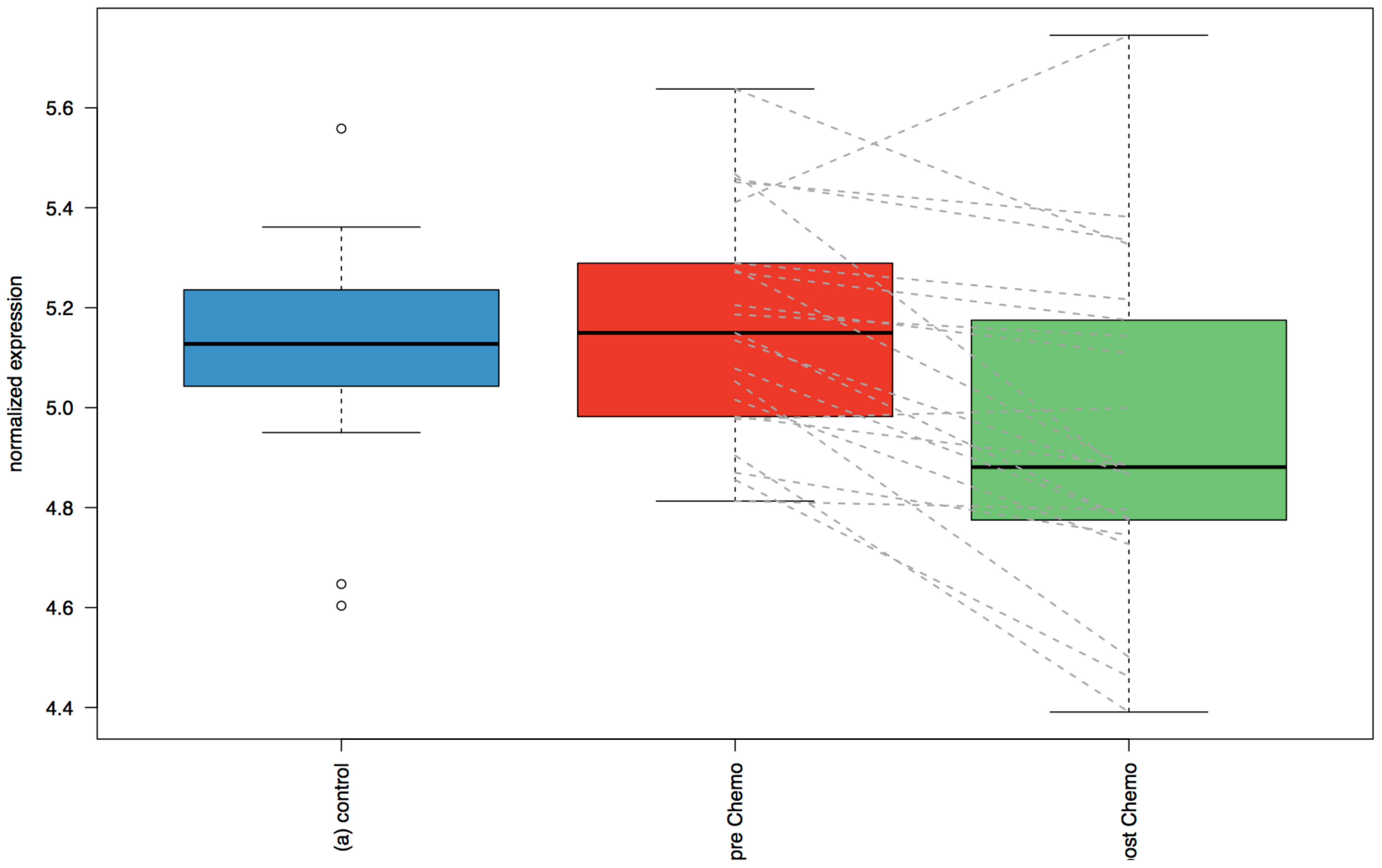
normalized expression
5.6
5.4
5.2
5.0
4.8
4.6
4.4
(a) control
pre Chemo
ost Chemo

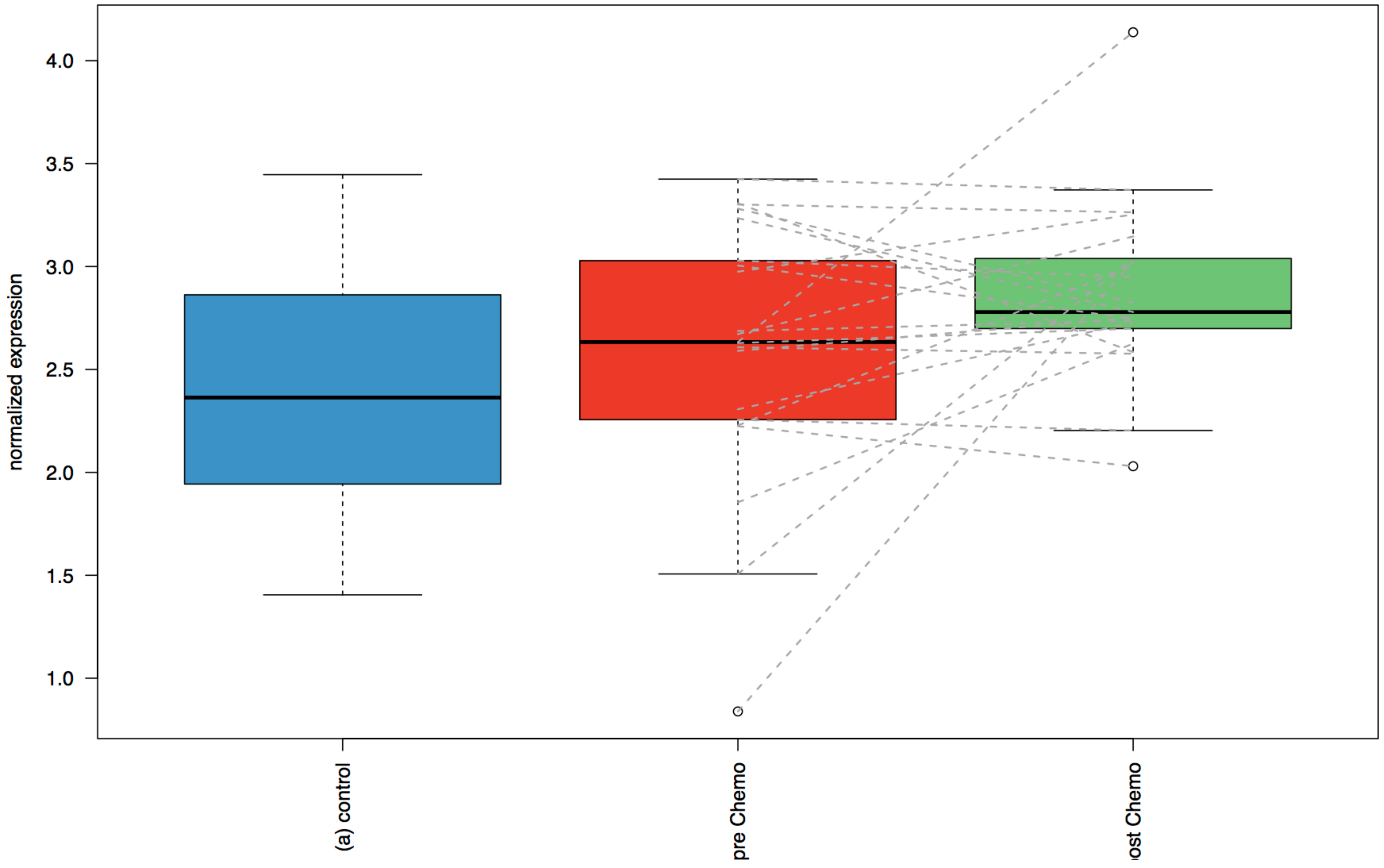
normalized expression
4.0
3.5
3.0
2.5
2.0
1.5
1.0
(a) control
pre Chemo
ost Chemo

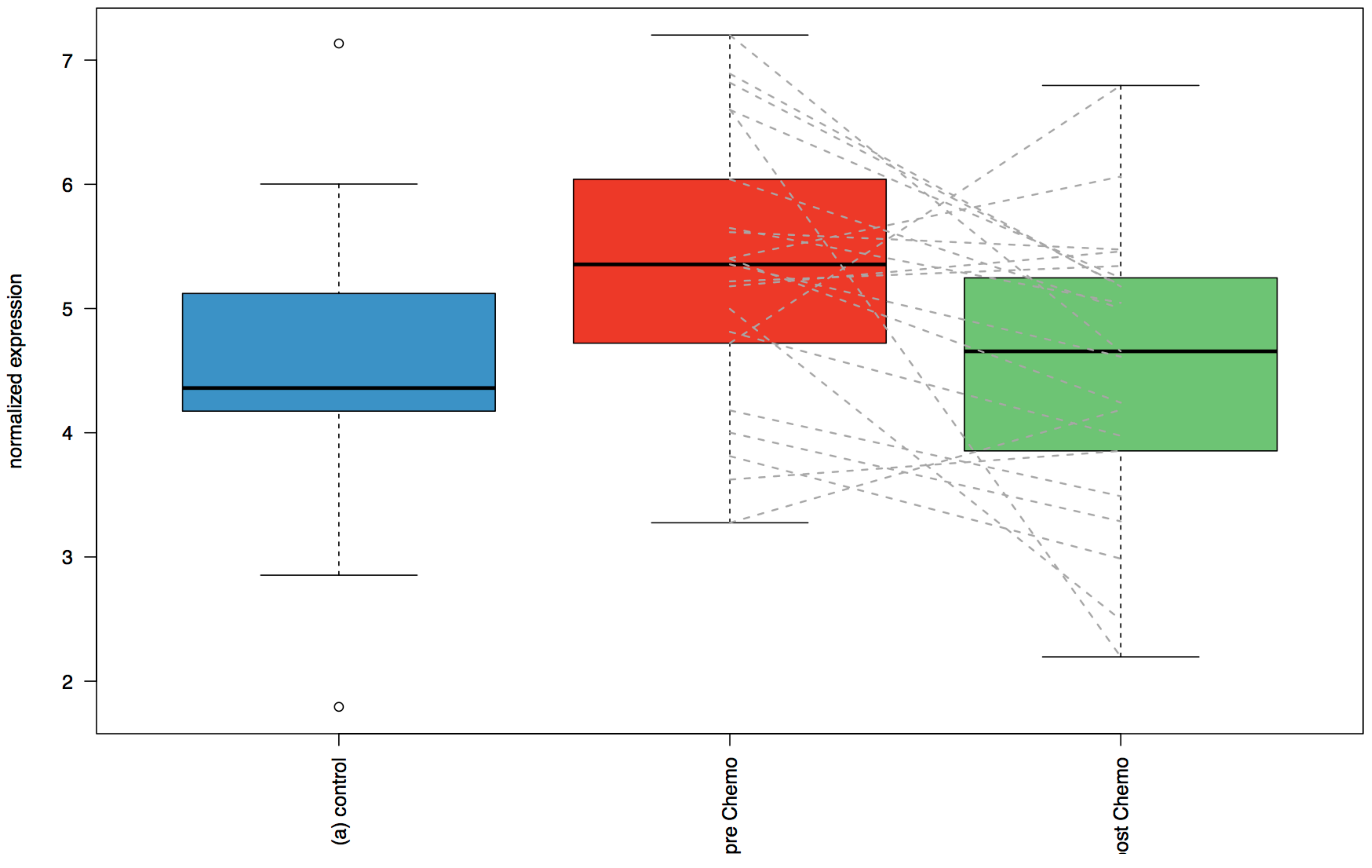

normalized expression
7
6
5
4
3
2
(a) control
pre Chemo
ost Chemo

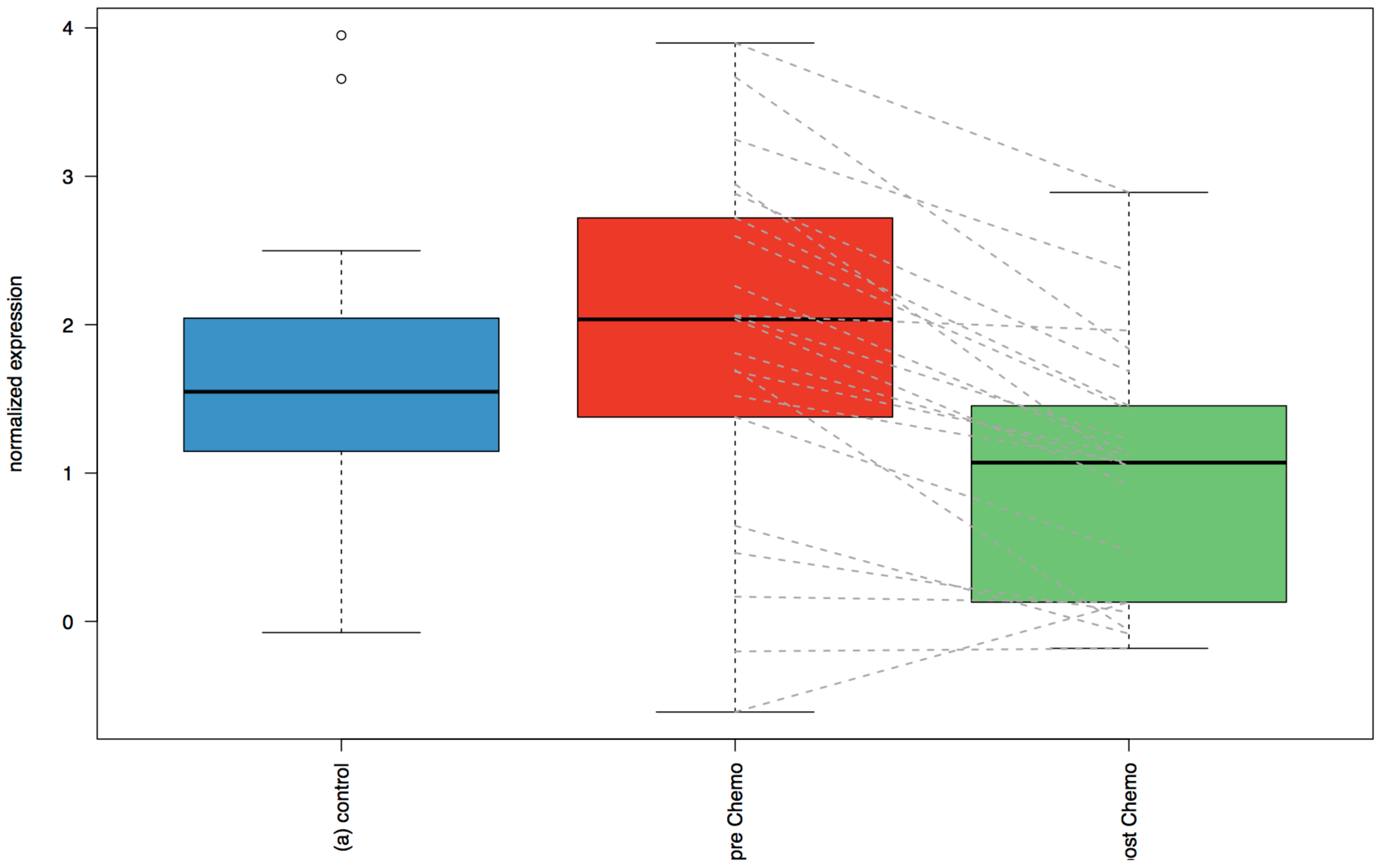
normalized expression
4
3
2
1
0
(a) control
pre Chemo
ost Chemo

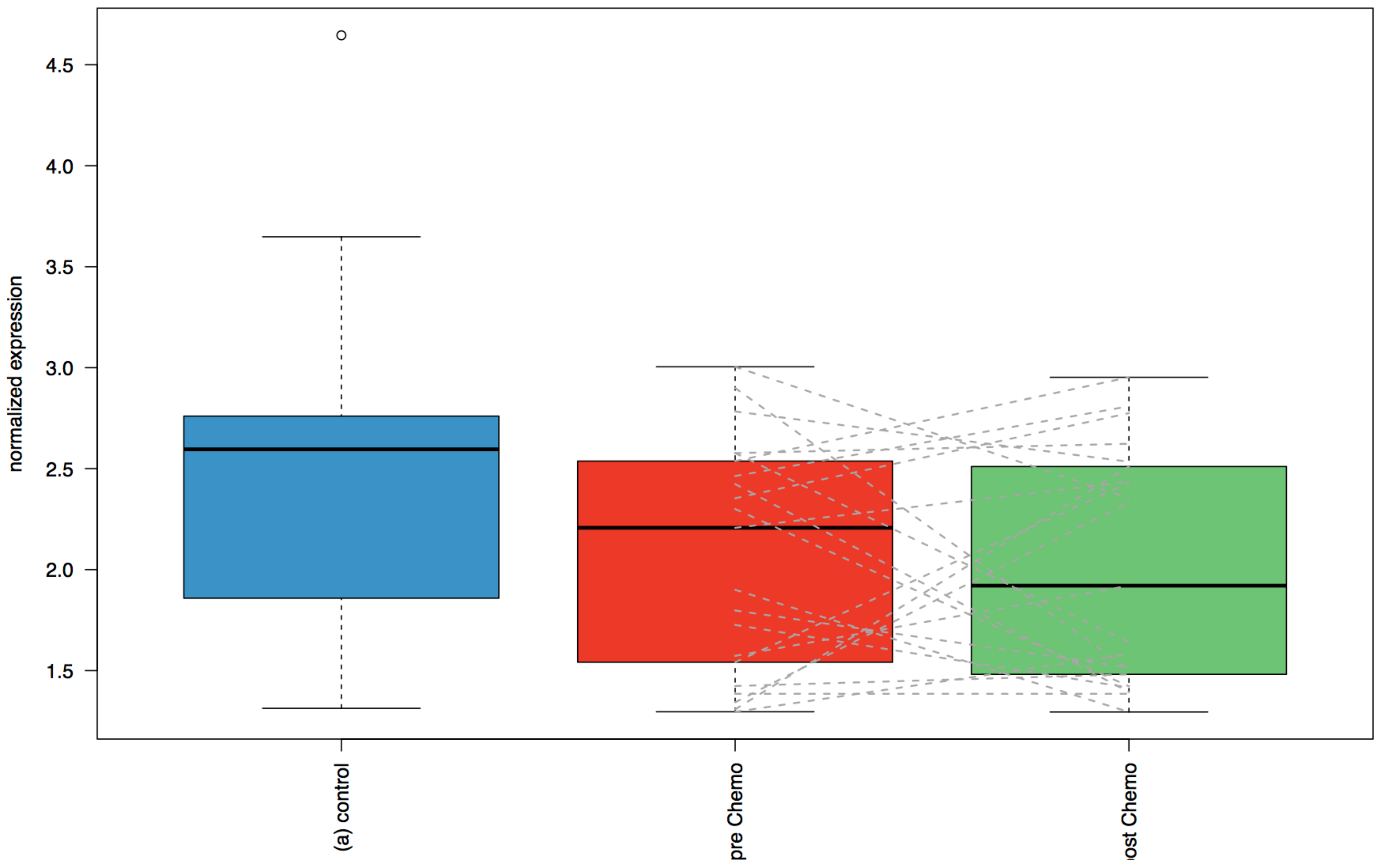

normalized expression
4.5
4.0
3.5
3.0
2.5
2.0
1.5
(a) control
pre Chemo
ost Chemo

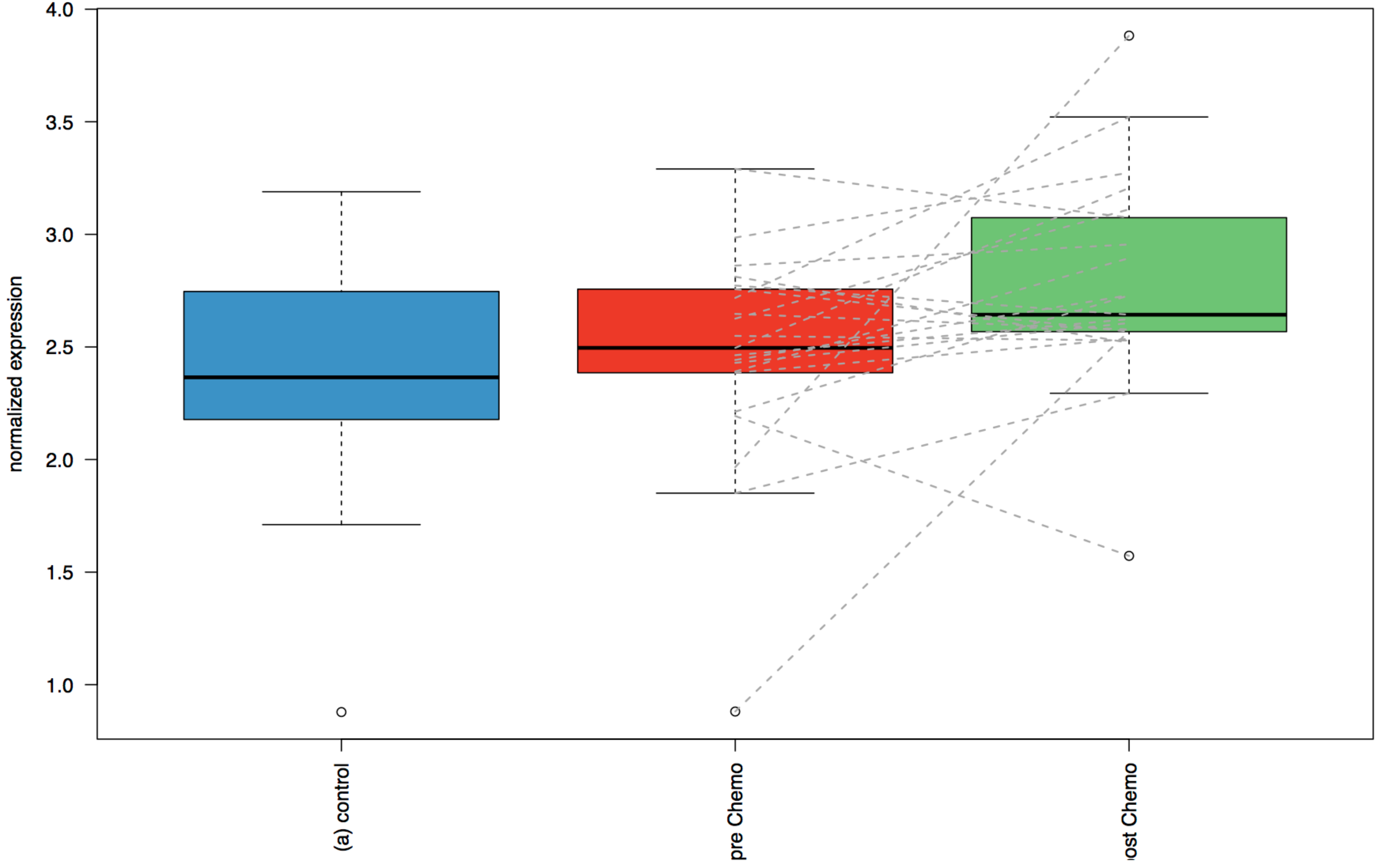
normalized expression
4.0
3.5
3.0
2.5
2.0
1.5
1.0
(a) control
pre Chemo
ost Chemo

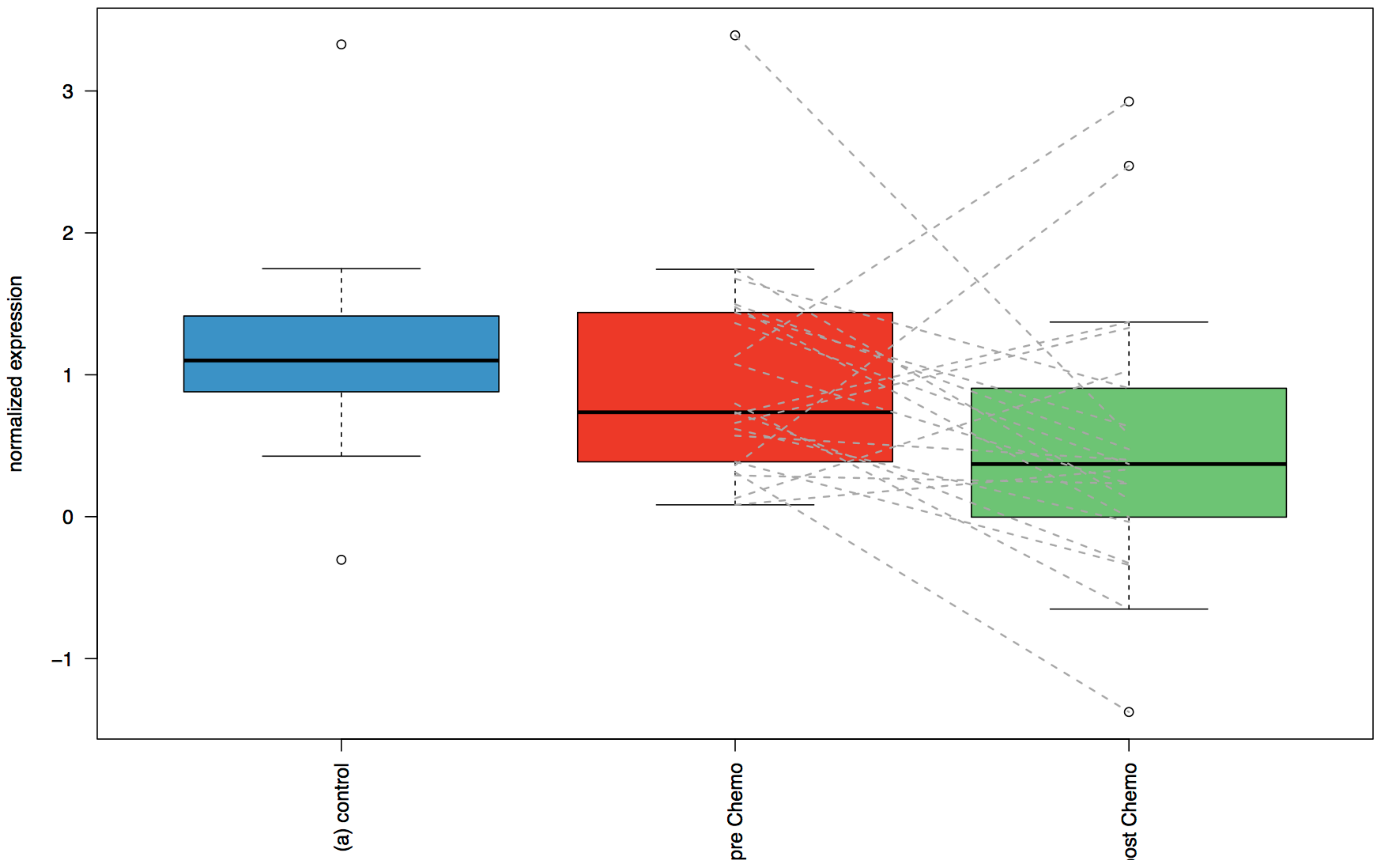
normalized expression
3
2
1
0
−1
(a) control
pre Chemo
ost Chemo

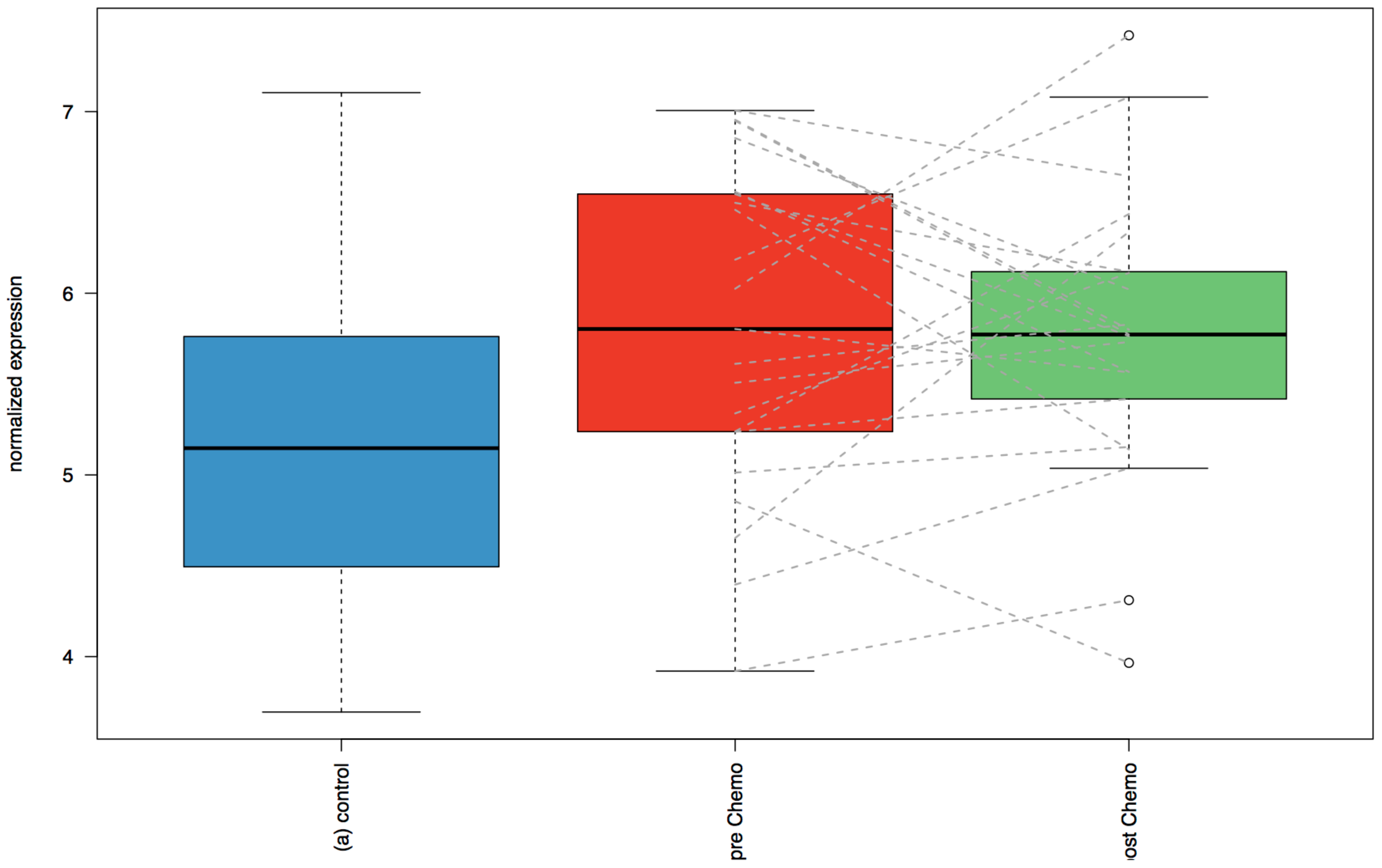
normalized expression
7
6
5
4
(a) control
pre Chemo
ost Chemo

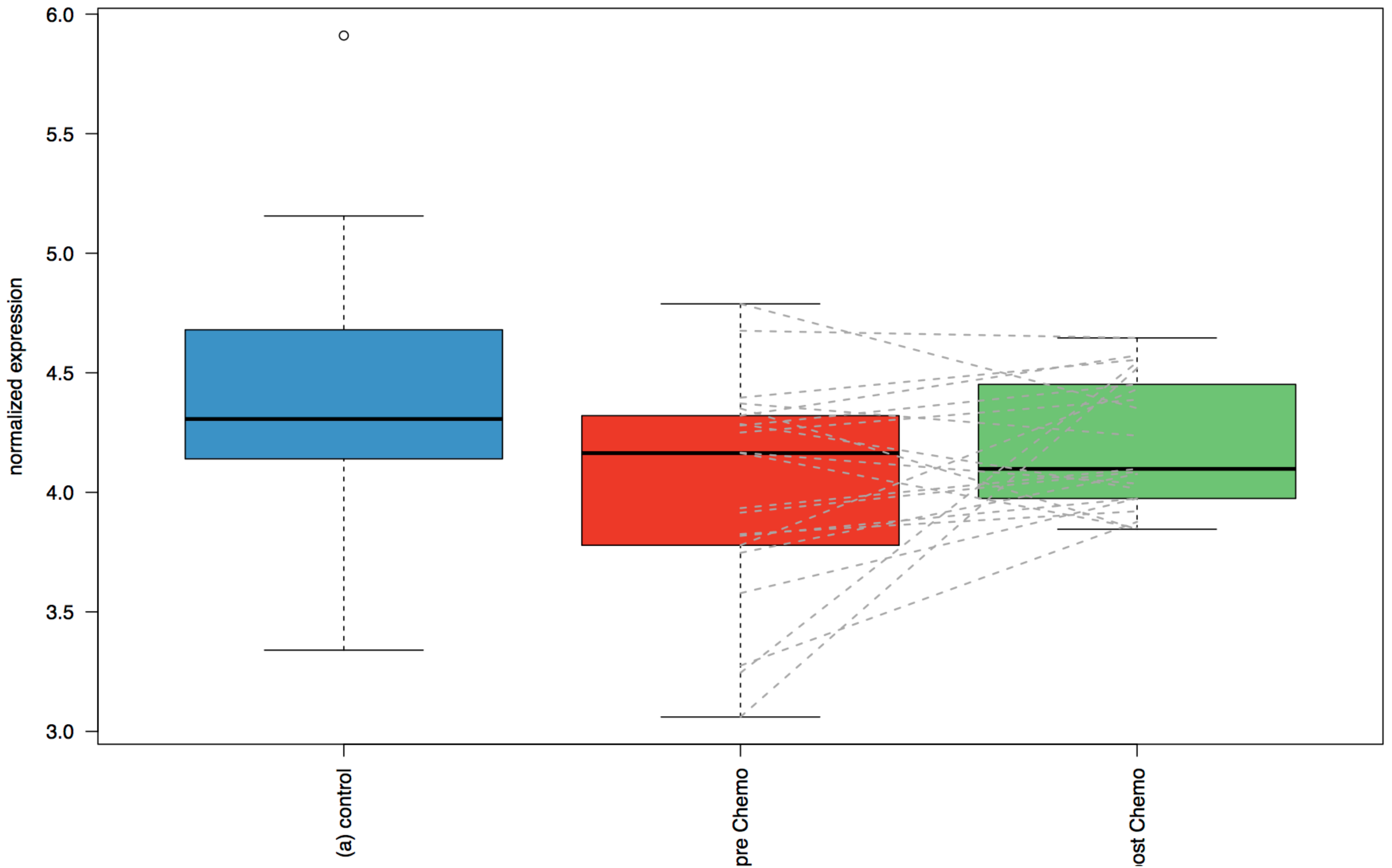
normalized expression
6.0
5.5
5.0
4.5
4.0
3.5
3.0
(a) control
pre Chemo
ost Chemo

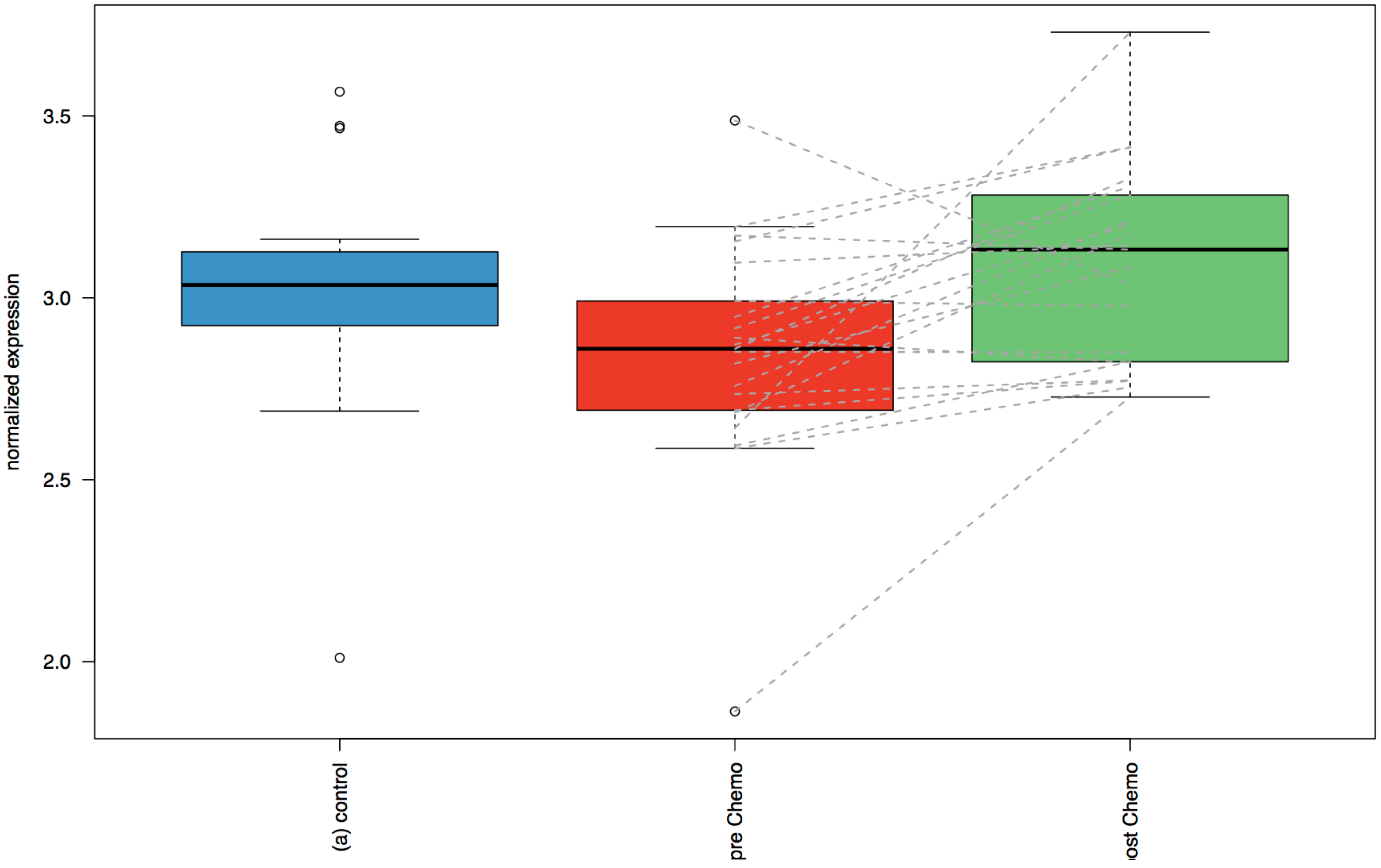
normalized expression
3.5
3.0
2.5
2.0
(a) control
pre Chemo
ost Chemo

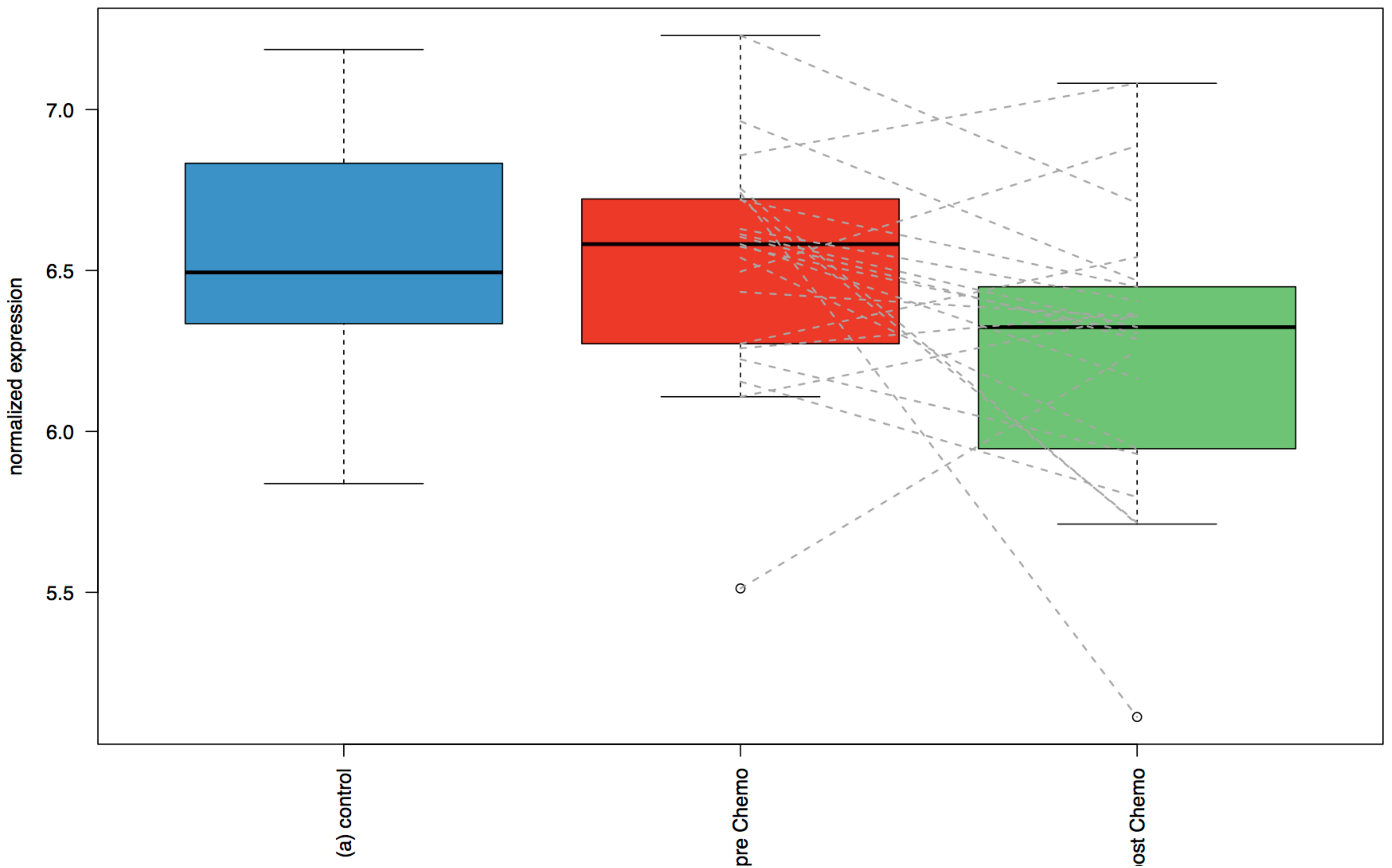

normalized expression
7.0
6.5
6.0
5.5
(a) control
pre Chemo
ost Chemo

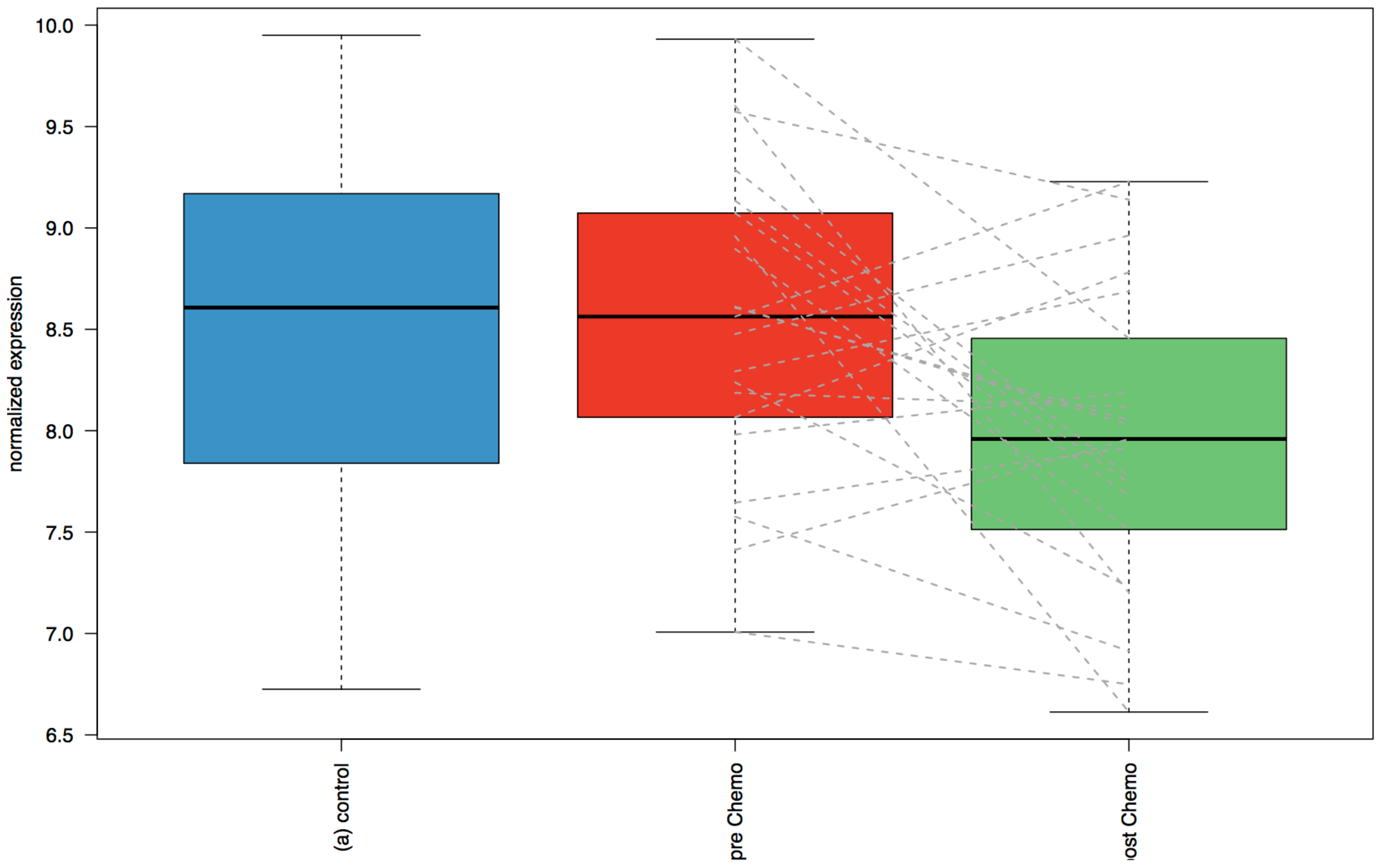
normalized expression
10.0
9.5
9.0
8.5
8.0
7.5
7.0
6.5
(a) control
pre Chemo
ost Chemo

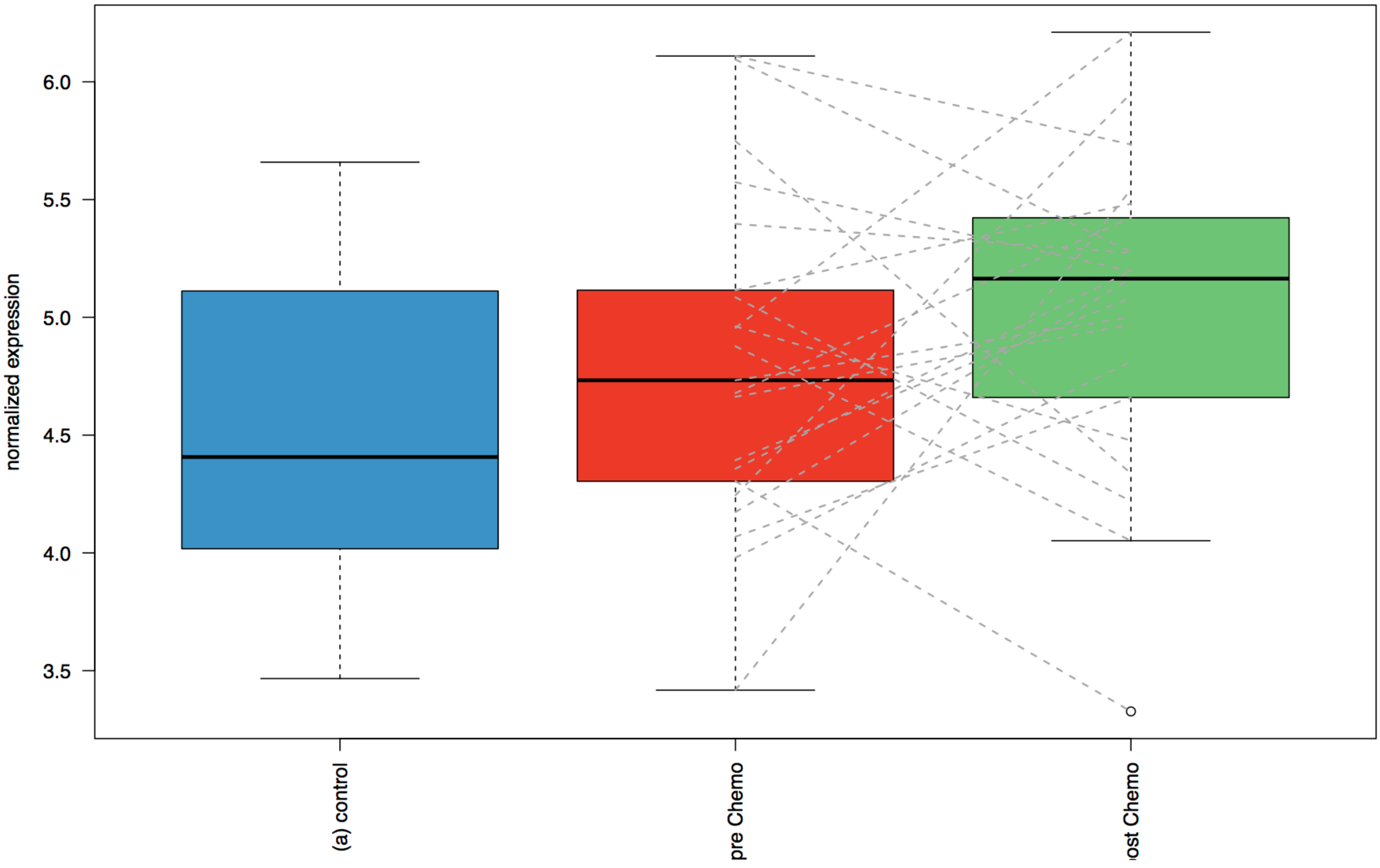
normalized expression
6.0
5.5
5.0
4.5
4.0
3.5
(a) control
pre Chemo
ost Chemo

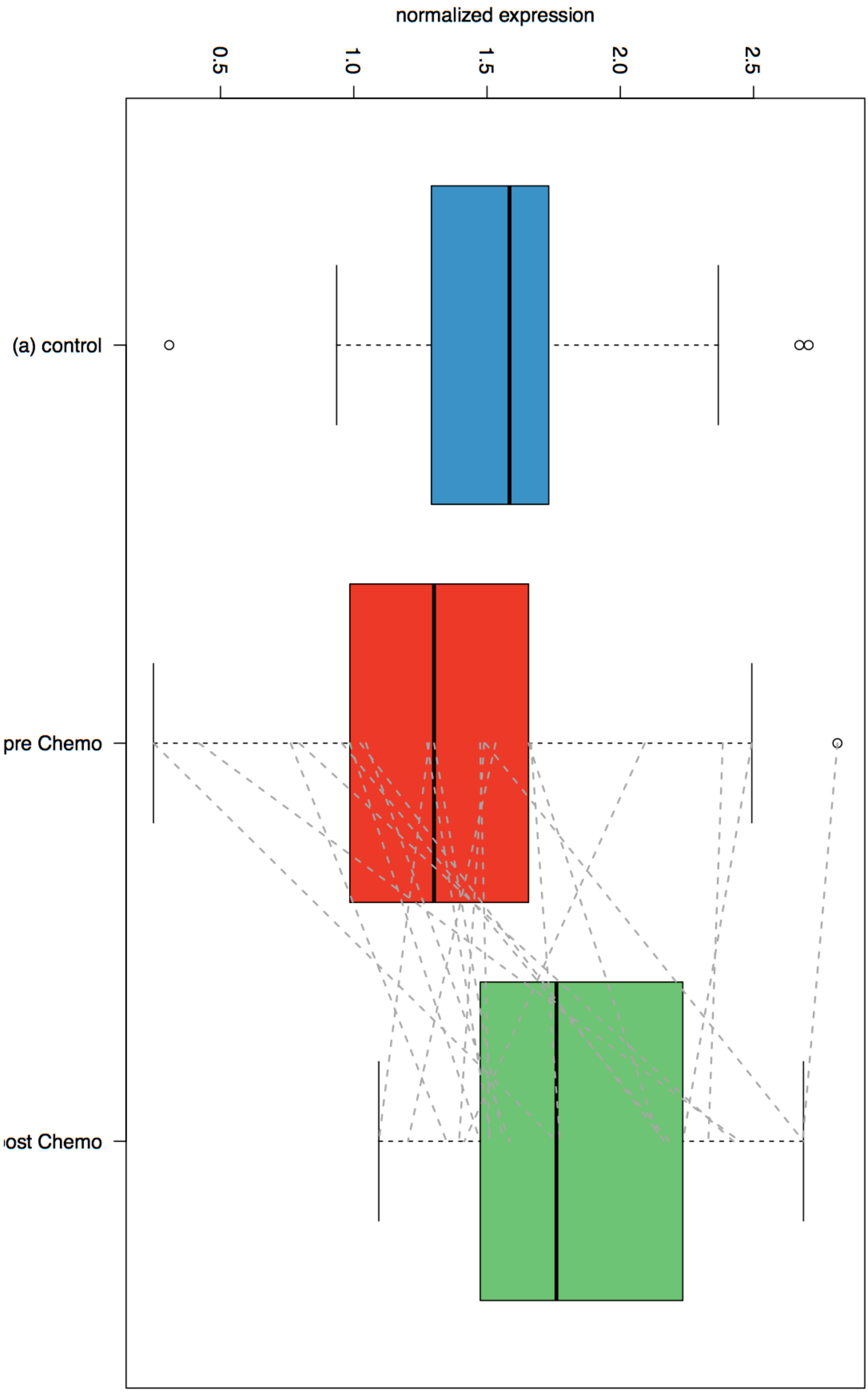
normalized expression
0.5
1.0
1.5
2.0
2.5
(a) control
pre Chemo
ost Chemo

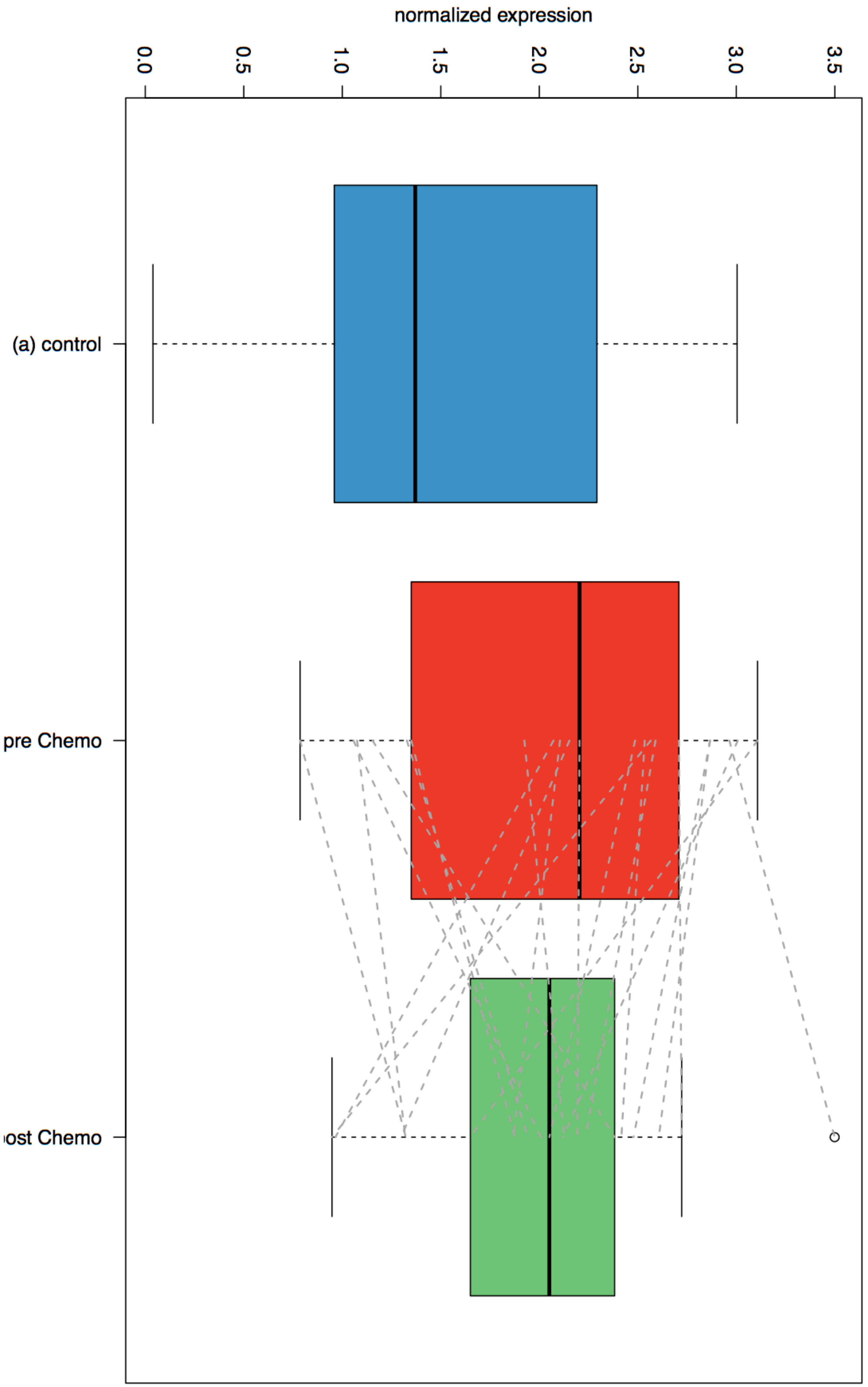
normalized expression
0.0
0.5
1.0
1.5
2.0
2.5
3.0
3.5
(a) control
pre Chemo
ost Chemo

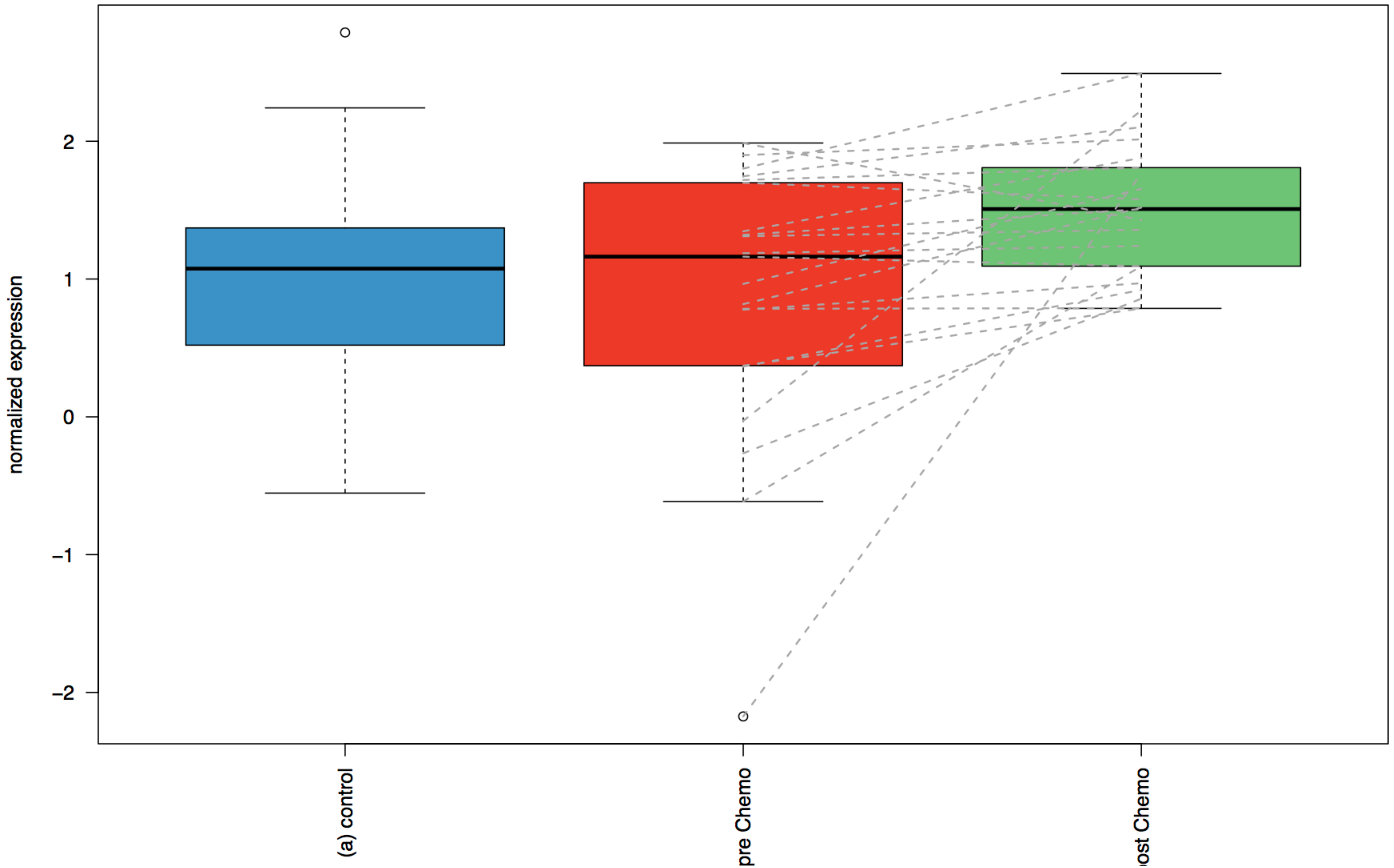
normalized expression
2
1
0
−1
−2
(a) control
pre Chemo
ost Chemo

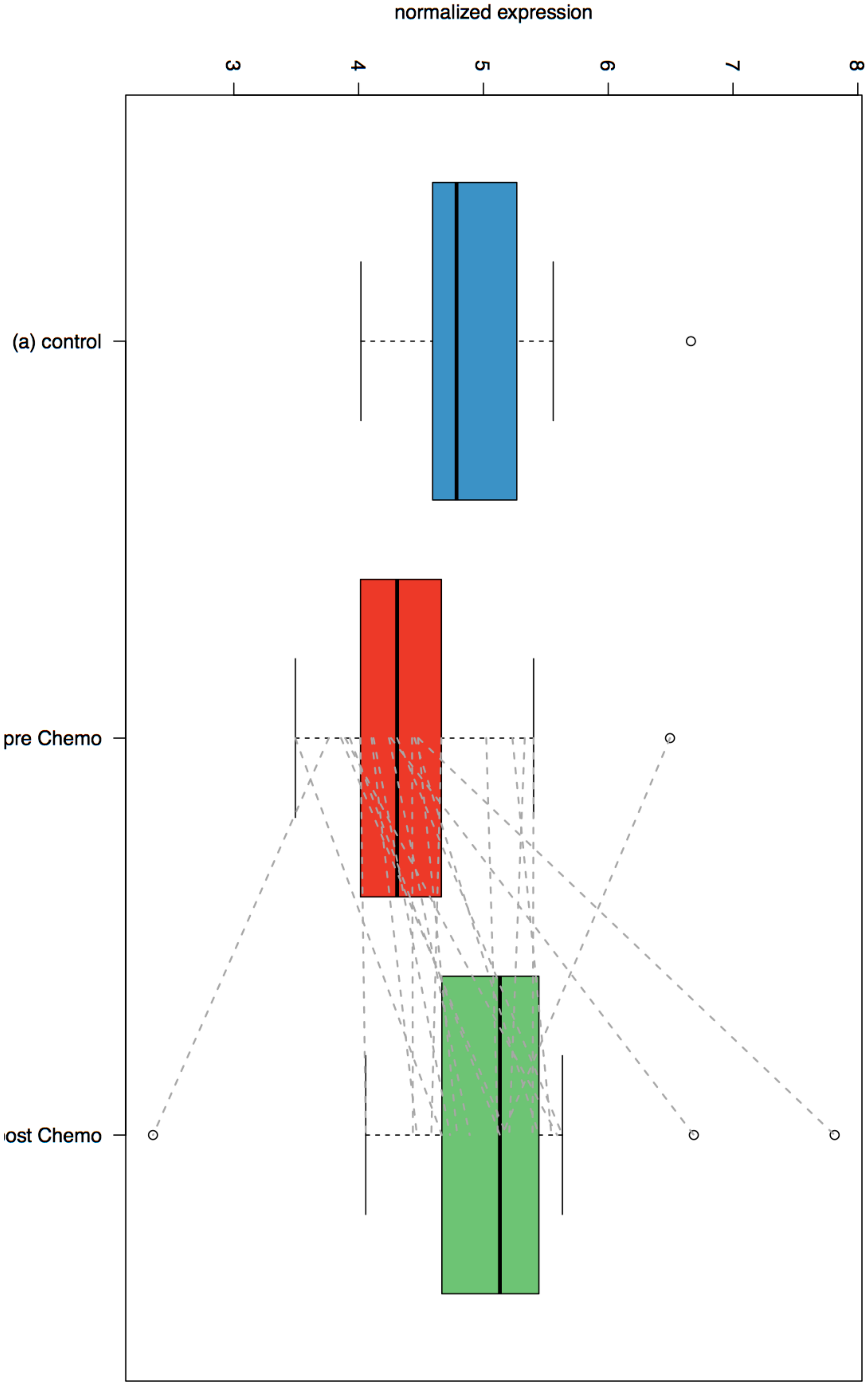

normalized expression
3
4
5
6
7
8
(a) control
pre Chemo
ost Chemo

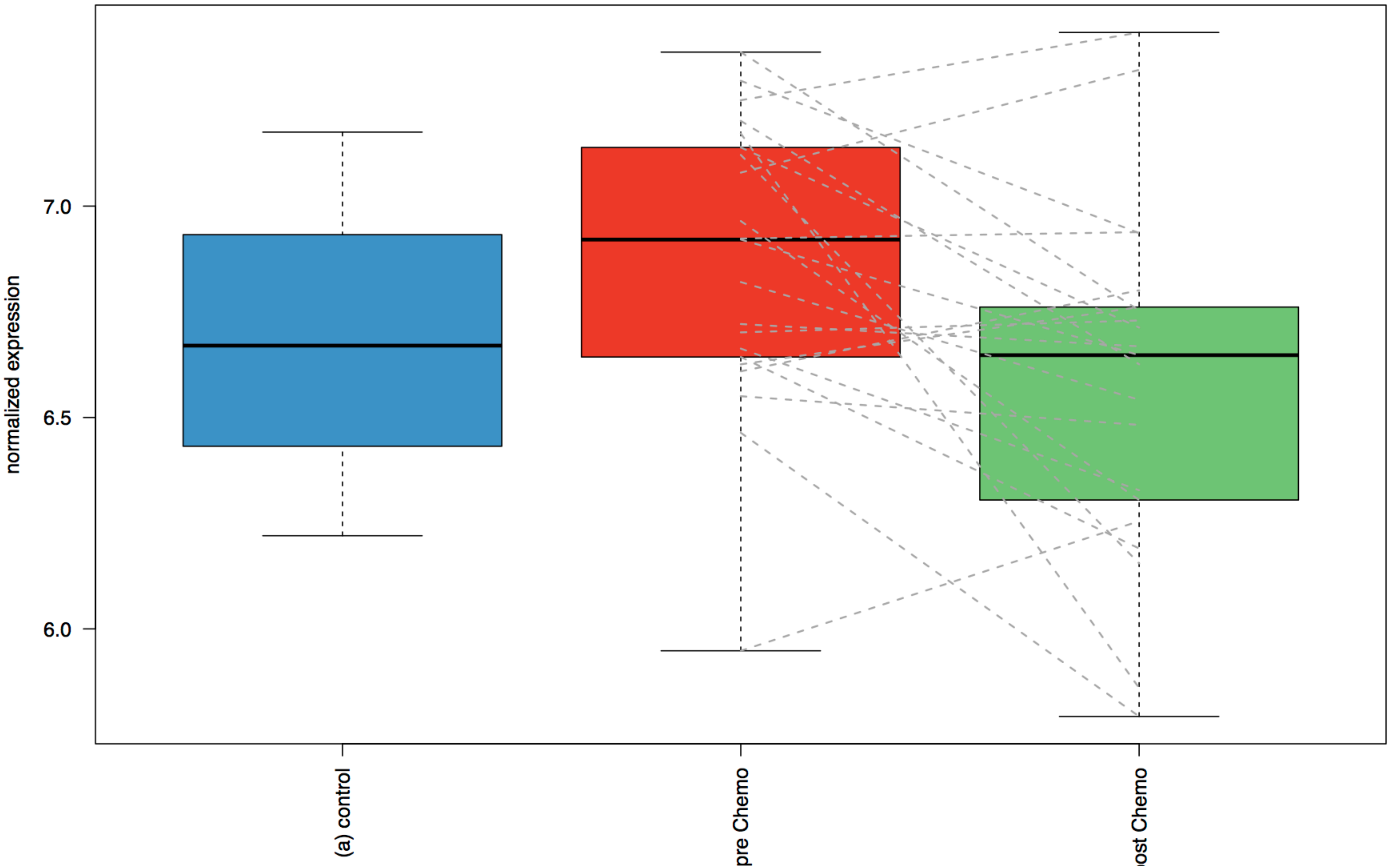
normalized expression
7.0
6.5
6.0
(a) control
pre Chemo
ost Chemo

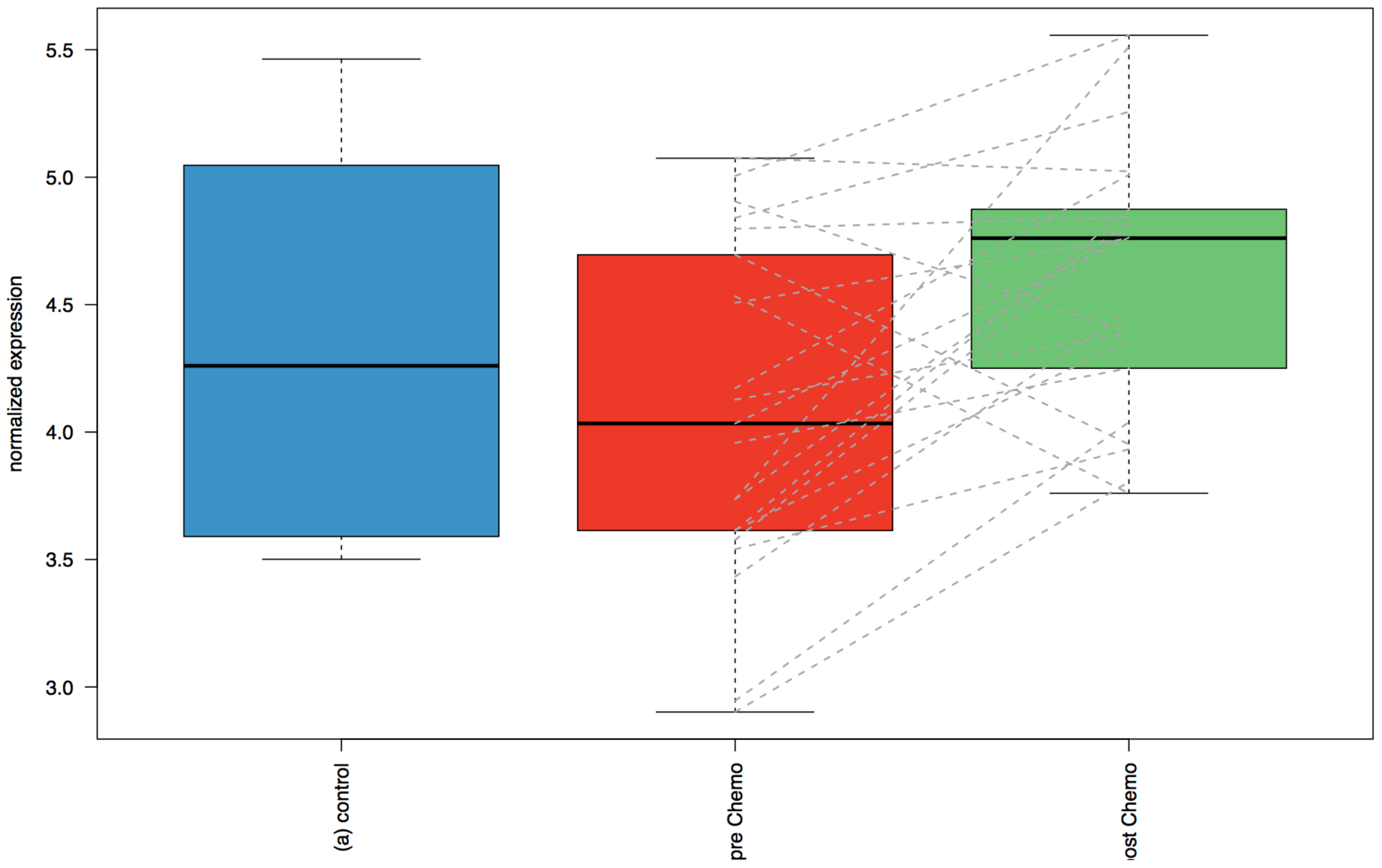

normalized expression
5.5
5.0
4.5
4.0
3.5
3.0
(a) control
pre Chemo
ost Chemo

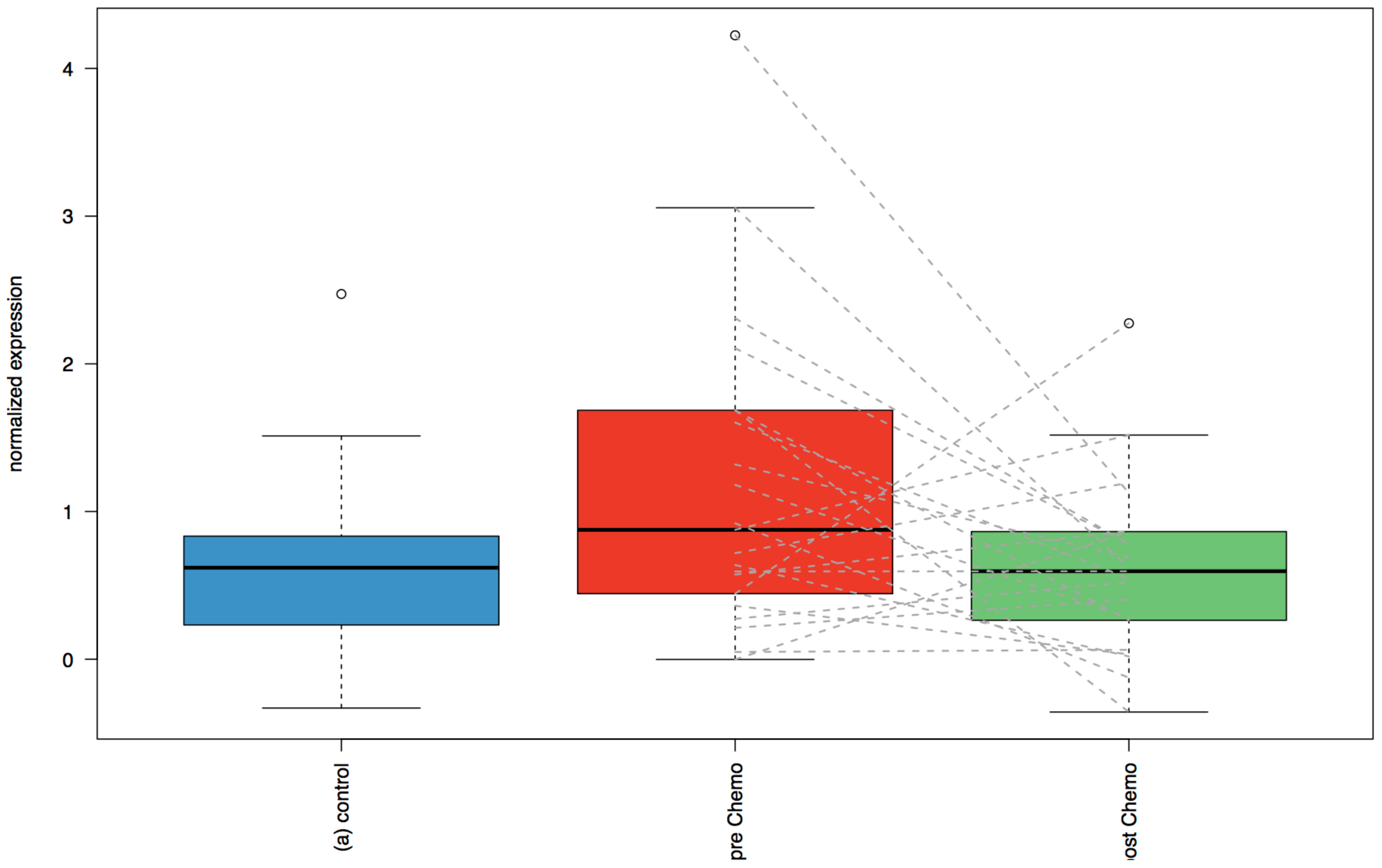
normalized expression
0
1
2
3
4
(a) control
pre Chemo
ost Chemo

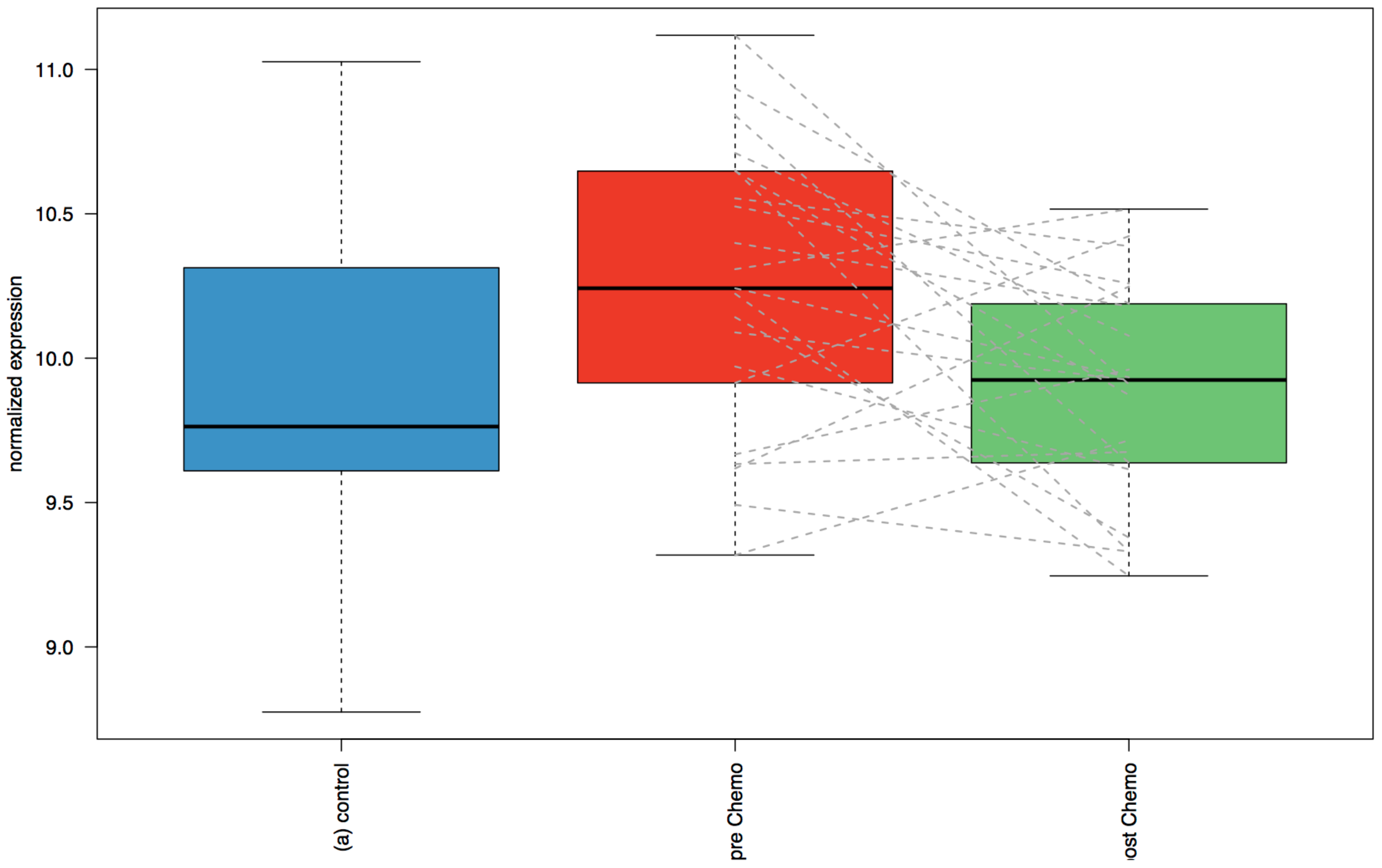
normalized expression
11.0
10.5
10.0
9.5
9.0
(a) control
pre Chemo
ost Chemo

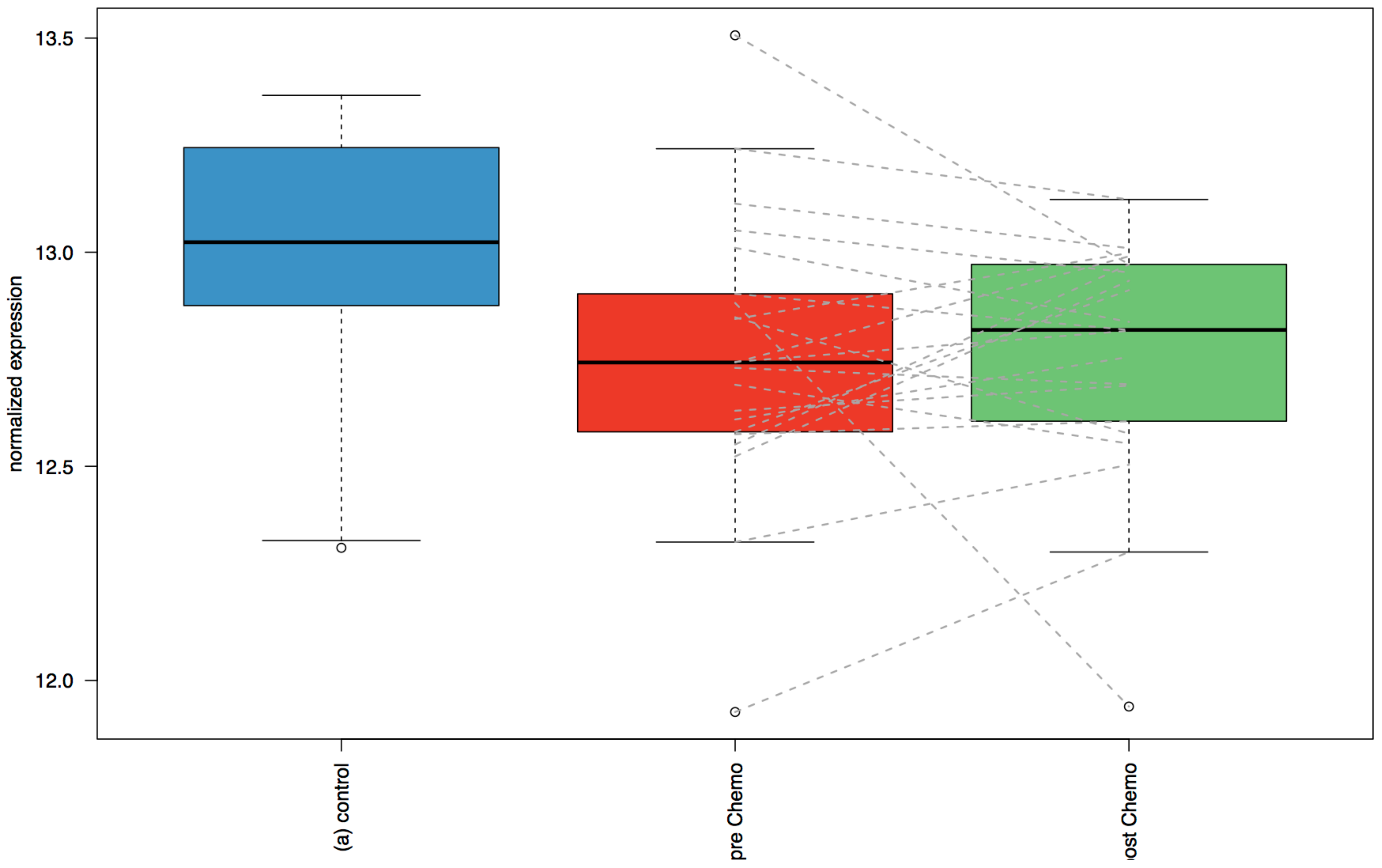
normalized expression
13.5
13.0
12.5
12.0
(a) control
pre Chemo
ost Chemo

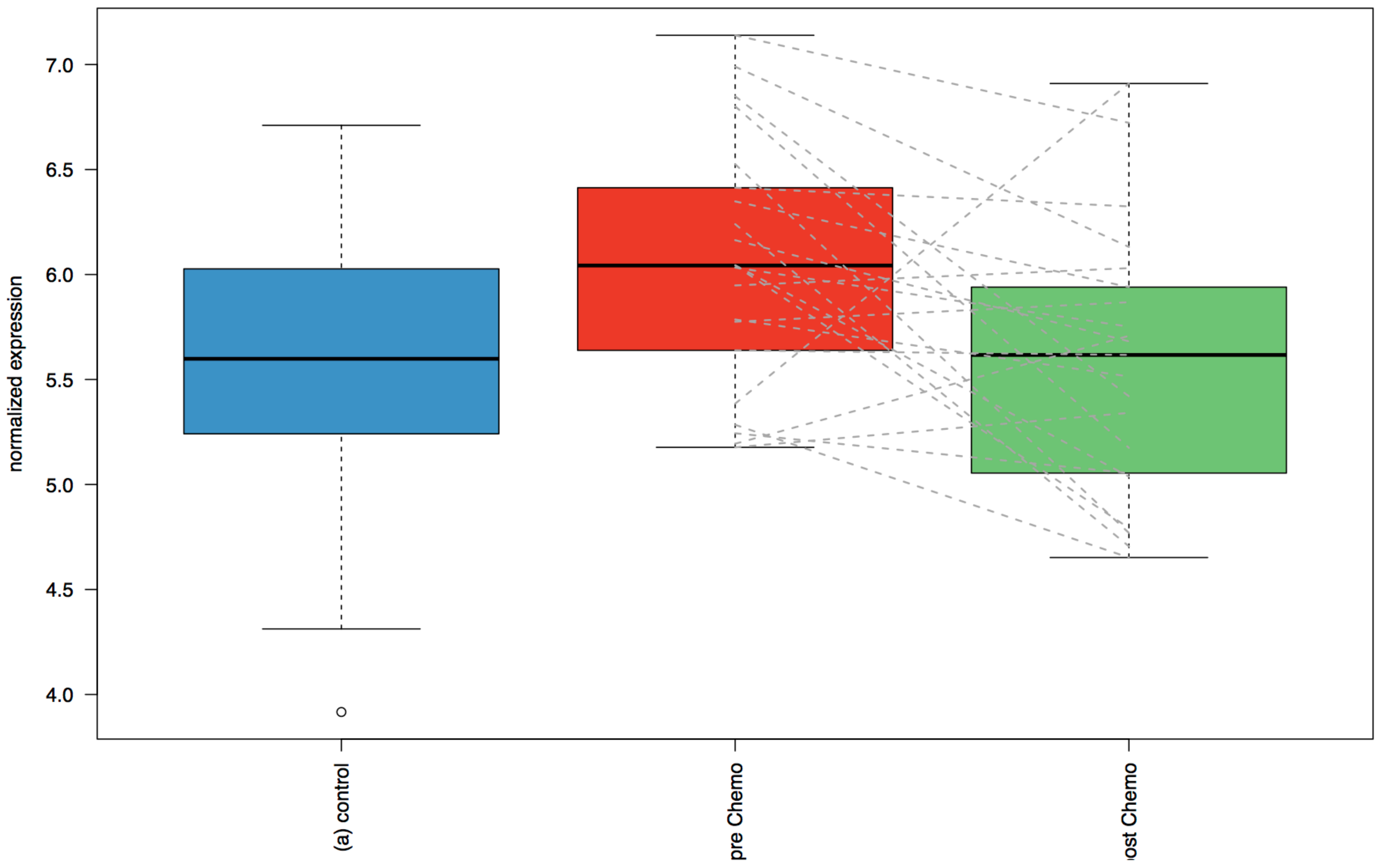
normalized expression
7.0
6.5
6.0
5.5
5.0
4.5
4.0
(a) control
pre Chemo
ost Chemo

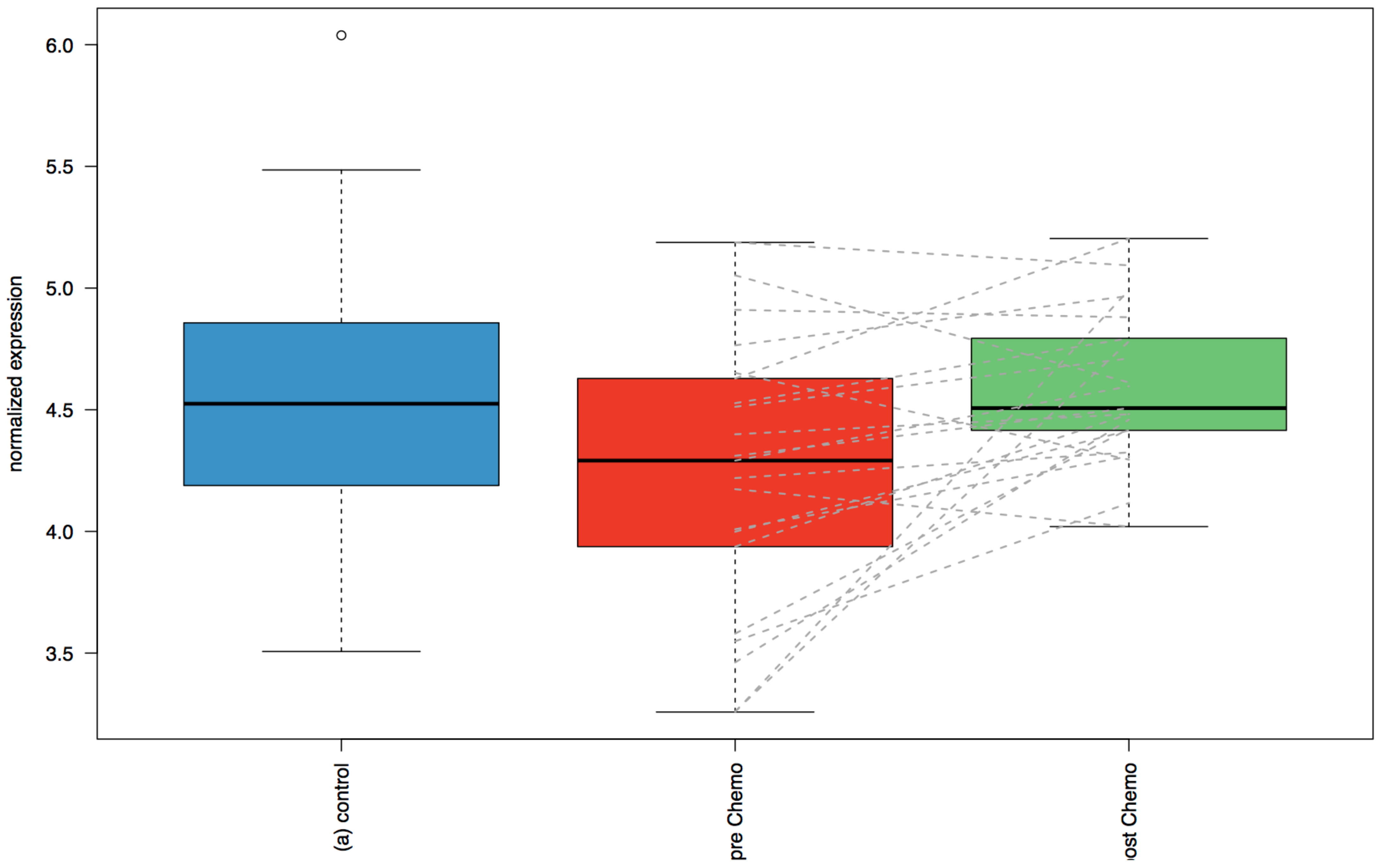

normalized expression
6.0
5.5
5.0
4.5
4.0
3.5
(a) control
pre Chemo
ost Chemo

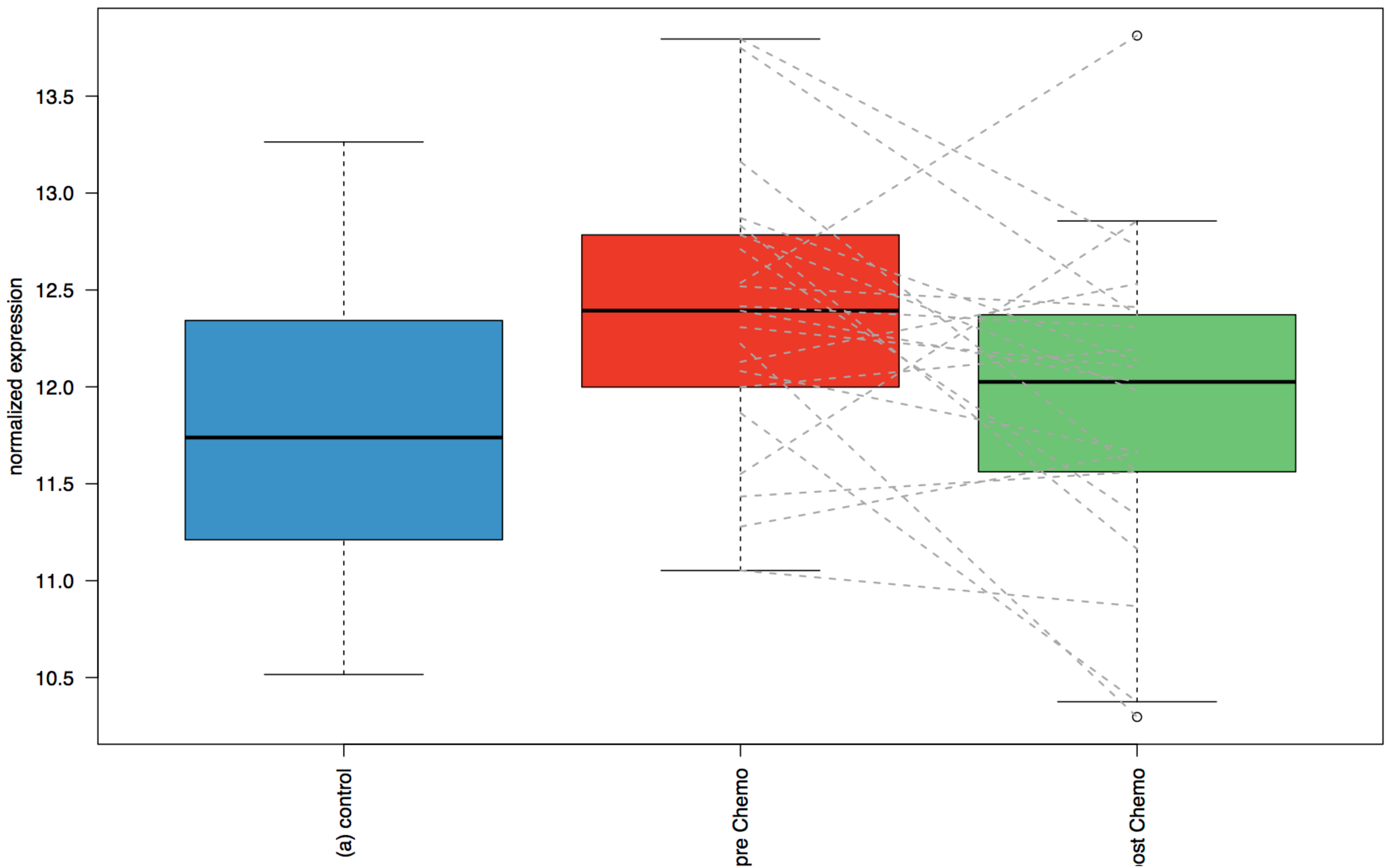
normalized expression
13.5
13.0
12.5
12.0
11.5
11.0
10.5
(a) control
pre Chemo
ost Chemo

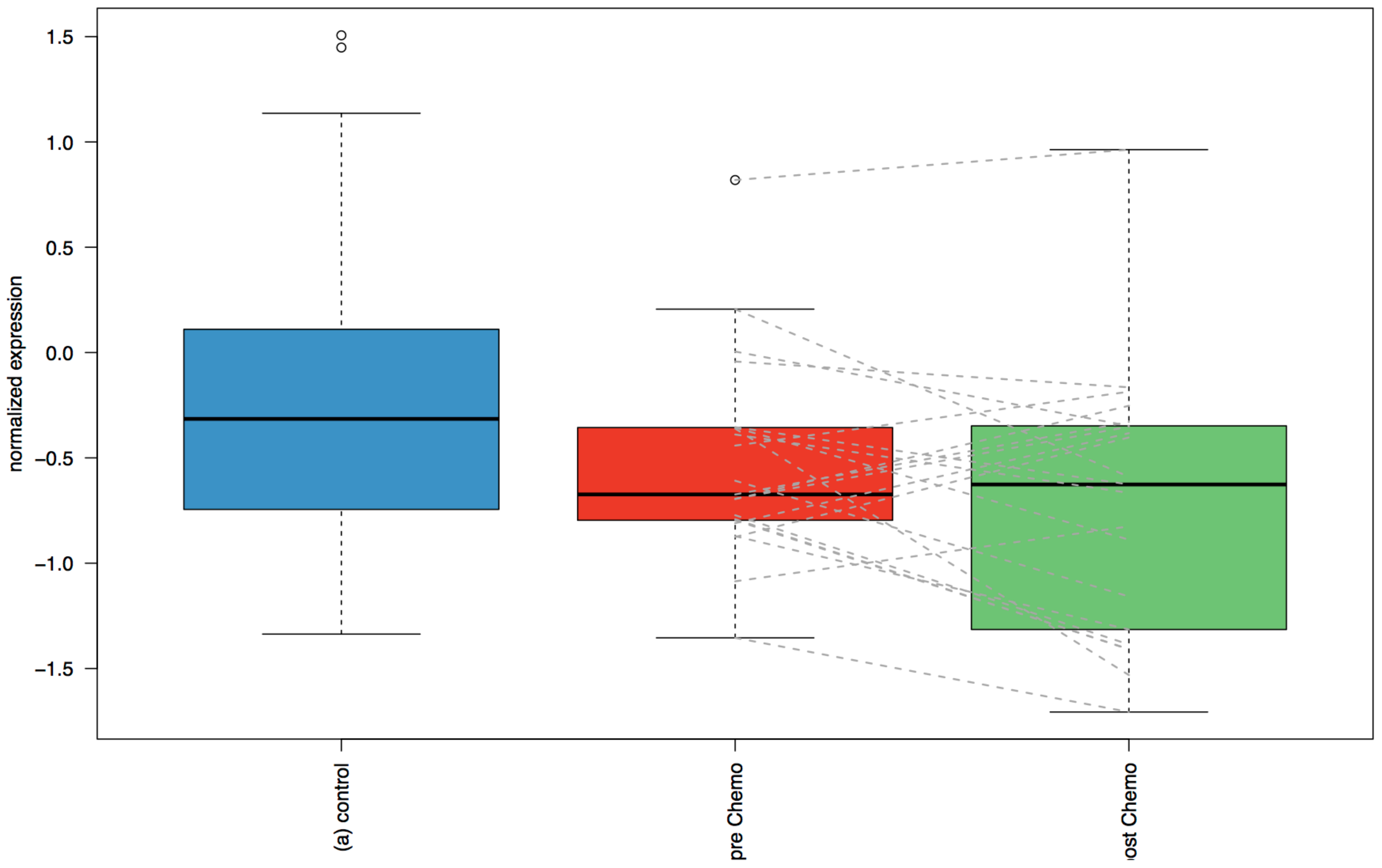

normalized expression
1.5
1.0
0.5
0.0
−0.5
−1.0
−1.5
(a) control
pre Chemo
ost Chemo

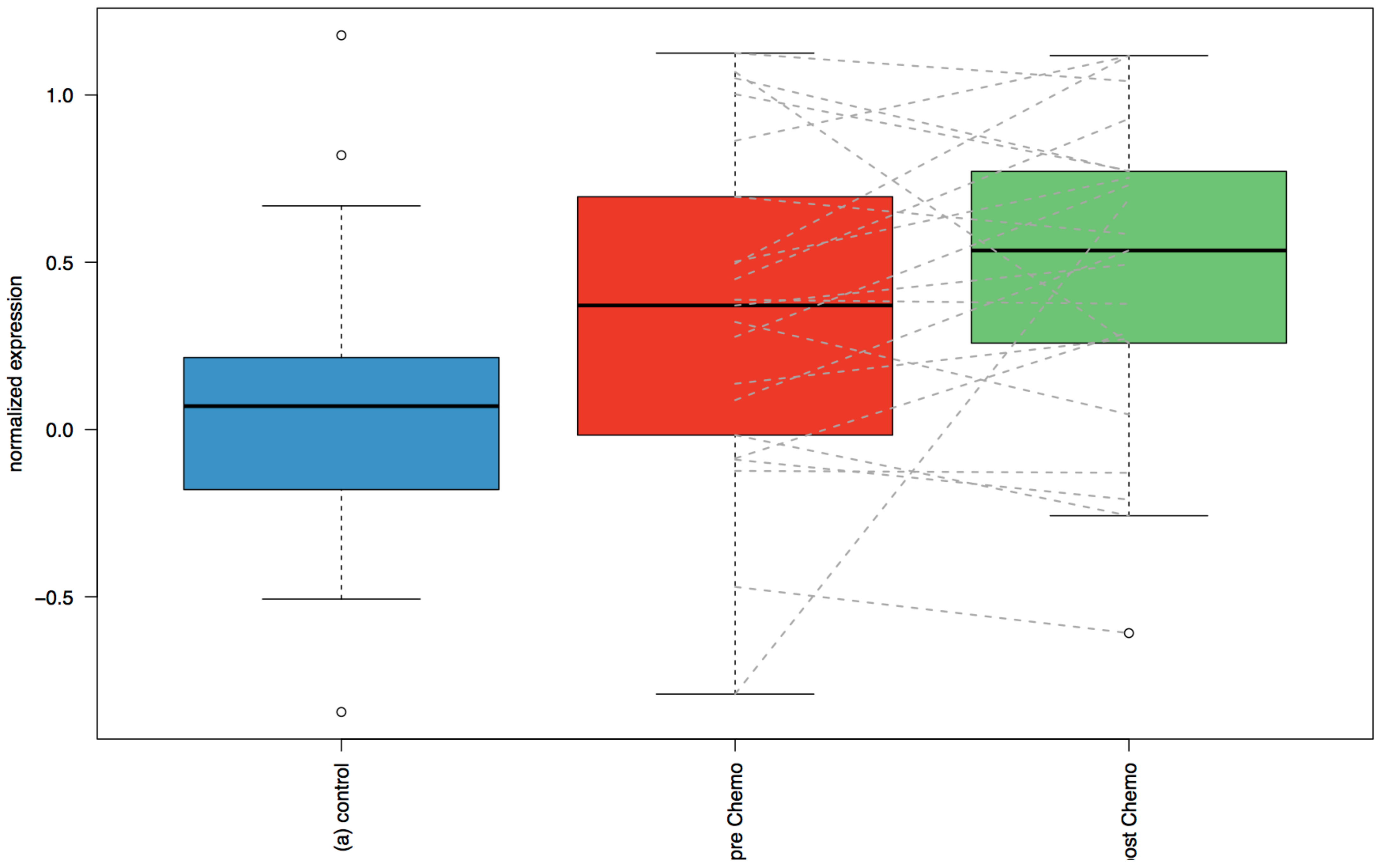

normalized expression
1.0
0.5
0.0
−0.5
(a) control
pre Chemo
ost Chemo

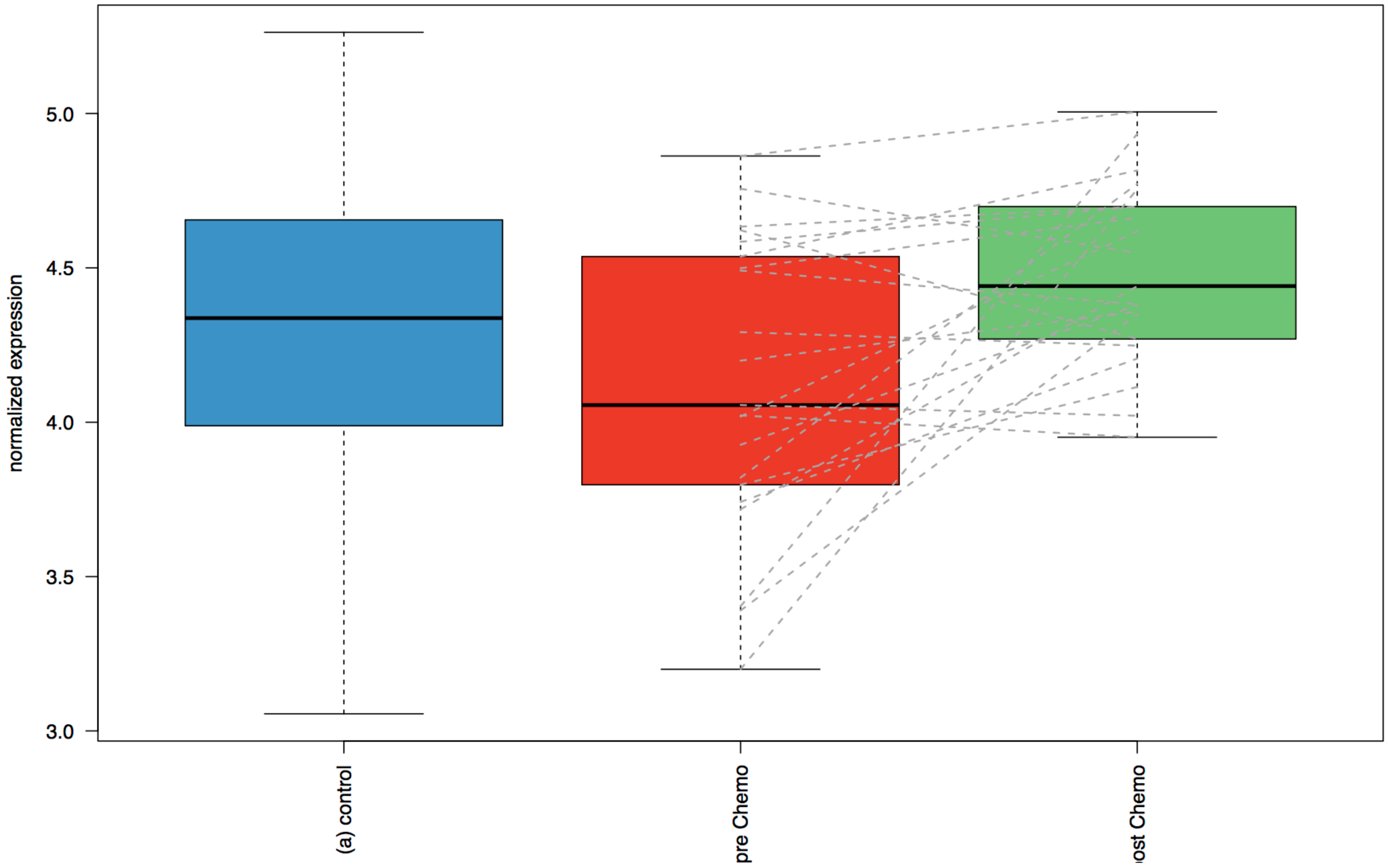
normalized expression
5.0
4.5
4.0
3.5
3.0
(a) control
pre Chemo
ost Chemo

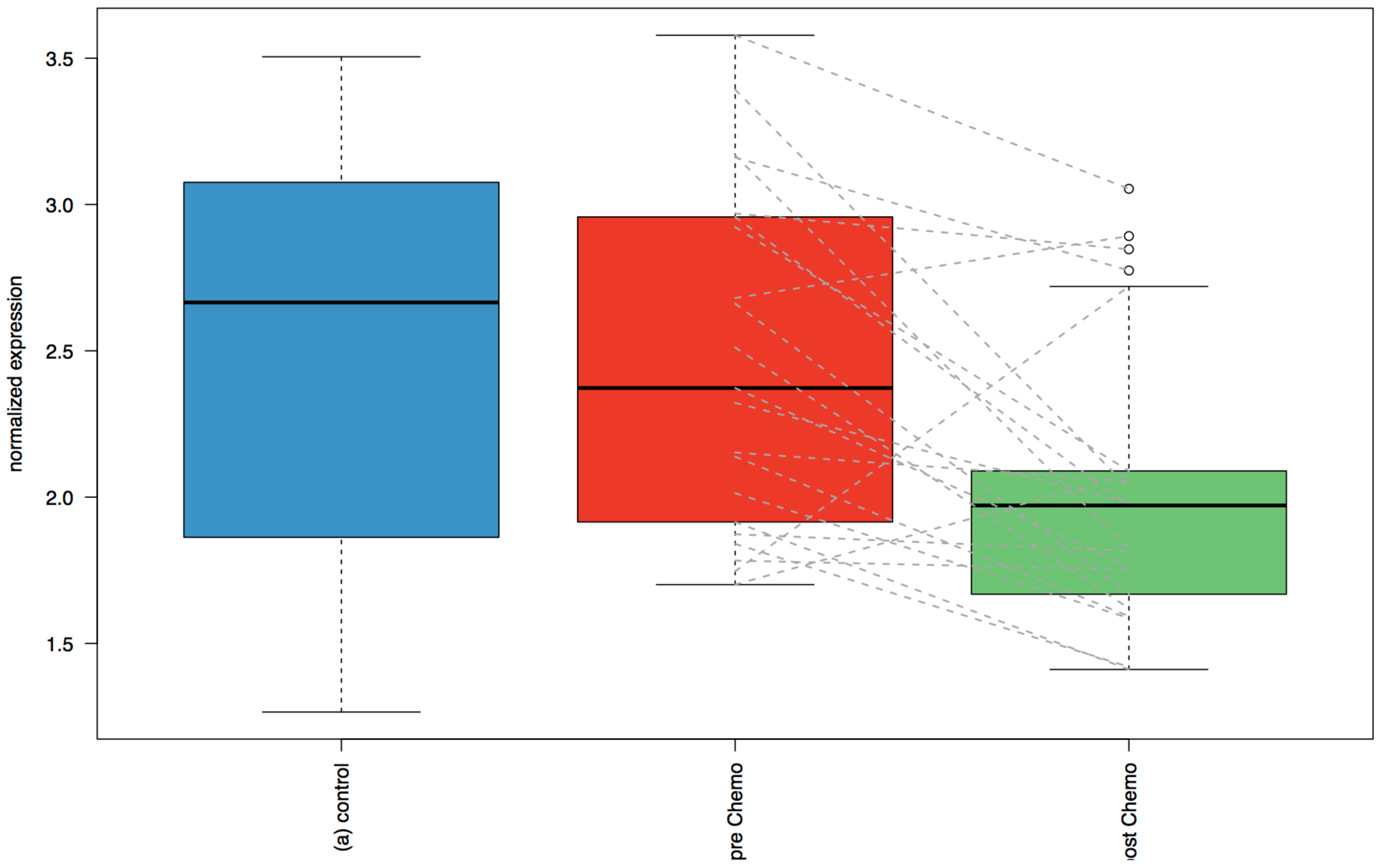
normalized expression
3.5
3.0
2.5
2.0
1.5
(a) control
pre Chemo
ost Chemo

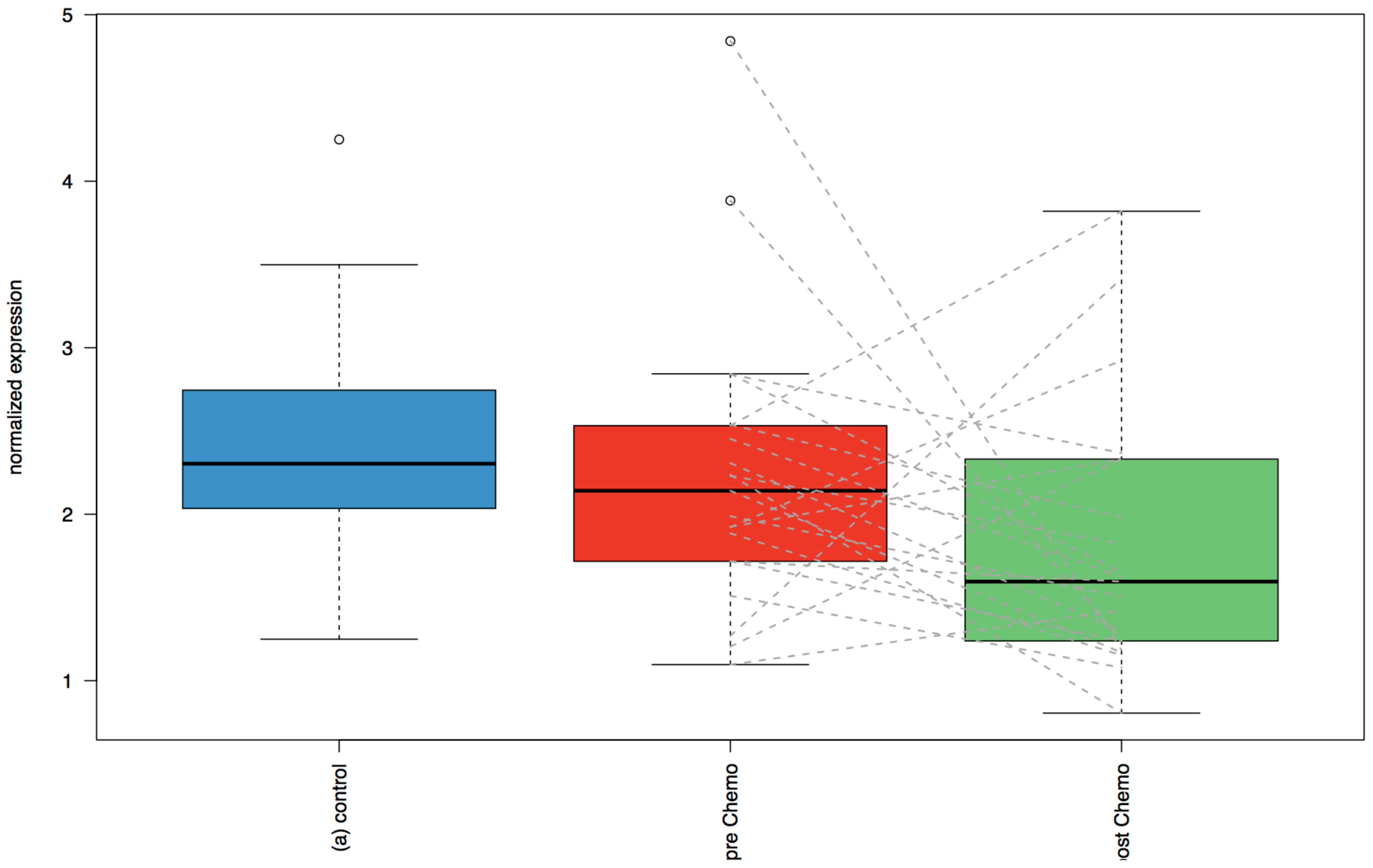
normalized expression
1
2
3
4
5
(a) control
pre Chemo
ost Chemo

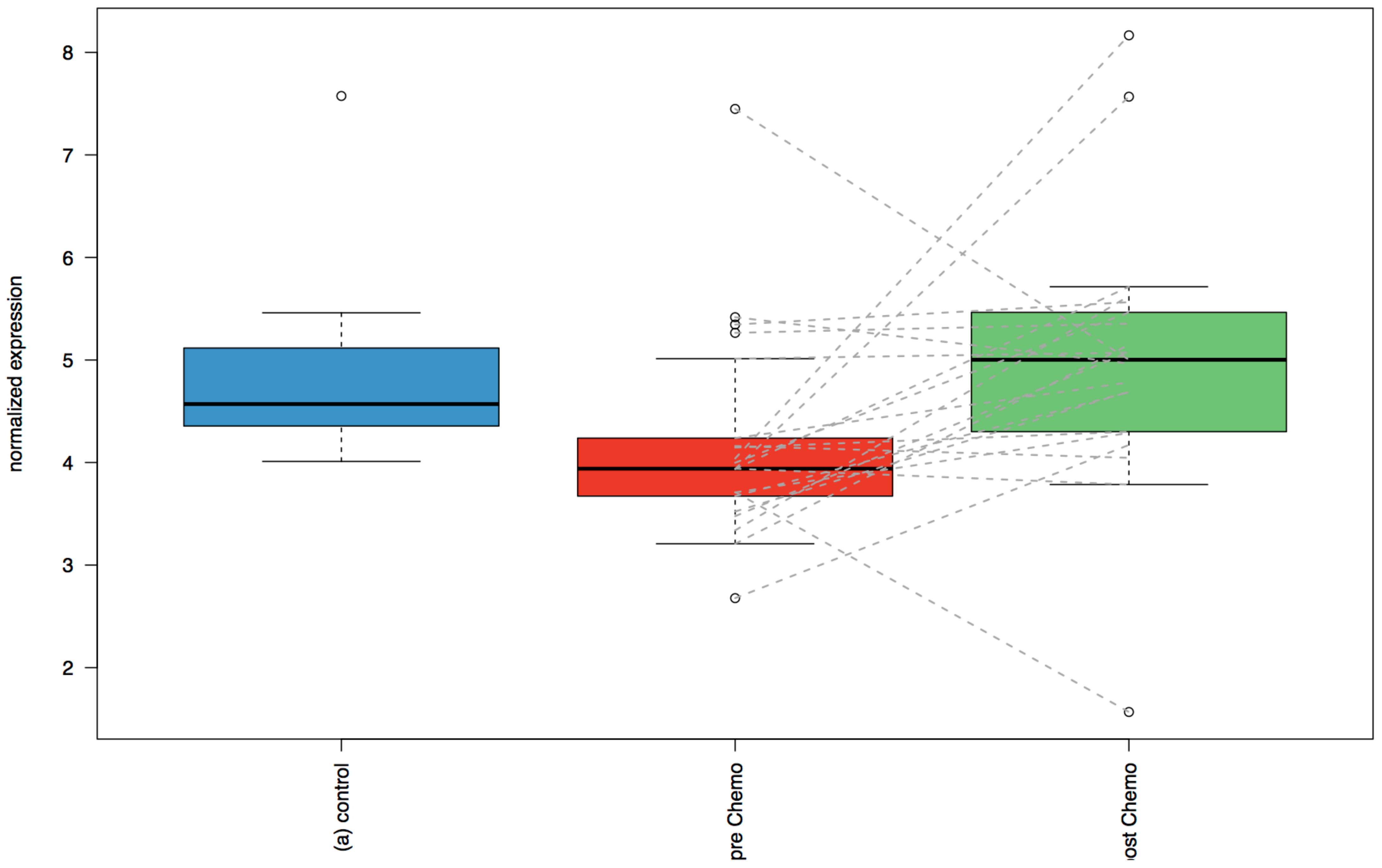
normalized expression
8
7
6
5
4
3
2
(a) control
pre Chemo
ost Chemo

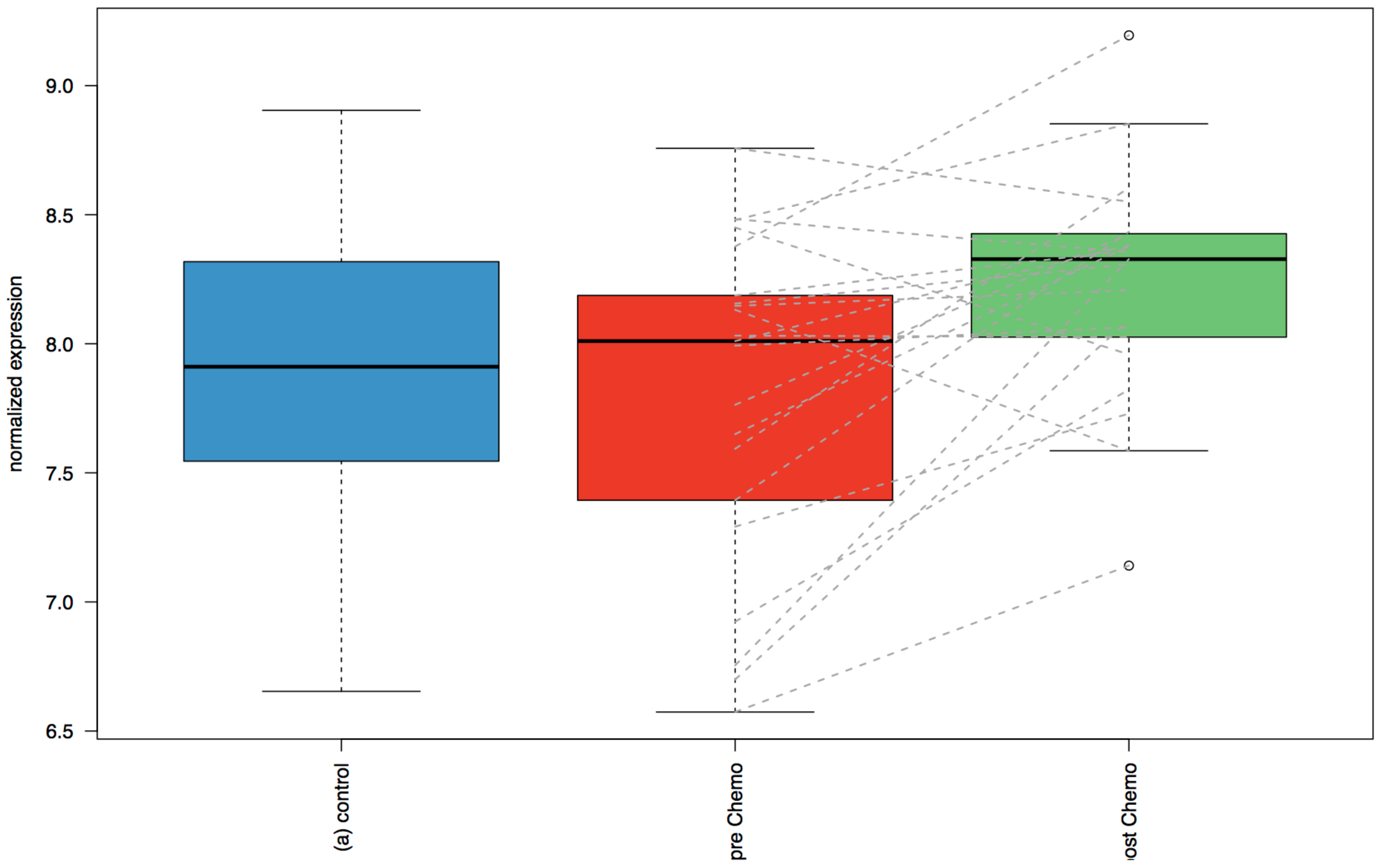
normalized expression
9.0
8.5
8.0
7.5
7.0
6.5
(a) control
pre Chemo
ost Chemo

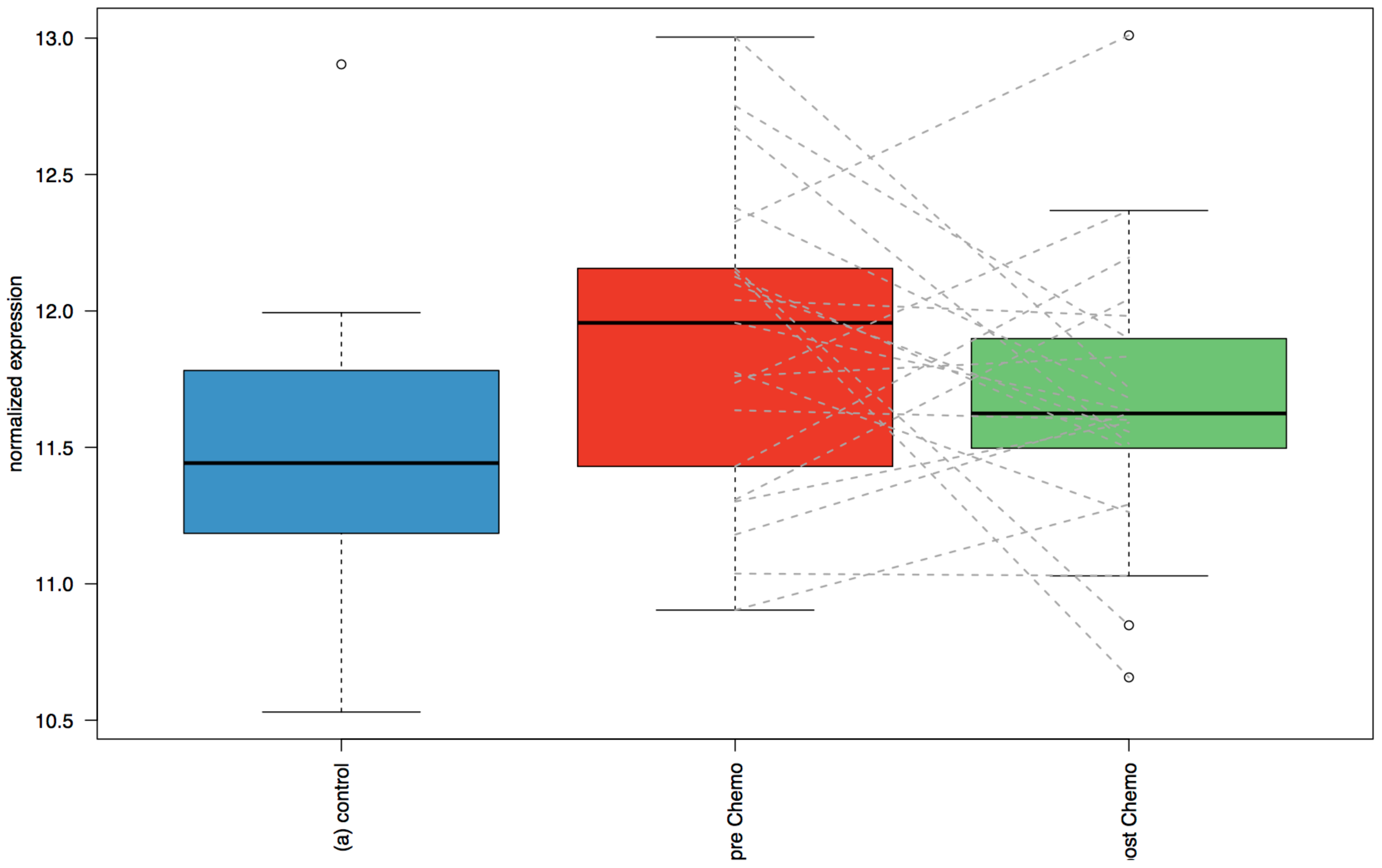

normalized expression
13.0
12.5
12.0
11.5
11.0
10.5
(a) control
pre Chemo
ost Chemo

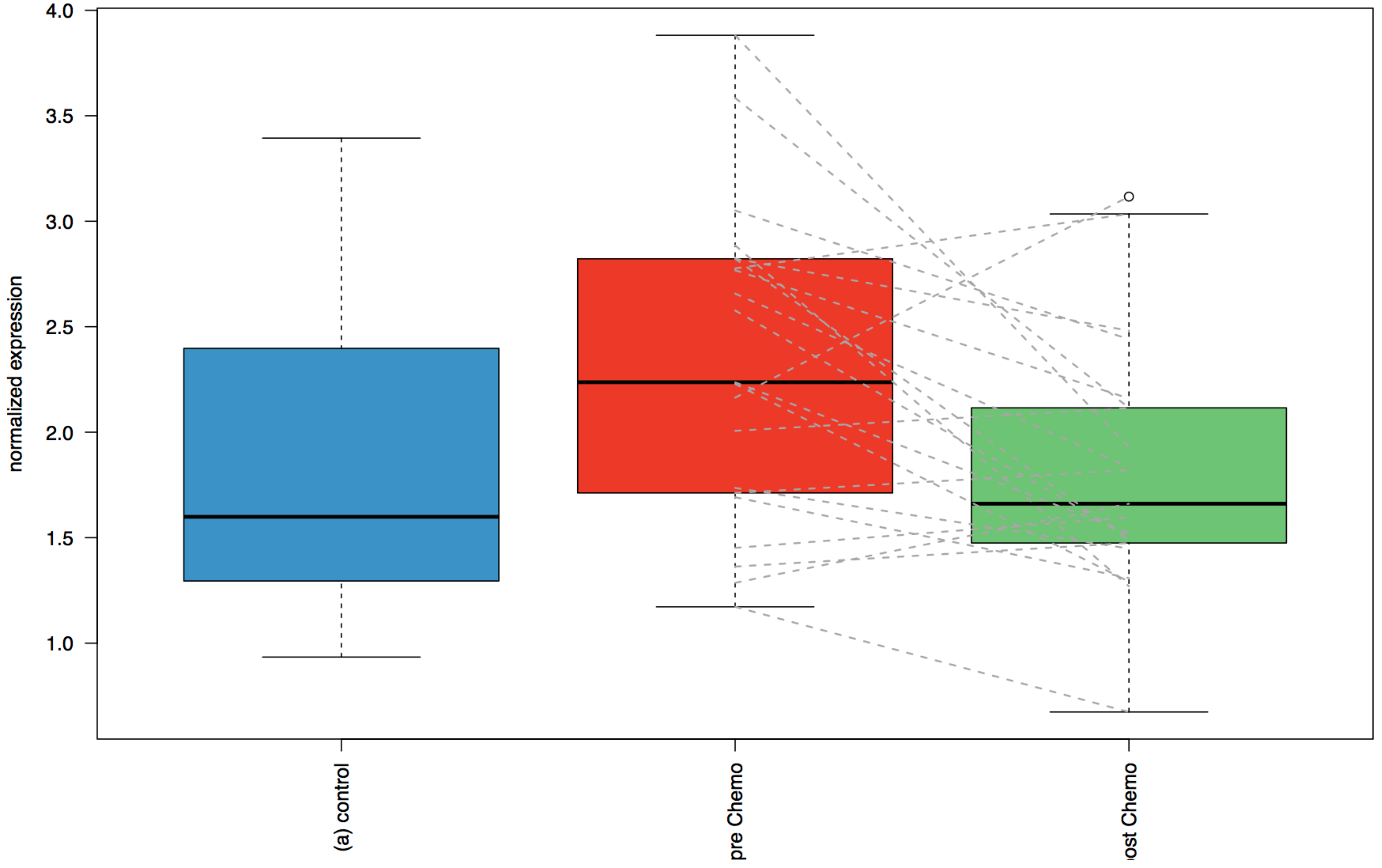
normalized expression
4.0
3.5
3.0
2.5
2.0
1.5
1.0
(a) control
pre Chemo
ost Chemo

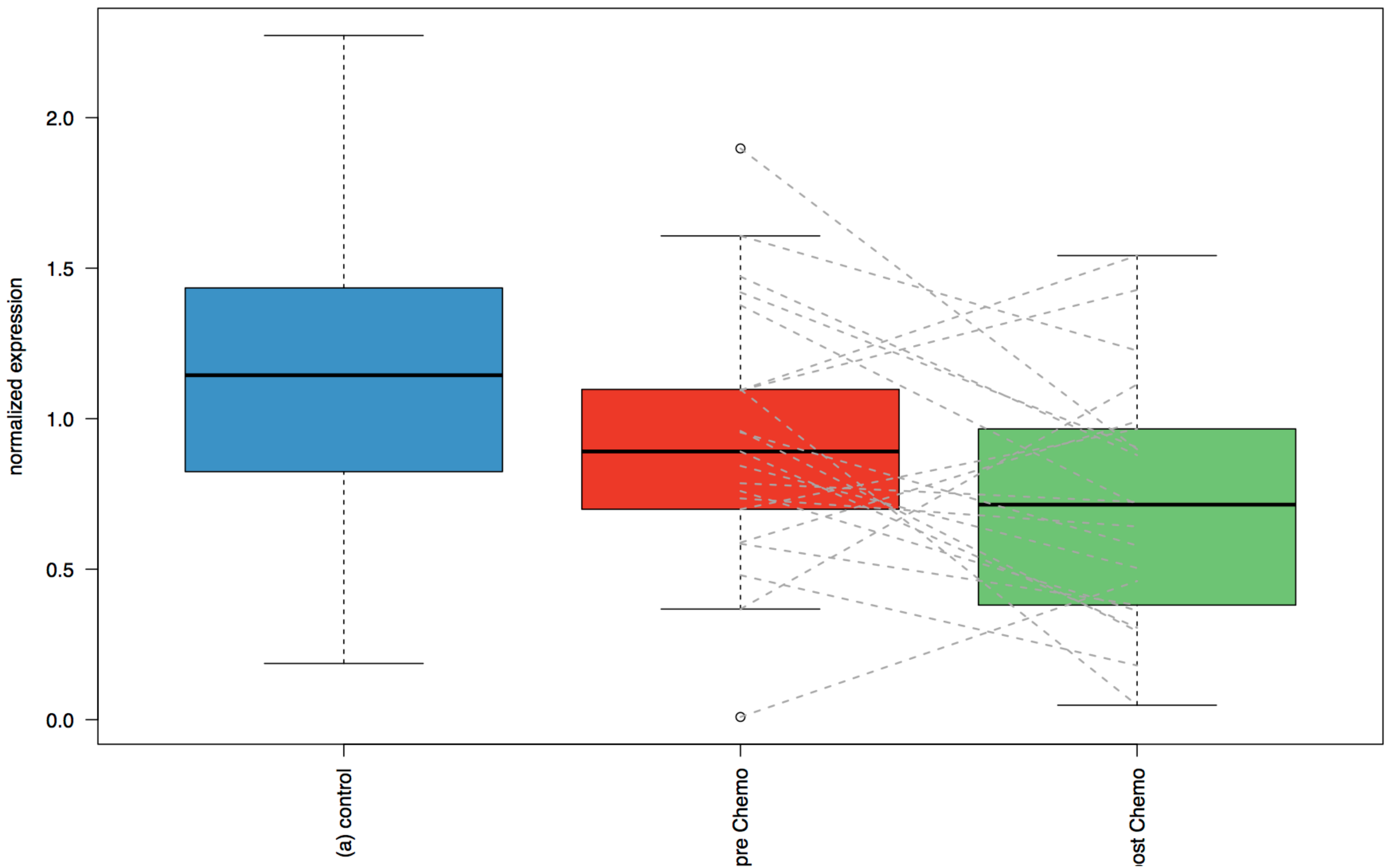

normalized expression
2.0
1.5
1.0
0.5
0.0
(a) control
pre Chemo
ost Chemo

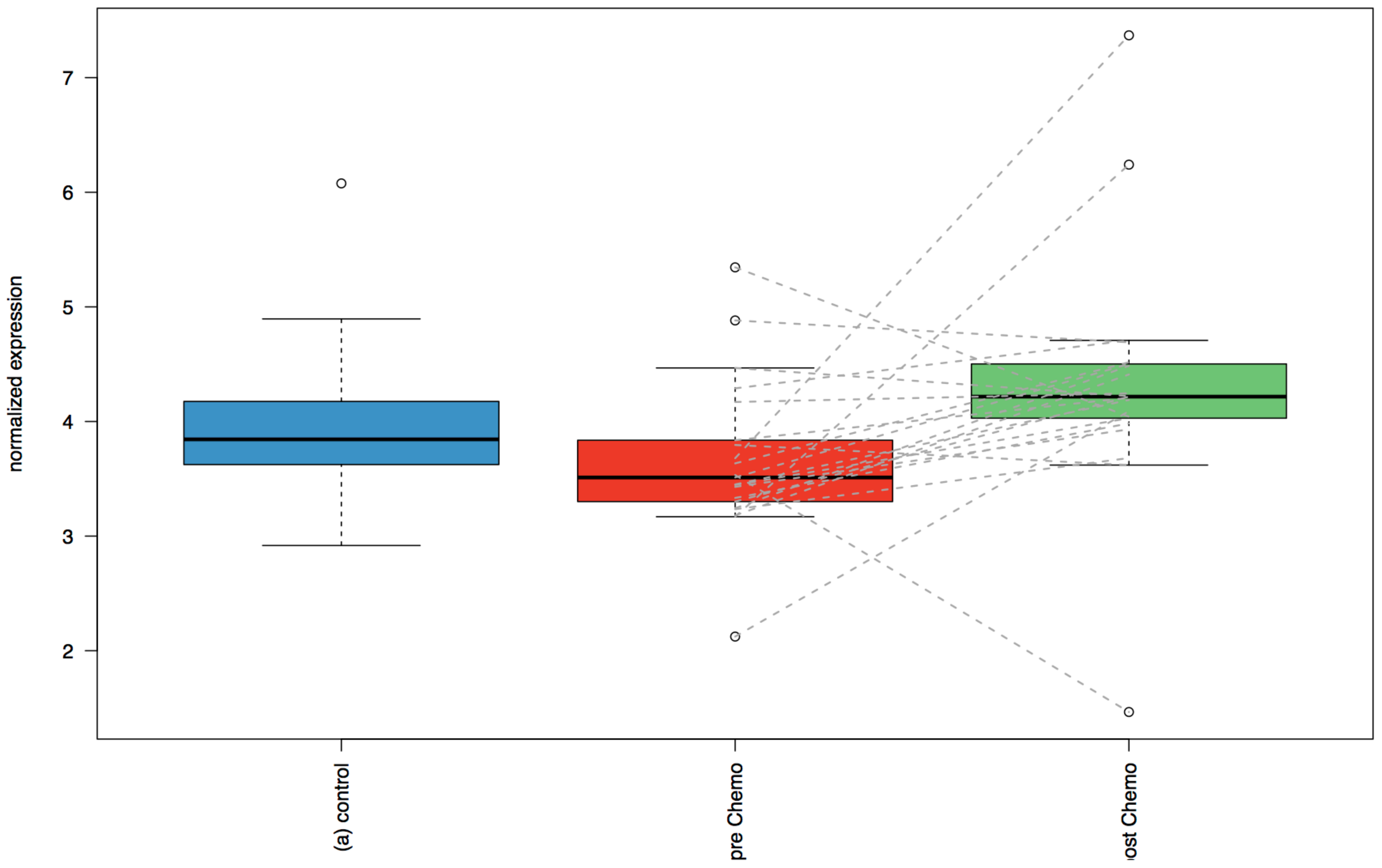
normalized expression
7
6
5
4
3
2
(a) control
pre Chemo
ost Chemo

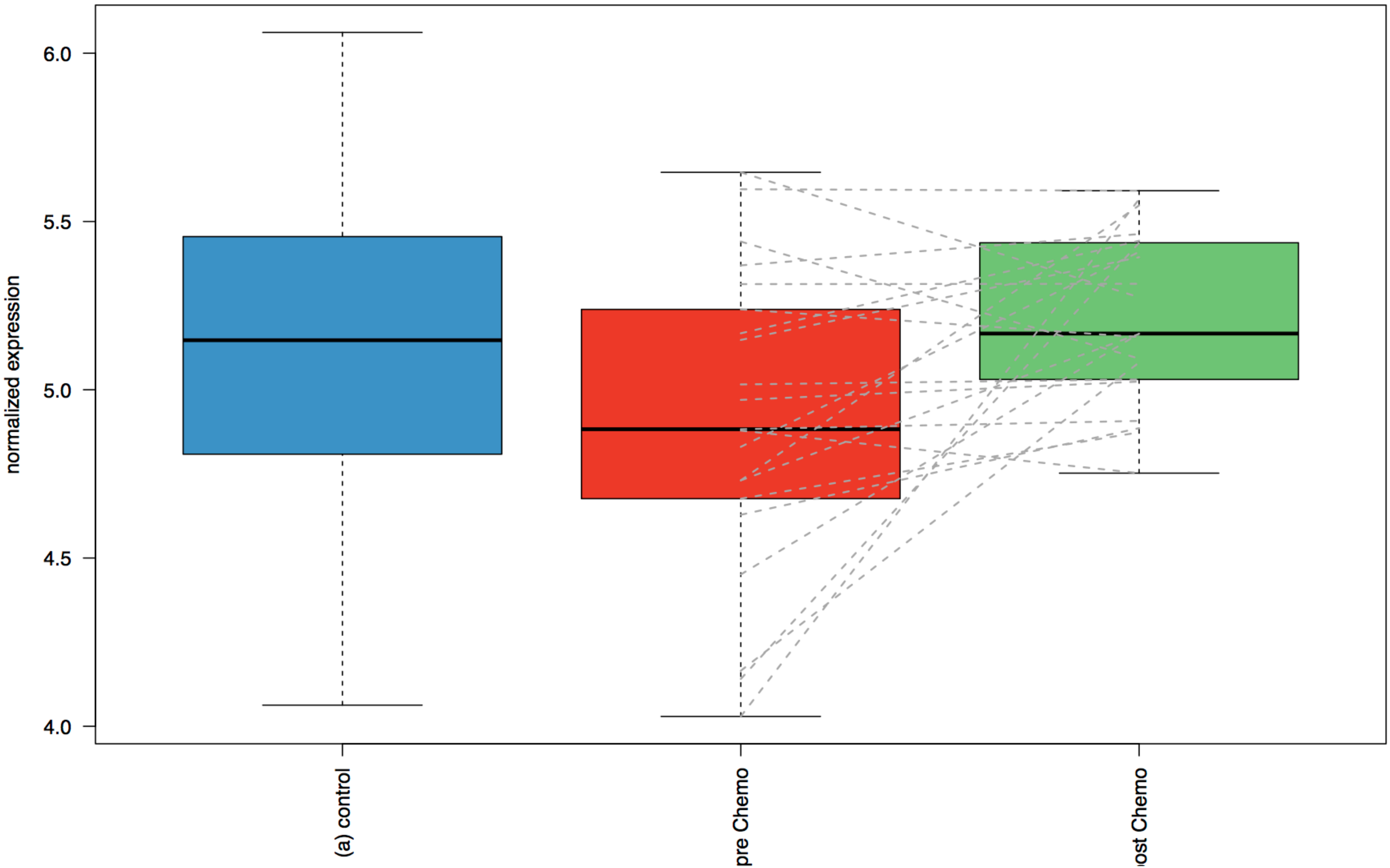
normalized expression
6.0
5.5
5.0
4.5
4.0
(a) control
pre Chemo
ost Chemo

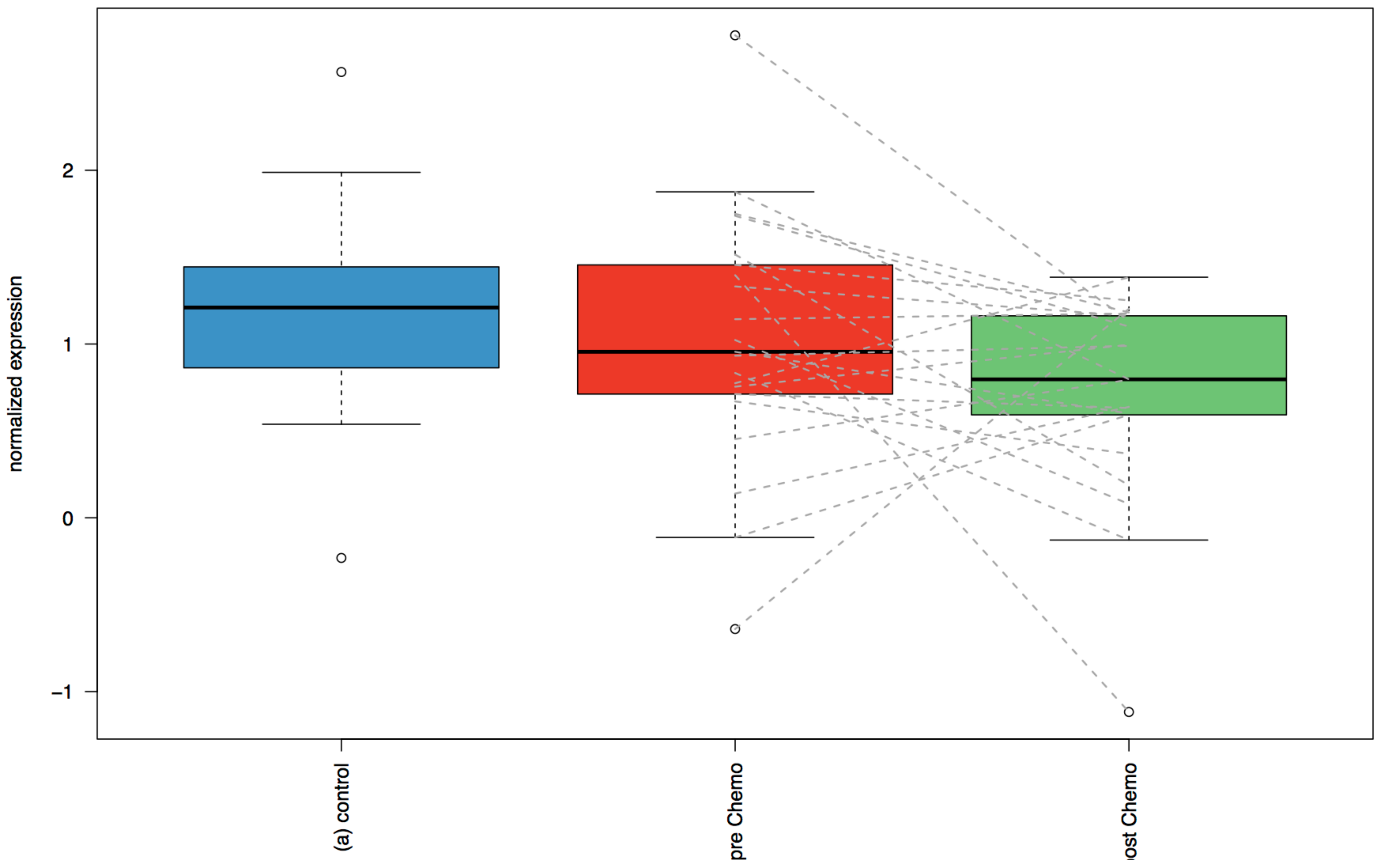
normalized expression
2
1
0
−1
(a) control
pre Chemo
ost Chemo

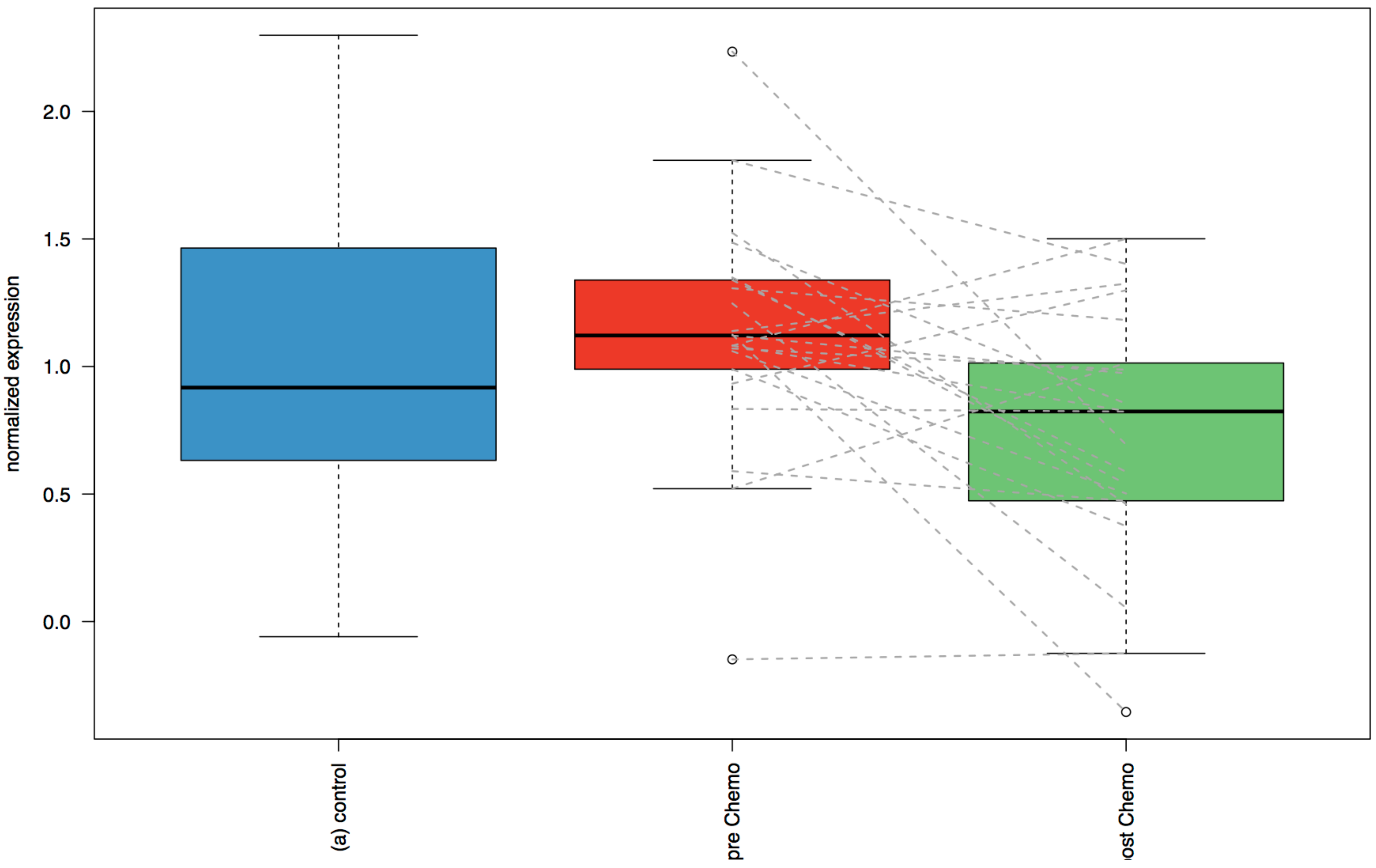

normalized expression
2.0
1.5
1.0
0.5
0.0
(a) control
pre Chemo
ost Chemo

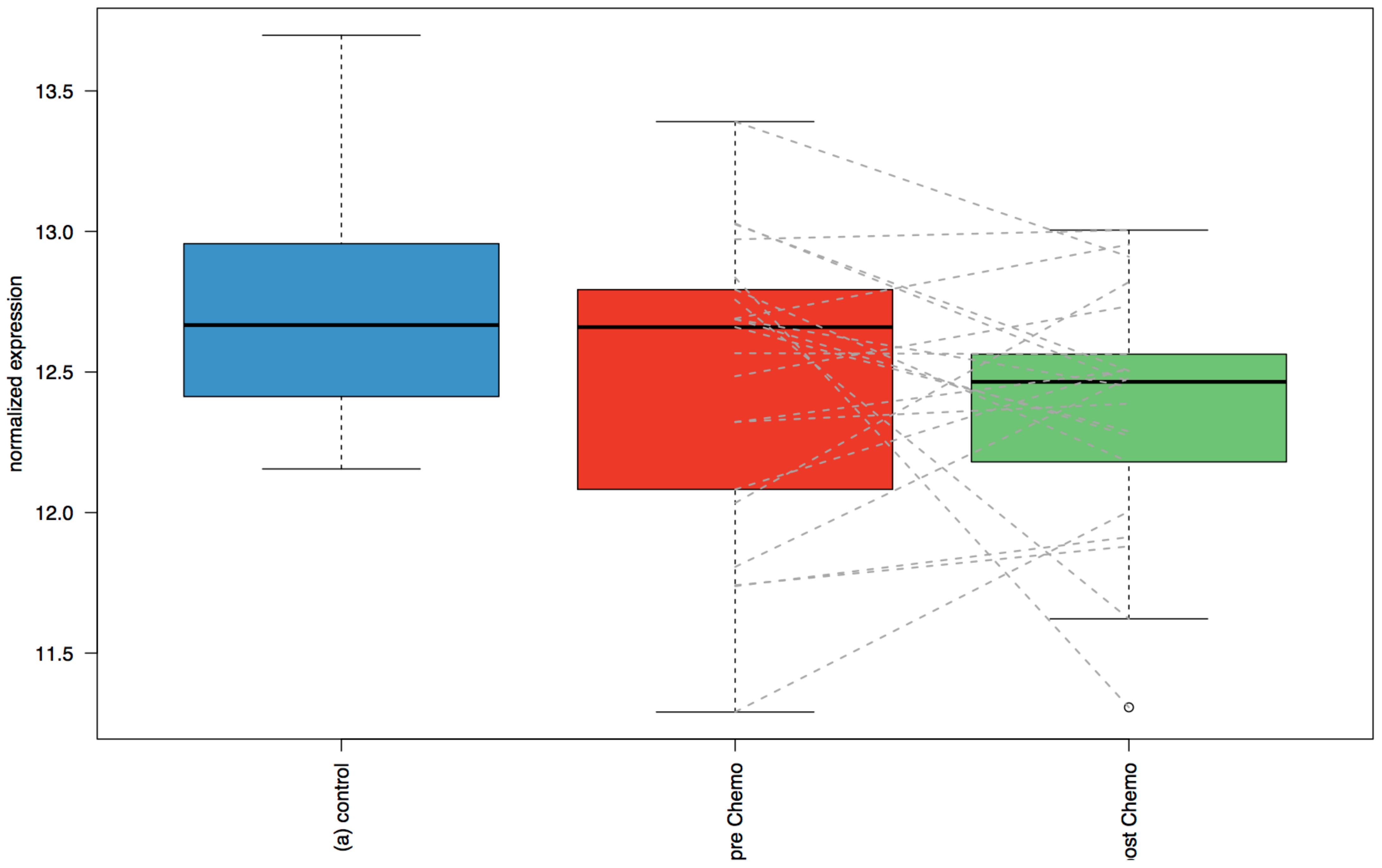
normalized expression
13.5
13.0
12.5
12.0
11.5
(a) control
pre Chemo
ost Chemo

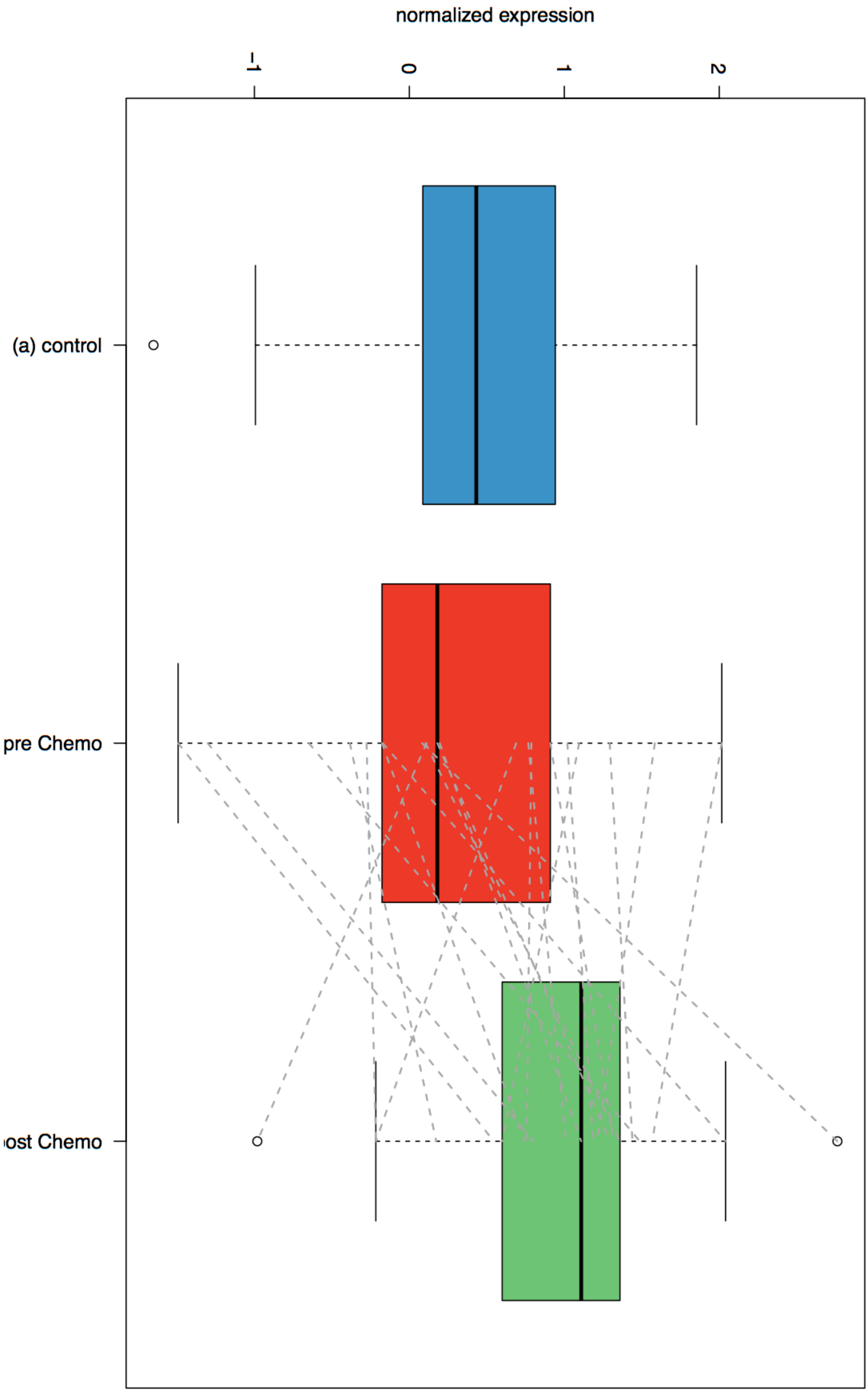
normalized expression
-1
0
1
2
(a) control
pre Chemo
ost Chemo

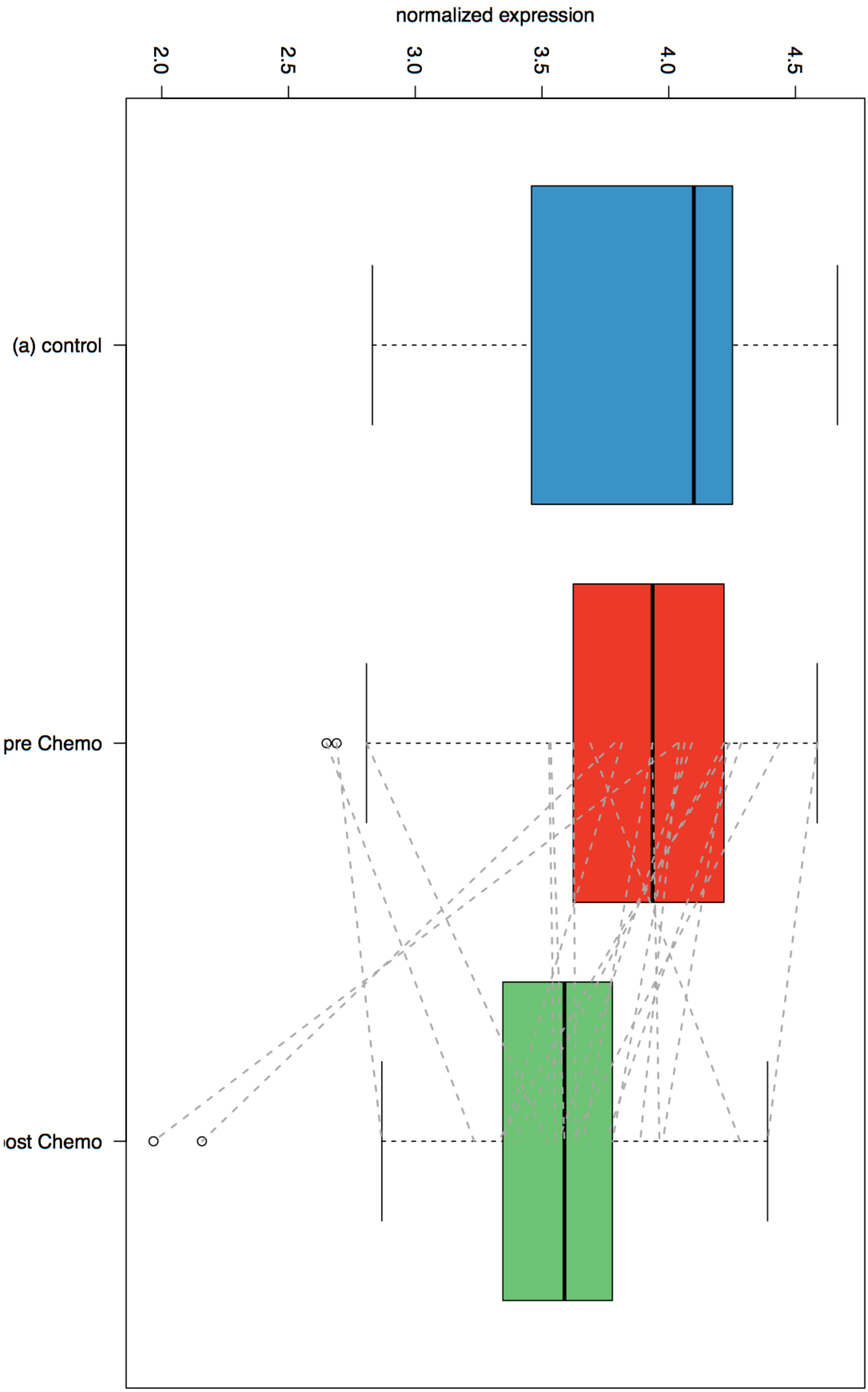
normalized expression
2.0
2.5
3.0
3.5
4.0
4.5
(a) control
pre Chemo
ost Chemo

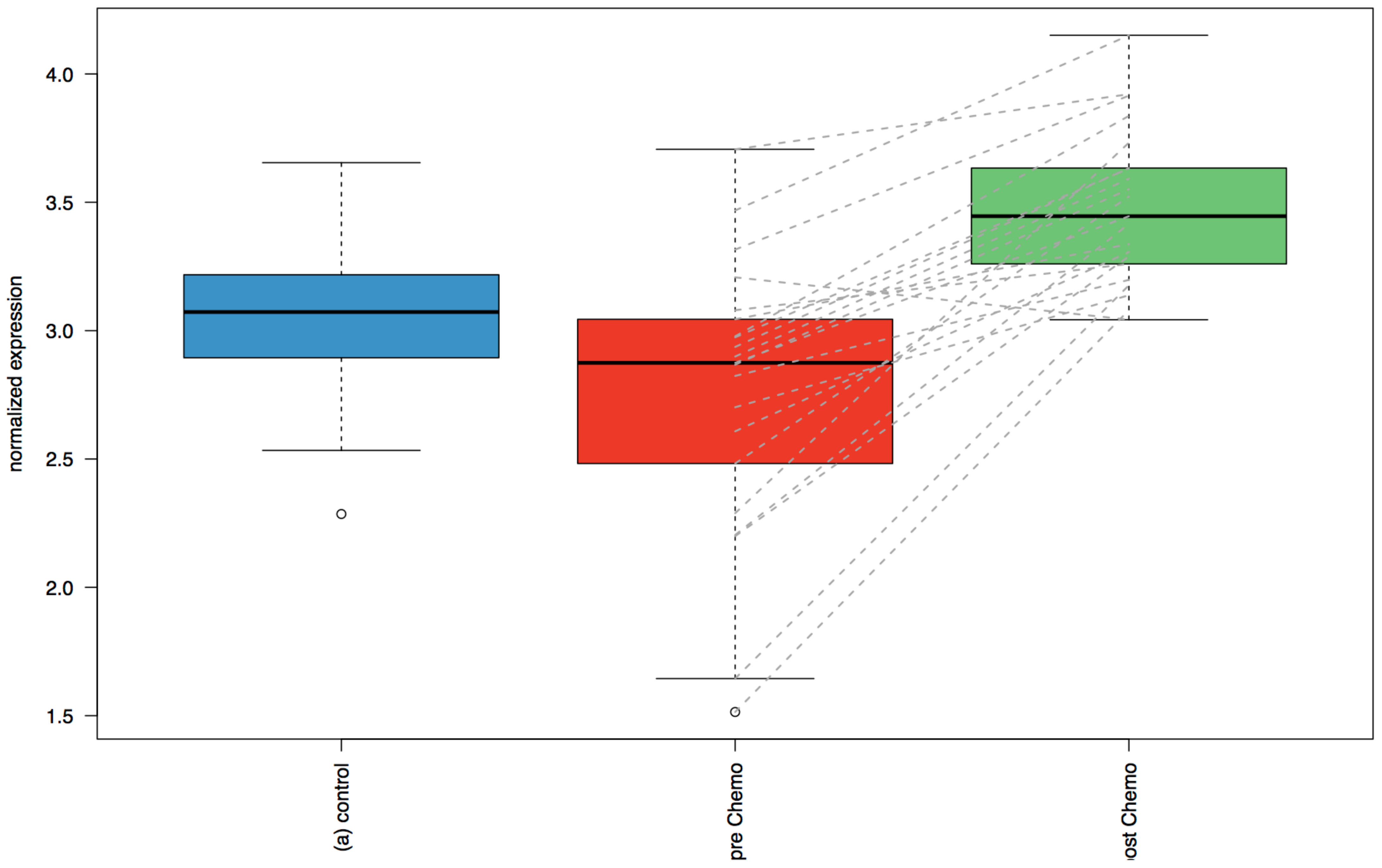
normalized expression
4.0
3.5
3.0
2.5
2.0
1.5
(a) control
pre Chemo
ost Chemo

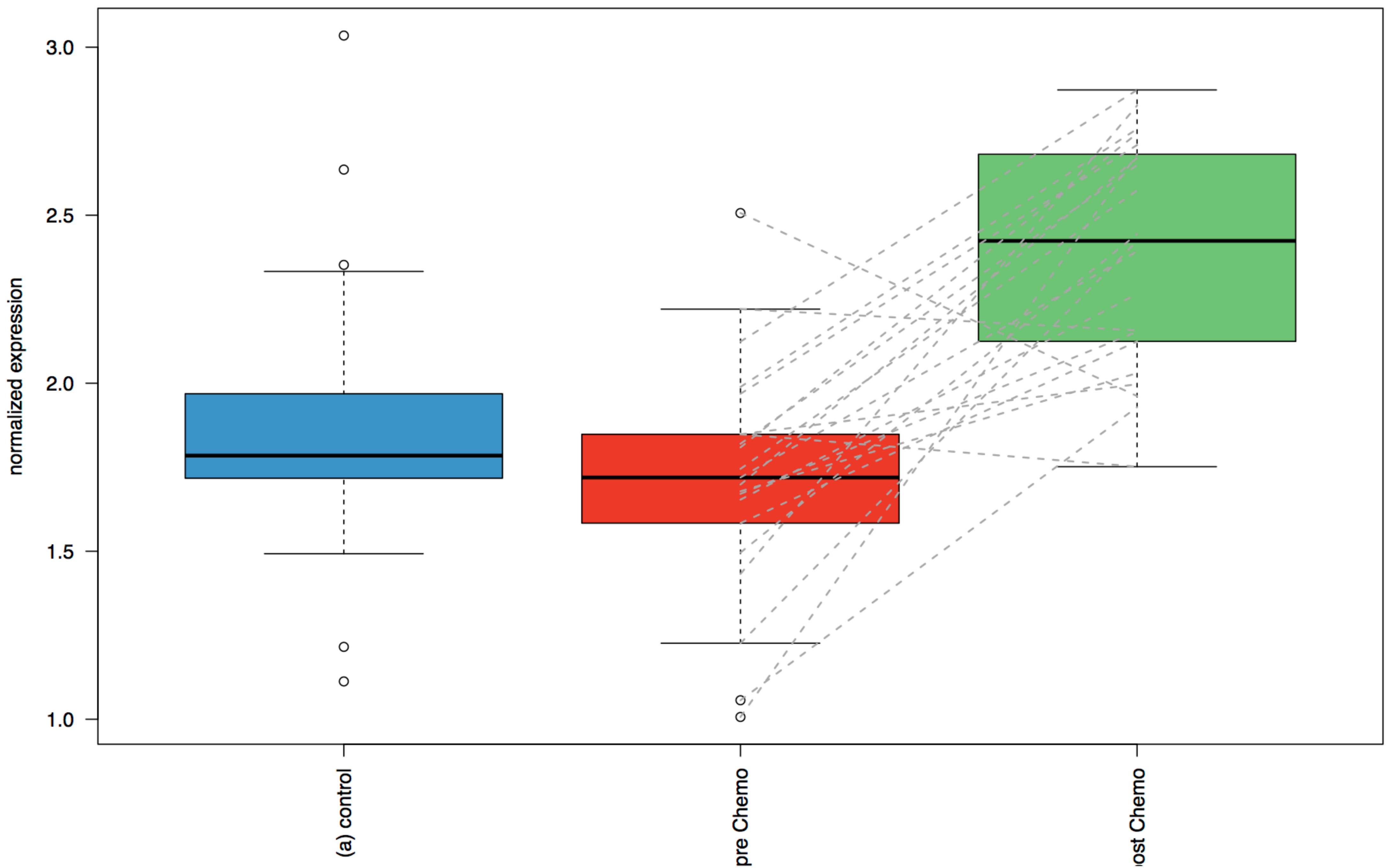
normalized expression
3.0
2.5
2.0
1.5
1.0
(a) control
pre Chemo
ost Chemo

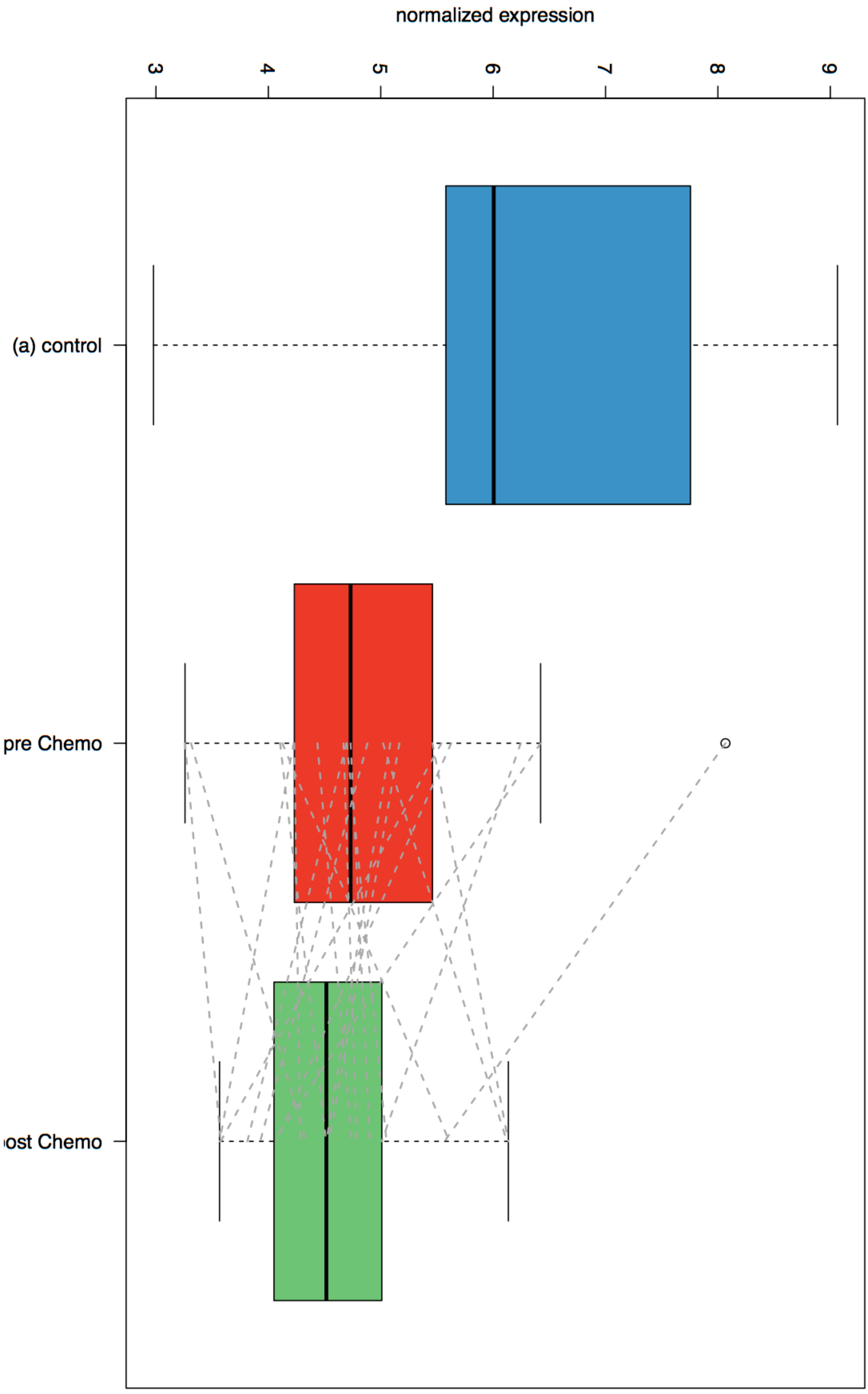
normalized expression
3
4
5
6
7
8
9
(a) control
pre Chemo
ost Chemo

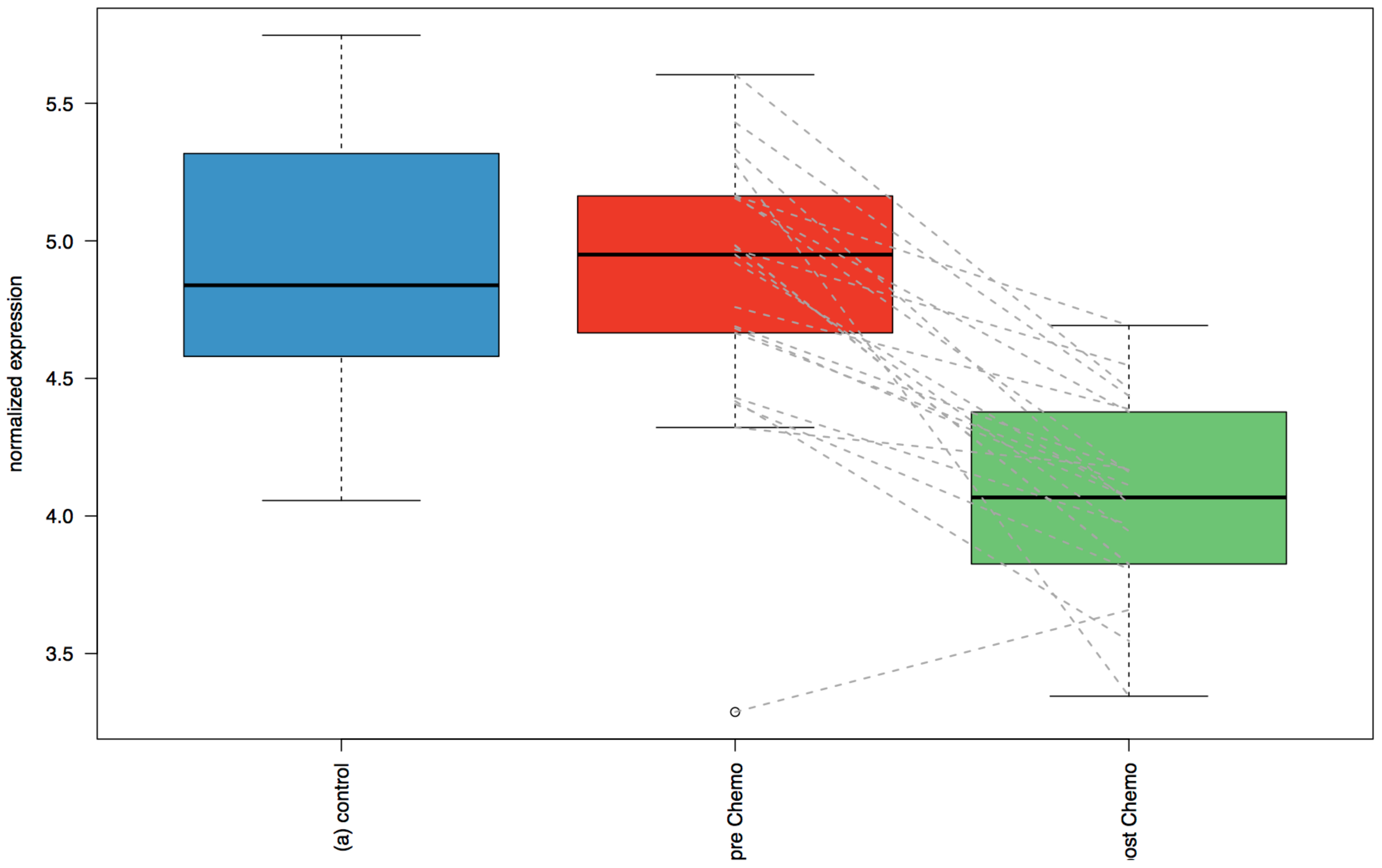
normalized expression
5.5
5.0
4.5
4.0
3.5
(a) control
pre Chemo
ost Chemo

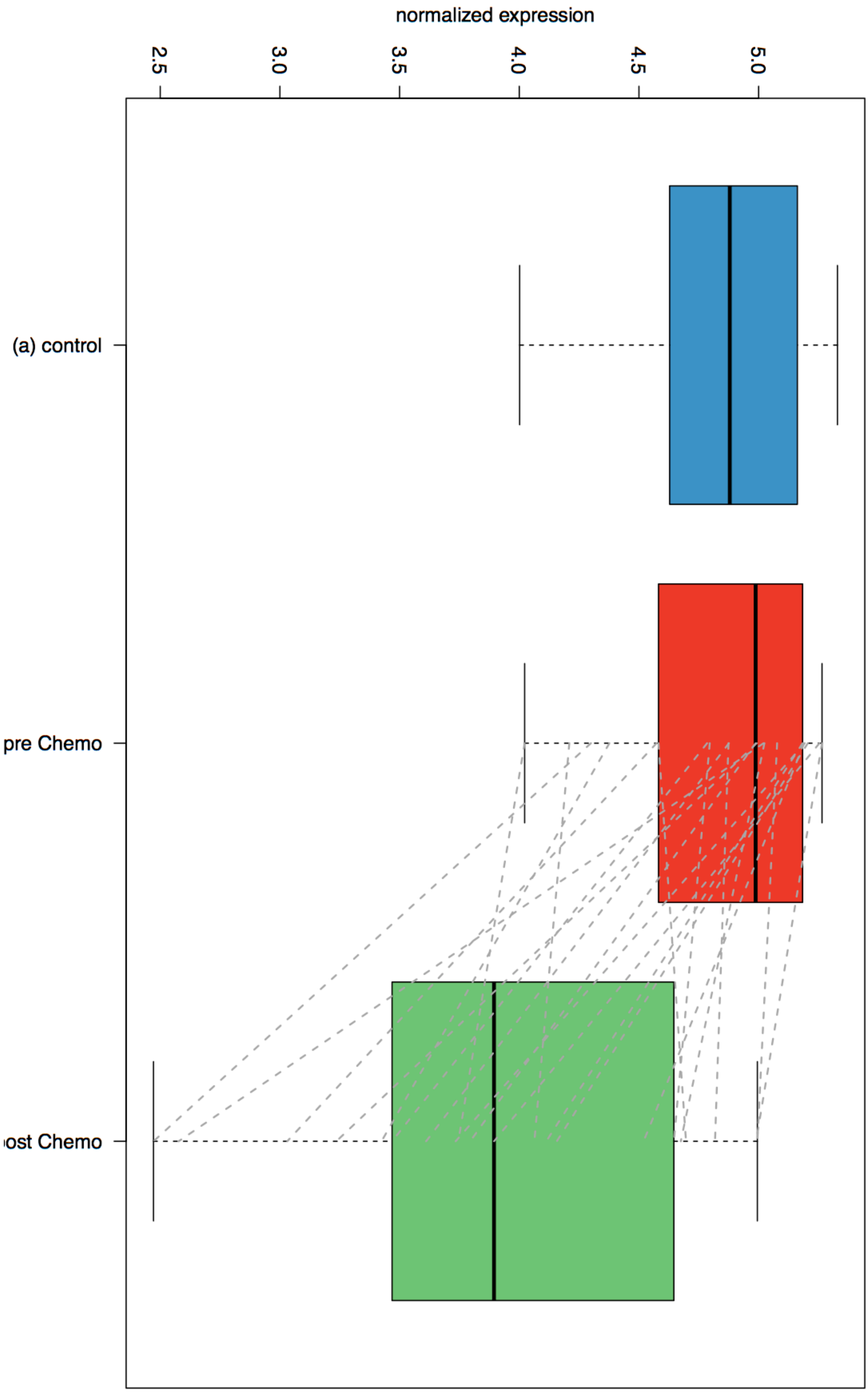
normalized expression
2.5
3.0
3.5
4.0
4.5
5.0
(a) control
pre Chemo
ost Chemo

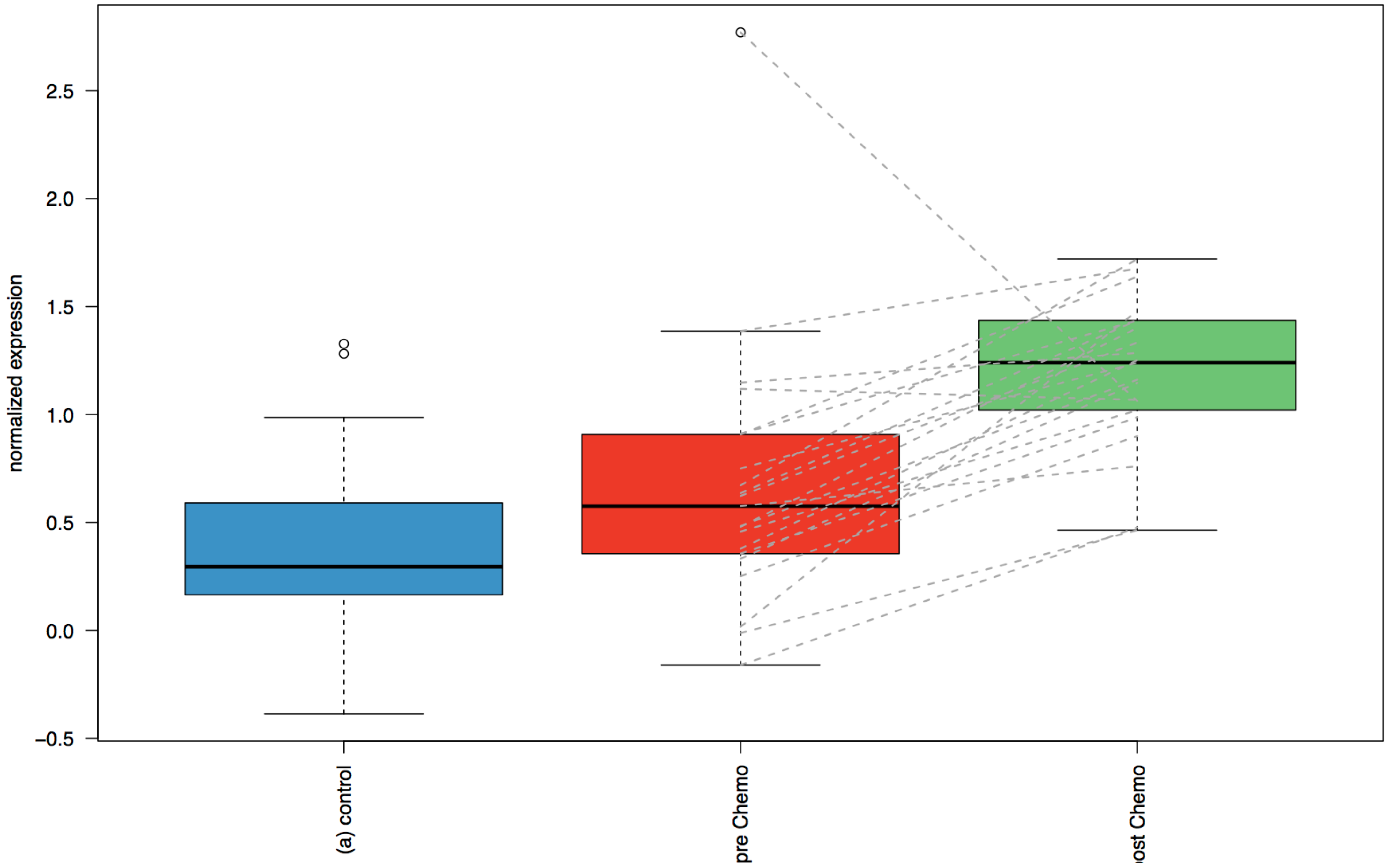

normalized expression
2.5
2.0
1.5
1.0
0.5
0.0
−0.5
(a) control
pre Chemo
ost Chemo

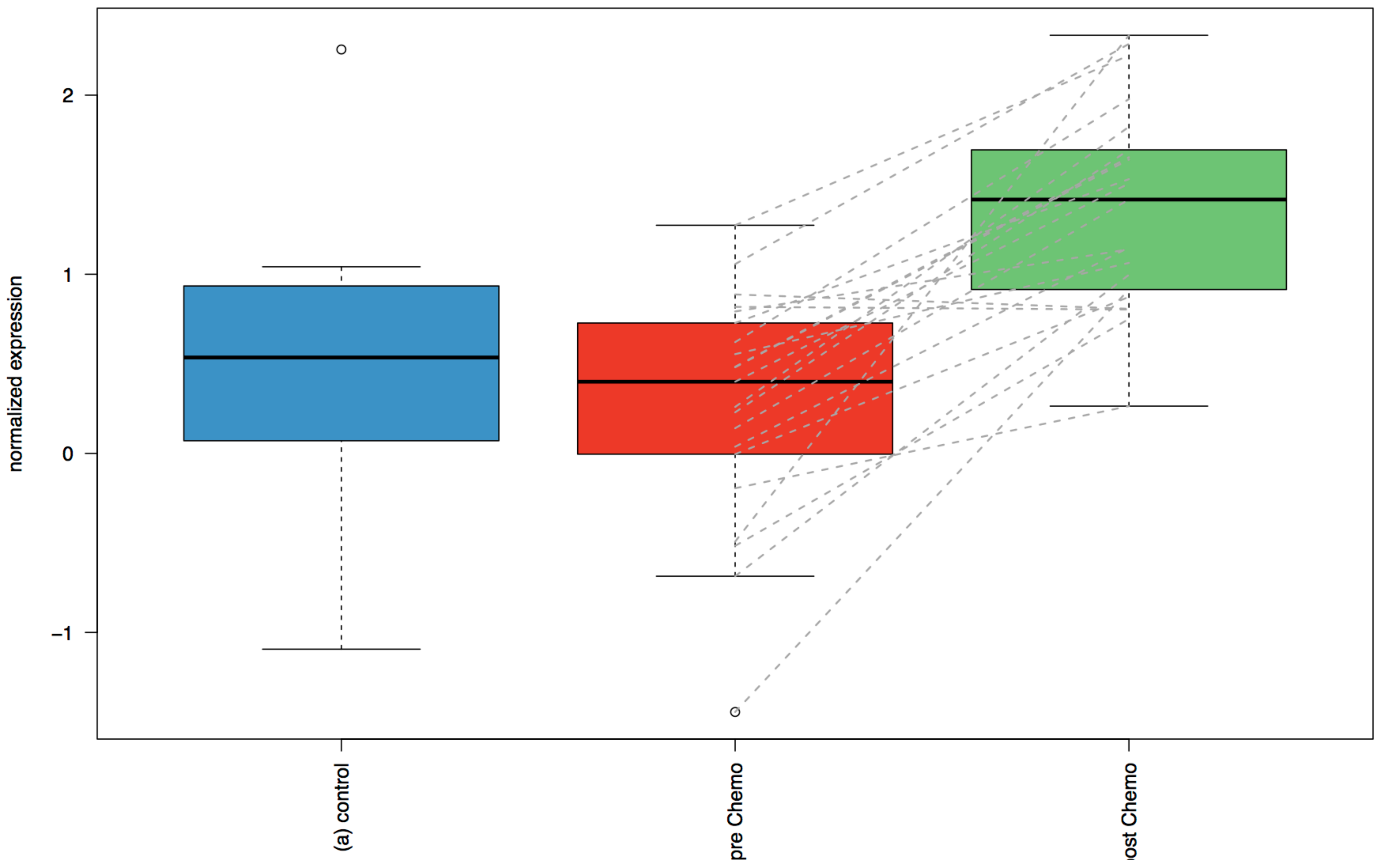
normalized expression
2
1
0
−1
(a) control
pre Chemo
ost Chemo

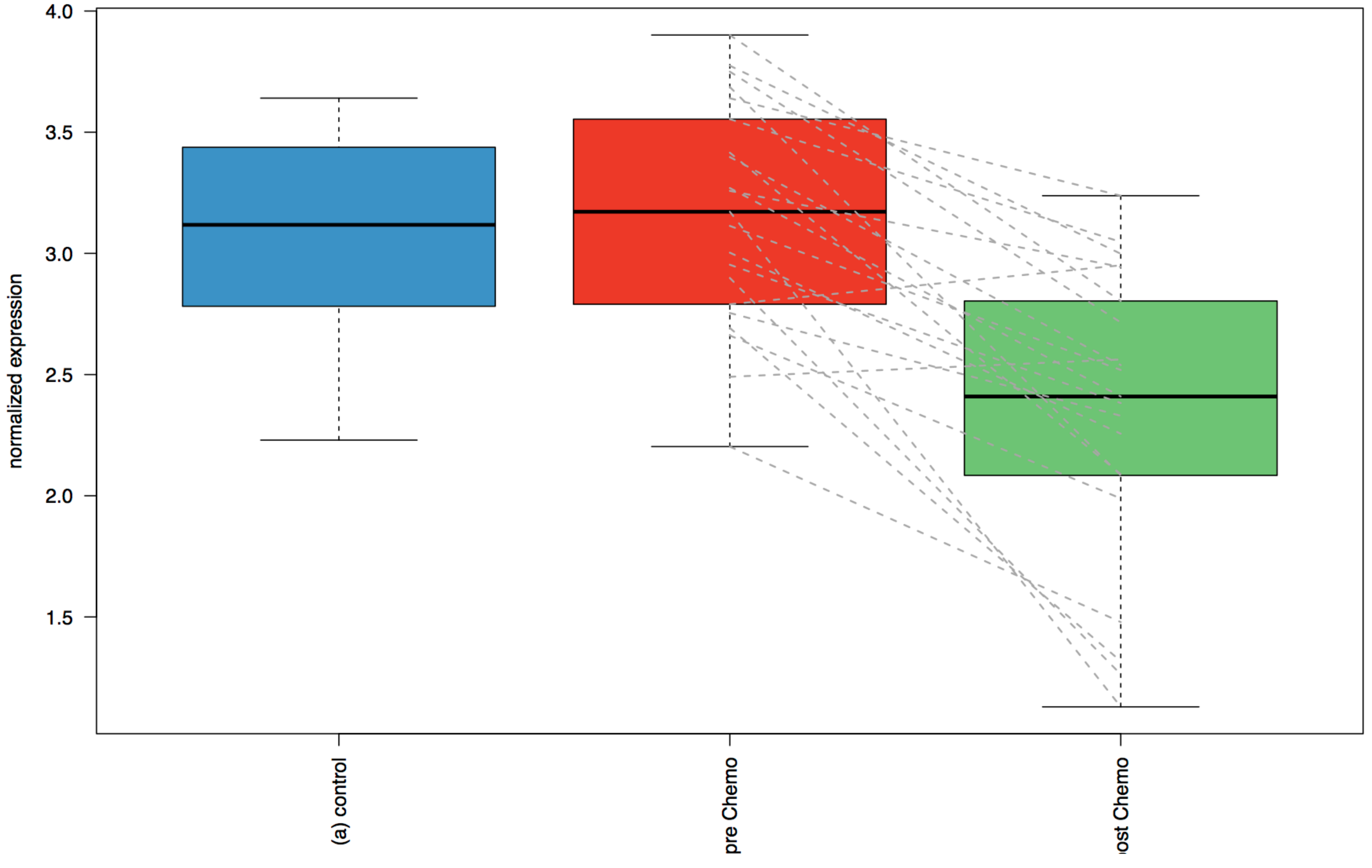
normalized expression
4.0
3.5
3.0
2.5
2.0
1.5
(a) control
pre Chemo
ost Chemo

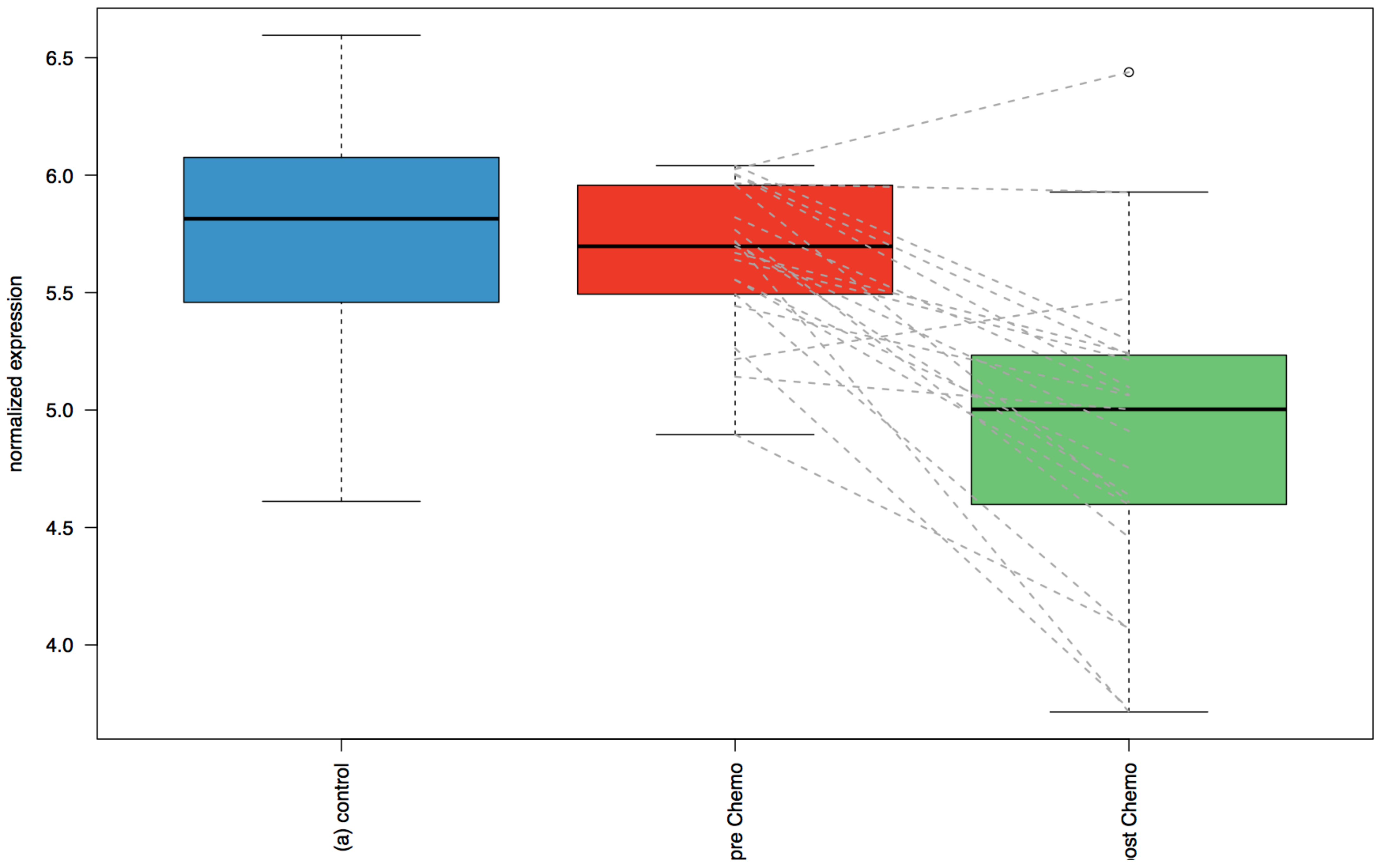
normalized expression
6.5
6.0
5.5
5.0
4.5
4.0
(a) control
pre Chemo
ost Chemo

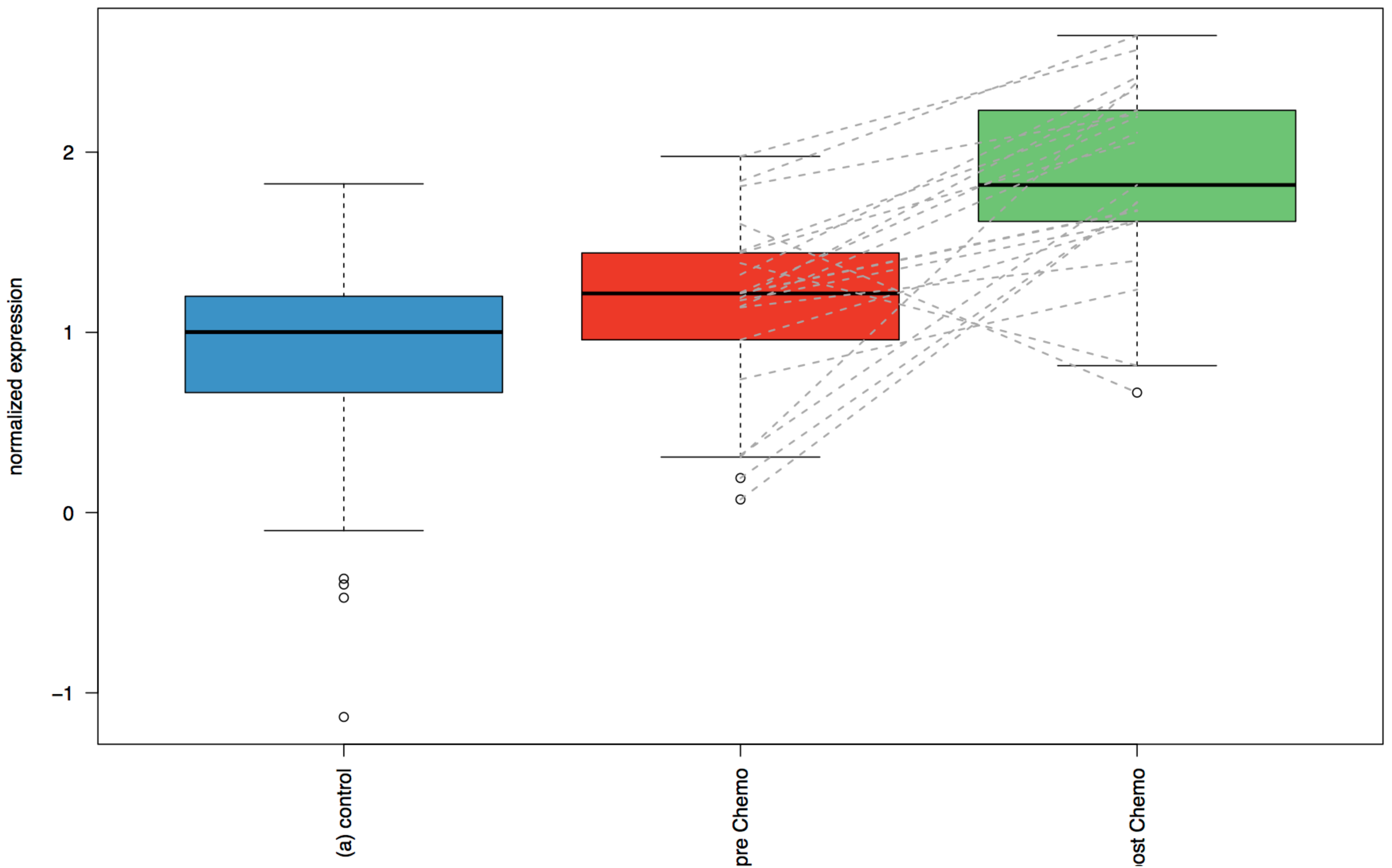

normalized expression
2
1
0
−1
(a) control
pre Chemo
ost Chemo

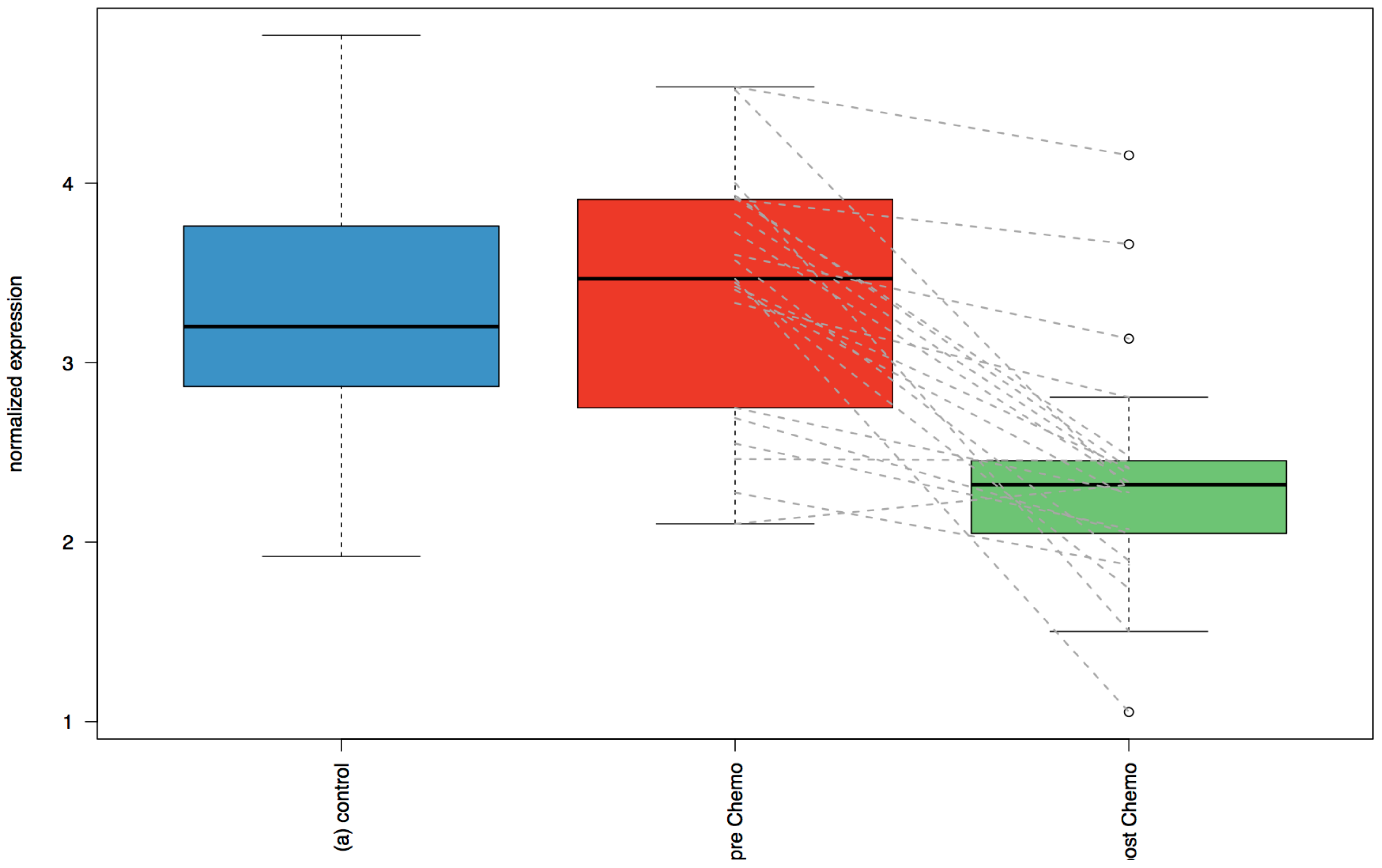
normalized expression
1
2
3
4
(a) control
pre Chemo
ost Chemo

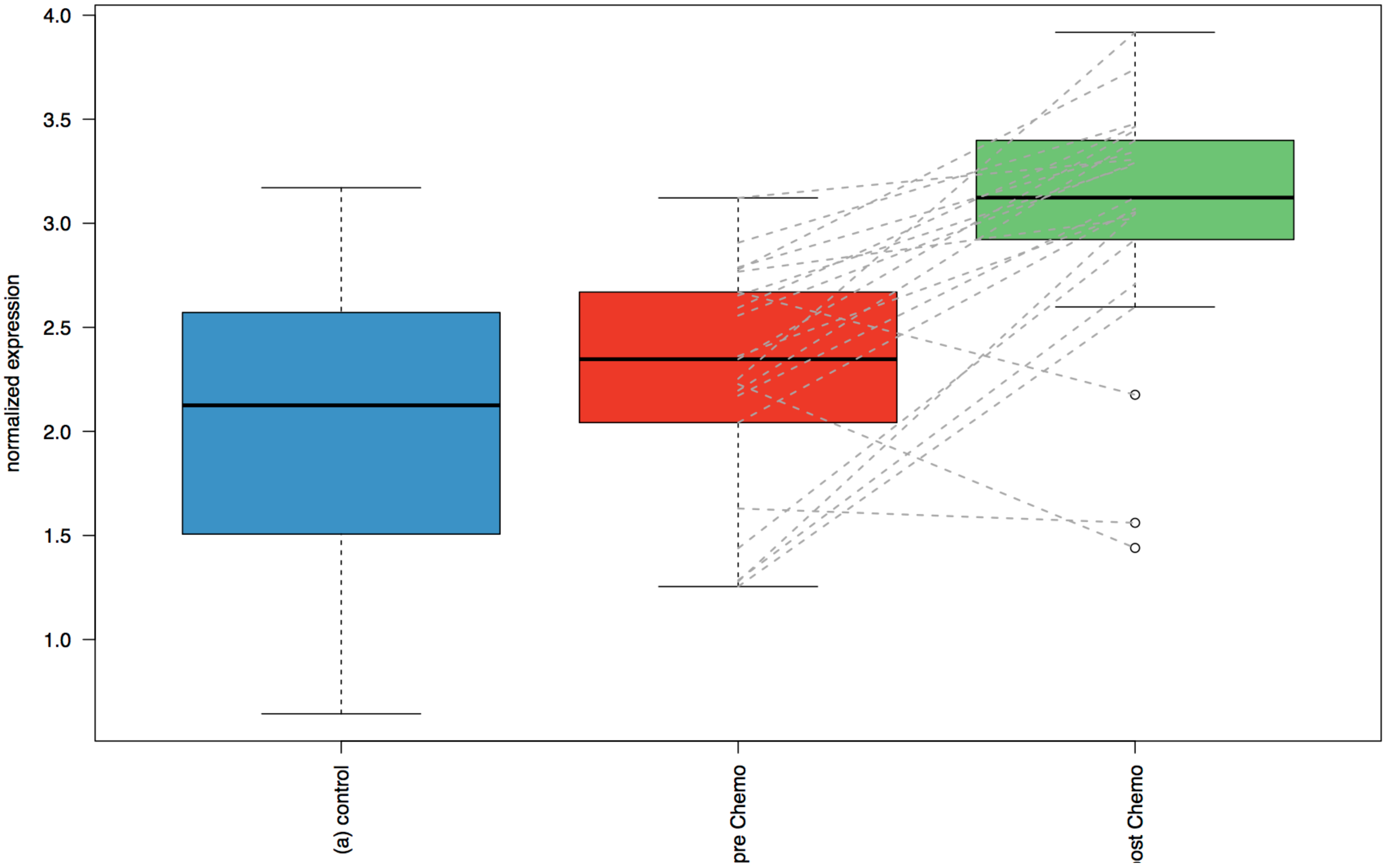
normalized expression
4.0
3.5
3.0
2.5
2.0
1.5
1.0
(a) control
pre Chemo
ost Chemo

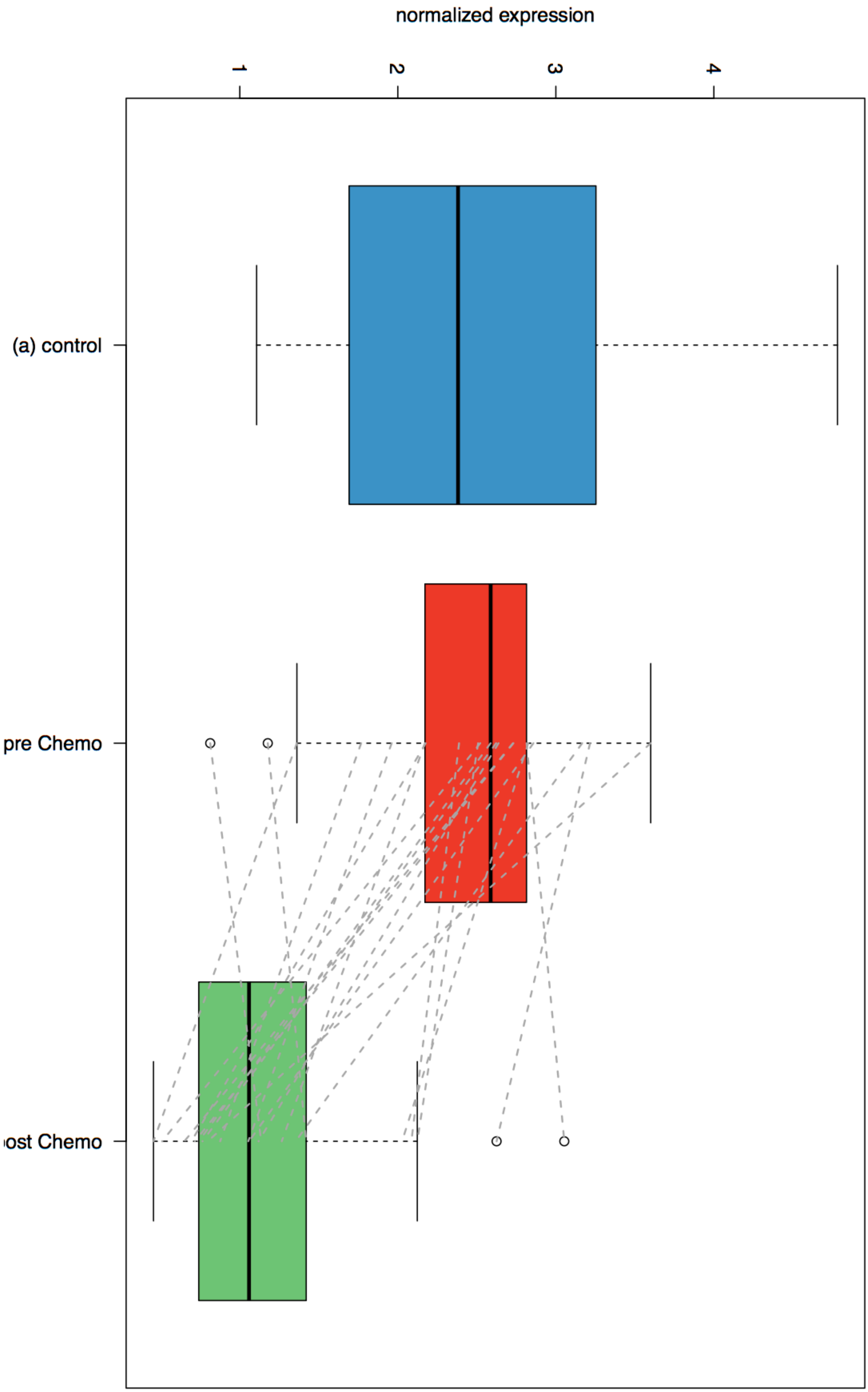
normalized expression
1
2
3
4
(a) control
pre Chemo
ost Chemo

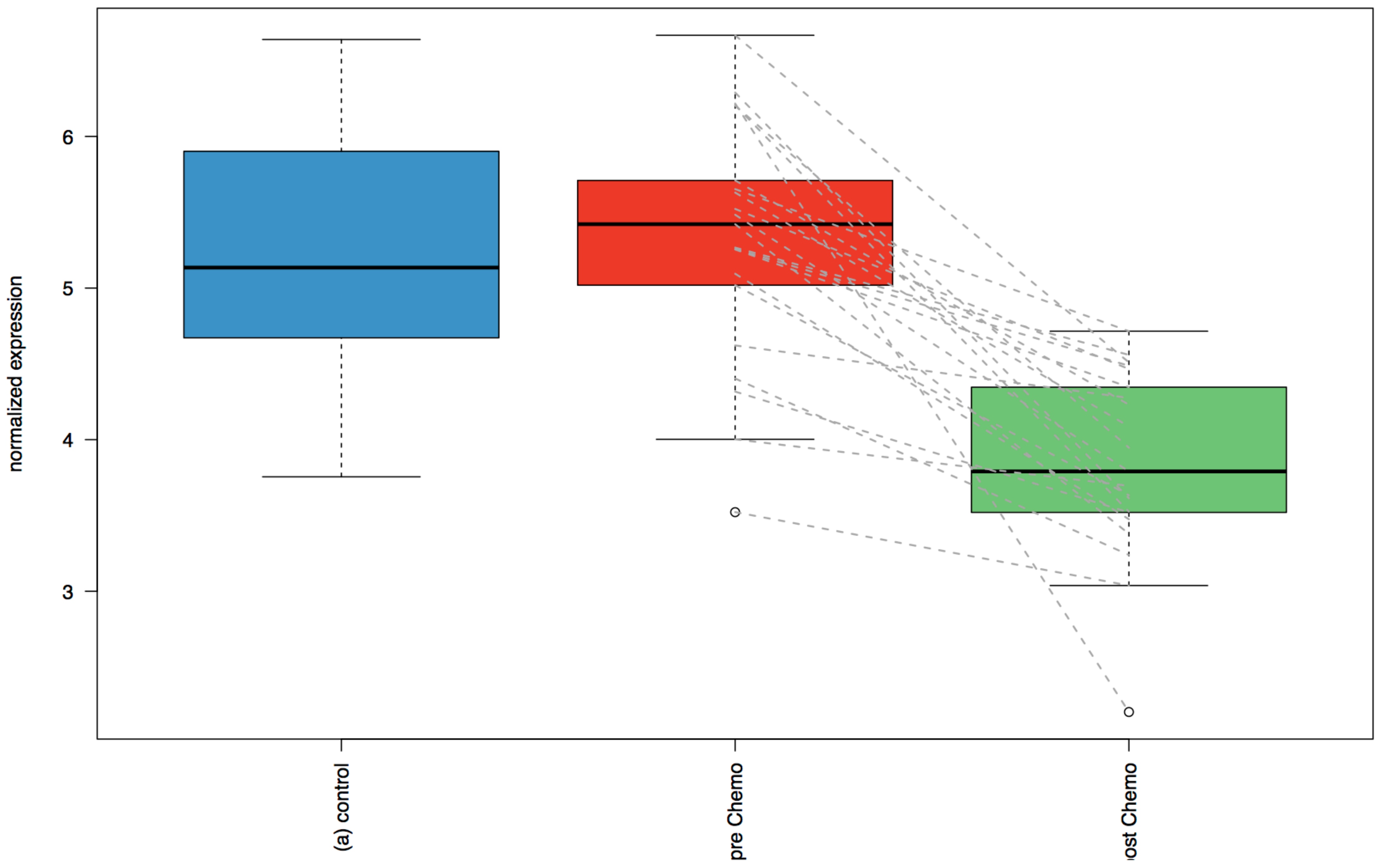
normalized expression
6
5
4
3
(a) control
pre Chemo
ost Chemo

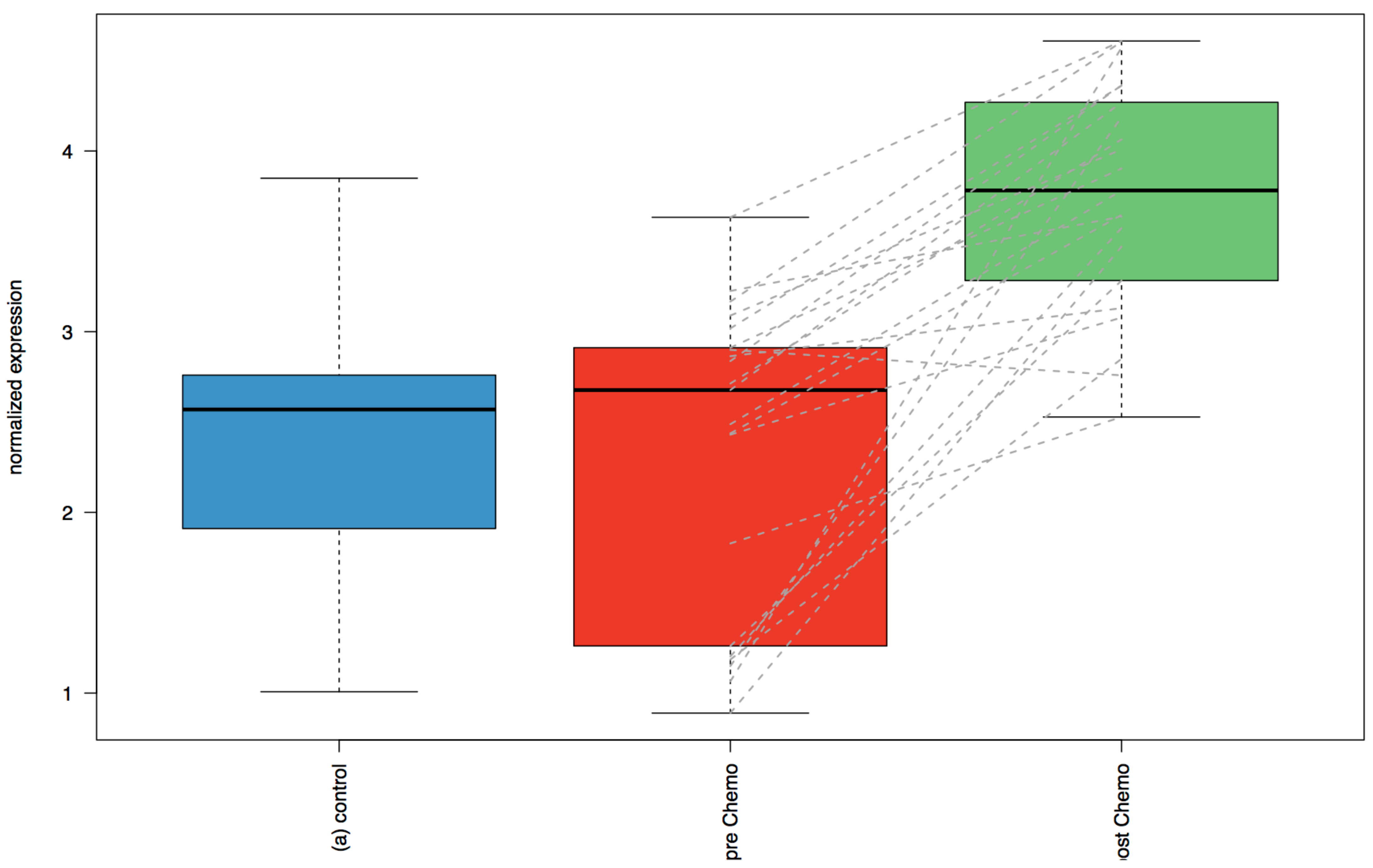
normalized expression
1
2
3
4
(a) control
pre Chemo
ost Chemo

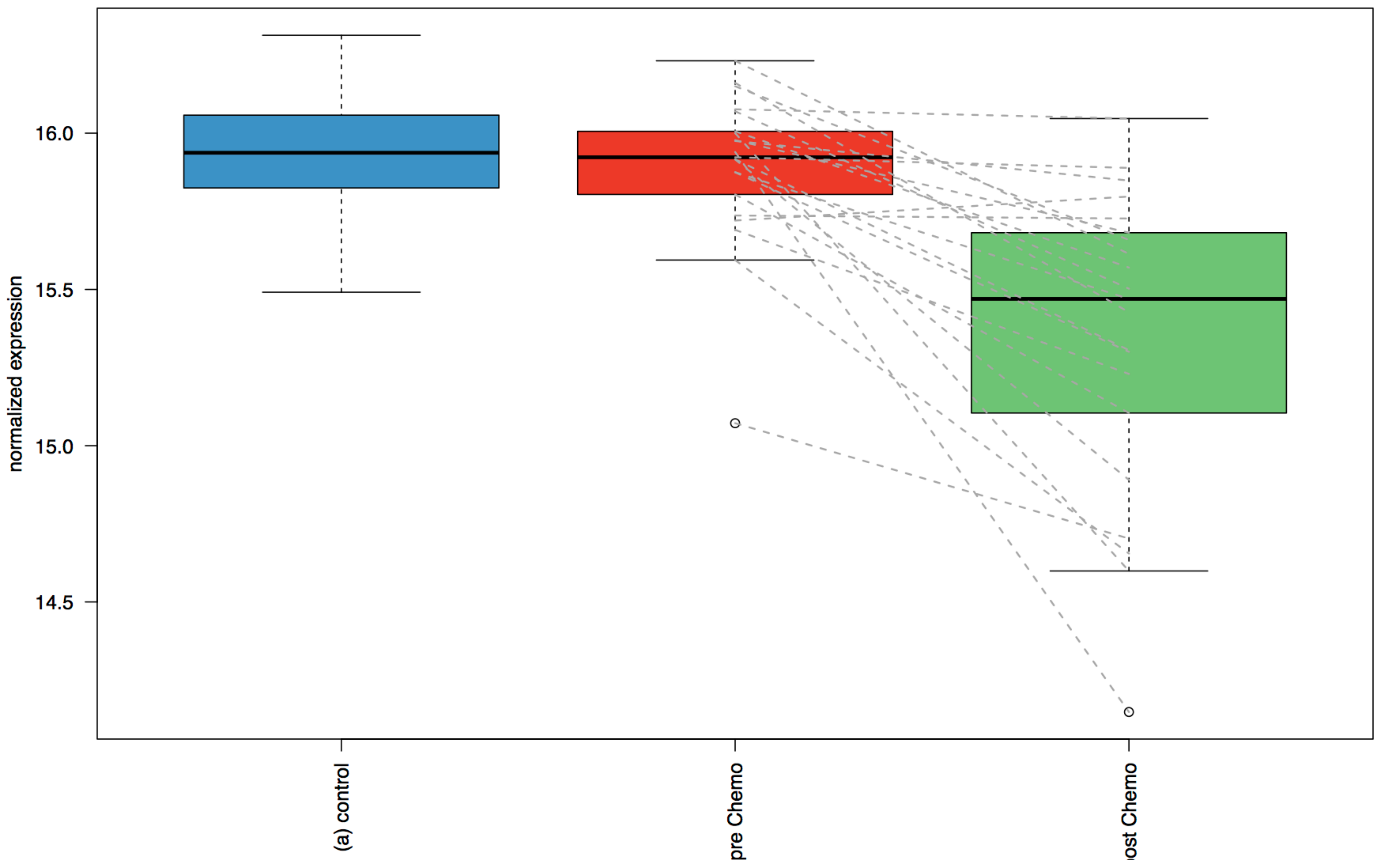
normalized expression
16.0
15.5
15.0
14.5
(a) control
pre Chemo
ost Chemo

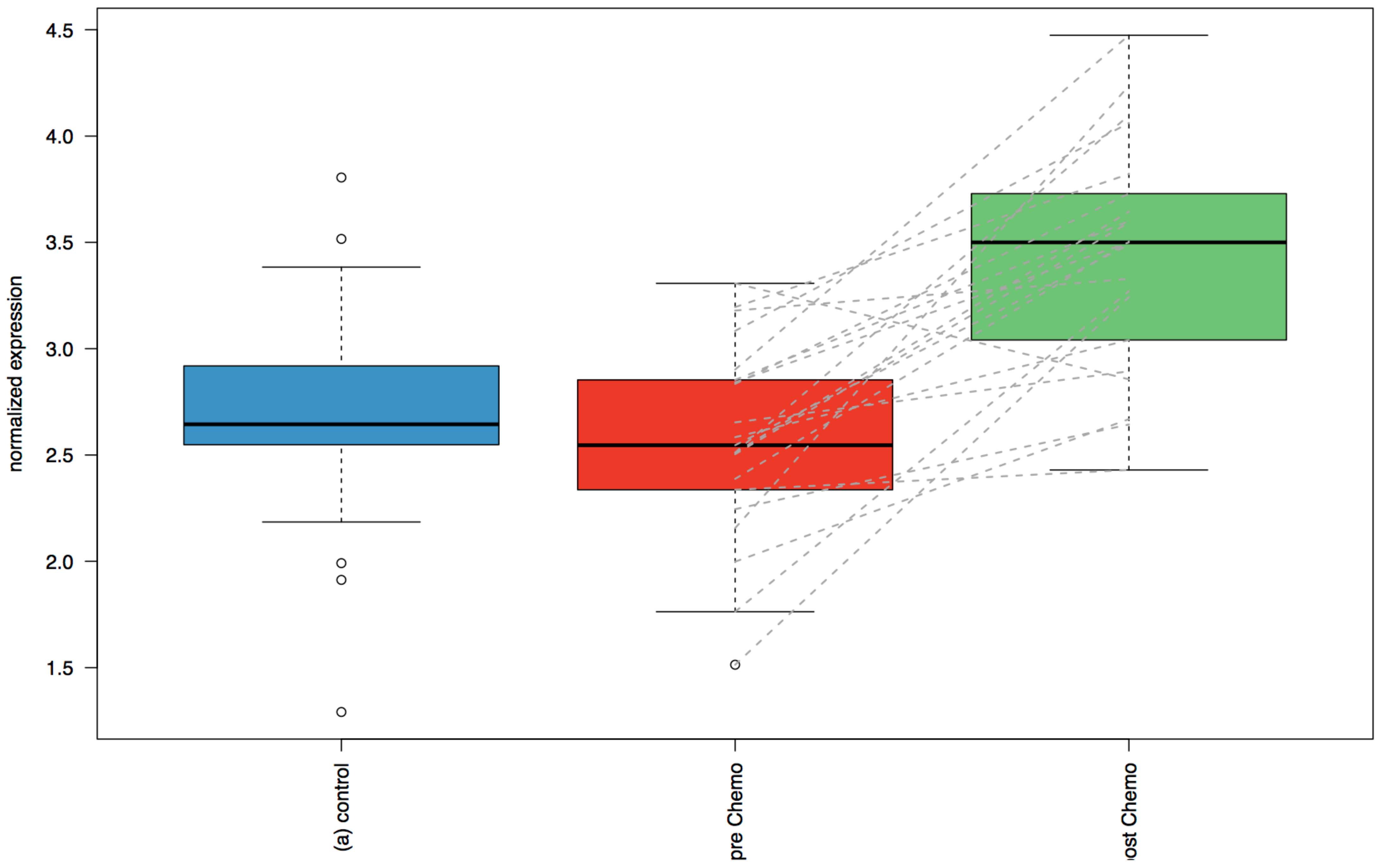

normalized expression
4.5
4.0
3.5
3.0
2.5
2.0
1.5
(a) control
pre Chemo
ost Chemo

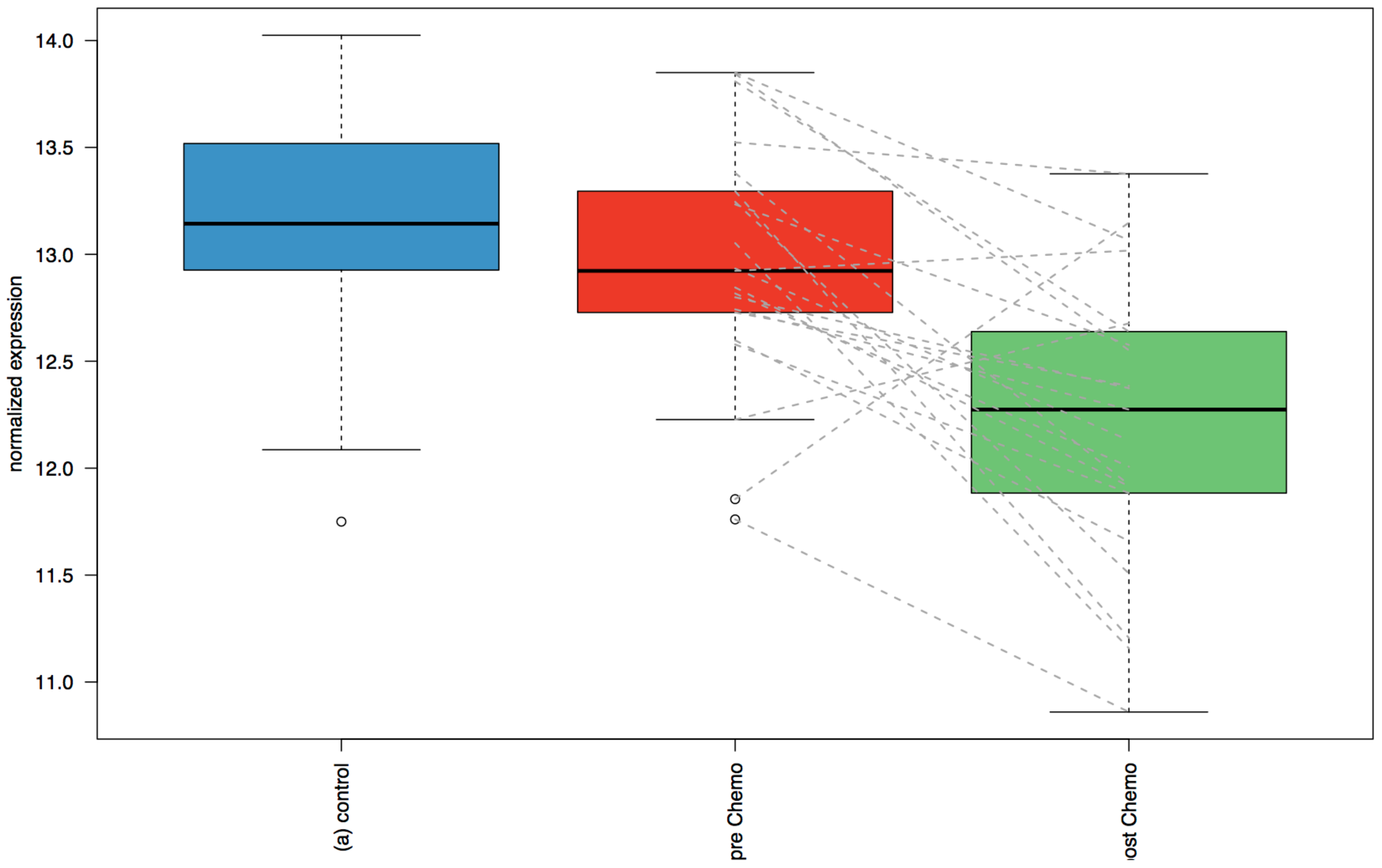
normalized expression
14.0
13.5
13.0
12.5
12.0
11.5
11.0
(a) control
pre Chemo
ost Chemo

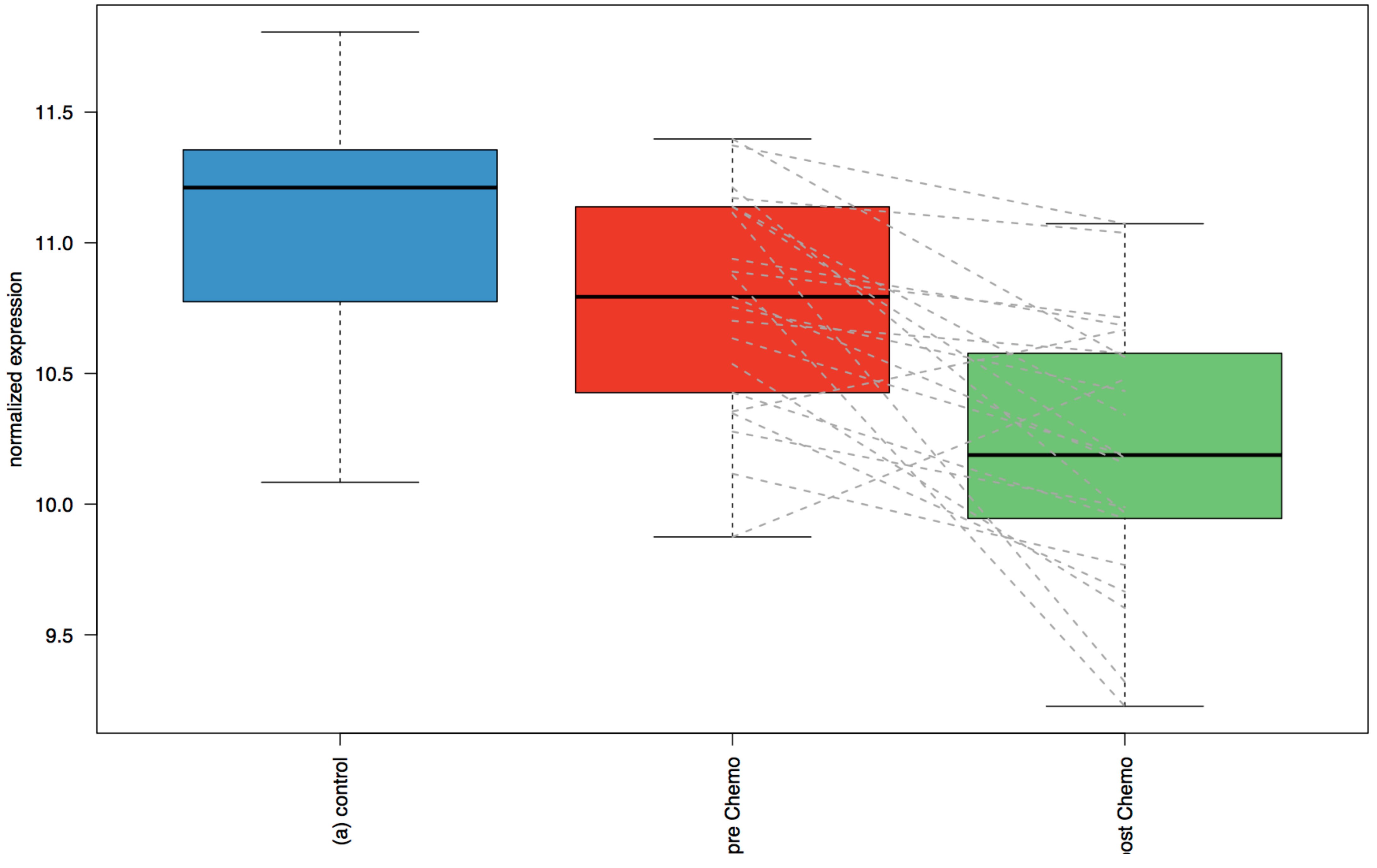
normalized expression
11.5
11.0
10.5
10.0
9.5
(a) control
pre Chemo
ost Chemo

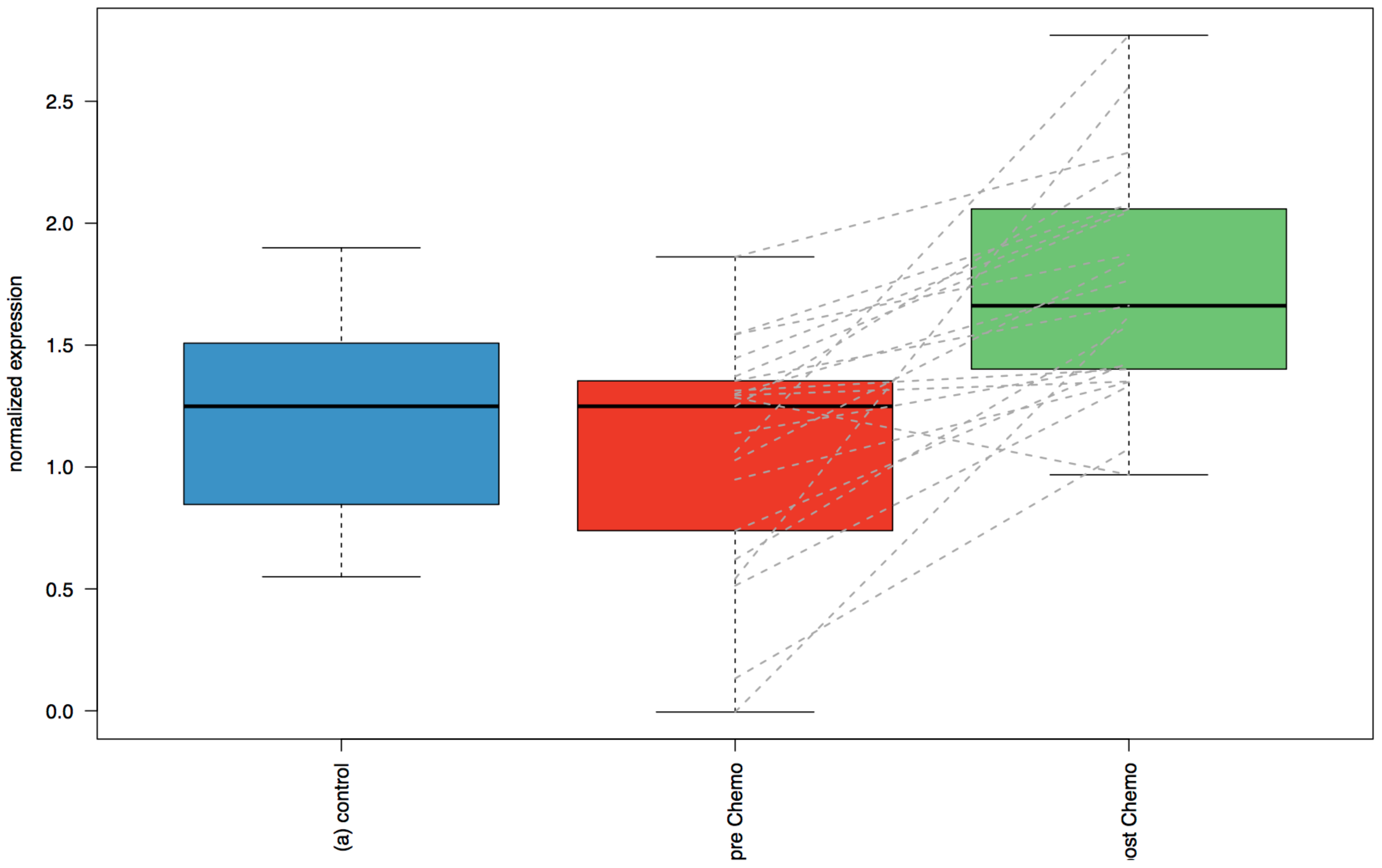
normalized expression
2.5
2.0
1.5
1.0
0.5
0.0
(a) control
pre Chemo
ost Chemo

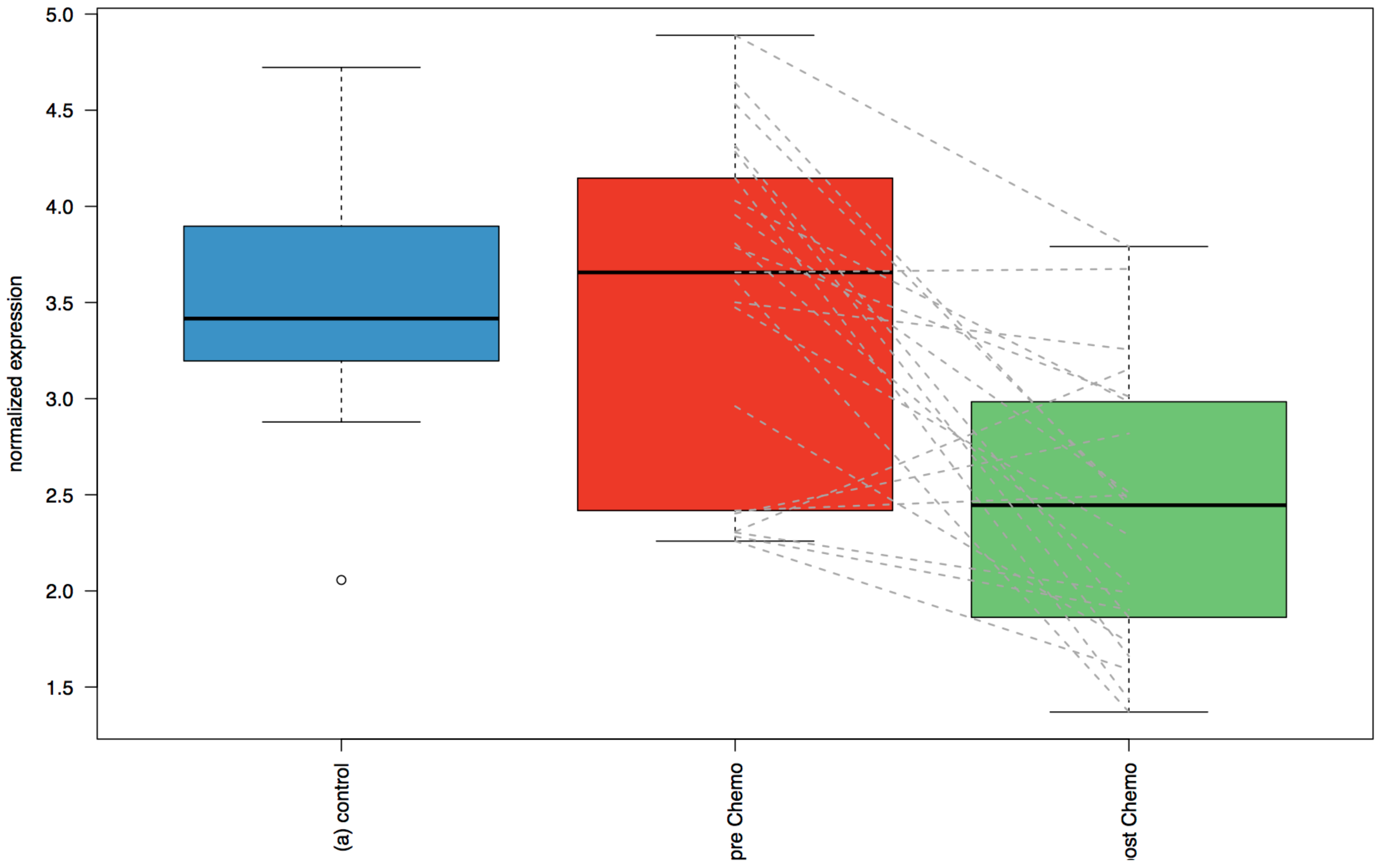

normalized expression
5.0
4.5
4.0
3.5
3.0
2.5
2.0
1.5
(a) control
pre Chemo
ost Chemo

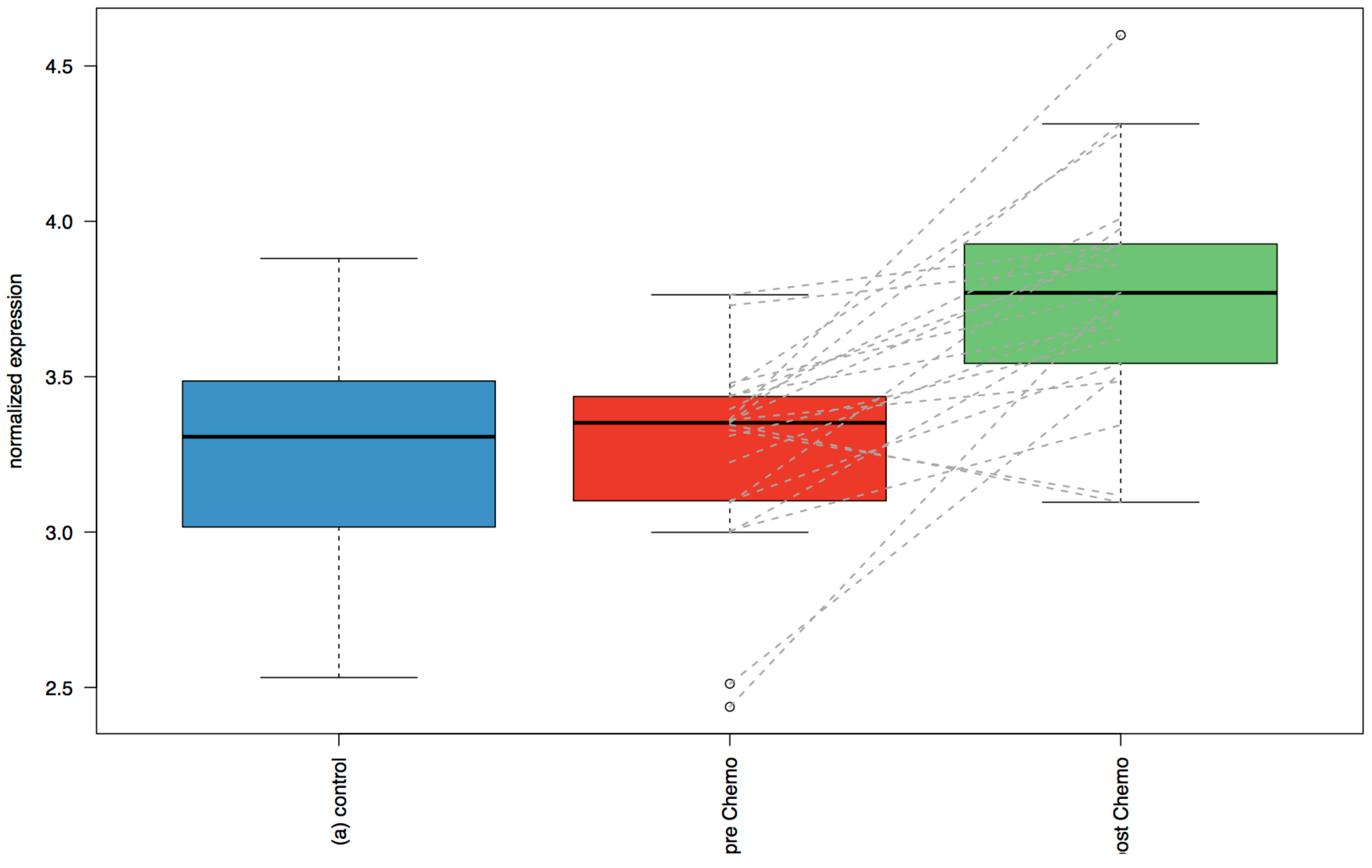
normalized expression
4.5
4.0
3.5
3.0
2.5
(a) control
pre Chemo
ost Chemo

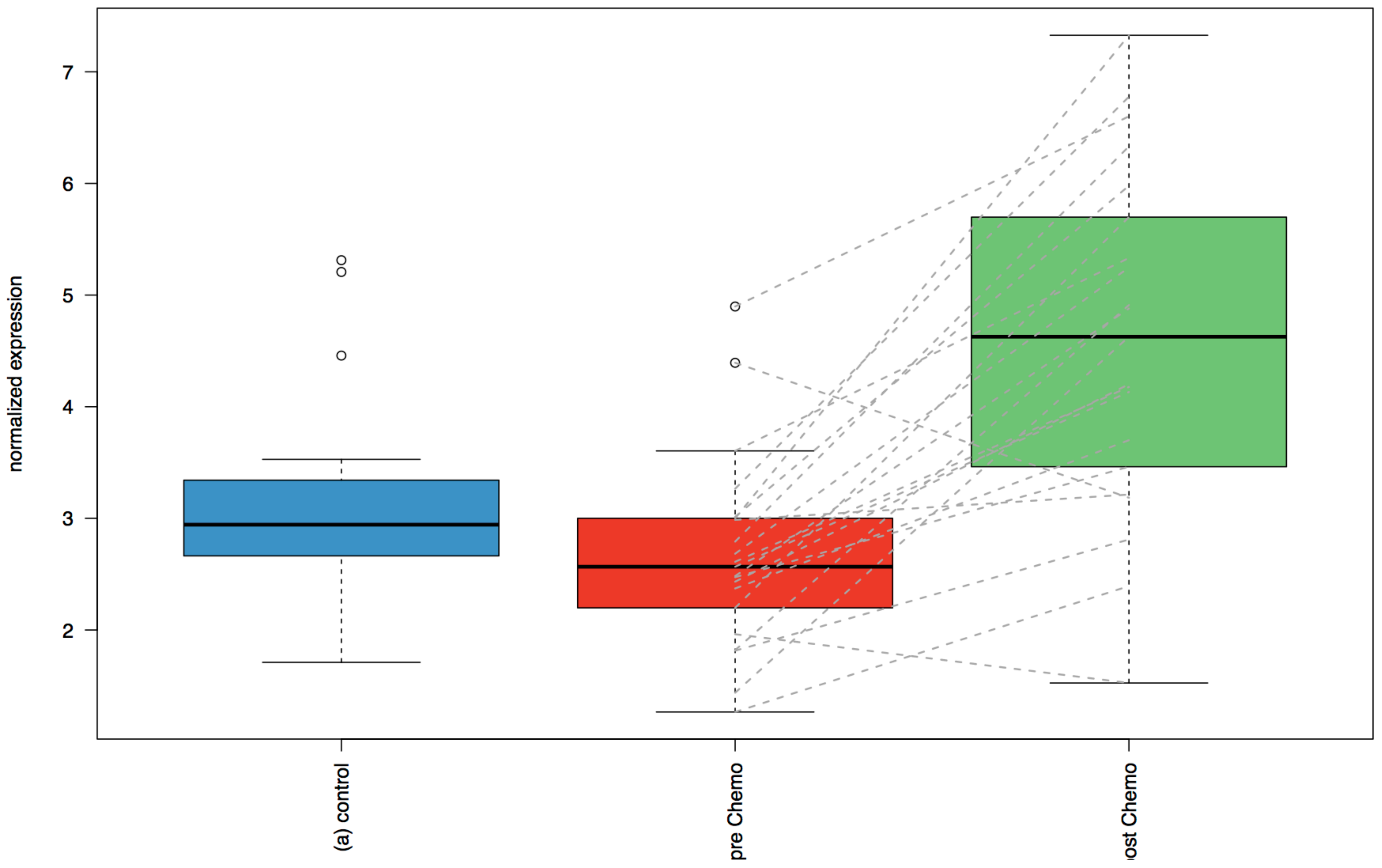
normalized expression
7
6
5
4
3
2
(a) control
pre Chemo
ost Chemo

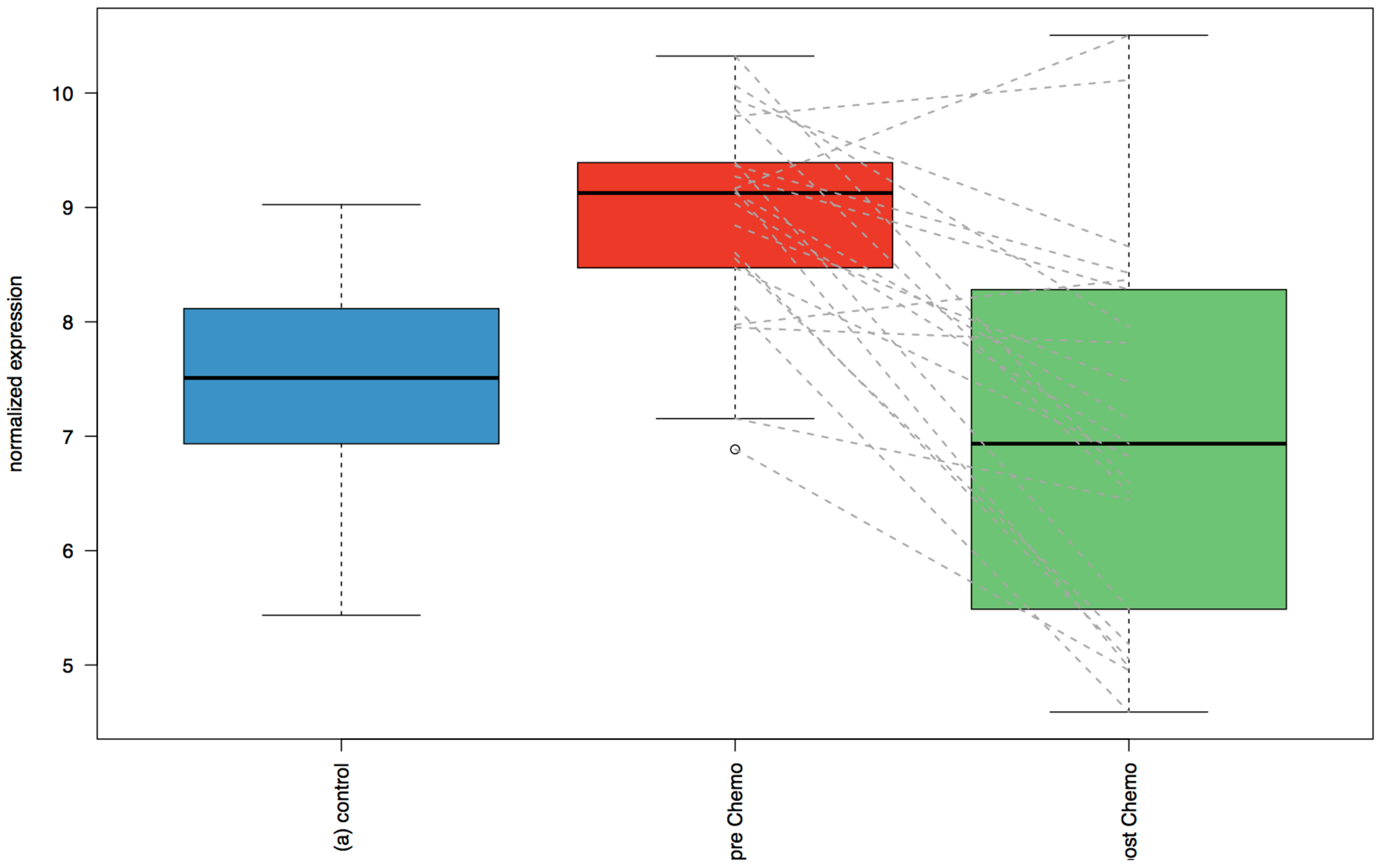
normalized expression
10
9
8
7
6
5
(a) control
pre Chemo
ost Chemo

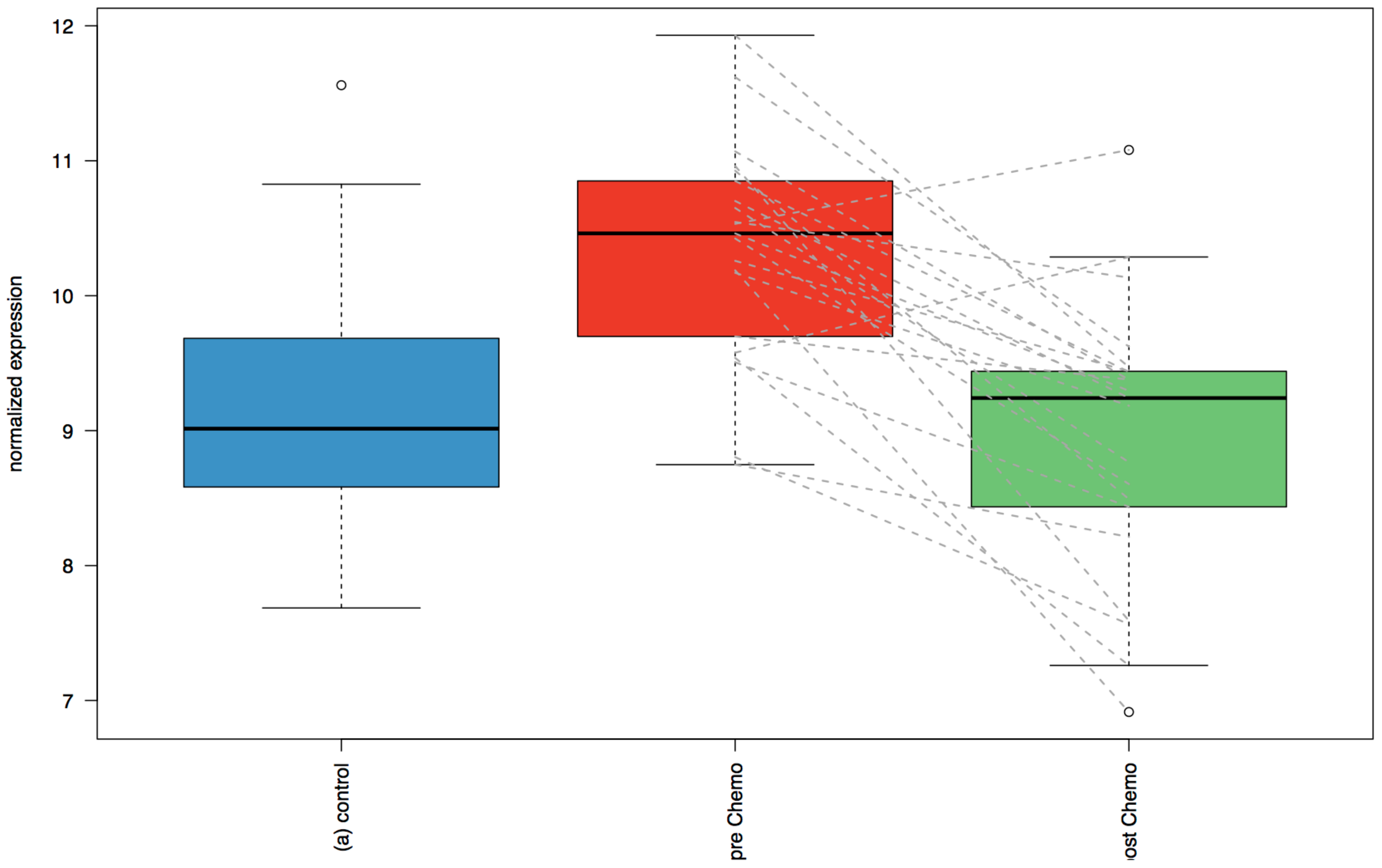
normalized expression
12
11
10
9
8
7
(a) control
pre Chemo
ost Chemo

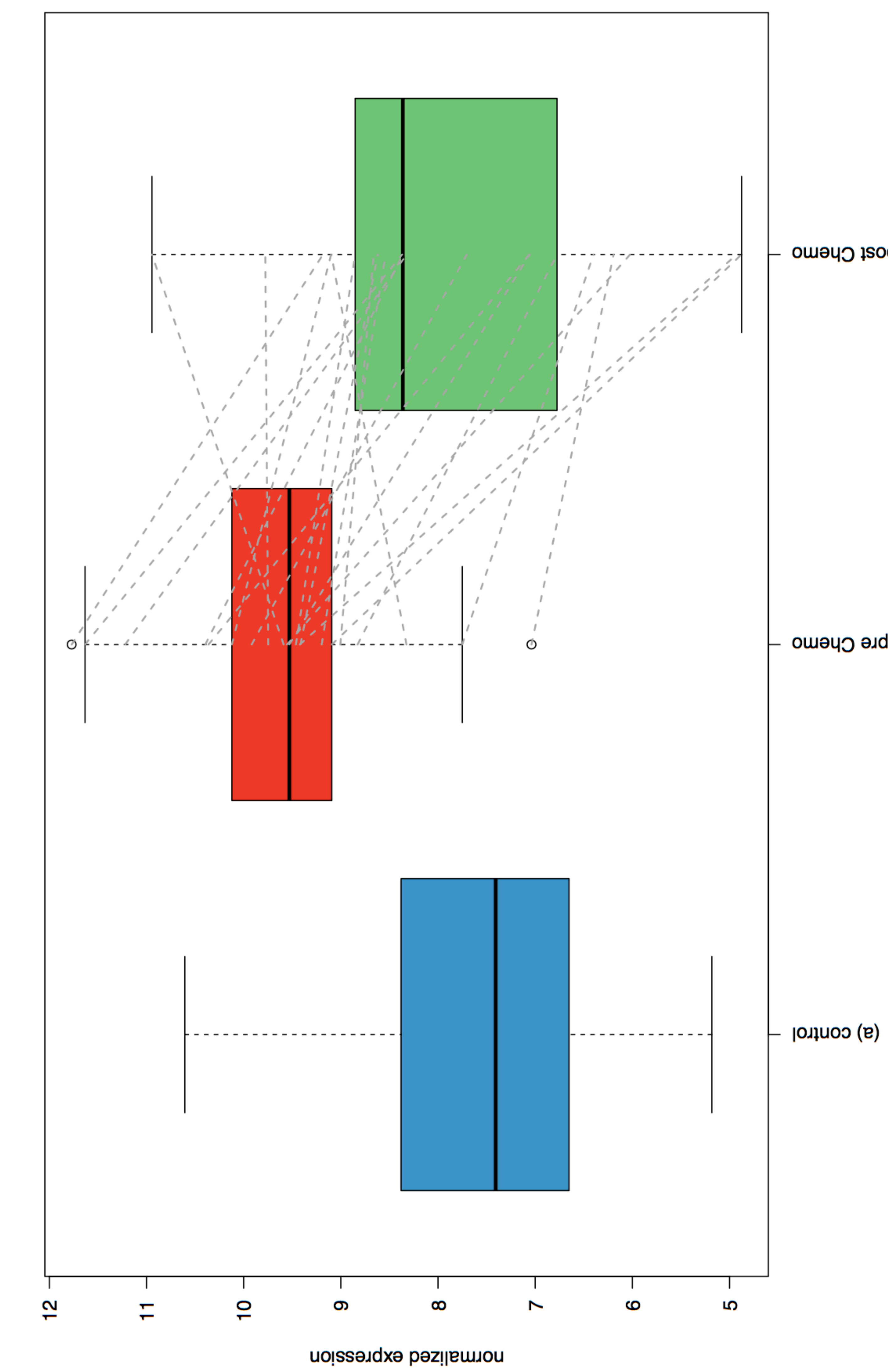
normalized expression
12
11
10
9
8
7
6
5
(a) control
pre Chemo
ost Chemo

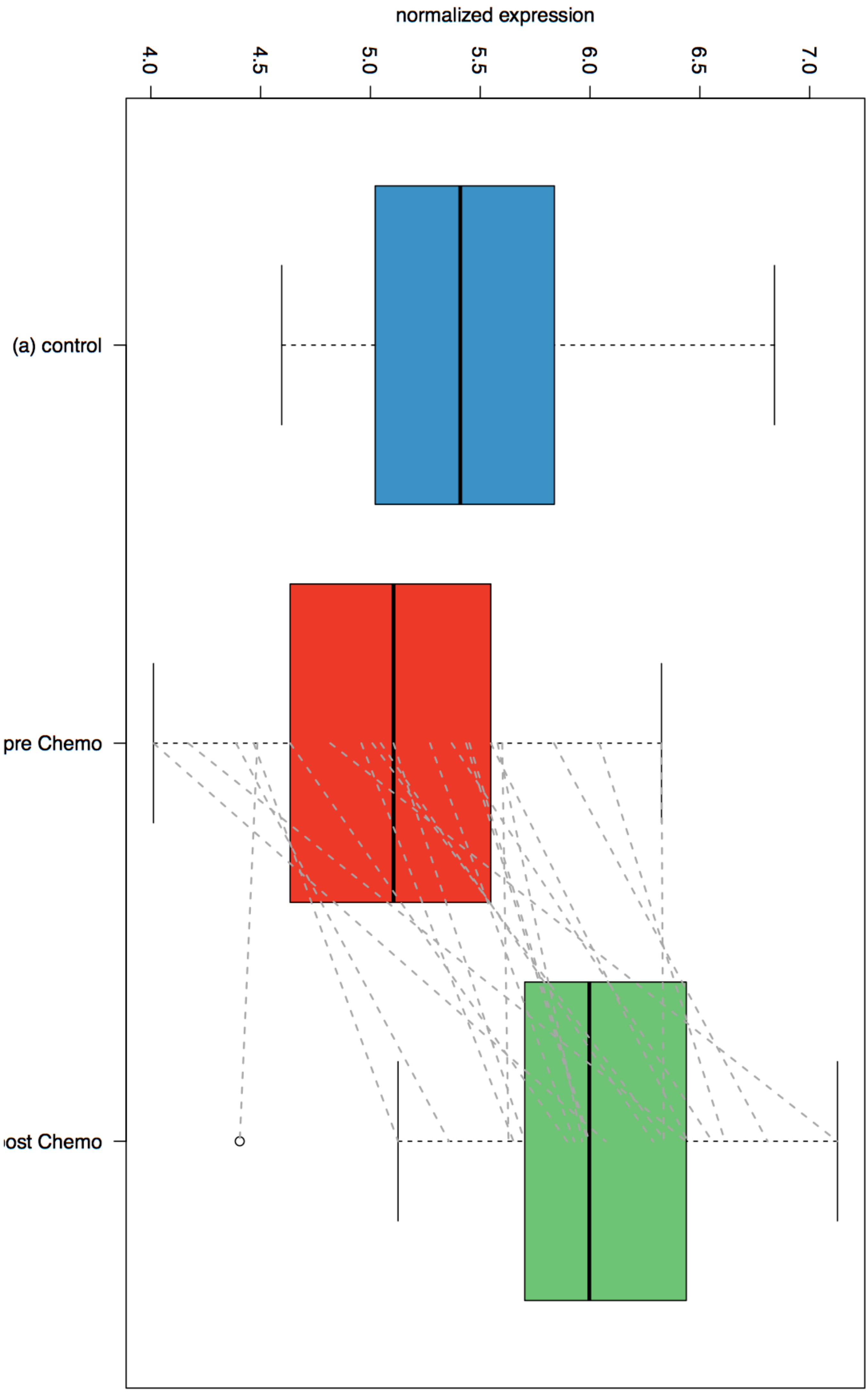
normalized expression
4.0
4.5
5.0
5.5
6.0
6.5
7.0
(a) control
pre Chemo
ost Chemo

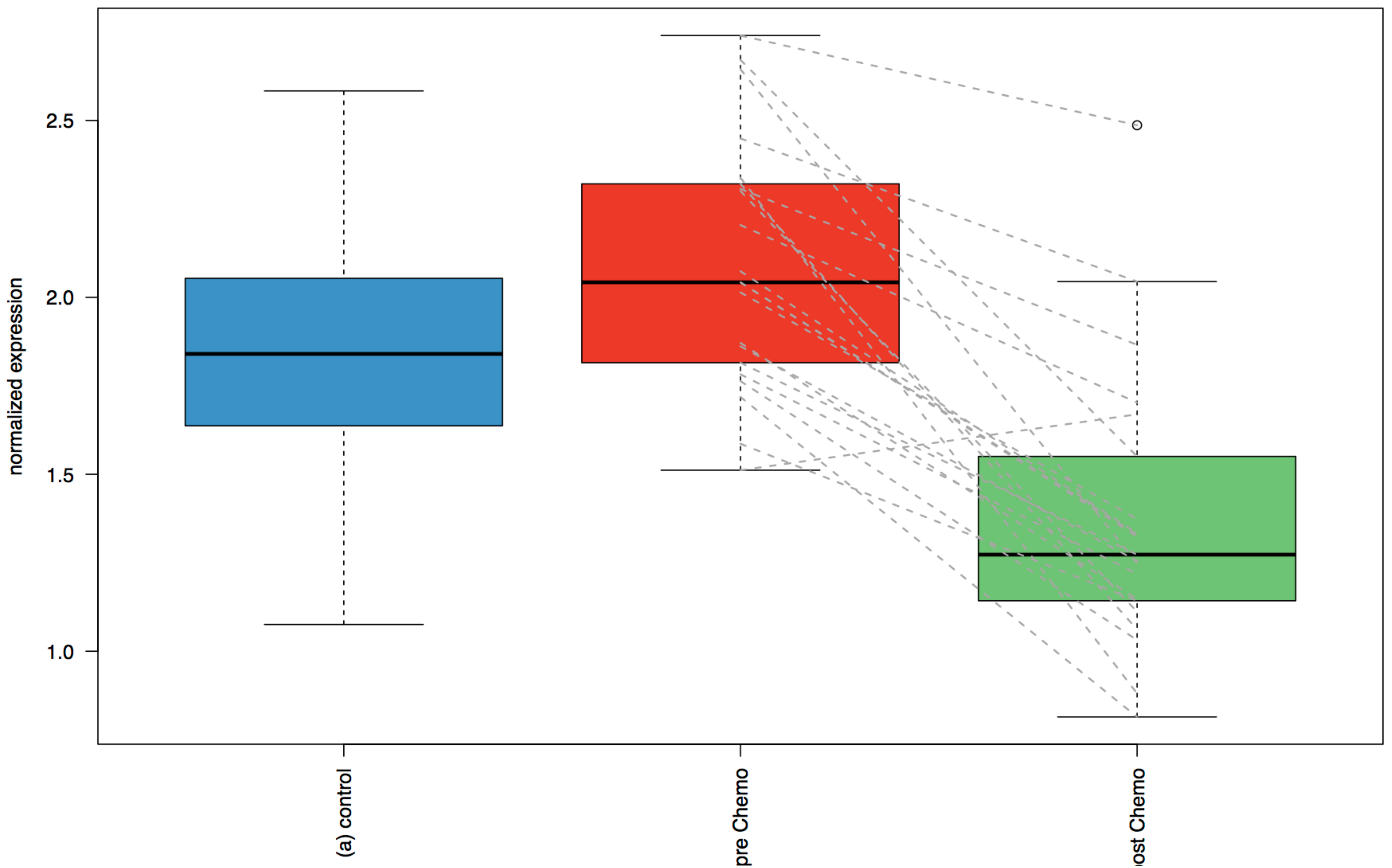
normalized expression
2.5
2.0
1.5
1.0
(a) control
pre Chemo
ost Chemo

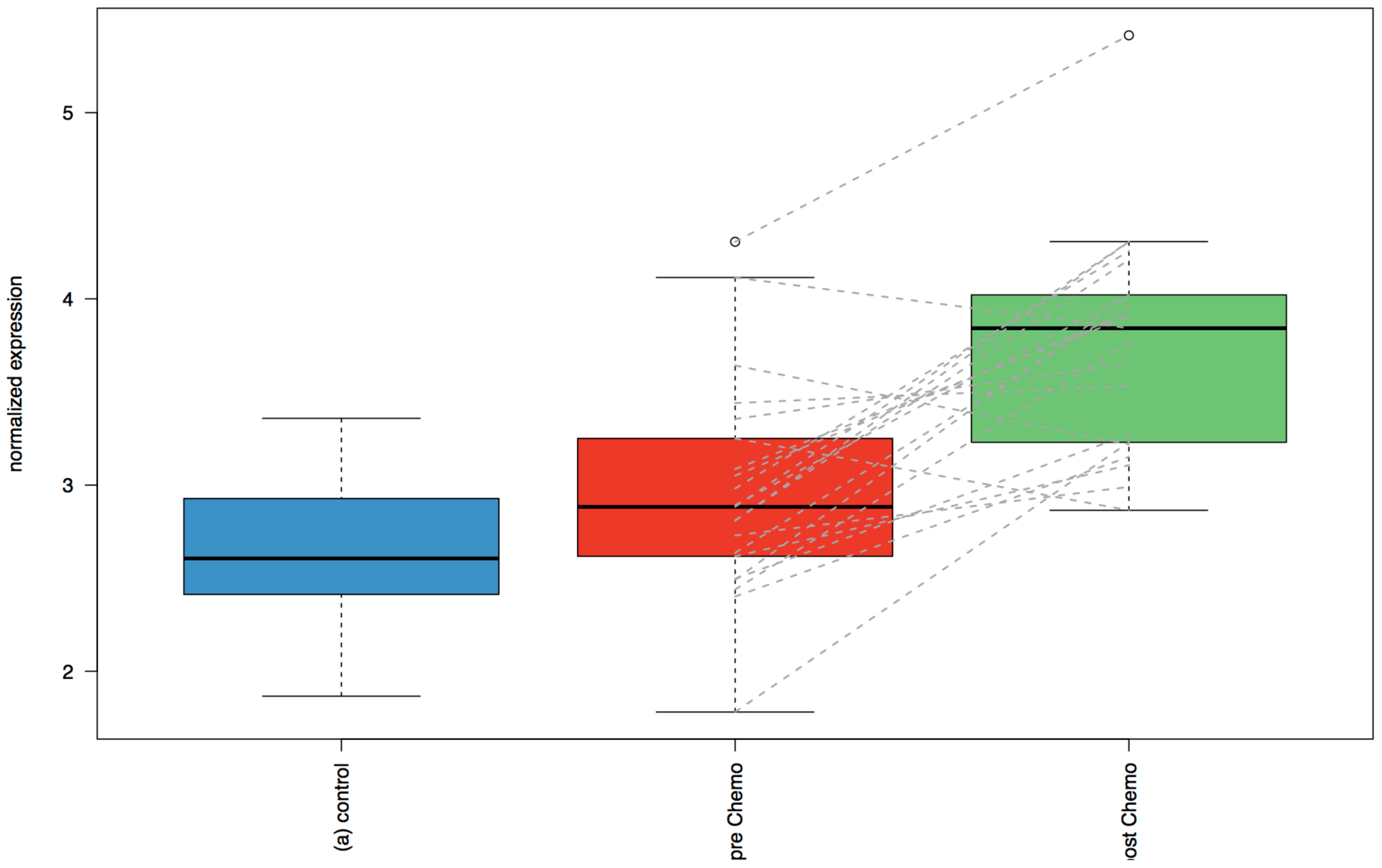
normalized expression
5
4
3
2
(a) control
pre Chemo
ost Chemo

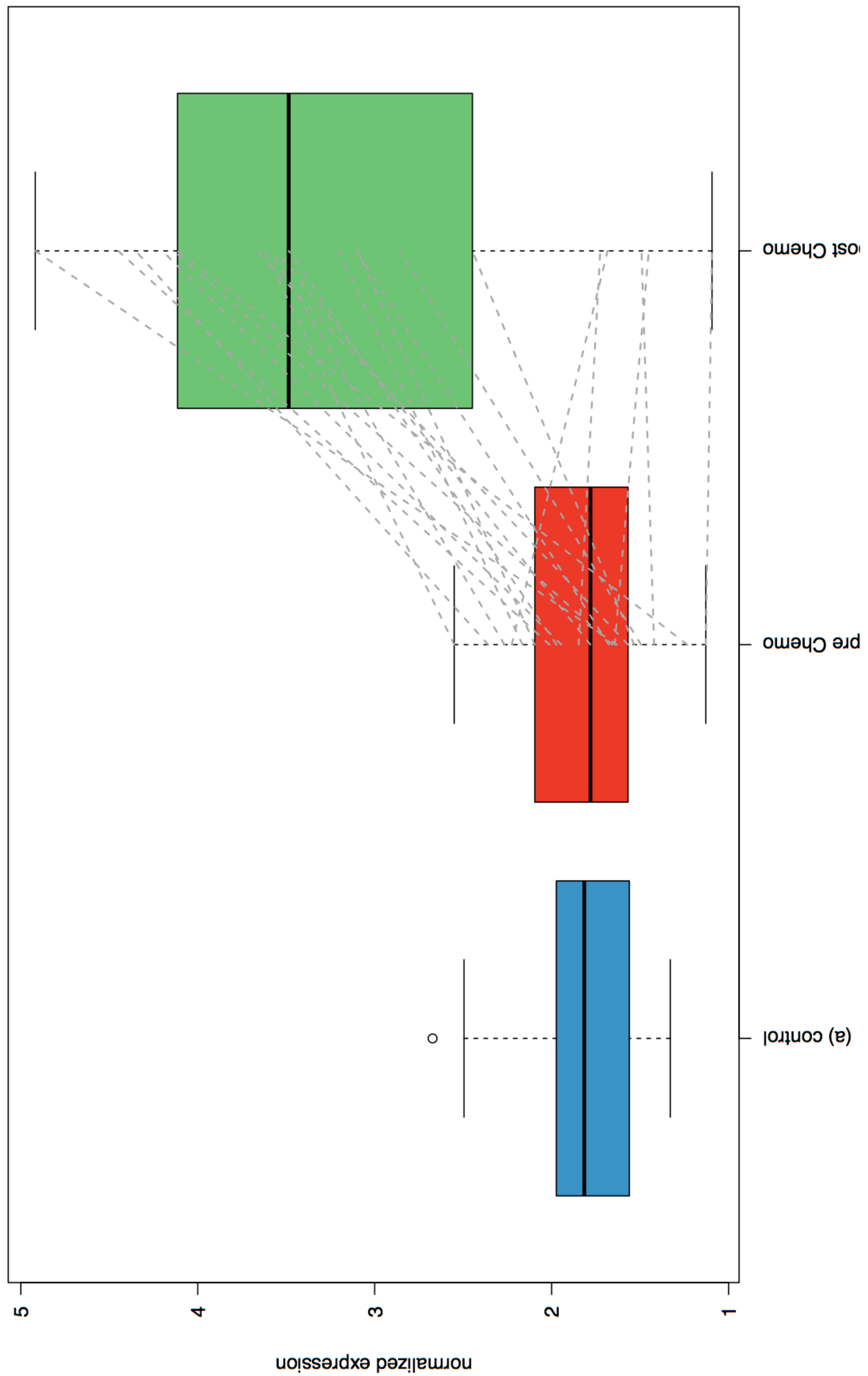

normalized expression
1
2
3
4
5
(a) control
pre Chemo
ost Chemo

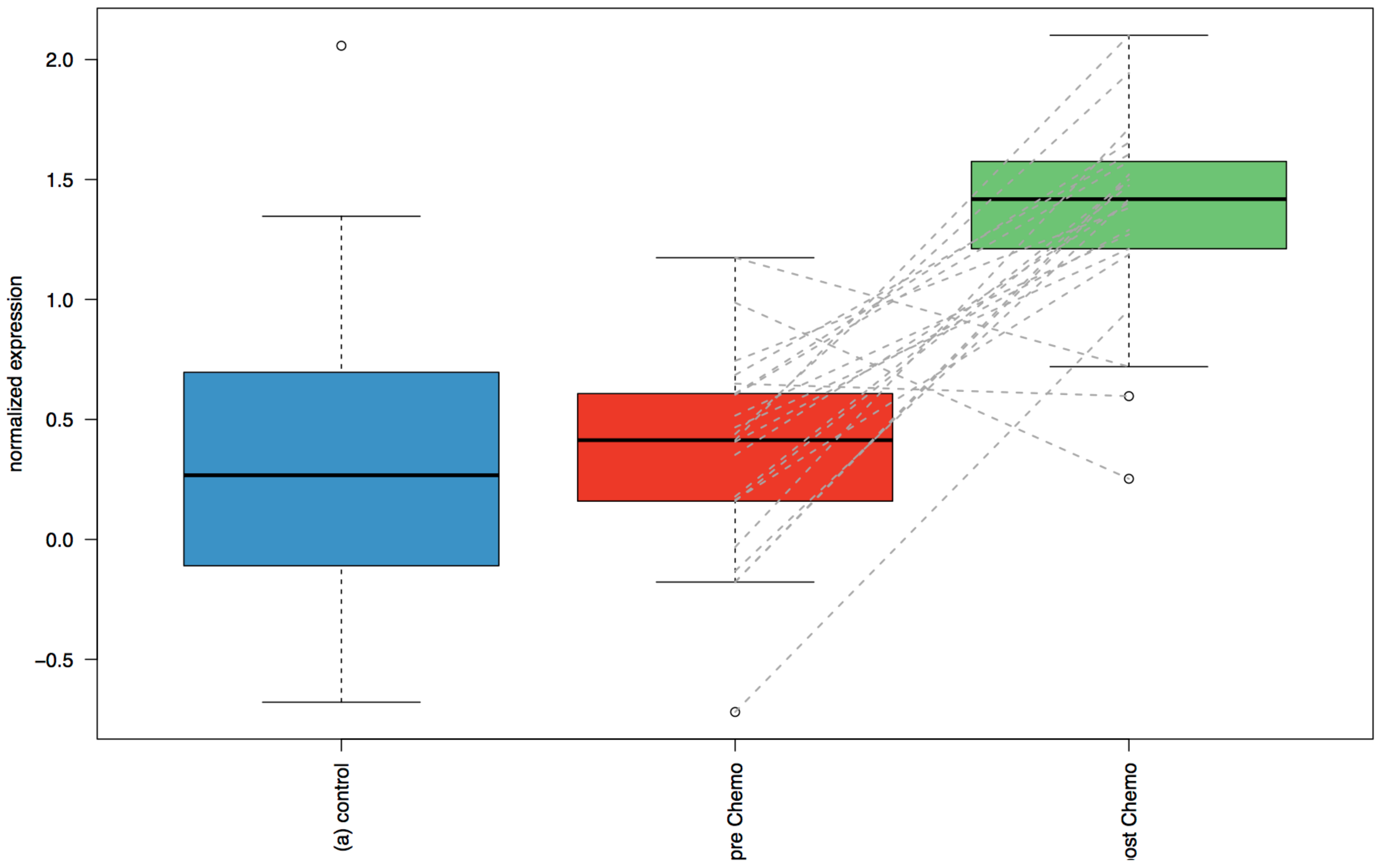
normalized expression
2.0
1.5
1.0
0.5
0.0
−0.5
(a) control
pre Chemo
ost Chemo

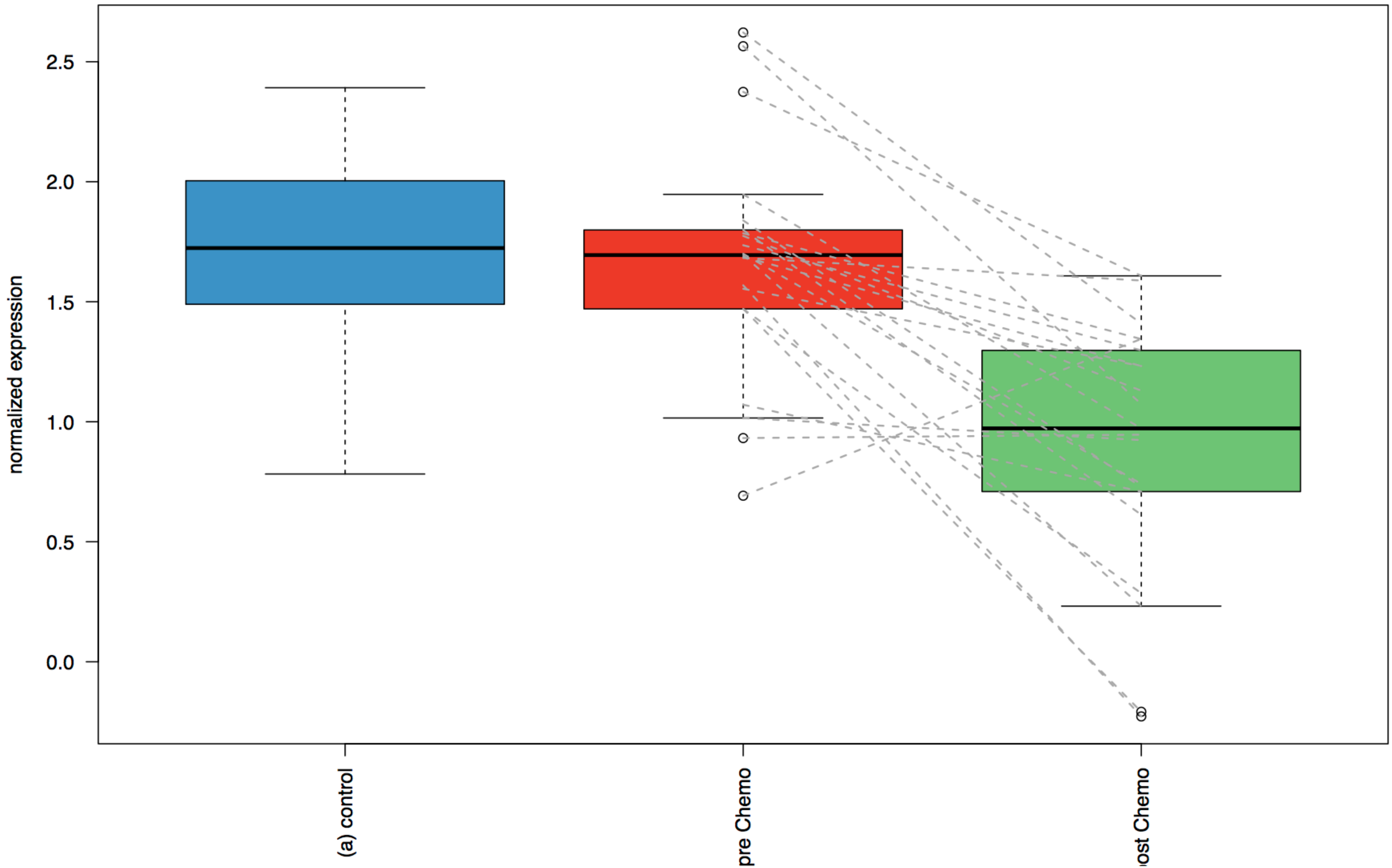
normalized expression
2.5
2.0
1.5
1.0
0.5
0.0
(a) control
pre Chemo
ost Chemo

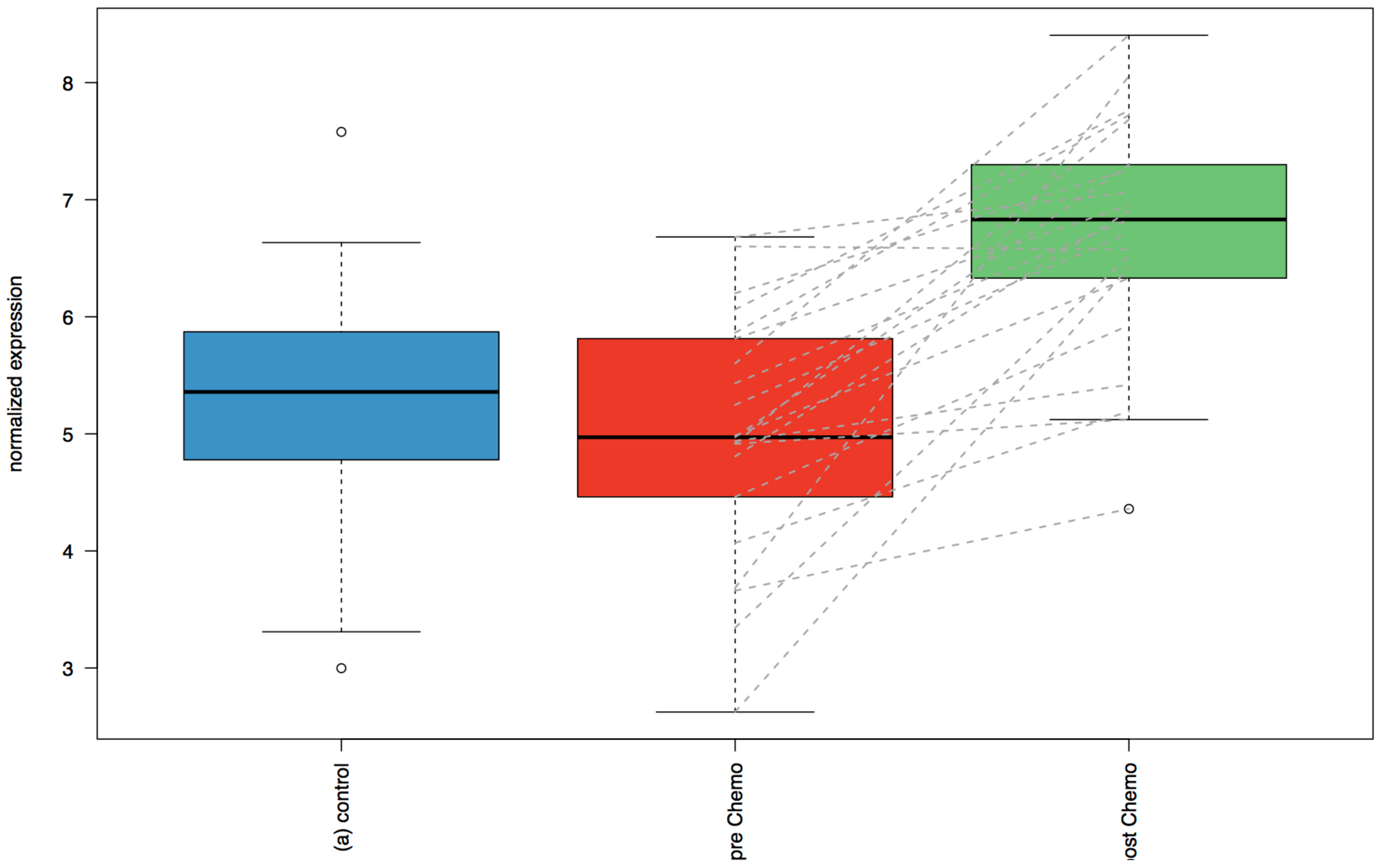
normalized expression
8
7
6
5
4
3
(a) control
pre Chemo
ost Chemo

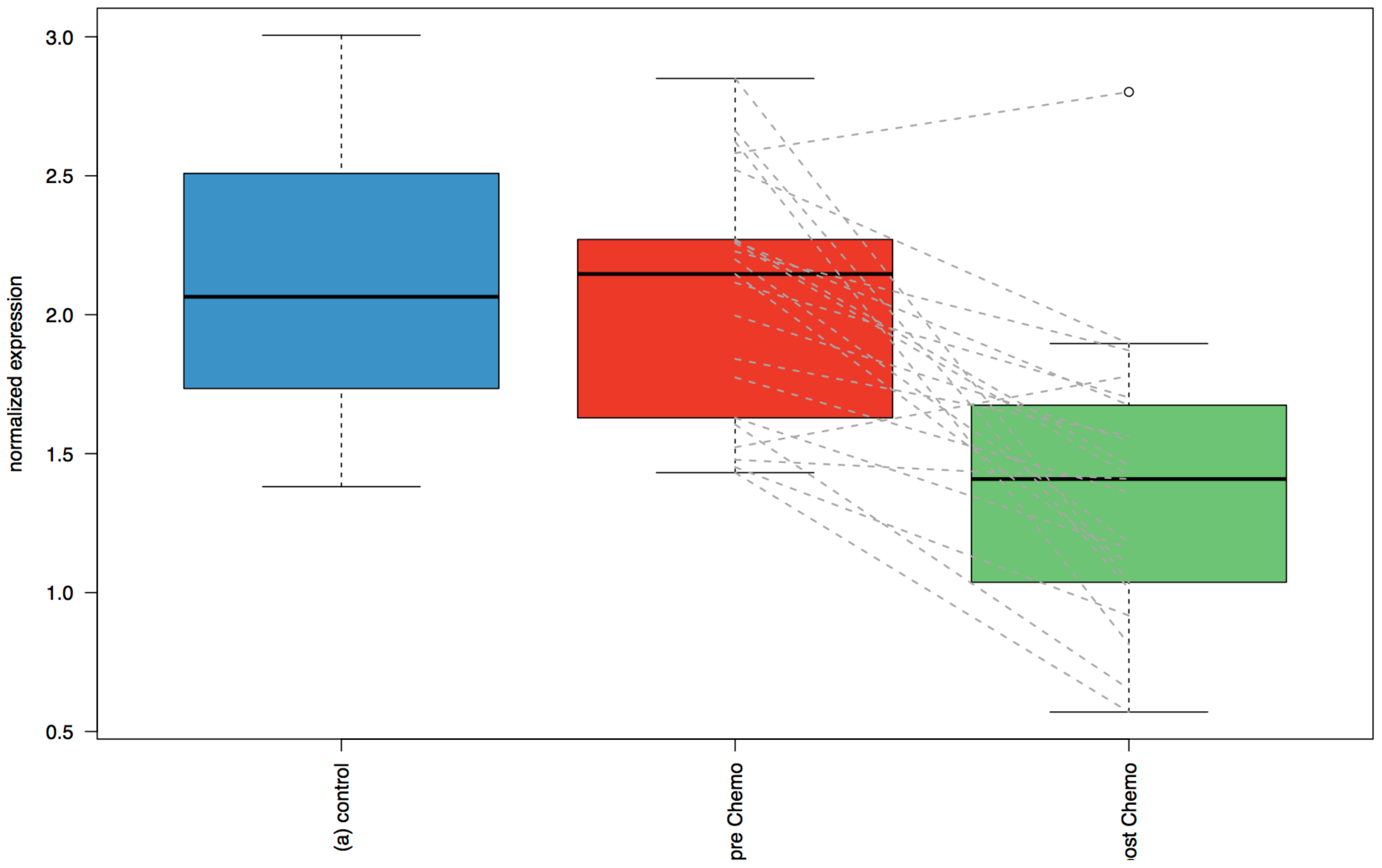

normalized expression
3.0
2.5
2.0
1.5
1.0
0.5
(a) control
pre Chemo
ost Chemo

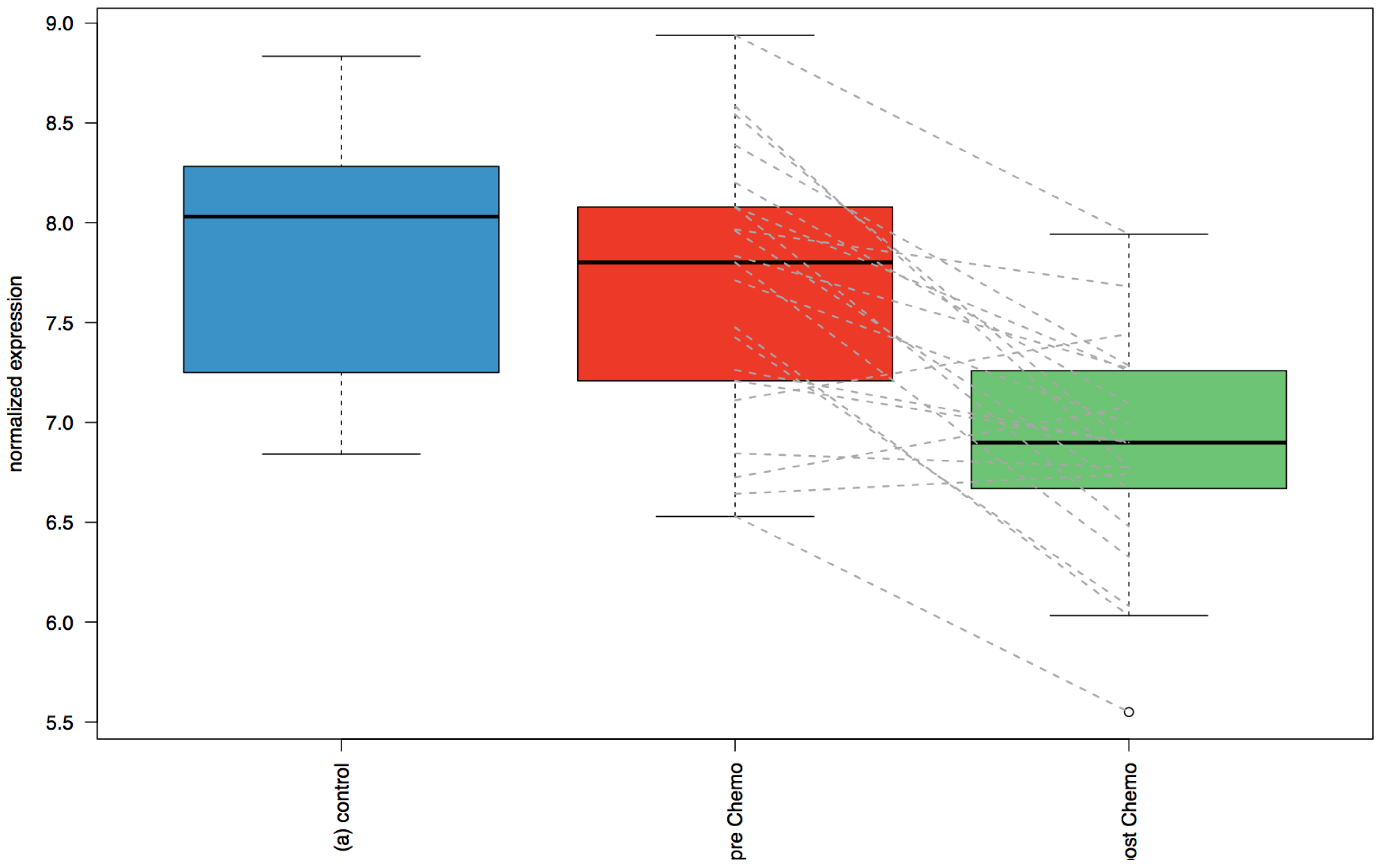
normalized expression
9.0
8.5
8.0
7.5
7.0
6.5
6.0
5.5
(a) control
pre Chemo
ost Chemo

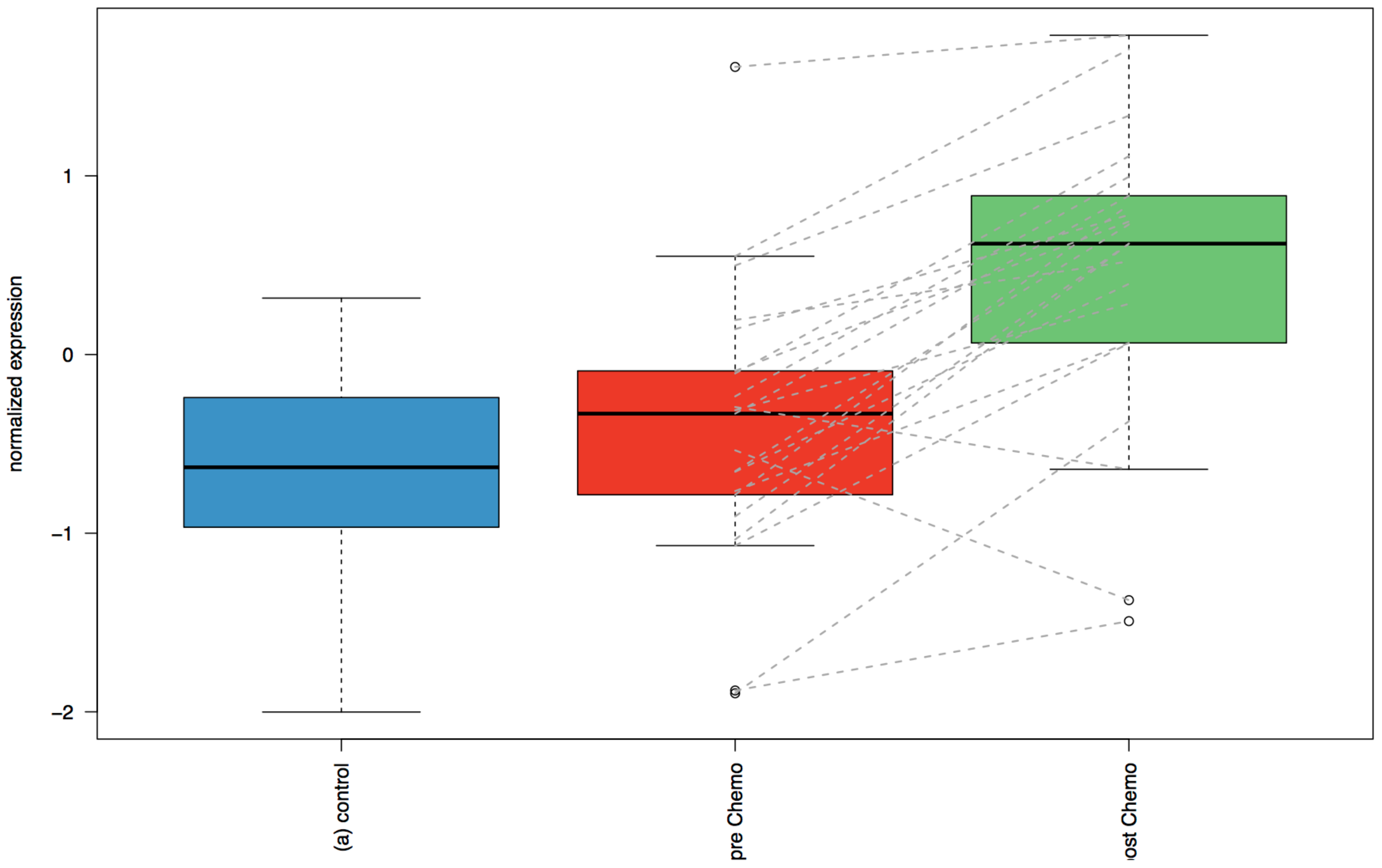
normalized expression
1
0
−1
−2
(a) control
pre Chemo
ost Chemo

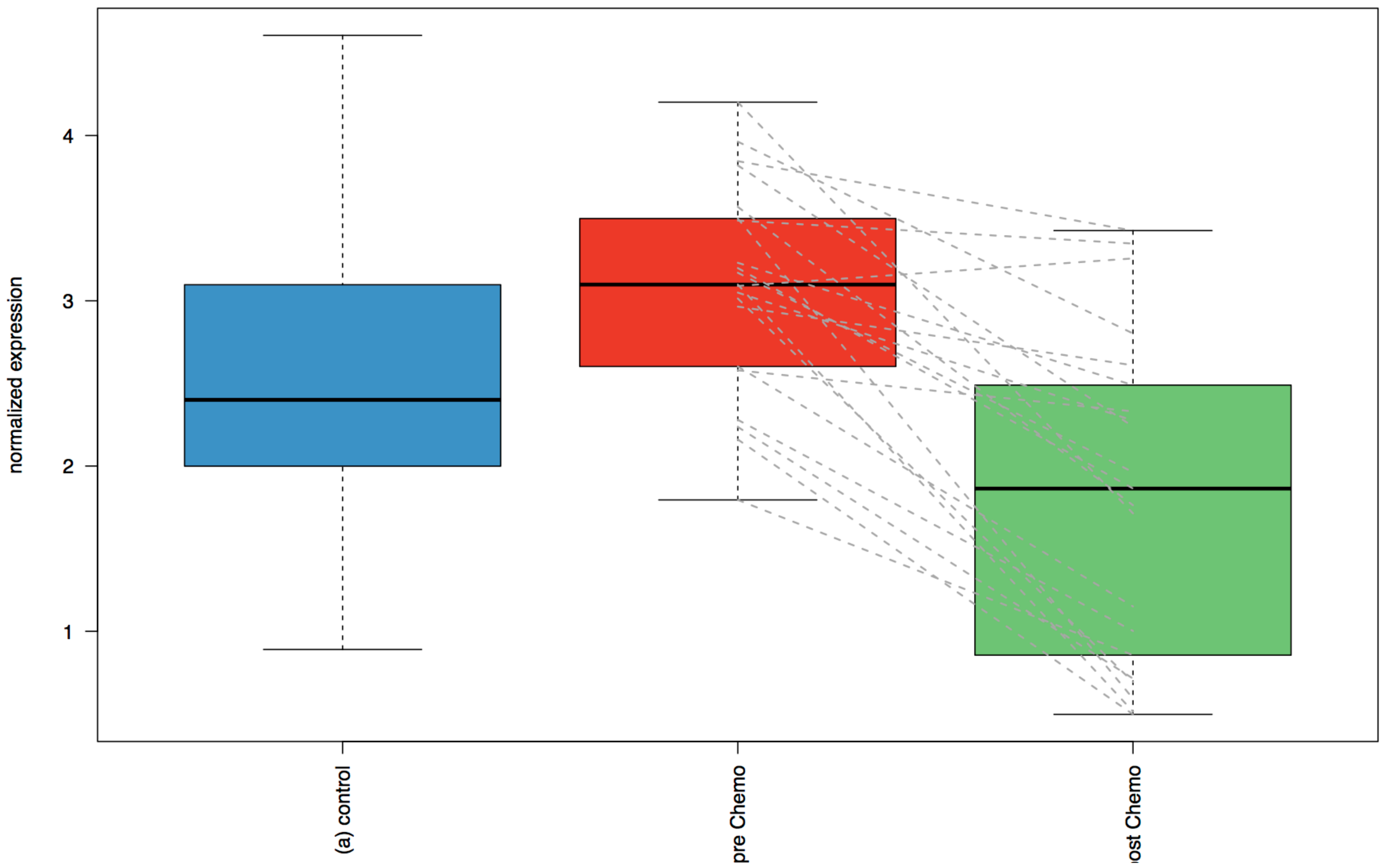
normalized expression
4
3
2
1
(a) control
pre Chemo
ost Chemo

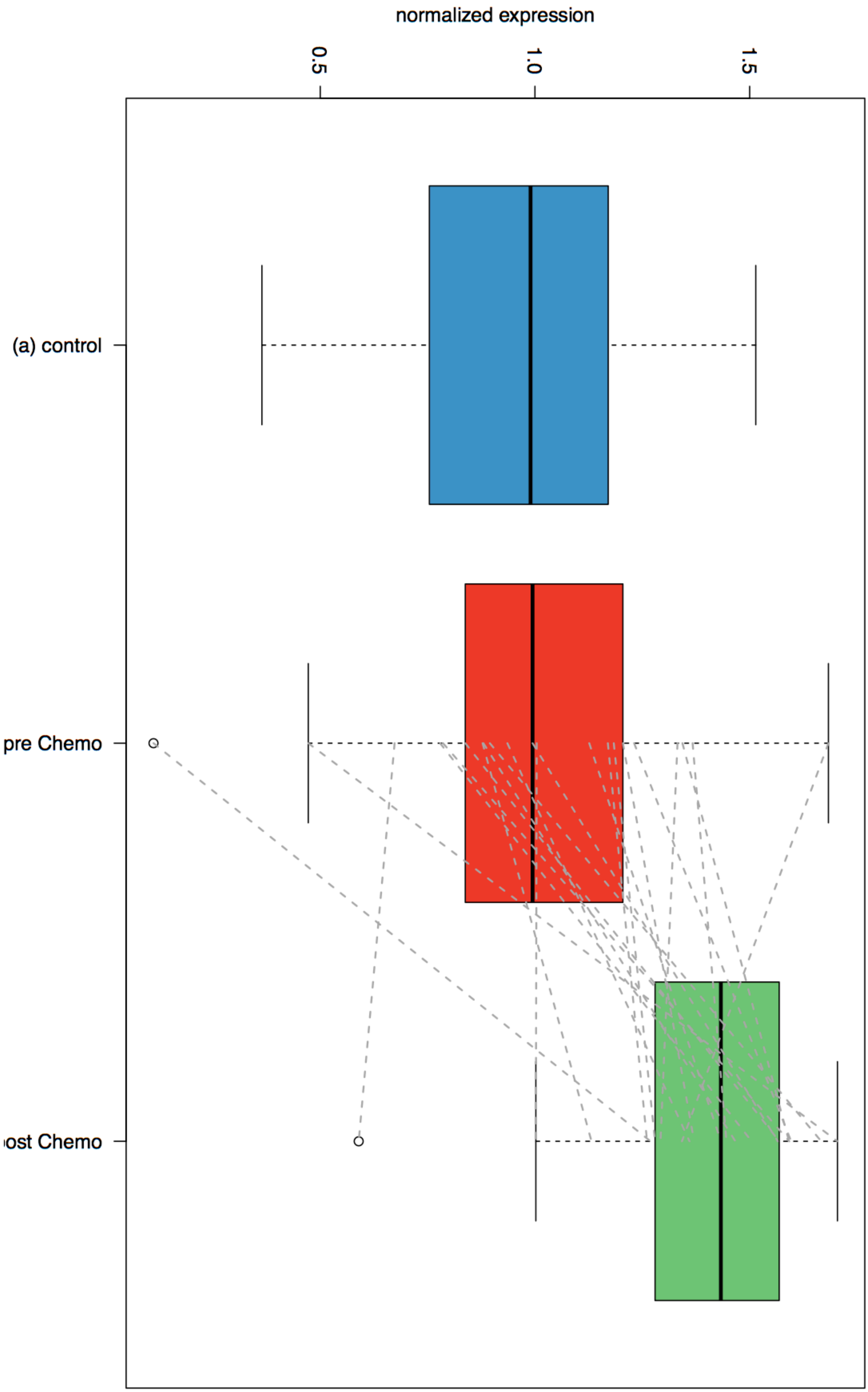
normalized expression
0.5
1.0
1.5
(a) control
pre Chemo
ost Chemo

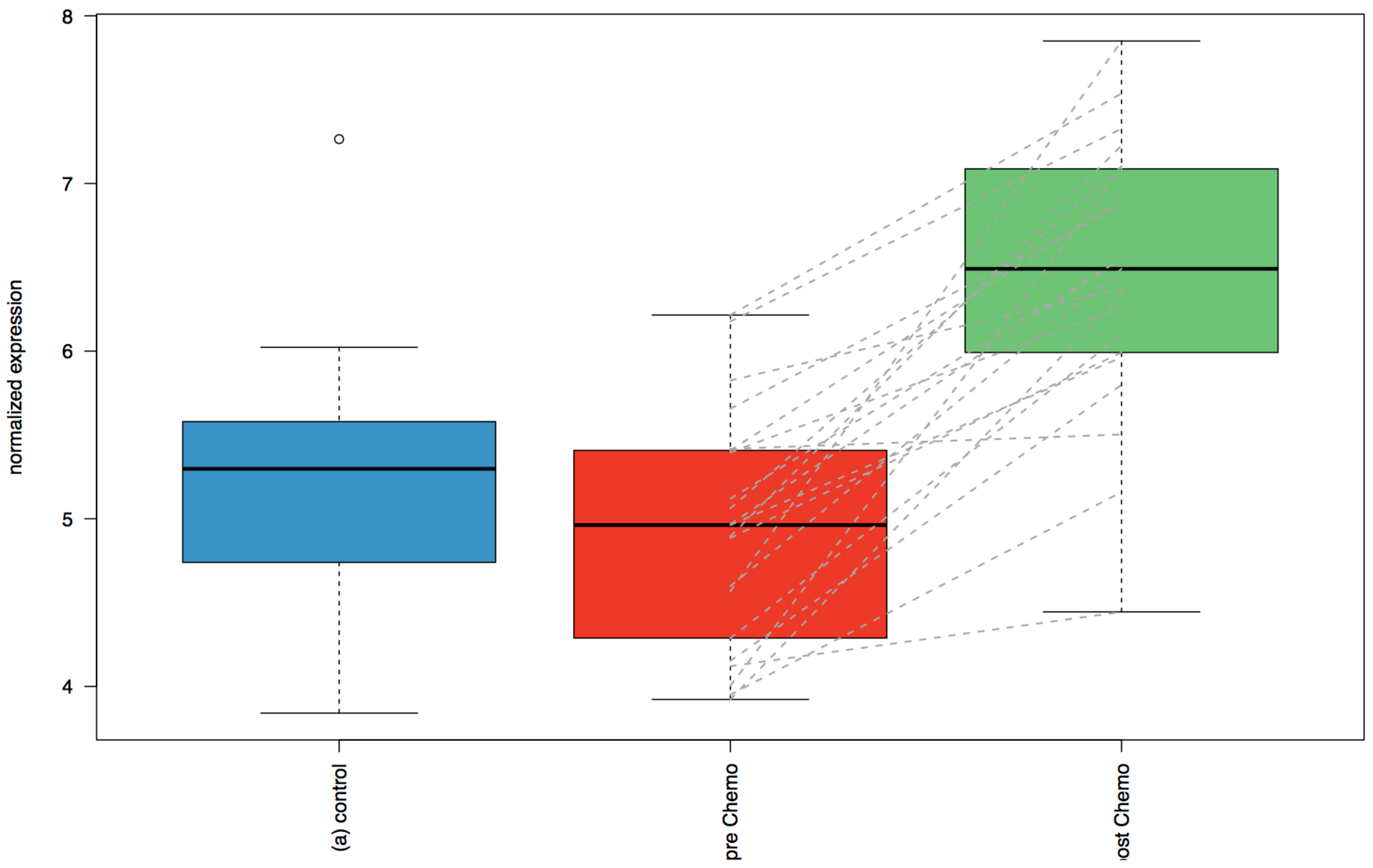
normalized expression
8
7
6
5
4
(a) control
pre Chemo
ost Chemo

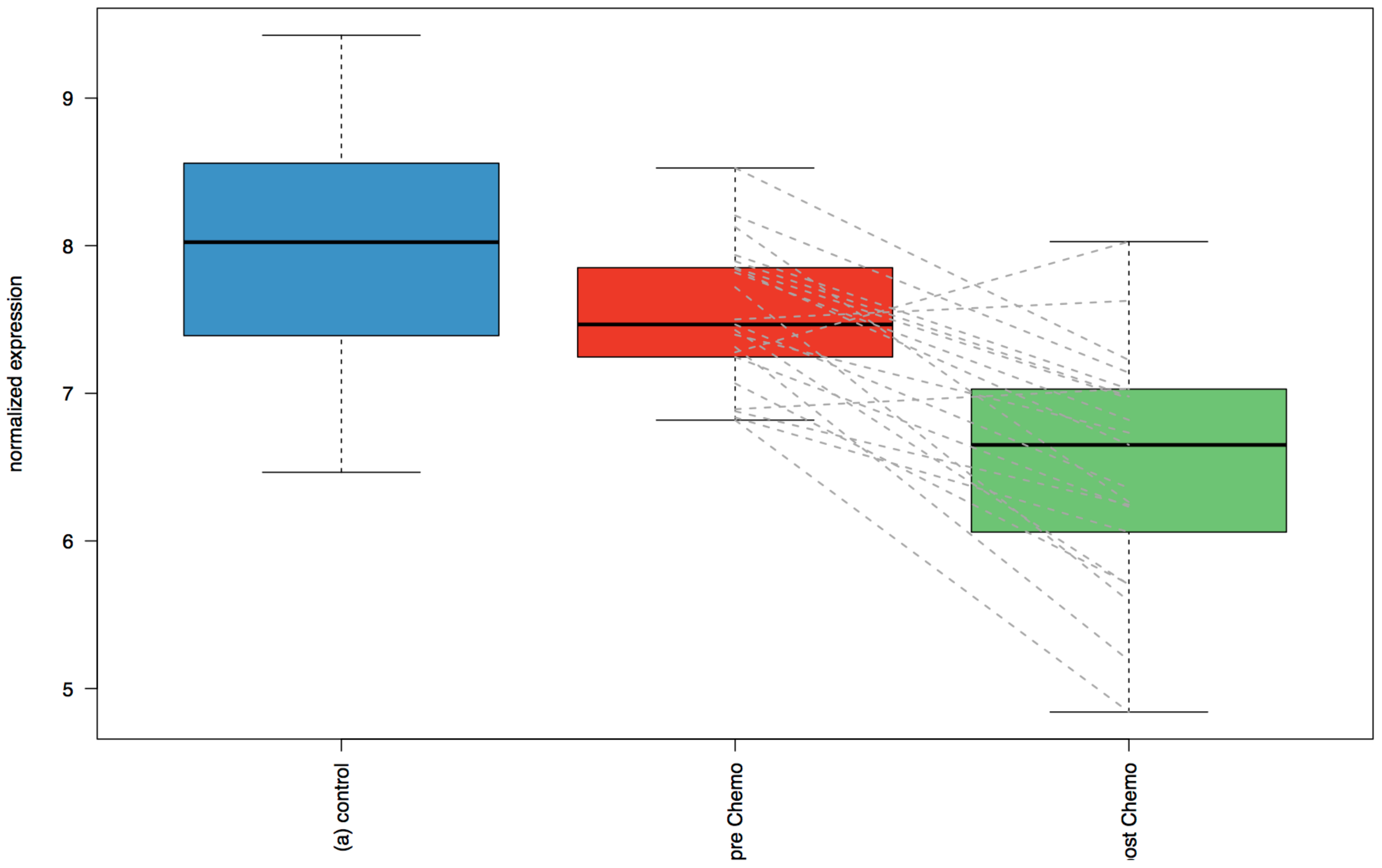
normalized expression
9
8
7
6
5
(a) control
pre Chemo
ost Chemo

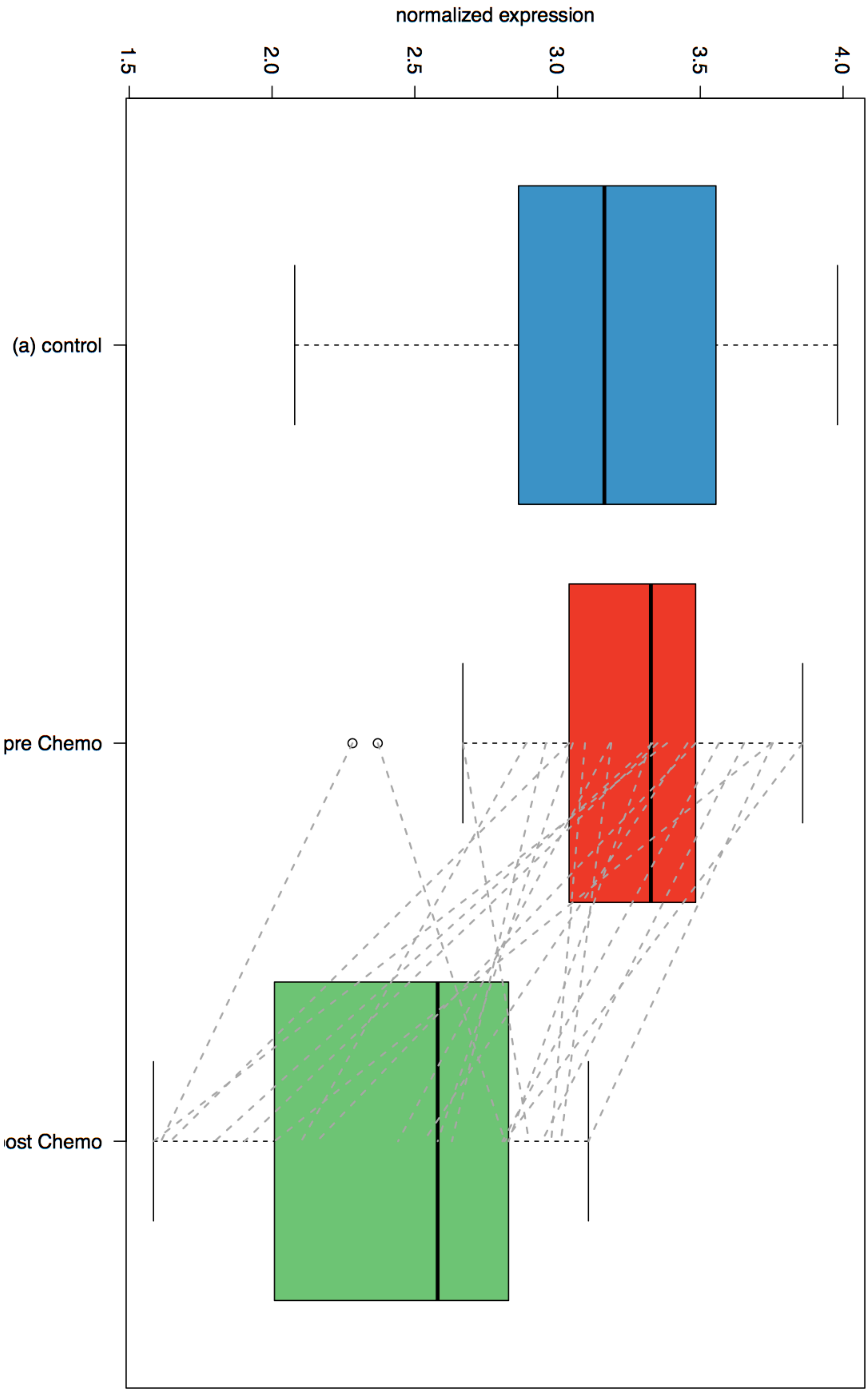
normalized expression
1.5
2.0
2.5
3.0
3.5
4.0
(a) control
pre Chemo
ost Chemo

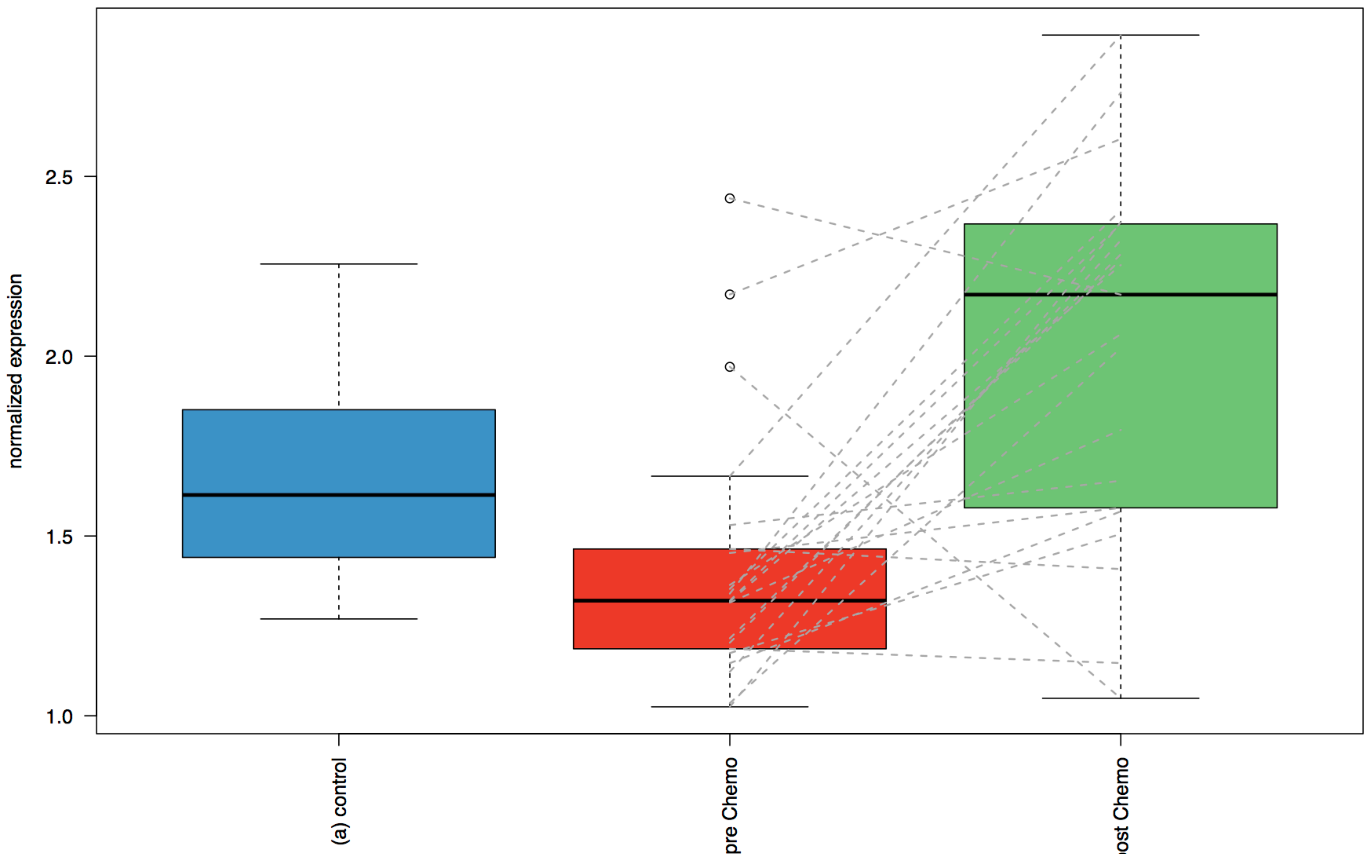
normalized expression
2.5
2.0
1.5
1.0
(a) control
pre Chemo
ost Chemo

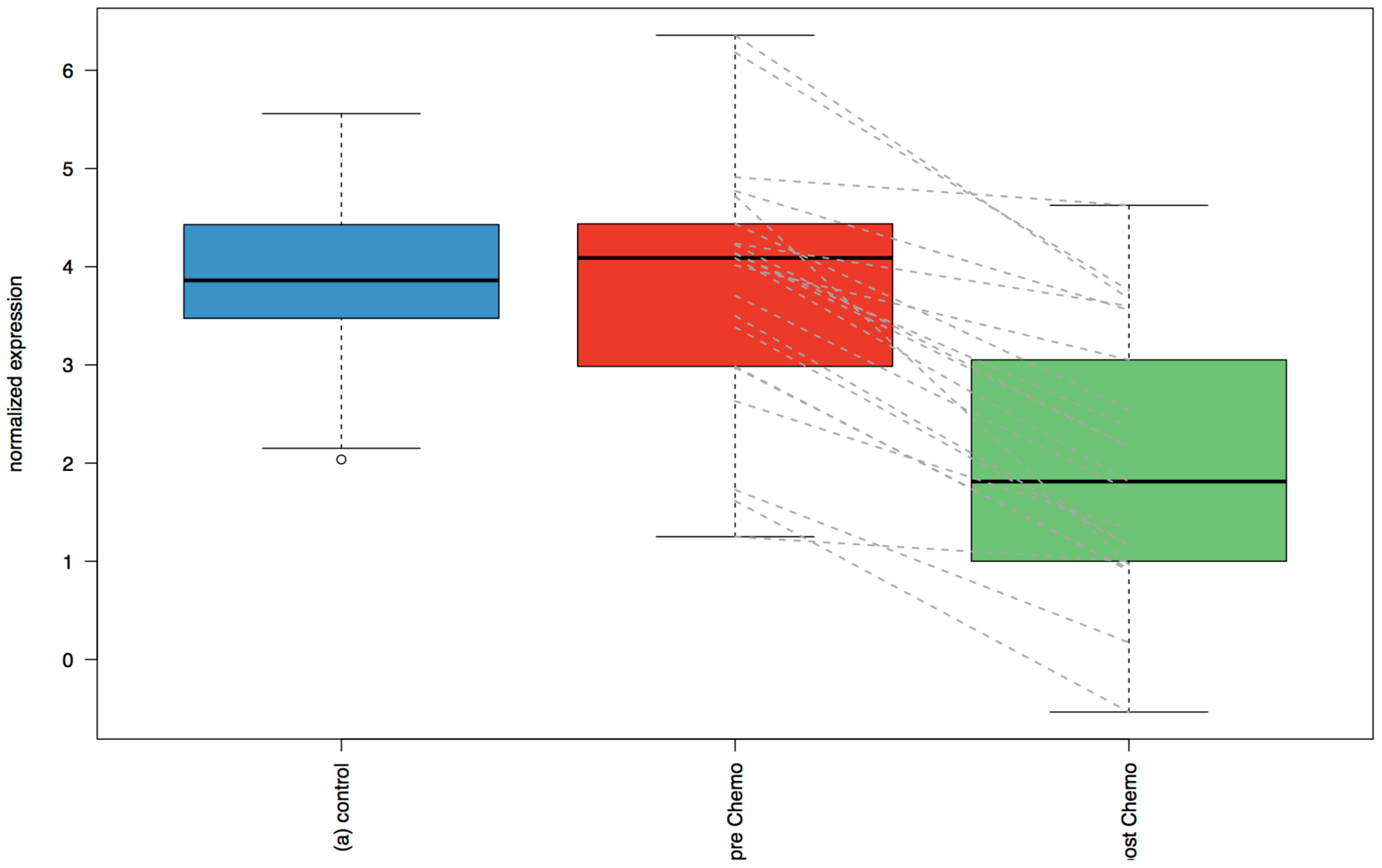
normalized expression
6
5
4
3
2
1
0
(a) control
pre Chemo
ost Chemo

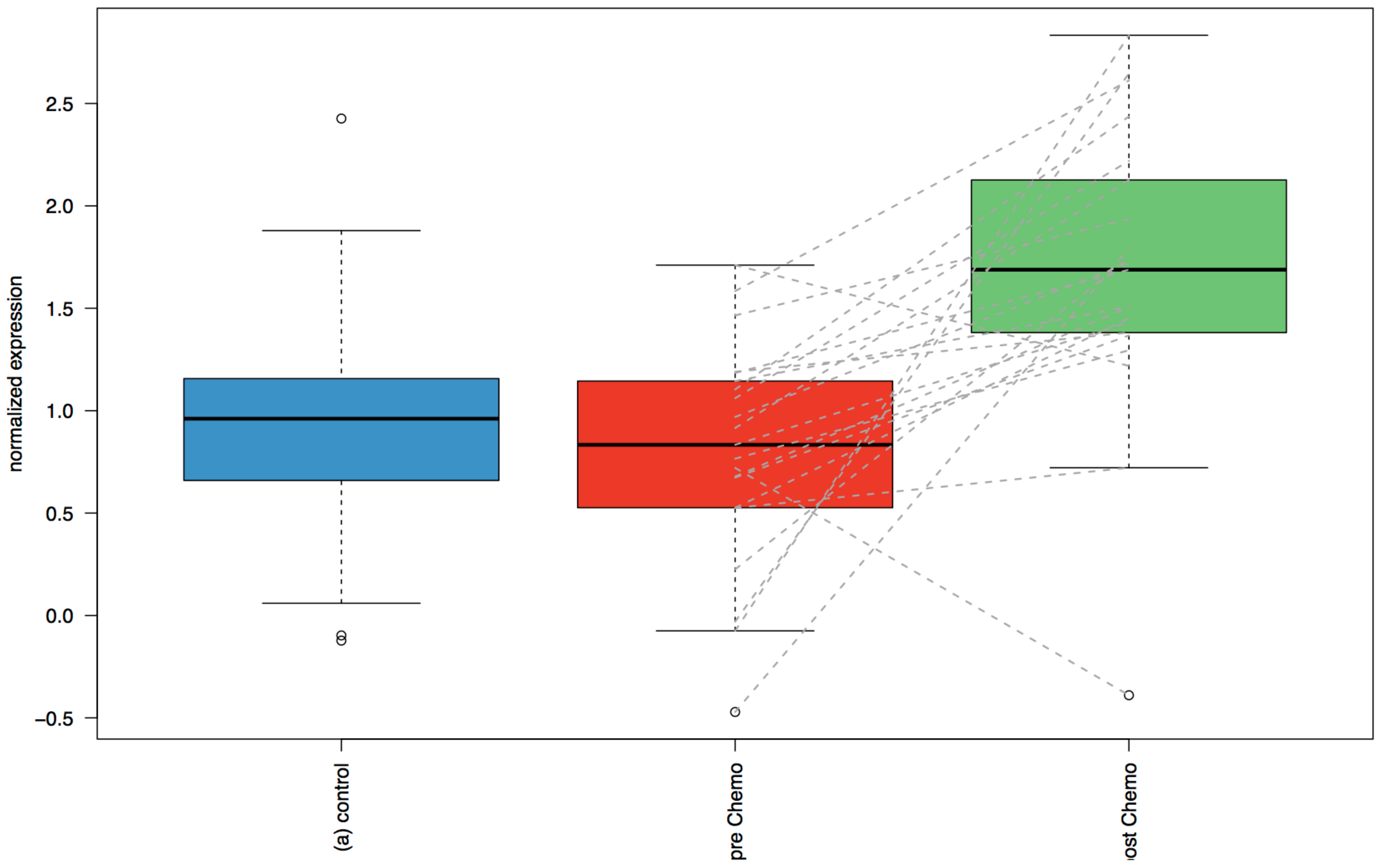
normalized expression
2.5
2.0
1.5
1.0
0.5
0.0
−0.5
(a) control
pre Chemo
ost Chemo

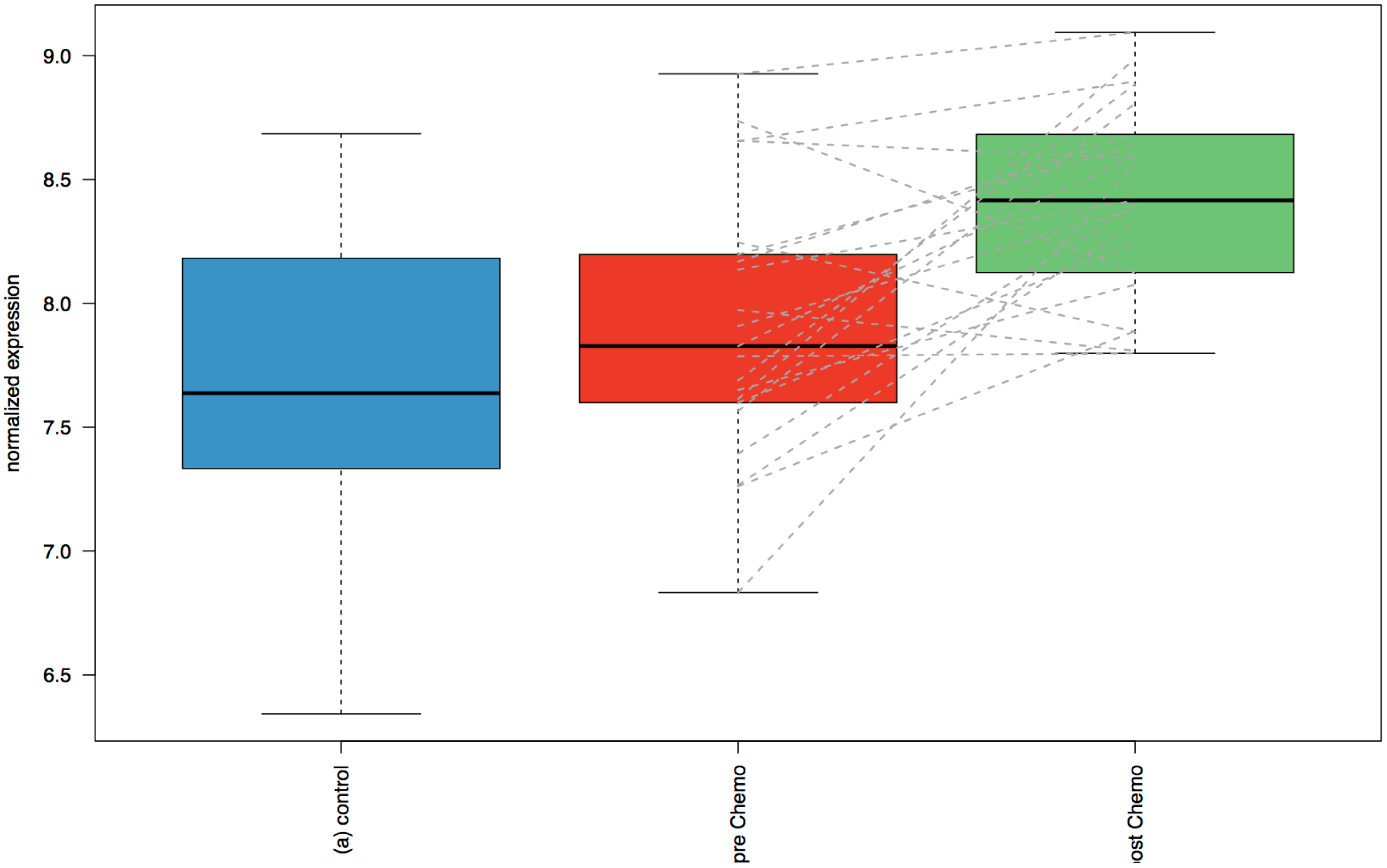
normalized expression
9.0
8.5
8.0
7.5
7.0
6.5
(a) control
pre Chemo
ost Chemo

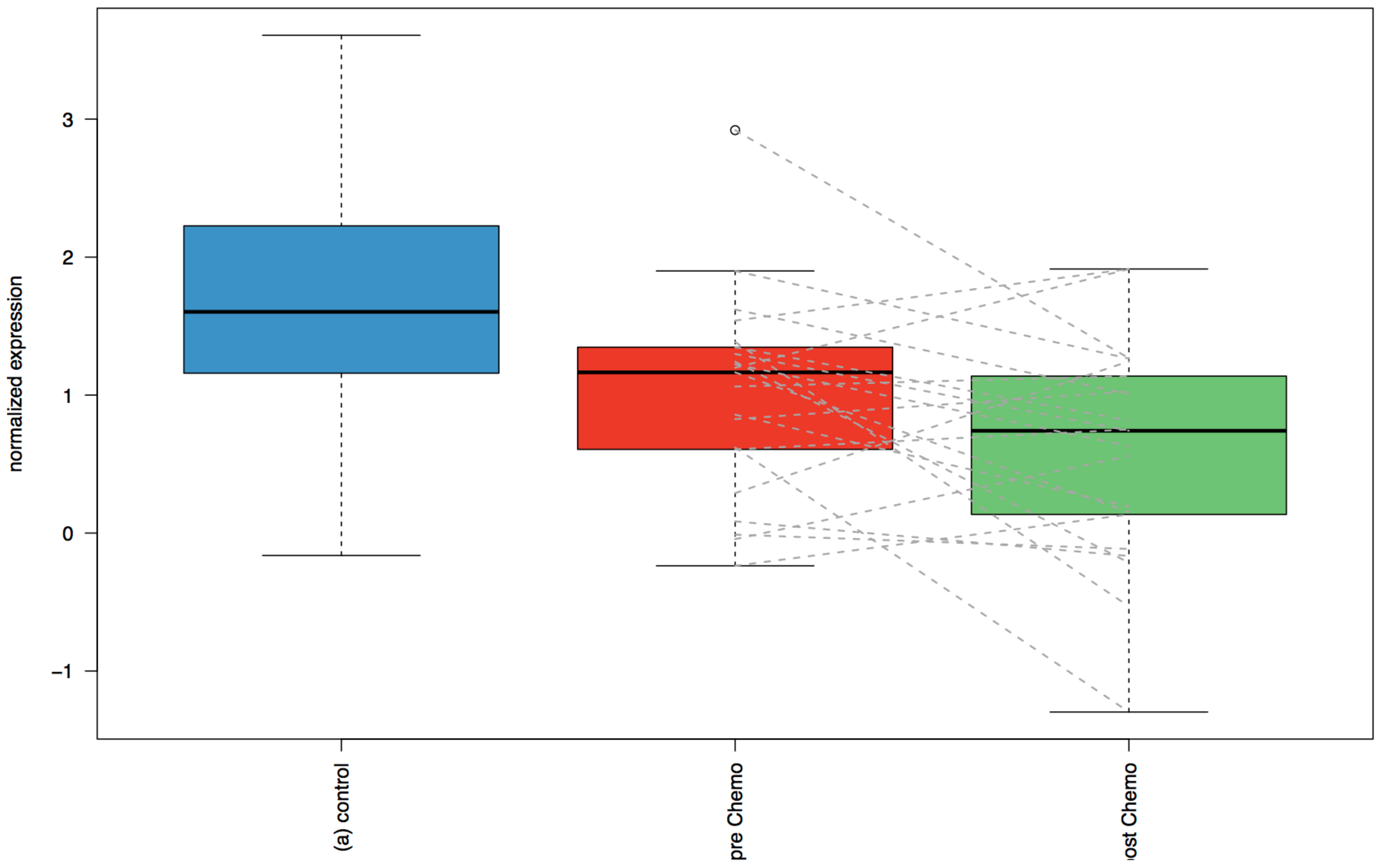
normalized expression
3
2
1
0
−1
(a) control
pre Chemo
ost Chemo

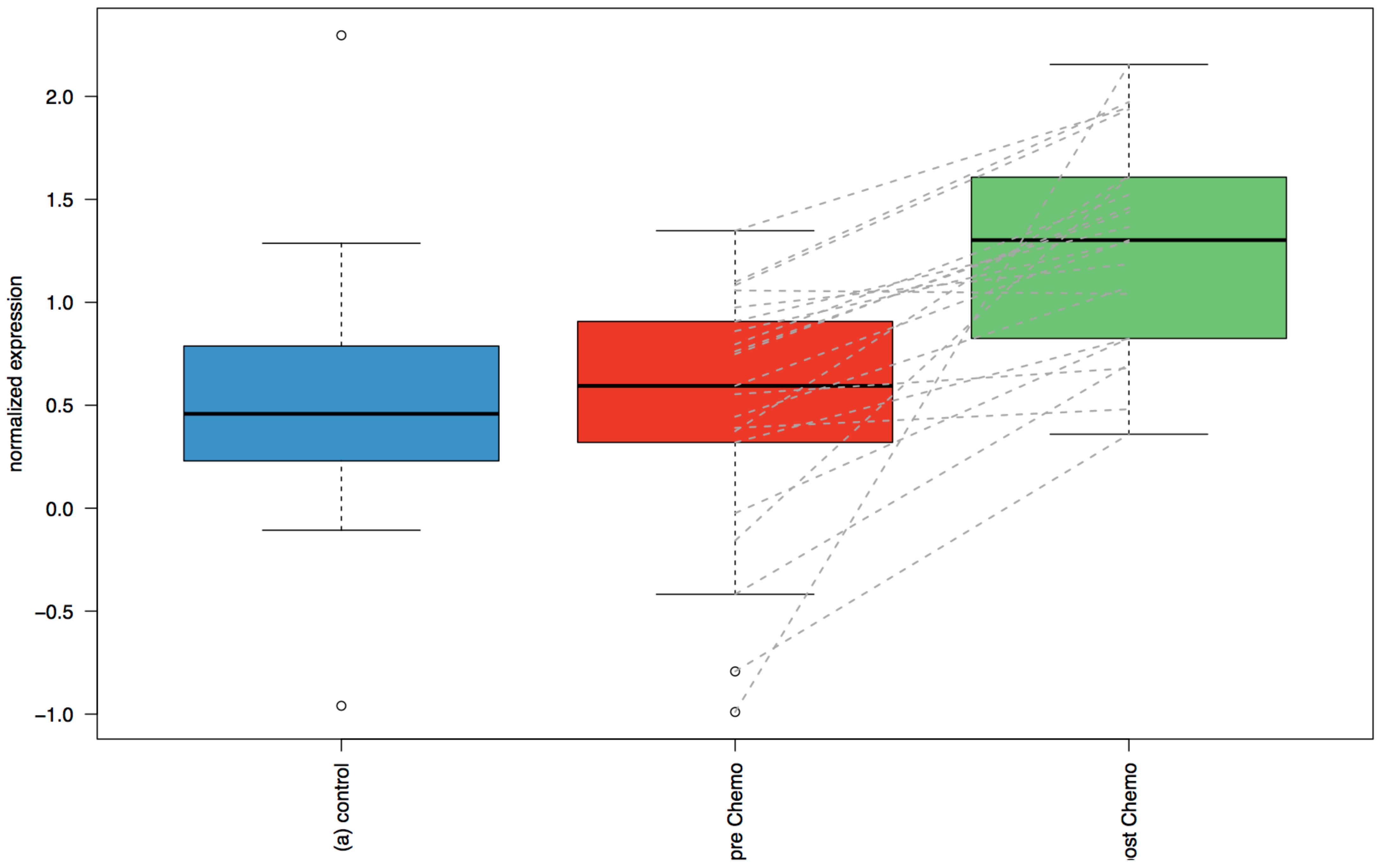
normalized expression
2.0
1.5
1.0
0.5
0.0
−0.5
−1.0
(a) control
pre Chemo
ost Chemo

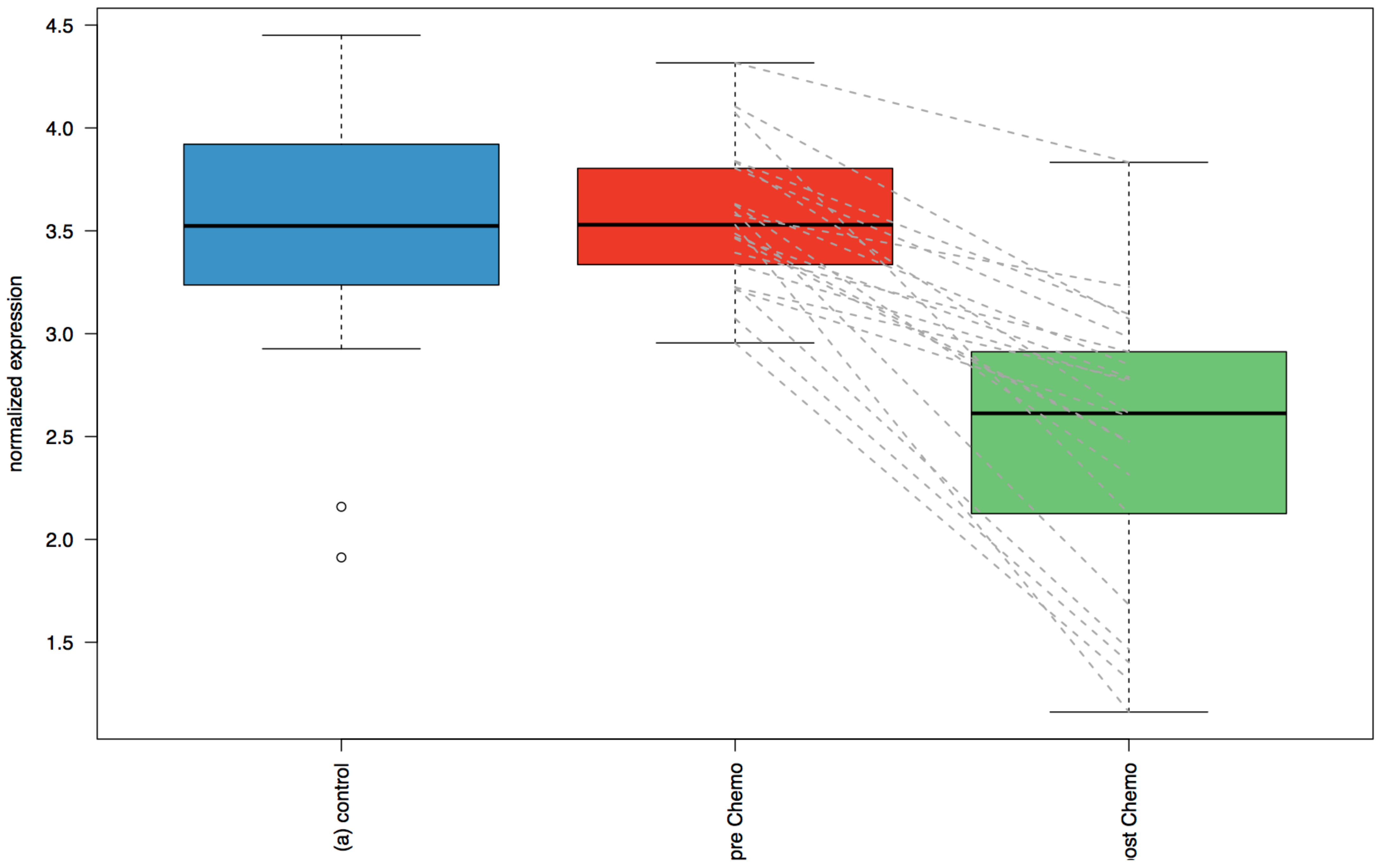
normalized expression
4.5
4.0
3.5
3.0
2.5
2.0
1.5
(a) control
pre Chemo
ost Chemo

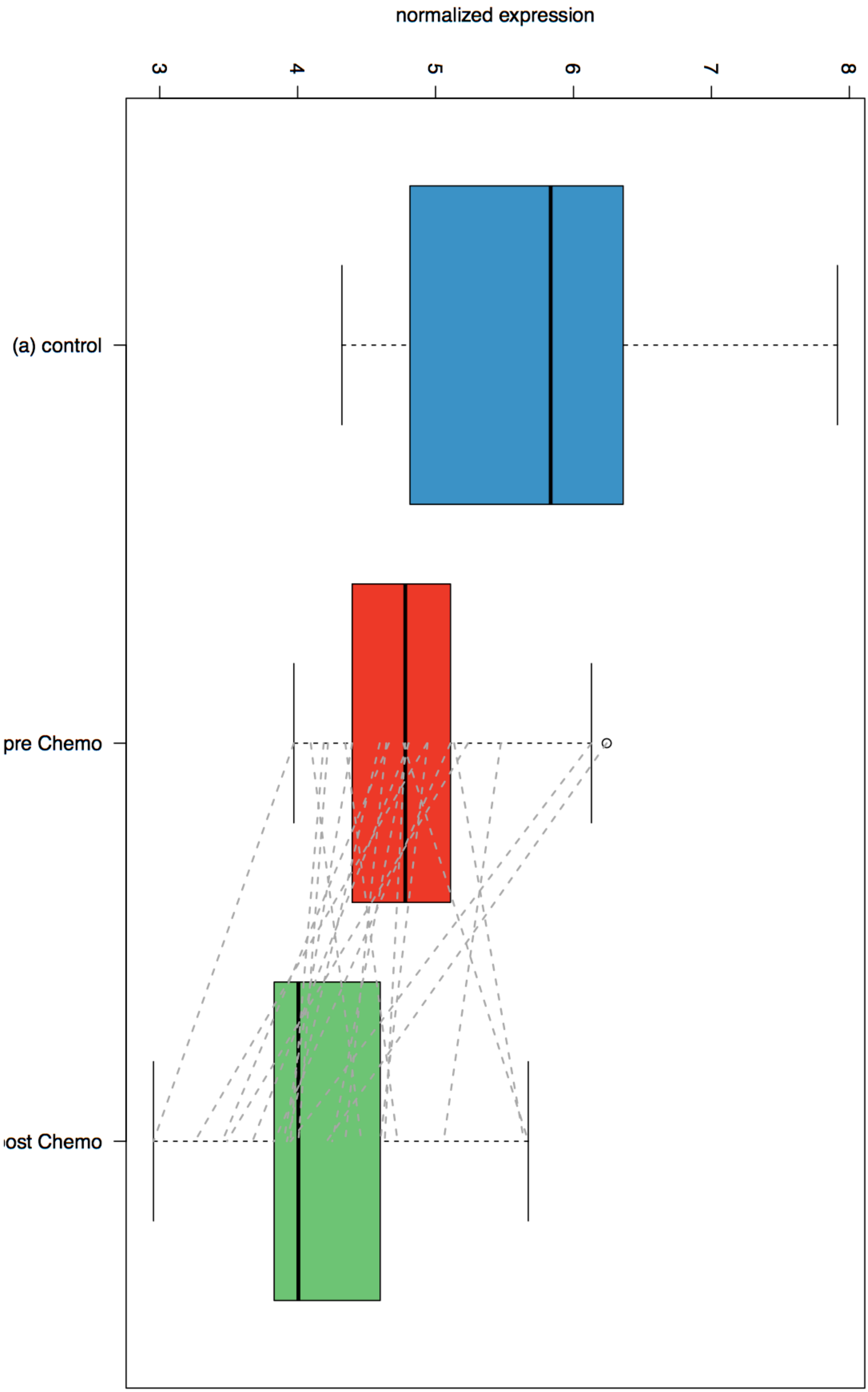

normalized expression
3
4
5
6
7
8
(a) control
pre Chemo
post Chemo

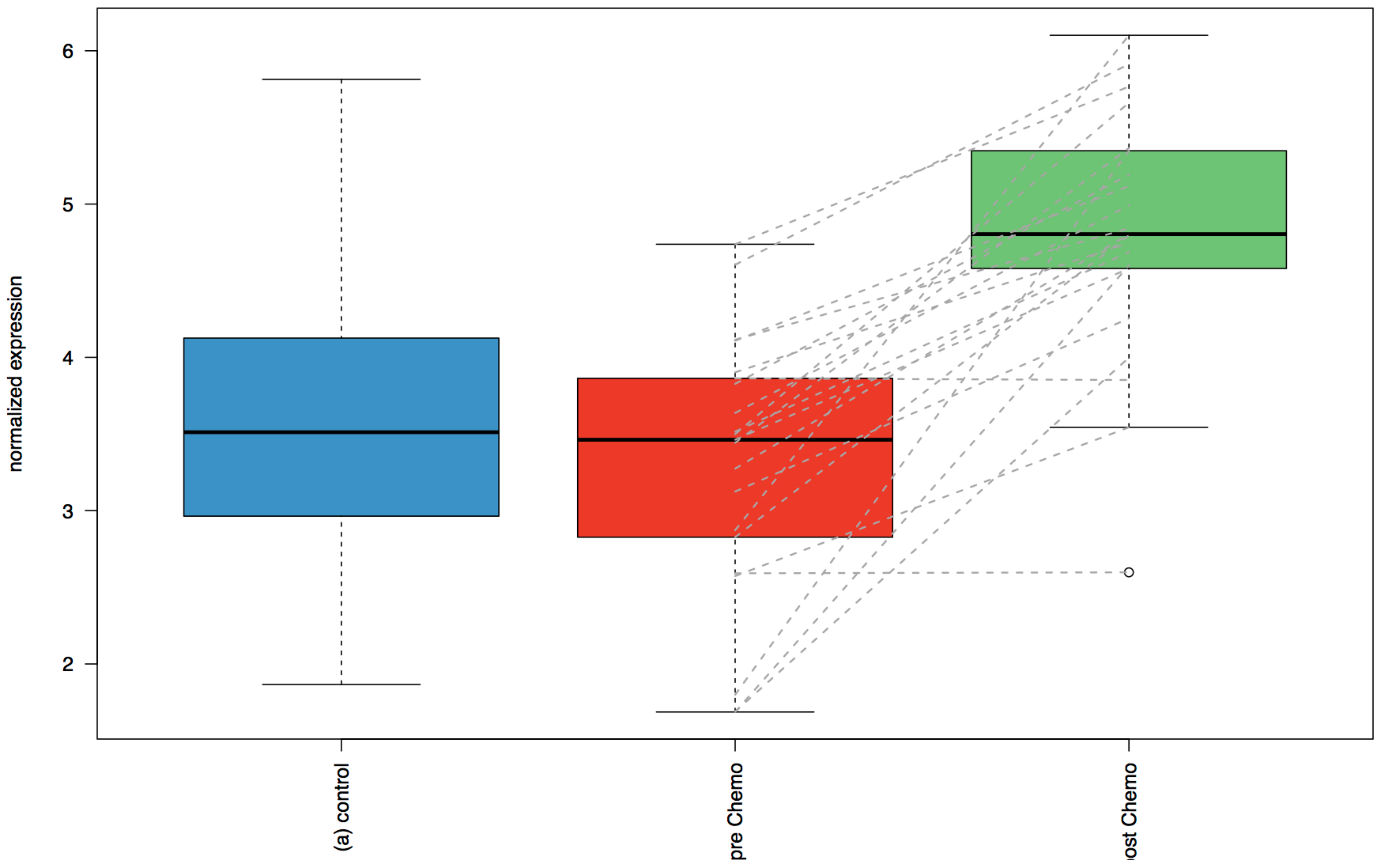
normalized expression
6
5
4
3
2
(a) control
pre Chemo
ost Chemo

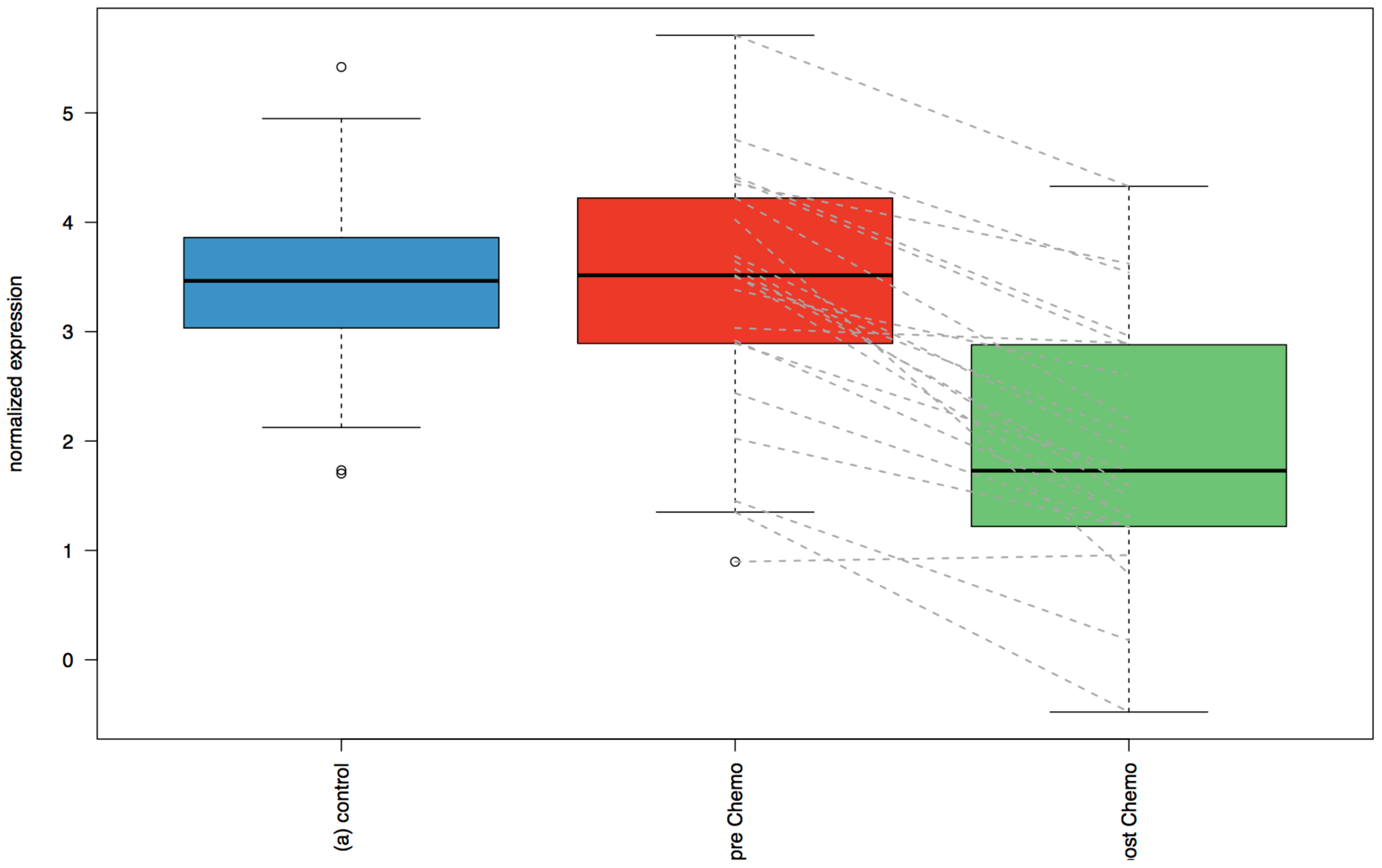
normalized expression
0
1
2
3
4
5
(a) control
pre Chemo
ost Chemo

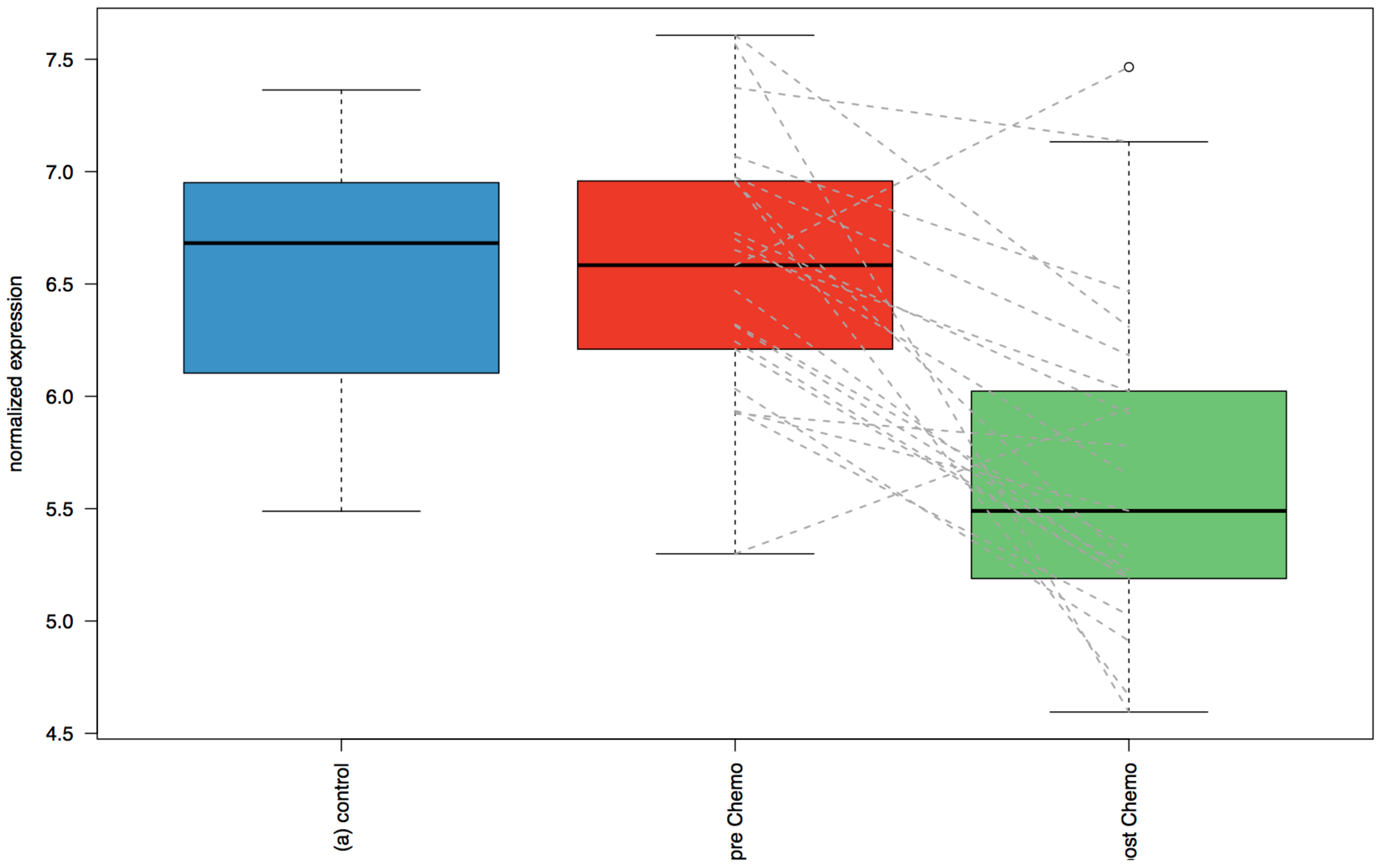

normalized expression
7.5
7.0
6.5
6.0
5.5
5.0
4.5
(a) control
pre Chemo
ost Chemo

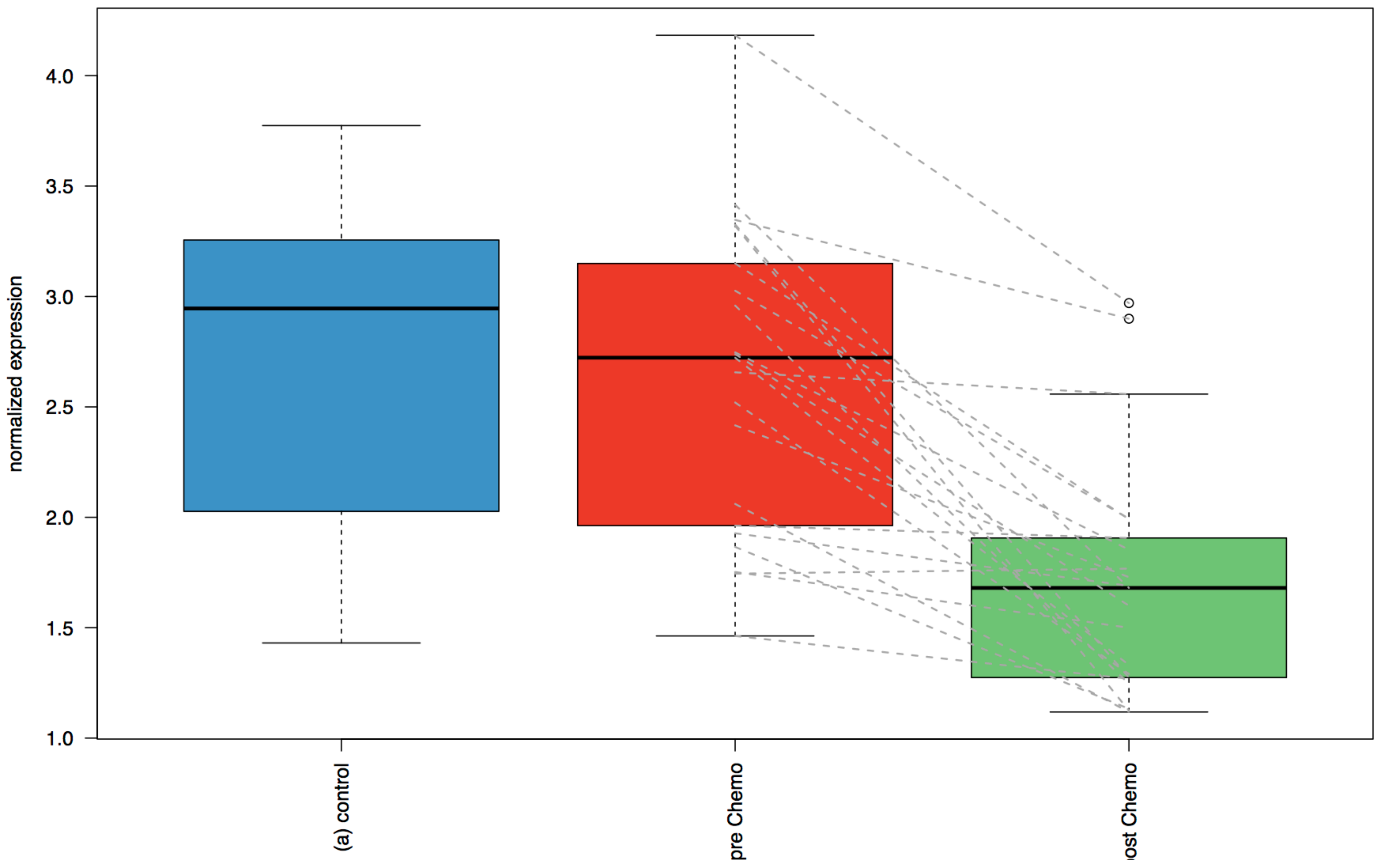

normalized expression
4.0
3.5
3.0
2.5
2.0
1.5
1.0
(a) control
pre Chemo
ost Chemo

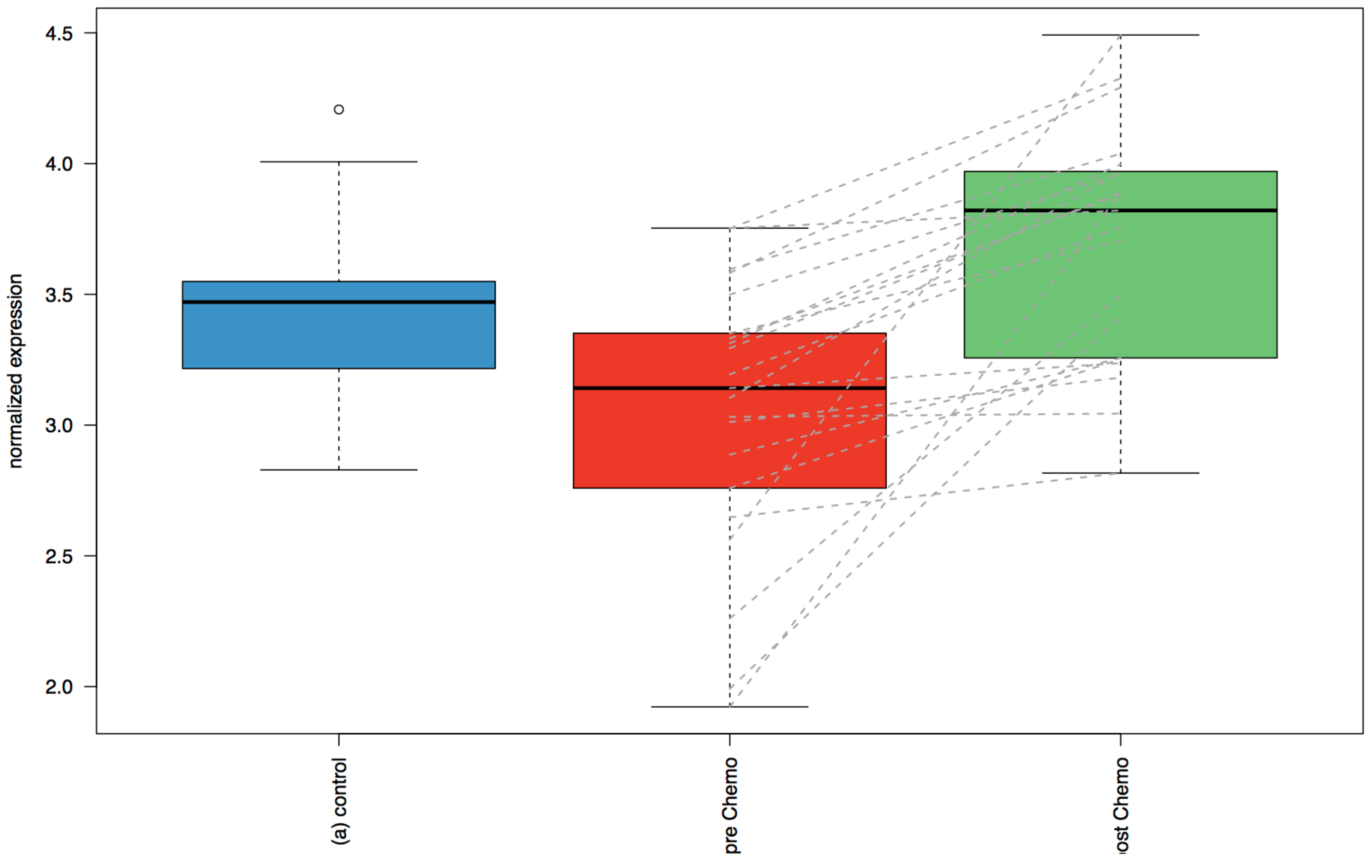
normalized expression
4.5
4.0
3.5
3.0
2.5
2.0
(a) control
pre Chemo
ost Chemo

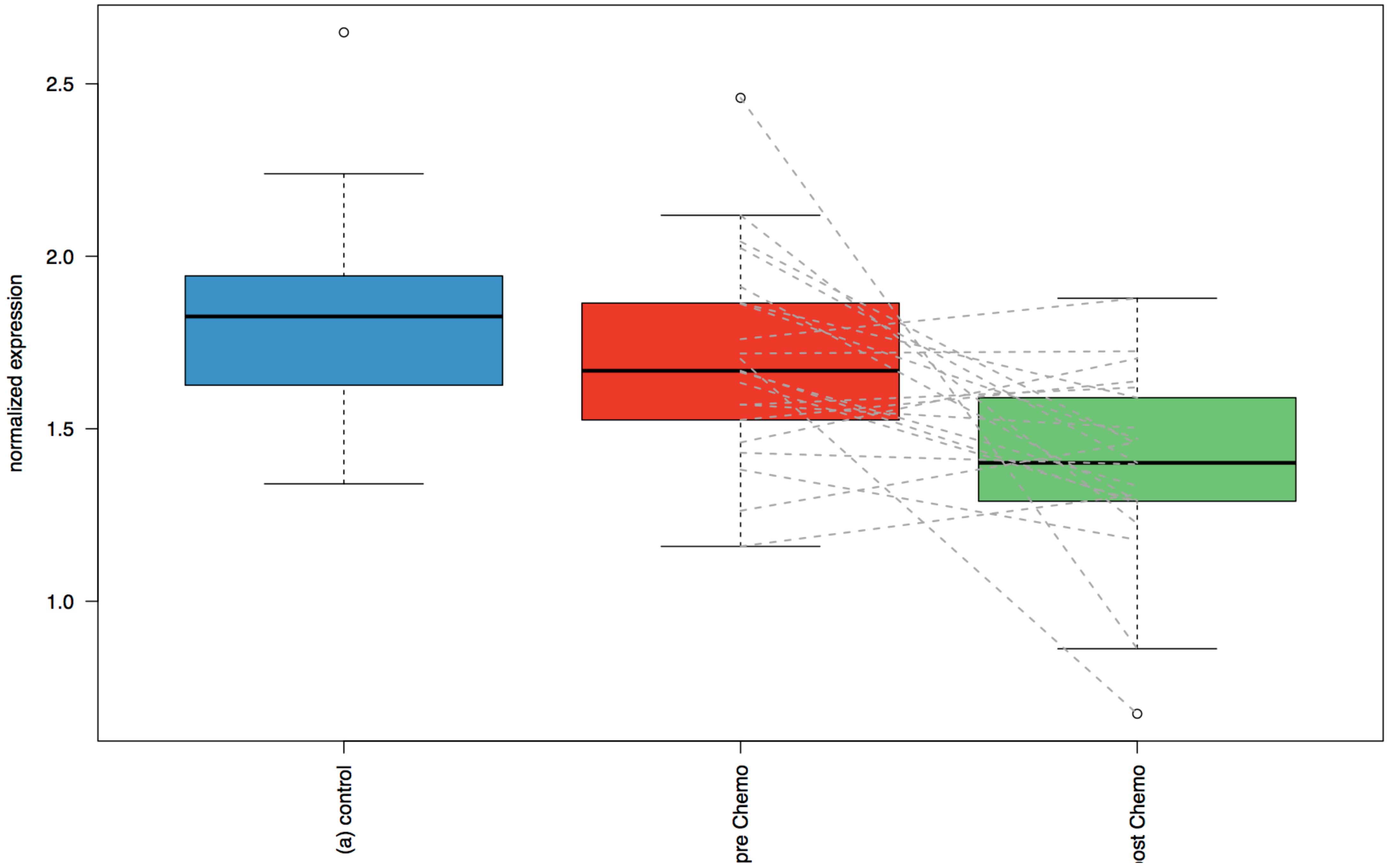
normalized expression
2.5
2.0
1.5
1.0
(a) control
pre Chemo
ost Chemo

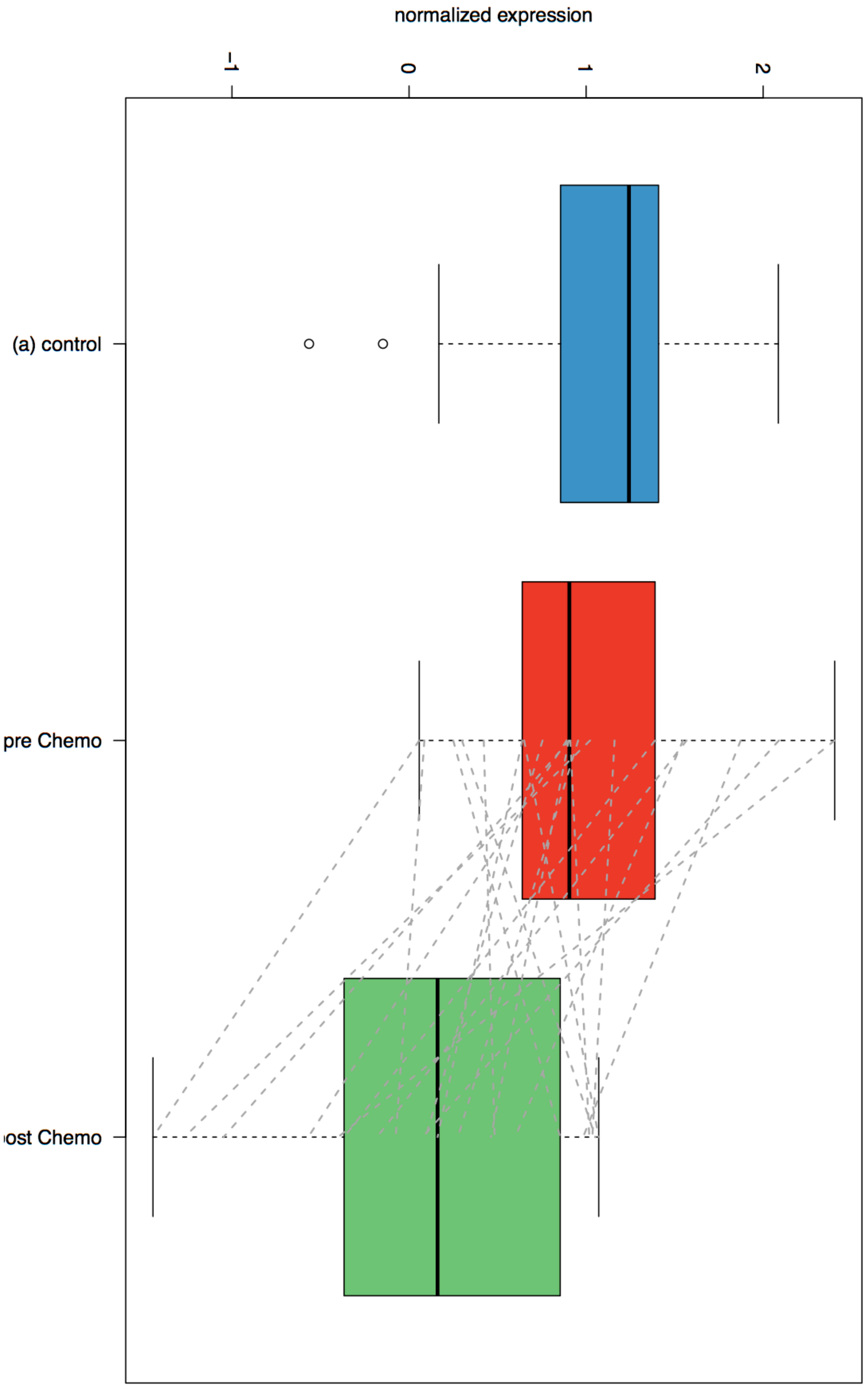

normalized expression
-1
0
1
2
(a) control
pre Chemo
ost Chemo

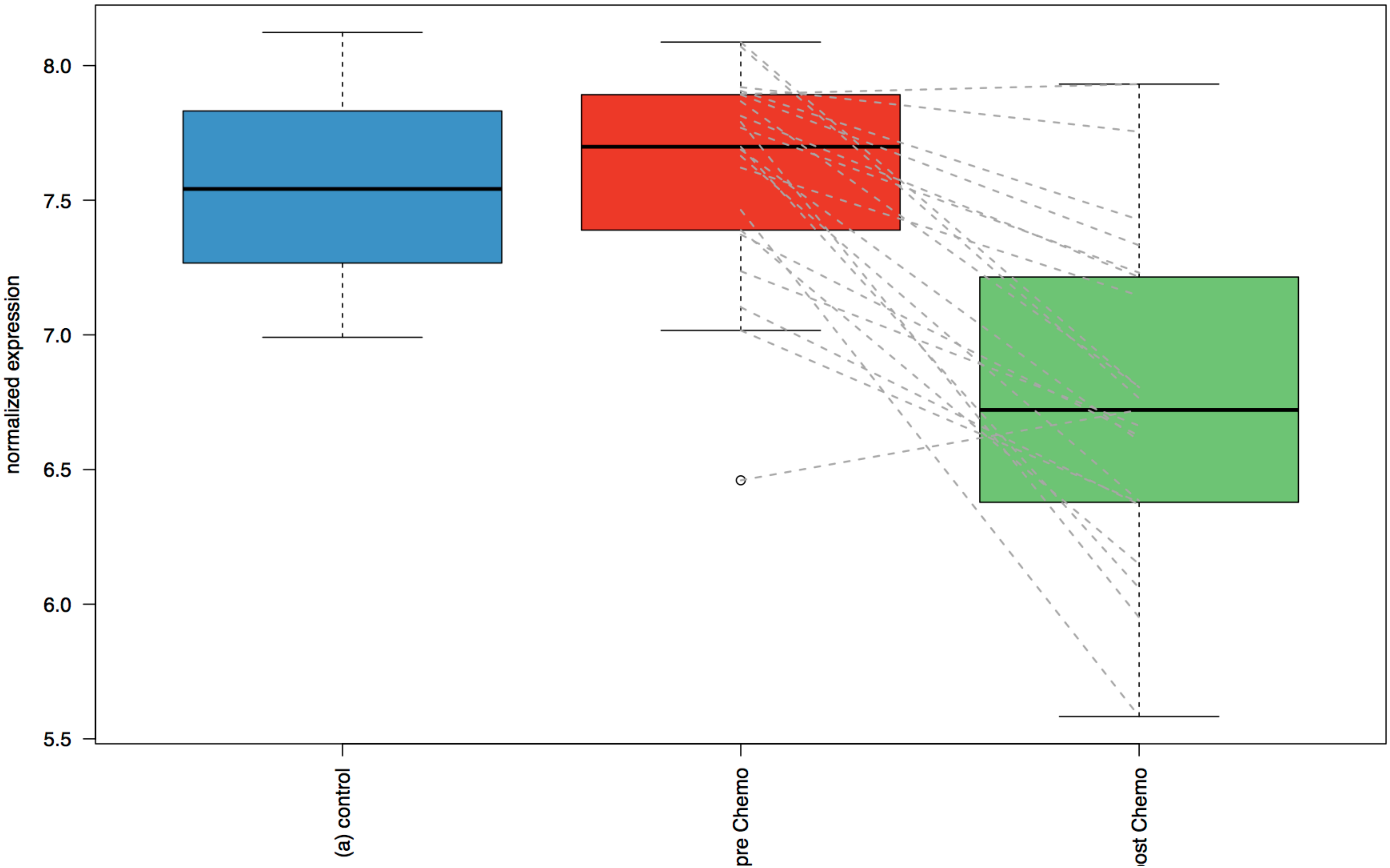
normalized expression
8.0
7.5
7.0
6.5
6.0
5.5
(a) control
pre Chemo
ost Chemo

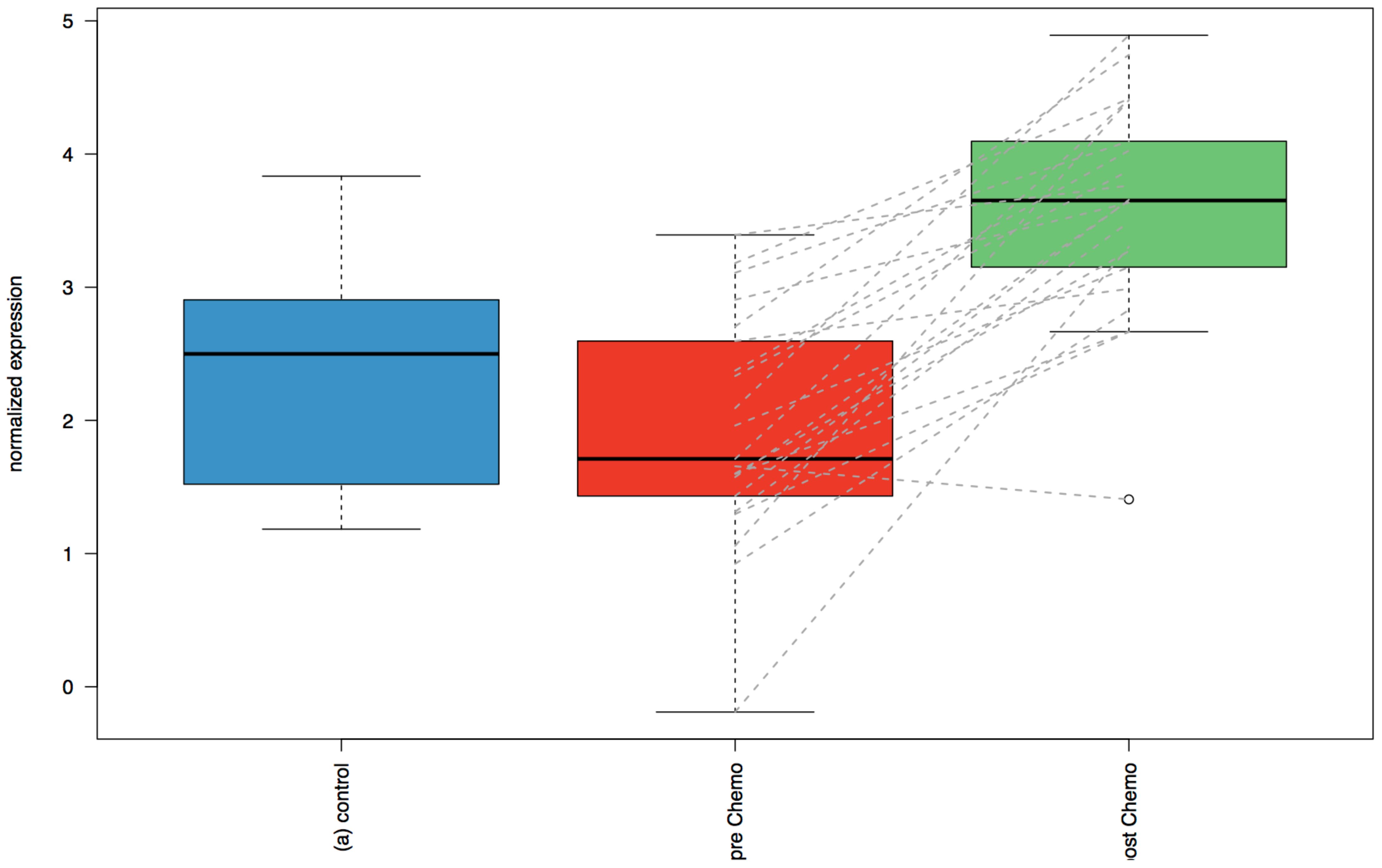
normalized expression
5
4
3
2
1
0
(a) control
pre Chemo
ost Chemo

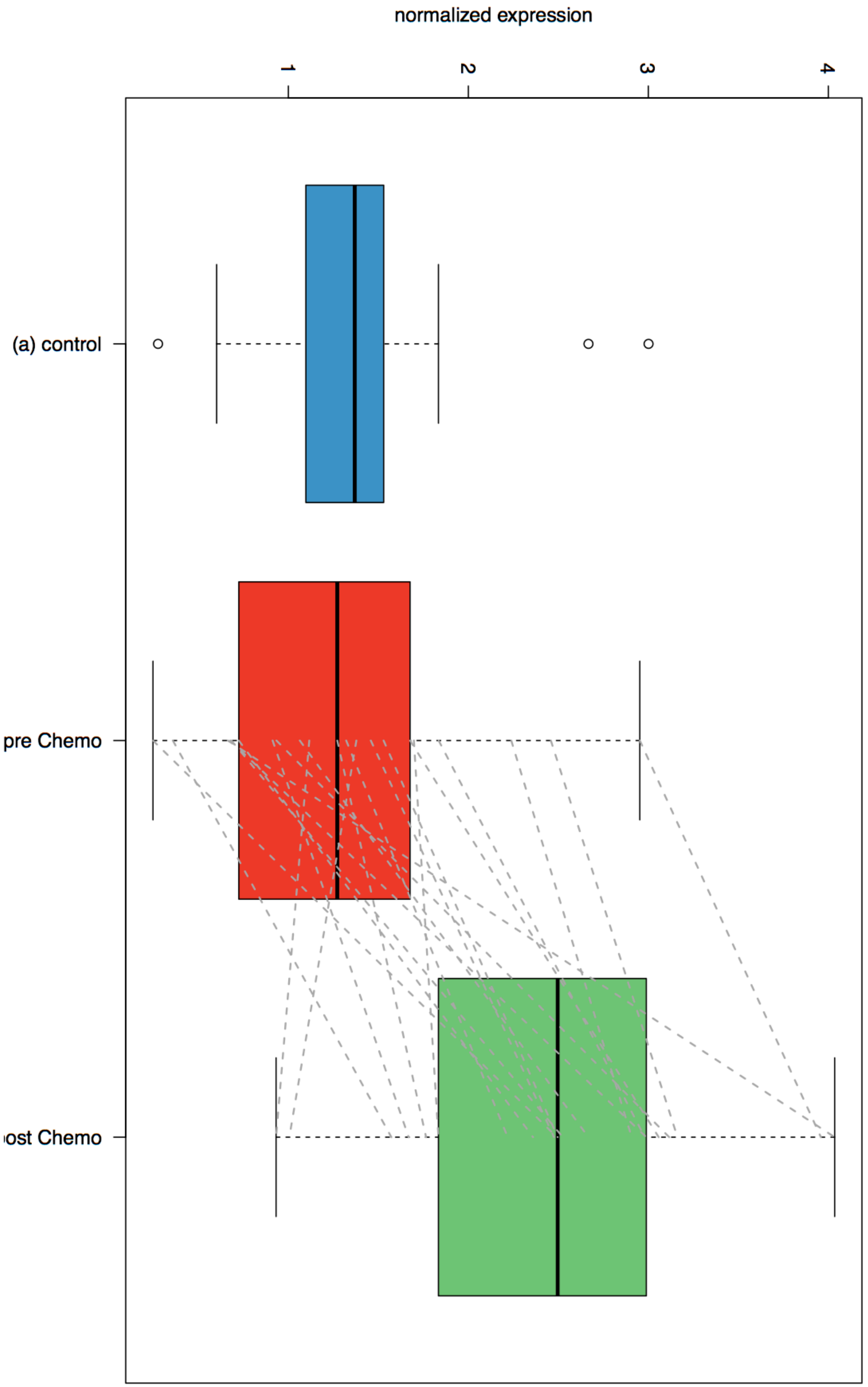
normalized expression
1
2
3
4
(a) control
pre Chemo
ost Chemo

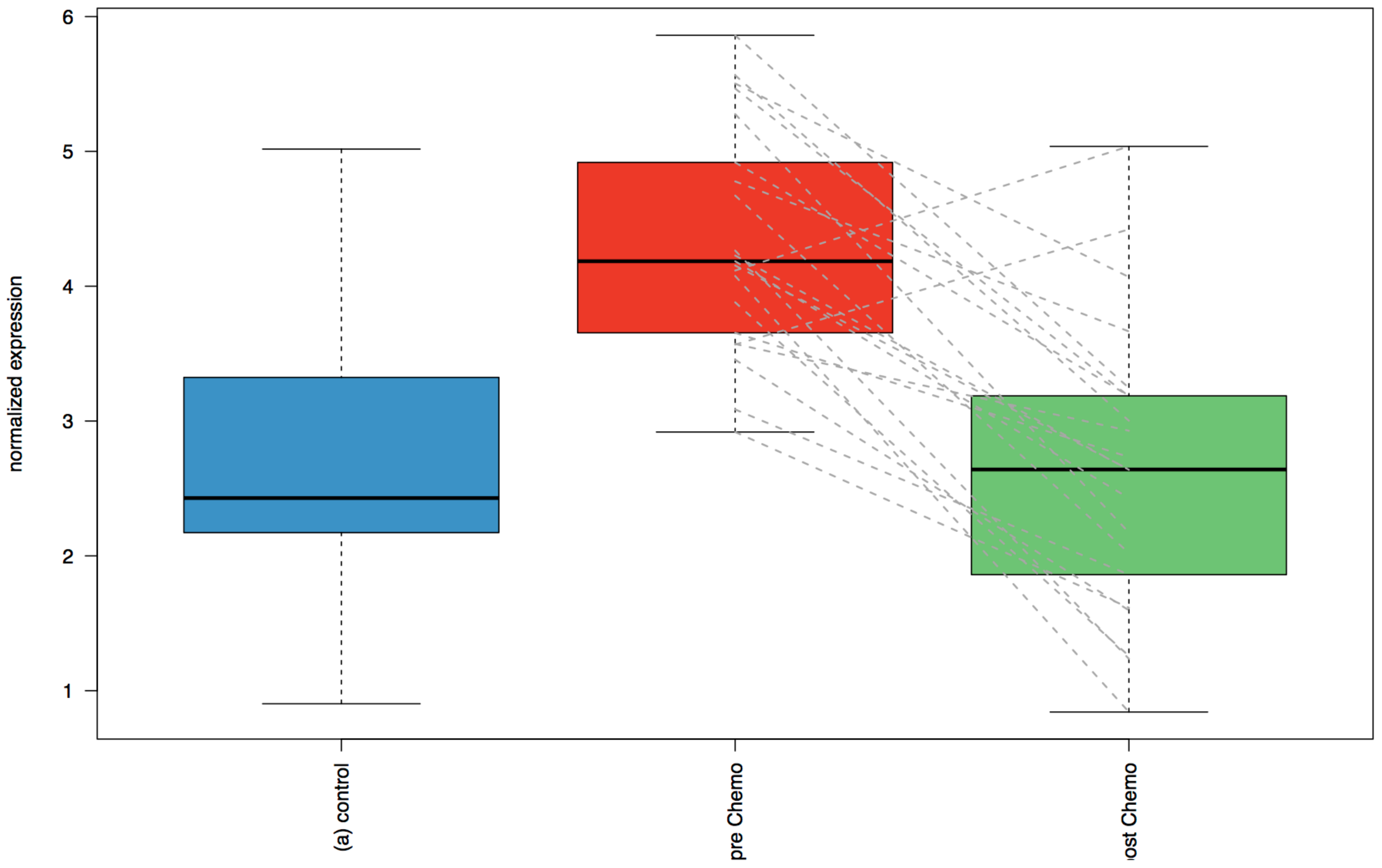
normalized expression
6
5
4
3
2
1
(a) control
pre Chemo
ost Chemo

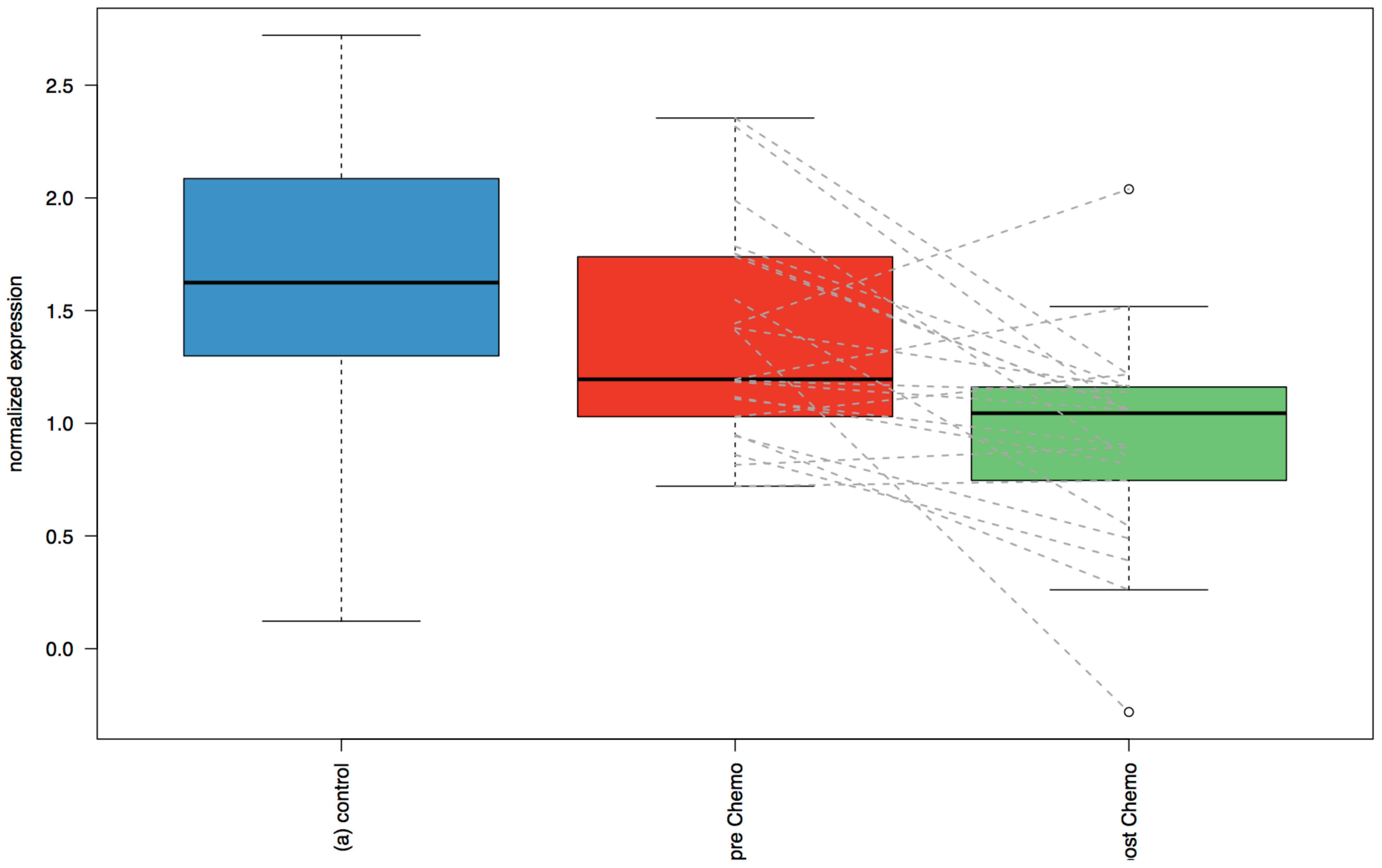
normalized expression
2.5
2.0
1.5
1.0
0.5
0.0
(a) control
pre Chemo
ost Chemo

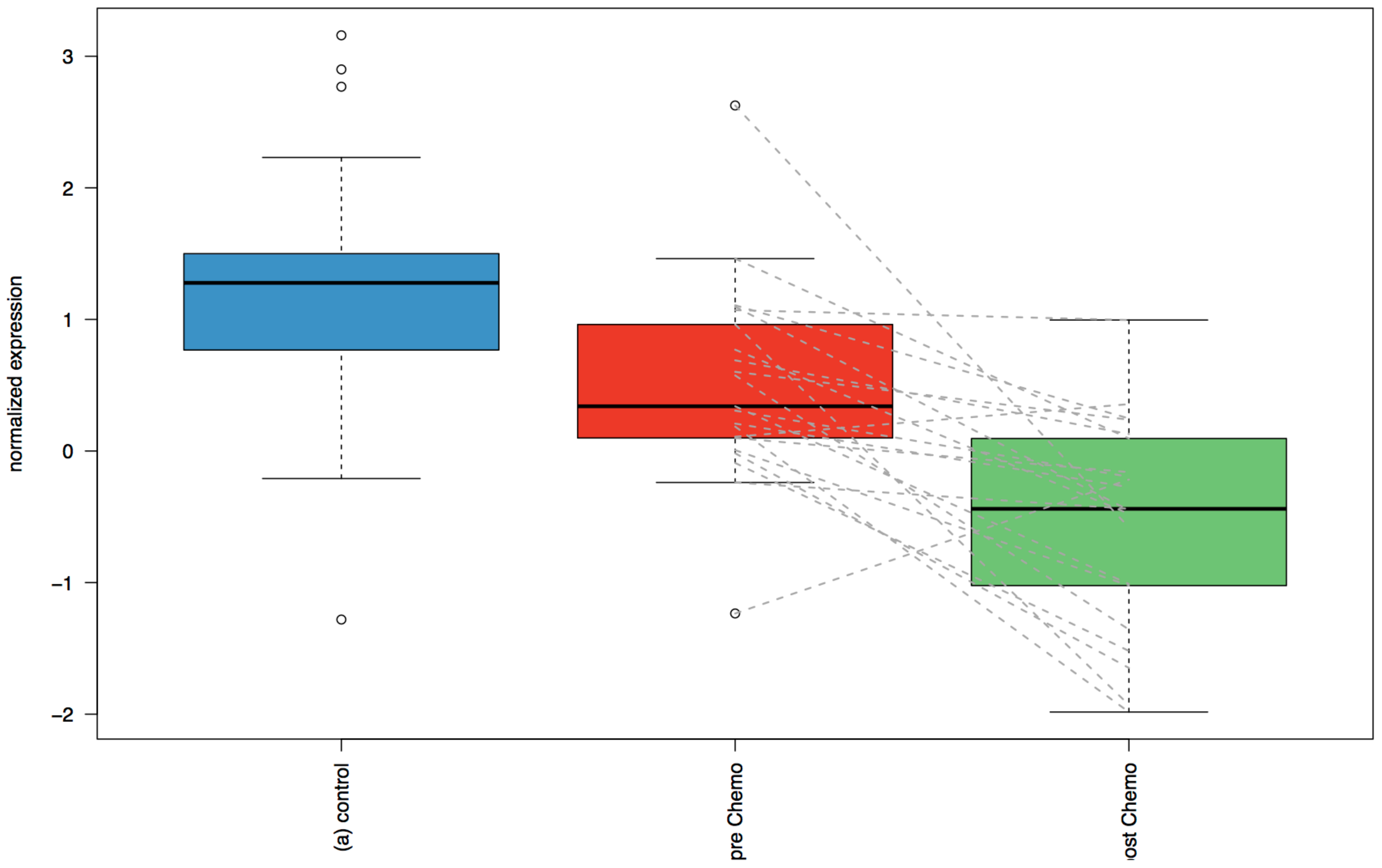
normalized expression
3
2
1
0
−1
−2
(a) control
pre Chemo
ost Chemo

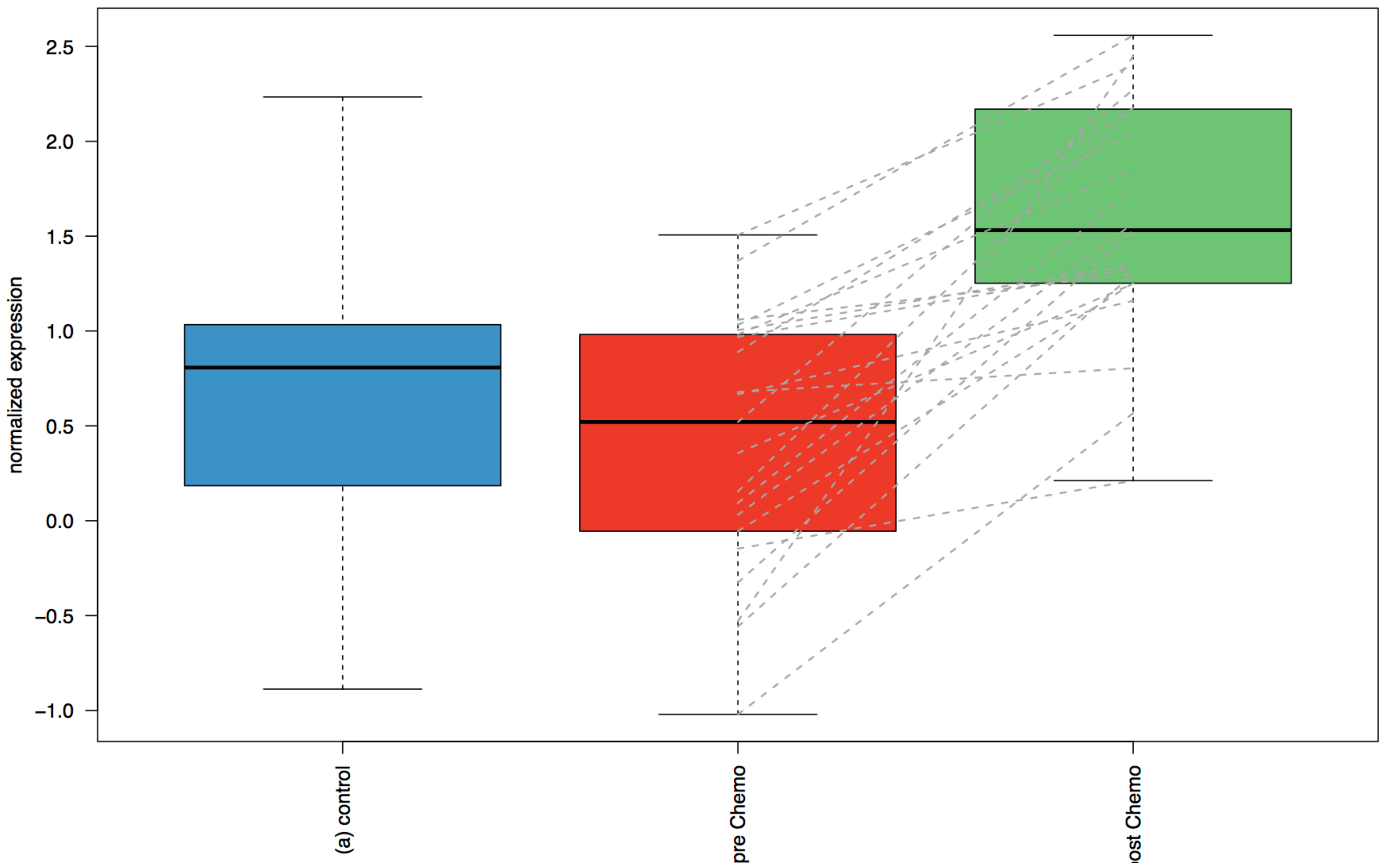
normalized expression
2.5
2.0
1.5
1.0
0.5
0.0
−0.5
−1.0
(a) control
pre Chemo
ost Chemo

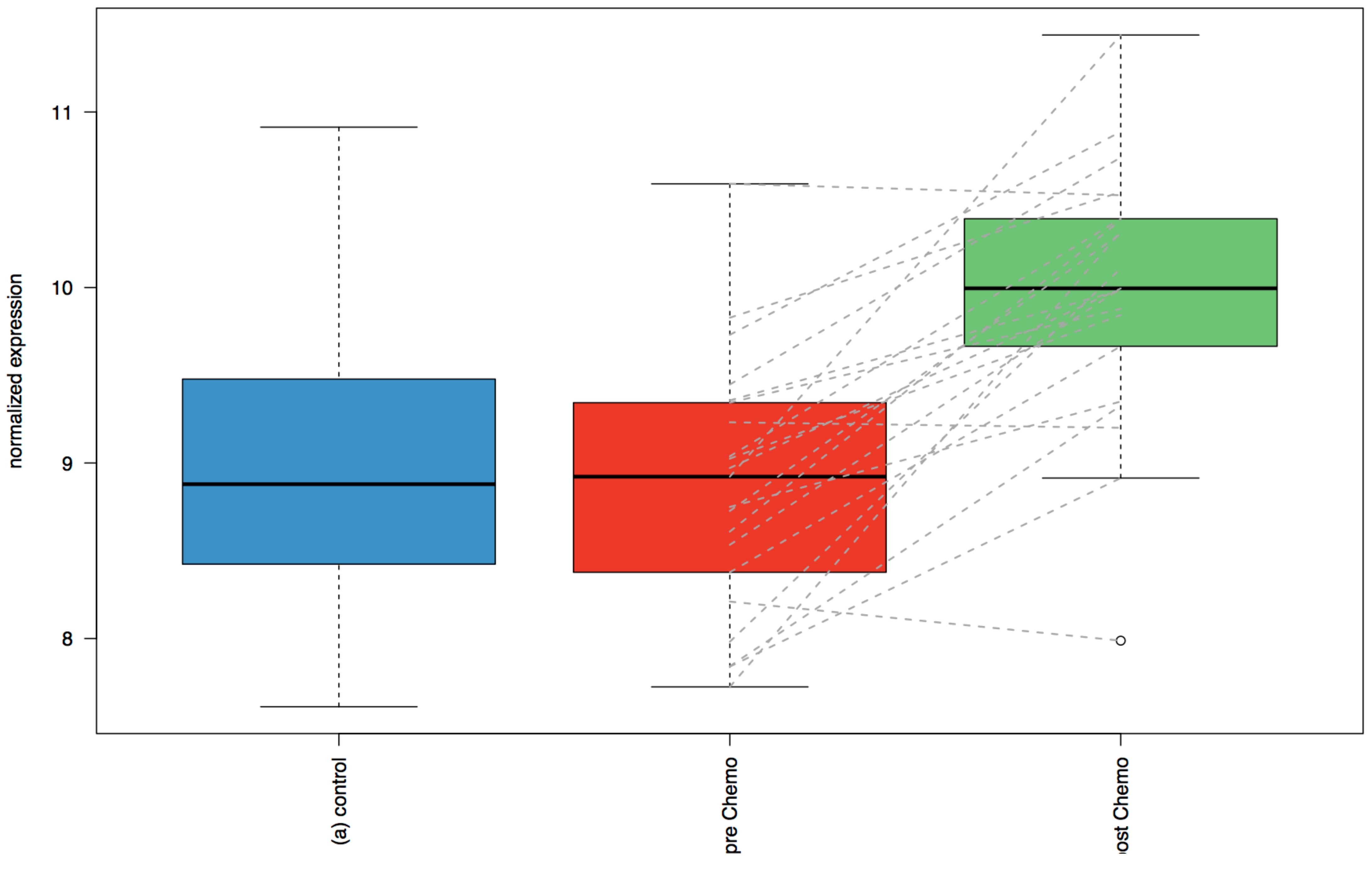

normalized expression
11
10
9
8
(a) control
pre Chemo
ost Chemo

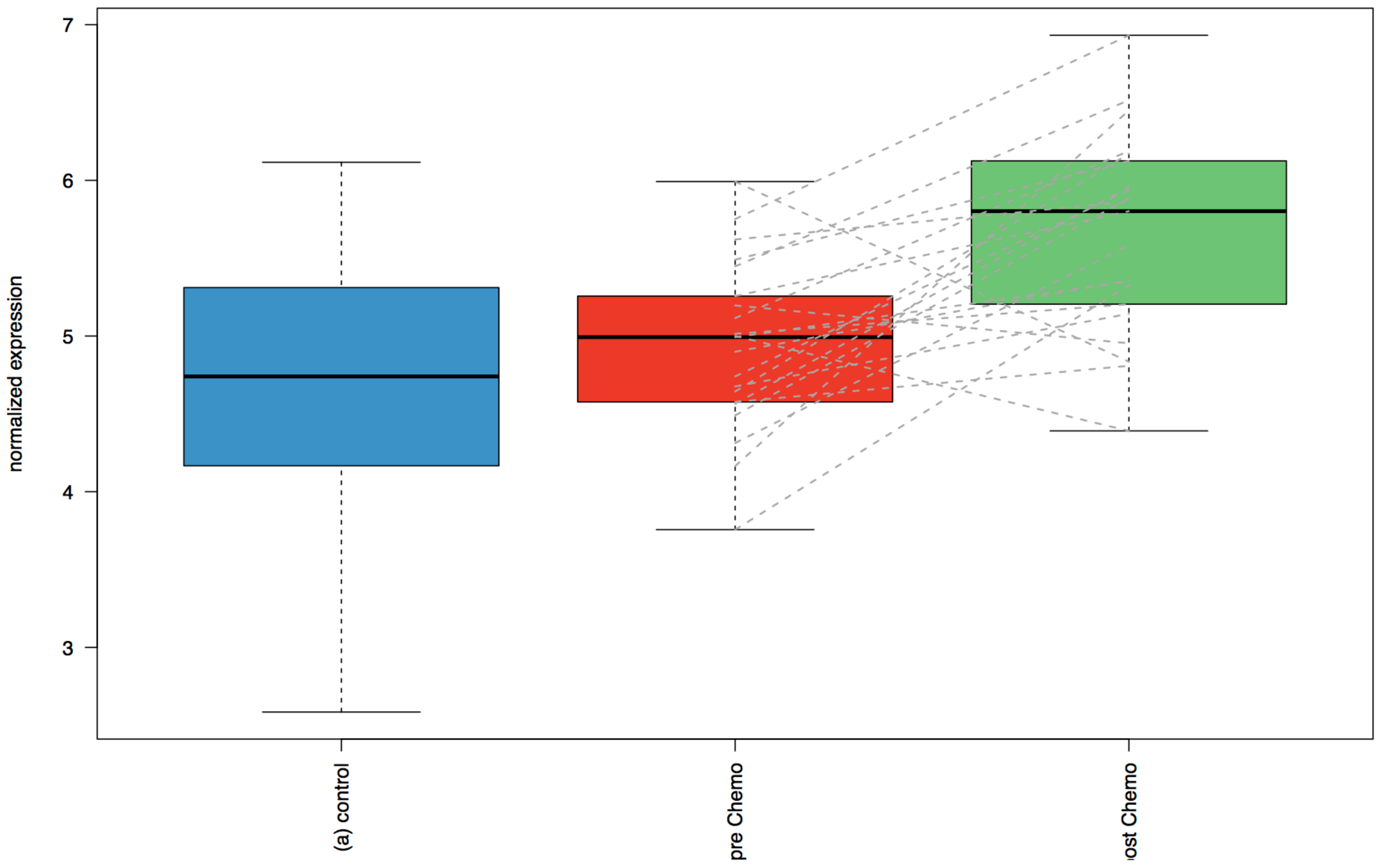

normalized expression
7
6
5
4
3
(a) control
pre Chemo
ost Chemo

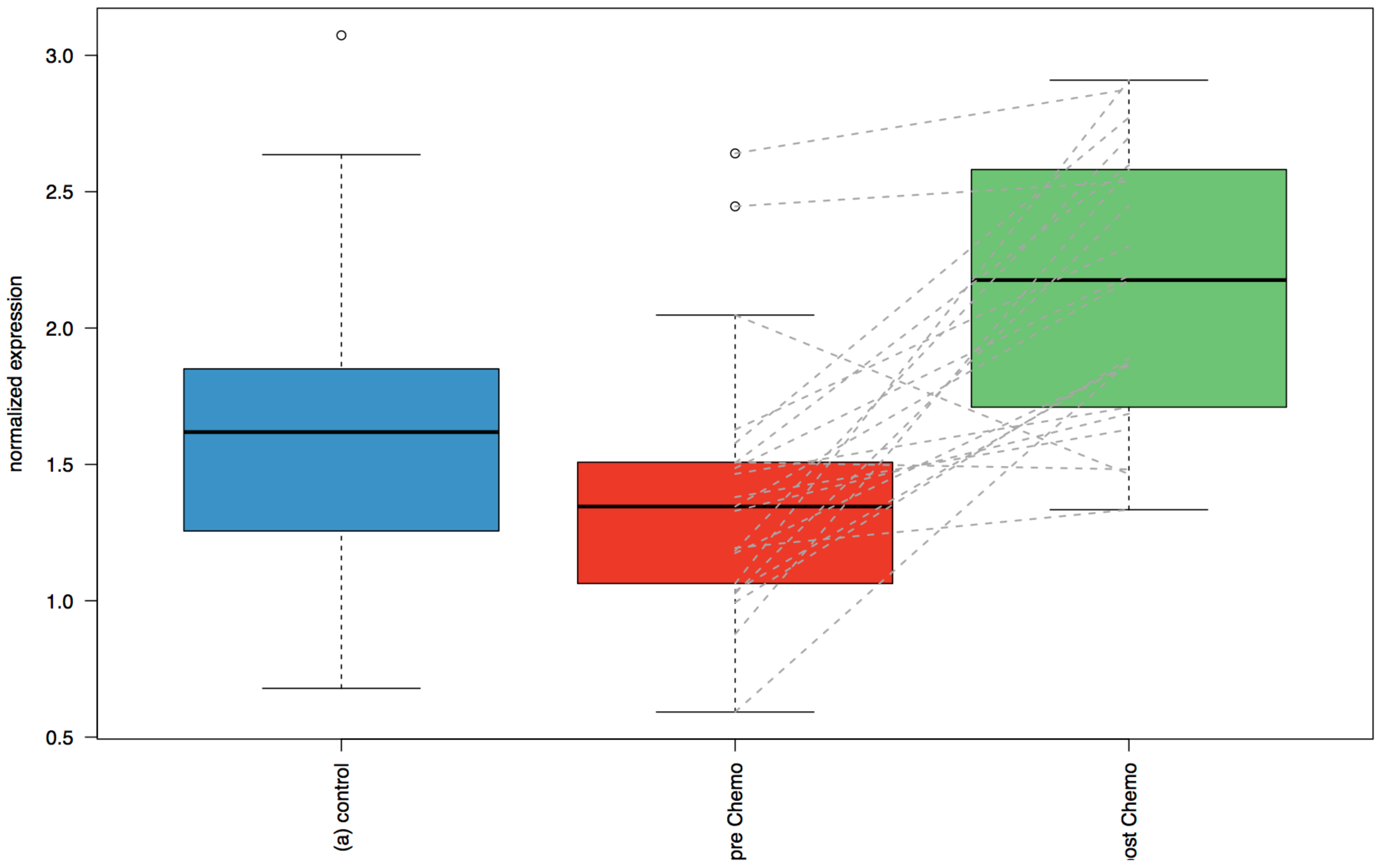
normalized expression
3.0
2.5
2.0
1.5
1.0
0.5
(a) control
pre Chemo
ost Chemo

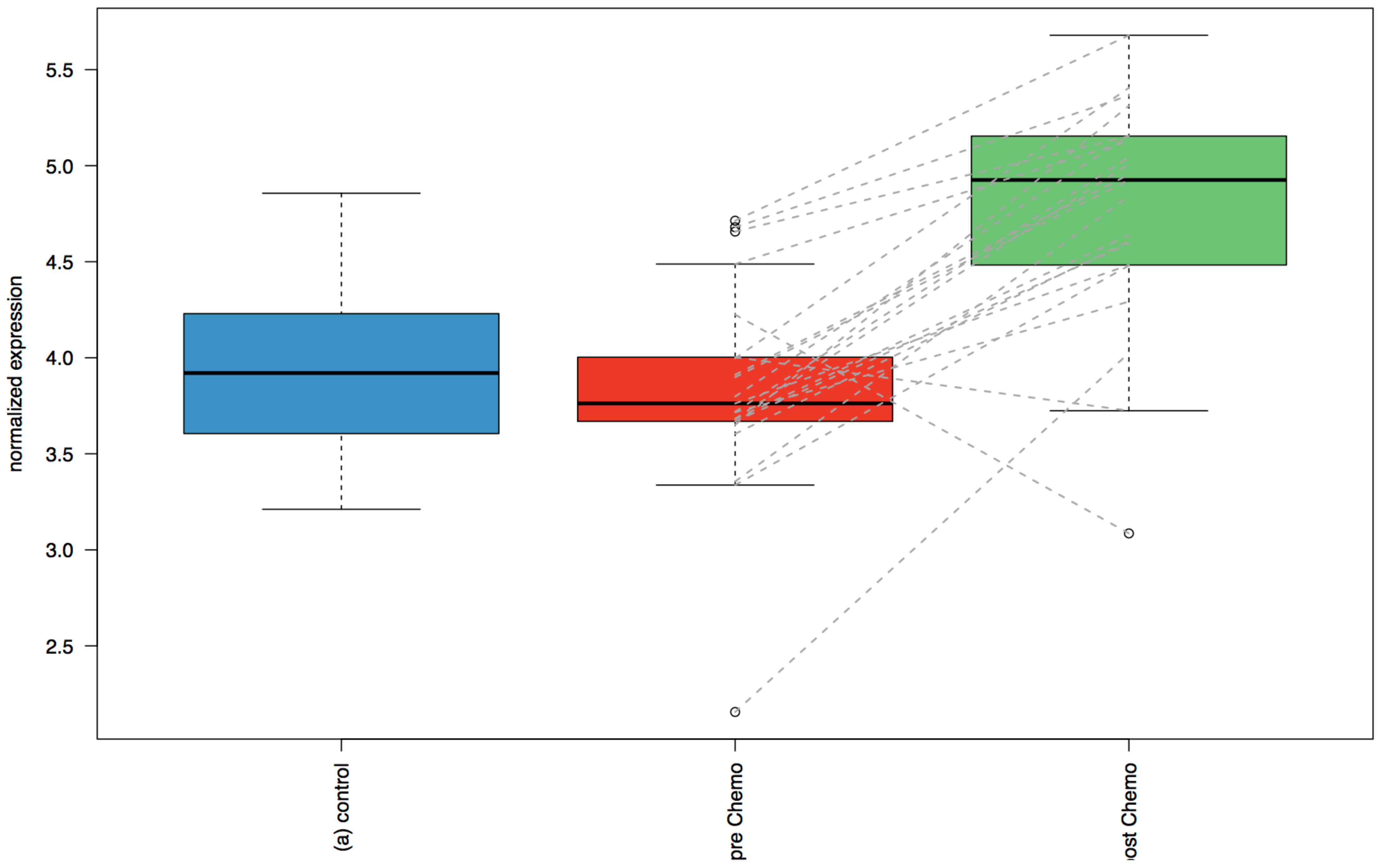
normalized expression
5.5
5.0
4.5
4.0
3.5
3.0
2.5
(a) control
pre Chemo
ost Chemo

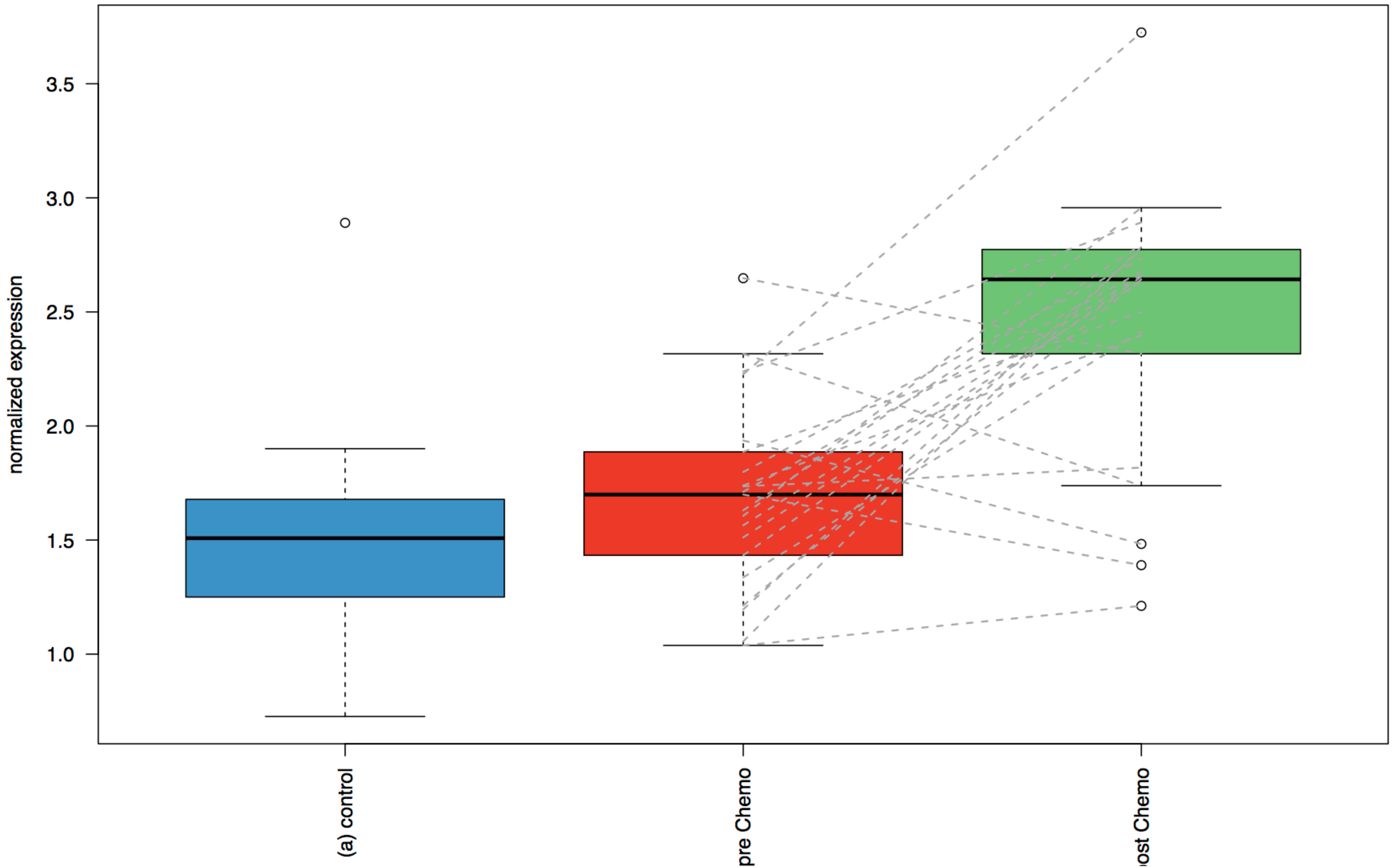
normalized expression
3.5
3.0
2.5
2.0
1.5
1.0
(a) control
pre Chemo
ost Chemo

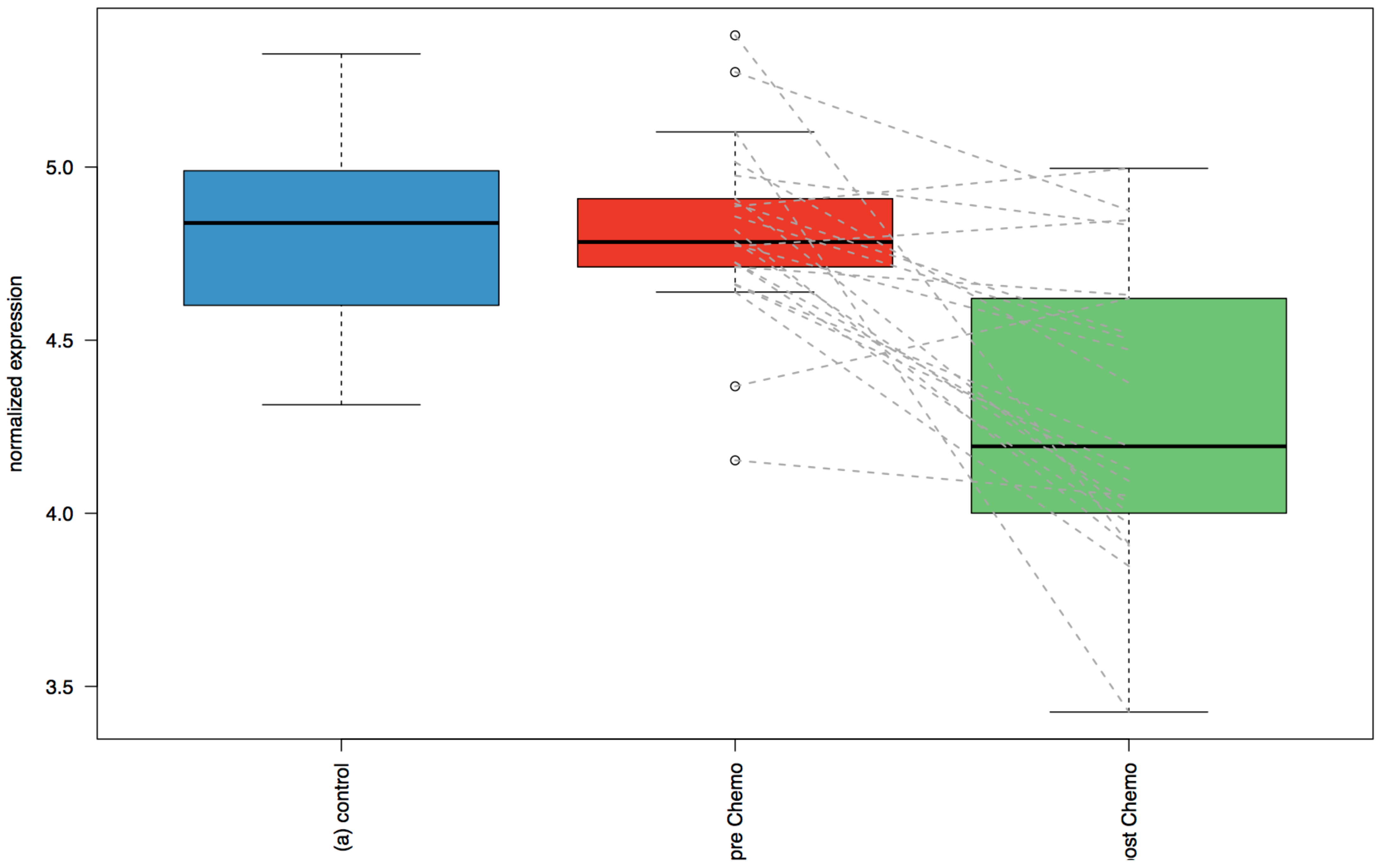

normalized expression
5.0
4.5
4.0
3.5
(a) control
pre Chemo
ost Chemo

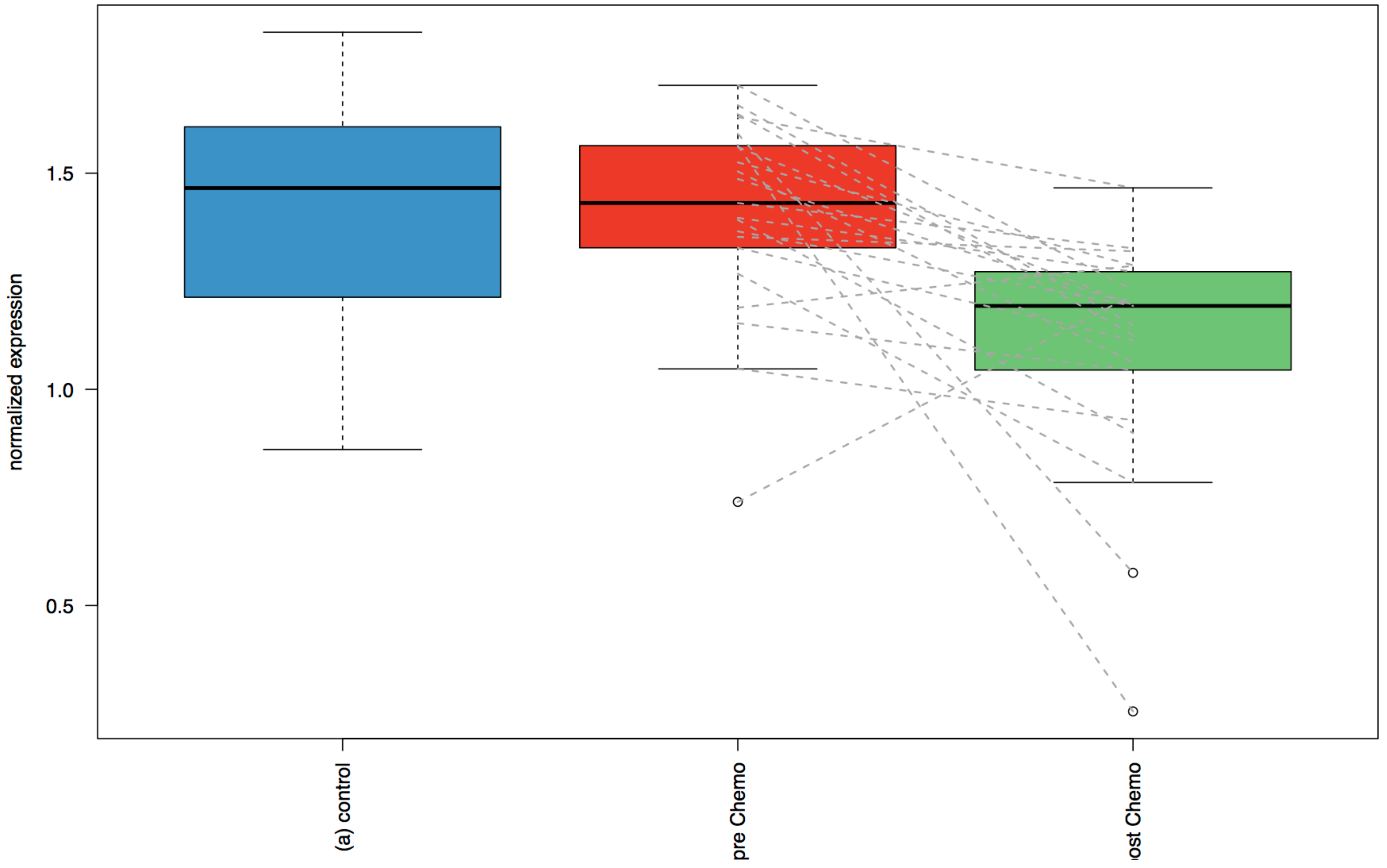

normalized expression
1.5
1.0
0.5
(a) control
pre Chemo
ost Chemo

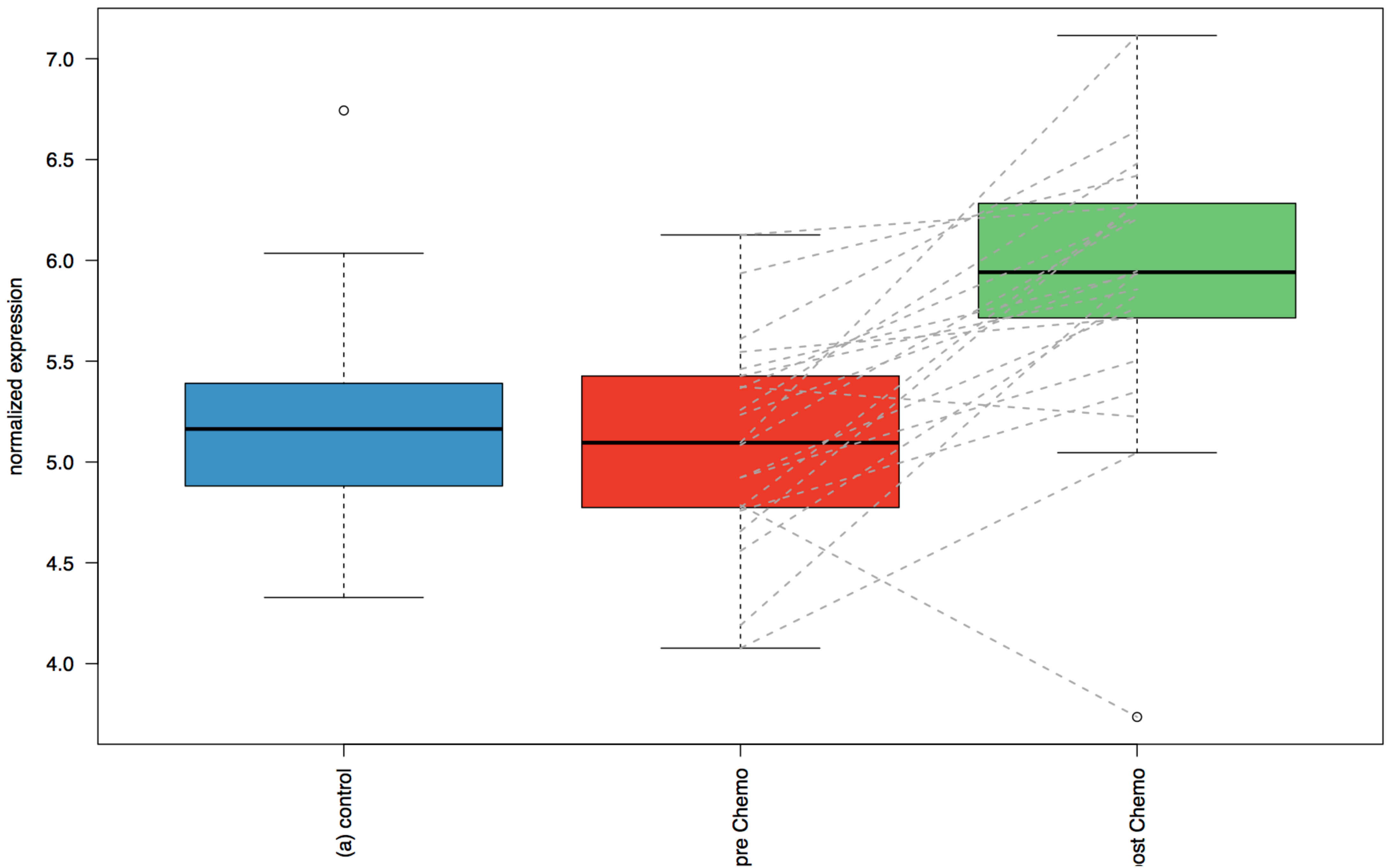

normalized expression
7.0
6.5
6.0
5.5
5.0
4.5
4.0
(a) control
pre Chemo
ost Chemo
